# Gold-Catalyzed “Back-to-Front” Synthesis of 4-Silyloxyindoles

**DOI:** 10.1021/acs.orglett.4c01581

**Published:** 2024-06-05

**Authors:** Miguel A. Muñoz-Torres, Samuel Suárez-Pantiga, Roberto Sanz

**Affiliations:** Área de Química Orgánica, Departamento de Química, Facultad de Ciencias, Universidad de Burgos, Pza. Misael Bañuelos s/n, 09001 Burgos, Spain

## Abstract

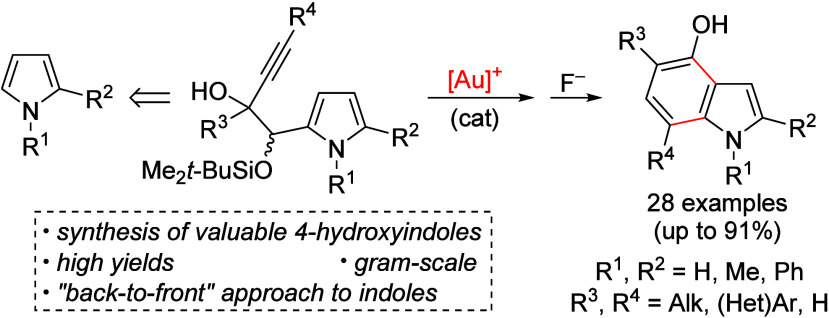

A series of pyrrol-yn-glycol derivatives were easily prepared from simple pyrroles through a three-step sequence involving hydroxyalkylation-alkynylation-*O*-silylation. Their reaction with IPrAuNTf_2_ triggers a C2-pyrrole attack onto the activated alkyne and subsequent highly selective 1,2-migration of the oxyalkyl group in the intermediate spirocycle. This approach enables the efficient synthesis of a wide selection of regioselectively functionalized 4-hydroxyindoles, which represent important yet challenging indole scaffolds, in high yields.

The 4-hydroxyindole motif is widespread in pharmaceuticals, natural products and bioactive molecules,^1^ underscoring its significance across various domains. Due to their potential utility in Pd-catalyzed reactions involving triflates,^2^ C4-oxy-substituted indoles have garnered substantial attention in indole chemistry. However, the C4-position of the indole nucleus exhibits diminished nucleophilicity compared to other positions. Consequently, the synthesis of 4-substituted indoles, particularly 4-hydroxyindole derivatives, predominantly relies on cyclization of regioselectively functionalized nitrogenated aromatics, whose preparation typically involves multistep processes.^3^ Alternatively, particular methods have been reported for activating the C4–H bond in appropriately 3-functionalized indoles.^4^ Therefore, the development of efficient approaches for accessing C4-oxy-substituted indoles remains an appealing yet challenging pursuit.^5^ Additionally, while the prevailing approach for accessing the indole scaffold entails the installation of the pyrrole ring onto a suitable nitrogen-functionalized benzene derivative (“front-to-back” approach), the alternative but less explored “back-to-front” strategy, involving benzannulation of a functionalized pyrrole derivative, offers notable advantages for controlling regioselective functionalization at the C4–C7 positions of the indole core,^6^ as elegantly demonstrated by Maji et al. using allylboronic acids (Scheme 1, eq 1).^6a^

**Scheme 1:**
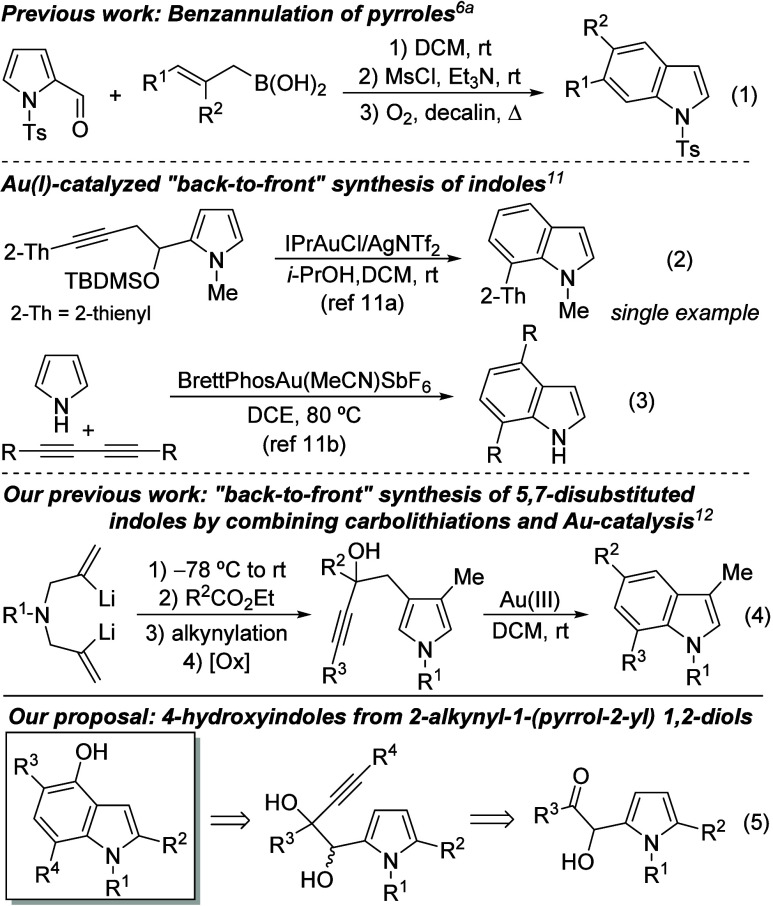
Previous Au-Catalyzed “Back-to-Front” Indole Synthesis and This Work

On the other hand, gold-catalysis has emerged as a versatile tool for constructing diverse carbon- and heterocyclic frameworks,^7^ particularly through the annulation of suitable functionalized alkynes to synthesize various indole skeletons.^8^ However, while benzannulation of indolyl alkynols represents a well-established route to carbazole synthesis,^9^ the analogous benzannulation of alkyne-tethered pyrroles remains underdeve-loped.^10^ To date, only particular examples of Au-catalyzed synthesis of indoles from pyrroles have been reported.^11^ Hashmi presented a single example of a 7-substituted indole from a 2-alkynylpyrrole (Scheme 1, eq 2), while Ohno and Fujii described the double hydroarylation of pyrroles with 1,3-diynes to yield 4,7-disubstituted indoles (eq 3). Recently, we have taken advantage of carbolithiation reactions to access to pyrrolyl alkynols, which upon Au-catalyzed cyclization, afford 5,7-disubstituted indoles (eq 4).^12^ At this point, we envisioned that elusive 4-hydroxyindoles could be synthesized via the benzannulation of 2-alkynyl-1-(pyrrol-2-yl) 1,2-diols, which would be derived from pyrrolyl α-acyloins (Scheme 1, eq 5).

To synthesize the required pyrrolyl diol derivatives **1** and **2**, we utilized the hydroxyalkylation of pyrroles with glyoxals. We followed the procedure described by Ren et al., involving the reaction of pyrroles with arylglyoxals in the presence of HFIP as a promoter.^13^ The readily obtained pyrrol-2-yl α-acyloins **S1a-j** were then treated with alkynyl organometallics to produce pyrrol-2-yl glycols **1**. Alternatively, the heteroaryl α-acyloins **S1** were *O*-protected, providing *O*-silyl acyloins **S2–4**. Their further alkynylation gave rise to monosilylated pyrrolyl glycols **2–2”**, which could also be obtained through the selective monosilylation of **1** (Scheme 2).^14^

**Scheme 2:**
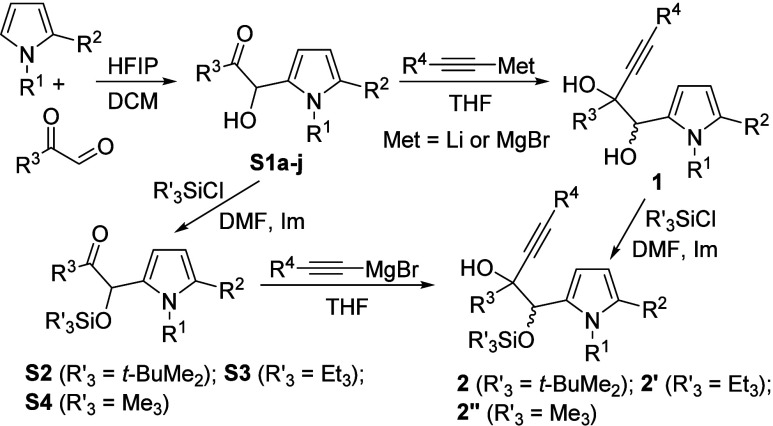
Preparation of Starting Pyrrolyl Diols 1 and 2

Pyrrolyl glycol **1aa** was chosen as the model substrate to assess its reactivity under various gold complexes (Scheme 3). After exhaustive screening,^14^ two catalytic systems were selected although they exhibited only moderate selectivity, yielding two distinct hydroxyindole derivatives, 4-hydroxyindole **3aa** and 5-hydroxyindole **4aa**. Both compounds could stem from an initial C-2 pyrrole attack onto the activated alkyne, leading to a common intermediate **A** that could evolve through a 1,2-hydroxyalkyl shift, eventually producing **3aa**. However, the formation of 5-hydroxyindole **4aa** suggests a pinacol rearrangement before protodeauration. Not unexpectedly, the 7-hydroxyindole **5aa**, derived from a competitive 1,2-alkenyl migration in **A**, was not detected.^11a^ Furthermore, the homopropargyl alcohol moiety in **1aa** could potentially undergo a hydroxyl attack onto the alkyne, ultimately producing furan derivative **6aa**, which was observed in trace amounts (Scheme 3). Despite obtaining only moderate selectivity with both catalytic systems, IPrAuNTf_2_ and SPhosAu(MeCN)SbF_6_, a brief study was conducted with selected pyrrolyl diols **1ab,af,ah** (Scheme 3). Similar selectivity trends were observed for the tested substrates with each gold complex, except for the butyl-substituted **1ah**, which yielded a higher amount of furan derivative **6ah**. Consequently, 4-hydroxyindoles **3** and 5-hydroxyindoles **4** could be isolated only in moderate yields.

**Scheme 3:**
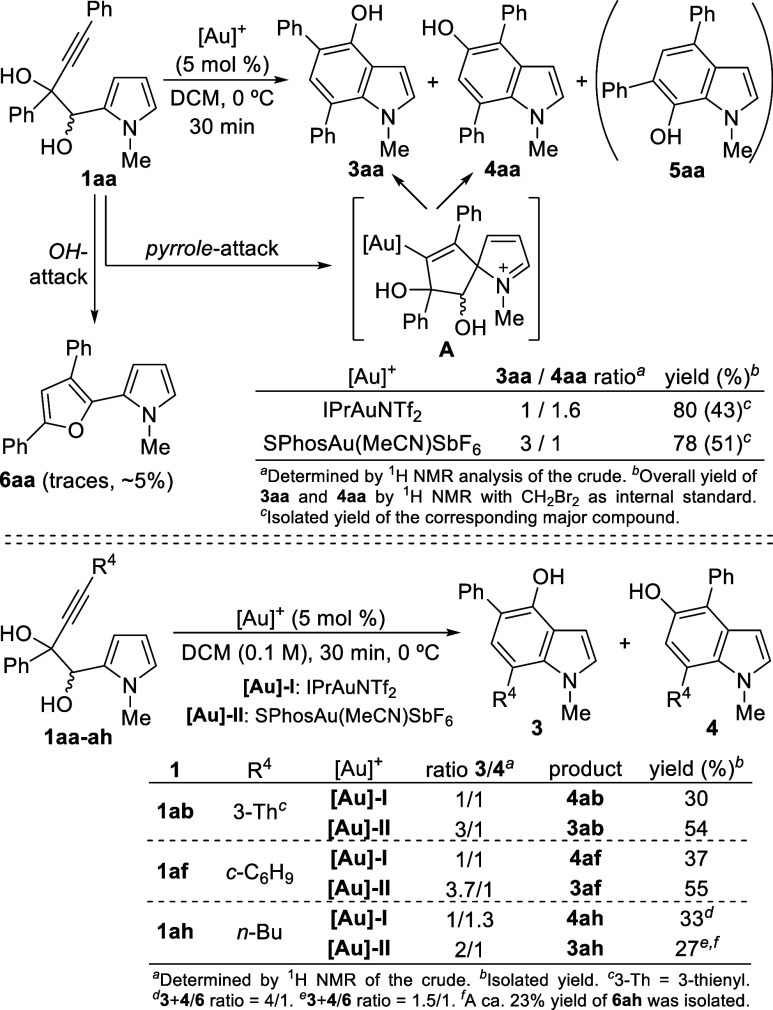
Gold-Catalyzed Cyclization of Glycols 1aa-ah

To prevent the competitive formation of 5-hydroxyindoles **4** and furans **6**, we aimed to employ *O*-silylated pyrrolyl glycols **2** (Scheme 4). Initially, the effect of the trialkylsilyl group was investigated using **2aa**, **2’aa** and **2”aa** as starting substrates, with IPrAuNTf_2_ as the catalyst. This resulted in the generation of a mixture of 4-silyloxyindoles **7** and 7-silyloxyindoles **8**, with the best selectivity and yield achieved using the bulkier TBDMS-protected diol **2aa**. Subsequently, the effect of the gold(I) catalyst on the selectivity was evaluated using **2aa**, both the counteranion^15^ and ligand was studied, showing no significant improvement compared to IPrAuNTf_2_.^14^ However, encouragingly, the increase of the reaction dilution to 0.01 M led to almost complete selectivity toward 4-silyloxyindole **7aa**.^14^

**Scheme 4:**
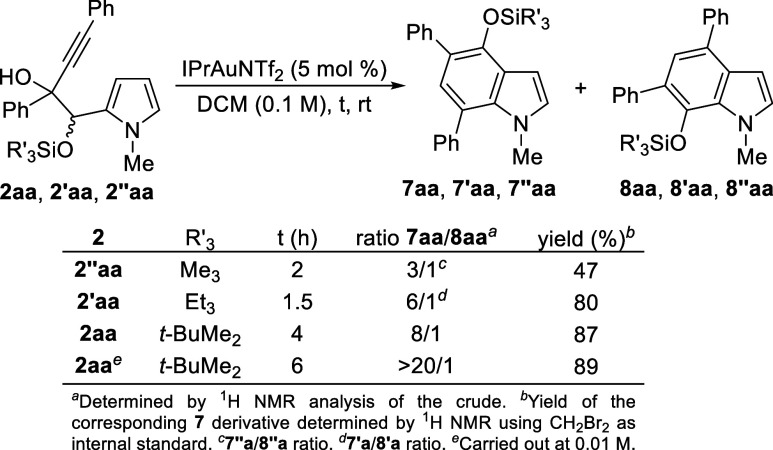
Au-Catalyzed Benzannulation of 2aa, 2’aa and 2”aa

With the optimized conditions in hand, the scope of this procedure for the preparation of 4-silyloxyindoles **7** was studied (Table 1). First, we reduced the catalyst loading to 3 mol % without compromising process efficiency, allowing the isolation of model **7aa** in 84% yield (entry 1). Then, the impact of the alkyne substituent (R^4^) was investigated using a wide range of *O*-monosilylated pyrrolyl diols **2aa-ak** (entries 1–11). These glycols efficiently underwent cyclization, yielding the corresponding silyloxyindoles **7** with high to almost complete selectivity in most cases (entries 1–7). Slightly lower regioselectivity was obtained for (*c*)-alkyl-substituted alkynes (entries 8–10), with terminal alkyne **2ak** showing the most significant effect (entry 11). Next, the influence of the propargylic substituent (R^3^) was examined using (hetero)aryl- and alkenyl-substituted starting alkynes (entries 12–21). With aromatic groups possessing diverse electronic and steric properties, as well as a heteroaromatic or a methyl group as R^3^, almost complete selectivity was observed, providing the corresponding 4-silyloxyindoles **7** in good to high yields (entries 12–19). A slight decrease in selectivity was noted with *o*-tolyl and 3-thienyl groups as the propargyl substituents (entries 15 and 16). Notably, with a secondary propargylic alcohol substrate (R^3^ = H) the benzannulation occurred with complete regioselectivity, accompanied by in situ desilylation leading to 4-hydroxyindoles **3ga** and **3gb** (entries 20 and 21). Finally, different R^1^ and R^2^ substituents on the pyrrole ring were evaluated (entries 22–28). Remarkably, *N*H substrates derived from pyrrolyl α-acyloins **2h,i** exhibited enhanced regioselectivity in the benzannulation reaction, providing access to 4-silyloxy-1*H*-indoles **7ha**-**hh** and **7ia**-**ib** in high yields (entries 22–26). Furthermore, *N*-phenyl-4-silyloxyindoles **7ja,jh** were obtained with comparable efficiency (entries 27 and 28), demonstrating enhanced selectivity in the case of **2jh** bearing a butyl-substituted alkyne (entry 28 vs 8).

**Table 1:**
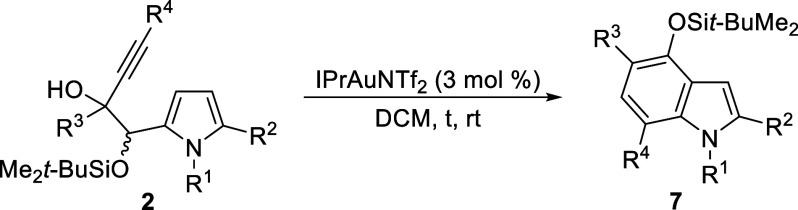
Synthesis of 4-Silyloxyindoles 7^a^

entry	**2**	R^1^	R^2^	R^3^	R^4^	t (h)	product	r.r.^b^**7**/**8**	yield (%)^c^
1	**2aa**	Me	H	Ph	Ph	6	**7aa**	>20/1^d^	84
2	**2ab**	Me	H	Ph	3-Th	6	**7ab**	>20/1^d^	83
3	**2ac**	Me	H	Ph	4-MeOC_6_H_4_	6	**7ac**	>20/1^d^	80
4	**2ad**	Me	H	Ph	4-ClC_6_H_4_	6	**7ad**	16/1	78
5	**2ae**	Me	H	Ph	2-MeC_6_H_4_	6	**7ae**	10/1	90^e^
6	**2af**	Me	H	Ph	*c*-C_6_H_9_	4	**7af**	>20/1^d^	66
7	**2ag**	Me	H	Ph	C(Me)=CH_2_	5	**7ag**	>20/1^d^	69
8	**2ah**	Me	H	Ph	*n*-Bu	6	**7ah**	5/1 (8/1)^f^	81^g^
9	**2ai**	Me	H	Ph	*c*-C_3_H_5_	6	**7ai**	6/1	72
10^h^	**2aj**	Me	H	Ph	*c*-C_6_H_11_	16	**7aj**	5/1	74^g^
11	**2ak**	Me	H	Ph	H	3	**7ak**	3/1	61
12	**2ba**	Me	H	4-FC_6_H_4_	Ph	6	**7ba**	>20/1^d^	88
13	**2bf**	Me	H	4-FC_6_H_4_	*c*-C_6_H_9_	6	**7bf**	>20/1^d^	62
14	**2ca**	Me	H	4-MeOC_6_H_4_	Ph	5	**7ca**	>20/1	87
15^h^	**2da**	Me	H	2-MeC_6_H_4_	Ph	16	**7da**	8/1	48^i^^,^^j^
16	**2ea**	Me	H	3-Th	Ph	8	**7ea**	6/1 (10/1)^f^	91^k^
17	**2fa**	Me	H	Me	Ph	8	**7fa**	17/1^d^	90
18	**2fb**	Me	H	Me	3-Th	6	**7fb**	16/1^d^	74
19	**2ff**	Me	H	Me	*c*-C_6_H_9_	7	**7ff**	14/1^d^	66^g^
20	**2ga**	Me	H	H	Ph	8	**3ga**	>20/1^d^	64
21^h^	**2gb**	Me	H	H	3-Th	16	**3gb**	>20/1^d^	50
22	**2ha**	H	H	Ph	Ph	2	**7ha**	>20/1	84
23	**2hb**	H	H	Ph	3-Th	2	**7hb**	>20/1	83
24	**2hh**	H	H	Ph	*n*-Bu	2	**7hh**	10/1	85
25^h^	**2ia**	H	Me	Ph	Ph	16	**7ia**	13/1	63
26^h^	**2ib**	H	Me	Ph	3-Th	16	**7ib**	15/1	55
27	**2ja**	Ph	H	Ph	Ph	4	**7ja**	>20/1	80
28	**2jh**	Ph	H	Ph	*n*-Bu	4	**7jh**	>20/1	81

a Reaction conditions: **2** (0.3 mmol), IPrAuNTf_2_ (3 mol %), in DCM (30 mL) at rt.

b Determined by ^1^H NMR of the crude reaction mixture.

c Isolated yield of **7** after column chromatography.

d Slightly lower regioselectivity (8–17/1) was observed when the reactions were conducted at 0.1 M.

e Isolated as a 10/1 mixture of regioisomers.

f Carried out at 0.005 M. An additional catalyst loading and 16 h were required.

g Isolated as a 14/1 mixture of regioisomers.

h An additional catalyst loading (3 mol %) was added.

i Lower yield due to impurities in the starting substrate **2da**.

j Isolated as a 8/1 mixture of regioisomers.

k Isolated as a 6/1 mixture of regioisomers.

Regarding the mechanism (Scheme 5),^16^ the initial step would involve the pyrrole ring attacking the activated alkyne, forming intermediate **A**, which subsequently undergoes an oxyalkyl 1,2-shift to yield intermediate **B**.^11a,17^ This shift is favored over a competitive 1,2-alkenyl migration leading to intermediate **C**. Protodeauration and loss of water from **B** result in the formation of 4-oxyindole derivatives **3** and **7**. If a pinacol rearrangement occurs before protodeauration, via intermediates **D** and **E**, the 5-hydroxyindoles **4** are obtained. The formation of 7-oxyindoles **8**, obtained competitively but in minor amounts from alkynols with R^4^ = H and (*c*)-Alk, is proposed to involve a 1,2-alkenyl migration from **A** to **C**. However, a more likely scenario is a C3-pyrrole attack directly yielding intermediate **C**. This counterintuitive C3-pyrrole activation has been previously postulated and supported by DFT in the Ag(I)-catalyzed synthesis of indoles from pyrrol-3-yl ynols.^18^ Moreover, the formation of pyrrolyl furans **6** may be explained by a competitive homopropargylic OH-attack onto the corresponding activated alkyne **1-[Au]**. Despite the easiness of the heterocyclodehydration reaction of 3-yne-1,2-diols,^19^ in our scenario, the pyrrole attack is favored. Notably, the high regioselectivity observed for most substrates **2** can be rationalized by considering both the C2-pyrrole attack, which is favored due to its more nucleophilic character, and the greater migratory ability of the oxyalkyl group over the alkenyl one, because of its enhanced ability to stabilize a positive charge in the transition state.^11a,17^

**Scheme 5:**
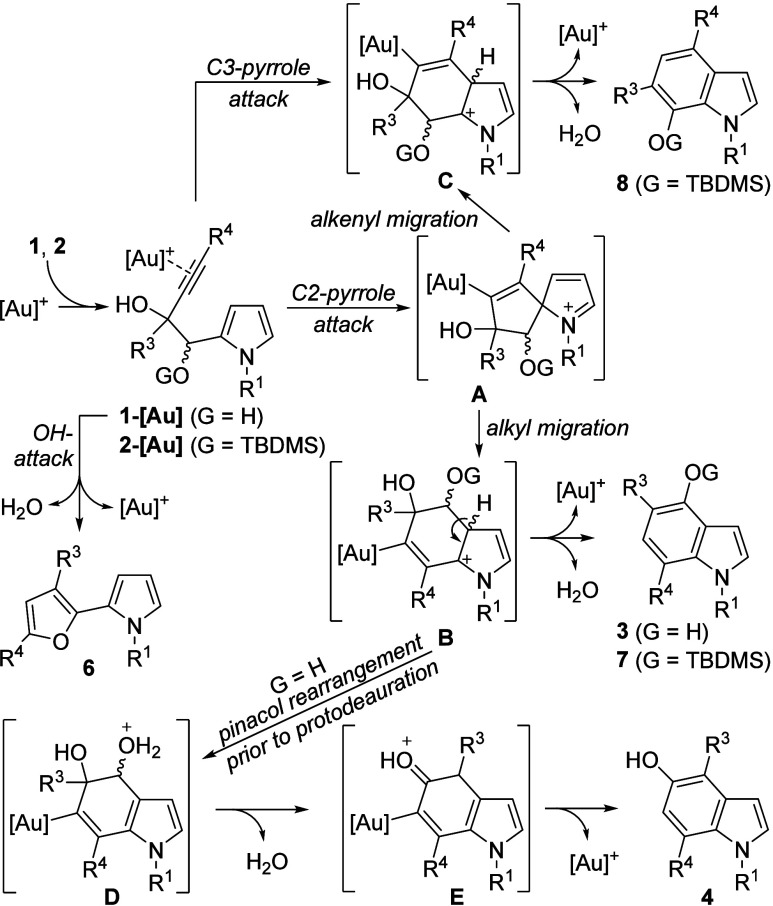
Mechanistic Proposal

To display the practical utility of our methodology, we conducted a scale-up experiment with 2 mmol of **2aa**, at 0.04 M and using 3 mol % of catalyst, which yielded 662 mg of **7aa** (80% yield). As expected, the silyl protecting group could be readily removed using TBAF to deliver a wide variety of 4-hydroxyindoles **3** in high yields (Scheme 6).^14^ Furthermore, the heterocyclic nucleus in indole **7ba** could be reduced to the corresponding indoline **9** using NaBH_3_CN.^20^ Lastly, C-3 functionalization via Bro̷nsted acid-catalyzed direct nucleophilic substitution with alcohols was explored with **7jh** resulting in the formation of 3-functionalized-4-silyloxyindole **10** (Scheme 6).^21^

**Scheme 6:**
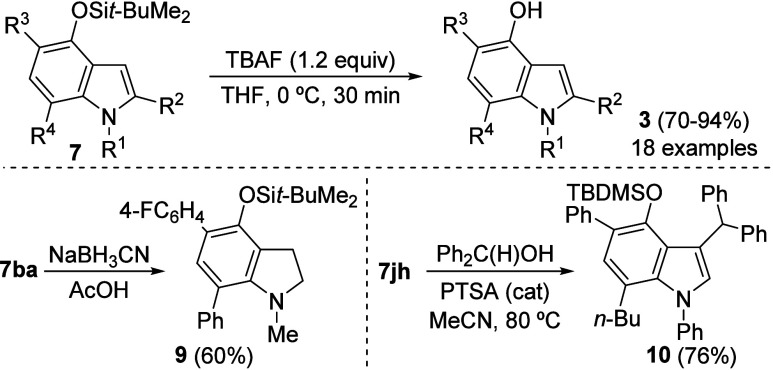
Further Transformations of 4-Silyloxyindoles 7: Synthesis of 4-Hydroxyindoles 3, Indoline 9 and Indole 10

In summary, we have developed an efficient and versatile synthetic route for obtaining 4-hydroxyindole derivatives with additional substituents regioselectively located at the benzenoid ring and C-2 position. Key highlights of this methodology include precise control over the regioselectivity of the cyclization, use of readily available starting materials and catalysts, broad substrate scope for base-insensitive substituents, scalability, straightforward access to elusive 4-oxygenated indole scaffolds, and late-stage synthetic transformations.

## Data Availability

The data underlying this study are available in the published article and its Supporting Information.

## Supporting Information Available

Experimental details, characterization data, and NMR spectra (PDF)
FAIR data, including the primary NMR FID files, for compounds **S1b-j**, **S2a-j**, **S3a**, **S4a**, **1aa-jh**, **2aa-jh**, **2’aa, 2”aa**, **3aa-ia**, **4aa-ah**, **6ah**, **7aa-jh**, **7’aa**, **7”aa**, **9**, **10** (ZIP)
