# Supplementary material for: Gold-Catalyzed “Back-to-Front” Synthesis of 4-Silyloxyindoles
Source: Org Lett. 2024 Jun 5;26(23):4969–74. doi: 10.1021/acs.orglett.4c01581 (PMC11187626; doi:10.1021/acs.orglett.4c01581)

Gold-Catalyzed "Back-to-front" Synthesis of 4-Silyloxyindoles

Miguel A. Muñoz-Torres, Samuel Suárez-Pantiga, and Roberto Sanz*

Área de Química Orgánica, Departamento de Química, Facultad de Ciencias, Universidad de Burgos,

Pza. Misael Bañuelos s/n, 09001-Burgos, Spain

e-mail: rsd@ubu.es

Supporting Information

Index

General information S1

Synthesis and characterization data of starting materials **1** and **2** S1

General procedure A for the synthesis of acyloins **S1** S1

General procedure B for the synthesis of protected acyloins **S2**-**S4** S3

General procedure C for the synthesis of alkynols **1** S7

General procedures D for the synthesis of alkynols **2** S11

Gold-catalyzed cyclization of glycol **1aa** S24

Optimization studies S24

Synthesis and characterization data of 4-hydroxyindoles **3** and 5-hydroxyindoles **4** S26

General procedure E for the gold-catalyzed cyclization of diols **1** S26

Gold-catalyzed cyclization of **2aa** S29

Optimization studies S29

Synthesis and characterization data of 4-silyloxyindoles **7** S31

General procedure F for the gold-catalyzed cyclization of **2** S31

Synthesis and characterization data of 4-hydroxyindoles **3** S42

General procedure G for the synthesis of 4-hydroxyindoles **3** from 4-silyloxyindoles **7** S42

Synthesis and characterization data of indoline **9** S47

Synthesis and characterization data of C3-functionalized indole **10** S47

Scale-up synthesis of **7aa** S48

^{1}H, ^{13}C and selected NOE 1D NMR spectra S49

General information

General methods: All common reagents and solvents were obtained from commercial suppliers and used without any further purification. TLC was performed on aluminum-backed plates coated with silica gel 60 with F_{254} indicator; the chromatograms were visualized under ultraviolet light and/or by staining with a Ce/Mo reagent and subsequent heating. NMR spectra were measured on Varian Mercury-Plus 300 MHz, Bruker Avance 300 MHz and Bruker Avance 500 MHz spectrometers. ^{1}H NMR: splitting pattern abbreviations are: s, singlet; br s, broad singlet; d, doublet; t, triplet; q, quartet; dd, double doublet; ddd, doublets of doublets of doublets; ddt, double doublet of triplets; dt, doublet of triplets; dq, doublet of quartets; td, triplet of doublets; qd, quartet of doublets; quin, quintuplet; hept, heptet; ad, apparent doublet; at, apparent triplet; aq, apparent quartet; m, multiplet; the chemical shifts are reported in ppm using residual solvent peak as reference. ^{13}C NMR spectra were recorded at 75.4 MHz or 125.7 MHz using broadband proton decoupling, and chemical shifts are reported in ppm using residual solvent peaks as reference ($CDCl_3$: δ 77.16; acetone-d_6: δ 29.84) and the multiplicities were determined by DEPT experiments. High-resolution mass spectra (HRMS) were recorded on a LC-MS instrument (1260 Infinity, Agilent) equipped with a QTOF analyzer using ESI (+). Low-resolution mass spectra (LRMS) measurements were recorded on an Agilent 6890N/5973 Network GC System, equipped with an HP-5MS column. Melting points were measured on a Gallenkamp apparatus using open capillary tubes.

Synthesis and characterization data of starting materials 1 and 2

General procedure A for the synthesis of acyloins S1[1]

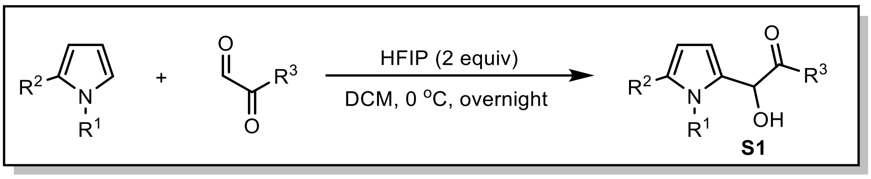

A mixture of the corresponding pyrrole (20 mmol, 1 equiv), glyoxal (24 mmol, 1.2 equiv) and HFIP (2 equiv) in CH_2Cl_2 (80 mL, 0.25 M) was stirred at 0 °C overnight. Then, H_2O (40 mL) was added. The organic phase was separated, and the aqueous phase was extracted with CH_2Cl_2 (2 × 20 mL). The combined organic layers were washed with H_2O and brine, dried over anhydrous Na_2SO_4 and the solvents were removed under reduced pressure. The residue was purified by flash column chromatography using mixtures of hexane and EtOAc as eluents to obtain the corresponding acyloins **S1**, whose spectroscopic data are shown below. The acyloins **S1a**,**h**,**i** were also successfully synthesized, and NMR data matches with those found in the literature.[1]

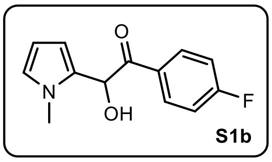

1-(4-Fluorophenyl)-2-hydroxy-2-(1-methyl-1*H*-pyrrol-2-yl)ethan-1-one (S1b): Following the general procedure A with 1-methyl-1*H*-pyrrole (1.8 mL, 20 mmol) and 4-fluorophenylglyoxal hydrate (4.08 g, 24 mmol), the crude product was purified by recrystallization (Et_2O/DCM, 5/1), affording pure S**1b**

[1] Yang J.; Liu, S.; Gui, J.; Xiong, D.; Li, J.; Wang, Z.; Ren, J. *J. Org. Chem.* **2022**, *87*, 6352–6361.

as a pale pink solid (3.41 g, 73%); mp = 140–142 °C; R_f = 0.27 (hexane/EtOAc, 3/1). **^{1}H NMR** (300 MHz, $CDCl_3$) δ (ppm): 8.00–7.88 (m, 2H), 7.14–7.00 (m, 2H), 6.65–6.57 (m, 1H), 6.01–5.94 (m, 2H), 5.90 (dd, J = 3.6, 1.7 Hz, 1H), 4.12 (d, J = 6.2 Hz, 1H), 3.68 (s, 3H). **^{13}C NMR** (75.4 MHz, $CDCl_3$) δ (ppm): 196.6 (C), 166.1 (d, $^1J_{C-F}$ = 256.6 Hz, C), 131.9 (d, $^3J_{C-F}$ = 9.5 Hz, 2 × CH), 130.3 (d, $^4J_{C-F}$ = 3.2 Hz, C), 129.2 (C), 124.5 (CH), 116.0 (d, $^2J_{C-F}$ = 22.0 Hz, 2 × CH), 110.2 (CH), 107.4 (CH), 69.0 (CH), 34.2 (CH_3). **HRMS** (ESI-TOF): calculated for $C_{13}H_{12}NO_2FNa^+$ $[M+Na]^+$ 256.0744; found 256.0753.

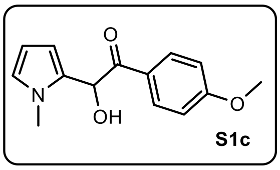

2-Hydroxy-1-(4-methoxyphenyl)-2-(1-methyl-1*H*-pyrrol-2-yl)ethan-1-one (S1c): Following the general procedure A with 1-methyl-1*H*-pyrrole (1.8 mL, 20 mmol) and 4-methoxyphenylglyoxal hydrate (4.37 g, 24 mmol), the crude product was purified by column chromatography (hexane/EtOAc, 2/1), affording pure **1c** as a pale yellow solid (3.73 g, 76%); mp = 115–117 °C; R_f = 0.32 (hexane/EtOAc, 2/1). **^{1}H NMR** (300 MHz, $CDCl_3$) δ (ppm): 7.97–7.86 (m, 2H), 6.96–6.82 (m, 2H), 6.61 (dd, J = 2.7, 1.8 Hz, 1H), 6.01 (dd, J = 3.7, 2.7 Hz, 1H), 5.98–5.92 (m, 2H), 4.41–4.25 (m, 1H), 3.82 (s, 3H), 3.68 (s, 3H). **^{13}C NMR** (75.4 MHz, $CDCl_3$) δ (ppm): 196.4 (C), 164.1 (C), 131.5 (2 × CH), 130.0 (C), 126.7 (C), 124.1 (CH), 113.9 (2 × CH), 109.9 (CH), 107.3 (CH), 68.9 (CH), 55.5 (CH_3), 34.2 (CH_3). **HRMS** (ESI-TOF): calculated for $C_{14}H_{15}NNaO_3^+$ $[M+Na]^+$ 269.0977; found 269.0987.

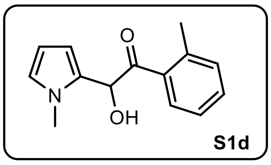

2-Hydroxy-2-(1-methyl-1*H*-pyrrol-2-yl)-1-(*o*-tolyl)ethan-1-one (S1d): Following the general procedure A with 1-methyl-1*H*-pyrrole (1.8 mL, 20 mmol) and *o*-tolylglyoxal (3.55 g, 24 mmol), the crude product was purified by column chromatography (hexane/EtOAc, 3/1), affording pure **S1c** as a pale pink solid (1.83 g, 40%); mp = 121–123 °C ; R_f = 0.34 (hexane/EtOAc, 3/1). **^{1}H NMR** (300 MHz, $CDCl_3$) δ (ppm): δ 7.56 (dd, J = 7.7, 1.5 Hz, 1H), 7.42–7.36 (m, 1H), 7.33–7.26 (m, 1H), 7.23–7.17 (m, 1H), 6.60 (at, J = 2.2 Hz, 1H), 6.08–5.91 (m, 2H), 4.27–4.23 (m, 1H), 3.69 (s, 3H), 2.60 (s, 3H). **^{13}C NMR** (75.4 MHz, $CDCl_3$) δ (ppm): 200.7 (C), 139.5 (C), 134.1 (C), 132.21 (CH), 132.16 (CH), 129.2 (CH), 129.1 (C), 125.7 (CH), 124.1 (CH), 109.5 (CH), 107.3 (CH), 69.8 (CH), 34.1 (CH_3), 21.4 (CH_3). **HRMS** (ESI-TOF): calculated for $C_{14}H_{15}NNaO_3^+$ $[M+Na]^+$ 252.0995; found 252.1003.

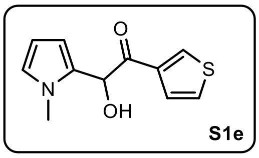

2-Hydroxy-2-(1-methyl-1*H*-pyrrol-2-yl)-1-(thiophen-3-yl)ethan-1-one (S1e): Following the general procedure A with 1-methyl-1*H*-pyrrole (1.8 mL, 20 mmol) and oxo(thiophen-3-yl)acetaldehyde (3.36 g, 24 mmol), the crude product was purified by column chromatography (hexane/EtOAc, 3/1), affording pure **S1e** as an orange oil (3.23 g, 73%); R_f = 0.28 (hexane/EtOAc, 3/1). **^{1}H NMR** (300 MHz, $CDCl_3$) δ (ppm): 7.98–7.90 (m, 1H), 7.51 (d, J = 5.0 Hz, 1H), 7.29 (dd, J = 5.0, 2.9 Hz, 1H), 6.70–6.57 (m, 1H), 6.13 (dd, J = 3.5, 1.7 Hz, 1H), 6.07 (at, J = 3.1 Hz, 1H), 5.75 (d, J = 4.8 Hz, 1H), 4.26–4.16 (m, 1H), 3.61 (s, 3H). **^{13}C NMR** (75.4 MHz, $CDCl_3$) δ (ppm): 192.4 (C), 138.1 (CH), 134.6 (C), 129.1 (CH), 127.6 (CH), 126.4 (CH), 124.7 (CH), 111.0 (CH), 107.6 (CH), 70.6 (CH), 34.3 (CH_3). **HRMS** (ESI-TOF): calculated for $C_{11}H_{12}NO_2S^+$ $[M+H]^+$ 222.0583; found 222.0586.

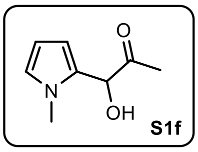

1-Hydroxy-1-(1-methyl-1*H*-pyrrol-2-yl)propan-2-one (S1f): Following the general procedure A with 1-methyl-1*H*-pyrrole (1.8 mL, 20 mmol) and pyruvic aldehyde, (35-45% w/w aq solution, 3.6 mL, 24 mmol), the crude product was purified by column chromatography (hexane/EtOAc, 2/1), affording pure **S1f** as an orange oil (1.68 g, 55%); R_f = 0.37 (hexane/EtOAc, 2/1). **^{1}H NMR** (300 MHz, $CDCl_3$) δ (ppm): 6.64 (t, J = 2.2 Hz, 1H), 6.15–6.07 (m, 2H), 5.20 (s, 1H), 3.84 (bs, 1H), 3.59 (s, 3H), 2.18 (s, 3H). **^{13}C NMR** (75.4 MHz, $CDCl_3$) δ (ppm): 207.1 (C), 127.8 (C), 124.8 (CH), 110.4 (CH), 107.5 (CH), 73.4 (CH), 34.3 (CH_3), 25.8 (CH_3). **HRMS** (ESI-TOF): calculated for $C_8H_{12}NO_2^+$ $[M+H]^+$ 154.0857; found 154.0863.

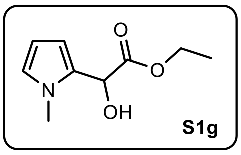

Ethyl 2-hydroxy-2-(1-methyl-1*H*-pyrrol-2-yl)acetate (S1g): Following the general procedure A with 1-methyl-1*H*-pyrrole (1.8 mL., 20 mmol) and ethyl glyoxalate solution (50% w/w solution in toluene, 4.90 mL, 24 mmol), the crude product was purified by column chromatography (hexane/EtOAc, 2/1), affording pure **S1g** as an orange oil (1.10 g, 30%); R_f = 0.35 (hexane/EtOAc, 2/1). **^{1}H NMR** (300 MHz, $CDCl_3$) δ (ppm): 6.63 (at, J = 2.2 Hz, 1H), 6.14–5.99 (m, 2H), 5.22 (d, J = 6.9 Hz, 1H), 4.41–4.21 (m, 2H), 3.68 (s, 3H), 3.26 (bs, 1H), 1.31 (t, J = 7.1 Hz, 3H). **^{13}C NMR** (75.4 MHz, $CDCl_3$) δ (ppm): 172.8 (C), 129.2 (C), 124.1 (CH), 108.4 (CH), 107.0 (CH), 66.4 (CH), 62.1 (CH_2), 34.1 (CH_3), 14.1 (CH_3). **HRMS** (ESI-TOF): calculated for $C_9H_{14}NO_3^+$ $[M+H]^+$ 184.0968; found 184.0968.

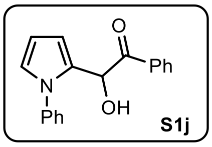

2-Hydroxy-1-phenyl-2-(1-phenyl-1*H*-pyrrol-2-yl)ethan-1-one (S1j): Following the general procedure A with 1-phenyl-1*H*-pyrrole (2.86 g, 20 mmol) and phenylglyoxal hydrate (3.65 g, 24 mmol), after heating to reflux for 2 h to achieve complete conversion, the crude product was purified by column chromatography (hexane/EtOAc, 5/1), affording pure **S1j** as a yellow oil (4.16 g, 75%); R_f = 0.31 (hexane/EtOAc, 5/1). **^{1}H NMR** (300 MHz, $CDCl_3$) δ (ppm): 7.72–7.43 (m, 8H), 7.40–7.27 (m, 2H), 6.99–6.90 (m, 1H), 6.23 (at, J = 3.4 Hz, 1H), 6.17–6.10 (m, 1H), 5.91–5.77 (m, 1H), 4.56–4.31 (m, 1H). **^{13}C NMR** (75.4 MHz, $CDCl_3$) δ (ppm): 197.1 (C), 139.2 (C), 133.6 (C), 133.5 (CH), 130.2 (C), 129.5 (2 × CH), 128.7 (2 × CH), 128.5 (2 × CH), 127.8 (CH), 126.0 (2 × CH), 123.7 (CH), 110.7 (CH), 109.3 (CH), 67.9 (CH). **HRMS** (ESI-TOF): could not be recorded.

General procedure B for the synthesis of protected acyloins S2-S4

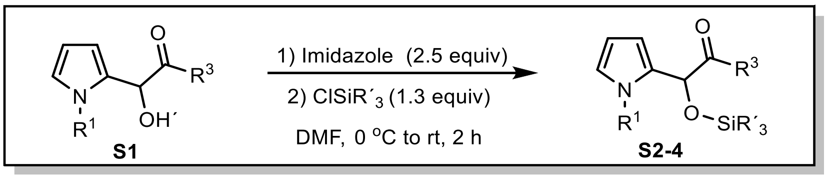

To a stirred solution of the corresponding acyloin **S1** (10 mmol, 1 equiv) in DMF (20 mL, 0.5 M), imidazole (1.70 g, 25 mmol, 2.5 equiv) and the chlorotrialkylsilane (13 mmol, 1.3 equiv) were added at 0 °C. The resulting solution was stirred at rt until **S1** was consumed as determined by GC/MS or TLC (~2 h). Then, H_2O (20 mL) was added and the resulting solution was extracted with CH_2Cl_2 (3 × 20 mL). The combined organic layers were washed with water (4 × 30 mL) to remove DMF, dried over anhydrous Na_2SO_4, and

the solvents were eliminated under reduced pressure. The resulting residue was purified by flash column chromatography using mixtures of hexane and EtOAc as eluents to obtain the corresponding protected acyloins **S2-4**.

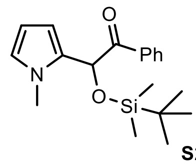

S2

2-((*Tert*-butyldimethylsilyl)oxy)-2-(1-methyl-1*H*-pyrrol-2-yl)-1-phenylethan-1-one (S2): Following the general procedure B with acyloin **S1a** (2.15 g, 10 mmol) and *tert*-butyldimethylchlorosilane (1.96 g, 13 mmol), **S2** was obtained pure without further purification; yellow oil (3.26 g, 99%); R_f = 0.44 (hexane/EtOAc, 10/1). ^{1}**H NMR** (300 MHz, $CDCl_3$) δ (ppm): 8.07–7.97 (m, 2H), 7.54–7.46 (m, 1H), 7.44–7.35 (m, 2H), 6.60–6.53 (m, 1H), 6.14 (dd, *J* = 3.5, 1.7 Hz, 1H), 6.06–5.98 (m, 2H), 3.64 (s, 3H), 0.91 (s, 9H), 0.07 (s, 3H), 0.00 (s, 3H). ^{13}**C NMR** (75.4 MHz, $CDCl_3$) δ (ppm): 197.0 (C), 135.3 (C), 133.0 (CH), 129.3 (2 × CH), 129.1 (C), 128.4 (2 × CH), 124.3 (CH), 110.4 (CH), 107.3 (CH), 73.0 (CH), 34.8 (CH_3), 25.9 (3 × CH_3), 18.4 (C), –4.8 (CH_3), –4.9 (CH_3). **HRMS** (ESI-TOF): calculated for $C_{19}H_{28}NO_2Si^+$ $[M+H]^+$ 330.1884; found 330.1877.

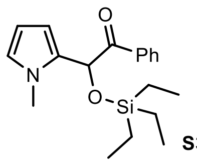

S3

2-(1-Methyl-1*H*-pyrrol-2-yl)-1-phenyl-2-((triethylsilyl)oxy)ethan-1-one (S3): Following the general procedure B with acyloin **S1a** (2.15 g, 10 mmol) and chlorotriethylsilane (1.96 g, 13 mmol) the crude product was purified by column chromatography (hexane/EtOAc, 20/1), affording pure **S3** as a yellow oil (2.44 g, 74%); R_f = 0.30 (hexane/EtOAc, 20/1). ^{1}**H NMR** (300 MHz, $CDCl_3$) δ (ppm): 8.10–7.96 (m, 2H), 7.56–7.35 (m, 3H), 6.62–6.53 (m, 1H), 6.20–6.14 (m, 1H), 6.11–6.03 (m, 2H), 3.67 (s, 3H), 1.02–0.84 (m, 9H), 0.73–0.55 (m, 6H). ^{13}**C NMR** (75.4 MHz, $CDCl_3$) δ (ppm): 196.9 (C), 135.3 (C), 133.0 (CH), 129.2 (C), 129.1 (2 × CH), 128.4 (2 × CH), 124.2 (CH), 110.4 (CH), 107.3 (CH), 72.6 (CH), 34.7 (CH_3), 6.8 (3 × CH_3) 4.9 (3 × CH_2). **HRMS** (ESI-TOF): calculated for $C_{19}H_{28}NO_2Si^+$ $[M+H]^+$ 330.1884; found 330.1887.

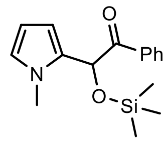

S4

2-(1-Methyl-1*H*-pyrrol-2-yl)-1-phenyl-2-((trimethylsilyl)oxy)ethan-1-one (S4): Following the general procedure B with acyloin **S1a** (2.15 g, 10 mmol) and chlorotrimethylsilane (1.65 mL, 13 mmol), the crude product was purified by column chromatography employing deactivated silica-gel (hexane/EtOAc, 25/1), affording pure **S4** as a yellow oil (0.98 g, 34%); R_f = 0.17 (hexane/EtOAc, 25/1). ^{1}**H NMR** (300 MHz, $CDCl_3$) δ (ppm): 8.02–7.96 (m, 2H), 7.54–7.46 (m, 1H), 7.44–7.35 (m, 2H), 6.62–6.55 (m, 1H), 6.14–6.07 (m, 2H), 6.03 (at, *J* = 3.1 Hz, 1H), 3.65 (s, 3H), 0.12 (s, 9H). ^{13}**C NMR** (75.4 MHz, $CDCl_3$) δ (ppm): 196.8 (C), 135.3 (C), 133.0 (CH), 129.0 (2 × CH), 128.6 (C), 128.4 (2 × CH), 124.3 (CH), 110.7 (CH), 107.3 (CH), 72.0 (CH), 34.5 (CH_3), 0.17 (3 × CH_3). **HRMS** (ESI-TOF): calculated for $C_{16}H_{21}NNaO_2Si^+$ $[M+Na]^+$ 310.1234; found 310.1242.

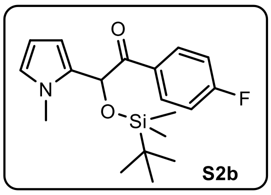

S2b

2-((*Tert*-butyldimethylsilyl)oxy)-1-(4-fluorophenyl)-2-(1-methyl-1*H*-pyrrol-2-yl)ethan-1-one (S2b): Following the general procedure B with acyloin **S1b** (2.33 g, 10 mmol) and *tert*-butyldimethylchlorosilane (1.96 g, 13 mmol), the crude product was purified by column chromatography (hexane/EtOAc, 10/1), affording pure **S2b** as a pale pink solid (2.92 g, 84%); mp = 133–135 °C; R_f = 0.40 (hexane/EtOAc, 10/1). **^{1}H NMR** (300 MHz, $CDCl_3$) *δ* (ppm): 8.18–8.08 (m, 2H), 7.16–6.99 (m, 2H), 6.63–6.57 (m, 1H), 6.22–6.15 (m, 1H), 6.08 (at, *J* = 3.1 Hz, 1H), 5.98 (s, 1H), 3.66 (s, 3H), 0.95 (s, 9H), 0.10 (s, 3H), 0.04 (s, 3H). **^{13}C NMR** (75.4 MHz, $CDCl_3$) *δ* (ppm): 195.4 (C), 165.6 (d, $^1J_{C–F}$ = 254.9 Hz, C), 132.1 (d, $^3J_{C–F}$ = 9.1 Hz, 2 × CH) 131.5 (d, $^4J_{C–F}$ = 3.0 Hz, C). 129.0 (C), 124.3 (CH), 115.4 (d, $^2J_{C–F}$ = 21.80 Hz, 2 × CH), 110.2 (CH), 107.3 (CH), 73.3 (CH), 34.7 (CH_3), 25.8 (3 × CH_3), 18.3 (C), –4.9 (CH_3), –5.0 (CH_3). **HRMS** (ESI-TOF): calculated for $C_{19}H_{27}FNO_2Si^+$ $[M+H]^+$ 348.1790; found 348.1794.

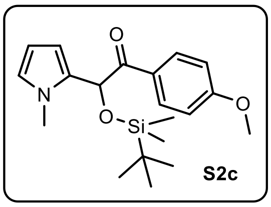

S2c

2-((*Tert*-butyldimethylsilyl)oxy)-1-(4-methoxyphenyl)-2-(1-methyl-1*H*-pyrrol-2-yl)ethan-1-one (S2c): Following the general procedure B with acyloin **S1c** (2.45 g, 10 mmol) and *tert*-butyldimethylchlorosilane (1.96 g, 13 mmol), the crude product was purified by column chromatography (hexane/EtOAc, 10/1), affording pure **S2c** as a yellow oil (3.52 g, 98%); R_f = 0.32 (hexane/EtOAc, 10/1). **^{1}H NMR** (300 MHz, $CDCl_3$) *δ* (ppm): 8.05 (d, *J* = 8.8 Hz, 2H), 6.87 (d, *J* = 8.8 Hz, 2H), 6.58–6.52 (m, 1H), 6.13 (dd, *J* = 3.2, 1.5 Hz, 1H), 6.02 (at, *J* = 3.2 Hz, 1H), 5.94 (s, 1H), 3.83 (s, 3H), 3.63 (s, 3H), 0.90 (s, 9H), 0.05 (s, 3H), –0.01 (s, 3H). **^{13}C NMR** (75.4 MHz, $CDCl_3$) *δ* (ppm): 195.4 (C), 163.4 (C), 131.8 (2 × CH), 129.7 (C), 128.1 (C), 124.1 (CH), 113.6 (2 × CH), 110.0 (CH), 107.2 (CH), 73.1 (CH), 55.5 (CH_3), 34.8 (CH_3), 25.9 (3 × CH_3), 18.4 (C), –4.8 (CH_3), –5.0 (CH_3). **HRMS** (ESI-TOF): calculated for $C_{20}H_{29}NNaO_3Si^+$ $[M+Na]^+$ 382.1817; found 382.1809.

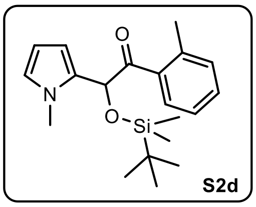

S2d

2-((*Tert*-butyldimethylsilyl)oxy)-2-(1-methyl-1H-pyrrol-2-yl)-1-(o-tolyl)ethan-1-one (S2d): Following the general procedure B with acyloin **S1d** (2.29 g, 10 mmol) and *tert*-butyldimethylchlorosilane (1.96 g, 13 mmol), the crude product was purified by column chromatography (hexane/EtOAc, 20/1), affording pure **S2d** as a yellow oil (2.75 g, 80%); R_f = 0.29 (hexane/EtOAc, 20/1). **^{1}H NMR** (300 MHz, $CDCl_3$) *δ* (ppm): 7.51–7.48 (m, 1H), 7.38–7.25 (m, 1H), 7.23–7.18 (m, 2H), 6.60 (at, *J* = 2.3 Hz, 1H), 6.07–6.02 (m, 2H), 5.89 (d, *J* = 1.7 Hz, 1H), 3.68 (s, 3H), 2.35 (s, 3H), 0.94 (s, 9H), 0.14 (s, 3H), 0.03 (s, 3H).**^{13}C NMR** (75.4 MHz, $CDCl_3$) *δ* (ppm): 201.4 (C), 137.7 (C), 137.4 (C), 131.4 (CH), 130.6 (CH), 128.1 (C), 127.6 (CH), 125.1 (CH), 124.2 (CH), 110.6 (CH), 107.4 (CH), 74.2 (CH), 34.9 (CH_3), 25.8 (3 × CH_3), 20.2 (CH_3), 18.3 (C), –4.99, –5.02. **HRMS** (ESI-TOF): calculated for $C_{20}H_{30}NO_2Si^+$ $[M+H]^+$ 344.2040; found 344.2047.

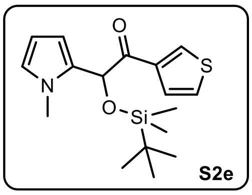

S2e

2-((*Tert*-butyldimethylsilyl)oxy)-2-(1-methyl-1*H*-pyrrol-2-yl)-1-(thiophen-3-yl)ethan-1-one (S2e): Following the general procedure B with acyloin **S1e** (2.21 g, 10 mmol) and *tert*-butyldimethylchlorosilane (1.96 g, 13 mmol), the crude product was purified by column chromatography (hexane/EtOAc, 20/1),

affording pure **S2e** as a yellow oil (2.62 g, 78%); R_f = 0.27 (hexane/EtOAc, 20/1). **^{1}H NMR** (300 MHz, $CDCl_3$) δ (ppm): 8.17 (dd, J = 3.0, 1.2 Hz, 1H), 7.59 (dd, J = 5.1, 1.2 Hz, 1H), 7.22 (dd, J = 5.1, 3.0 Hz, 1H), 6.56 (at, J = 2.3 Hz, 1H), 6.20 (dd, J = 3.7, 1.8 Hz, 1H), 6.06 (dd, J = 3.7, 2.7 Hz, 1H), 5.73 (s, 1H), 3.60 (s, 3H), 0.92 (s, 9H), 0.07 (s, 3H), 0.01 (s, 3H). **^{13}C NMR** (75.4 MHz, $CDCl_3$) δ (ppm): 191.5 (C), 139.0 (C), 133.8 (CH), 129.5 (C), 128.2 (CH), 125.4 (CH), 124.2 (CH), 110.1 (CH), 107.3 (CH), 74.3 (CH), 34.7 (CH_3), 25.9 (3 × CH_3), 18.4 (C), –4.9 (CH_3), –5.0 (CH_3). **HRMS** (ESI-TOF): calculated for $C_{17}H_{26}NO_2SSi^+$ $[M+H]^+$ 336.1448; found 336.1450.

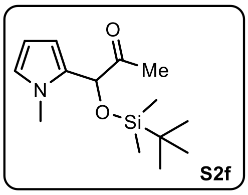

1-((*Tert*-butyldimethylsilyl)oxy)-1-(1-methyl-1*H*-pyrrol-2-yl)propan-2-one (S2f): Following the general procedure B with acyloin **S1f** (1.53 g, 10 mmol) and *tert*-butyldimethylchlorosilane (1.96 g, 13 mmol), **S2f** was obtained pure without further purification; red oil (2.06 g, 77%); R_f = 0.38 (hexane/EtOAc, 10/1). **^{1}H NMR** (300 MHz, $CDCl_3$) δ (ppm): 6.57 (at, J = 2.3 Hz, 1H), 6.17 (dd, J = 3.5, 1.7 Hz, 1H), 6.10–6.05 (m, 1H), 5.13 (s, 1H), 3.55 (s, 3H), 2.17 (s, 3H), 0.94 (s, 9H), 0.10 (s, 3H), –0.06 (s, 3H).**^{13}C NMR** (75.4 MHz, $CDCl_3$) δ (ppm): 206.9 (C), 129.0 (C), 123.9 (CH), 109.6 (CH), 107.2 (CH), 75.5 (CH), 34.5 (CH_3), 25.7 (3 × CH_3), 25.2 (C), 18.2 (CH_3), –5.2 (CH_3), –5.3 (CH_3). **HRMS** (ESI-TOF): calculated for $C_{14}H_{26}NO_2Si^+$ $[M+H]^+$ 268.1727; found 268.1727.

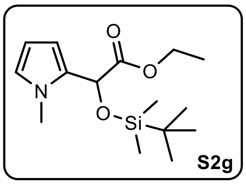

2-((*Tert*-butyldimethylsilyl)oxy)-1-phenyl-2-(1-phenyl-1*H*-pyrrol-2-yl)ethan-1-one (S2g): Following the general procedure B with acyloin **S1g** (1.83 g, 10 mmol) and *tert*-butyldimethylchlorosilane (1.96 g, 13 mmol), **S2g** was obtained slightly contaminated with undefined silicon impurities. The crude product was used in the next step without further purification; red oil (2.67 g, 90%); R_f = 0.38 (hexane/EtOAc, 10/1). **^{1}H NMR** (500 MHz, $CDCl_3$) δ (ppm): 6.56 (at, J = 2.2 Hz, 1H), 6.09 (dd, J = 3.6, 1.9 Hz, 1H), 6.04–6.01 (m, 1H), 4.28–4.09 (m, 2H), 3.66 (s, 3H), 1.25 (t, J = 7.1 Hz, 3H), 0.90 (s, 9H), 0.07 (s, 3H), -0.03 (s, 3H). **^{13}C NMR** (125.7 MHz, $CDCl_3$) δ (ppm): 171.5 (C), 129.2 (C), 124.0 (CH), 109.6 (CH), 106.9 (CH), 69.1 (CH), 61.3 (CH_2), 34.7 (CH_3), 25.8 (3 × CH_3), 18.3 (C), 14.3 (CH_3), –5.12 (CH_3), –5.13 (CH_3). **HRMS** (ESI-TOF): calculated for $C_{15}H_{27}NNaO_3Si^+$ $[M+Na]^+$ 320.1652; found 320.1661.

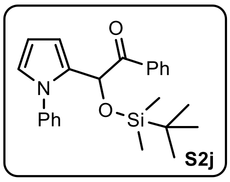

2-((*Tert*-butyldimethylsilyl)oxy)-1-phenyl-2-(1-phenyl-1*H*-pyrrol-2-yl)ethan-1-one (S2j): Following the general procedure B with acyloin **S1j** (2.77 g, 10 mmol) and *tert*-butyldimethylchlorosilane (1.96 g, 13 mmol), the crude product was purified by column chromatography (hexane/EtOAc, 15/1), affording pure **S2j** as a yellow oil (2.27 g, 58%); R_f = 0.47 (hexane/EtOAc, 10/1). **^{1}H NMR** (300 MHz, $CDCl_3$) δ (ppm): 7.82–7.72 (m, 2H), 7.56–7.30 (m, 8H), 6.90–6.78 (m, 1H), 6.31–6.27 (m, 1H), 6.25–6.20 (m, 1H), 5.92–5.88 (m, 1H), 0.89 (s, 9H), –0.05 (s, 6H). **^{13}C NMR** (75.4 MHz, $CDCl_3$) δ (ppm): 196.8 (C), 139.5 (C), 135.4 (C), 132.7 (CH), 130.3 (C), 129.3 (2 × CH), 129.0 (2 × CH), 128.3 (2 × CH), 127.9 (CH), 126.6 (2 × CH), 123.9 (CH), 111.8 (CH), 109.0 (CH), 71.8 (CH), 25.9 (3 × CH_3), 18.4 (C), –4.4 (CH_3), –4.6 (CH_3). **HRMS** (ESI-TOF): calculated for $C_{24}H_{29}NNaO_2Si^+$ $[M+Na]^+$ 414.1860; found 414.1868.

General procedure C for the synthesis of alkynols 1

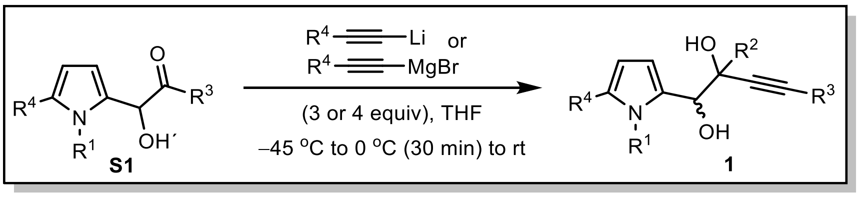

For the synthesis of alkynols **1aa**–**1aj**, *n*-BuLi (9 mmol, 3.6 mL of a 2.5 M solution in hexane, 3 equiv) was added to a solution of the appropriate alkyne (9.6 mmol, 3.2 equiv) in THF (15 mL, 0.64 M referred to the alkyne) at –45 °C. For the synthesis of alkynols **1ha**-**1hh**, *n*-BuLi (12 mmol, 4.8 mL of a 2.5 M solution in hexane, 4 equiv) was added to a solution of the appropriate alkyne (12.6 mmol, 4.2 equiv) in THF (20 mL, 0.63 M referred to alkyne) at –45 °C. For the synthesis of alkynols **1ia** and **1ib**, EtMgBr (12 mmol, 4 mL of a 3 M solution in diethylether, 4 equiv) was added to a solution of the appropriate alkyne (12.6 mmol, 4.2 equiv.) in THF (20 mL, 0.63 M referred to the alkyne) at –45 °C. In all cases, the resulting solution was stirred for 30 min at 0 °C to obtain the corresponding lithium or magnesium acetylide. The corresponding acyloin **S1** (3 mmol, 1 equiv) in THF (5 mL) was added dropwise to the acetylide solution at –45 °C. After 5 min at this temperature, the resulting mixture was stirred at 0 °C for 30 min. Then, the mixture was stirred at rt until the starting acyloin was consumed as determined by TLC. Aqueous NH_4Cl (20 mL) was added and THF was removed under reduced pressure. The residue was extracted with Et_2O (3 × 15 mL) The combined organic layers were dried over anhydrous Na_2SO_4 and the solvents were removed under reduced pressure. The residue was purified by flash column chromatography using mixtures of hexane and EtOAc as eluents to obtain the corresponding alkynols **1**.

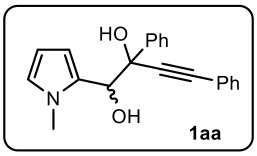

1-(1-Methyl-1*H*-pyrrol-2-yl)-2,4-diphenylbut-3-yne-1,2-diol (1aa): Following the general procedure C with 2-hydroxy-2-(1-methyl-1*H*-pyrrol-2-yl)-1-phenylethan-1-one (**S1a**) (646 mg, 3 mmol) and phenylacetylene (1.05 mL, 9.6 mmol), the crude product was purified by column chromatography (hexane/EtOAc, 3/1), affording pure **1aa** as a brown solid (628 mg, 66%). Obtained and isolated as only one diastereoisomer whose relative configuration was not established. mp = 139–141 °C; R_f = 0.21 (hexane/EtOAc, 3/1). **^{1}H NMR** (300 MHz, $CDCl_3$) δ (ppm): 7.69–7.61 (m, 2H), 7.60–7.52 (m, 2H), 7.42–7.32 (m, 6H), 6.56–6.50 (m, 1H), 6.42 (dd, *J* = 3.6, 1.6 Hz, 1H), 6.17–6.09 (m, 1H), 4.94 (d, *J* = 4.5 Hz, 1H), 3.36–3.30 (s, 4H), 2.64 (d, *J* = 4.5 Hz, 1H). **^{13}C NMR** (75.4 MHz, $CDCl_3$) δ (ppm): 141.0 (C), 131.9 (2 × CH), 129.4 (C), 128.8 (CH), 128.4 (2 × CH), 128.3 (CH), 128.0 (2 × CH), 126.70 (2 × CH), 123.2 (CH), 122.3 (C), 108.6 (CH), 107.2 (CH), 89.8 (C), 87.4 (C), 76.6 (C), 74.6 (CH), 34.1 (CH_3). **HRMS** (ESI-TOF): calculated for $C_{21}H_{20}NO_2^+$ $[M+H]^+$ 318.1489; found 318.1488.

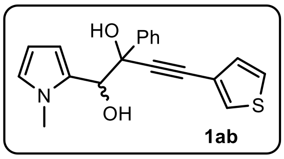

1-(1-Methyl-1*H*-pyrrol-2-yl)-2-phenyl-4-(thiophen-3-yl)but-3-yne-1,2-diol (1ab): Following the general procedure C with 2-hydroxy-2-(1-methyl-1*H*-pyrrol-2-yl)-1-phenylethan-1-one (**S1a**) (646 mg, 3 mmol) and 3-ethynylthiophene (0.95 mL, 9.6 mmol), the crude product was purified by

column chromatography (hexane/EtOAc, 2/1), affording pure **1ab** as a pale yellow solid (631 mg, 65%). Obtained and isolated as only one diastereoisomer whose relative configuration was not established; mp = 138–140 °C; R_f = 0.33 (hexane/EtOAc, 2/1). **^{1}H NMR** (300 MHz, $CDCl_3$) δ (ppm): 7.66–7.56 (m, 2H), 7.55 (dd, J = 3.0, 1.2 Hz, 1H), 7.39–7.29 (m, 4H), 7.20 (dd, J = 5.0, 1.2 Hz, 1H), 6.54–6.47 (m, 1H), 6.40 (dd, J = 3.6, 1.6 Hz, 1H), 6.16–6.08 (m, 1H), 4.92 (d, J = 4.5 Hz, 1H), 3.26 (s, 3H), 2.62 (d, J = 4.5 Hz, 1H). **^{13}C NMR** (75.4 MHz, $CDCl_3$) δ (ppm): 140.9 (C), 130.0 (CH), 129.7 (CH), 129.4 (C), 128.3 (CH), 128.0 (2 × CH), 126.7 (2 × CH), 125.6 (CH), 123.2 (CH), 121.3 (C), 108.6 (CH), 107.2 (CH), 89.5 (C), 82.6 (C), 76.6 (C), 74.5 (CH), 34.1 (CH_3). **HRMS** (ESI-TOF): calculated for $C_{19}H_{18}NO_2S^+$ $[M+H]^+$ 324.1053; found 324.1052.

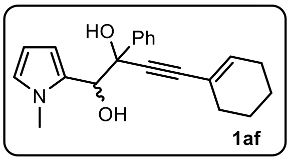

4-(Cyclohex-1-en-1-yl)-1-(1-methyl-1*H*-pyrrol-2-yl)-2-phenylbut-3-yne-1,2-diol (1af): Following the general procedure C with 2-hydroxy-2-(1-methyl-1*H*-pyrrol-2-yl)-1-phenylethan-1-one (**S1a**) (646 mg, 3 mmol) and ethynylcyclohexene (1.13 mL, 9.6 mmol), the crude product was purified by column chromatography (hexane/EtOAc, 3/1), affording pure **1af** as a yellow solid (521 mg, 54%); mp = 133–135 °C; R_f = 0.16 (hexane/EtOAc, 3/1). **^{1}H NMR** (300 MHz, $CDCl_3$) δ (ppm): 7.61–7.52 (m, 2H), 7.37–7.28 (m, 3H), 6.50 (at, J = 2.2 Hz, 1H), 6.36 (dd, J = 3.8, 1.8 Hz, 1H), 6.25 (tt, J = 3.8, 1.8 Hz, 1H), 6.10 (dd, J = 3.8, 2.7 Hz, 1H), 4.90–4.79 (m, 1H), 3.25 (s, 3H), 3.02 (bs, 1H), 2.51 (bs, 1H), 2.27–2.08 (m, 4H), 1.81–1.56 (m, 4H). **^{13}C NMR** (75.4 MHz, $CDCl_3$) δ (ppm): 141.3 (C), 136.2 (CH), 129.5 (C), 128.1 (CH), 128.0 (2 × CH), 126.7 (2 × CH), 123.1 (CH), 120.0 (C), 108.5 (CH), 107.1 (CH), 89.5 (C), 86.8 (C), 76.5 (C), 74.6 (CH), 34.1 (CH_3), 29.1 (CH_2), 25.8 (CH_2), 22.3 (CH_2), 21.5 (CH_2). **HRMS** (ESI-TOF): calculated for $C_{21}H_{24}NO_2^+$ $[M+H]^+$ 322.1802; found 322.1804.

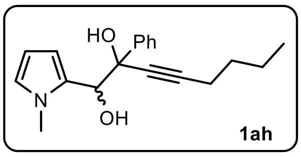

1-(1-Methyl-1*H*-pyrrol-2-yl)-2-phenyloct-3-yne-1,2-diol (1ah): Following the general procedure C with 2-hydroxy-2-(1-methyl-1*H*-pyrrol-2-yl)-1-phenylethan-1-one (**S1a**) (646 mg, 3 mmol) and 1-hexyne (1.1 mL, 9.6 mmol), the crude product was purified by column chromatography (hexane/EtOAc, 3/1), affording pure **1ah** as a yellow oil (624 mg, 70%). Obtained and isolated as only one diastereoisomer whose relative configuration was not established; R_f = 0.28 (hexane/EtOAc, 3/1). **^{1}H NMR** (300 MHz, $CDCl_3$) δ (ppm): 7.61–7.49 (m, 2H), 7.37–7.29 (m, 3H), 6.52–6.45 (m, 1H), 6.34 (dd, J = 3.5, 1.5 Hz, 1H), 6.14–6.05 (m, 1H), 4.81 (s, 1H), 3.21 (s, 3H), 2.98 (bs, 1H), 2.59 (bs, 1H), 2.37 (t, J = 7.0 Hz, 2H), 1.61 (quin, J = 7.0 Hz, 2H), 1.47 (hex, J = 7.2 Hz, 2H), 0.96 (t, J = 7.2 Hz, 3H). **^{13}C NMR** (75.4 MHz, $CDCl_3$) δ (ppm): 141.5 (C), 129.4 (C), 128.1 (CH), 127.9 (2 × CH), 126.7 (2 × CH), 123.1 (CH), 108.4 (CH), 107.1 (CH), 88.6 (C), 80.8 (C), 76.2 (C), 74.5 (CH), 34.0 (CH_3), 30.7 (CH_2), 22.2 (CH_2), 18.7 (CH_2), 13.7 (CH_3). **HRMS** (ESI-TOF): calculated for $C_{19}H_{24}NO_2^+$ $[M+H]^+$ 298.1802; found 298.1798.

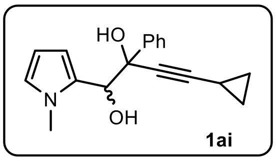

4-Cyclopropyl-1-(1-methyl-1*H*-pyrrol-2-yl)-2-phenylbut-3-yne-1,2-diol (1ai): Following the general procedure C with 2-hydroxy-2-(1-methyl-1*H*-pyrrol-2-yl)-1-phenylethan-1-one (**S1a**) (646 mg, 3 mmol), ethynylcyclopropane (0.88 mL, 9.6 mmol), the crude product was purified by

column chromatography (hexane/EtOAc, 3/1), affording pure **1ai** as an orange solid (566 mg, 67%). Obtained and isolated as only one diastereoisomer whose relative configuration was not established; mp = 128–130 °C; R_f = 0.27 (hexane/EtOAc, 3/1). **^{1}H NMR** (300 MHz, $CDCl_3$) δ (ppm): 7.57–7.47 (m, 2H), 7.35–7.29 (m, 3H), 6.54–6.45 (m, 1H), 6.32 (dd, J = 3.5, 1.7 Hz, 1H), 6.14–6.03 (m, 1H), 4.79 (s, 1H), 3.23 (s, 3H), 2.90 (bs, 1H), 2.45 (bs, 1H), 1.48–1.33 (m, 1H), 0.92–0.74 (m, 4H). **^{13}C NMR** (75.4 MHz, $CDCl_3$) δ (ppm): 141.5 (C), 129.5 (C), 128.1 (CH), 128.0 (2 × CH), 126.6 (2 × CH), 123.1 (CH), 108.4 (CH), 107.1 (CH), 91.60 (C), 76.2 (C), 75.9 (C), 74.6 (CH), 34.1 (CH_3), 8.5 (CH_2), 8.4 (CH_2), –0.3 (CH). **HRMS** (ESI-TOF): calculated for $C_{18}H_{20}NO_2^+$ $[M+H]^+$ 282.1489; found 282.1485.

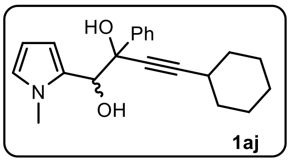

4-Cyclohexyl-1-(1-methyl-1*H*-pyrrol-2-yl)-2-phenylbut-3-yne-1,2-diol (1aj): Following the general procedure C with 2-hydroxy-2-(1-methyl-1*H*-pyrrol-2-yl)-1-phenylethan-1-one (**S1a**) (646 mg, 3 mmol) and cyclohexylacetylene (1.25 mL, 9.6 mmol), the crude product was purified by column chromatography (hexane/EtOAc, 3/1), affording pure **1aj** as an orange solid (621 mg, 64%). Obtained and isolated as 20/1 mixture of diastereoisomers; mp = 136–138 °C; R_f = 0.27 (hexane/EtOAc, 3/1). Data for major diastereoisomer: **^{1}H NMR** (300 MHz, $CDCl_3$) δ (ppm): 7.67–7.51 (m, 2H), 7.44–7.28 (m, 3H), 6.51 (at, J = 2.2 Hz, 1H), 6.37 (dd, J = 3.7, 1.8 Hz, 1H), 6.13 (dd, J = 3.7, 2.7 Hz, 1H), 4.83 (s, 1H), 3.24 (s, 3H), 2.59 (tt, J = 9.3, 3.8 Hz, 1H), 2.04–1.20 (m, 12H). **^{13}C NMR** (75.4 MHz, $CDCl_3$) δ (ppm): 141.5 (C), 129.4 (C), 128.0 (CH), 127.8 (2 × CH), 126.7 (2 × CH), 122.9 (CH), 108.4 (CH), 107.0 (CH), 92.5 (C), 80.6 (C), 76.1 (CH), 74.5 (C), 34.0 (CH_3), 32.6 (CH_2), 32.5 (CH_2), 29.2 (CH), 25.8 (CH_2), 24.9 (2 × CH_2). **HRMS** (ESI-TOF): calculated for $C_{21}H_{26}NO_2^+$ $[M+H]^+$ 324.1958; found 324.1966.

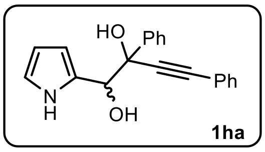

2,4-Diphenyl-1-(1*H*-pyrrol-2-yl)but-3-yne-1,2-diol (1ha): Following the general procedure C with 2-hydroxy-1-phenyl-2-(1*H*-pyrrol-2-yl)ethan-1-one (**S1h**) (604 mg, 3 mmol) and phenylacetylene (1.05 mL, 9.6 mmol), the crude product was purified by column chromatography (hexane/EtOAc, 2/1), affording pure **1ha** as a dark green foam (373 mg, 41%). Obtained and isolated as a 3.6/1 mixture of diastereoisomers; mp = 144–146 °C; R_f = 0.35 (hexane/EtOAc, 2/1). Data for major diastereoisomer: **^{1}H NMR** (300 MHz, $CDCl_3$) δ (ppm): 8.61 (bs, 1H), 7.69–7.30 (m, 10H), 6.74 (aq, J = 2.2 Hz, 1H), 6.17–6.11 (m, 2H), 5.00 (s, 1H), 3.32–2.51 (m, 2H). **^{13}C NMR** (75.4 MHz, $CDCl_3$) δ (ppm): 140.4 (C), 132.0 (2 × CH), 129.0 (CH), 128.5 (2 × CH), 128.4 (CH), 128.2 (CH), 128.1 (C), 126.5 (2 × CH), 122.2 (C), 118.2 (CH), 108.5 (CH), 108.1 (CH), 89.7 (C), 87.4 (C), 76.5 (C), 76.2 (CH). **HRMS** (ESI-TOF): calculated for $C_{21}H_{17}NO_2^+$ $[M+H]^+$ 304.1332; found 304.1334.

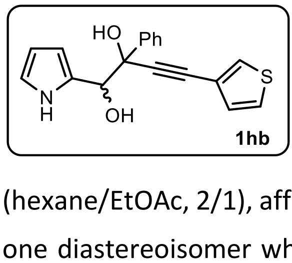

2-Phenyl-1-(1*H*-pyrrol-2-yl)-4-(thiophen-3-yl)but-3-yne-1,2-diol (1hb): Following the general procedure C with 2-hydroxy-1-phenyl-2-(1*H*-pyrrol-2-yl)ethan-1-one (**S1h**) (604 mg, 3 mmol) and 3-ethynylthiophene (0.95 mL, 9.6 mmol), the crude product was purified by column chromatography (hexane/EtOAc, 2/1), affording pure **1hb** as a green solid (613 mg, 66%). Obtained and isolated as only one diastereoisomer whose relative configuration was not established; mp = 129–131 °C; R_f = 0.29

(hexane/EtOAc, 2/1). **^{1}H NMR** (300 MHz, acetone-d$_6$) δ (ppm): 9.56 (bs, 1H), 7.72–7.41 (m, 4H), 7.34–7.06 (m, 4H), 6.65 (aq, *J* = 2.3 Hz, 1H), 5.96–5.89 (m, 2H), 5.01 (s, 1H), 4.97 (d, *J* = 4.7 Hz, 1H), 4.60 (d, *J* = 4.7 Hz, 1H). **^{13}C NMR** (75.4 MHz, acetone-d$_6$) δ (ppm): 143.1 (C), 130.7 (CH), 129.8 (CH), 128.00 (2 × CH), 127.97 (CH), 127.5 (2 × CH), 126.7 (CH), 122.9 (C), 117.7 (CH), 107.9 (CH), 107.7 (CH), 92.1 (C), 81.5 (C), 77.0 (C), 76.8 (CH). One aromatic peak was missing due to overlapping. **HRMS** (ESI-TOF): calculated for $C_{18}H_{15}NNaO_2^+$ [M+Na]$^+$ 333.0747; found 333.0741.

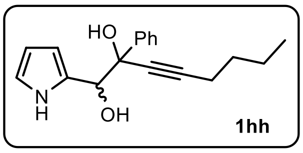

1hh

2-Phenyl-1-(1*H*-pyrrol-2-yl)oct-3-yne-1,2-diol (1hh): Following the general procedure C with 2-hydroxy-1-phenyl-2-(1*H*-pyrrol-2-yl)ethan-1-one (**S1h**) (604 mg, 3 mmol) and 1-hexyne (1.1 mL, 9.6 mmol), the crude product was purified by column chromatography (hexane/EtOAc, 2/1), affording pure **1hh** as a yellow oil (527 mg, 62%). Obtained and isolated as a 15/1 mixture of diastereoisomers; R$_f$ = 0.32 (hexane/EtOAc, 2/1). Data for the major diastereoisomer: **^{1}H NMR** (300 MHz, CDCl$_3$) δ (ppm): 8.66 (bs, 1H), 7.55–7.47 (m, 2H), 7.36–7.26 (m, 3H), 6.69–6.61 (m, 1H), 6.09 (aq, *J* = 2.9 Hz, 1H), 6.05–5.94 (m, 1H), 4.84 (s, 1H), 3.52–3.02 (m, 2H), 2.34 (t, *J* = 7.1 Hz, 2H), 1.66–1.36 (m, 4H), 0.99 (t, *J* = 6.3 Hz, 3H). **^{13}C NMR** (75.4 MHz, CDCl$_3$) δ (ppm): 140.8 (C), 128.0 (C), 127.8 (CH), 127.7 (2 × CH), 126.3 (2 × CH), 117.9 (CH), 108.3 (CH), 107.6 (CH), 88.2 (C), 76.0 (C) 75.9 (CH), 30.6 (CH$_2$), 22.0 (CH$_2$), 18.5 (CH$_2$), 13.6 (CH$_3$). **HRMS** (ESI-TOF): calculated for $C_{18}H_{21}NNaO_2^+$ [M+Na]$^+$ 306.1465; found 306.1474.

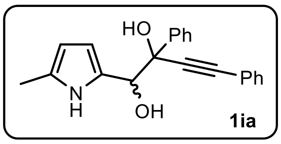

1ia

1-(5-Methyl-1*H*-pyrrol-2-yl)-2,4-diphenylbut-3-yne-1,2-diol (1ia): Following the general procedure C with 2-hydroxy-2-(5-methyl-1*H*-pyrrol-2-yl)-1-phenylethan-1-one (**S1i**) (646 mg, 3 mmol) and phenylacetylene (1.05 mL, 9.6 mmol), the crude product was purified by column chromatography (hexane/EtOAc, 3/1), affording pure **1ia** as a beige solid (352 mg, 37%). Obtained and isolated as only one diastereoisomer whose relative configuration was not determined; mp = 153–155 °C; R$_f$ = 0.40 (hexane/EtOAc, 2/1). **^{1}H NMR** (300 MHz, CDCl$_3$) δ (ppm): 8.29 (bs, 1H), 7.70–7.62 (m, 2H), 7.54–7.46 (m, 2H), 7.42–7.29 (m, 6H), 6.01 (at, *J* = 2.9 Hz, 1H), 5.78 (at, *J* = 2.9 Hz, 1H), 4.88 (s, 1H), 3.13 (bs, 1H), 2.59 (bs, 1H), 2.20 (s, 3H). **^{13}C NMR** (75.4 MHz, CDCl$_3$) δ (ppm): 140.6 (C), 131.9 (2 × CH), 129.0 (CH), 128.5 (2 × CH), 128.4 (CH), 128.2 (2 × CH), 126.5 (2 × CH), 126.4 (C), 122.3 (C), 109.1 (CH), 105.9 (CH), 89.9 (C), 87.3 (C), 76.32 (CH), 76.30 (C), 13.2 (CH$_3$). One aromatic peak was missing due to overlapping. **HRMS** (ESI-TOF): calculated for $C_{21}H_{19}NNaO_2^+$ [M+Na]$^+$ 340.1308; found 340.1316.

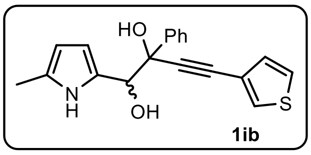

1ib

1-(5-Methyl-1*H*-pyrrol-2-yl)-2-phenyl-4-(thiophen-3-yl)but-3-yne-1,2-diol (1ib): Following the general procedure C with 2-hydroxy-2-(5-methyl-1*H*-pyrrol-2-yl)-1-phenylethan-1-one (**S1i**) (646 mg, 3 mmol) and 3-ethynylthiophene (0.95 mL, 9.6 mmol), the crude product was purified by column chromatography (hexane/EtOAc, 2/1), affording pure **1ib** as an orange oil (388 mg, 40%). Obtained and isolated as only one diastereoisomer whose relative configuration was not determined; R$_f$ = 0.36 (hexane/EtOAc, 2/1). **^{1}H NMR** (300 MHz, CDCl$_3$) δ (ppm): 8.36 (bs, 1H), 7.70–7.60 (m, 2H),

7.52 (dd, J = 3.0, 1.2 Hz, 1H), 7.44–7.25 (m, 4H), 7.17 (dd, J = 5.0, 1.2 Hz, 1H), 6.00 (at, J = 3.0 Hz, 1H), 5.79 (at, J = 3.0 Hz, 1H), 4.90 (s, 1H), 3.31 (bs, 1H), 2.87 (bs, 1H), 2.21 (s, 3H). **^{13}C NMR** (75.4 MHz, $CDCl_3$) δ (ppm): 140.6 (C), 129.9 (CH), 129.7 (CH), 128.3 (CH), 128.3 (CH), 128.2 (2 × CH), 126.5 (2 × CH), 126.4 (C), 125.7 (CH), 121.3 (C), 109.0 (CH), 105.8 (CH), 89.6 (C), 82.4 (C), 76.3 (C), 76.2 (CH), 13.1 (CH_3). **HRMS** (ESI-TOF): calculated for $C_{19}H_{18}NO_2S^+$ [M+H]$^+$ 324.1053; found 324.1060.

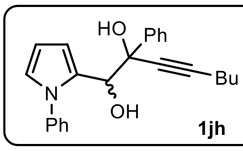

1jh

2-Phenyl-1-(1-phenyl-1*H*-pyrrol-2-yl)oct-3-yne-1,2-diol (1jh): Following the general procedure C with 2-hydroxy-1-phenyl-2-(1-phenyl-1*H*-pyrrol-2-yl)ethan-1-one (**S1j**) (832 mg, 3 mmol) and 1-hexyne (1.1 mL, 9.6 mmol), the crude product was purified by column chromatography (hexane/EtOAc, 3/1), affording pure **1jh** as a yellow oil (485 mg, 45%). Obtained and isolated as only one diastereoisomer whose relative configuration was not determined; R_f = 0.40 (hexane/EtOAc, 3/1). **^{1}H NMR** (300 MHz, $CDCl_3$) δ (ppm): 7.59–7.18 (m, 8H), 7.15–7.04 (m, 2H), 6.78–6.70 (m, 2H), 6.31 (at, J = 3.2 Hz, 1H), 4.71 (s, 1H), 3.00 (bs, 1H), 2.57 (bs, 1H), 2.37 (t, J = 7.1 Hz, 2H), 1.73–1.35 (m, 4H), 0.97 (t, J = 7.3 Hz, 3H). **^{13}C NMR** (75.4 MHz, $CDCl_3$) δ (ppm): 141.6 (C), 139.5 (C), 130.5 (C), 129.0 (2 × CH), 127.9 (2 × CH), 127.8 (CH), 127.5 (CH), 126.8 (2 × CH), 126.5 (2 × CH), 123.3 (CH), 109.1 (CH), 108.7 (CH), 88.4 (C), 81.1 (C), 76.1 (C), 73.3 (CH), 30.7 (CH_2), 22.2 (CH_2), 18.7 (CH_2), 13.7 (CH_3). **HRMS** (ESI-TOF): calculated for $C_{24}H_{26}NO_2^+$ [M+H]$^+$ 360.1958; found 360.1965.

General procedures D for the synthesis of alkynols 2

*General procedure D.1 for the synthesis of alkynols **2***

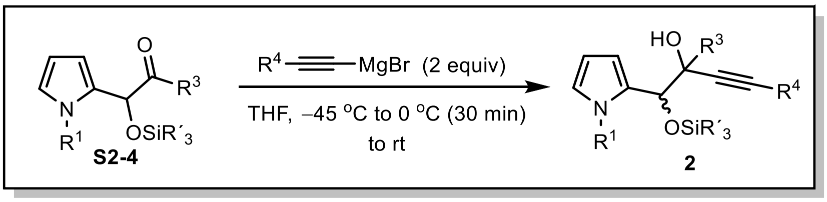

EtMgBr (4 mmol, 1.33 mL of a 3 M solution in Et_2O, 2 equiv) was added to a solution of the appropriate alkyne (4.4 mmol, 2.2 equiv) in THF (10 mL, 0.44 M referred to the alkyne) at –45 °C. The resulting solution was stirred for 20 min at 0 °C to obtain the corresponding magnesium acetylide. The corresponding ketone **S2-4** (2 mmol, 1 equiv) in THF (5 mL) was added dropwise to the acetylide solution at –45 °C. After 5 min at this temperature, the resulting mixture was stirred at 0 °C for 30 min. Then, the mixture was stirred at rt until the electrophile was consumed as determined by TLC. Aqueous NH_4Cl (20 mL) was added and most of THF was removed under reduced pressure. The residue was extracted with Et_2O (3 × 15 mL). The combined organic layers were dried over anhydrous Na_2SO_4 and the solvents were removed under reduced pressure. The residue was purified by flash column chromatography using mixtures of hexane and EtOAc as eluents to obtain the corresponding alkynols **2aa**-**ag**,**ak**,**ba**,**bf**,**ca**,**ea**,**fa**,**fb**,**ff**,**ja**. **2da** was employed in the next step without further purification.

*General procedure D.2 for the synthesis of alkynols **2***

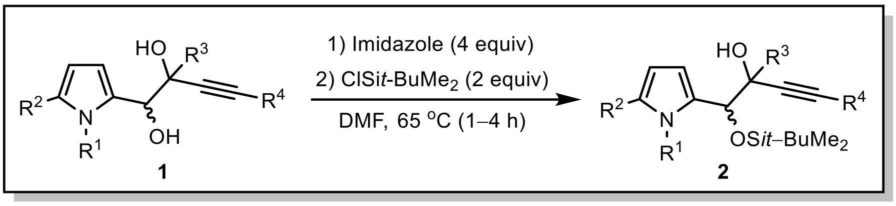

A mixture of the corresponding alkynol **1** (1 mmol, 1 equiv), imidazole (272 mg, 4 mmol, 4 equiv) and *tert*-butyldimethylchlorosilane (301 mg, 2 mmol, 2 equiv) in anhydrous DMF (10 mL, 0.1 M) was heated at 65 °C until the starting alkynol **1** was consumed as determined by TLC (1–4 h). The reaction mixture was cooled and H_2O (10 mL) was added. The residue was extracted with DCM (3 × 10 mL) and the organic phase was washed with H_2O (3 × 20 mL). The combined organic layers were dried over anhydrous Na_2SO_4 and the solvents were removed under reduced pressure. The residue was purified by flash column chromatography using mixtures of hexane and EtOAc as eluents to obtain the corresponding alkynols **2ah**–**aj**,**ha**–**hh**,**ia**,**ib**,**jh**.

*General procedure D.3 for the synthesis of alkynols **2***

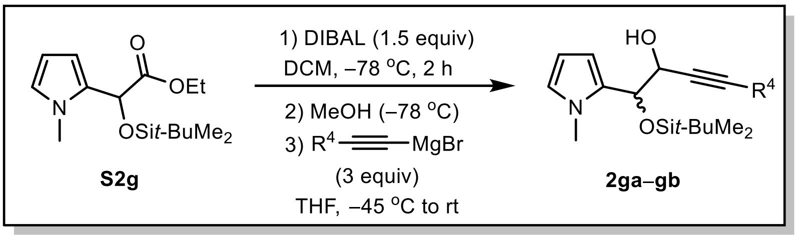

Diisobutylaluminium hydride (3 mmol, 3 mL of a 1 M solution in hexane, 1.5 equiv) was added dropwise at –78 °C to a solution of protected acyloin **S2g** (2 mmol, 595 mg, 1 equiv) in anhydrous DCM (20 mL, 0.1 M) under nitrogen atmosphere. The resulting mixture was stirred at –78 °C for 2 h. Then, MeOH (10 mL) was added at –78 °C. After the MeOH was removed under reduced pressure, the residue was extracted with DCM (3 × 10 mL). The combined organic layers were dried over anhydrous Na_2SO_4 and the solvents were removed under reduced pressure. The corresponding aldehyde derivative (reduction product of ketoester **S2g**) was employed in the next step without further purification.

EtMgBr (6 mmol, 2 mL of a 3 M solution in diethylether, 3 equiv) was added to a solution of the appropriate alkyne (6.4 mmol, 3.2 equiv) in THF (10 mL, 0.64 M referred to the alkyne) at –45 °C. The resulting solution was stirred for 30 min at 0 °C to obtain the corresponding magnesium acetylide. A solution of the crude aldehyde obtained in the previous step (considering 2 mmol, 1 equiv) in THF (5 mL) was added dropwise to the acetylide solution at –45 °C. After 5 min at this temperature, the resulting mixture was stirred at 0 °C for 30 min. Then, the mixture was stirred at rt for 1 h. Aqueous NH_4Cl (20 mL) was added and most of THF was eliminated under reduced pressure. The residue was extracted with Et_2O (3 × 15 mL). The combined organic layers were dried over anhydrous Na_2SO_4 and the solvents were removed under reduced pressure. The residue was purified by flash column chromatography using mixtures of hexane and EtOAc as eluents to obtain the corresponding alkynols **2ga** and **2gb**.

*Characterization data of alkynols **2***

In the ^{1}H NMR and ^{13}C NMR spectra of alkynols **2** some peaks exhibit broad signals attributed to restricted rotation, which are denoted as bs (broad signals). Additionally, in most instances, the CH bonded to silyloxy group (*C*HOSi) does not appear, likely due to this restricted rotation.

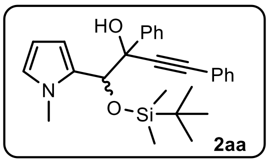

1-((*Tert*-butyldimethylsilyl)oxy)-1-(1-methyl-1*H*-pyrrol-2-yl)-2,4-diphenylbut-3-yn-2-ol (2aa): Following the general procedure D.1 with 2-((*tert*-butyldimethylsilyl)oxy)-2-(1-methyl-1*H*-pyrrol-2-yl)-1-phenylethan-1-one (**S2a**) (659 mg, 2 mmol) and phenylacetylene (0.48 mL, 4.4 mmol), the crude product was purified by column chromatography (hexane/EtOAc, 7/1), affording pure **2aa** as a yellow gel (535 mg, 62%). Obtained and isolated as only one diastereoisomer whose relative configuration was not determined; R_f = 0.42 (hexane/EtOAc, 7/1). **^{1}H NMR** (300 MHz, $CDCl_3$) δ (ppm): 7.66–7.52 (m, 4H), 7.41–7.29 (m, 6H), 6.49–6.44 (m, 1H), 6.29–6.23 (m, 1H), 6.08 (at, J = 2.9 Hz, 1H), 4.87 (s, 1H), 3.41–3.24 (m, 4H), 0.87 (s, 9H), –0.14 (s, 2H), –0.20 (s, 3H). **^{13}C NMR** (75.4 MHz, $CDCl_3$) δ (ppm): 141.1 (C), 131.7 (2 × CH), 129.6 (C), 128.4 (CH), 128.3 (2 × CH), 127.9 (CH), 127.6 (2 × CH), 126.9 (2 × CH), 123.0 (C), 122.6 (CH, bs), 110.2 (CH, bs), 106.9 (CH), 91.4 (C), 86.0 (C), 76.9 (C), 34.3 (CH_3, bs), 25.7 (3 × CH_3), 18.2 (C), –5.1 (CH_3), –5.7 (CH_3). The *C*HOSi peak was missing due to restricted rotation. **HRMS** (ESI-TOF): calculated for $C_{27}H_{34}NO_2Si^+$ $[M+H]^+$ 432.2353; found 432.2349.

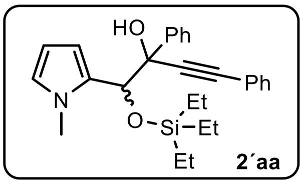

1-(1-Methyl-1*H*-pyrrol-2-yl)-2,4-diphenyl-1-((triethylsilyl)oxy)but-3-yn-2-ol (2´aa): Following the general procedure D.1 with 2-(1-methyl-1*H*-pyrrol-2-yl)-1-phenyl-2-((triethylsilyl)oxy)ethan-1-one (**S3a**) (659 mg, 2 mmol) and phenylacetylene (0.48 mL, 4.4 mmol), the crude product was purified by column chromatography (hexane/EtOAc, 7/1), affording pure **2´aa** as a yellow gel (578 mg, 67%). Obtained and isolated as only one diastereoisomer whose relative configuration was not determined; R_f = 0.32 (hexane/EtOAc, 7/1). **^{1}H NMR** (300 MHz, $CDCl_3$) δ (ppm): 7.64–7.46 (m, 4H), 7.39–7.26 (m, 6H), 6.42 (at, J = 2.1 Hz, 1H), 6.27–6.17 (m, 1H), 6.10–5.98 (m, 1H), 4.89 (s, 1H), 3.32 (s, 1H), 3.27 (s, 3H), 0.82 (t, J = 7.8 Hz, 9H), 0.44 (q, J = 7.8 Hz, 6H). **^{13}C NMR** (75.4 MHz, $CDCl_3$) δ (ppm): 141.2 (C), 131.8 (2 × CH), 129.8 (C), 128.5 (CH), 128.4 (2 × CH), 128.0 (CH), 127.7 (2 × CH), 127.0 (2 × CH), 123.1 (C), 122.7 (CH, bs), 110.2 (CH), 107.0 (CH), 91.6 (C), 85.9 (C), 77.0 (C), 76.4 (CH, bs), 34.5 (CH_3), 6.7 (3 × CH_3), 4.7 (3 × CH_2). **HRMS** (ESI-TOF): calculated for $C_{27}H_{34}NO_2Si^+$ $[M+H]^+$ 432.2353; found 432.2361.

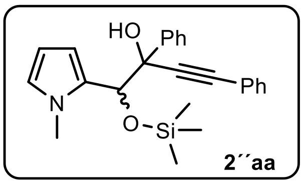

1-(1-Methyl-1*H*-pyrrol-2-yl)-2,4-diphenyl-1-((trimethylsilyl)oxy)but-3-yn-2-ol (2´´aa): Following the general procedure D.1 with 2-(1-methyl-1*H*-pyrrol-2-yl)-1-phenyl-2-((trimethylsilyl)oxy)ethan-1-one (**S4a**) (575 mg, 2 mmol) and phenylacetylene (0.48 mL, 4.4 mmol), the crude product was purified by column chromatography (hexane/EtOAc, 10/1), affording pure **2´´aa** as a yellow oil (413 mg, 53%). Obtained and isolated as only one diastereoisomer whose relative configuration was not determined; R_f = 0.38 (hexane/EtOAc, 7/1). **^{1}H NMR** (300 MHz, $CDCl_3$) δ (ppm):

7.62–7.49 (m, 4H), 7.39–7.27 (m, 6H), 6.44 (at, *J* = 2.1 Hz, 1H), 6.21 (dd, *J* = 3.5, 1.8 Hz, 1H), 6.11–6.03 (m, 1H), 4.84 (s, 1H), 3.35 (s, 1H), 3.28 (s, 3H), –0.05 (s, 9H). **^{13}C NMR** (75.4 MHz, $CDCl_3$) *δ* (ppm): 141.01 (C), 131.9 (2 × CH), 129.5 (C), 128.5 (CH), 128.4 (2 × CH), 128.0 (CH), 127.7 (2 × CH), 127.0 (2 × CH), 123.1 (C), 122.9 (CH), 110.1 (CH), 106.9 (CH), 91.5 (C), 85.9 (C), 76.8 (CH), 76.6 (C), 34.6 (CH_3), –0.24 (3 × CH_3). **HRMS** (ESI-TOF): calculated for $C_{24}H_{28}NO_2Si^+$ $[M+H]^+$ 390.1884; found 390.1894.

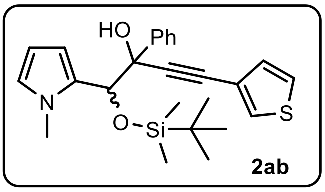

1-((*Tert*-butyldimethylsilyl)oxy)-1-(1-methyl-1*H*-pyrrol-2-yl)-2-phenyl-4-(thiophen-3-yl)but-3-yn-2-ol (2ab): Following the general procedure D.1 with 2-((*tert*-butyldimethylsilyl)oxy)-2-(1-methyl-1*H*-pyrrol-2-yl)-1-phenylethan-1-one (**S2a**) (659 mg, 2 mmol) and 3-ethynylthiophene (0.44 mL, 4.4 mmol), the crude product was purified by column chromatography (hexane/EtOAc, 10/1), affording pure **2ab** as a yellow gel (568 mg, 65%). Obtained and isolated as only one diastereoisomer whose relative configuration was not determined; R_f = 0.23 (hexane/EtOAc, 10/1). **^{1}H NMR** (300 MHz, $CDCl_3$) *δ* (ppm): 7.65–7.56 (m, 2H), 7.55–7.51 (m, 1H), 7.35–7.29 (m, 4H), 7.21 (d, *J* = 5.0 Hz, 1H), 6.49–6.41 (m, 1H), 6.31–6.20 (m, 1H), 6.08 (t, *J* = 3.0 Hz, 1H), 3.32 (s, 3H), 3.28 (bs, 1H), 0.89 (s, 9H), –0.12 (s, 3H), –0.18 (s, 3H). **^{13}C NMR** (75.4 MHz, $CDCl_3$) *δ* (ppm): 141.0 (C), 130.0 (CH), 129.7 (C, bs), 129.0 (CH), 128.0 (CH), 127.7 (2 × CH), 127.0 (2 × CH), 125.4 (CH), 122.6 (CH, bs), 122.1 (C), 110.3 (CH, bs), 107.0 (CH), 91.1 (C), 81.3 (C), 77.1 (C), 34.4 (CH_3, bs), 25.8 (3 × CH_3), 18.3 (C), –5.0 (CH_3), –5.6 (CH_3). The *C*HOSi peak was missing due to restricted rotation. **HRMS** (ESI-TOF): calculated for $C_{25}H_{32}NO_2SSi^+$ $[M+H]^+$ 438.1918; found 438.1913.

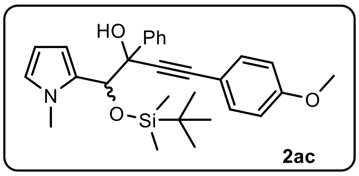

1-((*Tert*-butyldimethylsilyl)oxy)-4-(4-methoxyphenyl)-1-(1-methyl-1H-pyrrol-2-yl)-2-phenylbut-3-yn-2-ol (2ac): Following the general procedure D.1 with 2-((*tert*-butyldimethylsilyl)oxy)-2-(1-methyl-1*H*-pyrrol-2-yl)-1-phenylethan-1-one (**S2a**) (659 mg, 2 mmol) and 4-ethynylanisole (582 mg, 4.4 mmol), the crude product was purified by column chromatography (hexane/EtOAc, 7/1), affording pure **2ac** as a yellow gel (351 mg, 38%). Obtained and isolated as 1.5/1 mixture of diastereoisomers; R_f = 0.36 (hexane/EtOAc, 5/1). Data for both diastereoisomers: **^{1}H NMR** (300 MHz, $CDCl_3$) *δ* (ppm): δ 7.66–7.56 (m, 2H, both), 7.53–7.42 (m, 4H, both), 7.44–7.22 (m, 8H, both), 6.95–6.80 (m, 4H, both), 6.48–6.43 (m, 2H, both), 6.28–6.18 (m, 2H, both), 6.13 (t, *J* = 3.0 Hz, 1H, minor), 6.07 (t, *J* = 3.0 Hz, 1H, major), 4.86 (s, 2H, both), 3.85 (s, 3H, major), 3.83 (s, 3H, minor), 3.40–3.25 (m, 8H, both), 0.90–0.83 (m, 18H, both), –0.14 (s, 6H, both), –0.20 (s, 6H, both). **^{13}C NMR** (75.4 MHz, $CDCl_3$) *δ* (ppm): 159.7 (C), 159.5 (C), 142.8 (C), 141.2 (C), 133.2 (2 × CH), 133.0 (2 × CH), 127.81 (2 × CH), 127.79 (2 × CH), 127.6 (2 × CH), 127.0 (2 × CH), 126.9 (CH), 126.3 (CH), 122.7 (CH, bs), 115.3 (C), 115.1 (C), 114.0, (2 × CH) 113.9 (2 × CH), 109.8 (CH) 109.7 (CH), 107.0 (CH), 106.9 (CH), 90.5 (C), 86.6 (C), 85.9 (C), 84.0 (C), 79.0 (C), 76.9 (CH, bs), 55.3 (CH_3), 55.3 (CH_3), 33.6 (CH_3), 30.2 (CH_3), 25.7 (6 × CH_3), 18.2 (2 × C),–5.1 (2 × CH_3), –5.7 (2 × CH_3). One quaternary peak from the major diastereoisomer and four signals from the minor one were missing. **HRMS** (ESI-TOF): calculated for $C_{28}H_{36}NO_3Si^+$ $[M+H]^+$ 462.2459; found 462.2452.

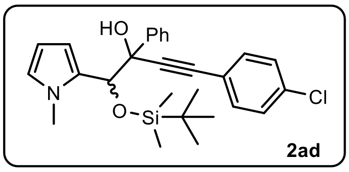

1-((*Tert*-butyldimethylsilyl)oxy)-4-(4-chlorophenyl)-1-(1-methyl-1*H*-pyrrol-2-yl)-2-phenylbut-3-yn-2-ol (2ad): Following the general procedure D.1 with 2-((*tert*-butyldimethylsilyl)oxy)-2-(1-methyl-1*H*-pyrrol-2-yl)-1-phenylethan-1-one (**S2a**) (659 mg, 2 mmol) and 1-chloro-4-ethynylbenzene (600 mg, 4.4 mmol), the crude product was purified by column chromatography (hexane/EtOAc, 10/1), affording pure **2ad** as a yellow gel (587 mg, 63%). Obtained and isolated as only one diastereoisomer whose relative configuration was not determined; R_f = 0.36 (hexane/EtOAc, 10/1). **^{1}H NMR** (300 MHz, $CDCl_3$) δ (ppm): 7.63–7.55 (m, 2H), 7.50–7.43 (m, 2H), 7.40–7.30 (m, 6H), 6.52–6.40 (m, 1H), 6.33–6.20 (m, 1H), 6.13–6.02 (m, 1H), 3.44–3.17 (m, 4H), 0.87 (s, 9H), –0.15 (s, 3H), –0.20 (s, 3H). **^{13}C NMR** (75.4 MHz, $CDCl_3$) δ (ppm): 141.0 (C), 134.5 (C), 133.0 (2 × CH), 129.6 (C, bs), 128.7 (2 × CH), 128.0 (CH), 127.7 (2 × CH), 126.9 (2 × CH), 122.6 (CH, bs), 121.5 (C), 110.2 (CH, bs), 107.0 (CH), 92.5 (C), 84.9 (C), 77.0 (C), 34.3 (CH_3, bs), 25.8 (3 × CH_3), 18.2 (C), –5.0 (CH_3), –5.67 (CH_3). The *C*HOSi peak was missing due to restricted rotation. **HRMS** (ESI-TOF): calculated for $C_{27}H_{33}ClNO_2Si^+$ $[M+H]^+$ 466.1964; found 466.1972.

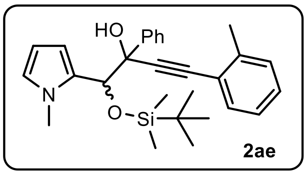

1-((*Tert*-butyldimethylsilyl)oxy)-1-(1-methyl-1H-pyrrol-2-yl)-2-phenyl-4-(*o*-tolyl)but-3-yn-2-ol (2ae): Following the general procedure D.1 with 2-((*tert*-butyldimethylsilyl)oxy)-2-(1-methyl-1*H*-pyrrol-2-yl)-1-phenylethan-1-one (**S2a**) (659 mg, 2 mmol) and 2-ethynyltoluene (0.55 mL, 4.4 mmol), the crude product was purified by column chromatography (hexane/EtOAc, 10/1), affording pure **2ae** as a yellow gel (383 mg, 43%). Obtained and isolated as only one diastereoisomer whose relative configuration was not determined; R_f = 0.37 (hexane/EtOAc, 7/1). **^{1}H NMR** (300 MHz, $CDCl_3$) δ (ppm): 7.75–7.64 (m, 2H), 7.57–7.48 (m, 1H), 7.41–7.13 (m, 6H), 6.53–6.47 (m, 1H), 6.38–6.25 (m, 1H), 6.13–6.06 (m, 1H), 4.88 (s, 1H), 3.41 (bs, 3H), 3.24–3.16 (m, 1H), 2.55 (s, 3H), 0.85 (s, 9H), –0.18 (s, 3H), –0.22 (s, 3H). **^{13}C NMR** (75.4 MHz, $CDCl_3$) δ (ppm): 141.6 (C), 140.5 (C), 132.2 (CH), 129.6 (CH), 128.4 (CH), 127.9 (CH), 127.7 (2 × CH), 127.1 (2 × CH), 125.6 (CH), 122.8 (C), 122.7 (CH, bs), 110.3 (CH, bs), 107.0 (CH), 95.1 (C), 85.4 (C), 77.3 (C), 34.6 (CH_3), 25.8 (3 × CH_3), 21.1 (CH_3), 18.3 (C), –5.10 (CH_3), –5.7 (CH_3). One quaternary peak and the *C*HOSi peak were missing. **HRMS** (ESI-TOF): calculated for $C_{28}H_{36}NO_2Si^+$ $[M+H]^+$ 446.2510; found 446.2518.

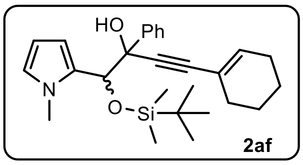

1-((*Tert*-butyldimethylsilyl)oxy)-4-(cyclohex-1-en-1-yl)-1-(1-methyl-1H-pyrrol-2-yl)-2-phenylbut-3-yn-2-ol (2af): Following the general procedure D.1 with 2-((*tert*-butyldimethylsilyl)oxy)-2-(1-methyl-1*H*-pyrrol-2-yl)-1-phenylethan-1-one (**S2a**) (659 mg, 2 mmol) and 1-ethynylcyclohexene (0.52 mL, 4.4 mmol), the crude product was purified by column chromatography (hexane/EtOAc, 10/1), affording pure **2ae** as a yellow gel (488 mg, 56%). Obtained and isolated as only one diastereoisomer whose relative configuration was not determined; R_f = 0.29 (hexane/EtOAc, 10/1). **^{1}H NMR** (300 MHz, $CDCl_3$) δ (ppm): 7.64–7.48 (m, 2H), 7.34–7.23 (m, 3H), 6.51–6.39 (m, 1H), 6.29–6.18 (m, 2H), 6.1–6.04 (m, 1H), 4.80 (s, 1H), 3.30 (bs, 3H), 3.18 (s, 1H), 2.31–2.12 (m, 4H), 1.78–1.59 (m, 4H), 0.88 (s, 9H), –0.14 (s, 3H),–0.20 (s, 3H). **^{13}C NMR** (75.4 MHz, $CDCl_3$) δ (ppm): 141.5 (C), 135.0 (CH), 129.9 (C, bs),

127.7 (CH), 127.5 (2 × CH), 127.0 (2 × CH), 122.4 (CH, bs), 120.5 (C), 110.2 (CH, bs), 106.8 (CH), 88.5 (C), 88.0 (C), 76.8 (C), 34.4 (CH_3), 29.2 (CH_2), 25.8 (3 × CH_3), 25.7 (CH_2), 22.4 (CH_2), 21.6 (CH_2), 18.2 (C), -5.1 (CH_3), -5.7 (CH_3). The *C*HOSi peak was missing due to restricted rotation. **HRMS** (ESI-TOF): calculated for $C_{27}H_{38}NO_2Si^+$ $[M+H]^+$ 436.2666; found 436.2663.

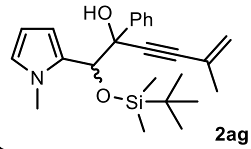

2ag

1-((*Tert*-butyldimethylsilyl)oxy)-5-methyl-1-(1-methyl-1*H*-pyrrol-2-yl)-2-phenylhex-5-en-3-yn-2-ol (2ag): Following the general procedure D.1 with 2-((*tert*-butyldimethylsilyl)oxy)-2-(1-methyl-1*H*-pyrrol-2-yl)-1-phenylethan-1-one (**S2a**) (659 mg, 2 mmol) and 2-methylbut-1-en-3-yne (0.42 mL, 4.4 mmol), the crude product was purified by column chromatography (hexane/EtOAc, 7/1), affording pure **2ag** as a yellow gel (427 mg, 54%). Obtained and isolated as only one diastereoisomer whose relative configuration was not established; R_f = 0.40 (hexane/EtOAc, 7/1). **^{1}H NMR** (300 MHz, $CDCl_3$) δ (ppm): 7.60–7.50 (m, 2H), 7.37–7.25 (m, 3H), 6.49–6.40 (m, 1H), 6.28–6.18 (m, 1H), 6.11–6.03 (m, 1H), 5.45–5.39 (m, 1H), 5.37–5.28 (m, 1H), 4.81 (s, 1H), 3.27 (bs, 3H), 3.26–3.19 (m, 1H), 2.00 (s, 3H), 0.89 (s, 9H), –0.14 (s, 3H), –0.19 (s, 3H). **^{13}C NMR** (75.4 MHz, $CDCl_3$) δ (ppm): 141.1 (C), 129.7 (C, bs), 127.9 (CH), 127.6 (2 × CH), 127.0 (2 × CH), 126.6 (C), 122.6 (CH, bs), 122.2 (CH_2), 110.2 (CH, bs), 106.9 (CH), 90.5 (C), 87.3 (C), 76.8 (C), 34.3 (CH_3, bs), 25.8 (3 × CH_3), 23.5 (CH_3), 18.3 (C), –5.0 (CH_3), –5.7 (CH_3). The *C*HOSi peak was missing due to restricted rotation. **HRMS** (ESI-TOF): calculated for $C_{24}H_{34}NO_2Si^+$ $[M+H]^+$ 396.2353; found 396.2351.

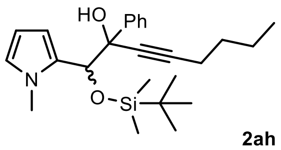

2ah

1-((*Tert*-butyldimethylsilyl)oxy)-1-(1-methyl-1*H*-pyrrol-2-yl)-2-phenyloct-3-yn-2-ol (2ah): Following the general procedure D.2 with 1-(1-methyl-1*H*-pyrrol-2-yl)-2-phenyloct-3-yne-1,2-diol (**1ah**) (297 mg, 1 mmol) for 4 h, the crude product was purified by column chromatography (hexane/EtOAc, 7/1), affording pure **2ah** as a yellow gel (160 mg, 39%). Obtained and isolated as only one diastereoisomer whose relative configuration was not determined; R_f = 0.42 (hexane/EtOAc, 10/1). **^{1}H NMR** (300 MHz, $CDCl_3$) δ (ppm): 7.61–7.47 (m, 2H), 7.37–7.20 (m, 3H), 6.51–6.38 (m, 1H), 6.24–6.11 (m, 1H), 6.05 (at, *J* = 3.0 Hz, 1H), 4.75 (s, 1H), 3.27 (bs, 3H), 3.11 (s, 1H), 2.35 (t, *J* = 7.0 Hz, 2H), 1.70–1.43 (m, 4H), 0.98 (t, *J* = 7.0 Hz, 3H), 0.86 (s, 9H), –0.17 (s, 3H), –0.22 (s, 3H). **^{13}C NMR** (75.4 MHz, $CDCl_3$) δ (ppm): 141.7 (C), 130.1 (C, bs), 127.7 (CH), 127.5 (2 × CH), 127.1 (2 × CH), 122.4 (CH, bs), 110.1 (CH, bs), 106.8 (CH), 86.8 (C), 82.1 (C), 76.6 (C), 34.4 (CH_3, bs), 30.8 (CH_2), 25.8 (3 × CH_3), 22.2 (CH_2), 18.8 (CH_2), 18.3 (C), 13.8 (CH_3), –5.0 (CH_3), –5.7 (CH_3). The *C*HOSi peak was missing due to restricted rotation. **HRMS** (ESI-TOF): calculated for $C_{25}H_{38}NO_2Si^+$ $[M+H]^+$ 412.2666; found 412.2676.

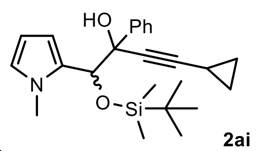

2ai

1-((*Ter*t-butyldimethylsilyl)oxy)-4-cyclopropyl-1-(1-methyl-1*H*-pyrrol-2-yl)-2-phenylbut-3-yn-2-ol (2ai): Following the general procedure D.2 with 4-cyclopropyl-1-(1-methyl-1*H*-pyrrol-2-yl)-2-phenylbut-3-yne-1,2-diol (**1ai**) (281 mg, 1 mmol) for 4 h, the crude product was purified by column chromatography (hexane/EtOAc, 7/1), affording pure **2ai** as an orange gel (158 mg, 40%). Obtained and isolated as only one diastereoisomer whose relative configuration was not determined; R_f = 0.38

(hexane/EtOAc, 7/1). **^{1}H NMR** (300 MHz, $CDCl_3$) δ (ppm): 7.57–7.46 (m, 2H), 7.33–7.24 (m, 3H), 6.50–6.36 (m, 1H), 6.23–6.11 (m, 1H), 6.09–6.01 (m, 1H), 4.75 (s, 1H), 3.25 (bs, 3H), 3.17–3.04 (m, 1H), 1.50–1.28 (m, 1H), 0.96–0.60 (m, 13H), –0.14 (s, 3H), –0.20 (s, 3H).**^{13}C NMR** (75.4 MHz, $CDCl_3$) δ (ppm): 141.5 (C), 129.9 (C, bs), 127.7 (CH), 127.5 (2 × CH), 127.0 (2 × CH), 122.3 (CH, bs), 110.0 (CH, bs), 106.8 (CH), 89.6 (C), 77.4 (C), 76.5 (C), 34.3 (CH_3, bs), 25.8 (3 × CH_3), 18.3 (C), 8.2 (CH_2), 8.1 (CH_2), –0.2 (CH), –5.0 (CH_3), –5.7 (CH_3). The *C*HOSi peak was missing due to restricted rotation. **HRMS** (ESI-TOF): calculated for $C_{24}H_{34}NO_2Si^+$ $[M+H]^+$ 396.2353; found 396.2350.

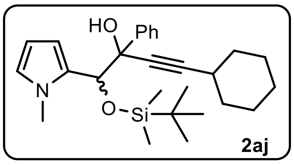

1-((*Tert*-butyldimethylsilyl)oxy)-4-cyclohexyl-1-(1-methyl-1H-pyrrol-2-yl)-2-phenylbut-3-yn-2-ol (2aj): Following the general procedure D.2 with 4-cyclohexyl-1-(1-methyl-1*H*-pyrrol-2-yl)-2-phenylbut-3-yne-1,2-diol (**1aj**) (323 mg, 1 mmol) for 4 h, the crude product was purified by column chromatography (hexane/EtOAc, 10/1), affording pure **2aj** as a yellow gel (162 mg, 37%). Obtained and isolated as only one diastereoisomer whose relative configuration was not determined; R_f = 0.43 (hexane/EtOAc, 10/1). **^{1}H NMR** (300 MHz, $CDCl_3$) δ (ppm): 7.61–7.50 (m, 2H), 7.32–7.22 (m, 3H), 6.44 (t, J = 2.3 Hz, 1H), 6.30–6.13 (m, 1H), 6.05 (t, J = 3.0 Hz, 1H), 4.74 (s, 1H), 3.30 (bs, 3H), 3.05 (s, 1H), 2.53 (tt, J = 9.3, 3.8 Hz, 1H), 2.03–1.71 (m, 4H), 1.67–1.27 (m, 6H), 0.86 (s, 9H), –0.20 (s, 3H), –0.24 (s, 3H). **^{13}C NMR** (75.4 MHz, $CDCl_3$) δ (ppm): 142.0 (C), 130.2 (C, bs), 127.6 (CH), 127.5 (2 × CH), 127.1 (2 × CH), 122.3 (CH, bs), 110.1 (CH, bs), 106.8 (CH), 90.9 (C), 81.9 (C), 76.6 (C), 34.4 (CH_3, bs), 32.7 (2 × CH_2), 29.4 (CH), 26.0 (CH_2), 25.8 (3 × CH_3), 25.1 (2 × CH_2), 18.2 (C), -5.1 (CH_3), -5.7 (CH_3). The *C*HOSi peak was missing due to restricted rotation. **HRMS** (ESI-TOF): calculated for $C_{27}H_{40}NO_2Si^+$ $[M+H]^+$ 438.2823; found 438.2830.

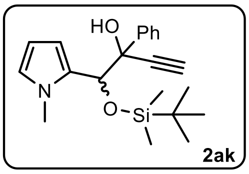

1-((*T*ert-butyldimethylsilyl)oxy)-1-(1-methyl-1H-pyrrol-2-yl)-2-phenylbut-3-yn-2-ol (2ak): Following the general procedure D.1 with 2-((*tert*-butyldimethylsilyl)oxy)-2-(1-methyl-1*H*-pyrrol-2-yl)-1-phenylethan-1-one (**S2a**) (659 mg, 2 mmol) and ethynyl magnesium bromide (4 mmol, 8 mL of a 0.5 M solution in THF), the crude product was purified by column chromatography (hexane/EtOAc, 10/1), affording pure **2ak** as a yellow gel (320 mg, 45%). Obtained and isolated as only one diastereoisomer whose relative configuration was not determined; R_f = 0.35 (hexane/EtOAc, 10/1). **^{1}H NMR** (300 MHz, $CDCl_3$) δ (ppm): 7.59–7.50 (m, 2H), 7.35–7.24 (m, 3H), 6.57–6.38 (m, 1H), 6.31–6.16 (m, 1H), 6.10–6.03 (m, 1H), 4.79 (s, 1H), 3.32–3.19 (m, 4H), 2.68 (s, 1H), 0.88 (s, 9H), –0.13 (s, 3H), –0.20 (s, 3H). **^{13}C NMR** (75.4 MHz, $CDCl_3$) δ (ppm): 140.5 (C), 129.3 (C, bs), 128.0 (CH), 127.7 (2 × CH), 126.9 (2 × CH), 122.6 (CH, bs), 110.3 (CH, bs), 107.0 (CH), 86.0 (C), 76.5 (C), 74.4 (CH), 34.4 (CH_3, bs), 25.8 (3 × CH_3), 18.3 (C), -5.0 (CH_3), -5.6 (CH_3). The *C*HOSi peak was missing due to restricted rotation. **HRMS** (ESI-TOF): calculated for $C_{21}H_{30}NO_2Si^+$ $[M+H]^+$ 356.2040; found 356.2050.

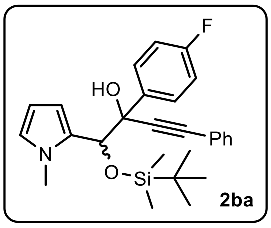

1-((*Tert*-butyldimethylsilyl)oxy)-2-(4-fluorophenyl)-1-(1-methyl-1*H*-pyrrol-2-yl)-4-phenylbut-3-yn-2-ol (2ba): Following the general procedure D.1 with 2-((*tert*-butyldimethylsilyl)oxy)-1-(4-fluorophenyl)-2-(1-methyl-1*H*-pyrrol-2-yl)ethan-1-one (**S2b**) (695 mg, 2 mmol) and phenylacetylene (0.48 mL, 4.4 mmol), the crude product was purified by column chromatography (hexane/EtOAc, 10/1), affording pure **2ba** as a yellow gel (539 mg, 60%). Obtained and isolated as only one diastereoisomer whose relative configuration was not determined; R_f = 0.34 (hexane/EtOAc, 10/1). **^{1}H NMR** (300 MHz, $CDCl_3$) δ (ppm): 7.71–7.52 (m, 4H), 7.45–7.34 (m, 3H), 7.11–6.95 (m, 2H), 6.56–6.43 (m, 1H), 6.39–6.19 (m, 1H), 6.15–6.07 (m, 1H), 4.87 (s, 1H), 3.50–3.27 (m, 4H), 0.91 (s, 9H), –0.09 (s, 3H), –0.15 (s, 3H). **^{13}C NMR** (75.4 MHz, $CDCl_3$) δ (ppm): 162.7 (d, $^1J_{C–F}$ = 246.2 Hz, C), 137.0 (C), 131.8 (2 × CH), 129.5 (C, bs), 128.9 (d, $^3J_{C–F}$ = 8.1 Hz, 2 × CH), 128.6 (CH), 128.5 (2 × CH), 122.83 (CH, bs), 122.75 (C, bs), 114.4 (d, $^2J_{C–F}$ = 21.4 Hz, 2 × CH), 110.4 (CH, bs), 107.1 (CH), 91.2 (C), 86.3 (C), 76.6 (C), 34.5 (CH_3, bs), 25.8 (3 × CH_3), 18.3 (C), –5.0 (CH_3), –5.6 (CH_3). The *C*HOSi peak was missing due to restricted rotation. **HRMS** (ESI-TOF): calculated for $C_{27}H_{33}FNO_2Si^+$ $[M+H]^+$ 450.2259; found 450.2269.

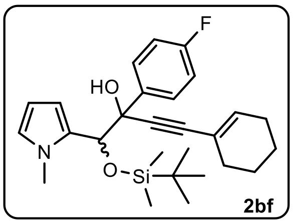

1-((*Tert*-butyldimethylsilyl)oxy)-4-(cyclohex-1-en-1-yl)-2-(4-fluorophenyl)-1-(1-methyl-1*H*-pyrrol-2-yl)but-3-yn-2-ol (2bf): Following the general procedure D.1 with 2-((*tert*-butyldimethylsilyl)oxy)-1-(4-fluorophenyl)-2-(1-methyl-1*H*-pyrrol-2-yl)ethan-1-one (**S2b**) (695 mg, 2 mmol) and 1-ethynylcyclohexene (0.52 mL, 4.4 mmol), the crude product was purified by column chromatography (hexane/EtOAc, 10/1), affording pure **2bf** as a yellow gel (544 mg, 60%). Obtained and isolated as only one diastereoisomer whose relative configuration was not determined; R_f = 0.33 (hexane/EtOAc, 10/1). **^{1}H NMR** (300 MHz, $CDCl_3$) δ (ppm): 7.54–7.42 (m, 2H), 7.01–6.87 (m, 2H), 6.46–6.37 (m, 1H), 6.22–6.09 (m, 2H), 6.06–5.98 (m, 1H), 4.72 (s, 1H), 3.29 (bs, 3H), 3.16 (s, 1H), 2.27–2.05 (m, 4H), 1.75–1.56 (m, 4H), 0.84 (s, 9H), –0.17 (s, 3H), –0.23 (s, 3H). **^{13}C NMR** (75.4 MHz, $CDCl_3$) δ (ppm): 162.6 (d, $^1J_{C–F}$ = 246.2 Hz, C), 137.3 (C), 135.4 (CH), 129.6 (C, bs), 128.9 (d, $^3J_{C–F}$ = 8.1 Hz, 2 × CH), 122.7 (CH, bs), 120.4 (C), 114.3 (d, $^2J_{C–F}$ = 21.4 Hz, 2 × CH), 110.3 (CH, bs), 107.0 (CH), 88.3 (C), 88.2 (C), 76.4 (C), 34.5 (CH_3, bs), 29.2 (CH_2), 25.83 (3 × CH_3), 25.77 (CH_2), 22.4 (CH_2), 21.6 (CH_2), 18.3 (C), –5.0 (CH_3), –5.6 (CH_3). The *C*HOSi peak was missing due to restricted rotation. **HRMS** (ESI-TOF): calculated for $C_{27}H_{37}FNO_2Si^+$ $[M+H]^+$ 454.2572; found 454.2581.

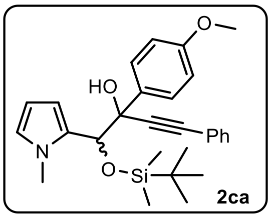

1-((*Tert*-butyldimethylsilyl)oxy)-2-(4-methoxyphenyl)-1-(1-methyl-1*H*-pyrrol-2-yl)-4-phenylbut-3-yn-2-ol (2ca): Following the general procedure D.1 with 2-((*tert*-butyldimethylsilyl)oxy)-1-(4-methoxyphenyl)-2-(1-methyl-1*H*-pyrrol-2-yl)ethan-1-one (**S2c**) (719 mg, 2 mmol) and phenylacetylene (0.48 mL, 4.4 mmol), the crude product was purified by column chromatography (hexane/EtOAc, 7/1), affording pure **2ca** as a yellow gel (508 mg, 55%). Obtained and isolated as only one diastereoisomer whose relative configuration was not determined; R_f = 0.32 (hexane/EtOAc, 7/1). **^{1}H NMR** (300 MHz, $CDCl_3$) δ (ppm): 7.59–7.46 (m, 4H), 7.40–7.31 (m, 3H), 6.90–6.76 (m, 2H), 6.55–6.39 (m, 1H), 6.32–6.21 (m, 1H), 6.13–6.03 (m, 1H), 4.85 (s, 1H), 3.81 (s, 3H),

3.38–3.32 (s, 1H), 3.27 (bs, 3H), 0.89 (s, 9H), –0.10 (s, 3H), –0.17 (s, 3H). **^{13}C NMR** (75.4 MHz, $CDCl_3$) δ (ppm): 159.4 (C), 133.3 (C), 131.8 (2 × CH), 129.8 (C, bs), 128.4 (2 × CH), 128.3 (2 × CH), 123.1 (C), 122.5 (CH, bs), 113.0 (2 × CH), 110.2 (CH, bs), 106.9 (CH), 91.7 (C), 85.9 (C), 76.6 (C), 55.4 (CH_3), 34.5 (CH_3, bs), 25.8 (3 × CH_3), 18.3 (C), –5.0 (CH_3), –5.5 (CH_3). The *C*HOSi peak was missing due to restricted rotation. **HRMS** (ESI-TOF): calculated for $C_{28}H_{36}NO_3Si^+$ $[M+H]^+$ 462.2459; found 462.2460.

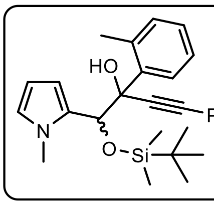

2da

1-((*Tert*-butyldimethylsilyl)oxy)-1-(1-methyl-1*H*-pyrrol-2-yl)-4-phenyl-2-(o-tolyl)but-3-yn-2-ol (2da): Following the general procedure D.1 with 2-((*tert*-butyldimethylsilyl)oxy)-2-(1-methyl-1*H*-pyrrol-2-yl)-1-(*o*-tolyl)ethan-1-one (**S2d**) (687 mg, 2 mmol) and phenylacetylene (0.48 mL, 4.4 mmol), affording **2da** as a yellow gel (321 mg, 36%). Obtained as only one diastereoisomer whose relative configuration was not determined; R_f = 0.40 (hexane/EtOAc, 7/1). **2da** was used in the cyclization step without further purification.

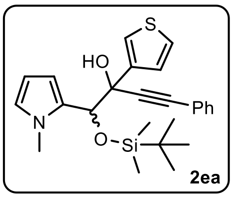

2ea

1-((*Tert*-butyldimethylsilyl)oxy)-1-(1-methyl-1*H*-pyrrol-2-yl)-4-phenyl-2-(thiophen-3-yl)but-3-yn-2-ol (2ea): Following the general procedure D.1 with 2-((*tert*-butyldimethylsilyl)oxy)-2-(1-methyl-1*H*-pyrrol-2-yl)-1-(thiophen-3-yl)ethan-1-one (**S2e**) (671 mg, 2 mmol) and phenylacetylene (0.48 mL, 4.4 mmol), the crude product was purified by column chromatography (hexane/EtOAc, 7/1), affording pure **2ea** as a yellow gel (621 mg, 71%). Obtained and isolated as only one diastereoisomer whose relative configuration was not determined; R_f = 0.33 (hexane/EtOAc, 7/1). **^{1}H NMR** (300 MHz, $CDCl_3$) δ (ppm): 7.56–7.47 (m, 2H), 7.42–7.32 (m, 4H), 7.19 (dd, *J* = 5.0, 3.0 Hz, 1H), 6.98 (d, *J* = 5.0 Hz, 1H), 6.55–6.40 (m, 1H), 6.30–6.17 (m, 1H), 6.13–6.02 (m, 1H), 4.91 (s, 1H), 3.37 (s, 1H), 3.31 (bs, 3H), 0.89 (s, 9H), –0.05 (s, 3H), –0.15 (s, 3H). **^{13}C NMR** (75.4 MHz, $CDCl_3$) δ (ppm): 142.8 (C), 131.8 (2 × CH), 129.7 (C, bs), 128.5 (CH), 128.4 (2 × CH), 127.0 (CH), 124.8 (CH), 123.5 (CH), 122.9 (C), 122.5 (CH, bs), 110.2 (CH, bs), 107.0 (CH), 91.4 (C), 85.3 (C), 75.8 (CH, bs), 74.8 (C), 34.3 (CH_3, bs), 25.8 (3 × CH_3), 18.3 (C), –4.9 (CH_3), –5.5 (CH_3). **HRMS** (ESI-TOF): calculated for $C_{25}H_{32}NO_2SSi^+$ $[M+H]^+$ 438.1918; found 438.1931.

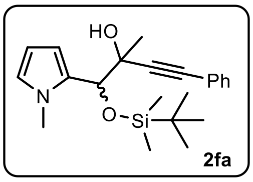

2fa

1-((*Tert*-butyldimethylsilyl)oxy)-2-methyl-1-(1-methyl-1*H*-pyrrol-2-yl)-4-phenylbut-3-yn-2-ol (2fa): Following the general procedure D.1 with 1-((*tert*-butyldimethylsilyl)oxy)-1-(1-methyl-1*H*-pyrrol-2-yl)propan-2-one (**S2f**) (535 mg, 2 mmol) and phenylacetylene (0.48 mL, 4.4 mmol), the crude product was purified by column chromatography (hexane/EtOAc, 7/1), affording pure **2fa** as a yellow gel (429 mg, 58%). Obtained as a 3/1 mixture of diastereoisomers and isolated as only one diastereoisomer whose relative configuration was not determined; R_f = 0.30 (hexane/EtOAc, 7/1). **^{1}H NMR** (300 MHz, $CDCl_3$) δ (ppm): 7.51–7.42 (m, 2H), 7.38–7.30 (m, 3H), 6.63–6.55 (m, 1H), 6.24 (dd, *J* = 3.6, 1.8 Hz, 1H), 6.15–6.09 (m, 1H), 4.84 (s, 1H), 3.75 (s, 3H), 2.96 (s, 1H), 1.53 (s, 3H), 0.96 (s, 9H), 0.12 (s, 3H), –0.09 (s, 3H). **^{13}C NMR** (75.4 MHz, $CDCl_3$) δ (ppm): 131.7 (2 × CH), 130.2 (C), 128.3 (2 × CH), 128.2 (CH), 123.2 (C), 123.0 (C), 110.1 (CH), 107.0 (CH), 92.6 (C), 83.6 (C), 75.3 (CH, bs), 71.8 (C), 35.3 (CH_3, bs), 25.9 (3 × CH_3), 25.6

(CH_3), 18.3 (C), –4.8 (CH_3), –5.3 (CH_3). **HRMS** (ESI-TOF): calculated for $C_{22}H_{32}NO_2Si^+$ $[M+H]^+$ 370.2197; found 370.2191.

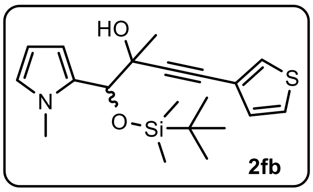

1-((*Tert*-butyldimethylsilyl)oxy)-2-methyl-1-(1-methyl-1*H*-pyrrol-2-yl)-4-(thiophen-3-yl)but-3-yn-2-ol (2fb): Following the general procedure D.1 with 1-((*tert*-butyldimethylsilyl)oxy)-1-(1-methyl-1*H*-pyrrol-2-yl)propan-2-one (**S2f**) (535 mg, 2 mmol) and 3-ethynylthiophene (0.44 mL, 4.4 mmol), the crude product was purified by column chromatography (hexane/EtOAc, 7/1), affording pure **2fb** as a yellow gel (466 mg, 62%). Obtained as a 1/1 mixture of diastereoisomers and isolated as a 1.5/1 mixture of diastereoisomers; R_f = 0.29 (hexane/EtOAc, 7/1). Data for both diastereoisomers: **^{1}H NMR** (300 MHz, $CDCl_3$) δ (ppm): 7.46–7.39 (m, 2H, both), 7.31–7.23 (m, 2H, both), 7.14–7.07 (m, 2H, both), 6.62–6.53 (m, 2H, both), 6.33–6.27 (m, 1H, min.), 6.25–6.20 (m, 1H, maj.), 6.16–6.08 (m, 2H, both), 4.82 (s, 1H, maj.), 4.79 (s, 1H, min.), 3.74 (s, 6H, both), 2.93 (bs, 2H, both), 1.56 (s, 3H, min.), 1.49 (s, 3H, min.), 0.95 (s, 18H, both), 0.11 (s, 3H, maj.), 0.07 (s, 3H, min.), –0.10 (s, 3H, maj.), –0.10 (s, 3H, min.). **^{13}C NMR** (75.4 MHz, $CDCl_3$) δ (ppm): 130.5 (C, bs), 130.1 (C, bs), 129.88 (CH), 129.85 (CH), 128.7 (CH), 128.6 (CH), 125.3 (CH), 125.2 (CH), 123.2 (CH, bs), 122.8 (CH, bs), 122.0 (C), 110.1 (CH, bs), 109.6 (CH, bs), 107.1 (CH), 107.0 (CH), 92.1 (C), 91.4 (C), 79.6 (C), 78.8 (C), 75.3 (CH, bs), 75.0 (CH, bs), 72.2 (C), 71.8 (C), 35.3 (CH_3, bs), 35.0 (CH_3, bs), 25.9 (6 × CH_3), 25.5 (CH_3), 25.4 (CH_3), 18.28 (C), 18.26 (C), -4.7 (CH_3), -4.8 (CH_3), -5.29 (CH_3), -5.34 (CH_3). One quaternary peak was missing due to overlapping. **HRMS** (ESI-TOF): calculated for $C_{20}H_{30}NO_2SSi^+$ $[M+H]^+$ 376.1761; found 376.1768.

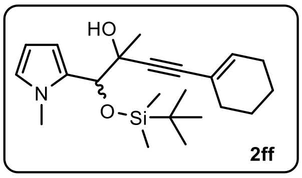

1-((*Tert*-butyldimethylsilyl)oxy)-4-(cyclohex-1-en-1-yl)-2-methyl-1-(1-methyl-1H-pyrrol-2-yl)but-3-yn-2-ol (2ff): Following the general procedure D.1 with 1-((*tert*-butyldimethylsilyl)oxy)-1-(1-methyl-1*H*-pyrrol-2-yl)propan-2-one (**S2f**) (535 mg, 2 mmol) and 1-ethynylcyclohexene (0.52 mL, 4.4 mmol), the crude product was purified by column chromatography (hexane/EtOAc, 7/1), affording pure **2ff** as a yellow gel (434 mg, 58%). Obtained and isolated as a 2/1 mixture of diastereoisomers; R_f = 0.31 (hexane/EtOAc, 7/1). Data for both diastereoisomers: **^{1}H NMR** (300 MHz, $CDCl_3$) δ (ppm): 6.60–6.52 (m, 2H, both), 6.27–6.21 (m, 1H, min.), 6.15 (dd, J = 3.5, 1.8 Hz, 1H, maj.), 6.11–6.03 (m, 4H, both), 4.72 (s, 1H, maj.), 4.69 (s, 1H, min.), 3.71 (s, 6H, both), 2.78 (s, 1H, maj.), 2.75 (s, 1H, min.), 2.16–2.03 (m, 8H, both), 1.70–1.55 (m, 8H, both), 1.47 (s, 3H, min.), 1.40 (s, 3H, maj.), 0.92 (s, 18H, both), 0.07 (s, 3H, maj.), 0.03 (s, 3H, min.), –0.14 (s, 3H, maj.), –0.15 (s, 3H, min.). **^{13}C NMR** (75.4 MHz, $CDCl_3$) δ (ppm): 134.9 (CH), 134.7 (CH), 130.6 (C), 130.3 (C), 123.0 (CH, bs), 122.6 (CH, bs), 120.3 (C), 109.9 (CH, bs), 109.4 (CH, bs), 107.0 (CH), 106.8 (CH), 89.6 (C), 88.8 (C), 86.2 (C), 85.3 (C), 75.3 (2 × CH, bs), 71.9 (C), 71.6 (C), 35.2 (CH_3, bs), 35.0 (CH_3, bs), 29.13 (CH_2), 29.08 (CH_2), 25.8 (6 × CH_3), 25.61 (2 × CH_2), 25.56 (2 × CH_3), 22.3 (2 × CH_2), 21.5 (2 × CH_2), 18.21 (C), 18.17 (C), –4.8 (CH_3), –4.9 (CH_3), –5.39 (CH_3), –5.44 (CH_3). One quaternary peak was missing due to overlapping. **HRMS** (ESI-TOF): calculated for $C_{22}H_{36}NO_2Si^+$ $[M+H]^+$ 374.2510; found 374.2509.

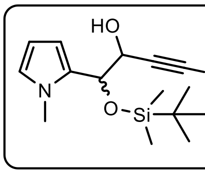
2ga

1-((*Tert*-butyldimethylsilyl)oxy)-1-(1-methyl-1*H*-pyrrol-2-yl)-4-phenylbut-3-yn-2-ol (2ga): Following the general procedure D.3 with phenylacetylene (0.70 mL, 6.4 mmol) the crude product was purified by column chromatography (hexane/EtOAc, 5/1), affording pure **2ga** as a yellow oil (213 mg, 30% referred to starting material **S2g**). Obtained as a 5/1 mixture of diastereoisomers and isolated as only one diastereoisomer whose relative configuration was not determined; R_f = 0.25 (hexane/EtOAc, 5/1). **^{1}H NMR** (300 MHz, $CDCl_3$) δ (ppm): 7.52–7.42 (m, 2H), 7.38–7.30 (m, 3H), 6.66–6.55 (m, 1H), 6.23 (dd, *J* = 3.5, 1.8 Hz, 1H), 6.16–6.08 (m, 1H), 4.88 (d, *J* = 7.0 Hz, 1H), 4.69 (d, *J* = 7.0 Hz, 1H), 2.37–2.04 (m, 1H), 0.91 (s, 9H), 0.09 (s, 3H), –0.04 (s, 3H). **^{13}C NMR** (75.4 MHz, $CDCl_3$) δ (ppm): 131.9 (2 × CH), 130.3 (C), 128.5 (CH), 128.4 (2 × CH), 123.8 (CH), 122.9 (C), 109.9 (CH), 107.3 (CH), 88.5 (C), 85.6 (C), 72.5 (CH), 67.3 (CH), 35.0 (CH_3), 25.8 (3 × CH_3), 18.3 (C), –4.7 (CH_3), –5.1 (CH_3). **HRMS** (ESI-TOF): calculated for $C_{21}H_{30}NO_2Si^+$ [M+H]$^+$ 356.2040; found 356.2050.

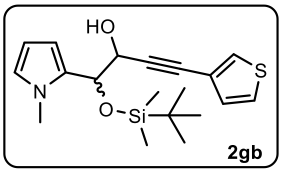
2gb

1-((*Tert*-butyldimethylsilyl)oxy)-1-(1-methyl-1*H*-pyrrol-2-yl)-4-(thiophen-3-yl)but-3-yn-2-ol (2gb): Following the general procedure D.3 with phenylacetylene (0.63 mL, 6.4 mmol) the crude product was purified by column chromatography (hexane/EtOAc, 6/1), affording pure **2gb** as a yellow oil (181 mg, 25% referred to starting material **S2g**). Obtained as a 5/1 mixture of diastereoisomers and isolated as only one diastereoisomer whose relative configuration was not determined; R_f = 0.24 (hexane/EtOAc, 6/1). **^{1}H NMR** (300 MHz, $CDCl_3$) δ (ppm): 7.46 (dd, *J* = 3.1, 1.0 Hz, 1H), 7.28 (dd, *J* = 3.1, 1.8 Hz, 1H), 7.13 (dd, *J* = 5.0, 1.0 Hz, 1H), 6.67–6.53 (m, 1H), 6.21 (dd, *J* = 3.5, 1.8 Hz, 1H), 6.13–6.06 (m, 1H), 4.85 (d, *J* = 7.0 Hz, 1H), 4.66 (dd, *J* = 7.0, 4.9 Hz, 1H), 3.74 (s, 3H), 2.14 (d, *J* = 4.9 Hz, 1H), 0.91 (s, 9H), 0.08 (s, 3H), –0.04 (s, 3H). **^{13}C NMR** (75.4 MHz, $CDCl_3$) δ (ppm): 130.2 (C), 130.0 (CH), 129.1 (CH), 125.3 (CH), 123.90 (CH), 121.9 (C), 109.9 (CH), 107.3 (CH), 88.1 (C), 80.9 (C), 72.6 (CH), 67.2 (CH), 35.0 (CH_3), 25.9 (3 × CH_3), 18.4 (C), –4.7 (CH_3), –5.1 (CH_3). **HRMS** (ESI-TOF): calculated for $C_{19}H_{28}NO_2SSi^+$ [M+H]$^+$ 362.1605; found 362.1610.

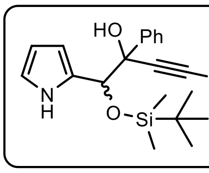
2ha

1-((*Tert*-butyldimethylsilyl)oxy)-2,4-diphenyl-1-(1*H*-pyrrol-2-yl)but-3-yn-2-ol (2ha): Following the general procedure D.2 with 2,4-diphenyl-1-(1*H*-pyrrol-2-yl)but-3-yne-1,2-diol (**1ha**) (303 mg, 1 mmol) for 1 h, the crude product was purified by column chromatography (hexane/EtOAc, 5/1), affording pure **2ha** as a yellow gel (163 mg, 39%). Obtained and isolated as only one diastereoisomer whose relative configuration was not determined; R_f = 0.35 (hexane/EtOAc, 5/1). **^{1}H NMR** (300 MHz, $CDCl_3$) δ (ppm): 8.56 (bs, 1H), 7.73–7.63 (m, 2H), 7.56–7.46 (m, 2H), 7.43–7.27 (m, 6H), 6.85–6.72 (m, 1H), 6.17–6.02 (m, 2H), 4.83 (s, 1H), 3.16 (s, 1H), 0.84 (s, 9H), –0.19 (s, 3H), –0.27 (s, 3H). **^{13}C NMR** (75.4 MHz, $CDCl_3$) δ (ppm): 141.1 (C), 131.9 (2 × CH), 129.3 (C), 128.6 (CH), 128.5 (2 × CH), 127.9 (CH), 127.8 (2 × CH), 126.7 (2 × CH), 122.8 (C), 118.1 (CH), 108.8 (CH), 107.8 (CH), 91.0 (C), 86.2 (C), 77.1 (CH), 76.4 (C), 25.8

(3 × CH_3), 18.2 (C), –5.53 (CH_3), –5.7 (CH_3). **HRMS** (ESI-TOF): calculated for $C_{26}H_{32}NO_2Si^+$ $[M+H]^+$ 418.2197; found 418.2201.

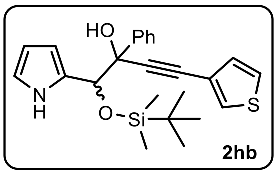

1-((*Tert*-butyldimethylsilyl)oxy)-2-phenyl-1-(1*H*-pyrrol-2-yl)-4-(thiophen-3-yl)but-3-yn-2-ol (2hb): Following the general procedure D.2 with 2-phenyl-1-(1*H*-pyrrol-2-yl)-4-(thiophen-3-yl)but-3-yne-1,2-diol (**1hb**) (309 mg, 1 mmol) for 1 h, the crude product was purified by column chromatography (hexane/EtOAc, 7/1), affording **2hb** as a yellow gel (182 mg, 43%) contaminated with unidentified silicon-derived impurities. Obtained and isolated as only one diastereoisomer whose relative configuration was not determined; R_f = 0.33 (hexane/EtOAc, 5/1). **^{1}H NMR** (300 MHz, $CDCl_3$) δ (ppm): 8.56 (bs, 1H), 7.73–7.60 (m, 2H), 7.54–7.47 (m, 1H), 7.40–7.27 (m, 4H), 7.23–7.13 (m, 1H), 6.83–6.70 (m, 1H), 6.10 (aq, *J* = 2.7 Hz, 1H), 6.07–6.03 (m, 1H), 4.83 (s, 1H), 3.28–3.15 (m, 1H), 0.85 (s, 9H), –0.17 (s, 3H), –0.26 (s, 3H). **^{13}C NMR** (75.4 MHz, $CDCl_3$) δ (ppm): 141.0 (C), 130.0 (CH), 129.22 (C), 129.17 (CH), 127.9 (CH), 127.8 (2 × CH), 126.6 (2 × CH), 125.5 (CH), 121.8 (C), 118.1 (CH), 108.8 (CH), 107.8 (CH), 90.6 (C), 81.3 (C), 77.0 (CH), 76.5 (C), 25.8 (3 × CH_3), 18.2 (C), –5.5 (CH_3), –5.7 (CH_3). **HRMS** (ESI-TOF): calculated for $C_{24}H_{30}NO_2SSi^+$ $[M+H]^+$ 424.1761; found 424.1763.

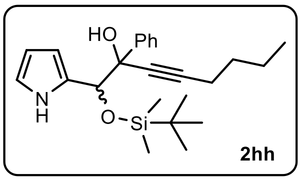

1-((*Tert*-butyldimethylsilyl)oxy)-2-phenyl-1-(1*H*-pyrrol-2-yl)-4-(thiophen-3-yl)but-3-yn-2-ol (2hh): Following the general procedure D.2 with 2-phenyl-1-(1*H*-pyrrol-2-yl)oct-3-yne-1,2-diol (**1hh**) (283 mg, 1 mmol) for 1 h, the crude product was purified by column chromatography (hexane/EtOAc, 7/1), affording pure **2hh** as a yellow gel (227 mg, 57%). Obtained and isolated as only one diastereoisomer whose relative configuration was not determined; R_f = 0.32 (hexane/EtOAc, 7/1). **^{1}H NMR** (300 MHz, $CDCl_3$) δ (ppm): 8.54 (bs, 1H), 7.69–7.53 (m, 2H), 7.40–7.22 (m, 3H), 6.83–6.62 (m, 1H), 6.19–5.91 (m, 2H), 4.71 (s, 1H), 3.05–2.96 (m, 1H), 2.35 (t, *J* = 6.5 Hz, 2H), 1.71–1.43 (m, 4H), 0.99 (t, *J* = 6.0 Hz, 3H), 0.83 (s, 9H),–0.22 (s, 3H), –0.30 (s, 3H). **^{13}C NMR** (75.4 MHz, $CDCl_3$) δ (ppm): 141.7 (C), 129.5 (C), 127.7 (CH), 127.6 (2 × CH), 126.7 (2 × CH), 117.8 (CH), 108.6 (CH), 107.7 (CH), 86.9 (C), 81.8 (C), 77.0 (CH), 75.9 (C), 30.8 (CH_2), 25.8 (3 × CH_3), 22.1 (CH_2), 18.6 (CH_2), 18.2 (C), 13.7 (CH_3), –5.6 (CH_3), –5.8 (CH_3). **HRMS** (ESI-TOF): calculated for $C_{24}H_{36}NO_2Si^+$ $[M+H]^+$ 398.2510; found 398.2515.

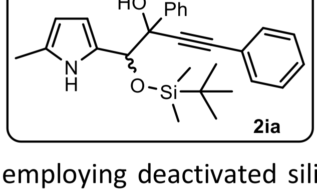

1-((*Tert*-butyldimethylsilyl)oxy)-1-(5-methyl-1*H*-pyrrol-2-yl)-2,4-diphenylbut-3-yn-2-ol (2ia): Following the general procedure D.2 with 1-(5-methyl-1*H*-pyrrol-2-yl)-2,4-diphenylbut-3-yne-1,2-diol (**1ia**) (317 mg, 1 mmol) for 1 h, the crude product was purified by column chromatography employing deactivated silica-gel (hexane/EtOAc, 40/1), affording pure **2ia** as a yellow gel (173 mg, 40%). Obtained and isolated as only one diastereoisomer whose relative configuration was not determined; R_f = 0.29 (hexane/EtOAc, 10/1). **^{1}H NMR** (300 MHz, $CDCl_3$) δ (ppm): 8.23 (bs, 1H), 7.76–7.65 (m, 2H), 7.57–7.46 (m, 2H), 7.40–7.28 (m, 6H), 5.97 (at, *J* = 2.8 Hz, 1H), 5.77 (at, *J* = 2.5 Hz, 1H), 4.71 (s, 1H), 3.16 (s, 1H), 2.24 (s, 3H), 0.82 (s, 9H), –0.23 (s, 3H), –0.29 (s, 3H). **^{13}C NMR** (75.4 MHz,

$CDCl_3$) δ (ppm): 141.3 (C), 131.8 (2 × CH), 128.6 (CH), 128.5 (2 × CH), 128.1 (C), 127.9 (CH), 127.8 (2 × CH), 127.7 (C), 126.8 (2 × CH), 122.9 (C), 109.1 (CH), 105.6 (CH), 91.2 (C), 86.1 (C), 77.3 (CH), 76.3 (C), 25.8 (3 × CH_3), 18.2 (C), 13.2 (CH_3), –5.5 (CH_3), –5.7 (CH_3). **HRMS** (ESI-TOF): calculated for $C_{27}H_{34}NO_2Si^+$ $[M+H]^+$ 432.2353; found 432.2363.

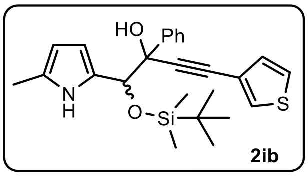

1-((*Tert*-butyldimethylsilyl)oxy)-1-(5-methyl-1*H*-pyrrol-2-yl)-2-phenyl-4-(thiophen-3-yl)but-3-yn-2-ol (2ib): Following the general procedure D.2 with 1-(5-methyl-1*H*-pyrrol-2-yl)-2-phenyl-4-(thiophen-3-yl)but-3-yne-1,2-diol (**1ib**) (323 mg, 1 mmol) for 1 h, the crude product was purified by column chromatography (hexane/EtOAc, 10/1), affording **2ib** as a yellow gel (171 mg, 39%) contaminated with unidentified silicon-derived impurities. Obtained and isolated as only one diastereoisomer whose relative configuration was not determined; R_f = 0.27 (hexane/EtOAc, 10/1). **^{1}H NMR** (300 MHz, $CDCl_3$) δ (ppm): 8.19 (bs, 1H), 7.72–7.61 (m, 2H), 7.50 (dd, *J* = 3.0, 1.2 Hz, 1H), 7.39–7.28 (m, 4H), 7.17 (dd, *J* = 5.0, 1.2 Hz, 1H), 5.93 (at, *J* = 2.9 Hz, 1H), 5.75 (at, *J* = 3.2 Hz, 1H), 4.71 (s, 1H), 3.30–3.11 (m, 1H), 2.24 (s, 3H), 0.83 (s, 9H), –0.21 (s, 3H), –0.28 (s, 3H). **^{13}C NMR** (75.4 MHz, $CDCl_3$) δ (ppm): 141.2 (C), 129.9 (CH), 129.1 (CH), 128.1 (C), 127.9 (CH), 127.8 (2 × CH), 127.7 (C), 126.8 (2 × CH), 125.6 (CH), 121.9 (C), 109.2 (CH), 105.5 (CH), 90.8 (C), 81.2 (C), 77.2 (CH), 76.4 (C), 25.8 (3 × CH_3), 18.2 (C), 13.3 (CH_3),–5.5 (CH_3), –5.6 (CH_3). **HRMS** (ESI-TOF): calculated for $C_{25}H_{32}NO_2SSi^+$ $[M+H]^+$ 438.1918; found 438.1929.

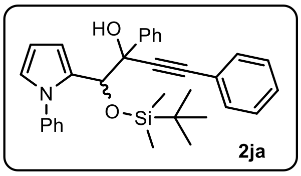

1-((*Tert*-butyldimethylsilyl)oxy)-2,4-diphenyl-1-(1-phenyl-1*H*-pyrrol-2-yl)but-3-yn-2-ol (2ja): Following the general procedure D.1 with 2-((*tert*-butyldimethylsilyl)oxy)-1-phenyl-2-(1-phenyl-1*H*-pyrrol-2-yl)ethan-1-one (**S2j**) (783 mg, 2 mmol) and phenylacetylene (0.48 mL, 4.4 mmol), the crude product was purified by column chromatography (hexane/EtOAc, 10/1), affording pure **2ja** as a yellow gel (592 mg, 60%). Obtained as a 1/1 mixture of diastereoisomers and isolated as a 2/1 mixture of diastereoisomers; R_f = 0.40 (hexane/EtOAc, 10/1). Data for both diastereoisomers: **^{1}H NMR** (300 MHz, $CDCl_3$) δ (ppm): 7.63–7.20 (m, 28H, both), 7.12–7.01 (m, 2H, both), 6.88–6.82 (m, 1H, min), 6.78–6.73 (m, 1H, maj), 6.70 (at, *J* = 2.2 Hz, 1H, maj), 6.65 (at, *J* = 2.2 Hz, 1H, min), 6.62–6.57 (m, 1H, min), 6.36–6.26 (m, 1H, maj), 4.84 (s, 1H, maj), 4.70 (s, 1H, min), 3.84 (s, 1H, min), 3.32 (s, 1H, maj), 0.90 (s, 9H, min), 0.83 (s, 9H, min), –0.20 (s, 3H, min), –0.24 (s, 3H, min), –0.28 (s, 3H, maj), –0.31 (s, 3H, maj). **^{13}C NMR** (75.4 MHz, $CDCl_3$) δ (ppm): 141.4 (C), 140.9 (C), 139.7 (C), 139.5 (C), 131.9 (2 × CH), 131.8 (2 × CH), 131.5 (C), 131.1 (C), 128.9 (2 × CH), 128.8 (2 × CH), 128.41 (4 × CH), 128.37 (2 × CH), 127.9 (CH), 127.8 (2 × CH), 127.7 (2 × CH), 127.44 (CH), 127.36 (CH), 127.1 (2 × CH), 127.02 (2 × CH), 126.98 (2 × CH), 126.9 (2 × CH), 123.2 (C), 123.1 (C), 122.5 (CH), 122.4 (CH), 110.7 (CH), 110.6 (CH), 108.8 (CH), 108.7 (CH), 91.5 (C), 91.0 (C), 86.04 (C), 85.99 (C), 77.6 (2 × CH), 74.8 (C), 74.2 (C), 25.83 (3 × CH_3), 25.77 (3 × CH_3), 18.20 (C), 18.16 (C), -4.7 (CH_3), -5.0 (CH_3), -5.2 (CH_3), -5.4 (CH_3). One CH peak was missing due to overlapping. **HRMS** (ESI-TOF): calculated for $C_{32}H_{36}NO_2Si^+$ $[M+H]^+$ 494.2510; found 494.2519.

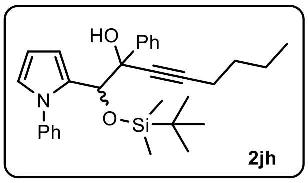

1-((*Tert*-butyldimethylsilyl)oxy)-2-phenyl-1-(1-phenyl-1*H*-pyrrol-2-yl)oct-3-yn-2-ol (2jh): Following the general procedure D.2 with 2-phenyl-1-(1-phenyl-1*H*-pyrrol-2-yl)oct-3-yne-1,2-diol (**1jh**) (360 mg, 1 mmol) for 4 h, the crude product was purified by column chromatography (hexane/EtOAc, 15/1), affording pure **2jh** as a yellow gel (308 mg, 65%). Obtained and isolated as only one diastereoisomer whose relative configuration was not determined; R_f = 0.46 (hexane/EtOAc, 10/1). **^{1}H NMR** (300 MHz, $CDCl_3$) δ (ppm): 7.52–7.31 (m, 5H), 7.26–7.20 (m, 3H), 7.09–7.02 (m, 2H), 6.74–6.57 (m, 2H), 6.27 (at, *J* = 2.6 Hz, 1H), 4.69 (s, 1H), 3.07 (s, 1H), 2.34 (t, *J* = 6.7 Hz, 2H), 1.71–1.41 (m, 4H), 0.95 (t, *J* = 7.1 Hz, 3H), 0.78 (s, 9H), –0.36 (s, 3H), –0.39 (s, 3H). **^{13}C NMR** (75.4 MHz, $CDCl_3$) δ (ppm): 142.1 (C), 139.8 (C), 131.8 (C), 128.8 (2 × CH), 127.62 (CH), 127.59 (2 × CH), 127.3 (CH), 127.1 (2 × CH), 127.0 (2 × CH), 122.4 (CH), 110.5 (CH), 108.6 (CH), 86.9 (C), 82.0 (C), 77.1 (CH), 74.1 (C), 30.8 (CH_2), 25.7 (3 × CH_3), 22.2 (CH_2), 18.8 (CH_2), 18.2 (C), 13.8 (CH_3), –5.1 (CH_3), –5.5 (CH_3). **HRMS** (ESI-TOF): calculated for $C_{30}H_{40}NO_2Si^+$ $[M+H]^+$ 474.2823; found 474.2834.

Gold-catalyzed cyclization of glycol 1aa

Optimization studies

As pointed out in the manuscript, a comprehensive investigation into the reaction parameters governing the gold-catalyzed cyclization of glycols **1** was performed. The following *Table S1* includes the detailed optimization process carried out with selected glycol **1aa**, considering the influence of different parameters in the selectivity of the reaction for the synthesis of the corresponding 4-hydroxyindole **3aa**, 5-hydroxyindole **4aa** and furan derivative **6aa**.

The model reaction of glycol **1aa** in the presence of different metal-catalysts was chosen, modifying temperature reaction and solvents. Initially, drawing from our prior experience, we selected DCM as the solvent, employing PPh_3AuNTf_2 (5 mol%) as the catalyst. Under these reaction conditions, after 30 min we observed a 1/1 mixture of 4-hydroxyindole **3aa** and 5-hydroxyindole **4aa**, albeit with a low overall yield (entry 1). Decreasing the reaction temperature to 0 °C, while retaining PPh_3AuNTf_2 as catalyst, resulted in a slightly higher yield and a cleaner reaction crude. Nevertheless, it had no impact on the regioisomer ratio of the hydroxyindole derivatives **3aa**/**4aa** (entry 2). So, we decided to further evaluate a selection of gold (I) complexes as potential catalysts for the proposed cyclization. When using $IPrAuNTf_2$ as catalyst, we noted a significant improvement in the overall yield, with the regioisomer ratio favoring 5-hydroxyindole formation (1/1.6). Gratifyingly, decreasing the reaction temperature to 0 °C resulted in an increased overall yield, although it had no effect on the regioisomer ratio (entries 3 and 4). Under these conditions, we observed traces (< 5%) of furan derivative **6aa,** which is likely derived from the direct nucleophilic attack of the secondary hydroxyl group in **1aa** onto the triple bond activated by the gold catalyst. Similar results were obtained using $IPrAuMeCNBF_4$ as catalyst under the same reaction conditions (entry 5).

*Table S1: Evaluation of the gold-catalyzed cyclization of glycol **1aa***

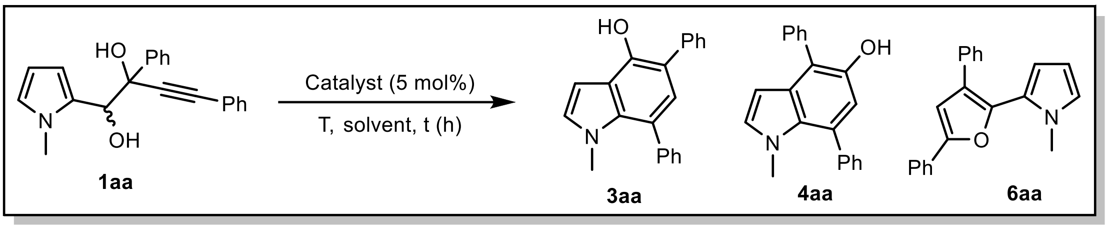

entry	catalyst	T (°C)	t (h)	solvent	r.r. 3aa:4aa[a]	yield (%)[b]	6aa (%)
1	PPh_3AuNTf_2	rt	0.5	DCM	1/1	18	–
2	PPh_3AuNTf_2	0	0.5	DCM	1/1	30	–
3	$IPrAuNTf_2$	rt	0.5	DCM	1/1.6	71	<5
**4**	**$IPrAuNTf_2$**	**0**	**0.5**	**DCM**	**1/1.6**	**80**	**<5**
5	$IPrAuMeCNBF_4$	0	0.5	DCM	1/1.6	78	<5
6	$MorDalPhosAuNTf_2$	0	0.5	DCM	1/1	74	6
7	$JohnPhosAuMeCNSbF_6$	0	0.5	DCM	1/1.1	71	<5
8	$XphosAuNTf_2$	0	0.5	DCM	1.5/1	66	<5
9	$SphosAuNTf_2$	0	0.5	DCM	2.3/1	58	5
**10**	**$SPhosAuMeCNSbF_6$**	**0**	**0.5**	**DCM**	**3/1**	**78**	**5**
11	$BrettPhosAuNTf_2$	0	0.5	DCM	1.5/1	65	5
12	AuCl	0	0.5	DCM	–[c]	–	–
13	$IPrAuCl/AgNTf_2$	0	0.5	DCM	1/1.2	54	<5
14	$IPrAuCl/AgBF_4$	0	0.5	DCM	1/2,3	23	<5
15	$IPrAuCl/AgSbF_6$	0	0.5	DCM	1/1,3	57	<5
16[d]	$(ArO)_3PAuCl/AgOTf$	0	0.5	DCM	–[c]	–	–
17	$AgSbF_6$	0	3	DCM	–[c]	–	–
18	$NaAuCl_4·2H_2O$	rt	0.5	DCM	–[c]	–	–
19	$IPrAuNTf_2$	–30	1	DCM	1/1,2	78	<1
20	$SPhosAuMeCNSbF_6$	–30	1	DCM	3/1	72	<1
21	$IPrAuNTf_2/PTSA$	0	0.5	DCM	1/1,2	24	–
22	$IPrAuNTf_2$	0	0.5	Toluene	1,7/1	55	20
23	IPrAuCl	0	1	HFIP	3/1	35	–

[a]Regioisomeric ractio determined by ^{1}H NMR analysis of the crude. [b]Overall yield of **3aa** and **4aa** determined by ^{1}H NMR with CH_2Br_2 as internal standard. [c]Only decomposition or unidentified products were observed. [d]Ar = 2,4-*t*-$Bu_2C_6H_3$.

Some experiments were conducted involving the use of various commercially available gold(I) complexes with different phosphine ligands, maintaining DCM as the solvent at 0°C (entries 6–11). Complete conversion towards the formation of **3aa** and **4aa** occurred in all cases. Remarkably, the best result was obtained when employing $SPhosAuMeCNSbF_6$ as catalyst, leading to a 3/1 regioisomer ratio favoring the formation of the 4-hydroxyindole derivative **3aa** with an overall yield of 78%, although 5% of furan derivative **6aa** was also observed (entry 10). On the other hand, AuCl was not able to promote cyclization, resulting in unidentified products in the reaction mixture (entry 12). In order to study the

effect of silver counter anions, we selected IPrAuCl bearing a chloride as gold(I) pre-catalyst (entries 13–15). Unfortunately, the overall yield of the transformation was lower, and the regioselectivity of the cyclization was not controlled, resulting in reaction mixtures with significant decomposition. In addition, when we used as catalyst a gold(I) chloride complex bearing a phosphite ligand along with AgOTf, only unidentified decomposition products were observed (entry 16). Additionally, the utilization of a gold(III) complex such as $NaAuCl_4 \cdot 2H_2O$ with $AgSbF_6$ also led to decomposition. At this stage, the most favorable outcomes in the cyclization process were attained when utilizing $SPhosAuMeCNSBF_6$ as catalyst, resulting in a 3/1 selectivity favoring the formation of 4-hydroxyindole **3aa**, employing DCM as the solvent at 0°C (entry 10). Conversely, when employing $IPrAuNTf_2$ under identical reaction conditions, we achieved a slightly improved selectivity in the formation of the **4aa** indole derivative (r.r: 1/1.6, entry 4). With these findings in mind, we attempted to lower the temperature to –30°C with both catalysts in an effort to enhance the regioselectivity. However, the regioisomeric ratio did not appreciably change (entries 19 and 20). On the other hand, considering that a pinacol-type rearrangement is involved in the formation of 5-hydroxyindole **4aa**, we hypothesized that the introduction of a Brønsted acid such as PTSA into the reaction mixture could favor the formation of **4aa**. However, we observed a 1/1.2 ratio of **3aa**/**4aa** with a low overall yield (entry 21). Subsequently, we tested toluene as a solvent with $IPrAuNTf_2$ as catalyst (entry 22). Despite achieving full conversion in the benzannulation process, the reaction yield decreased to 55%, without improving the regioselectivity, likely due to the competitive formation of furan derivative **6aa** (20%). Finally, we attempted HFIP as a solvent in the presence of IPrAuCl, resulting in a 3/1 regioisomeric ratio, but with a moderate yield (entry 23).[2]

Synthesis and characterization data of 4-hydroxyindoles 3 and 5-hydroxyindoles 4

General procedure E for the gold-catalyzed cyclization of diols 1

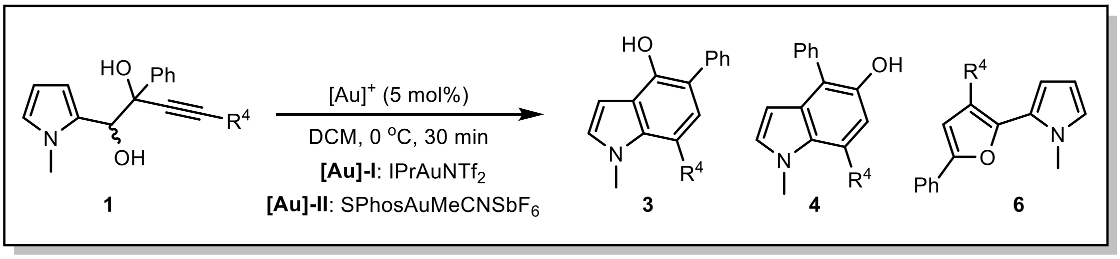

Catalyst **[Au]-I** or **[Au]-II** (0.01 mmol, 0.05 equiv) was added to a solution of the corresponding alkynol **1** (0.2 mmol, 1 equiv) in anhydrous DCM (2 mL, 0.1 M) with continuous stirring at 0 °C. After 30 min, DCM was removed under reduced pressure and the residue was purified directly by flash column chromatography using hexane and EtOAc mixtures as eluent, affording the corresponding 4-hydroxyindoles **3**, 5-hydroxyindoles **4** and furan derivatives **6**.

[2] Tzouras, N. V.; Zorba, L. P.; Kaplanai, E.; Tsoureas, N.; Nelson, D. J.; Nolan, S. P.; Vougioukalakis, G. C. *ACS Catal.* **2023**, *13,* 8845–8860.

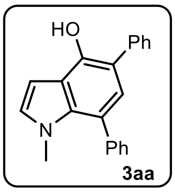
3aa

1-Methyl-5,7-diphenyl-1*H*-indol-4-ol (3aa): Following the general procedure E using SPhosAuMeCNSbF_6 (**[Au]-II**) with 1-(1-methyl-1*H*-pyrrol-2-yl)-2,4-diphenylbut-3-yne-1,2-diol (**1aa**), the crude product was obtained as a 3/1 (**3aa/4aa**) mixture of regioisomers, and was purified by column chromatography (hexane/EtOAc, 5/1), affording pure **3aa** as a pale pink solid (31 mg, 51%); mp = 122–124 °C; R_f = 0.25 (hexane/EtOAc, 5/1). **^{1}H NMR** (300 MHz, $CDCl_3$) *δ* (ppm): 7.63–7.31 (m, 10H), 6.99 (s, 1H), 6.96 (d, *J* = 3.2 Hz, 1H), 6.71 (d, *J* = 3.2 Hz, 1H), 5.60 (s, 1H), 3.35 (s, 3H). **^{13}C NMR** (75.4 MHz, $CDCl_3$) *δ* (ppm): 144.9 (C), 140.2 (C), 138.0 (C), 135.3 (C), 130.5 (2 × CH), 130.3 (CH), 129.6 (2 × CH), 129.4 (2 × CH), 127.8 (2 × CH), 127.2 (CH), 127.1 (CH), 126.6 (CH), 120.4 (C), 119.2 (C), 116.8 (C), 98.5 (CH), 36.8 (CH_3). **HRMS** (ESI-TOF): calculated for $C_{21}H_{18}NO^+$ $[M+H]^+$ 300.1383; found 300.1385.

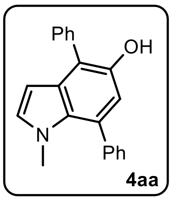
4aa

1-Methyl-4,7-diphenyl-1*H*-indol-5-ol (4aa): Following the general procedure E using IPrAuNTf_2 (**[Au]-I**) with 1-(1-methyl-1*H*-pyrrol-2-yl)-2,4-diphenylbut-3-yne-1,2-diol (**1aa**), the crude product was obtained as a 1/1.6 (**3aa/4aa**) mixture of regioisomers, and was purified by column chromatography (hexane/EtOAc, 5/1), affording pure **4aa** as a pale pink solid (26 mg, 43%); R_f = 0.27 (hexane/EtOAc, 5/1). NMR data matches with those found in the literature.[3]

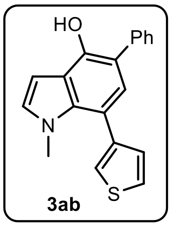
3ab

1-Methyl-5-phenyl-7-(thiophen-3-yl)-1*H*-indol-4-ol (3ab): Following the general procedure E using SPhosAuMeCNSbF_6 (**[Au]-II**) with 1-(1-methyl-1*H*-pyrrol-2-yl)-2-phenyl-4-(thiophen-3-yl)but-3-yne-1,2-diol (**1ab**), the crude product was obtained as a 3/1 (**3ab/4ab**) mixture of regioisomers, and was purified by column chromatography (hexane/EtOAc, 10/1), affording pure **3ab** as a brown solid (33 mg, 54%); mp = 130–132 °C; R_f = 0.25 (hexane/EtOAc, 10/1). **^{1}H NMR** (300 MHz, $CDCl_3$) *δ* (ppm): 7.62–7.45 (m, 4H), 7.42–7.28 (m, 3H), 7.22 (d, *J* = 4.8 Hz, 1H), 7.01 (s, 1H), 6.96 (d, *J* = 3.2 Hz, 1H), 6.69 (d, *J* = 3.2 Hz, 1H), 5.59 (s, 1H), 3.42 (s, 3H). **^{13}C NMR** (75.4 MHz, $CDCl_3$) *δ* (ppm): δ 145.1 (C), 140.1 (C), 137.9 (C), 135.6 (C), 130.7 (CH), 130.2 (CH), 129.6 (2 × CH), 129.4 (2 × CH), 127.2 (CH), 126.7 (CH), 124.6 (CH), 123.5 (CH), 119.2 (C), 116.8 (C), 114.7 (C), 98.5 (CH), 36.1 (CH_3). **HRMS** (ESI-TOF): calculated for $C_{19}H_{16}NOS^+$ $[M+H]^+$ 306.0947; found 306.0947.

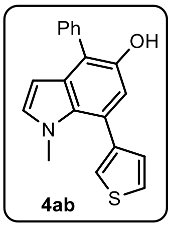
4ab

1-Methyl-4-phenyl-7-(thiophen-3-yl)-1*H*-indol-5-ol (4ab): Following the general procedure E using IPrAuNTf_2 (**[Au]-I**) with 1-(1-methyl-1*H*-pyrrol-2-yl)-2-phenyl-4-(thiophen-3-yl)but-3-yne-1,2-diol (**1ab**), the crude product was obtained as a 1/1 (**3ab/4ab**) mixture of regioisomers, and was purified by column chromatography (hexane/EtOAc, 10/1), affording pure **4ab** as a brown solid (18 mg, 30%); mp = 128–130 °C; R_f = 0.27 (hexane/EtOAc, 10/1). **^{1}H NMR** (300 MHz, $CDCl_3$) *δ* (ppm): 7.67–7.52 (m, 4H), 7.48–7.38 (m, 2H), 7.33 (dd, *J* = 3.0, 1.2 Hz, 1H), 7.24 (dd, *J* = 4.8, 1.2 Hz, 1H), 6.94 (d, *J* = 3.2 Hz, 1H), 6.84 (s, 1H), 6.28 (d, *J* = 3.2 Hz, 1H), 4.96 (s, 1H), 3.37 (s, 3H). **^{13}C NMR** (125.7 MHz, $CDCl_3$) *δ* (ppm):

[3] Chen, Z.; Huang, W.; Yi, L.; Dong, X.; Sheng, K.; Li, M. ;Bai, R.; Sidorenko, A.Y.; Huang, J.; Gu, Y. *Green Chem.* **2022**, *24*, 2919–2926.

145.2 (C), 139.8 (C), 135.6 (C), 131.9 (CH), 130.3 (CH), 130.2 (2 × CH), 129.9 (C), 129.7 (C), 129.4 (2 × CH), 127.9 (CH), 124.8 (CH), 123.7 (CH), 122.1 (C), 117.6 (C), 114.3 (CH), 100.1 (CH), 36.3 (CH_3) .**HRMS** (ESI-TOF): calculated for $C_{19}H_{16}NOS^+$ $[M+H]^+$ 306.0947; found 306.0947.

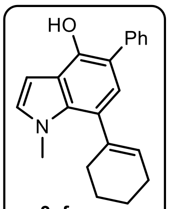

3af

7-(Cyclohex-1-en-1-yl)-1-methyl-5-phenyl-1*H*-indol-4-ol (3af): Following the general procedure E using $SPhosAuMeCNSbF_6$ (**[Au]-II**) with 4-(cyclohex-1-en-1-yl)-1-(1-methyl-1*H*-pyrrol-2-yl)-2-phenylbut-3-yne-1,2-diol (**1af**), the crude product was obtained as a 3.7/1 (**3af/4af**) mixture of regioisomers, and was purified by column chromatography (hexane/EtOAc, 10/1), affording pure **3af** as a yellow oil (33 mg, 55%); R_f = 0.25 (hexane/EtOAc, 10/1). **^{1}H NMR** (300 MHz, $CDCl_3$) δ (ppm): 7.58–7.44 (m, 4H), 7.40–7.32 (m, 1H), 6.94 (d, J = 3.2 Hz, 1H), 6.81 (s, 1H), 6.62 (d, J = 3.2 Hz, 1H), 5.81–5.73 (m, 1H), 5.44 (s, 1H), 3.85 (s, 3H) 2.46–2.31 (m, 2H), 2.28–2.16 (m, 2H), 1.96–1.65 (m, 4H). **^{13}C NMR** (75.4 MHz, $CDCl_3$) δ (ppm): 144.1 (C), 138.3 (C), 136.7 (C), 135.0 (C), 129.8 (CH), 129.6 (2 × CH), 129.3 (2 × CH), 127.5 (CH), 127.1 (CH), 124.9 (CH), 122.9 (C), 119.1 (C), 116.7 (C), 98.4 (CH), 35.6 (CH_3), 32.8 (CH_2), 25.6 (CH_2), 23.2 (CH_2), 22.3 (CH_2). **HRMS** (ESI-TOF): calculated for $C_{21}H_{22}NO^+$ $[M+H]^+$ 304.1696; found 304.1694.

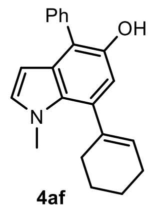

4af

7-(Cyclohex-1-en-1-yl)-1-methyl-4-phenyl-1*H*-indol-5-ol (4af): Following the general procedure E using $IPrAuNTf_2$ (**[Au]-I**) with 4-(cyclohex-1-en-1-yl)-1-(1-methyl-1*H*-pyrrol-2-yl)-2-phenylbut-3-yne-1,2-diol (**1af**), the crude product was obtained as a 1/1 (**3af/4af**) mixture of regioisomers, and was purified by column chromatography (hexane/EtOAc, 10/1), affording pure **4af** as a yellow oil (22 mg, 37%); R_f = 0.27 (hexane/EtOAc, 10/1). **^{1}H NMR** (300 MHz, $CDCl_3$) δ (ppm): 7.61–7.49 (m, 4H), 7.46–7.37 (m, 1H), 6.93 (d, J = 3.2 Hz, 1H), 6.67 (s, 1H), 6.23 (d, J = 3.2 Hz, 1H), 5.80 (dt, J = 3.7, 1.9 Hz, 1H), 4.91 (s, 1H), 3.83 (s, 3H), 2.53–2.36 (m, 2H), 2.31–2.19 (m, 2H), 1.94–1.71 (m, 4H). **^{13}C NMR** (75.4 MHz, $CDCl_3$) δ (ppm): 145.6 (C), 136.5 (C), 135.9 (C), 131.4 (CH), 130.2 (2 × CH), 130.2 (C), 129.7 (C), 129.3 (2 × CH), 129.1 (C), 127.6 (CH), 127.5 (CH), 116.4 (C), 112.5 (CH), 99.9 (CH), 35.9 (CH_3), 32.3 (CH_2), 25.5 (CH_2), 23.1 (CH_2), 22.2 (CH_2). **HRMS** (ESI-TOF): calculated for $C_{21}H_{22}NO^+$ $[M+H]^+$ 304.1696; found 304.1695.

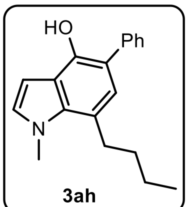

3ah

7-Butyl-1-methyl-5-phenyl-1*H*-indol-4-ol (3ah): Following the general procedure E using $SPhosAuMeCNSbF_6$ (**[Au]-II**) with 1-(1-methyl-1*H*-pyrrol-2-yl)-2-phenyloct-3-yne-1,2-diol (**1ah**), the crude product was obtained as a 2/1 (**3ah**/**4ah**) mixture of regioisomers (**3ah**+**4ah**/**6ah** ratio = 1.5/1), and was purified by column chromatography (hexane/EtOAc, 10/1), affording pure **3ah** as a yellow oil (15 mg, 27%); R_f = 0.24 (hexane/EtOAc, 10/1). **^{1}H NMR** (500 MHz, $CDCl_3$) δ (ppm): 7.57–7.45 (m, 4H), 7.40–7.33 (m, 1H), 6.92 (d, J = 3.2 Hz, 1H), 6.85 (s, 1H), 6.60 (d, J = 3.2 Hz, 1H), 5.39 (s, 1H), 4.02 (s, 3H), 3.06–2.98 (m, 2H), 1.76–1.66 (m, 2H), 1.48 (h, J = 7.3 Hz, 2H), 0.98 (t, J = 7.3 Hz, 3H). **^{13}C NMR** (75.4 MHz, $CDCl_3$) δ (ppm): 143.7 (C), 138.5 (C), 135.9 (C), 130.1 (CH), 129.6 (2 × CH), 129.3 (2 × CH), 127.0 (CH), 125.6 (CH), 119.8 (C), 119.5 (C), 116.9 (C), 98.4 (CH), 36.6 (CH_3), 35.2 (CH_2), 31.9 (CH_2), 22.7 (CH_2), 14.2 (CH_3). **HRMS** (ESI-TOF): calculated for $C_{19}H_{22}NO^+$ $[M+H]^+$ 280.1696; found 280.1697.

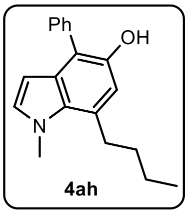

7-Butyl-1-methyl-4-phenyl-1*H*-indol-5-ol (4ah): Following the general procedure E using IPrAuNTf$_2$ (**[Au]-I**) with 1-(1-methyl-1*H*-pyrrol-2-yl)-2-phenyloct-3-yne-1,2-diol (**1ah**), the crude product was obtained as a 1/1.3 (**3ah**/**4ah**) mixture of regioisomers (**3ah**+**4ah**/**6ah** ratio = 4/1), and was purified by column chromatography (hexane/EtOAc, 10/1), affording pure **4ah** as a yellow oil (18 mg, 33%); R$_f$ = 0.26 (hexane/EtOAc, 10/1). **^{1}H NMR** (500 MHz, CDCl$_3$) *δ* (ppm): 7.59–7.52 (m, 4H), 7.46–7.40 (m, 1H), 6.92 (d, *J* = 3.2 Hz, 1H), 6.75 (s, 1H), 6.21 (d, *J* = 3.2 Hz, 1H), 4.91 (s, 1H), 4.03 (s, 3H), 3.12–3.02 (m, 2H), 1.83–1.73 (m, 2H), 1.56–1.49 (m, 2H), 1.02 (t, *J* = 7.4 Hz, 3H). **^{13}C NMR** (125.7 MHz, CDCl$_3$) *δ* (ppm): 145.5 (C), 135.9 (C), 131.6 (CH), 130.3 (2 × CH), 130.11 (C), 130.07 (C), 129.4 (2 × CH), 127.6 (CH), 127.5 (C), 115.8 (C), 113.3 (CH), 99.9 (CH), 37.0 (CH$_3$), 34.7 (CH$_2$), 32.3 (CH$_2$), 22.8 (CH$_2$), 14.2 (CH$_3$). **HRMS** (ESI-TOF): calculated for $C_{19}H_{22}NO^+$ $[M+H]^+$ 280.1696; found 280.1699.

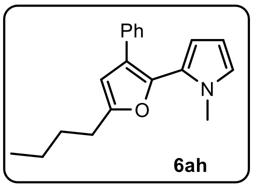

2-(5-Butyl-3-phenylfuran-2-yl)-1-methyl-1*H*-pyrrole (6ah): Following the general procedure E using SPhosAuMeCNSbF$_6$ (**[Au]-II**) with 1-(1-methyl-1*H*-pyrrol-2-yl)-2-phenyloct-3-yne-1,2-diol (**1ah**), the crude product was obtained as a 2/1 (**3ah**/**4ah**) mixture of regioisomers (**3ah**+**4ah**/**6ah** ratio = 1.5/1). The furan derivative was purified by column chromatography (hexane/EtOAc, 20/1), affording pure **6ah** as a yellow oil (13 mg, 23%); R$_f$ = 0.35 (hexane/EtOAc, 20/1). **^{1}H NMR** (300 MHz, CDCl$_3$) *δ* (ppm): 7.39–7.18 (m, 5H), 6.75–6.68 (m, 1H), 6.36–6.28 (m, 2H), 6.25–6.16 (m, 1H), 3.43 (s, 3H), 2.71 (t, *J* = 7.5 Hz, 2H), 1.72 (quin, *J* = 7.5 Hz, 2H), 1.54–1.38 (m, 3H), 0.98 (t, *J* = 7.3 Hz, 3H). **^{13}C NMR** (75.4 MHz, CDCl$_3$) *δ* (ppm): 156.4 (C), 140.4 (C), 134.0 (C), 128.6 (2 × CH), 127.2 (2 × CH), 126.7 (CH), 124.4 (C), 124.3 (C), 123.5 (CH), 111.3 (CH), 108.1 (CH), 106.6 (CH), 34.8 (CH$_3$), 30.2 (CH$_2$), 28.0 (CH$_2$), 22.4 (CH$_2$), 14.0 (CH$_3$). **HRMS** (ESI-TOF): calculated for $C_{19}H_{22}NO^+$ $[M+H]^+$ 280.1696; found 280.1698.

<u>Gold-catalyzed cyclization of 2aa</u>

Optimization studies

As pointed out in the manuscript, a comprehensive investigation into the reaction parameters governing the gold-catalyzed cyclization of **2aa**, **2´aa**, **2´´aa** was performed. The following *Table S2* includes the detailed optimization process carried out with selected alkynol **2aa**, considering the influence of different parameters in the selectivity of the reaction for the synthesis of the corresponding 4-silyloxyindole **7aa**.

Initially, we investigated the influence of the trialkylsilyl group by employing **2aa**, **2'aa**, and **2''aa** as starting substrates with IPrAuNTf$_2$ as catalyst (entries 1–3). The cyclization led to the formation of a mixture of the corresponding 4-silyloxyindoles **7** and 7-silyloxyindoles **8**, with the best selectivity and yield (8/1 regioisomeric ratio, 87% yield) achieved using the bulkier TBDMS-protected diol **2aa** (entry 3). Once we elucidated this effect of the silyl protecting group, we opted to utilize various commercially available gold(I) complexes.

Table S2: Evaluation of the gold-catalyzed benzannulation of ***2aa***

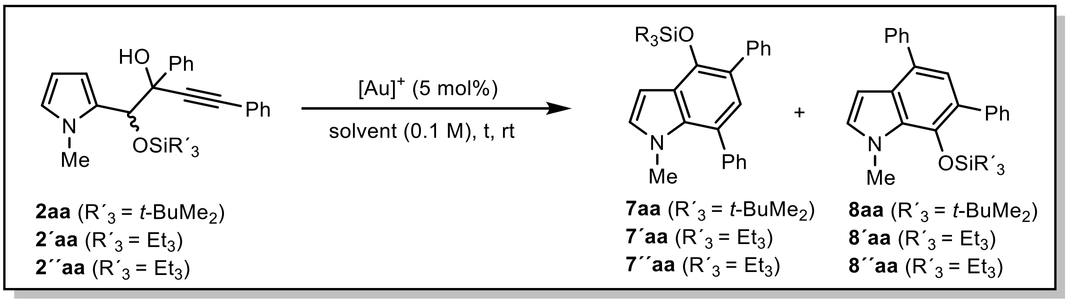

entry	2	[Au]$^+$	solvent	t(h)	r.r. 7aa/8aa[a]	yield (%)[b]
1	**2´´aa**	$IPrAuNTf_2$	DCM	2	3/1[c]	47[d]
2	**2´aa**	$IPrAuNTf_2$	DCM	1.5	6/1[e]	80[f]
3	**2aa**	$IPrAuNTf_2$	DCM	4	8/1	87
4[g]	**2aa**	$(ArO)_3PAuCl/AgOTf$	DCM	2	4.6/1	40
5	**2aa**	PPh_3AuNTf_2	DCM	16	3.3/1	63
6	**2aa**	$XPhosAuNTf_2$	DCM	16	6/1	47
7	**2aa**	$SPhosAuNTf_2$	DCM	16	3.3/1	70
8	**2aa**	$BrettPhosAuNTf_2$	DCM	16	9/1	66
9	**2aa**	$IPrAuCl/AgNTf_2$	DCM	4	8/1	73
10	**2aa**	IPrAuCl/AgOTf	DCM	2	5/1	55
11	**2aa**	$IPrAuCl/AgBF_4$	DCM	5	6/1	88
12	**2aa**	$IPrAuCl/AgSbF_6$	DCM	4	9/1	72
13	**2aa**	IPrAuCl/HFIP	HFIP	16	>20/1	40
14	**2aa**	AgOTf	DCM	–	–	–[h]
15	**2aa**	$NaAuCl_4·2H_2O$	DCM	–	–	–[h]
16[i]	**2aa**	**$IPrAuNTf_2$**	**DCM**	**6**	**>20/1**	**89**
17[j]	**2aa**	$IPrAuNTf_2$	DCM	1	9/1	95
18	**2aa**	$IPrAuNTf_2$ / PTSA (10 mol%)	DCM	2	5/1	81

[a]Regioisomeric ratio determined by ^1H NMR analysis of the crude. [b]Yield of **7aa** by ^1H NMR with CH_2Br_2 as internal standard. [c]**7´´aa/8´´aa** ratio. [d]Yield of **7´´aa**. [e]**7´aa/8´aa** ratio. [f]Yield of**7´aa**. [g]Ar = 2,4-*t*-$Bu_2C_6H_3$. [h]Only decomposition products were observed in the reaction crude. [i]Carried out at 0.01 M. [j]Carried out employing 10 mol% of catalyst.

When using a phosphite-gold(I) chloride in combination with a silver salt, as the catalytic system, the cyclization occurred in 2 h, although the regioselectivity decreased and the yield was moderate (entry 4 vs 3). Subsequently, we studied the performance of different gold(I) complexes bearing a variety of phosphine ligands. However, in none of these cases, the regioselectivity towards **7aa** was improved compared to the use of $IPrAuNTf_2$ (entries 5–7 vs 3). On the other hand, although the regioselectivity of the reaction process resulted slightly improved to 9/1 when employing $BrettPhosAuNTf_2$ as catalyst (entry 8), the yield was significantly lower. Additionally, we decided to study the counteranion effect using different silver salts. Firstly, no effect was observed employing $AgNTf_2$ as counteranion (entry 9). Alternatively, other counteranions such as AgOTf or $AgBF_4$ were proved. Nevertheless, the regioisomeric ratio was not improved (entries 10–11 vs 3). Lastly, although the regioisomeric ratio was

moderately improved when AgSbF6 was proved as counteranion, the yield was lower (entry 12). Surprisingly, when we used HFIP as a solvent with IPrAuCl as catalyst,[2] the reaction was completed after 16 h with high selectivity towards 4-silyloxyindole **7aa**, albeit with a low yield. In addition, the reaction crude showed appreciable unidentified decomposition products (entry 13). Finally, neither a silver salt such as AgOTf nor an Au(III) complex was capable of catalyzing the benzannulation of **2aa,** resulting in decomposition (entries 14 and 15). The optimal reaction conditions were achieved by increasing the dilution to 0.01 M, resulting in almost complete selectivity towards 4-silyloxyindole **7aa** (entry 16). With the aim of achieving an explanation of that fact, we tested to add a double amount of the optimal catalyst (10 mol%). The results showed that only the reaction time was affected since the reaction was faster without modifying the regioisomeric ratio significantly (entry 17 vs 3). Furthermore, we tested the effect of adding a catalytic amount of a Brønsted acid such as PTSA, into the reaction mixture along with IPrAuNTf$_2$. Under these conditions, the regioisomeric ratio slightly decreased to 5/1 (entry 18 vs 3), which suggests that traces of acid may be formed during the process (particularly if the protodeauration step is slow enough), facilitating alternative reaction pathways.

Synthesis and characterization data of 4-silyloxyindoles 7

General procedure F for the gold-catalyzed cyclization of 2

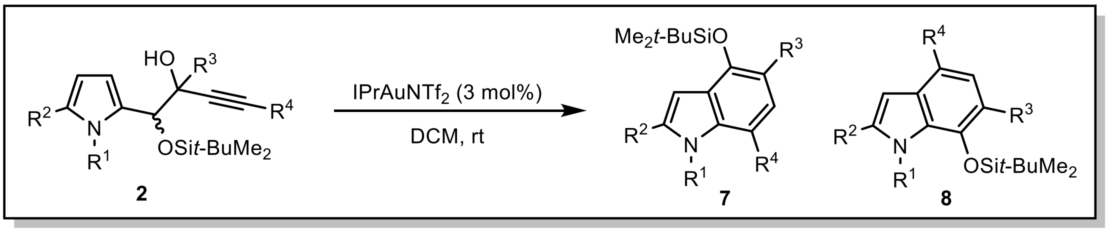

IPrAuNTf$_2$ (8 mg, 0.03 mmol, 0.03 equiv) was added to a solution of the corresponding alkynol **2** (0.3 mmol, 1 equiv) in anhydrous DCM (30 mL, 0.01 M) with continuous stirring at rt. The reaction mixture was stirred until the starting material completely disappeared, as determined by TLC. With some substrates, complete conversion required the addition of a second loading of IPrAuNTf$_2$ (8 mg, 0.03 mmol, 0.03 equiv) after 8 h of reaction. Once the reaction was completed, DCM was evaporated under reduced pressure and the residue was purified directly by flash column chromatography using mixtures of hexane and EtOAc as eluents to obtain the 4-silyloxyindoles **7**, which in some cases were isolated along with minor amounts of the corresponding regioisomeric 7-silyloxyindoles **8**. For **2ga** and **2gb** the corresponding 4-silyloxyindole derivatives were desilylated in situ, leading to the isolation of 4-hydroxyindoles **3ga** and **3gb**.

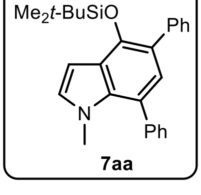

4-((*Tert*-butyldimethylsilyl)oxy)-1-methyl-5,7-diphenyl-1*H*-indole (7aa): Following the general procedure F with 1-((*tert*-butyldimethylsilyl)oxy)-1-(1-methyl-1*H*-pyrrol-2-yl)-2,4-diphenylbut-3-yn-2-ol (**2aa)** (129 mg, 0.3 mmol), the crude product was obtained as a >20/1 (**7aa**/**8aa**) mixture of regioisomers, and was purified by column chromatography (hexane/EtOAc, 50/1), affording pure **7aa** as a

colourless solid (104 mg, 84%); mp = 121–123 °C; R_f = 0.24 (hexane/EtOAc, 50/1). **^{1}H NMR** (300 MHz, $CDCl_3$) δ (ppm): 7.66–7.59 (m, 2H), 7.55–7.35 (m, 7H), 7.31–7.26 (m, 1H), 7.05 (s, 1H), 6.93 (d, J = 3.2 Hz, 1H), 6.64 (d, J = 3.2 Hz, 1H), 3.35 (s, 3H), 1.01 (s, 9H), –0.21 (s, 6H). **^{13}C NMR** (75.4 MHz, $CDCl_3$) δ (ppm): 144.8 (C), 140.39 (C), 140.37 (C), 135.1 (C), 130.52 (2 × CH), 130.48 (2 × CH), 129.9 (CH), 128.0 (2 × CH), 127.7 (2 × CH), 127.6 (CH), 127.0 (CH), 126.1 (CH), 124.1 (C), 122.9 (C), 121.4 (C), 100.1 (CH), 36.8 (CH_3), 26.0 (3 × CH_3), 18.5 (C), -4.2 (2 × CH_3). **HRMS** (ESI-TOF): calculated for $C_{27}H_{32}NOSi^+$ $[M+H]^+$ 414.2248; found 414.2252.

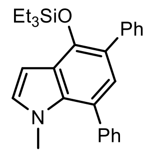

7´aa

1-Methyl-5,7-diphenyl-4-((triethylsilyl)oxy)-1*H*-indole (7´aa): $IPrAuNTf_2$ (4 mg, 5 mol%) was added to a solution of 1-(1-methyl-1*H*-pyrrol-2-yl)-2,4-diphenyl-1-((triethylsilyl)oxy)but-3-yn-2-ol (**2´aa**, 41 mg, 0.1 mmol) in anhydrous DCM (1 mL). The solution was stirred for 1.5 h. After this time, the solvent was evaporated under reduced pressure. The crude product was obtained, and isolated after column chromatography (hexane/EtOAc, 40/1), as a 6/1 (**7´aa/8´aa**) mixture of regioisomers; colourless solid (33 mg, 80%); mp = 118–120 °C; R_f = 0.29 (hexane/EtOAc, 40/1). Data for **7´aa**: **^{1}H NMR** (300 MHz, $CDCl_3$) δ (ppm): 7.74–7.58 (m, 2H), 7.57–7.25 (m, 8H), 7.05 (s, 1H), 6.94 (d, J = 3.2 Hz, 1H), 6.66 (d, J = 3.2 Hz, 1H), 3.36 (s, 3H), 0.85 (t, J = 8.0 Hz, 9H), 0.53 (t, J = 8.0 Hz, 6H). **^{13}C NMR** (75.4 MHz, $CDCl_3$) δ (ppm): 145.1 (C), 140.3 (C), 140.2 (C), 134.9 (C), 130.4 (2 × CH), 130.3 (2 × CH), 129.8 (CH), 127.9 (2 × CH), 127.6 (2 × CH), 127.3 (CH), 126.9 (CH), 126.0 (CH), 123.9 (C), 122.7 (C), 121.2 (C), 99.5 (CH), 36.7 (CH_3), 6.7 (3 × CH_3), 5.3 (3 × CH_2). **HRMS** (ESI-TOF): calculated for $C_{27}H_{32}NOSi^+$ $[M+H]^+$ 414.2248; found 414.2254.

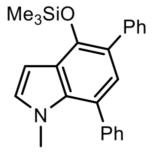

7´´aa

1-Methyl-5,7-diphenyl-4-((trimethylsilyl)oxy)-1*H*-indole (7´´aa): $IPrAuNTf_2$ (4 mg, 5 mol%) was added to a solution of 1-(1-methyl-1*H*-pyrrol-2-yl)-2,4-diphenyl-1-((trimethylsilyl)oxy)but-3-yn-2-ol (**2´´aa**, 39 mg, 0.1 mmol) in anhydrous DCM (1 mL). The solution was stirred for 2 h. After this time, the solvent was evaporated under reduced pressure. The crude product was obtained, and isolated after column chromatography (hexane/EtOAc, 50/1), as a 3/1 (**7´´aa/8´´aa**) mixture of regioisomers; colourless solid (17 mg, 47%); mp = 112–114 °C; R_f = 0.27 (hexane/EtOAc, 50/1). Data for both regioisomers: **^{1}H NMR** (300 MHz, $CDCl_3$) δ (ppm): 7.80–7.73 (m, 2H, min), 7.73–7.64 (m, 2H, maj), 7.60–7.28 (m, 16H, both), 7.19 (s, 1H, min), 7.11 (s, 1H, maj), 7.05 (d, J = 3.2 Hz, 1H, min), 6.96 (d, J = 3.2 Hz, 1H, maj), 6.69 (d, J = 3.2 Hz, 1H, min), 6.65 (d, J = 3.2 Hz, 1H, maj), 4.14 (s, 3H, min), 3.39 (s, 3H, maj), 0.05 (s, 9H, maj), –0.05 (s, 9H, min). **^{13}C NMR** (75.4 MHz, $CDCl_3$) δ (ppm): 144.8 (C), 141.1 (C), 140.7 (C), 140.4 (C), 140.2 (C), 138.9 (C), 135.1 (C), 131.4 (CH), 130.7 (2 × CH) 130.5 (2 × CH), 130.3 (2 × CH), 130.0 (2 × CH), 129.2 (C), 128.8 (CH), 128.5 (CH), 128.3 (CH), 128.0 (2 × CH), 127.7 (2 × CH), 127.3 (2 × CH), 127.0 (2 × CH), 126.8 (C), 126.62 (CH), 126.58 (CH), 126.1 (CH), 124.1 (C), 123.1 (CH), 122.8 (C), 121.5 (C), 101.1 (CH), 99.7 (CH), 36.8 (CH_3), 36.4 (CH_3), 0.61 (3 × CH_3), 0.4 (3 × CH_3). Two C peaks from minor regioisomer were missing. **HRMS** (ESI-TOF): calculated for $C_{24}H_{26}NOSi^+$ $[M+H]^+$ 372.1778; found 372.1789.

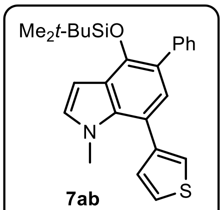

7ab

4-((*Tert*-butyldimethylsilyl)oxy)-1-methyl-5-phenyl-7-(thiophen-3-yl)-1*H*-indole (7ab): Following the general procedure F with 1-((*tert*-butyldimethylsilyl)oxy)-1-(1-methyl-1*H*-pyrrol-2-yl)-2-phenyl-4-(thiophen-3-yl)but-3-yn-2-ol (**2ab**) (131 mg, 0.3 mmol), the crude product was obtained as a >20/1 (**7ab**/**8ab**) mixture of regioisomers, and was purified by column chromatography (hexane/EtOAc, 30/1), affording pure **7ab** as a brown solid (105 mg, 83%); mp = 102–104 °C; R_f = 0.38 (hexane/EtOAc, 30/1). **^{1}H NMR** (300 MHz, $CDCl_3$) δ (ppm): 7.68–7.61 (m, 2H), 7.47–7.36 (m, 3H), 7.35–7.24 (m, 3H), 7.12 (s, 1H), 6.95 (d, J = 3.2 Hz, 1H), 6.66 (d, J = 3.2 Hz, 1H), 3.43 (s, 3H), 1.03 (s, 9H), –0.18 (s, 6H). **^{13}C NMR** (75.4 MHz, $CDCl_3$) δ (ppm): 145.0 (C), 140.31 (C), 140.28 (C), 135.5 (C), 130.7 (CH), 130.5 (2 × CH), 129.8 (CH), 128.0 (2 × CH), 127.7 (CH), 126.2 (CH), 124.5 (CH), 124.0 (C), 123.4 (CH), 122.9 (C), 115.8 (C), 100.1 (CH), 36.1 (CH_3), 26.0 (3 × CH_3), 18.5 (C), –4.2 (2 × CH_3). **HRMS** (ESI-TOF): calculated for $C_{25}H_{30}NOSSi^+$ $[M+H]^+$ 420.1812; found 420.1813.

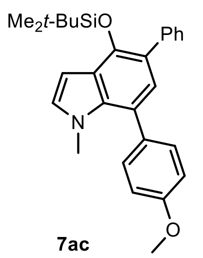

7ac

4-((*Tert*-butyldimethylsilyl)oxy)-7-(4-methoxyphenyl)-1-methyl-5-phenyl-1H-indole (7ac): Following the general procedure F with 1-((*tert*-butyldimethylsilyl)oxy)-4-(4-methoxyphenyl)-1-(1-methyl-1*H*-pyrrol-2-yl)-2-phenylbut-3-yn-2-ol (**2ac**) (141 mg, 0.3 mmol), the crude product was obtained as a >20/1 (**7ab**/**8ab**) mixture of regioisomers, and was purified by column chromatography (hexane/EtOAc, 40/1), affording pure **7ac** as a colourless solid (106 mg, 80%); mp = 105–107 °C; R_f = 0.23 (hexane/EtOAc, 40/1). **^{1}H NMR** (300 MHz, $CDCl_3$) δ (ppm): 7.67–7.60 (m, 2H), 7.49–7.36 (m, 4H), 7.33–7.24 (m, 1H), 7.04 (s, 1H), 7.01–6.96 (m, 2H), 6.92 (d, J = 3.2 Hz, 1H), 6.64 (d, J = 3.2 Hz, 1H), 3.90 (s, 3H), 3.38 (s, 3H), 1.02 (s, 9H), –0.20 (s, 6H). **^{13}C NMR** (75.4 MHz, $CDCl_3$) δ (ppm): 158.8 (C), 144.6 (C), 140.4 (C), 135.4 (C), 132.7 (C), 131.5 (2 × CH), 130.5 (2 × CH), 129.8 (CH), 128.0 (2 × CH), 127.7 (CH), 126.1 (CH), 124.0 (C), 122.9 (C), 121.0 (C), 113.1 (2 × CH), 100.0 (CH), 55.4 (CH_3), 36.7 (CH_3), 26.0 (3 × CH_3), 18.5 (C), -4.19 (CH_3), -4.20 (CH_3). **HRMS** (ESI-TOF): calculated for $C_{28}H_{34}NO_2Si^+$ $[M+H]^+$ 444.2353; found 444.2352.

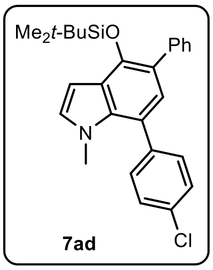

7ad

4-((*Tert*-butyldimethylsilyl)oxy)-7-(4-chlorophenyl)-1-methyl-5-phenyl-1*H*-indole (7ad): Following the general procedure F with 1-((*tert*-butyldimethylsilyl)oxy)-4-(4-chlorophenyl)-1-(1-methyl-1*H*-pyrrol-2-yl)-2-phenylbut-3-yn-2-ol (**2ad**) (140 mg, 0.3 mmol), the crude product was obtained as a 16/1 (**7ad**/**8ad**) mixture of regioisomers, and was purified by column chromatography (hexane/EtOAc, 40/1), affording pure **7ad** as a colourless solid (105 mg, 78%); mp = 169–171 °C; R_f = 0.27 (hexane/EtOAc, 40/1). **^{1}H NMR** (300 MHz, $CDCl_3$) δ (ppm): 7.67–7.57 (m, 2H), 7.50–7.35 (m, 6H), 7.35–7.24 (m, 1H), 7.03 (s, 1H), 6.94 (d, J = 3.2 Hz, 1H), 6.66 (d, J = 3.2 Hz, 1H), 3.38 (s, 3H), 1.02 (s, 9H), –0.19 (s, 6H). **^{13}C NMR** (75.4 MHz, $CDCl_3$) δ (ppm): 145.1 (C), 140.2 (C), 138.9 (C), 135.0 (C), 133.1 (C), 131.7 (2 × CH), 130.5 (2 × CH), 130.0 (CH), 128.1 (2 × CH), 127.9 (2 × CH), 127.6 (CH), 126.2 (CH), 124.2 (C), 123.1 (C), 119.9 (C), 100.3 (CH), 36.9 (CH_3), 26.0 (3 × CH_3), 18.5 (C), –4.2 (2 × CH_3). **HRMS** (ESI-TOF): calculated for $C_{27}H_{31}ClNOSi^+$ $[M+H]^+$ 448.1858; found 448.1862.

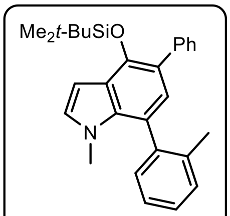

7ae

4-((*Tert*-butyldimethylsilyl)oxy)-1-methyl-5-phenyl-7-(*o*-tolyl)-1*H*-indole (7ae): Following the general procedure F with 1-((*tert*-butyldimethylsilyl)oxy)-1-(1-methyl-1*H*-pyrrol-2-yl)-2-phenyl-4-(o-tolyl)but-3-yn-2-ol (**2ae**) (134 mg, 0.3 mmol), the crude product was obtained, and isolated after column chromatography (hexane/EtOAc, 40/1), as a 10/1 (**7ae**/**8ae**) mixture of regioisomers; pale pink solid (115 mg, 90%); mp = 121–123 °C; R$_f$ = 0.29 (hexane/EtOAc, 40/1). Data for **7ae**: **^{1}H NMR** (300 MHz, $CDCl_3$) δ (ppm): 7.69–7.57 (m, 2H), 7.46–7.20 (m, 7H), 6.96 (s, 1H), 6.89 (d, *J* = 3.2 Hz, 1H), 6.62 (d, *J* = 3.2 Hz, 1H), 3.21 (s, 3H), 2.13 (s, 3H), 1.01 (s, 9H), –0.17 (s, 3H), –0.25 (s, 3H). **^{13}C NMR** (75.4 MHz, $CDCl_3$) δ (ppm): 144.5 (C), 140.5 (C), 139.8 (C), 137.7 (C), 135.2 (C), 131.0 (CH), 130.52 (2 × CH), 129.51 (CH), 129.48 (CH), 128.0 (2 × CH), 127.6 (CH), 126.7 (CH), 126.0 (CH), 125.2 (CH), 123.8 (C), 122.8 (C), 120.3 (C), 99.9 (CH), 35.3 (CH_3), 26.1 (3 × CH_3), 20.5 (CH_3), 18.5 (C), –4.1 (CH_3), –4.3 (CH_3). **HRMS** (ESI-TOF): calculated for $C_{28}H_{34}NOSi^+$ [M+H]$^+$ 428.2404; found 428.2416.

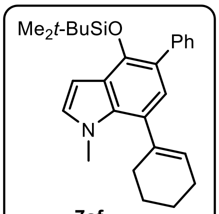

7af

4-((*Tert*-butyldimethylsilyl)oxy)-7-(cyclohex-1-en-1-yl)-1-methyl-5-phenyl-1*H*-indole (7af): Following the general procedure F with 1-((*tert*-butyldimethylsilyl)oxy)-4-(cyclohex-1-en-1-yl)-1-(1-methyl-1*H*-pyrrol-2-yl)-2-phenylbut-3-yn-2-ol (**2af**) (131 mg, 0.3 mmol), the crude product was obtained as a >20/1 (**7af**/**8af**) mixture of regioisomers, and was purified by column chromatography (hexane/EtOAc, 40/1), affording pure **7af** as a yellow solid (83 mg, 66%); mp = 132–134 °C; R$_f$ = 0.23 (hexane/EtOAc, 40/1). **^{1}H NMR** (300 MHz, $CDCl_3$) δ (ppm): 7.67–7.58 (m, 2H), 7.47–7.35 (m, 2H), 7.34–7.25 (m, 1H), 6.93 (d, *J* = 3.2 Hz, 1H), 6.91 (s, 1H), 6.58 (d, *J* = 3.2 Hz, 1H), 5.80 (tt, *J* = 3.8, 1.8 Hz, 1H), 3.87 (s, 3H), 2.48–2.35 (m, 2H), 2.33–2.18 (m, 2H), 1.95–1.69 (m, 4H), 1.00 (s, 9H), –0.23 (s, 6H). **^{13}C NMR** (75.4 MHz, $CDCl_3$) δ (ppm): 143.9 (C), 140.7 (C), 136.8 (C), 134.9 (C), 130.5 (2 × CH), 129.3 (CH), 127.9 (2 × CH), 127.3 (CH), 126.0 (CH), 125.8 (CH), 123.94 (C), 123.91 (C), 122.7 (C), 100.0 (CH), 35.6 (CH_3), 32.7 (CH_2), 26.0 (3 × CH_3), 25.6 (CH_2), 23.2 (CH_2), 22.3 (CH_2), 18.5 (C), –4.22 (2 × CH_3). **HRMS** (ESI-TOF): calculated for $C_{27}H_{36}NOSi^+$ [M+H]$^+$ 418.2561; found 418.2562.

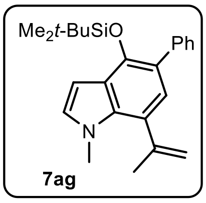

7ag

4-((*Tert*-butyldimethylsilyl)oxy)-1-methyl-5-phenyl-7-(prop-1-en-2-yl)-1*H*-indole (7ag): Following the general procedure F with 1-((*tert*-butyldimethylsilyl)oxy)-5-methyl-1-(1-methyl-1*H*-pyrrol-2-yl)-2-phenylhex-5-en-3-yn-2-ol (**2ag**) (119 mg, 0.3 mmol), the crude product was obtained as a >20/1 (**7ag**/**8ag**) mixture of regioisomers, and was purified by column chromatography (hexane/EtOAc, 50/1), affording pure **7ag** as a yellow oil (78 mg, 69%); R$_f$ = 0.18 (hexane/EtOAc, 50/1). **^{1}H NMR** (300 MHz, $CDCl_3$) δ (ppm): 7.68–7.53 (m, 2H), 7.45–7.35 (m, 2H), 7.34–7.24 (m, 1H), 6.97–6.91 (m, 2H), 6.58 (d, *J* = 3.2 Hz, 1H), 5.34 (s, 1H), 5.07 (s, 1H), 3.87 (s, 3H), 2.21 (s, 3H), 0.97 (s, 9H), –0.26 (s, 6H). **^{13}C NMR** (75.4 MHz, $CDCl_3$) δ (ppm): 144.3 (C), 144.1 (C), 140.5 (C), 134.4 (C), 130.5 (2 × CH), 129.5 (CH), 128.0 (2 × CH), 126.1 (CH), 125.4 (CH), 124.1 (C), 123.0 (C), 122.8 (C), 116.4 (CH_2), 100.1 (CH), 35.6 (CH_3), 26.8

(CH_3), 26.0 (3 × CH_3), 18.5 (C), –4.2 (2 × CH_3). **HRMS** (ESI-TOF): calculated for $C_{24}H_{32}NOSi^+$ $[M+H]^+$ 378.2248; found 378.2250.

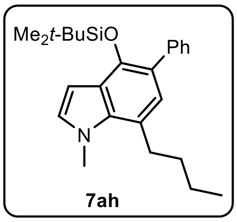

7-Butyl-4-((*tert*-butyldimethylsilyl)oxy)-1-methyl-5-phenyl-1*H*-indole (7ah): Following the general procedure F with 1-((*tert*-butyldimethylsilyl)oxy)-1-(1-methyl-1*H*-pyrrol-2-yl)-2-phenyloct-3-yn-2-ol (**2ah**) (123 mg, 0.3 mmol), the crude product was obtained as a 5/1 (**7ah/8ah**) mixture of regioisomers and was purified by column chromatography (hexane/EtOAc, 50/1), affording **7ah** as a 14/1 (**7ah/8ah**) mixture of regioisomers; colourless solid (96 mg, 81%); mp = 108–110 °C; R_f = 0.33 (hexane/EtOAc, 40/1). **^{1}H NMR** (300 MHz, $CDCl_3$) δ (ppm): 7.69–7.57 (m, 2H), 7.49–7.37 (m, 2H), 7.36–7.24 (m, 1H), 6.97 (s, 1H), 6.91 (d, J = 3.2 Hz, 1H), 6.57 (d, J = 3.2 Hz, 1H), 4.04 (s, 3H), 3.17–2.99 (m, 2H), 1.75 (quin, J = 8.0, 7.5 Hz, 2H), 1.60–1.45 (m, 2H), 1.04–0.97 (m, 12H), –0.24 (s, 6H). **^{13}C NMR** (75.4 MHz, $CDCl_3$) δ (ppm): 143.6 (C), 140.8 (C), 135.8 (C), 130.5 (2 × CH), 129.7 (CH), 128.0 (2 × CH), 126.6 (CH), 126.0 (CH), 124.5 (C), 122.9 (C), 120.5 (C), 100.0 (CH), 36.6 (CH_3), 35.1 (CH_2), 32.0 (CH_2), 26.0 (3 × CH_3), 22.8 (CH_2), 18.5 (C), 14.2 (CH_3), –4.3 (2 × CH_3). **HRMS** (ESI-TOF): calculated for $C_{25}H_{36}NOSi^+$ $[M+H]^+$ 394.2561; found 394.2568.

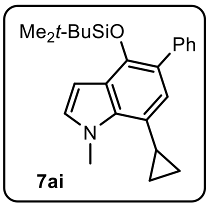

4-((*Tert*-butyldimethylsilyl)oxy)-7-cyclopropyl-1-methyl-5-phenyl-1*H*-indole (7ai): Following the general procedure F with 1-((*tert*-butyldimethylsilyl)oxy)-4-cyclopropyl-1-(1-methyl-1*H*-pyrrol-2-yl)-2-phenylbut-3-yn-2-ol (**2ai**) (119 mg, 0.3 mmol), the crude product was obtained as a 6/1 (**7ai/8ai**) mixture of regioisomers, and was purified by column chromatography (hexane/EtOAc, 50/1), affording pure **7ai** as a colourless solid (82 mg, 72%); mp = 106–108 °C; R_f = 0.28 (hexane/EtOAc, 50/1). **^{1}H NMR** (300 MHz, $CDCl_3$) δ (ppm): 7.63–7.53 (m, 2H), 7.46–7.35 (m, 2H), 7.34–7.23 (m, 1H), 6.95 (s, 1H), 6.93 (d, J = 3.2 Hz, 1H), 6.55 (d, J = 3.2 Hz, 1H), 4.24 (s, 3H), 2.50–2.32 (m, 1H), 1.05–0.90 (m, 13H), –0.25 (s, 6H). **^{13}C NMR** (75.4 MHz, $CDCl_3$) δ (ppm): 144.0 (C), 140.8 (C), 137.3 (C), 130.5 (2 × CH), 129.4 (CH), 128.0 (2 × CH), 126.0 (CH), 125.1 (CH), 124.0 (C), 122.6 (C), 120.4 (C), 99.9 (CH), 36.8 (CH_3), 26.0 (3 × CH_3), 18.5 (C), 13.2 (CH), 7.8 (2 × CH_2), -4.3 (CH_3).**HRMS** (ESI-TOF): calculated for $C_{24}H_{32}NOSi^+$ $[M+H]^+$ 378.2248; found 378.2254.

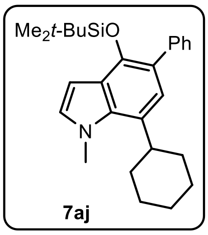

4-((*Tert*-butyldimethylsilyl)oxy)-7-cyclohexyl-1-methyl-5-phenyl-1*H*-indole (7aj): Following the general procedure F (with an additional catalyst loading after 8 h) with 1-((*tert*-butyldimethylsilyl)oxy)-4-cyclohexyl-1-(1-methyl-1*H*-pyrrol-2-yl)-2-phenylbut-3-yn-2-ol (**2aj**) (131 mg, 0.3 mmol), the crude product was obtained as a 5/1 (**7aj/8aj**) mixture of regioisomers, and was purified by column chromatography (hexane/EtOAc, 50/1), affording **7aj** as a 14/1 (**7aj**/**8aj**) mixture of regioisomers; colourless solid (93 mg, 74%); mp = 124–126 °C; R_f = 0.30 (hexane/EtOAc, 50/1). **^{1}H NMR** (300 MHz, $CDCl_3$) δ (ppm): 7.66–7.56 (m, 2H), 7.46–7.35 (m, 2H), 7.35–7.23 (m, 1H), 7.05 (s, 1H), 6.88 (d, J = 3.2 Hz, 1H), 6.55 (d, J = 3.2 Hz, 1H), 4.05 (s, 3H), 3.34 (tt, J = 11.5, 3.1 Hz, 1H), 2.19–1.77 (m, 5H), 1.73–1.28 (m, 5H), 0.99 (s, 1H), –0.26 (s, 6H). **^{13}C NMR** (75.4 MHz, $CDCl_3$) δ (ppm): 143.2 (C), 141.1

(C), 135.3 (C), 130.5 (2 × CH), 130.1 (CH), 128.0 (2 × CH), 126.4 (C), 126.0 (CH), 124.6 (C), 123.0 (C), 122.5 (CH), 99.9 (CH), 38.5 (CH), 37.3 (CH_3), 35.3 (2 × CH_2), 27.3 (2 × CH_2), 26.5 (CH_2), 26.1 (3 × CH_3), 18.5 (C), –4.2 (2 × CH_3). **HRMS** (ESI-TOF): calculated for $C_{27}H_{38}NOSi^+$ $[M+H]^+$ 420.2717; found 420.2726.

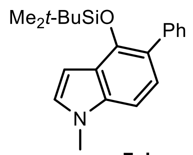

7ak

4-((*Tert*-butyldimethylsilyl)oxy)-1-methyl-5-phenyl-1*H*-indole (7ak): Following the general procedure F with 1-((*tert*-butyldimethylsilyl)oxy)-1-(1-methyl-1*H*-pyrrol-2-yl)-2-phenylbut-3-yn-2-ol (**2ak**) (107 mg, 0.3 mmol), the crude product was obtained as a 3/1 (**7ak**/**8ak**) mixture of regioisomers, and was purified by column chromatography (hexane/EtOAc, 50/1), affording pure **7ak** as a yellow oil (62 mg, 61%); R_f = 0.21 (hexane/EtOAc, 50/1). **^{1}H NMR** (300 MHz, $CDCl_3$) δ (ppm): 7.64–7.55 (m, 2H), 7.46–7.34 (m, 2H), 7.23 (d, J = 8.4 Hz, 1H), 7.07 (d, J = 8.4 Hz, 1H), 7.00 (d, J = 3.2 Hz, 1H), 6.57 (d, J = 3.2 Hz, 1H), 3.81 (s, 3H), 0.97 (s, 9H), –0.25 (s, 6H). **^{13}C NMR** (75.4 MHz, $CDCl_3$) δ (ppm): 145.5 (C), 140.8 (C), 138.2 (C), 130.6 (2 × CH), 128.0 (2 × CH), 127.8 (CH), 126.0 (CH), 125.2 (CH), 123.4 (C), 122.9 (C), 103.7 (CH), 99.9 (CH), 33.2 (CH_3), 26.0 (3 × CH_3), 18.5 (C), –4.3 (2 × CH_3). **HRMS** (ESI-TOF): calculated for $C_{21}H_{28}NOSi^+$ $[M+H]^+$ 338.1935; found 338.1936.

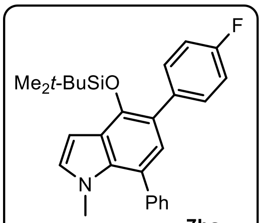

7ba

4-((*Tert*-butyldimethylsilyl)oxy)-5-(4-fluorophenyl)-1-methyl-7-phenyl-1*H*-indole (7ba): Following the general procedure F with 1-((*tert*-butyldimethylsilyl)oxy)-2-(4-fluorophenyl)-1-(1-methyl-1*H*-pyrrol-2-yl)-4-phenylbut-3-yn-2-ol (**2ba**) (135 mg, 0.3 mmol), the crude product was obtained as a >20/1 (**7ba**/**8ba**) mixture of regioisomers, and was purified by column chromatography (hexane/EtOAc, 40/1), affording pure **7ba** as a colourless solid (114 mg, 88%); mp = 139–141 °C; R_f = 0.30 (hexane/EtOAc, 40/1). **^{1}H NMR** (300 MHz, $CDCl_3$) δ (ppm): 7.63–7.34 (m, 7H), 7.13–7.01 (m, 2H), 6.98 (s, 1H), 6.91 (d, J = 3.2 Hz, 1H), 6.61 (d, J = 3.2 Hz, 1H), 3.33 (s, 3H), 0.99 (s, 9H), –0.20 (s, 6H). **^{13}C NMR** (75.4 MHz, $CDCl_3$) δ (ppm): 161.7 (d, $^1J_{C–F}$ = 244.4 Hz, C), 144.7 (C), 140.2 (CH), 136.4 (d, $^4J_{C–F}$ = 3.3 Hz, C), 135.2 (C), 131.9 (d, $^3J_{C–F}$ = 7.7 Hz, 2 × CH), 130.5 (2 × CH), 130.0 (CH), 127.8 (2 × CH), 127.4 (CH), 127.1 (CH), 124.1 (C), 121.8 (C), 121.5 (C), 114.8 (d, $^2J_{C–F}$ = 21.1 Hz, 2 × CH), 100.1 (CH), 36.8 (CH_3), 26.0 (3 × CH_3), 18.5 (C), –4.1 (2 × CH_3). **HRMS** (ESI-TOF): calculated for $C_{27}H_{31}FNOSi^+$ $[M+H]^+$ 432.2153; found 432.2155.

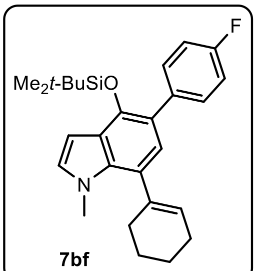

7bf

4-((*Tert*-butyldimethylsilyl)oxy)-7-(cyclohex-1-en-1-yl)-5-(4-fluorophenyl)-1-methyl-1*H*-indole (7bf): Following the general procedure F with 1-((*tert*-butyldimethylsilyl)oxy)-4-(cyclohex-1-en-1-yl)-2-(4-fluorophenyl)-1-(1-methyl-1*H*-pyrrol-2-yl)but-3-yn-2-ol (**2bf**) (136 mg, 0.3 mmol), the crude product was obtained as a >20/1 (**7bf/8bf**) mixture of regioisomers, and was purified by column chromatography (hexane/EtOAc, 50/1), affording pure **7bf** as a colourless solid (81 mg, 62%); mp = 133–135 °C; R_f = 0.42 (hexane/EtOAc, 40/1). **^{1}H NMR** (300 MHz, $CDCl_3$) δ (ppm): 7.61–7.47 (m, 2H), 7.13–7.00 (m, 2H), 6.89 (d, J = 3.2 Hz, 1H), 6.82 (s, 1H), 6.53 (d, J = 3.2 Hz, 1H), 5.82–5.72 (m, 1H), 3.83 (s, 3H), 2.45–2.32 (m, 2H), 2.27–2.12 (m, 2H), 1.92–1.67 (m, 4H), 0.96 (s, 9H), –0.25 (s, 6H). **^{13}C NMR** (75.4 MHz, $CDCl_3$) δ (ppm): 161.7 (d,

$^{1}J_{C-F}$ = 244.0 Hz, C), 143.9 (C), 136.8 (CH), 136.6 (d, $^{4}J_{C-F}$ = 3.3 Hz, C), 134.9 (C), 131.9 (d, $^{3}J_{C-F}$ = 7.7 Hz, 2 × CH), 129.5 (CH), 127.4 (CH), 125.6 (CH), 124.0 (C), 123.9 (C), 121.7 (C), 114.7 (d, $^{2}J_{C-F}$ = 21.0 Hz, 2 × CH), 100.0 (CH), 35.6 (CH_3), 32.7 (CH_2), 26.0 (3 × CH_3), 25.6 (CH_2), 23.2 (CH_2), 22.3 (CH_2), 18.5 (C), –4.13 (2 × CH_3). **HRMS** (ESI-TOF): calculated for $C_{27}H_{35}FNOSi^{+}$ $[M+H]^{+}$ 436.2466; found 436.2465.

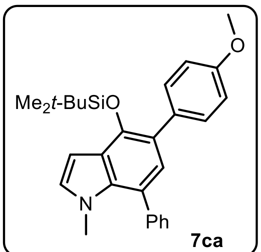

7ca

4-((*Tert*-butyldimethylsilyl)oxy)-5-(4-methoxyphenyl)-1-methyl-7-phenyl-1*H*-indole (7ca): Following the general procedure F with 1-((*tert*-butyldimethylsilyl)oxy)-2-(4-methoxyphenyl)-1-(1-methyl-1*H*-pyrrol-2-yl)-4-phenylbut-3-yn-2-ol (**2ca**) (139 mg, 0.3 mmol), the crude product was obtained as a >20/1 (**7ca**/**8ca**) mixture of regioisomers, and was purified by column chromatography (hexane/EtOAc, 40/1), affording pure **7ca** as a colourless solid (116 mg, 87%); mp = 126–128 °C; R_f = 0.38 (hexane/EtOAc, 40/1). **^{1}H NMR** (300 MHz, $CDCl_3$) δ (ppm): 7.63–7.31 (m, 7H), 7.01 (s, 1H), 6.98–6.86 (m, 3H), 6.61 (d, J = 3.2 Hz, 1H), 3.85 (s, 3H), 3.33 (s, 3H), 1.01 (s, 9H), –0.20 (s, 6H). **^{13}C NMR** (75.4 MHz, $CDCl_3$) δ (ppm): 158.2 (C), 144.7 (C), 140.4 (C), 134.93 (C), 132.9 (C), 131.4 (2 × CH), 130.5 (2 × CH), 129.8 (CH), 127.7 (2 × CH), 127.6 (CH), 127.0 (CH), 124.1 (C), 122.5 (C), 121.3 (C), 113.5 (2 × CH), 100.0 (CH), 55.5 (CH_3), 36.8 (CH_3), 26.1 (3 × CH_3), 18.5 (C), –4.1 (2 × CH_3). **HRMS** (ESI-TOF): calculated for $C_{28}H_{34}NO_2Si^{+}$ $[M+H]^{+}$ 444.2353; found 444.2358.

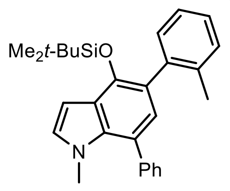

7da

4-((*Tert*-butyldimethylsilyl)oxy)-1-methyl-7-phenyl-5-(*o*-tolyl)-1*H*-indole (7da): Following the general procedure F (with an additional catalyst loading after 8 h) with 1-((*tert*-butyldimethylsilyl)oxy)-1-(1-methyl-1*H*-pyrrol-2-yl)-4-phenyl-2-(*o*-tolyl)but-3-yn-2-ol (**2da**) (134 mg, 0.3 mmol), the crude product was obtained, and isolated after column chromatography (hexane/EtOAc, 50/1), as a 8/1 (**7da**/**8da**) mixture of regioisomers; pale pink solid (62 mg, 48%); mp = 124–126 °C; R_f = 0.28 (hexane/EtOAc, 50/1). Data for **7da**: **^{1}H NMR** (300 MHz, $CDCl_3$) δ (ppm) 7.55–7.30 (m, 6H), 7.29–7.16 (m, 3H), 6.93 (d, J = 3.2 Hz, 1H), 6.89 (s, 1H), 6.66 (d, J = 3.2 Hz, 1H), 3.37 (s, 3H), 2.29 (s, 3H), 0.91 (s, 9H), –0.07 (s, 3H), –0.28 (s, 3H). **^{13}C NMR** (75.4 MHz, $CDCl_3$) δ (ppm): 144.9 (C), 140.4 (C), 140.0 (C), 137.8 (C), 135.1 (C), 131.7 (2 × CH), 130.5 (2 × CH), 129.8 (CH), 129.7 (CH), 128.1 (CH), 127.7 (C), 126.9 (CH), 126.8 (CH), 125.3 (CH), 123.6 (C), 122.8 (C), 120.9 (C), 99.9 (CH), 36.9 (CH_3), 25.9 (3 × CH_3), 20.5 (CH_3), 18.4 (C), –3.9 (CH_3), –4.3 (CH_3). **HRMS** (ESI-TOF): calculated for $C_{28}H_{34}NOSi^{+}$ $[M+H]^{+}$ 428.2404; found 428.2415.

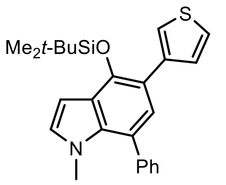

7ea

4-((*Tert*-butyldimethylsilyl)oxy)-1-methyl-7-phenyl-5-(thiophen-3-yl)-1*H*-indole (7ea): Following the general procedure F with 1-((*tert*-butyldimethylsilyl)oxy)-1-(1-methyl-1*H*-pyrrol-2-yl)-4-phenyl-2-(thiophen-3-yl)but-3-yn-2-ol (**2ea**) (131 mg, 0.3 mmol), the crude product was obtained, and isolated after column chromatography (hexane/EtOAc, 50/1), as a 6/1 (**7ea**/**8ea**) mixture of regioisomers; pale pink solid (115 mg, 91%); mp = 63–65 °C; R_f = 0.29 (hexane/EtOAc, 50/1). Data for **7ea**: **^{1}H NMR** (300 MHz, $CDCl_3$) δ (ppm): 7.55–7.31 (m, 8H), 7.09 (s, 1H), 6.91 (d, J = 3.2 Hz, 1H), 6.63 (d, J = 3.2 Hz, 1H), 3.33 (s, 3H), 1.06 (s, 9H), –0.13 (s, 6H). **^{13}C**

NMR (75.4 MHz, $CDCl_3$) δ (ppm): 145.0 (C), 140.6 (C) 140.3 (C), 135.1 (C), 130.5 (2 × CH), 129.9 (CH), 129.9 (CH), 127.8 (2 × CH), 127.1 (CH), 127.0 (CH), 124.20 (CH), 124.15 (C), 122.2 (CH), 121.3 (C), 118.0 (C), 100.1 (CH), 36.8 (CH_3), 26.1 (3 × CH_3), 18.5 (C), –4.3 (2 × CH_3). **HRMS** (ESI-TOF): calculated for $C_{25}H_{30}NOSSi^+$ $[M+H]^+$ 420.1812; found 420.1821.

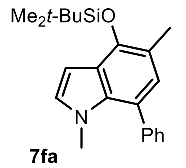

7fa

4-((*Tert*-butyldimethylsilyl)oxy)-1,5-dimethyl-7-phenyl-1*H*-indole (7fa): Following the general procedure F with 1-((*tert*-butyldimethylsilyl)oxy)-2-methyl-1-(1-methyl-1*H*-pyrrol-2-yl)-4-phenylbut-3-yn-2-ol (**2fa**) (111 mg, 0.3 mmol) the crude product was obtained as a 17/1 (**7fa**/**8fa**) mixture of regioisomers, and was purified by column chromatography (hexane/EtOAc, 40/1), affording pure **7fa** as a yellow oil (95 mg, 90%); R_f = 0.27 (hexane/EtOAc, 40/1). **^{1}H NMR** (300 MHz, $CDCl_3$) δ (ppm): 7.49–7.36 (m, 5H), 6.85 (d, J = 3.2 Hz, 1H), 6.83 (s, 1H), 6.53 (d, J = 3.2 Hz, 1H), 3.29 (s, 3H), 2.34 (s, 3H), 1.15 (s, 9H), 0.27 (s, 6H). **^{13}C NMR** (75.4 MHz, $CDCl_3$) δ (ppm): 145.6 (C), 140.7 (C), 134.4 (C), 130.4 (2 × CH), 129.4 (CH), 127.9 (CH), 127.6 (2 × CH), 126.9 (CH), 123.4 (C), 120.8 (C), 117.0 (C), 99.2 (CH), 36.7 (CH_3), 26.2 (3 × CH_3), 18.8 (C), 16.6 (CH_3), –3.2 (2 × CH_3). **HRMS** (ESI-TOF): calculated for $C_{22}H_{30}NOSi^+$ $[M+H]^+$ 352.2091; found 352.2091.

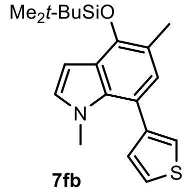

7fb

4-((*Tert*-butyldimethylsilyl)oxy)-1,5-dimethyl-7-(thiophen-3-yl)-1*H*-indole (7fb): Following the general procedure F with 1-((*tert*-butyldimethylsilyl)oxy)-2-methyl-1-(1-methyl-1*H*-pyrrol-2-yl)-4-(thiophen-3-yl)but-3-yn-2-ol (**2fb**) (113 mg, 0.3 mmol), the crude product was obtained as a 16/1 (**7fb**/**8fb**) mixture of regioisomers, and was purified by column chromatography (hexane/EtOAc, 40/1), affording pure **7fb** as a colourless solid (79 mg, 74%); mp = 126–128 °C; R_f = 0.28 (hexane/EtOAc, 40/1). **^{1}H NMR** (300 MHz, $CDCl_3$) δ (ppm): 7.34 (dd, J = 4.9, 3.0 Hz, 1H), 7.22 (dd, J = 3.0, 1.3 Hz, 1H), 7.16 (dd, J = 4.9, 1.3 Hz, 1H), 6.87–6.79 (m, 2H), 6.48 (d, J = 3.2 Hz, 1H), 3.33 (s, 3H), 2.29 (s, 3H), 1.11 (s, 9H), 0.23 (s, 6H). **^{13}C NMR** (75.4 MHz, $CDCl_3$) δ (ppm): 145.8 (C), 140.5 (C), 134.7 (C), 130.6 (CH), 129.2 (CH), 127.9 (CH), 124.2 (CH), 123.2 (C), 123.0 (CH), 116.9 (C), 115.0 (C), 99.0 (CH), 35.9 (CH_3), 26.1 (3 × CH_3), 18.6 (C), 16.4 (CH_3), –3.30 (2 × CH_3). **HRMS** (ESI-TOF): calculated for $C_{20}H_{28}NOSSi^+$ $[M+H]^+$ 358.1655; found 358.1660.

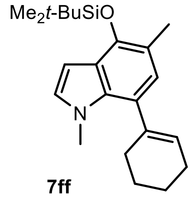

7ff

4-((*Tert*-butyldimethylsilyl)oxy)-7-(cyclohex-1-en-1-yl)-1,5-dimethyl-1*H*-indole (7ff): Following the general procedure F with 1-((*tert*-butyldimethylsilyl)oxy)-4-(cyclohex-1-en-1-yl)-2-methyl-1-(1-methyl-1*H*-pyrrol-2-yl)but-3-yn-2-ol (**2ff**) (112 mg, 0.3 mmol), the crude product was obtained, and isolated after column chromatography (hexane/EtOAc, 40/1), as a 14/1 (**7ff**/**8ff**) mixture of regioisomers; yellow gel (70 mg, 66%); R_f = 0.25 (hexane/EtOAc, 40/1). Data for **7ff**: **^{1}H NMR** (300 MHz, $CDCl_3$) δ (ppm): 6.85 (d, J = 3.2 Hz, 1H), 6.68 (s, 1H), 6.46 (d, J = 3.2 Hz, 1H), 5.78–5.67 (m, 1H), 3.80 (s, 3H), 2.45–2.15 (m, 7H), 1.95–1.68 (m, 4H), 1.12 (s, 9H), 0.23 (s, 6H). **^{13}C NMR** (75.4 MHz, $CDCl_3$) δ (ppm): 144.8 (C), 137.1 (C), 134.1 (C), 128.9 (CH), 127.0 (CH), 126.0 (CH), 123.4 (C), 116.8 (C), 99.1 (CH), 35.6 (CH_3), 32.7 (CH_2), 26.2 (3 × CH_3), 25.6 (CH_2), 23.2 (CH_2), 22.3 (CH_2),

18.7 (C), 16.5 (CH_3), -3.19 (2 × CH_3). **HRMS** (ESI-TOF): calculated for $C_{22}H_{34}NOSi^+$ [M+H]$^+$ 356.2404; found 356.2406.

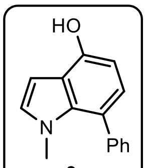

3ga

1-Methyl-7-phenyl-1*H*-indol-4-ol (3ga): Following the general procedure F with 1-((*tert*-butyldimethylsilyl)oxy)-1-(1-methyl-1*H*-pyrrol-2-yl)-4-phenylbut-3-yn-2-ol (**2ga**) (107 mg, 0.3 mmol), the crude product was obtained as a >20/1 (**3ga**/**5ga**) mixture of regioisomers, and was purified by column chromatography (hexane/EtOAc, 10/1), affording pure **3ga** as a colourless solid (43 mg, 64%); mp = 147–149 °C; R_f = 0.28 (hexane/EtOAc, 5/1). **^{1}H NMR** (300 MHz, $CDCl_3$) δ (ppm): 7.51–7.33 (m, 5H), 7.03 (d, *J* = 2.5 Hz, 1H), 6.94 (d, *J* = 3.2 Hz, 1H), 6.62 (d, *J* = 2.5 Hz, 1H), 6.41 (d, *J* = 3.2 Hz, 1H), 4.63 (s, 1H), 3.25 (s, 3H). **^{13}C NMR** (75.4 MHz, $CDCl_3$) δ (ppm): 148.8 (C), 139.9 (C), 132.0 (CH), 130.6 (C), 130.1 (2 × CH), 129.8 (C), 127.8 (2 × CH), 127.7 (C), 127.5 (CH), 114.0 (CH), 104.5 (CH), 100.5 (CH), 36.8 (CH_3). **HRMS** (ESI-TOF): calculated for $C_{15}H_{14}NO^+$ [M+H]$^+$ 224.1070; found 224.1078.

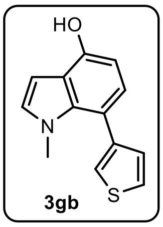

3gb

1-Methyl-7-(thiophen-3-yl)-1*H*-indol-4-ol (3gb): Following the general procedure F with 1-((*tert*-butyldimethylsilyl)oxy)-1-(1-methyl-1*H*-pyrrol-2-yl)-4-(thiophen-3-yl)but-3-yn-2-ol (**2gb**) (108 mg, 0.3 mmol), the crude product was obtained as a >20/1 (**3gb**/**5gb**) mixture of regioisomers, and was purified by column chromatography (hexane/EtOAc, 5/1), affording pure **3gb** as a yellow oil (34 mg, 50%); R_f = 0.20 (hexane/EtOAc, 5/1). **^{1}H NMR** (300 MHz, $CDCl_3$) δ (ppm): 7.39 (dd, *J* = 4.8, 3.0 Hz, 1H), 7.31–7.26 (m, 1H), 7.25–7.16 (m, 1H), 7.05 (d, *J* = 2.5 Hz, 1H), 6.97 (d, *J* = 3.2 Hz, 1H), 6.68 (d, *J* = 2.5 Hz, 1H), 6.42 (d, *J* = 3.2 Hz, 1H), 4.53 (bs, 1H), 3.35 (s, 3H). **^{13}C NMR** (75.4 MHz, $CDCl_3$) δ (ppm): 148.8 (C), 139.9 (C), 131.9 (CH), 130.5 (C), 130.3 (CH), 130.2 (C), 124.7 (CH), 123.7 (CH), 122.3 (C), 114.1 (CH), 104.8 (CH), 100.5 (CH), 36.2 (CH_3). **HRMS** (ESI-TOF): calculated for $C_{13}H_{12}NOS^+$ [M+H]$^+$ 230.0634; found 230.0640.

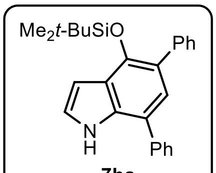

7ha

4-((*Tert*-butyldimethylsilyl)oxy)-5,7-diphenyl-1*H*-indole (7ha): Following the general procedure F with 1-((*tert*-butyldimethylsilyl)oxy)-2,4-diphenyl-1-(1*H*-pyrrol-2-yl)but-3-yn-2-ol (**2ha**) (125 mg, 0.3 mmol), the crude product was obtained as a >20/1 (**7ha**/**8ha**) mixture of regioisomers, and was purified by column chromatography (hexane/EtOAc, 15/1), affording pure **7ha** as a colourless solid (101 mg, 84%); mp = 146–148 °C; R_f = 0.38 (hexane/EtOAc, 10/1). **^{1}H NMR** (300 MHz, $CDCl_3$) δ (ppm): 8.42 (bs, 1H), 7.74–7.62 (m, 4H), 7.58–7.48 (m, 2H), 7.47–7.24 (m, 5H), 7.18 (at, *J* = 2.8 Hz, 1H), 6.80–6.66 (m, 1H), 1.02 (s, 9H), –0.18 (s, 6H). **^{13}C NMR** (75.4 MHz, $CDCl_3$) δ (ppm): 145.2 (C), 140.5 (C), 139.2 (C), 134.9 (C), 130.6 (2 × CH), 129.3 (2 × CH), 128.2 (2 × CH), 128.1 (2 × CH), 127.2 (CH), 126.2 (CH), 125.6 (CH), 124.4 (C), 123.4 (CH), 122.7 (C), 120.1 (C), 102.1 (CH), 26.0 (3 × CH_3), 18.5 (C), –4.2 (2 × CH_3). **HRMS** (ESI-TOF): calculated for $C_{26}H_{30}NOSi^+$ [M+H]$^+$ 400.2091; found 400.2095.

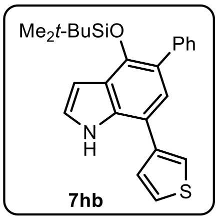

4-((*T*ert-butyldimethylsilyl)oxy)-5-phenyl-7-(thiophen-3-yl)-1*H*-indole (7hb): Following the general procedure F with 1-((*tert*-butyldimethylsilyl)oxy)-2-phenyl-1-(1*H*-pyrrol-2-yl)-4-(thiophen-3-yl)but-3-yn-2-ol (**2hb**) (127 mg, 0.3 mmol), the crude product was obtained as a >20/1 (**7hb**/**8hb**) mixture of regioisomers, and was purified by column chromatography (hexane/EtOAc, 10/1), affording pure **7hb** as a colourless solid (101 mg, 83%); mp = 161–163 °C; R_f = 0.31 (hexane/EtOAc, 10/1). **^{1}H NMR** (300 MHz, $CDCl_3$) δ (ppm): 8.39 (bs, 1H), 7.76–7.59 (m, 2H), 7.53–7.38 (m, 5H), 7.36–7.28 (m, 2H), 7.21–7.09 (m, 1H), 6.79–6.62 (m, 1H), 1.01 (s, 9H), –0.19 (s, 6H). **^{13}C NMR** (75.4 MHz, $CDCl_3$) δ (ppm): 145.1 (C), 140.4 (C), 139.8 (C), 135.0 (C), 130.6 (2 × CH), 128.1 (2 × CH), 127.6 (CH), 126.7 (CH), 126.3 (CH), 125.2 (CH), 124.3 (C), 123.4 (CH), 122.8 (C), 120.7 (CH), 115.0 (C), 102.2 (CH), 26.0 (3 × CH_3), 18.5 (C), –4.2 (2 × CH_3). **HRMS** (ESI-TOF): calculated for $C_{24}H_{28}NOSSi^+$ $[M+H]^+$ 406.1655; found 406.1658.

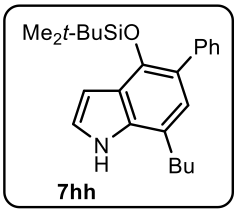

7-Butyl-4-((*tert*-butyldimethylsilyl)oxy)-5-phenyl-1*H*-indole (7hh): Following the general procedure F with 1-((*tert*-butyldimethylsilyl)oxy)-2-phenyl-1-(1*H*-pyrrol-2-yl)-4-(thiophen-3-yl)but-3-yn-2-ol (**2hh**) (119 mg, 0.3 mmol), the crude product was obtained as a 10/1 (**7hh**/**8hh**) mixture of regioisomers and was purified by column chromatography (hexane/EtOAc, 15/1), affording pure **7hh** as a colourless solid (97 mg, 85%); mp = 142–144 °C; R_f = 0.41 (hexane/EtOAc, 10/1). **^{1}H NMR** (300 MHz, $CDCl_3$) δ (ppm): 8.07 (bs, 1H), 7.70–7.58 (m, 2H), 7.50–7.38 (m, 2H), 7.36–7.27 (m, 1H), 7.16 (at, *J* = 2.8 Hz, 1H), 7.03 (s, 1H), 6.74–6.62 (m, 1H), 2.93–2.81 (m, 2H), 1.78 (quin, *J* = 7.6 Hz, 1H), 1.49 (sext, *J* = 7.3 Hz, 1H), 1.13–0.93 (m, 12H), –0.22 (s, 6H). **^{13}C NMR** (75.4 MHz, $CDCl_3$) δ (ppm): 143.6 (C), 140.9 (C), 136.1 (C), 130.6 (2 × CH), 128.0 (2 × CH), 126.0 (CH), 124.9 (CH), 123.6 (C), 122.8 (CH), 122.1 (C), 119.2 (C), 102.0 (CH), 32.0 (CH_2), 30.7 (CH_2), 26.0 (3 × CH_3), 22.9 (CH_2), 18.4 (C), 14.2 (CH_3), –4.3 (2 × CH_3). **HRMS** (ESI-TOF): calculated for $C_{24}H_{34}NOSi^+$ $[M+H]^+$ 380.2404; found 380.2412.

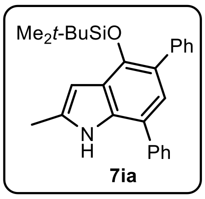

4-((*Tert*-butyldimethylsilyl)oxy)-2-methyl-5,7-diphenyl-1*H*-indole (7ia): Following the general procedure F (with an additional catalyst loading after 8 h) with 1-((*tert*-butyldimethylsilyl)oxy)-1-(5-methyl-1*H*-pyrrol-2-yl)-2,4-diphenylbut-3-yn-2-ol (**2ia**) (119 mg, 0.3 mmol), the crude product was obtained as a 13/1 (**7ia**/**8ia**) mixture of regioisomers, and was purified by column chromatography (hexane/EtOAc, 15/1), affording pure **7ia** as a yellow gel (78 mg, 85%); R_f = 0.41 (hexane/EtOAc, 10/1). **^{1}H NMR** (300 MHz, $CDCl_3$) δ (ppm): 8.12 (bs, 1H), 7.75–7.61 (m, 4H), 7.58–7.48 (m, 2H), 7.47–7.27 (m, 4H), 6.38 (s, 1H), 2.49 (s, 3H), 1.01 (s, 9H), –0.18 (s, 6H). **^{13}C NMR** (75.4 MHz, $CDCl_3$) δ (ppm): 144.3 (C), 140.7 (C), 139.5 (C), 135.0 (C), 134.2 (C), 130.6 (2 × CH), 129.2 (2 × CH), 128.2 (2 × CH), 128.0 (2 × CH), 127.0 (CH), 126.1 (CH), 124.5 (CH), 124.4 (C), 123.6 (C), 119.4 (C), 99.7 (CH), 26.0 (3 × CH_3), 18.5 (C), 14.0 (CH_3), –4.2 (2 × CH_3). **HRMS** (ESI-TOF): calculated for $C_{27}H_{32}NOSi^+$ $[M+H]^+$ 414.2248; found 414.2250.

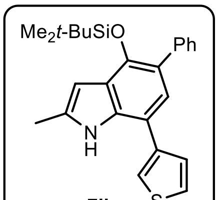

7ib

4-((*Tert*-butyldimethylsilyl)oxy)-2-methyl-5-phenyl-7-(thiophen-3-yl)-1*H*-indole (7ib): Following the general procedure F (with an additional catalyst loading after 8 h) with 1-((*tert*-butyldimethylsilyl)oxy)-1-(5-methyl-1*H*-pyrrol-2-yl)-2-phenyl-4-(thiophen-3-yl)but-3-yn-2-ol (**2ib**) (131 mg, 0.3 mmol), the crude product was obtained as a 15/1 (**7ib**/**8ib**) mixture of regioisomers, and was purified by column chromatography (hexane/EtOAc, 15/1), affording pure **7ib** as a yellow solid (73 mg, 55%); mp = 185–187 °C; R_f = 0.41 (hexane/EtOAc, 10/1). **^{1}H NMR** (300 MHz, $CDCl_3$) *δ* (ppm): 8.12 (bs, 1H), 7.67–7.59 (m, 2H), 7.54–7.37 (m, 5H), 7.35–7.28 (m, 1H), 7.21 (s, 1H), 6.36 (s, 1H), 2.49 (s, 3H), 0.99 (s, 9H), –0.21 (s, 6H). **^{13}C NMR** (75.4 MHz, $CDCl_3$) *δ* (ppm): 144.2 (C), 140.6 (C), 140.0 (C), 135.1 (C), 134.3 (C), 130.6 (2 × CH), 128.0 (2 × CH), 127.7 (CH), 126.6 (CH), 126.2 (CH), 124.3 (CH), 124.1 (CH), 123.6 (C), 120.6 (CH), 114.4 (C), 99.8 (CH), 26.0 (3 × CH_3), 18.5 (C), 14.0 (CH_3), –4.2 (2 × CH_3). **HRMS** (ESI-TOF): calculated for $C_{25}H_{30}NOSSi^+$ $[M+H]^+$ 420.1812; found 420.1821.

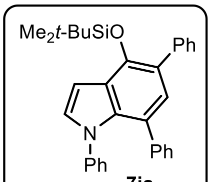

7ja

4-((*Tert*-butyldimethylsilyl)oxy)-1,5,7-triphenyl-1*H*-indole (7ja): Following the general procedure F with 1-((*tert*-butyldimethylsilyl)oxy)-2,4-diphenyl-1-(1-phenyl-1*H*-pyrrol-2-yl)but-3-yn-2-ol (**2ja**) (148 mg, 0.3 mmol), the crude product was obtained as a >20/1 (**7ja**/**8ja**) mixture of regioisomers, and was purified by column chromatography (hexane/EtOAc, 50/1), affording pure **7ja** as a colourless solid (114 mg, 80%); mp = 145–147 °C; R_f = 0.32 (hexane/EtOAc, 50/1). **^{1}H NMR** (300 MHz, $CDCl_3$) *δ* (ppm): 7.74–7.59 (m, 2H), 7.47–7.37 (m, 2H), 7.35–7.29 (m, 1H), 7.24–7.17 (m, 2H), 7.13–6.93 (m, 10H), 6.85 (d, *J* = 3.2 Hz, 1H), 1.04 (s, 9H), –0.17 (s, 6H). **^{13}C NMR** (75.4 MHz, $CDCl_3$) *δ* (ppm): 144.8 (C), 140.4 (C), 140.2 (C), 139.3 (C), 134.1 (C), 130.5 (2 × CH), 129.8 (CH), 129.3 (2 × CH), 128.4 (CH), 128.2 (2 × CH), 128.1 (2 × CH), 127.3 (2 × CH), 126.3 (CH), 126.2 (CH), 125.9 (CH), 125.8 (2 × CH), 124.8 (C), 124.2 (C), 121.6 (C), 102.2 (CH), 26.1 (3 × CH_3), 18.5 (C), –4.1 (2 × CH_3). **HRMS** (ESI-TOF): calculated for $C_{32}H_{34}NOSi^+$ $[M+H]^+$ 476.2404; found 476.2410.

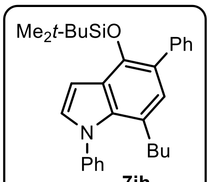

7jh

7-Butyl-4-((*tert*-butyldimethylsilyl)oxy)-1,5-diphenyl-1*H*-indole (7jh): Following the general procedure F with 1-((*tert*-butyldimethylsilyl)oxy)-2-phenyl-1-(1-phenyl-1*H*-pyrrol-2-yl)oct-3-yn-2-ol (**2jh**) (129 mg, 0.3 mmol), the crude product was obtained as a >20/1 (**7jh**/**8jh**) mixture of regioisomers, and was purified by column chromatography (hexane/EtOAc, 50/1), affording pure **7jh** as a yellow solid (111 mg, 81%); mp = 133–135 °C; R_f = 0.39 (hexane/EtOAc, 50/1). **^{1}H NMR** (300 MHz, $CDCl_3$) *δ* (ppm): 7.69–7.58 (m, 2H), 7.55–7.36 (m, 7H), 7.36–7.24 (m, 1H), 7.07 (d, *J* = 3.2 Hz, 1H), 6.98 (s, 1H), 6.74 (d, *J* = 2.7 Hz, 1H), 2.47–2.33 (m, 2H), 1.39–1.20 (m, 2H), 1.11–0.88 (m, 11H), 0.69 (t, *J* = 7.3 Hz, 3H), –0.21 (s, 6H). **^{13}C NMR** (75.4 MHz, $CDCl_3$) *δ* (ppm): 143.4 (C), 141.6 (C), 140.7 (C), 136.0 (C), 130.5 (2 × CH), 129.9 (CH), 128.8 (2 × CH), 128.1 (2 × CH), 128.04 (CH), 128.00 (2 × CH), 126.9 (CH), 126.1 (CH), 124.3 (C), 123.7 (C), 121.1 (C), 101.7 (CH), 33.5 (CH_2), 31.7 (CH_2), 26.1 (3 × CH3), 22.7 (CH_2), 18.5 (C), 13.9 (CH_3), –4.2 (2 × CH3). **HRMS** (ESI-TOF): calculated for $C_{30}H_{38}NOSi^+$ $[M+H]^+$ 456.2717; found 456.2726.

Synthesis and characterization data of 4-hydroxyindoles 3

General procedure G for the synthesis of 4-hydroxyindoles 3 from 4-silyloxyindoles 7

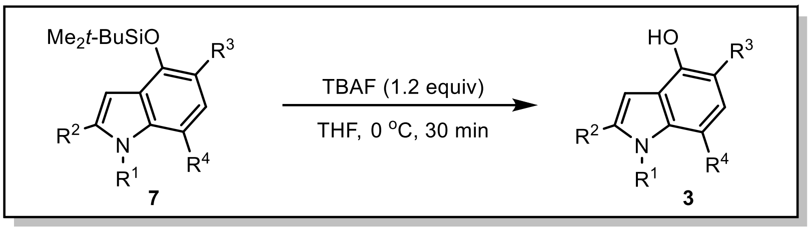

Tetra-*n*-butylammonium fluoride (0.24 mmol, 0.24 mL of a 1 M solution in THF, 1.2 equiv) was added dropwise to the corresponding 4-silyloxyindole **7** (0.2 mmol, 1 equiv) in anhydrous THF (4 mL, 0.05 M). The reaction mixture was stirred at this temperature for 30 min. Then, H_2O (4 mL) was added and the residue was extracted with EtOAc (4 × 5 mL). The combined organic layers were dried over anhydrous Na_2SO_4 and the solvents were removed under reduced pressure. The residue was purified by flash column chromatography using mixtures of hexane and EtOAc as eluents to obtain the corresponding 4-hydroxyindoles **3** (Table S3). The characterization data for **3aa**, **3ab**, **3af**, and **3ah** has been reported above using general procedure E from the corresponding diols **1**.

*Table S3: Synthesis of 4-hydroxyindoles **3** from 4-silyloxyindoles **7***

Entry	7	R^1	R^2	R^3	R^4	3	Yield (%)[a]
1	**7aa**	Me	H	Ph	Ph	**3aa**	80
2	**7ab**	Me	H	Ph	3-Th	**3ab**	76
3	**7ac**	Me	H	Ph	4-$MeOC_6H_4$	**3ac**	72
4	**7ad**	Me	H	Ph	4-ClC_6H_4	**3ad**	75
6	**7af**	Me	H	Ph	*c*-C_6H_9	**3af**	74
7	**7ag**	Me	H	Ph	C(Me)=CH_2	**3ag**	78
8	**7ah**	Me	H	Ph	*n*-Bu	**3ah**	75
9	**7ai**	Me	H	Ph	*c*-C_3H_5	**3ai**	78
11	**7ak**	Me	H	Ph	H	**3ak**	76
12	**7ba**	Me	H	4-FC_6H_4	Ph	**3ba**	80
13	**7bf**	Me	H	4-FC_6H_4	*c*-C_6H_9	**3bf**	78
14	**7ca**	Me	H	4-$MeOC_6H_4$	Ph	**3ca**	89
17	**7fa**	Me	H	Me	Ph	**3fa**	70
18	**7fb**	Me	H	Me	3-Th	**3fb**	88
19	**7ff**	Me	H	Me	*c*-C_6H_9	**3ff**	–[b]
20	**7ha**	H	H	Ph	Ph	**3ha**	94
21	**7hb**	H	H	Ph	3-Th	**3hb**	54
22	**7hh**	H	H	Ph	*n*-Bu	**3hh**	70
23	**7ia**	H	Me	Ph	Ph	**3ia**	93
25	**7ja**	Ph	H	Ph	Ph	**3ja**	–[b]

[a]Isolated yield of **3** referred to the corresponding indole derivative **7** after column chromatography.
[b]Decomposition was observed.

Characterization data:

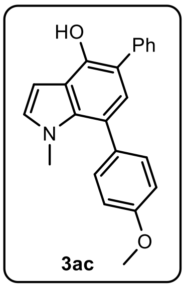

7-(4-Methoxyphenyl)-1-methyl-5-phenyl-1*H*-indol-4-ol (3ac): Following the general procedure G with 4-((*tert*-butyldimethylsilyl)oxy)-7-(4-methoxyphenyl)-1-methyl-5-phenyl-1*H*-indole (**7ac**) (89 mg, 0.2 mmol), the crude product was purified by column chromatography (hexane/EtOAc, 5/1), affording pure **3ac** as a colourless solid (47 mg, 72%); mp = 112–114 °C; R_f = 0.24 (hexane/EtOAc, 5/1). **^{1}H NMR** (300 MHz, $CDCl_3$) δ (ppm): 7.61–7.45 (m, 4H), 7.42–7.32 (m, 3H), 7.00–6.92 (m, 4H), 6.68 (d, J = 3.2 Hz, 1H), 5.57 (s, 1H), 3.88 (s, 3H), 3.36 (s, 3H). **^{13}C NMR** (75.4 MHz, $CDCl_3$) δ (ppm): 158.9 (C), 144.7 (C), 138.1 (C), 135.5 (C), 132.5 (C), 131.5 (2 × CH), 130.3 (CH), 129.6 (2 × CH), 129.4 (2 × CH), 127.2 (CH), 126.7 (CH), 120.0 (C), 119.2 (C), 116.8 (C), 113.2 (2 × CH), 98.4 (CH), 55.4 (CH_3), 36.8 (CH_3). **HRMS** (ESI-TOF): calculated for $C_{22}H_{20}NO_2^+$ $[M+H]^+$ 330.1489; found 330.1489.

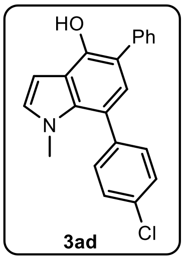

7-(4-Chlorophenyl)-1-methyl-5-phenyl-1*H*-indol-4-ol (3ad): Following the general procedure G with 4-((*tert*-butyldimethylsilyl)oxy)-7-(4-chlorophenyl)-1-methyl-5-phenyl-1*H*-indole (**7ad**) (89 mg, 0.2 mmol), the crude product was purified by column chromatography (hexane/EtOAc, 5/1), affording pure **3ad** as a colourless solid (50 mg, 75%); mp = 178–180 °C; R_f = 0.33 (hexane/EtOAc, 5/1). **^{1}H NMR** (300 MHz, $CDCl_3$) δ (ppm): 7.62–7.46 (m, 4H), 7.44–7.33 (m, 5H), 6.96 (d, J = 3.2 Hz, 1H), 6.94 (s, 1H), 6.71 (d, J = 3.2 Hz, 1H), 5.60 (s, 1H), 3.37 (s, 3H). **^{13}C NMR** (75.4 MHz, $CDCl_3$) δ (ppm): 145.2 (C), 138.7 (C), 137.8 (C), 135.2 (C), 133.2 (C), 131.7 (2 × CH), 130.4 (CH), 129.6 (2 × CH), 129.4 (2 × CH), 128.0 (2 × CH), 127.3 (CH), 126.6 (CH), 119.3 (C), 118.9 (C), 117.0 (C), 98.7 (CH), 36.9 (CH_3). **HRMS** (ESI-TOF): calculated for $C_{21}H_{17}NO^+$ $[M+H]^+$ 334.0993; found 334.0998.

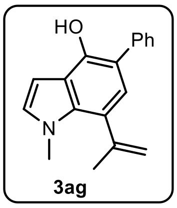

1-Methyl-5-phenyl-7-(prop-1-en-2-yl)-1*H*-indol-4-ol (3ag): Following the general procedure G with 4-((*tert*-butyldimethylsilyl)oxy)-1-methyl-5-phenyl-7-(prop-1-en-2-yl)-1*H*-indole (**7ag**) (76 mg, 0.2 mmol), the crude product was purified by column chromatography (hexane/EtOAc, 10/1), affording pure **3ag** as a yellow oil (41 mg, 78%); R_f = 0.19 (hexane/EtOAc, 10/1). **^{1}H NMR** (300 MHz, $CDCl_3$) δ (ppm): 7.63–7.47 (m, 4H), 7.44–7.34 (m, 1H), 6.99 (d, J = 3.2 Hz, 1H), 6.91 (s, 1H), 6.68 (d, J = 3.2 Hz, 1H), 5.53 (s, 1H), 5.39–5.33 (m, 1H), 5.11–5.05 (m, 1H), 3.89 (s, 3H), 2.23 (s, 3H). **^{13}C NMR** (75.4 MHz, $CDCl_3$) δ (ppm): 144.5 (C), 143.9 (C), 138.2 (C), 134.5 (C), 130.0 (CH), 129.6 (2 × CH), 129.3 (2 × CH), 127.1 (CH), 124.4 (CH), 122.0 (C), 119.3 (C), 116.7 (C), 116.5 (CH_2), 98.6 (CH), 35.6 (CH_3), 26.8 (CH_3). **HRMS** (ESI-TOF): calculated for $C_{18}H_{18}NO^+$ $[M+H]^+$ 264.1383; found 264.1384.

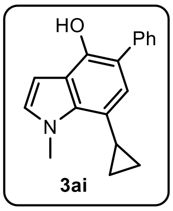

7-Cyclopropyl-1-methyl-5-phenyl-1*H*-indol-4-ol (3ai): Following the general procedure G with 4-((*tert*-butyldimethylsilyl)oxy)-7-cyclopropyl-1-methyl-5-phenyl-1*H*-indole (**7ai**) (75 mg, 0.2 mmol), the crude product was purified by column chromatography (hexane/EtOAc, 5/1), affording pure **3ai** as an orange oil (41 mg, 78%); R_f = 0.28 (hexane/EtOAc, 5/1). **^{1}H NMR** (300 MHz, $CDCl_3$) δ (ppm): 7.60–7.43 (m, 4H), 7.43–7.31 (m, 1H), 6.95 (d, J = 3.2 Hz, 1H), 6.87 (s, 1H), 6.60 (d, J = 3.2 Hz, 1H), 5.42 (s, 1H),

4.23 (s, 3H), 2.56–2.27 (m, 1H), 1.08–0.79 (m, 4H). **^{13}C NMR** (75.4 MHz, $CDCl_3$) *δ* (ppm): 144.2 (C), 138.4 (C), 137.4 (C), 129.9 (CH), 129.6 (2 × CH), 129.3 (2 × CH), 127.1 (CH), 124.3 (CH), 119.4 (C), 119.2 (C), 116.5 (C), 98.3 (CH), 36.8 (CH_3), 13.1 (CH), 7.8 (2 × CH_2). **HRMS** (ESI-TOF): calculated for $C_{18}H_{18}NO^+$ $[M+H]^+$ 264.1383; found 264.1385.

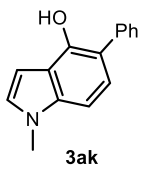
3ak

1-Methyl-5-phenyl-1*H*-indol-4-ol (3ak): Following the general procedure G with 4-((*tert*-butyldimethylsilyl)oxy)-1-methyl-5-phenyl-1*H*-indole (**7ak**) (67 mg, 0.2 mmol), the crude product was purified by column chromatography (hexane/EtOAc, 5/1), affording pure **3ak** as an orange solid (34 mg, 76%); mp = 102–104 °C; R_f = 0.32 (hexane/EtOAc, 5/1). **^{1}H NMR** (300 MHz, $CDCl_3$) *δ* (ppm): 7.58–7.33 (m, 5H), 7.13 (d, *J* = 8.4 Hz, 1H), 7.02 (d, *J* = 3.2 Hz, 1H), 6.99 (d, *J* = 8.4 Hz, 1H), 6.62 (d, *J* = 3.2 Hz, 1H), 5.54 (s, 1H), 3.80 (s, 3H). **^{13}C NMR** (75.4 MHz, $CDCl_3$) *δ* (ppm): 145.6 (C), 138.4 (C), 138.3 (C), 129.7 (2 × CH), 129.3 (2 × CH), 128.2 (CH), 127.1 (CH), 124.3 (CH), 118.1 (C), 117.2 (C), 102.5 (CH), 98.4 (CH), 33.2 (CH_3). **HRMS** (ESI-TOF): calculated for $C_{15}H_{14}NO^+$ $[M+H]^+$ 224.1070; found 224.1077.

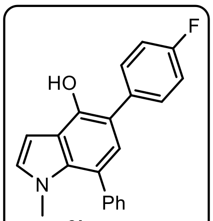
3ba

5-(4-Fluorophenyl)-1-methyl-7-phenyl-1*H*-indol-4-ol (3ba): Following the general procedure G with 4-((*tert*-butyldimethylsilyl)oxy)-5-(4-fluorophenyl)-1-methyl-7-phenyl-1*H*-indole (**7ba**) (86 mg, 0.2 mmol), the crude product was purified by column chromatography (hexane/EtOAc, 10/1), affording pure **3ba** as a pale pink solid (51 mg, 80%); mp = 152–154 °C; R_f = 0.17 (hexane/EtOAc, 10/1). **^{1}H NMR** (300 MHz, $CDCl_3$) *δ* (ppm): 7.63–7.31 (m, 7H), 7.23–7.12 (m, 2H), 6.96 (d, *J* = 3.2 Hz, 1H), 6.92 (s, 1H), 6.67 (d, *J* = 3.2 Hz, 1H), 5.37 (s, 1H), 3.34 (s, 3H). **^{13}C NMR** (75.4 MHz, $CDCl_3$) *δ* (ppm): 162.2 (d, $^1J_{C-F}$ = 246.2 Hz, C), 144.8 (C), 140.0 (C), 135.3 (C), 134.0 (d, $^4J_{C-F}$ = 3.4 Hz, C), 131.3 (d, $^3J_{C-F}$ = 8.0 Hz, 2 × CH), 130.5 (2 × CH), 127.8 (2 × CH), 127.2 (CH), 126.6 (CH), 120.5 (C), 119.3 (C), 116.1 (d, $^2J_{C-F}$ = 21.4 Hz, 2 × CH), 116.0 (C), 98.3 (CH), 36.8 (CH_3). One CH peak was missing due to overlapping. **HRMS** (ESI-TOF): calculated for $C_{21}H_{17}FNO^+$ $[M+H]^+$ 318.1289; found 318.1297.

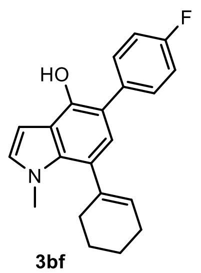
3bf

7-(Cyclohex-1-en-1-yl)-5-(4-fluorophenyl)-1-methyl-1*H*-indol-4-ol (3bf): Following the general procedure G with 4-((*tert*-butyldimethylsilyl)oxy)-7-(cyclohex-1-en-1-yl)-5-(4-fluorophenyl)-1-methyl-1*H*-indole (**7bf**) (87 mg, 0.2 mmol), the crude product was purified by column chromatography (hexane/EtOAc, 7/1), affording pure **3bf** as a pale pink solid (50 mg, 78%); mp = 133–135 °C; R_f = 0.31 (hexane/EtOAc, 5/1). **^{1}H NMR** (300 MHz, $CDCl_3$) *δ* (ppm): 7.62–7.46 (m, 2H), 7.24–7.11 (m, 2H), 6.96 (d, *J* = 3.2 Hz, 1H), 6.80 (s, 1H), 6.62 (d, *J* = 3.2 Hz, 1H), 5.78 (tt, *J* = 3.8, 1.8 Hz, 1H), 5.28 (s, 1H), 3.87 (s, 3H), 2.46–2.33 (m, 2H), 2.32–2.19 (m, 2H), 1.91–1.68 (m, 4H). **^{13}C NMR** (75.4 MHz, $CDCl_3$) *δ* (ppm): 162.1 (d, $^1J_{C-F}$ = 246.2 Hz, C), 144.1 (C), 136.6 (C), 135.0 (C), 134.3 (d, $^4J_{C-F}$ = 3.4 Hz, C), 131.3 (d, $^3J_{C-F}$ = 8.0 Hz, 2 × CH), 130.0 (CH), 127.6 (CH), 124.8 (CH), 123.0 (C), 119.2 (C), 116.0 (d, $^2J_{C-F}$ = 21.4 Hz, 2 × CH) 115.8 (C), 98.2 (CH), 35.6 (CH_3), 32.8 (CH_2), 25.6 (CH_2), 23.1 (CH_2), 22.2 (CH_2). **HRMS** (ESI-TOF): calculated for $C_{21}H_{21}FNO^+$ $[M+H]^+$ 322.1602; found 322.1611.

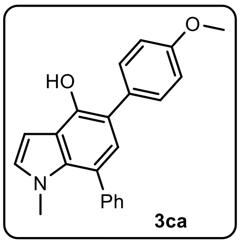

5-(4-Methoxyphenyl)-1-methyl-7-phenyl-1*H*-indol-4-ol (3ca): Following the general procedure G with 4-((*tert*-butyldimethylsilyl)oxy)-5-(4-methoxyphenyl)-1-methyl-7-phenyl-1*H*-indole (**7ca**) (89 mg, 0.2 mmol), the crude product was purified by column chromatography (hexane/EtOAc, 5/1), affording pure **3ca** as a colourless solid (59 mg, 89%); mp = 191–193 °C; R_f = 0.29 (hexane/EtOAc, 5/1). **^{1}H NMR** (300 MHz, $CDCl_3$) δ (ppm): 7.61–7.39 (m, 7H), 7.12–7.02 (m, 2H), 7.02–6.92 (m, 2H), 6.73 (d, J = 3.2 Hz, 1H), 5.59 (s, 1H), 3.90 (s, 3H), 3.38 (s, 3H). **^{13}C NMR** (75.4 MHz, $CDCl_3$) δ (ppm): 158.9 (C), 144.9 (C), 140.2 (C), 135.1 (C), 130.8 (2 × CH), 130.5 (2 × CH), 130.3 (CH), 130.1 (C), 127.8 (2 × CH), 127.0 (CH), 126.6 (CH), 120.2 (C), 119.2 (C), 116.5 (C), 114.8 (2 × CH), 98.4 (CH), 55.5 (CH_3), 36.8 (CH_3).**HRMS** (ESI-TOF): calculated for $C_{22}H_{20}NO_2^+$ $[M+H]^+$ 330.1489; found 330.1497.

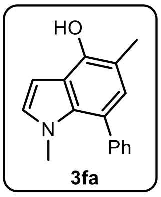

1,5-Dimethyl-7-phenyl-1*H*-indol-4-ol (3fa): Following the general procedure G with 4-((*tert*-butyldimethylsilyl)oxy)-1,5-dimethyl-7-phenyl-1*H*-indole (**7fa**) (70 mg, 0.2 mmol), the crude product was purified by column chromatography (hexane/EtOAc, 5/1), affording pure **3fa** as a yellow oil (33 mg, 70%); R_f = 0.27 (hexane/EtOAc, 5/1). **^{1}H NMR** (300 MHz, $CDCl_3$) δ (ppm): 7.57-7.32 (m, 5H), 6.89 (d, J = 3.2 Hz, 1H), 6.81 (s, 1H), 6.51 (d, J = 3.2 Hz, 1H), 4.87 (s, 1H), 3.29 (s, 3H), 2.35 (s, 3H). **^{13}C NMR** (75.4 MHz, $CDCl_3$) δ (ppm): 145.7 (C), 140.4 (C), 134.3 (C), 130.5 (2 × CH), 130.0 (CH), 127.7 (2 × CH), 127.6 (CH), 127.0 (CH), 120.0 (C), 119.2 (C), 111.2 (C), 96.6 (CH), 36.8 (CH_3), 14.9 (CH_3). **HRMS** (ESI-TOF): calculated for $C_{16}H_{16}NO^+$ $[M+H]^+$ 238.1226; found 238.1227.

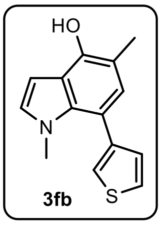

1,5-Dimethyl-7-(thiophen-3-yl)-1*H*-indol-4-ol (3fb): Following the general procedure G with 4-((*tert*-butyldimethylsilyl)oxy)-1,5-dimethyl-7-(thiophen-3-yl)-1*H*-indole (**7fb**) (72 mg, 0.2 mmol), the crude product was purified by column chromatography (hexane/EtOAc, 5/1), affording pure **3fb** as a yellow oil (43 mg, 88%); R_f = 0.26 (hexane/EtOAc, 5/1). **^{1}H NMR** (300 MHz, $CDCl_3$) δ (ppm): 7.43–7.27 (m, 1H), 7.25–7.12 (m, 2H), 6.96–6.79 (m, 2H), 6.50 (d, J = 3.2 Hz, 1H), 4.97–4.83 (m, 1H), 3.36 (s, 3H), 2.35 (s, 3H). **^{13}C NMR** (75.4 MHz, $CDCl_3$) δ (ppm): 146.0 (C), 140.3 (C), 134.7 (C), 130.7 (CH), 130.0 (CH), 127.7 (CH), 124.4 (CH), 123.3 (CH), 119.2 (C), 114.2 (C), 111.2 (C), 96.6 (CH), 36.0 (CH_3), 14.8 (CH_3). **HRMS** (ESI-TOF): calculated for $C_{14}H_{14}NOS^+$ $[M+H]^+$ 244.0791; found 244.0798.

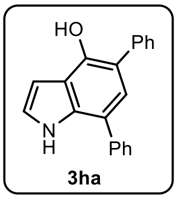

5,7-Diphenyl-1*H*-indol-4-ol (3ha): Following the general procedure G with 4-((*tert*-butyldimethylsilyl)oxy)-5,7-diphenyl-1*H*-indole (**7ha)** (80 mg, 0.2 mmol), the crude product was purified by column chromatography (hexane/EtOAc, 3/1), affording pure **3ha** as a gray solid (54 mg, 94%); mp = 153–155 °C; R_f = 0.28 (hexane/EtOAc, 3/1). **^{1}H NMR** (300 MHz, $CDCl_3$) δ (ppm): 8.47 (bs, 1H), 7.71–7.33 (m, 10H), 7.22–7.18 (m, 1H), 7.16 (s, 1H), 6.82–6.74 (m, 1H), 5.62 (s, 1H). **^{13}C NMR** (75.4 MHz, $CDCl_3$) δ (ppm): 145.2 (C), 139.1 (C), 138.1 (C), 135.1 (C), 129.7 (2 × CH), 129.4 (2 × CH), 129.3 (2 × CH), 128.2 (2 × CH), 127.4 (CH), 127.2

(CH), 124.6 (CH), 123.8 (CH), 119.1 (C), 118.2 (C), 118.0 (C), 100.6 (CH). **HRMS** (ESI-TOF): calculated for $C_{20}H_{16}NO^+$ $[M+H]^+$ 286.1226; found 286.1232.

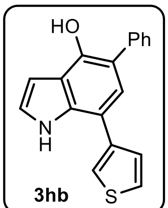
3hb

5-Phenyl-7-(thiophen-3-yl)-1*H*-indol-4-ol (3hb): Following the general procedure G with 4-((*tert*-butyldimethylsilyl)oxy)-5-phenyl-7-(thiophen-3-yl)-1*H*-indole (**7hb**) (81 mg, 0.2 mmol), the crude product was purified by column chromatography (hexane/EtOAc, 3/1), affording pure **3hb** as a gray solid (31 mg, 54%); mp = 142–144 °C; R_f = 0.38 (hexane/EtOAc, 3/1). **^{1}H NMR** (300 MHz, $CDCl_3$) δ (ppm): 8.49 (bs, 1H), 7.66–7.38 (m, 8H), 7.25–7.20 (m, 2H), 6.85–6.75 (m, 1H), 5.64 (s, 1H). **^{13}C NMR** (75.4 MHz, $CDCl_3$) δ (ppm): 145.1 (C), 139.6 (C), 137.9 (C), 135.1 (C), 129.6 (2 × CH), 129.4 (2 × CH), 127.5 (CH), 127.31 (CH), 126.66 (CH), 124.1 (CH), 123.7 (CH), 120.5 (CH), 118.0 (C), 117.9 (C), 114.0 (C), 100.6 (CH). **HRMS** (ESI-TOF): calculated for $C_{18}H_{14}NOS^+$ $[M+H]^+$ 292.0791; found 292.0798.

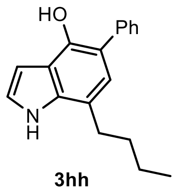
3hh

7-Butyl-5-phenyl-1*H*-indol-4-ol (3hh): Following the general procedure G with 7-butyl-4-((*tert*-butyldimethylsilyl)oxy)-5-phenyl-1*H*-indole (**7hh**) (76 mg, 0.2 mmol), the crude product was purified by column chromatography (hexane/EtOAc, 3/1), affording pure **3hh** as a green oil (37 mg, 70%); R_f = 0.30 (hexane/EtOAc, 3/1). **^{1}H NMR** (300 MHz, $CDCl_3$) δ (ppm): 8.15 (bs, 1H), 7.62–7.45 (m, 4H), 7.44–7.34 (m, 1H), 7.22–7.14 (m, 1H), 6.94 (s, 1H), 6.82–6.77 (m, 1H), 5.49 (s, 1H), 2.89–2.69 (m, 2H), 1.74 (quin, *J* = 7.9 Hz, 2H), 1.46 (sext, *J* = 7.3 Hz, 2H), 0.99 (t, *J* = 7.3 Hz, 3H). **^{13}C NMR** (75.4 MHz, $CDCl_3$) δ (ppm): 143.7 (C), 138.6 (C), 136.2 (C), 129.7 (2 × CH), 129.3 (2 × CH), 127.1 (CH), 123.9 (CH), 123.3 (CH), 118.2 (C), 117.5 (C), 100.4 (CH), 32.1 (CH_2), 30.6 (CH_2), 22.8 (CH_2), 14.1 (CH_2). One quternary peak was missing due to overlapping. **HRMS** (ESI-TOF): calculated for $C_{18}H_{20}NO^+$ $[M+H]^+$ 266.1539; found 266.1547.

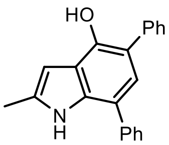
3ia

2-Methyl-5,7-diphenyl-1*H*-indol-4-ol (3ia): Following the general procedure G with 4-((*tert*-butyldimethylsilyl)oxy)-2-methyl-5,7-diphenyl-1*H*-indole **7ia** (83 mg, 0.2 mmol) the crude product was purified by column chromatography (hexane/EtOAc, 5/1), affording pure **3ia** as a colourless solid (56 mg, 93%); mp = 137–139 °C; R_f = 0.31 (hexane/EtOAc, 5/1). **^{1}H NMR** (300 MHz, $CDCl_3$) δ (ppm): 8.16 (bs, 1H), 7.68–7.45 (m, 8H), 7.42–7.32 (m, 2H), 7.07 (s, 1H), 6.43 (s, 1H), 5.51 (s, 1H), 2.47 (s, 3H). **^{13}C NMR** (75.4 MHz, $CDCl_3$) δ (ppm): 144.4 (C), 139.3 (C), 138.3 (C), 135.2 (C), 134.7 (C), 129.7 (2 × CH), 129.4 (2 × CH), 129.3 (2 × CH), 128.2 (2 × CH), 127.2 (CH), 127.0 (CH), 123.6 (CH), 118.7 (C), 118.5 (C), 118.3 (C), 98.2 (CH), 13.9 (CH_3). **HRMS** (ESI-TOF): calculated for $C_{21}H_{18}NO^+$ $[M+H]^+$ 300.1383; found 300.1390.

Synthesis and characterization data of indoline 9[4]

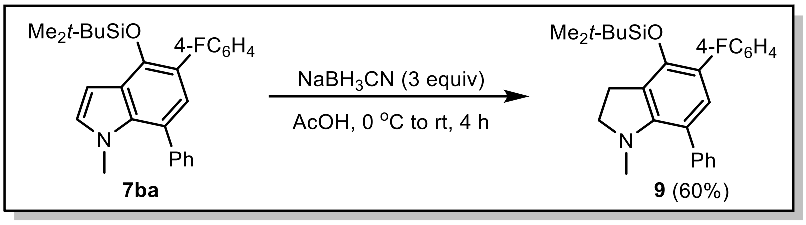

$NaBH_3CN$ (38 mg, 0.6 mmol, 3 equiv) was added to a solution of 4-((*tert*-butyldimethylsilyl)oxy)-5-(4-fluorophenyl)-1-methyl-7-phenyl-1*H*-indole (**7ba**) (86 mg, 0.2 mmol, 1 equiv) in AcOH (0.5 mL, 0.4 M) at 0 °C. The resulting mixture was stirred at rt for 3 h. Then, H_2O (2 mL) was added and the residue was extracted with Et_2O (3 × 5 mL). The combined organic layers were washed with water (2 × 10 mL), dried over anhydrous Na_2SO_4 and the solvents were removed under reduced pressure. The residue was purified by flash column chromatography using a 50/1 mixture of hexane/EtOAc as eluent to obtain the indoline derivative **9** as a pink pale solid (52 mg, 60%).

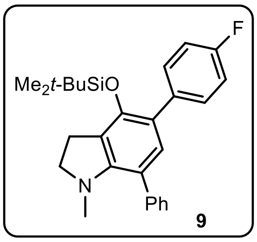

4-((*Tert*-butyldimethylsilyl)oxy)-5-(4-fluorophenyl)-1-methyl-7-phenylindoline (9): mp = 126–128 °C; R_f = 0.26 (hexane/EtOAc, 50/1). **^{1}H NMR** (300 MHz, $CDCl_3$) δ (ppm): 7.49–7.28 (m, 7H), 7.09–6.99 (m, 2H), 6.94 (s, 1H), 3.34 (t, J = 8.4 Hz, 2H), 2.99 (t, J = 8.4 Hz, 2H), 2.37 (s, 3H), 0.92 (s, 9H), –0.25 (s, 6H). **^{13}C NMR** (75.4 MHz, $CDCl_3$) δ (ppm): 161.8 (d, $^1J_{C-F}$ = 244.7 Hz, C), 151.6 (C), 148.1 (C), 140.5 (C), 135.9 (d, $^4J_{C-F}$ = 3.3 Hz, C), 133.1 (CH), 131.4 (d, $^3J_{C-F}$ = 7.8 Hz, 2 × CH), 129.4 (2 × CH), 128.1 (2 × CH), 126.6 (CH), 124.3 (C), 122.3 (C), 119.6 (C), 114.8 (d, $^2J_{C-F}$ = 21.1 Hz, 2 × CH), 58.0 (CH_2), 40.3 (CH_3), 27.2 (CH_2), 26.0 (3 × CH_3), 18.4 (C), –4.1 (2 × CH_3). **HRMS** (ESI-TOF): calculated for $C_{27}H_{33}FNOSi^+$ $[M+H]^+$ 434.2310; found 434.2319.

Synthesis and characterization data of C3-functionalized indole derivative 10[5]

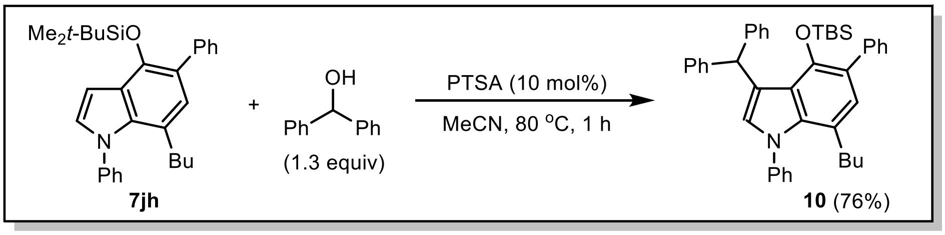

A mixture of 7-butyl-4-((*tert*-butyldimethylsilyl)oxy)-1,5-diphenyl-1*H*-indole (**7jh**) (114 mg, 0.25 mmol, 1 equiv), benzhydrol (60 mg, 0.325 mmol, 1.3 equiv) and *p*-toluenesulfonic acid monohydrate (5 mg, 0.025 mmol, 0.1 equiv) in MeCN (5 mL, 0.05 M) was stirred at 80 °C for 1 h. Then, the reaction mixture was allowed to cool to rt and H_2O (5 mL) was added. The residue was extracted with CH_2Cl_2 (3 × 5 mL). The combined organic layers were dried over anhydrous Na_2SO_4 and the solvents were removed under reduced pressure. The residue was purified by flash column chromatography using a 50/1 mixture of

[4] Kumar, Y.; Florvall, L. *Synth. Commun.* **1983**, *13*, 489–493.
[5] Sanz, R.; Martínez, A.; Miguel, D.; Álvarez-Gutiérrez, J. M.; Rodríguez, F. *Adv. Synth. Catal.* **2006**, *348*, 1841–1845.

hexane/EtOAc as eluent to obtain the C3-functionalized indole derivative **10** as a colourless solid (118 mg, 76%).

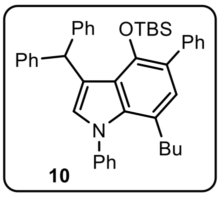

3-Benzhydryl-7-butyl-4-((*tert*-butyldimethylsilyl)oxy)-1,5-diphenyl-1*H*-indole (10): mp = 108–110 °C; R_f = 0.34 (hexane/EtOAc, 50/1). **^{1}H NMR** (300 MHz, $CDCl_3$) δ (ppm): 7.56–7.15 (m, 20H), 6.98 (s, 1H), 6.48 (s, 1H), 6.31 (s, 1H), 2.45–2.25 (m, 2H), 1.34 (quin, J = 7.6 Hz, 2H), 1.05–0.79 (m, 11H), 0.71 (t, J = 7.3 Hz, 3H), –0.24 (s, 6H). **^{13}C NMR** (75.4 MHz, $CDCl_3$) δ (ppm): 145.7 (2 × C), 145.0 (C), 141.8 (C), 141.1 (C), 137.1 (C), 131.3 (CH), 130.8 (2 × CH), 129.5 (4 × CH), 128.7 (2 × CH), 128.5 (CH), 128.1 (2 × CH), 128.0 (4 × CH), 127.8 (CH), 127.4 (CH), 127.2 (CH), 126.0 (CH), 125.9 (2 × CH), 124.9 (C), 122.9 (C), 121.1 (C), 119.6 (C), 47.6 (CH), 33.3 (CH_2), 31.2 (CH_2), 26.7 (3 × CH_3), 22.8 (CH_2), 18.8 (C), 13.9 (CH_3), –3.3 (2 × CH_3). **HRMS** (ESI-TOF): calculated for $C_{43}H_{48}NOSi^+$ $[M+H]^+$ 622.3500; found 622.3506.

Scale-up synthesis of 7aa

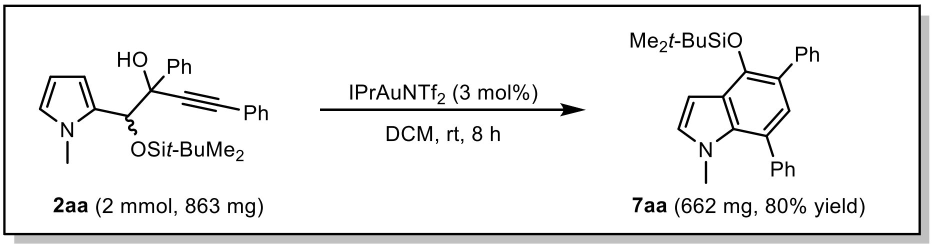

IPrAuNTf$_2$ (52 mg, 0.06 mmol, 0.03 equiv) was added to a solution of 1-((*tert*-butyldimethylsilyl)oxy)-1-(1-methyl-1*H*-pyrrol-2-yl)-2,4-diphenylbut-3-yn-2-ol (**2aa**) (863 mg, 2 mmol, 1 equiv) in anhydrous DCM (50 mL, 0.04 M) with continuous stirring at rt. The reaction mixture was stirred for 8 h. Once the reaction was completed, as determined by TLC, the solvent was evaporated under reduced pressure. The crude product was obtained as a 18/1 (**7aa**/**8aa**) mixture of regioisomers and was purified by column chromatography (hexane/EtOAc, 50/1), affording pure 4-((*tert*-butyldimethylsilyl)oxy)-1-methyl-5,7-diphenyl-1*H*-indole (**7aa**) as a colourless solid (662 mg, 80%); mp = 121–123 °C; R_f = 0.24 (hexane/EtOAc, 50/1).

^{1}H, ^{13}C and selected NOE 1D NMR Spectra

Figure S1: ^{1}H NMR of compound **S1b** in $CDCl_3$ at 300 MHz.

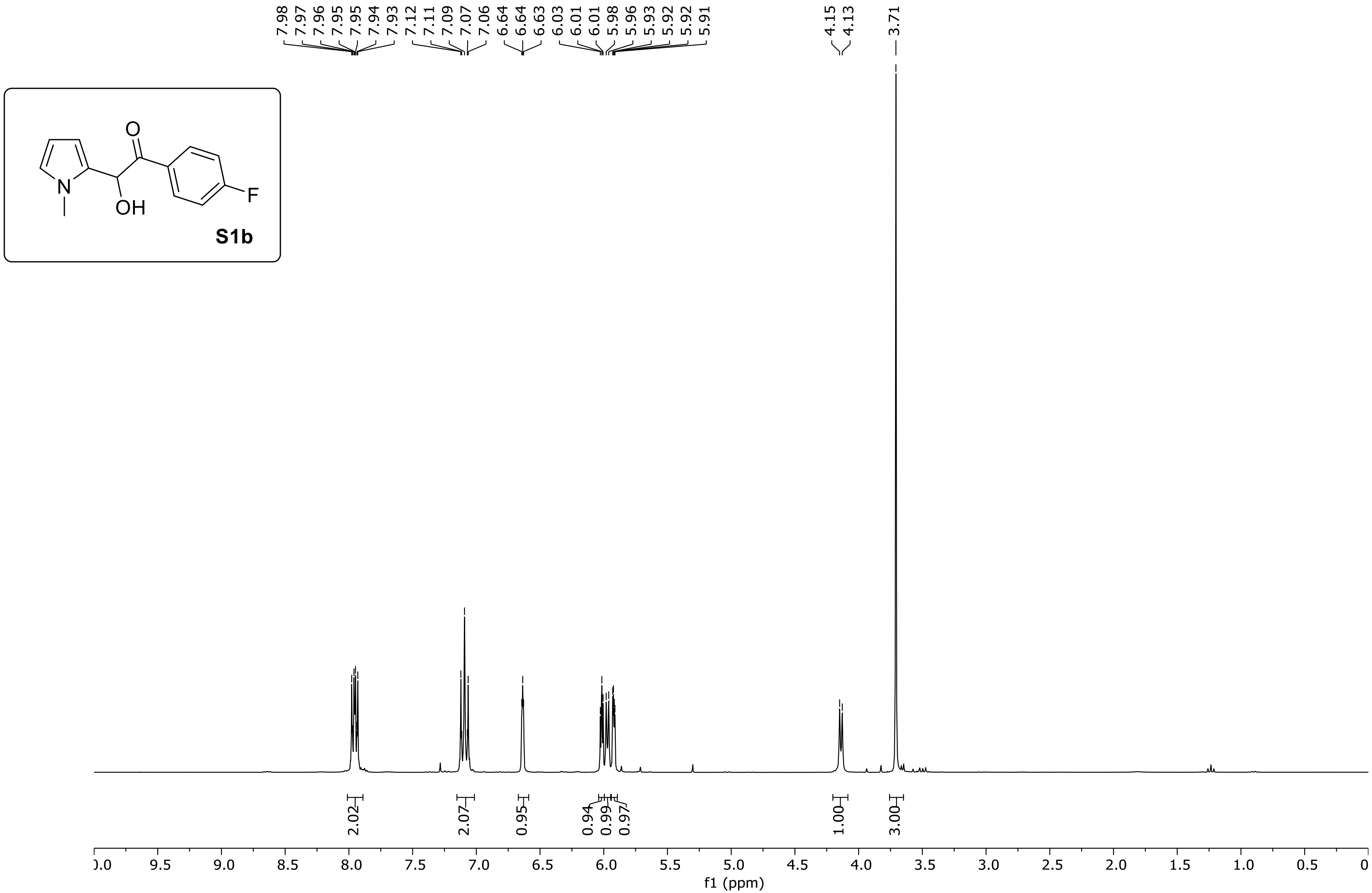

Figure S2: ^{13}C NMR of compound **S1b** in $CDCl_3$ at 75.4 MHz.

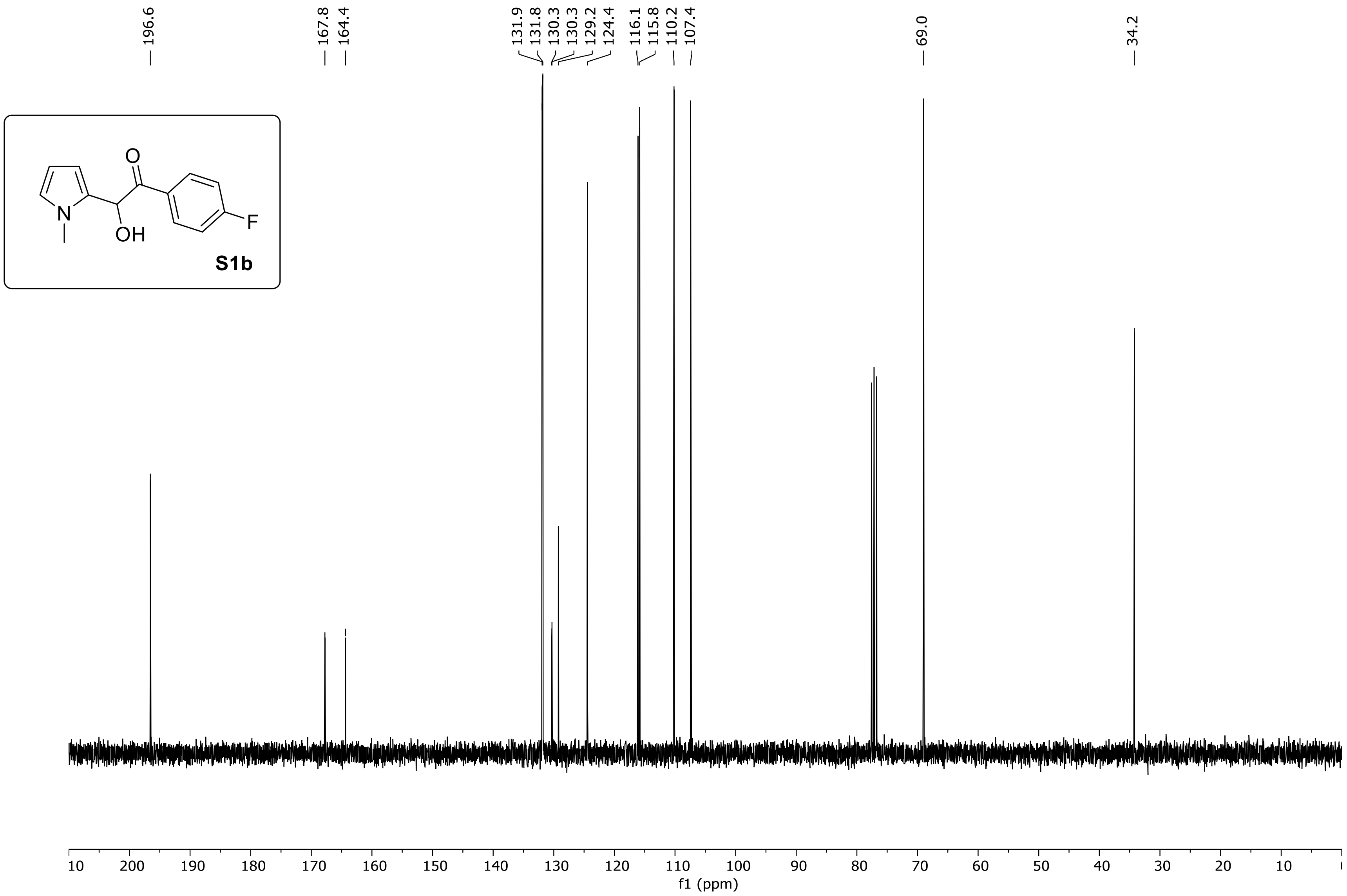

Figure S3: ^{1}H NMR of compound **S1c** in $CDCl_3$ at 300 MHz.

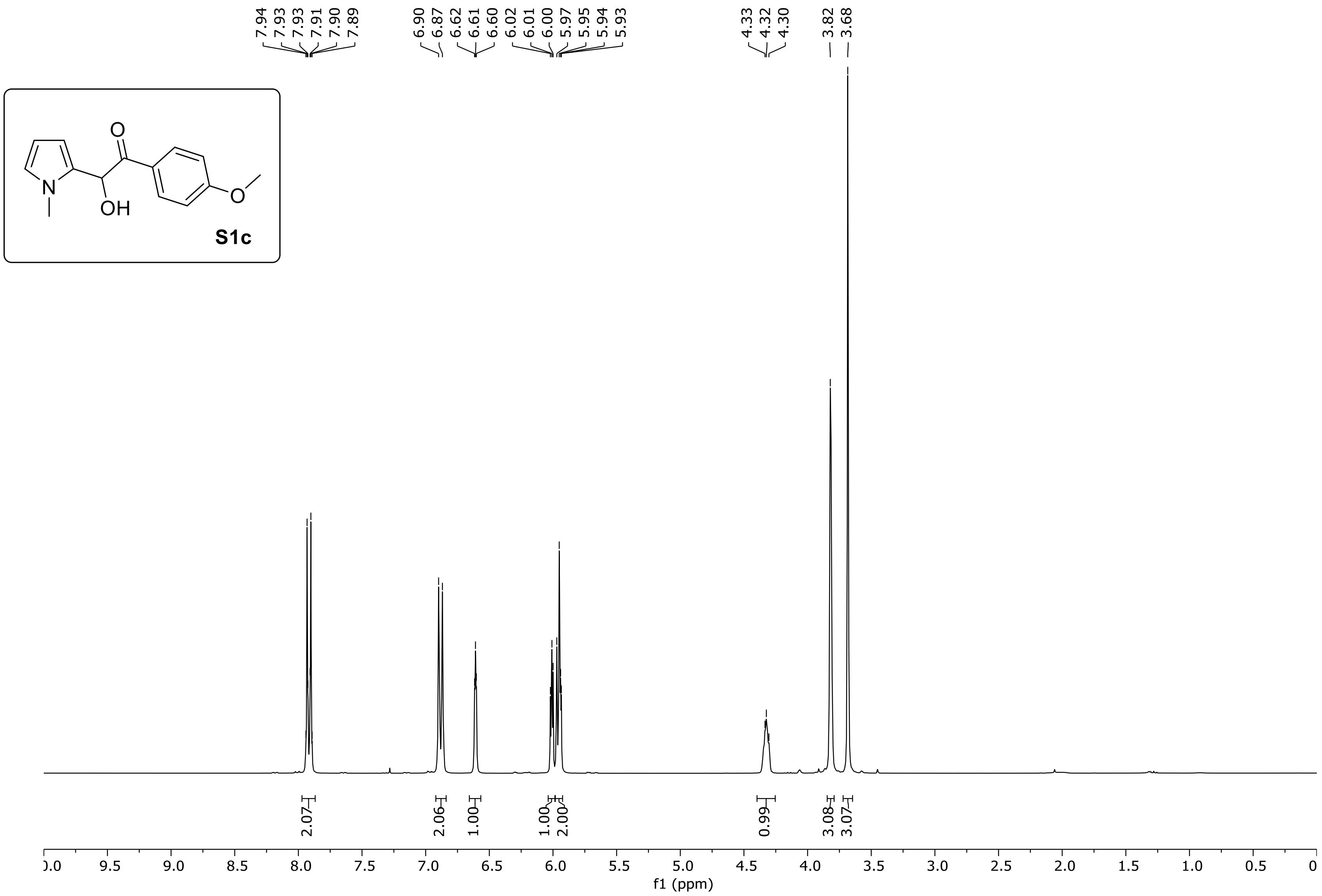

Figure S4: ^{13}C NMR of compound **S1c** in $CDCl_3$ at 75.4 MHz.

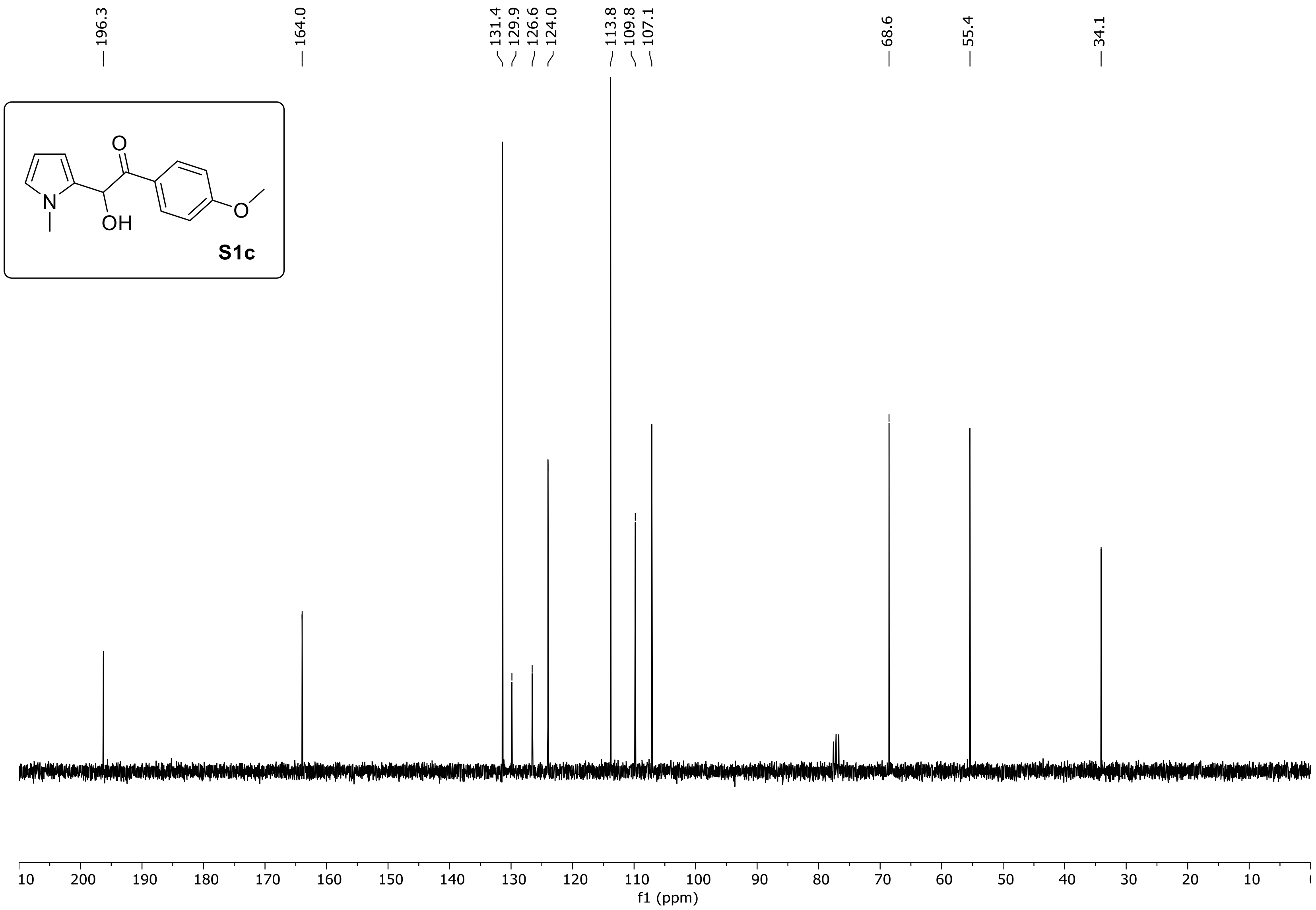

Figure S5: ^{1}H NMR of compound **S1d** in $CDCl_3$ at 300 MHz.

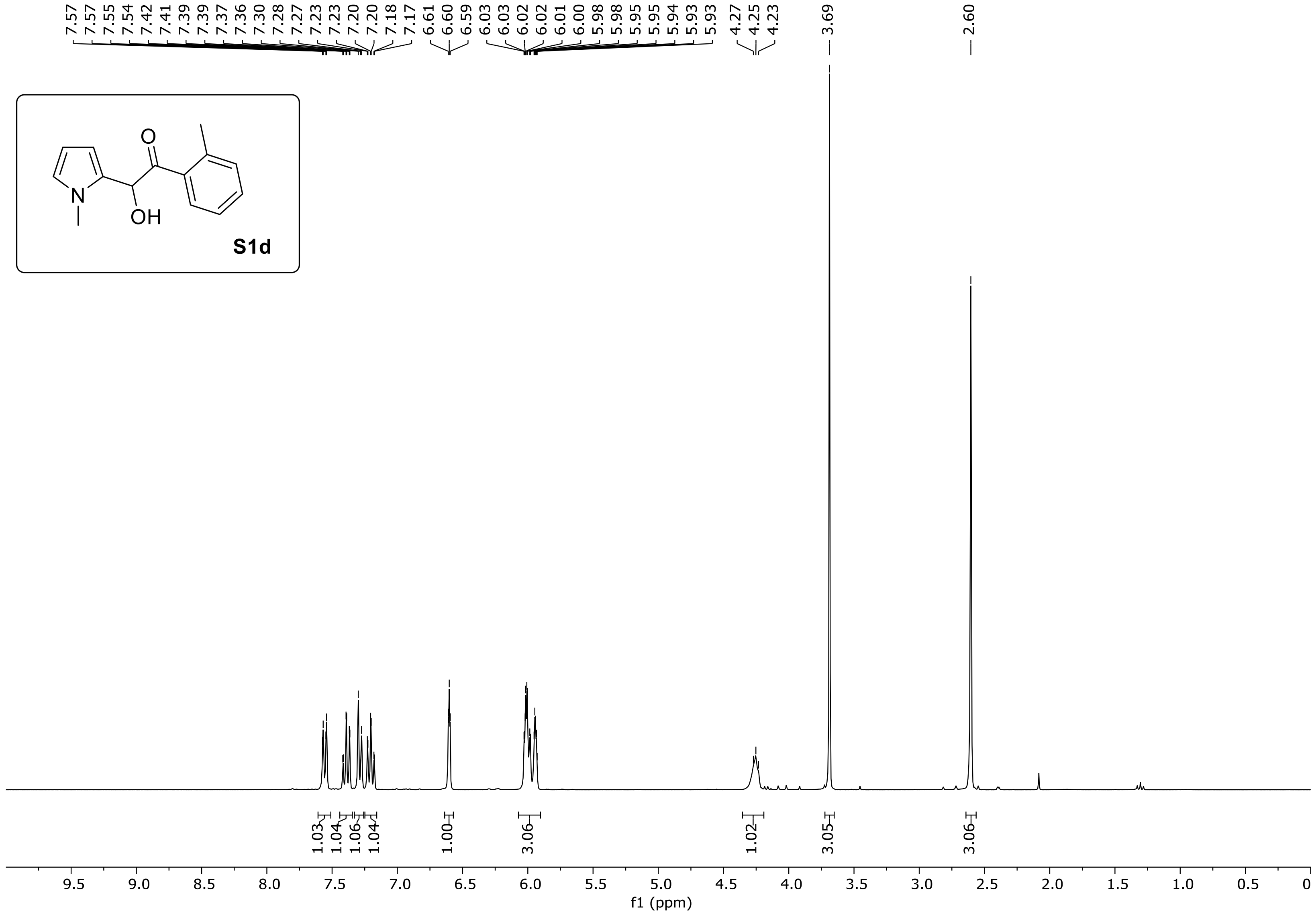

Figure S6: ^{13}C NMR of compound **S1d** in $CDCl_3$ at 75.4 MHz.

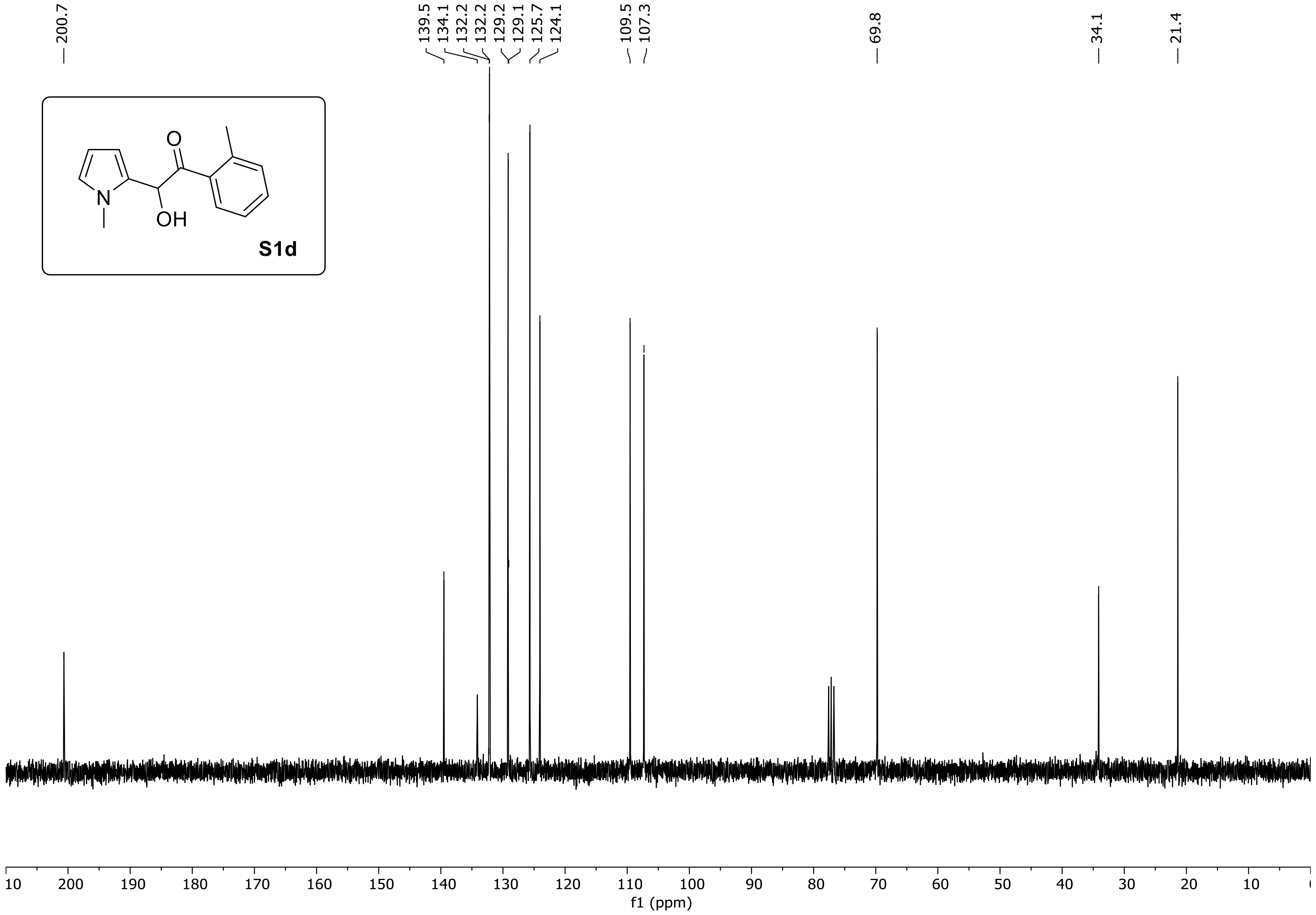

Figure S7: ^{1}H NMR of compound **S1e** in $CDCl_3$ at 300 MHz.

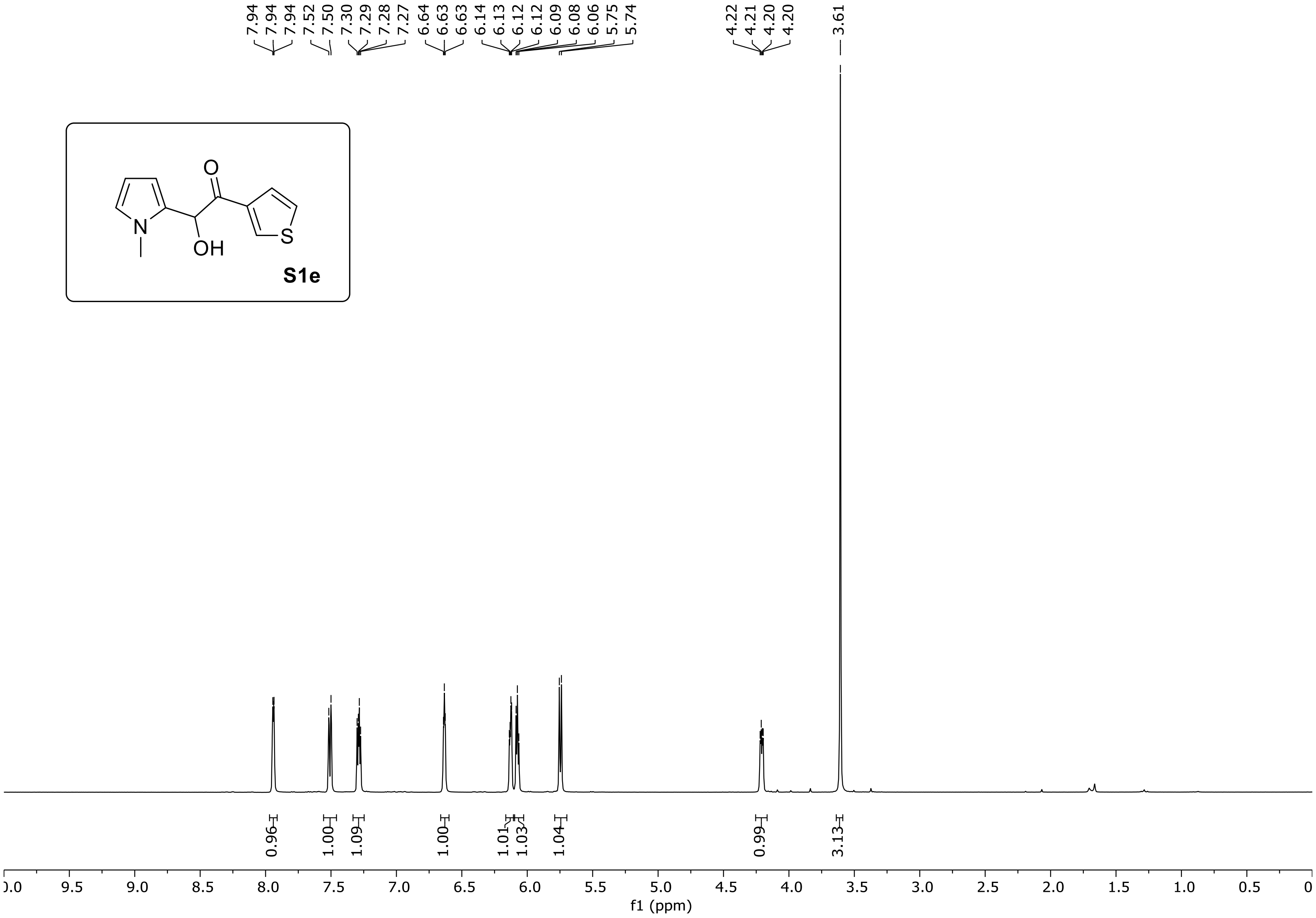

Figure S8: ^{13}C NMR of compound **S1e** in $CDCl_3$ at 75.4 MHz.

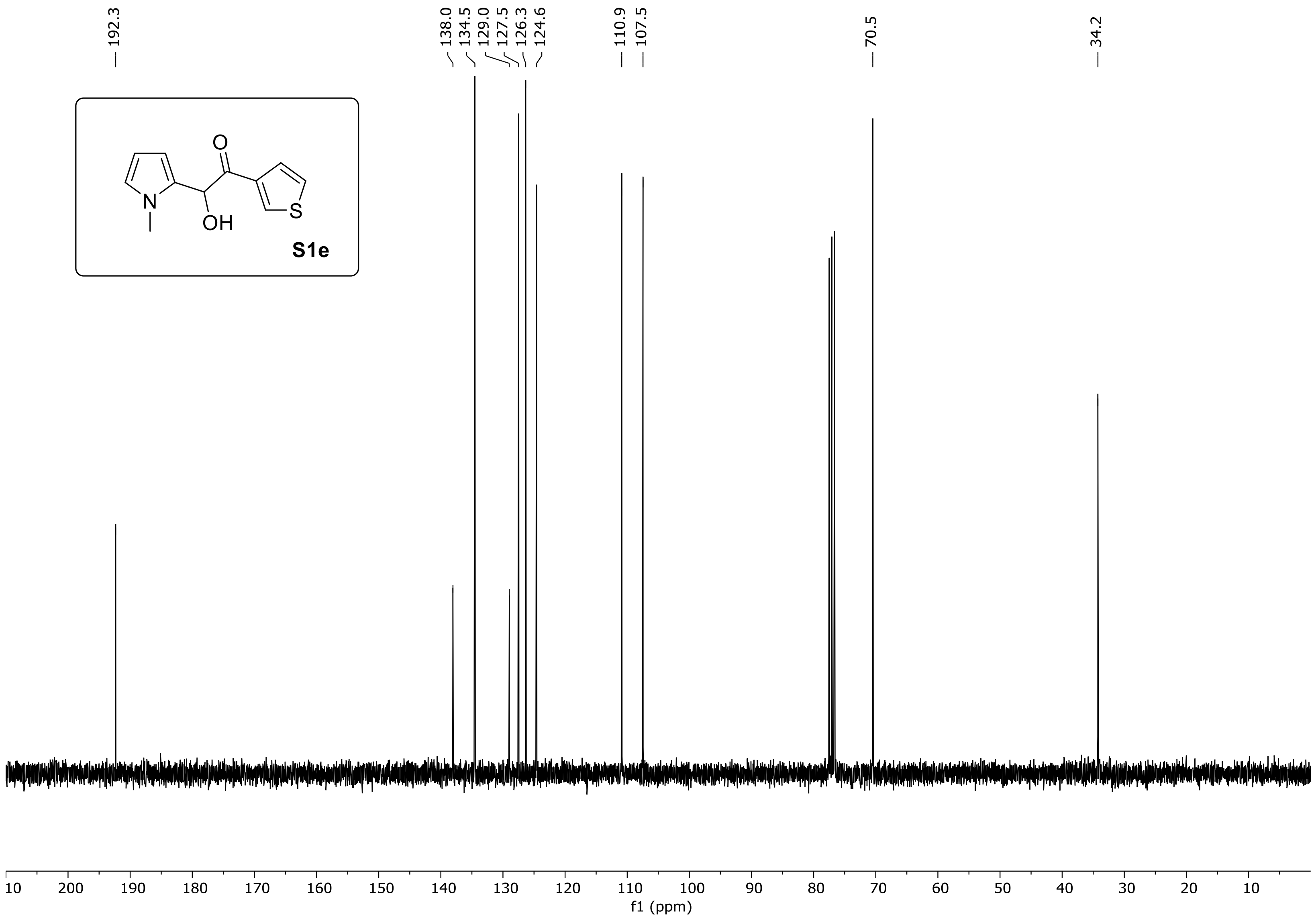

Figure S9: ^{1}H NMR of compound **S1f** in $CDCl_3$ at 300 MHz.

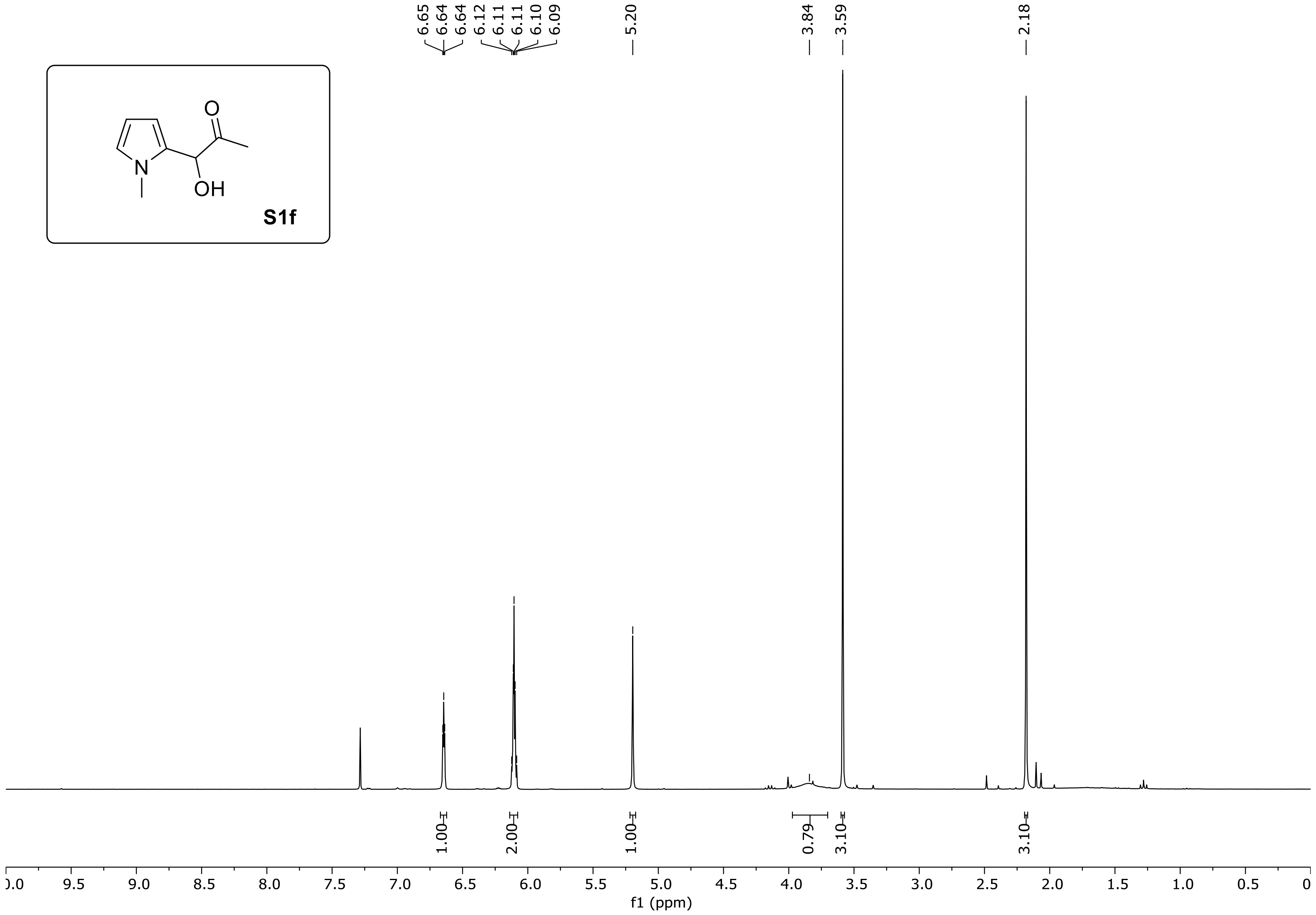

Figure S10: ^{13}C NMR of compound **S1f** in $CDCl_3$ at 75.4 MHz.

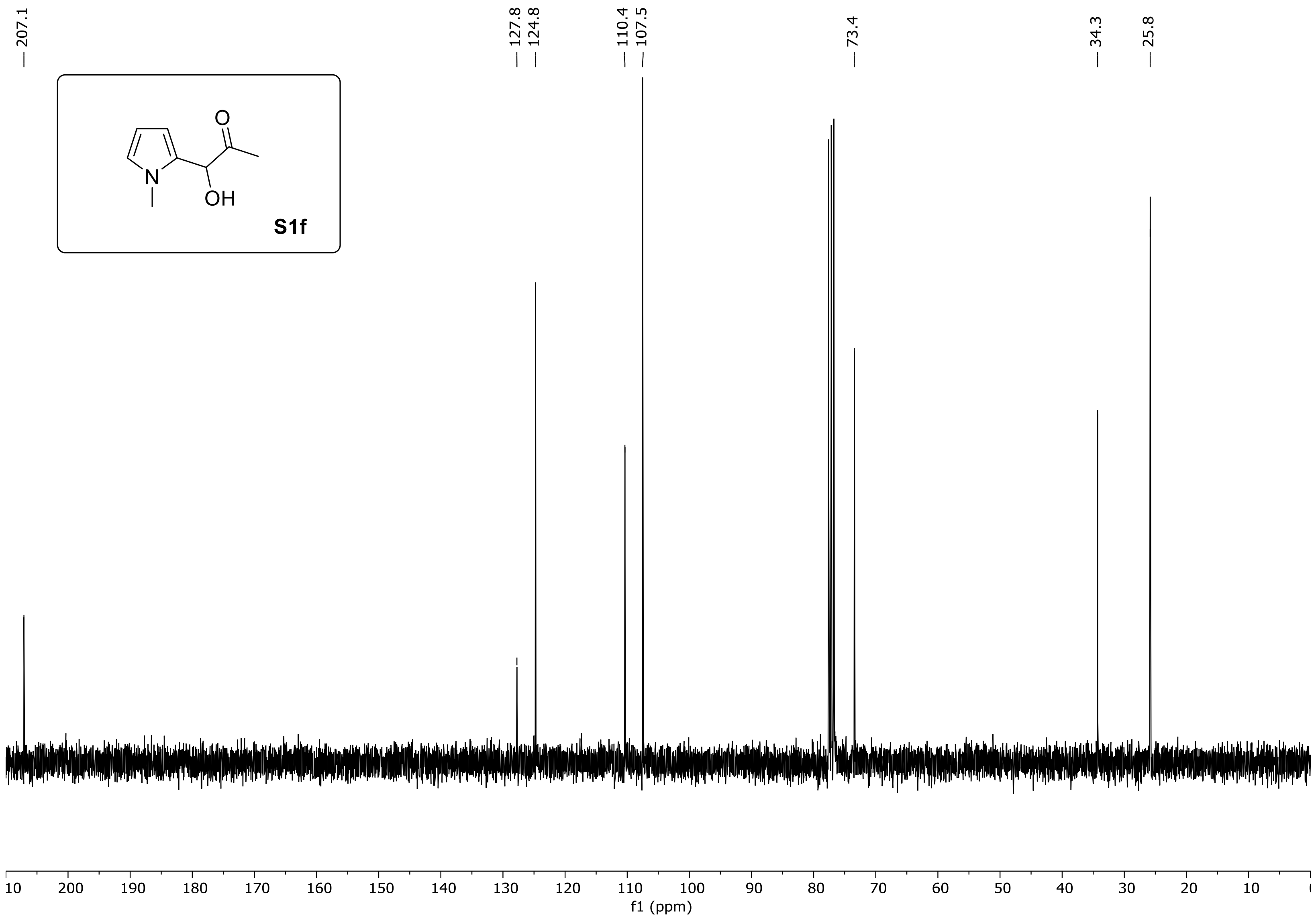

Figure S11: ^{1}H NMR of compound **S1g** in $CDCl_3$ at 300 MHz.

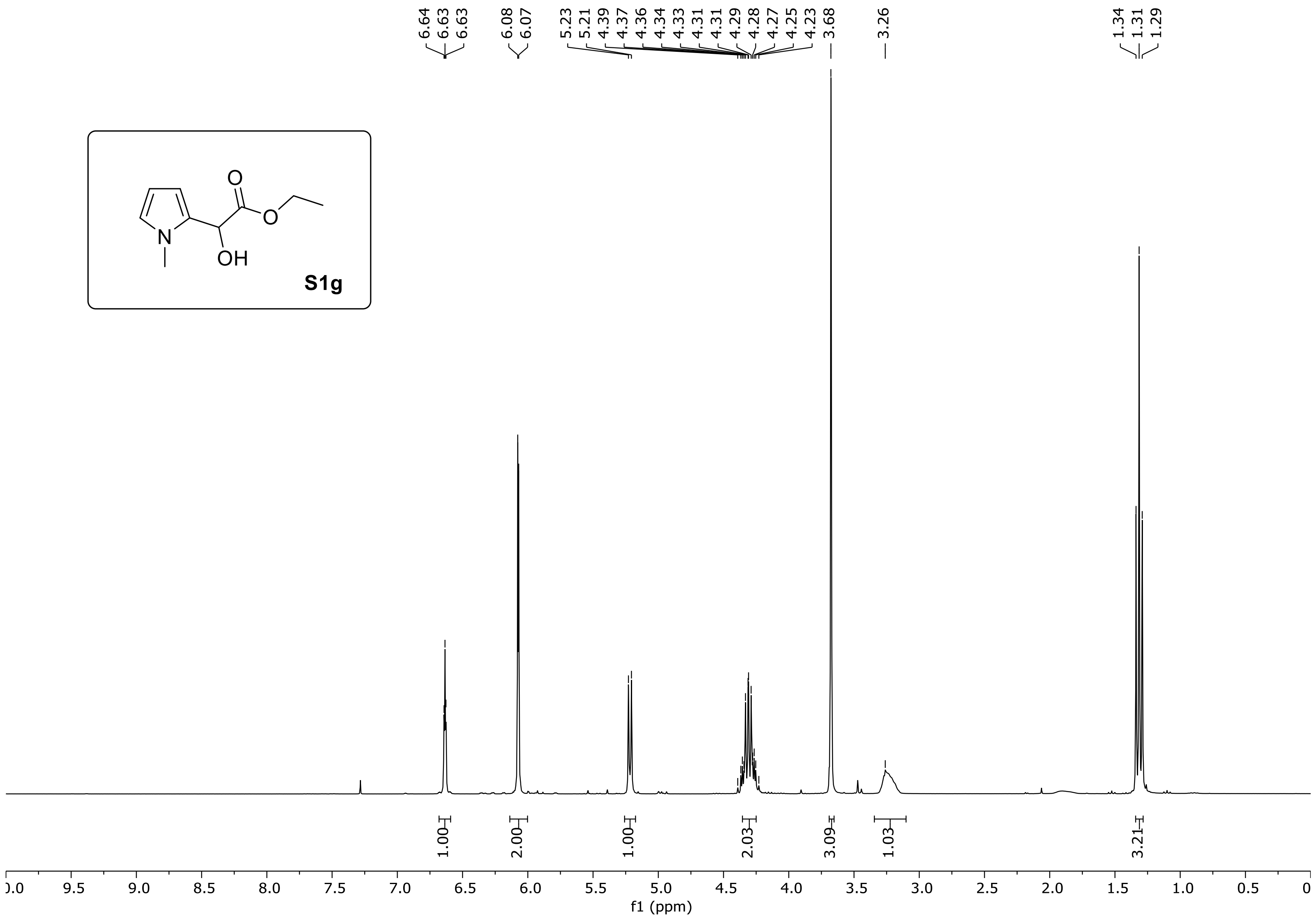

Figure S12: ^{13}C NMR of compound **S1g** in $CDCl_3$ at 75.4 MHz.

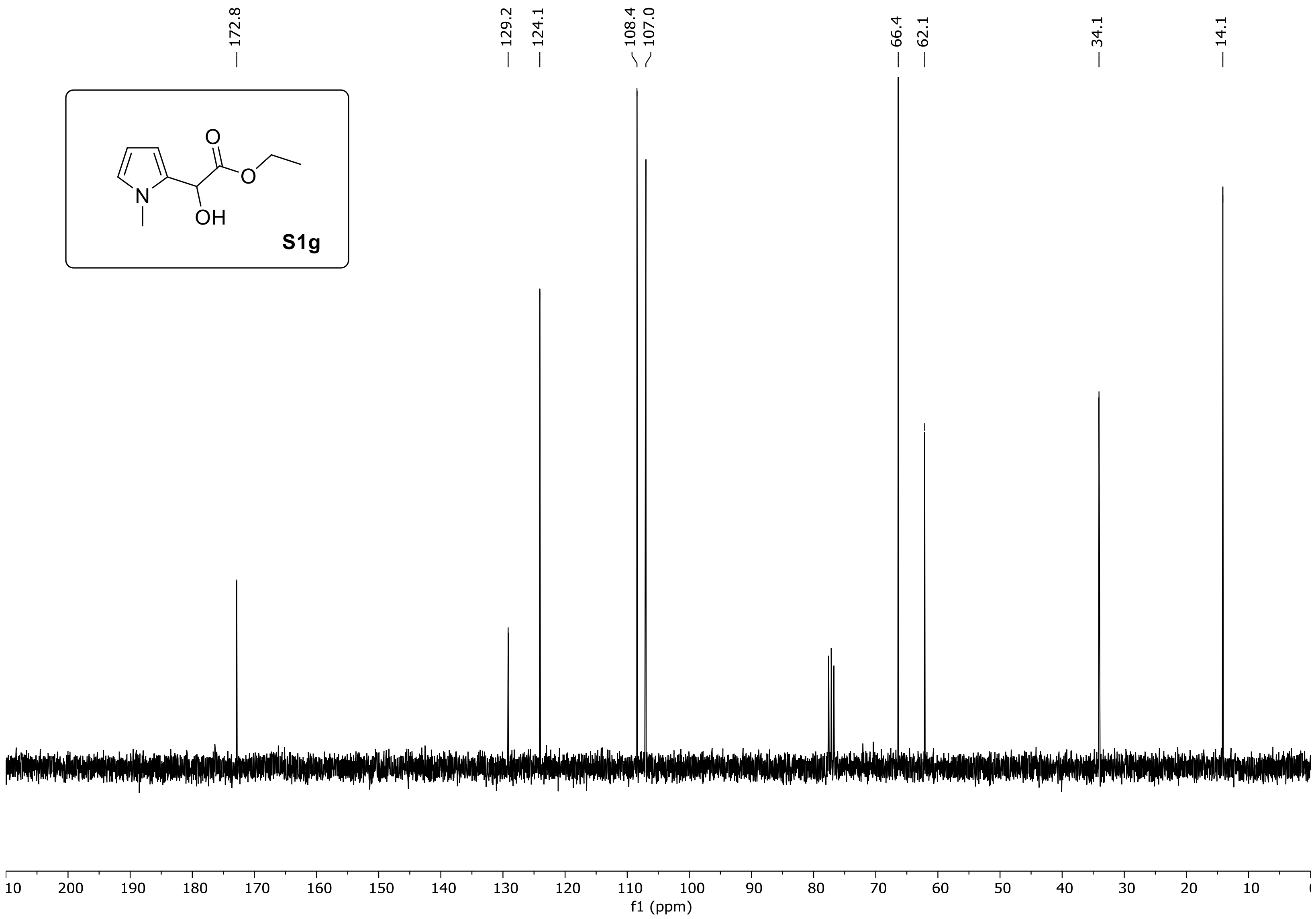

Figure S13: ^{1}H NMR of compound **S1j** in $CDCl_3$ at 300 MHz.

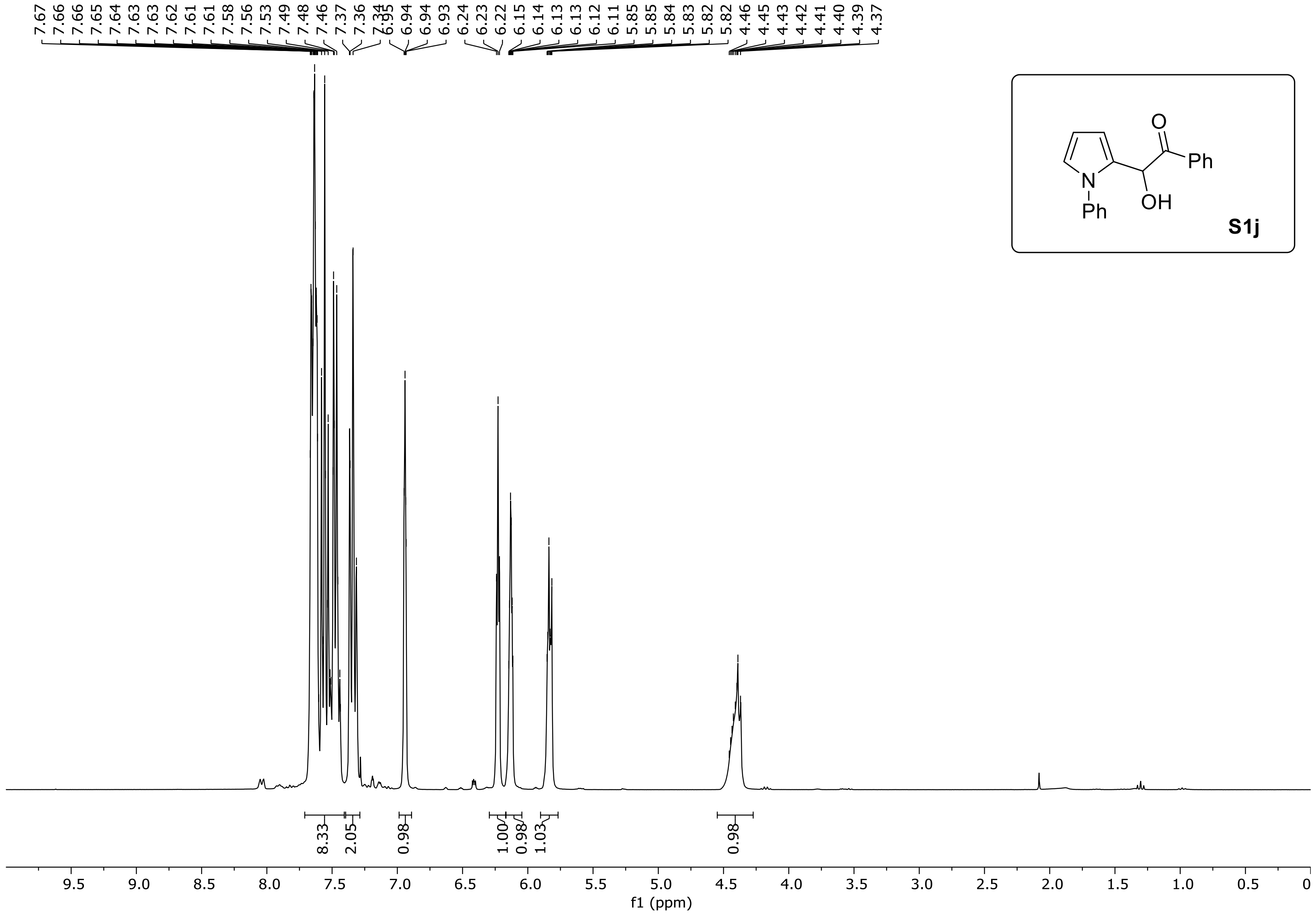

Figure S14: ^{13}C NMR of compound **S1j** in $CDCl_3$ at 75.4 MHz.

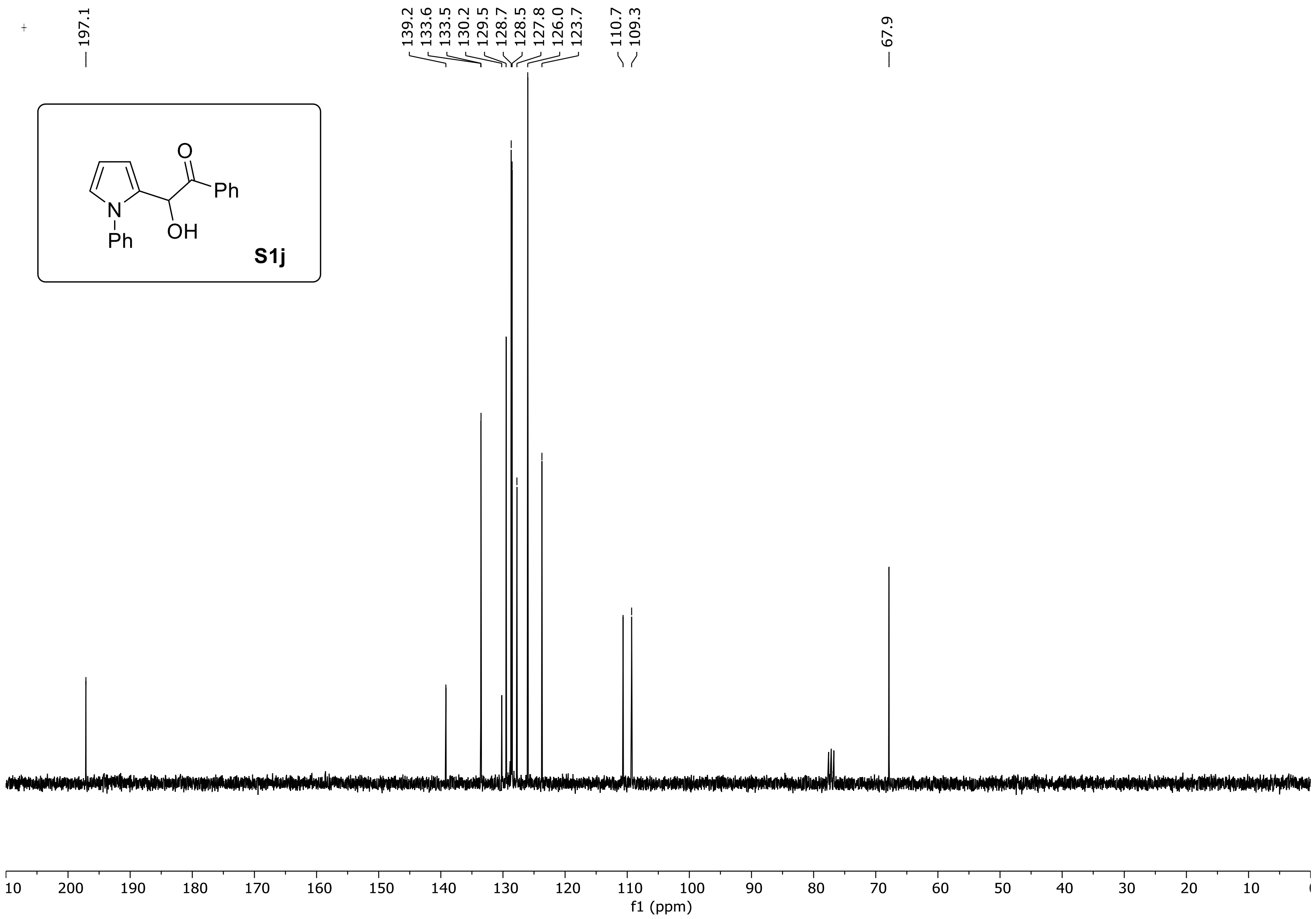

Figure S15: ^{1}H NMR of compound **S2** in $CDCl_3$ at 300 MHz.

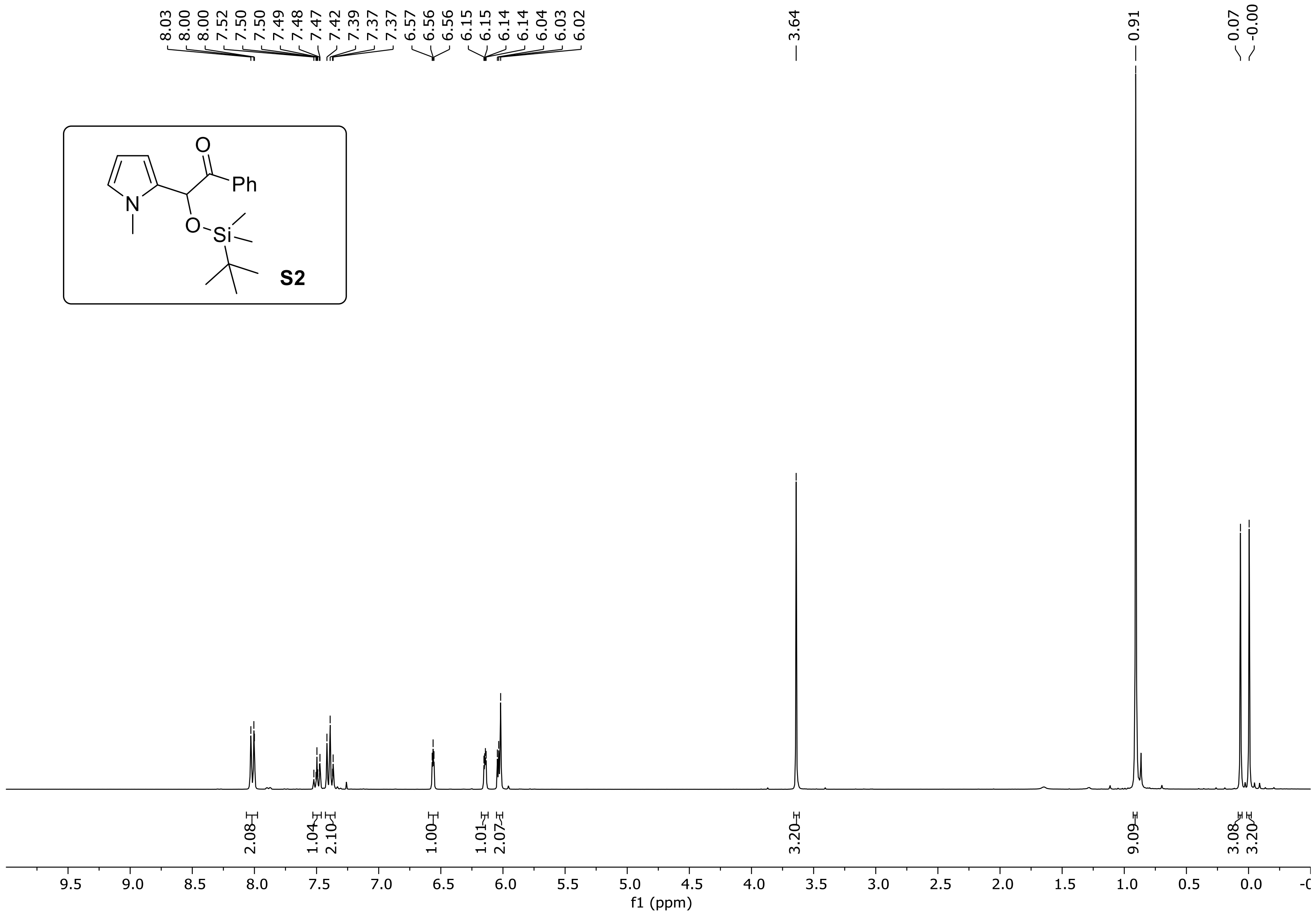

Figure S16: ^{13}C NMR of compound **S2** in $CDCl_3$ at 75.4 MHz.

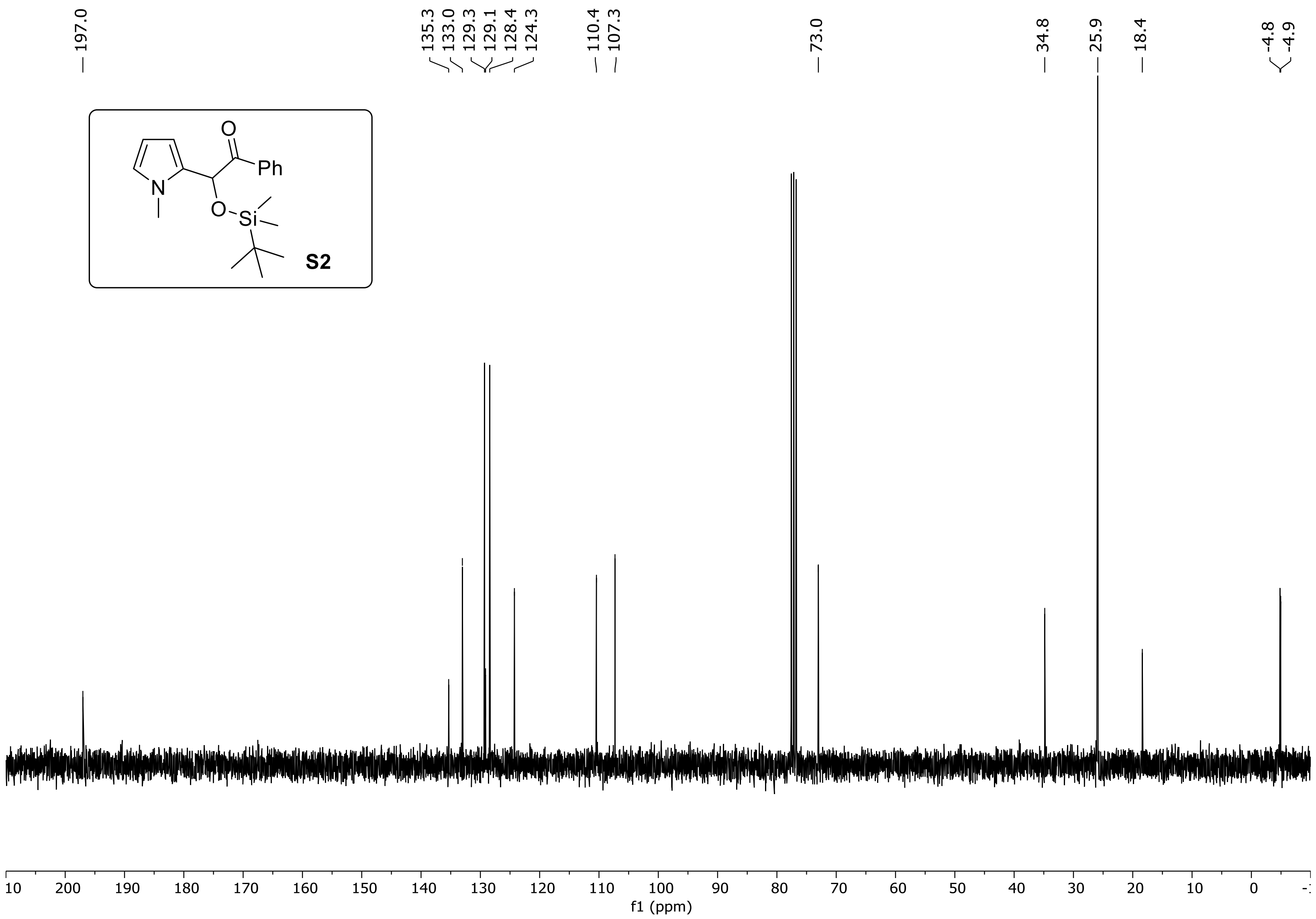

Figure S17: ^{1}H NMR of compound **S3** in $CDCl_3$ at 300 MHz.

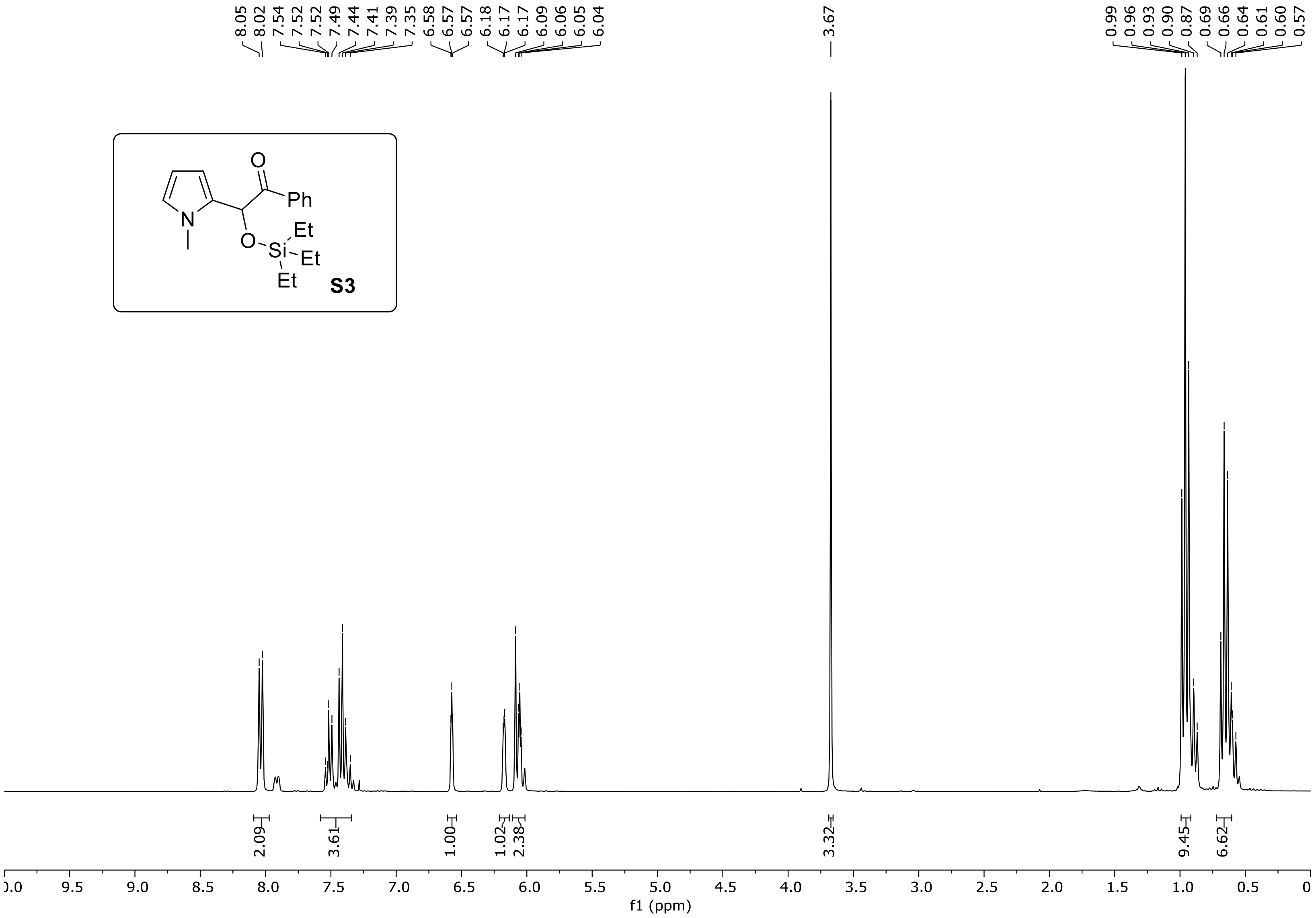

Figure S18: ^{13}C NMR of compound **S3** in $CDCl_3$ at 75.4 MHz.

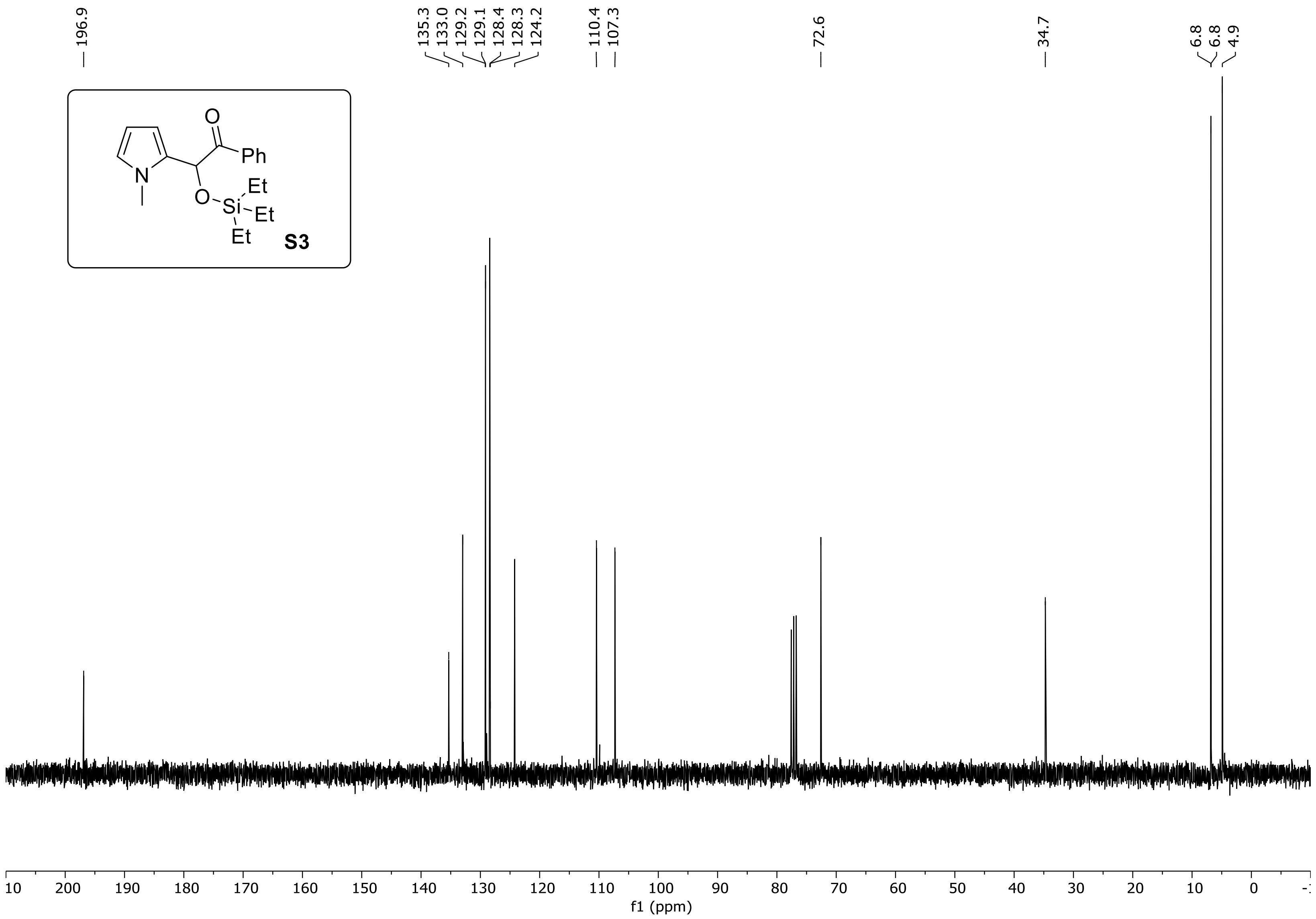

Figure S19: ^{1}H NMR of compound **S4** in $CDCl_3$ at 300 MHz.

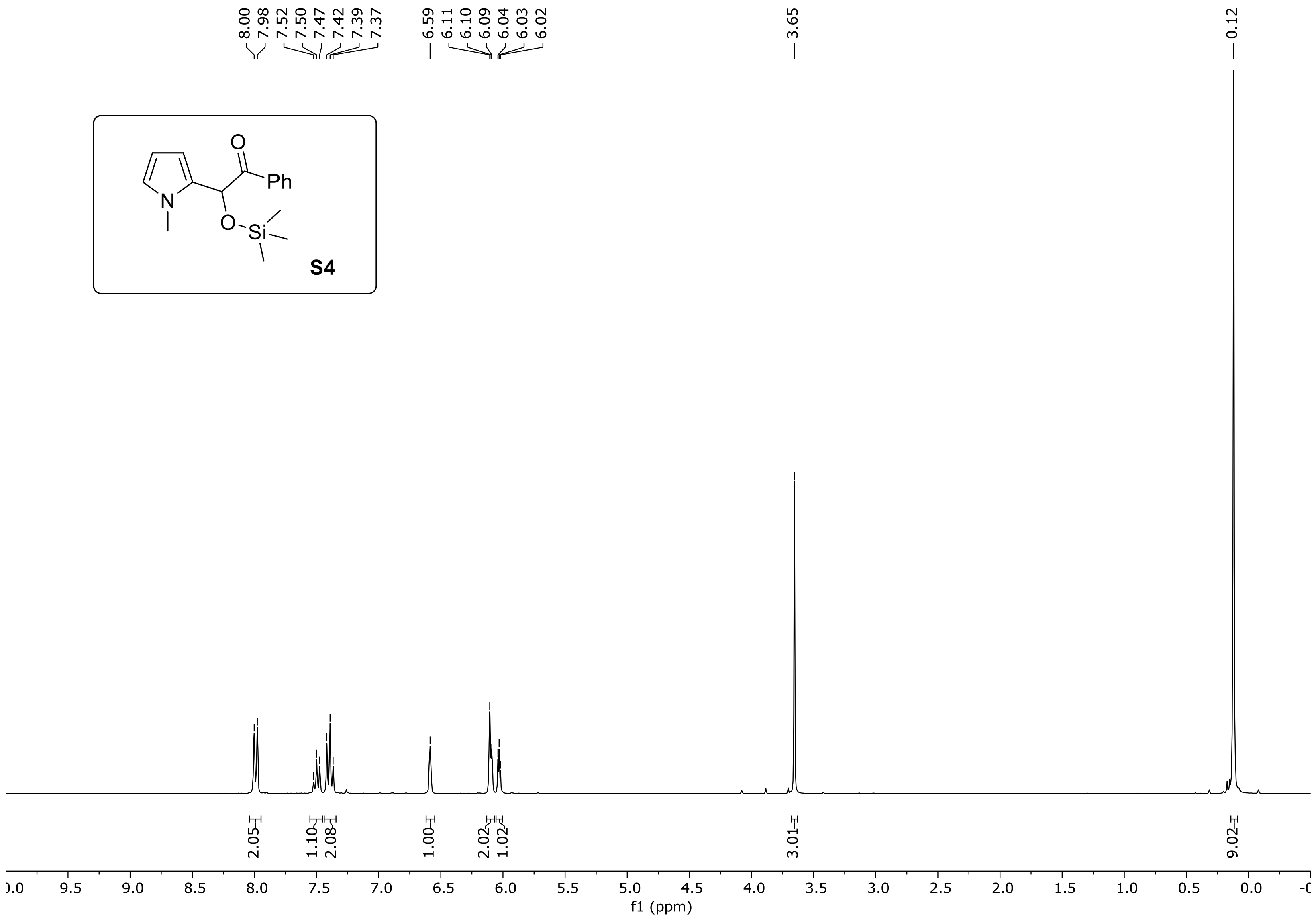

Figure S20: ^{13}C NMR of compound **S4** in $CDCl_3$ at 75.4 MHz.

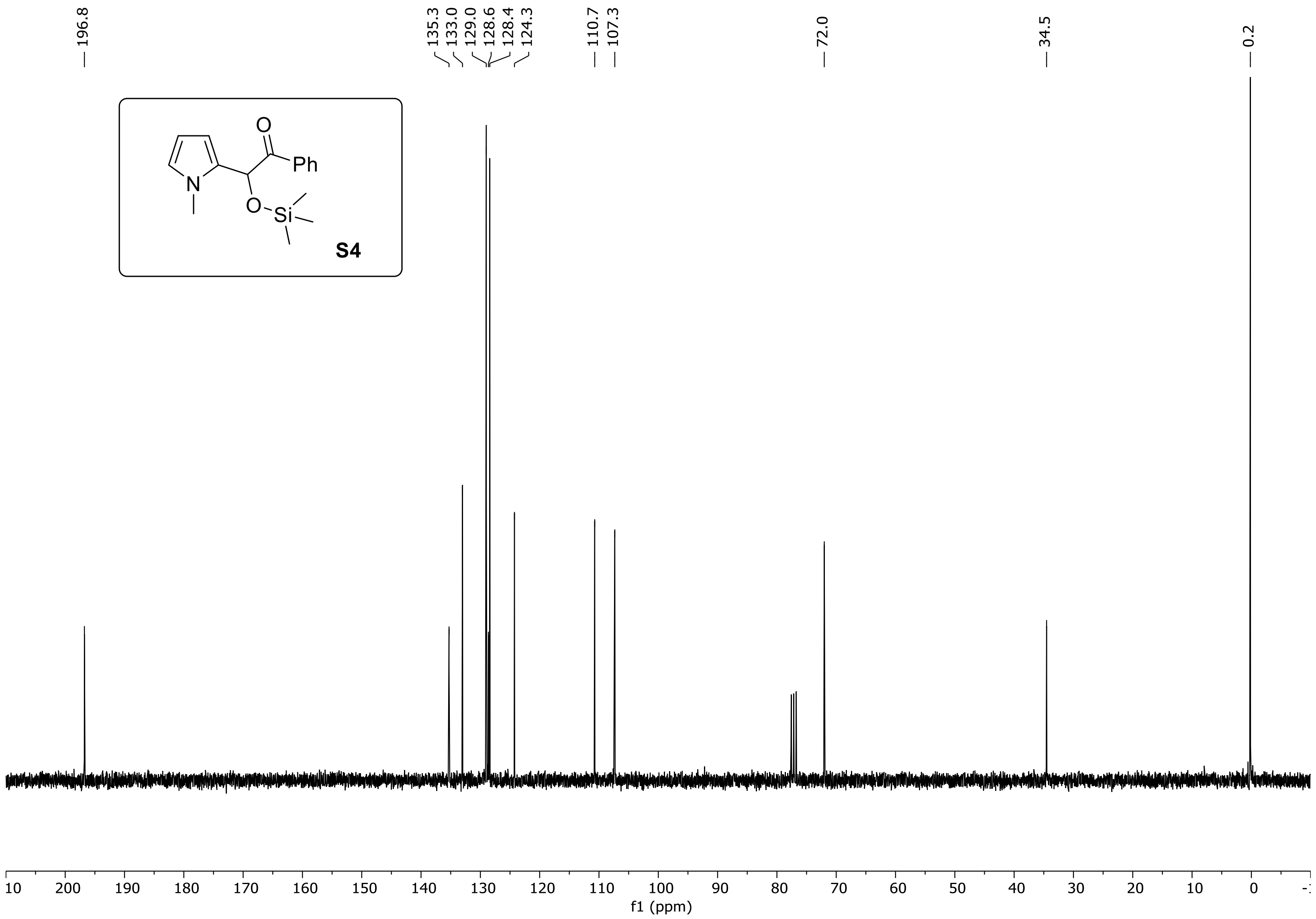

Figure S21: ^{1}H NMR of compound **S2b** in $CDCl_3$ at 300 MHz.

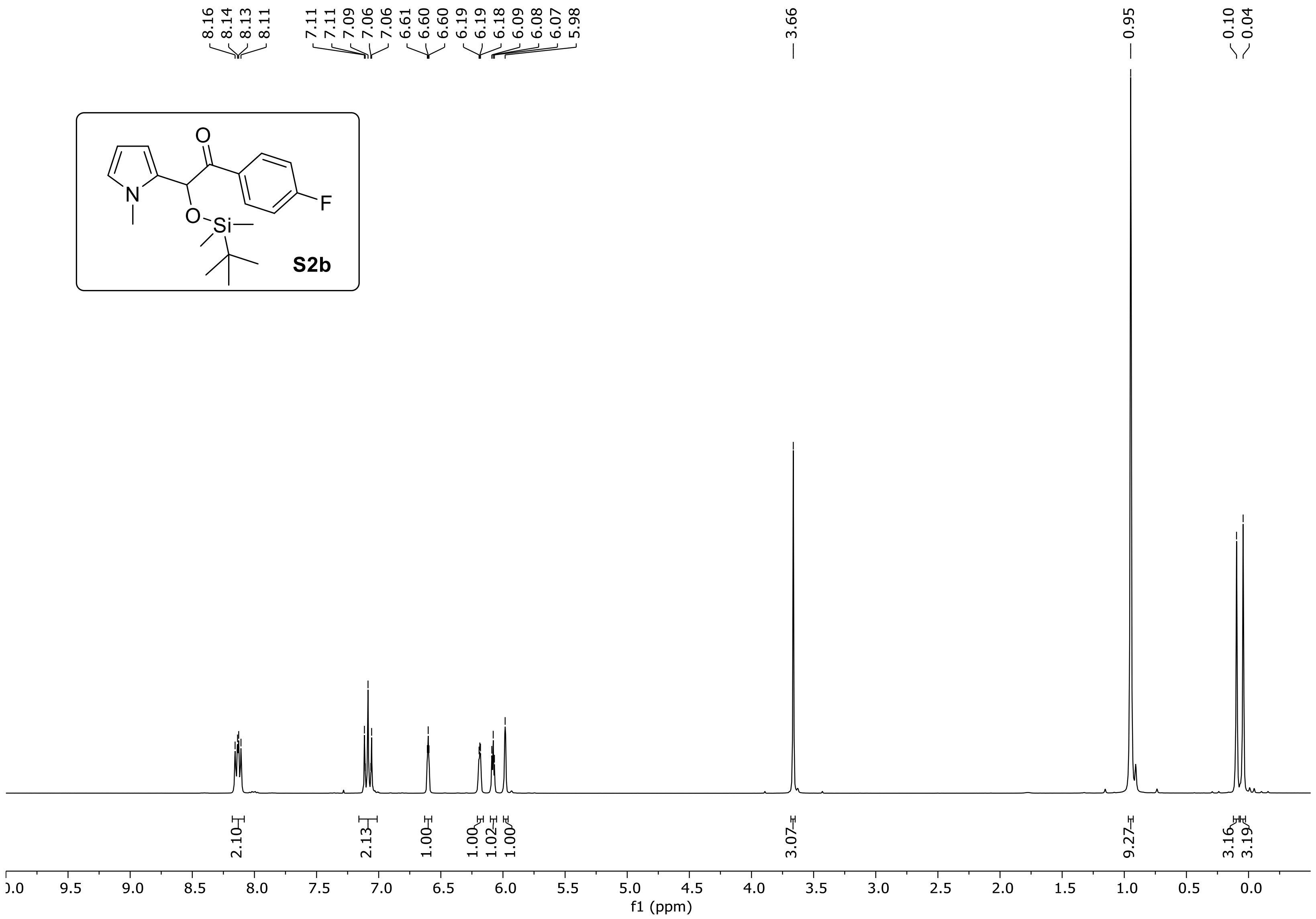

Figure S22: ^{13}C NMR of compound **S2b** in $CDCl_3$ at 75.4 MHz.

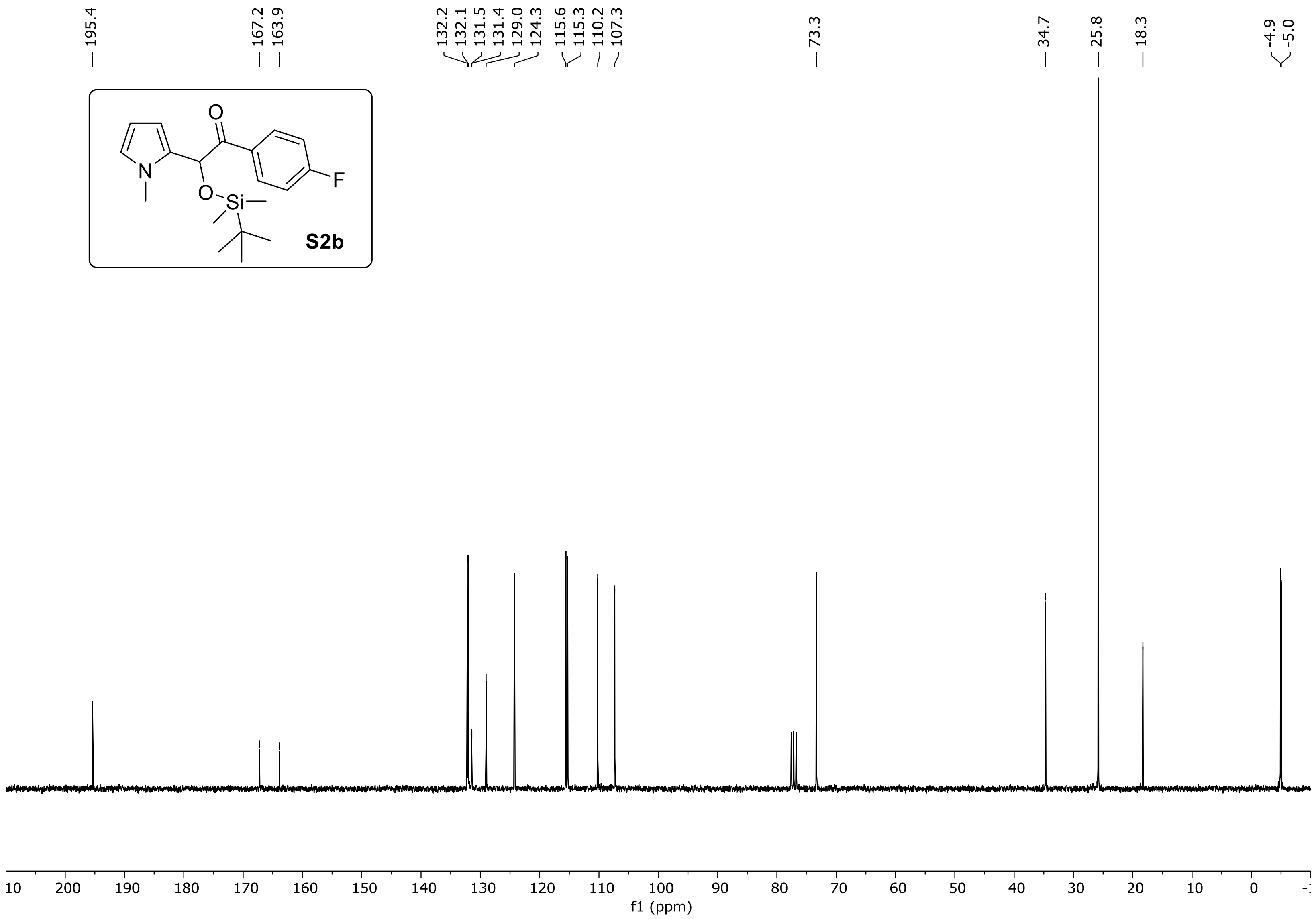

Figure S23: ^{1}H NMR of compound **S2c** in $CDCl_3$ at 300 MHz.

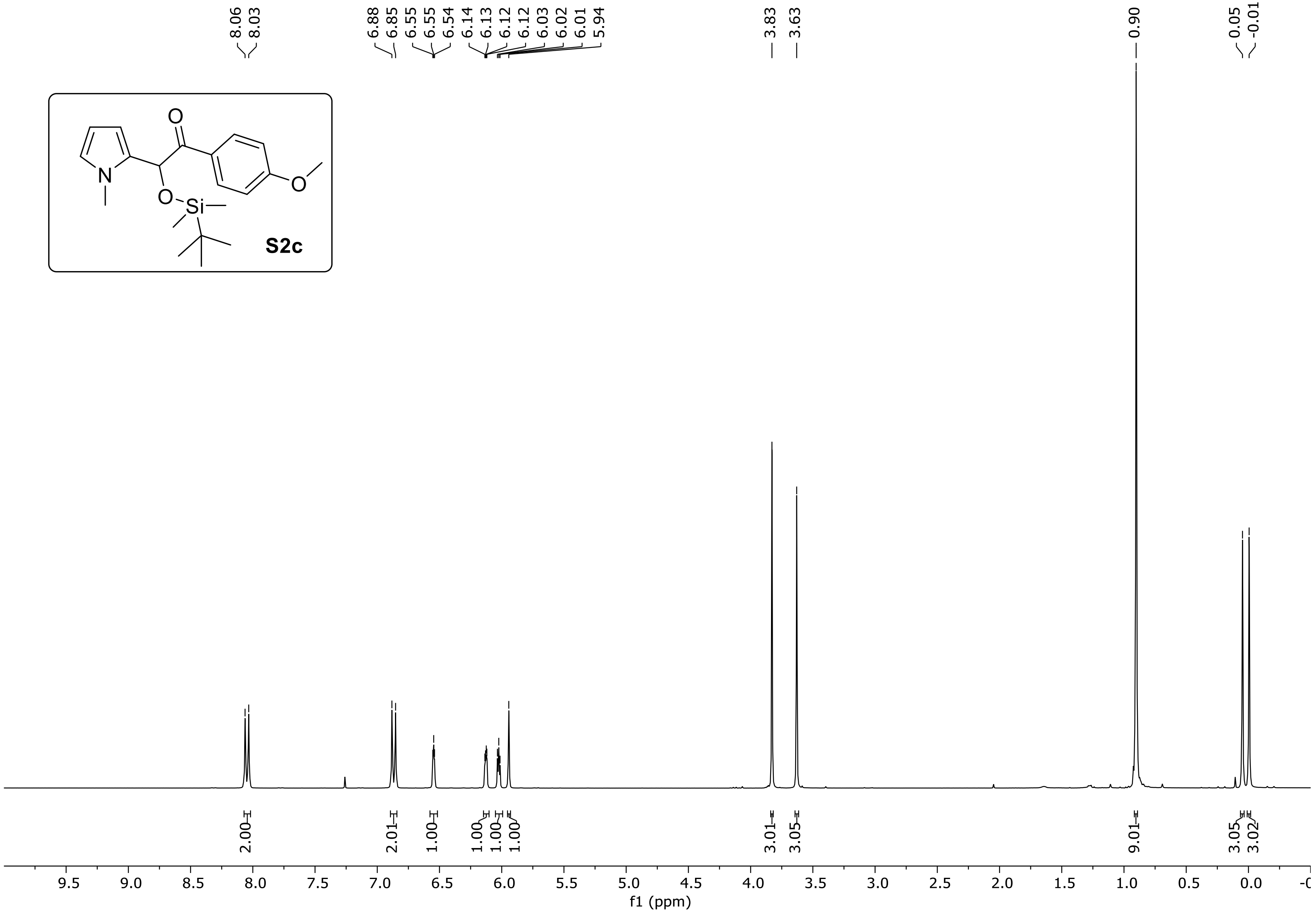

Figure S24: ^{13}C NMR of compound **S2c** in $CDCl_3$ at 75.4 MHz.

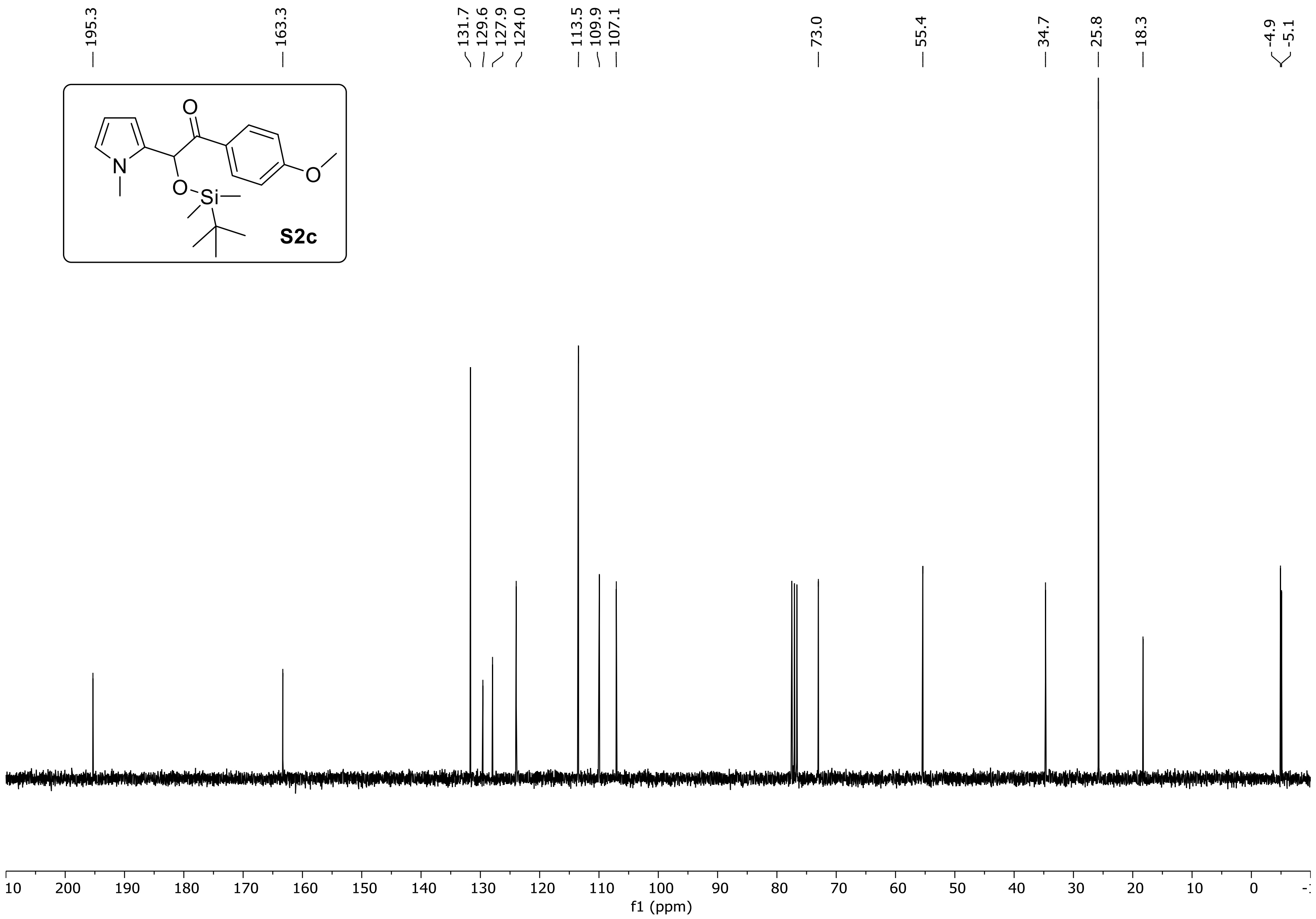

Figure S25: ^{1}H NMR of compound **S2d** in $CDCl_3$ at 300 MHz.

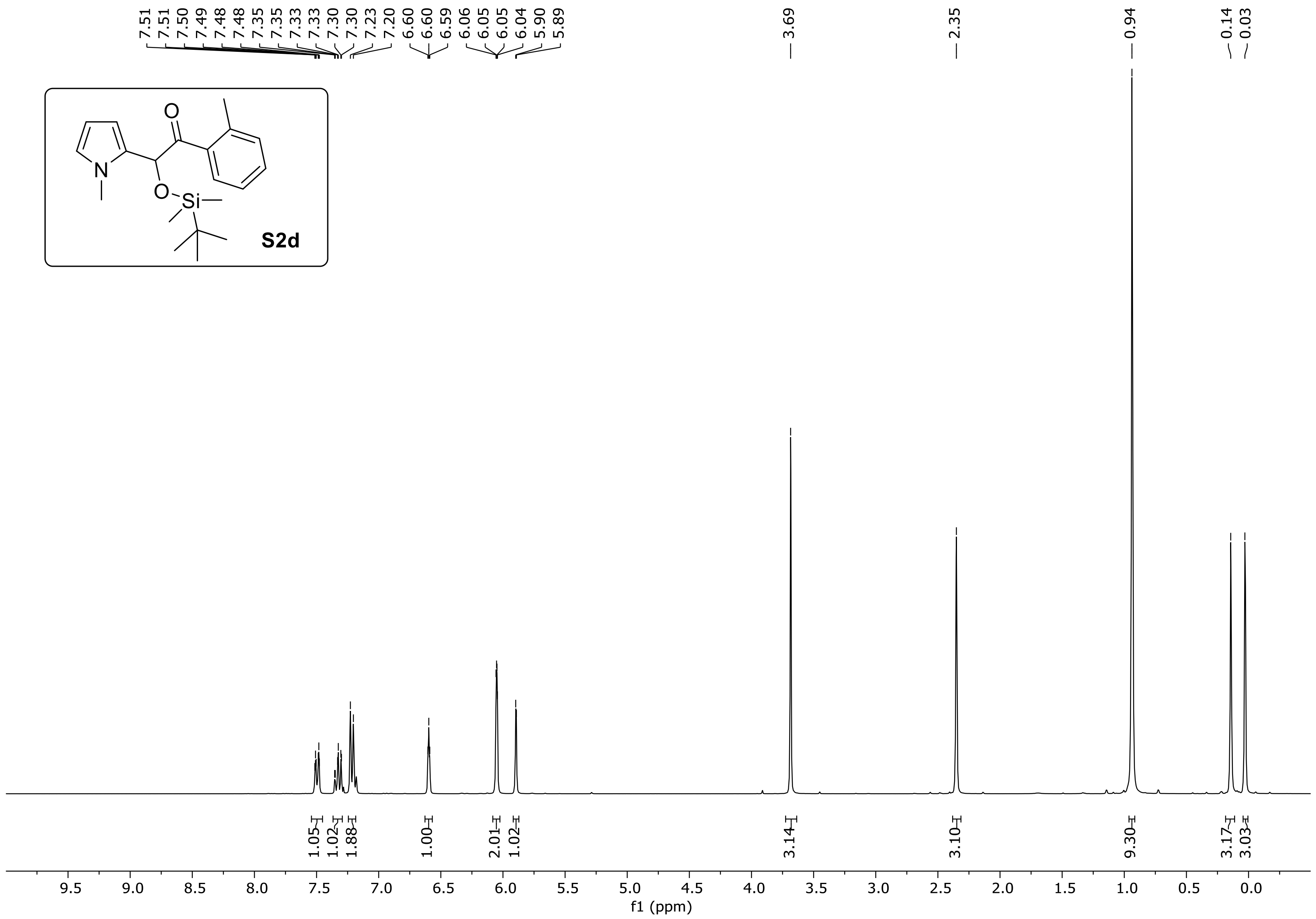

Figure S26: ^{13}C NMR of compound **S2d** in $CDCl_3$ at 75.4 MHz.

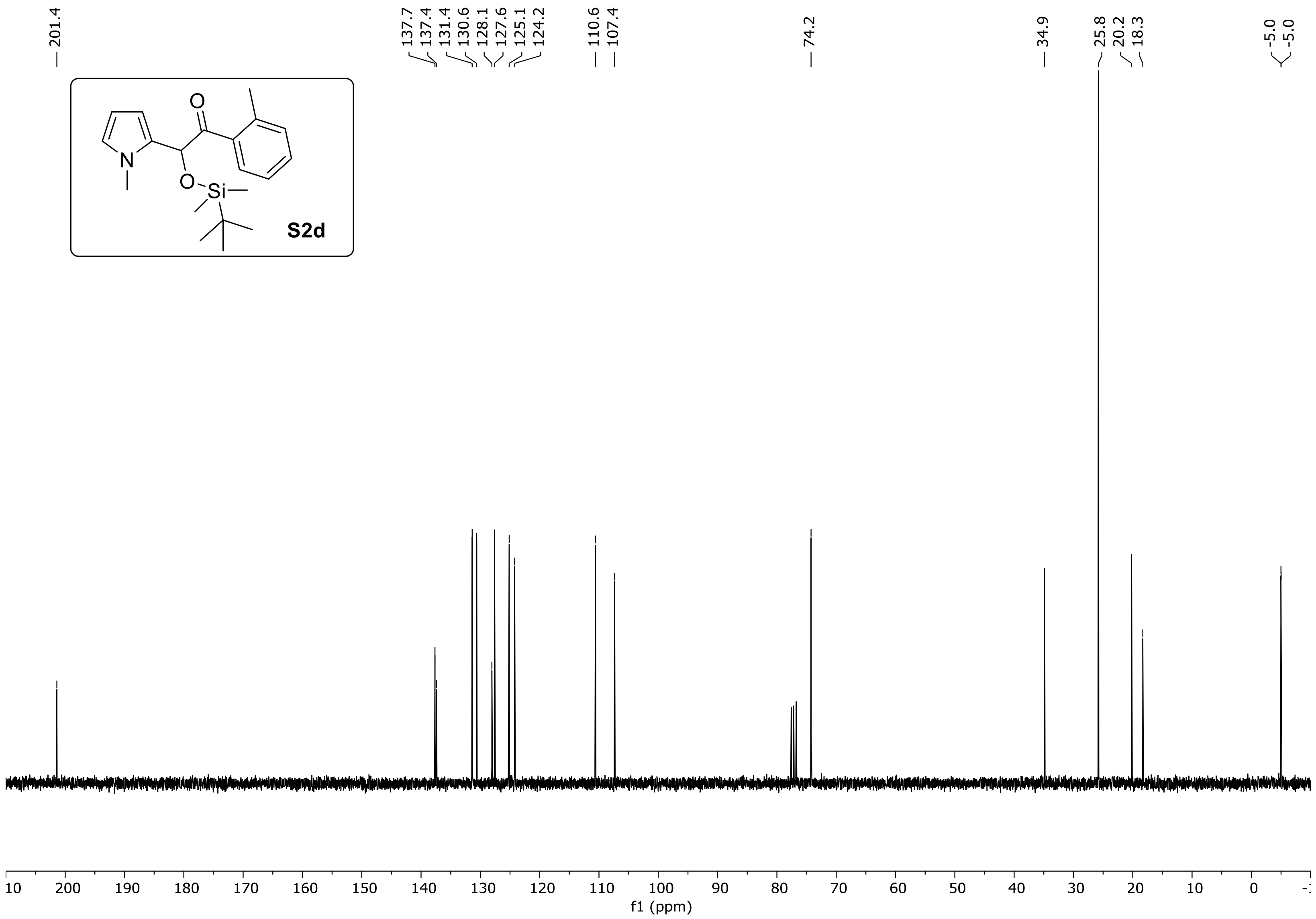

Figure S27: ^{1}H NMR of compound **S2e** in $CDCl_3$ at 300 MHz.

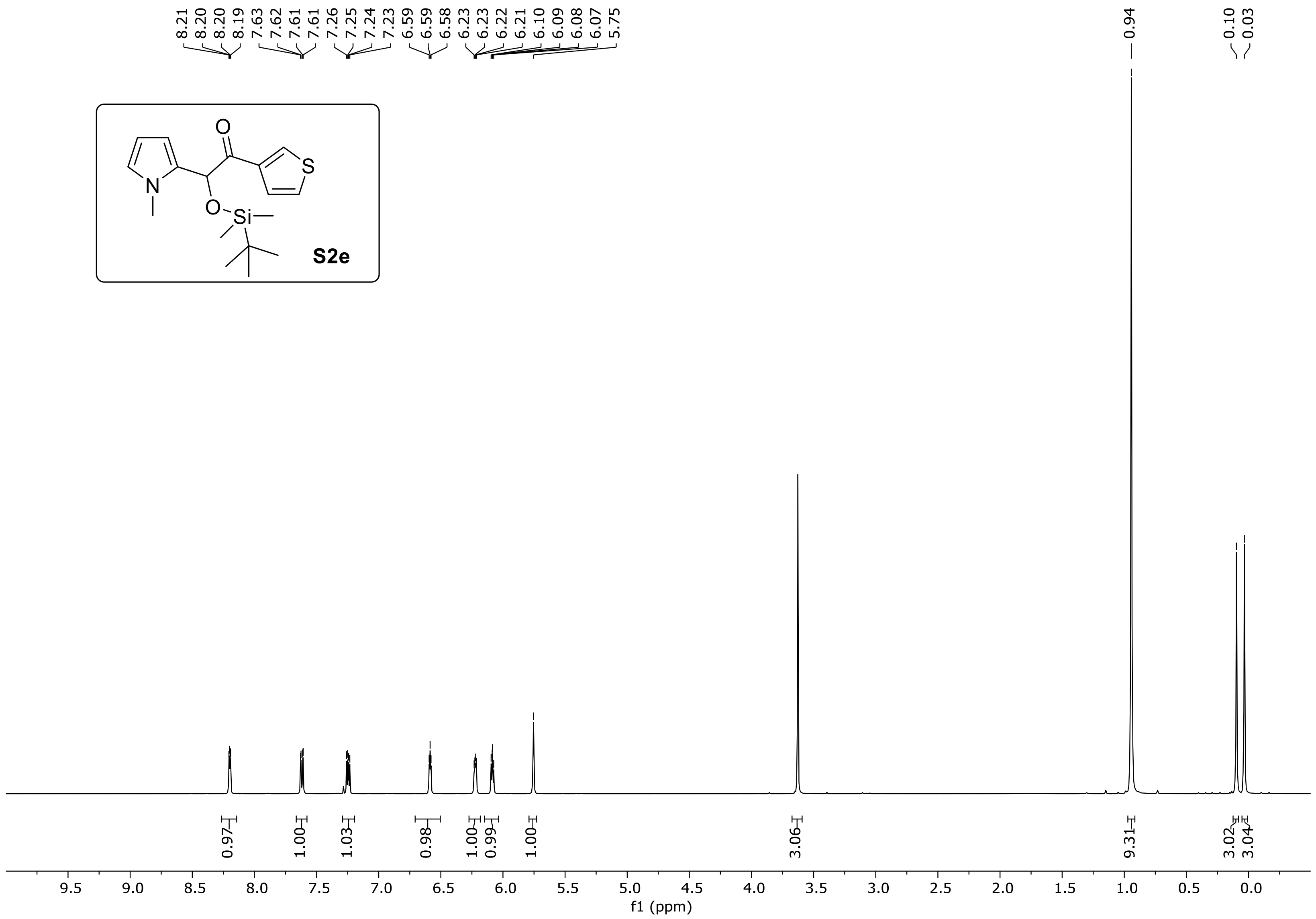

Figure S28: ^{13}C NMR of compound **S2e** in $CDCl_3$ at 75.4 MHz.

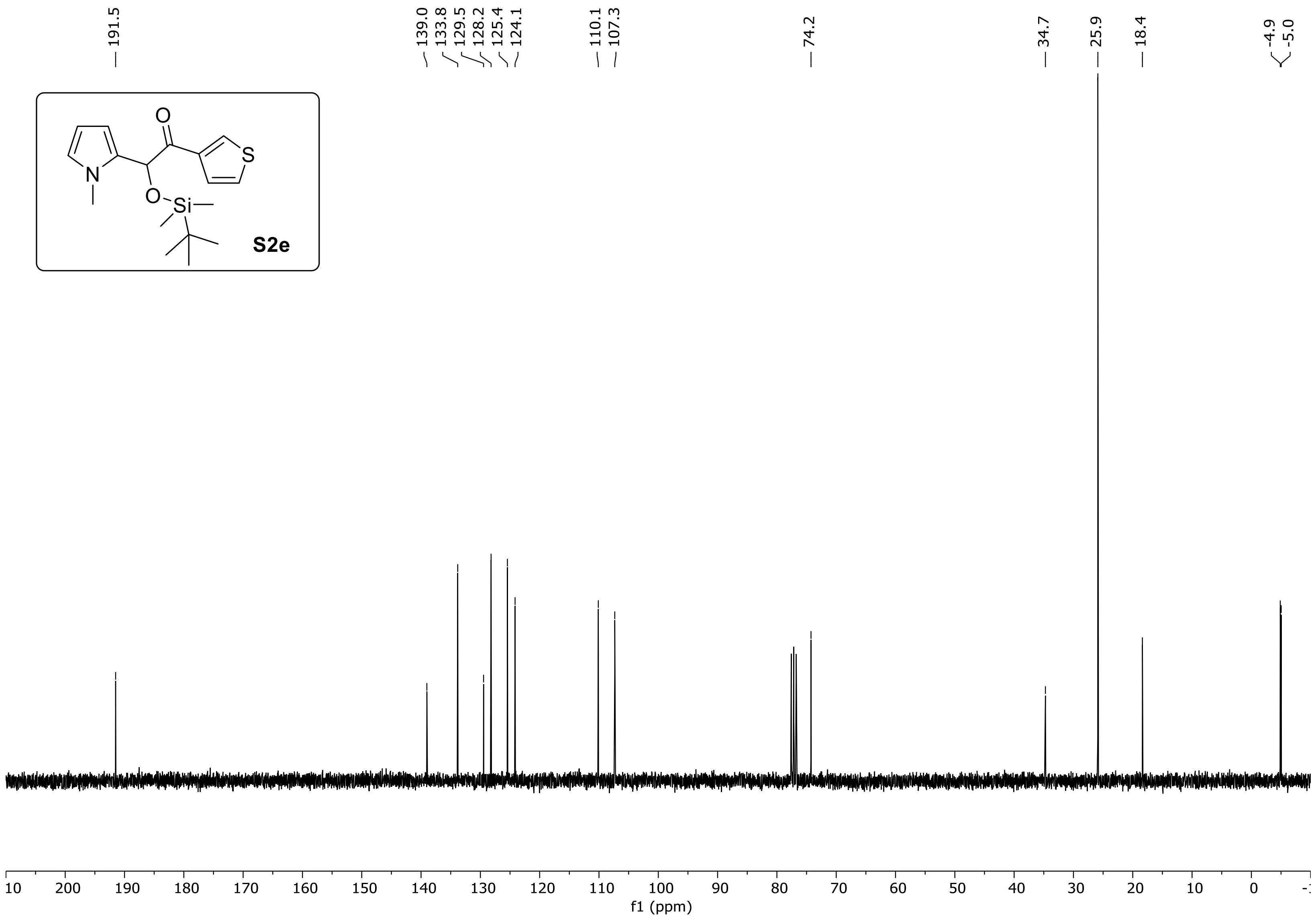

Figure S29: ^1H NMR of compound **S2f** in $CDCl_3$ at 300 MHz.

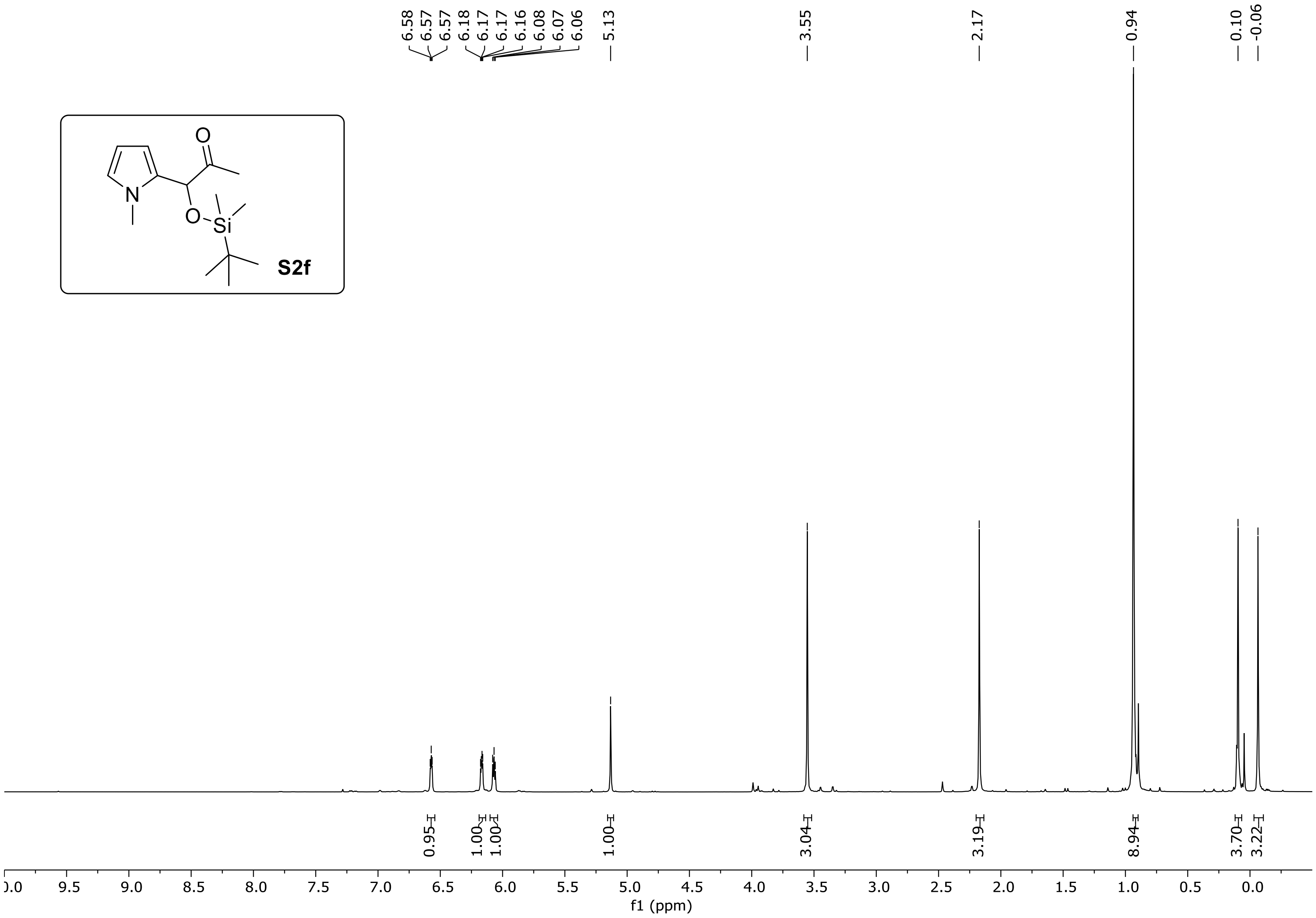

Figure S30: ^{13}C NMR of compound **S2f** in $CDCl_3$ at 75.4 MHz.

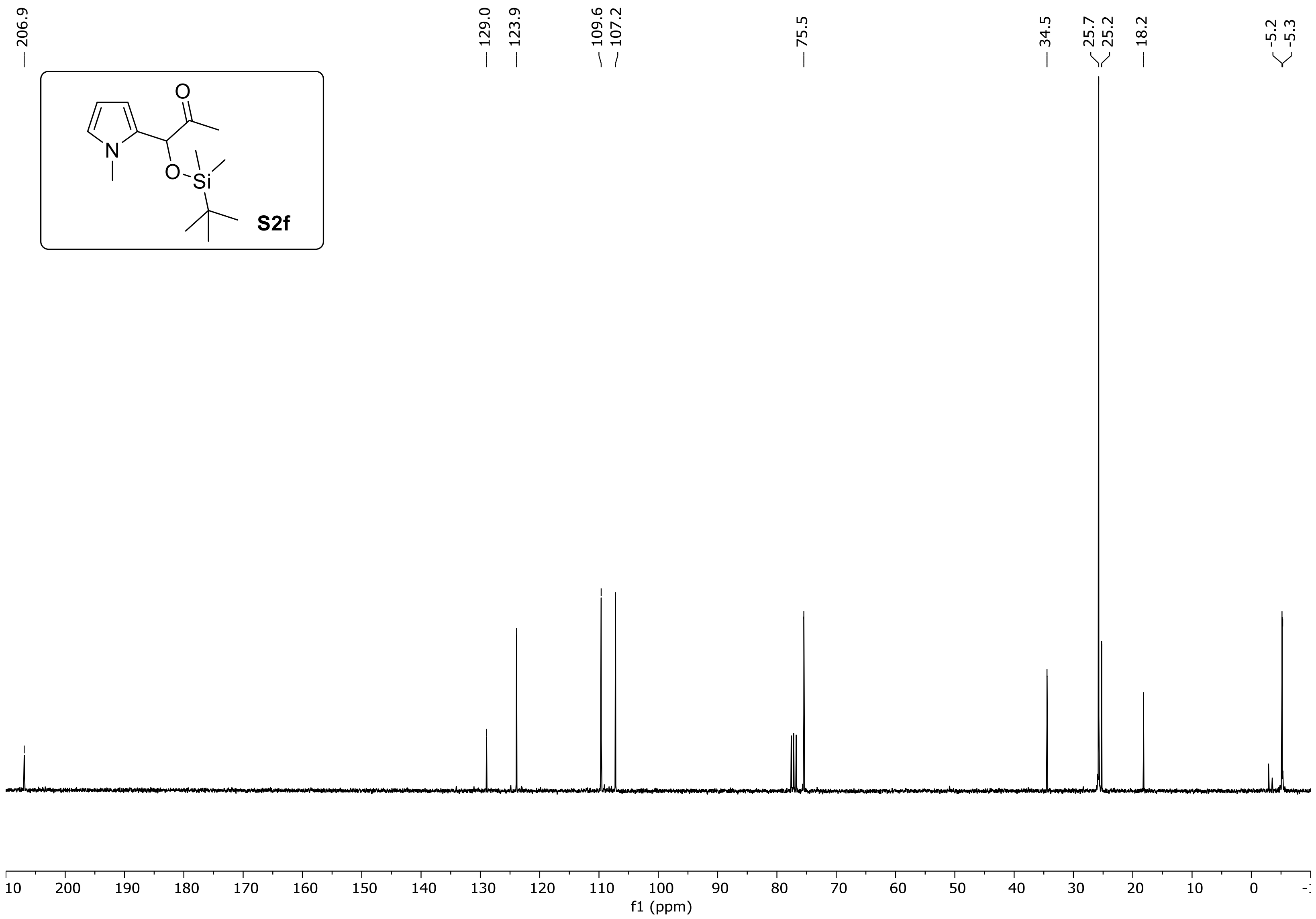

Figure S31: ^{1}H NMR of compound **S2g** in $CDCl_3$ at 500 MHz.

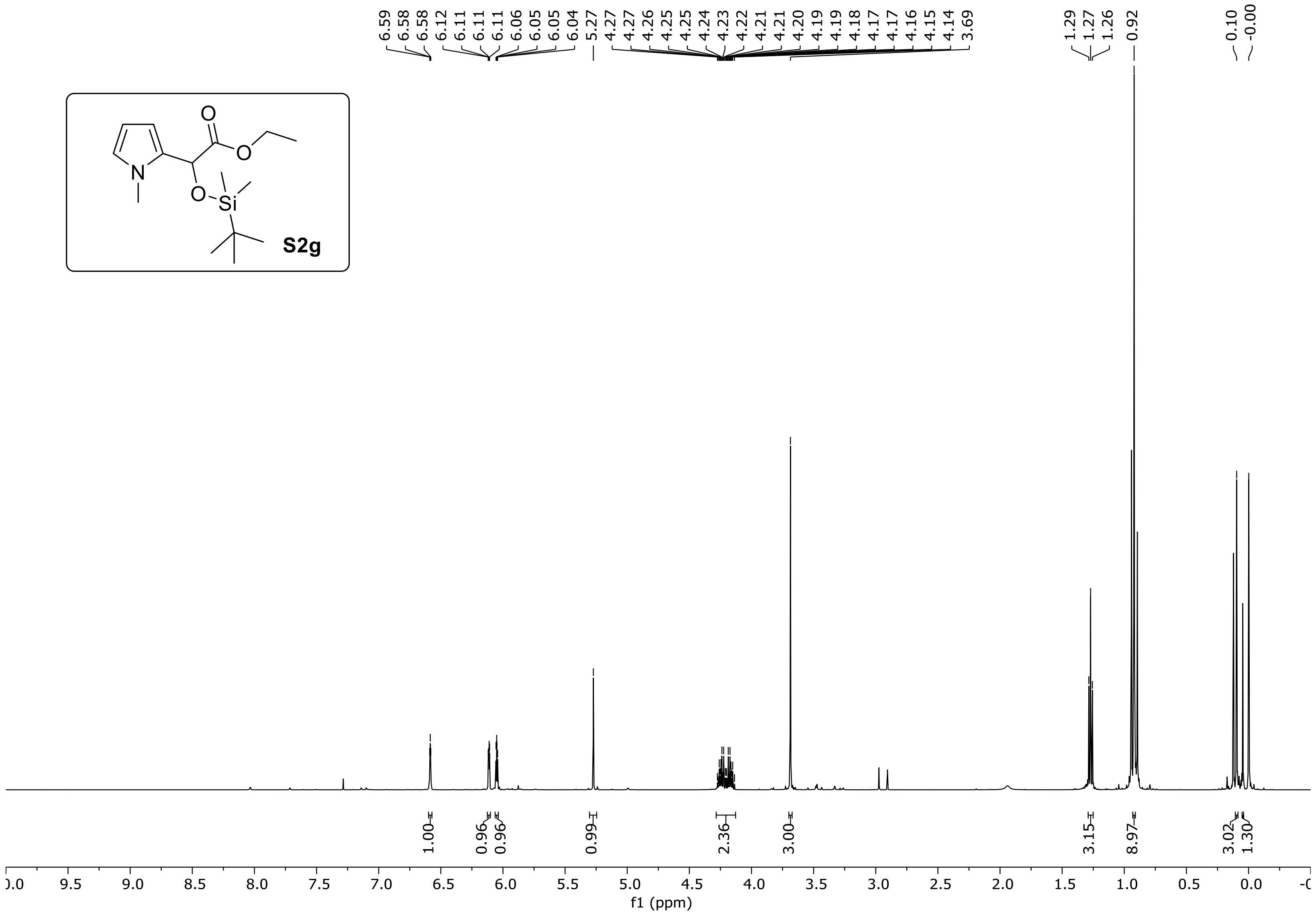

Figure S32: ^{13}C NMR of compound **S2g** in $CDCl_3$ at 125.7 MHz.

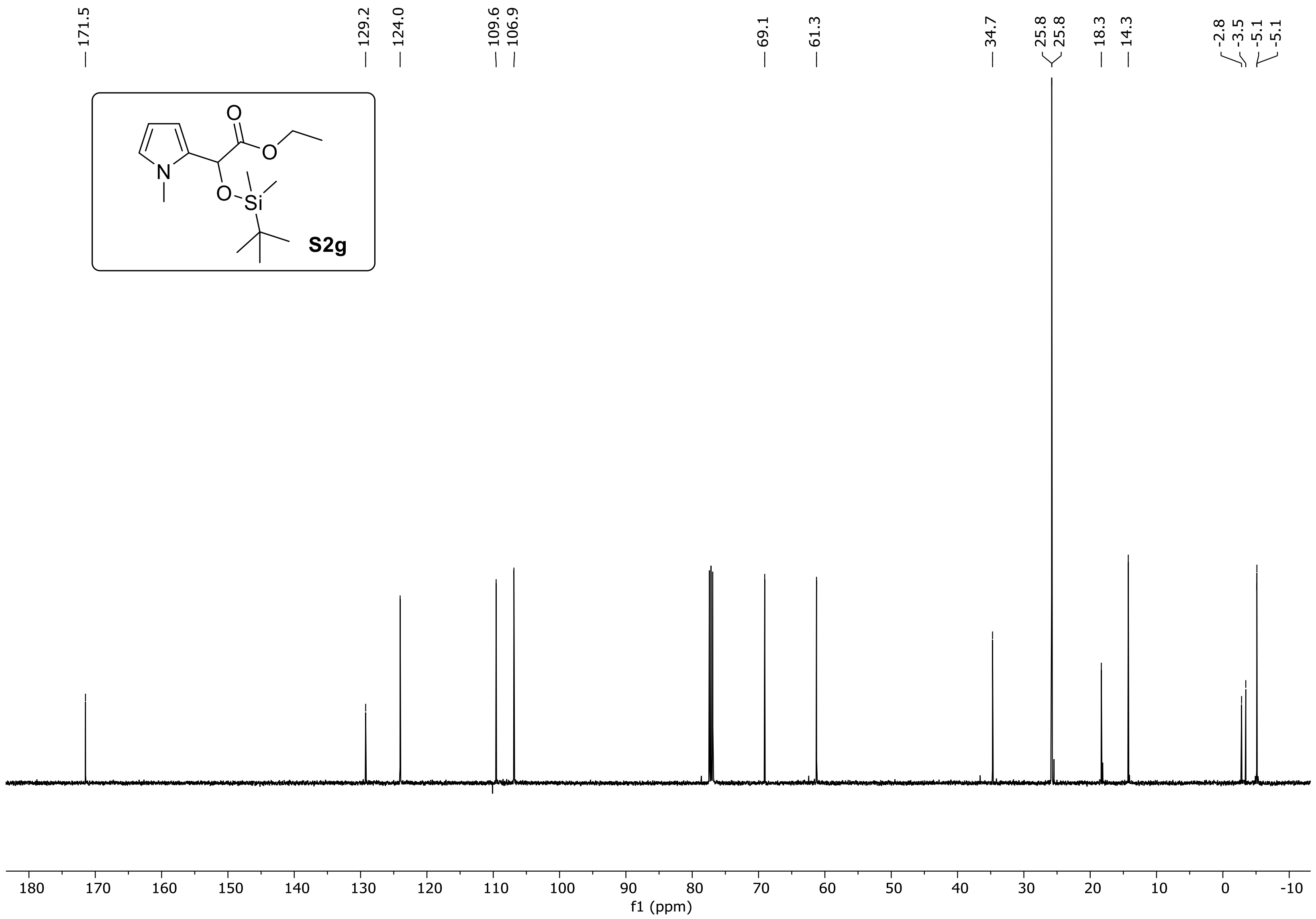

Figure S33: ^{1}H NMR of compound **S2j** in $CDCl_3$ at 300 MHz.

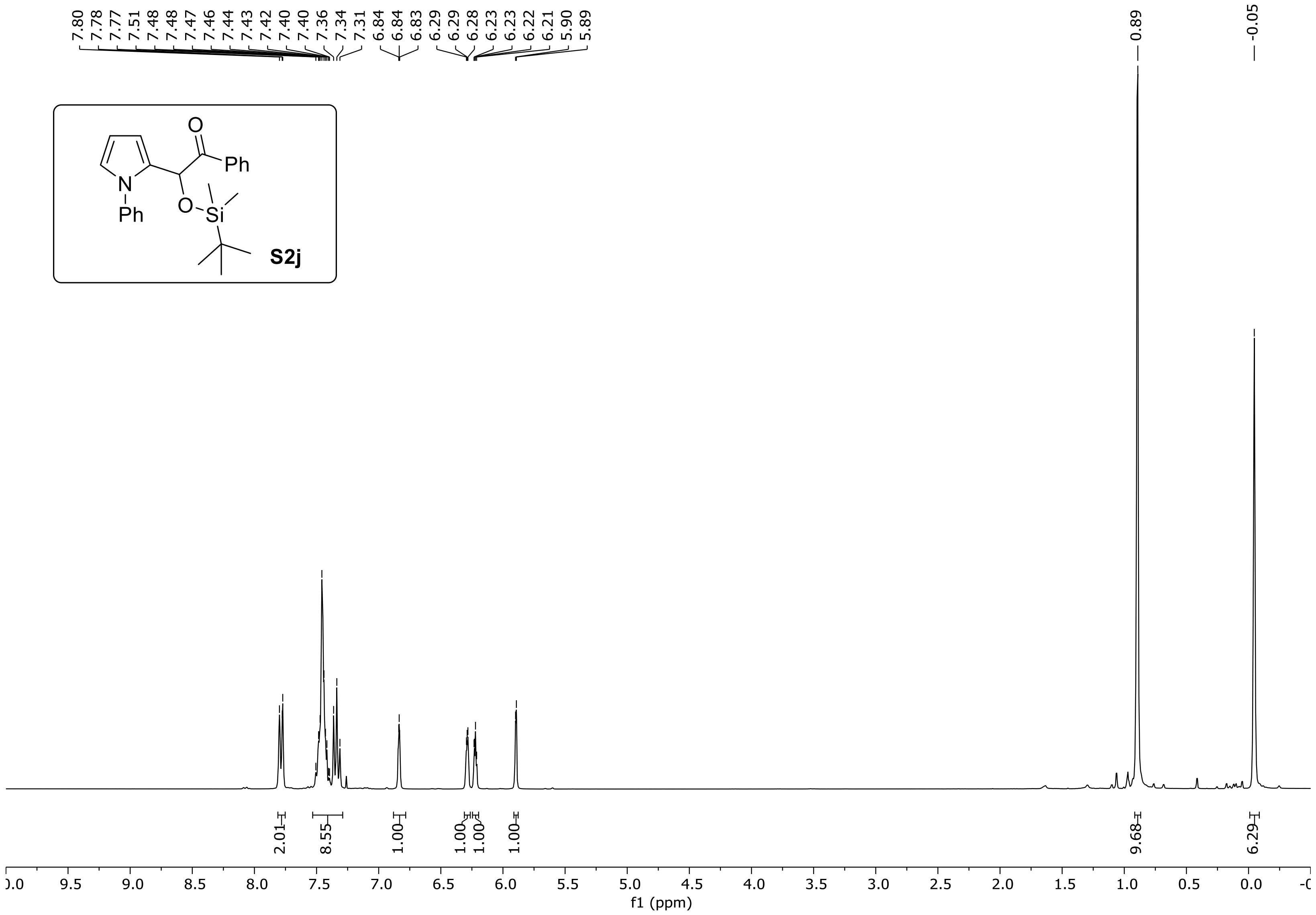

Figure S34: ^{13}C NMR of compound **S2j** in $CDCl_3$ at 75.4 MHz.

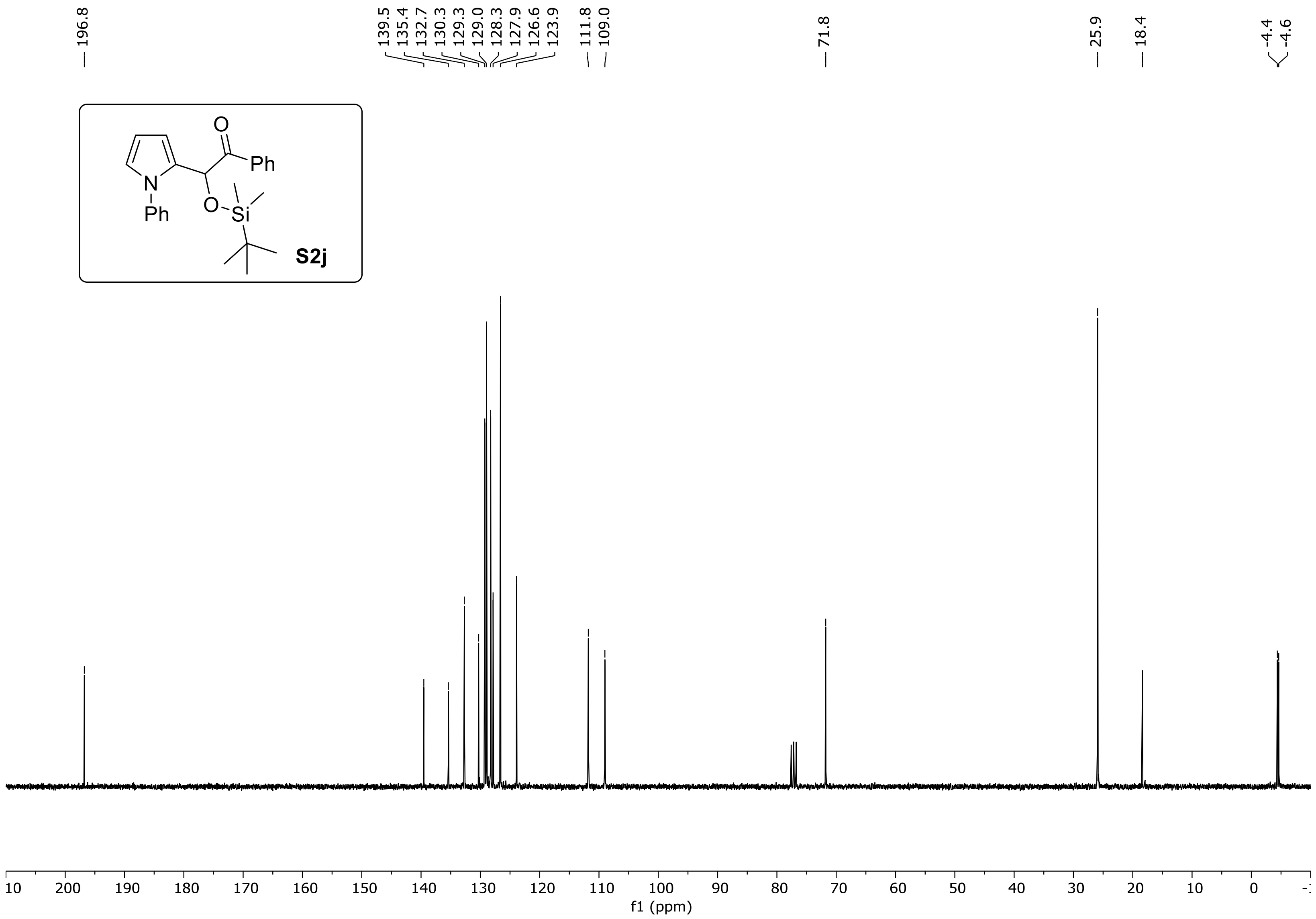

Figure S35: ^{1}H NMR of compound **1aa** in $CDCl_3$ at 300 MHz.

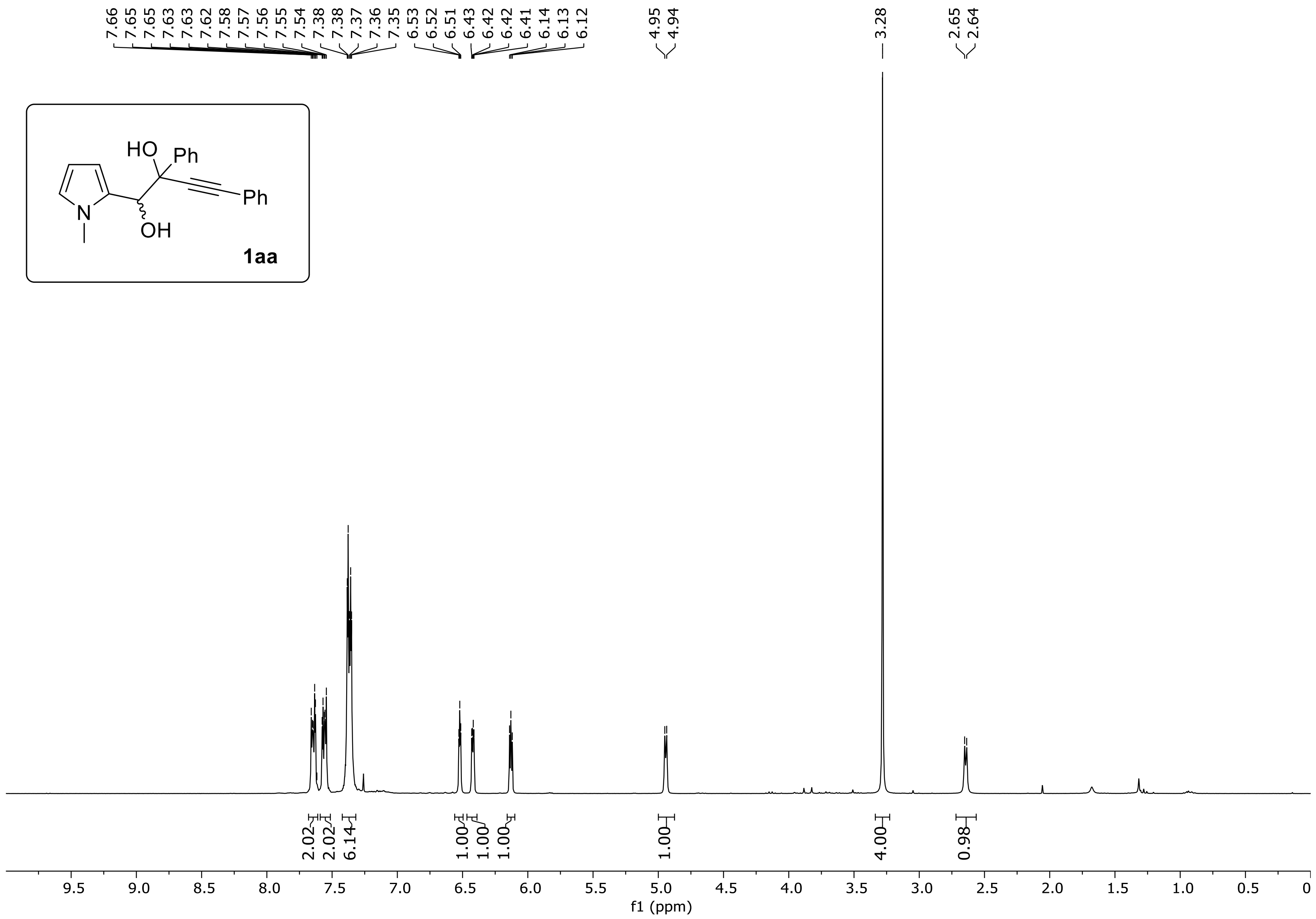

Figure S36: ^{13}C NMR of compound **1aa** in $CDCl_3$ at 75.4 MHz.

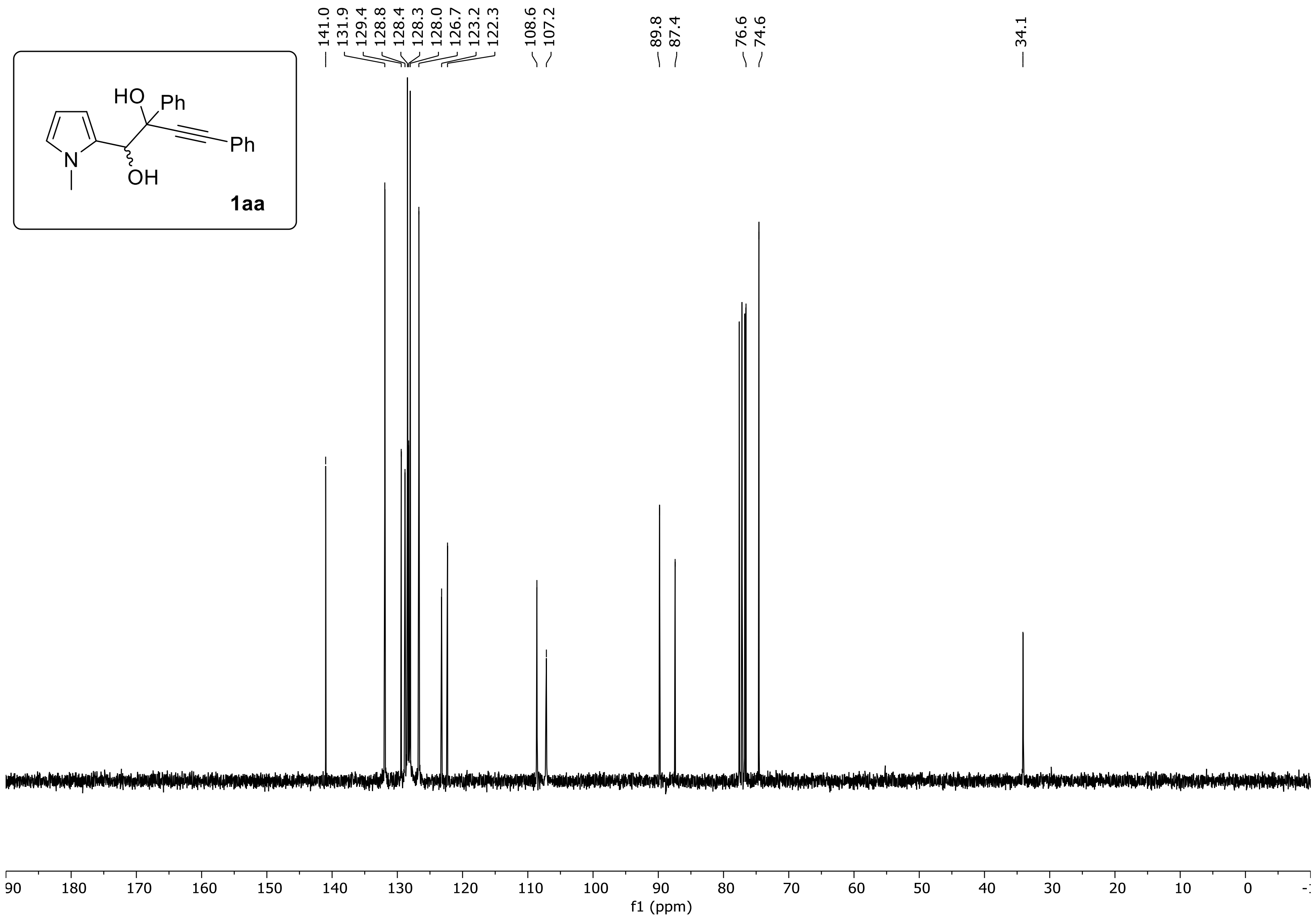

Figure S37: ^{1}H NMR of compound **1ab** in $CDCl_3$ at 300 MHz.

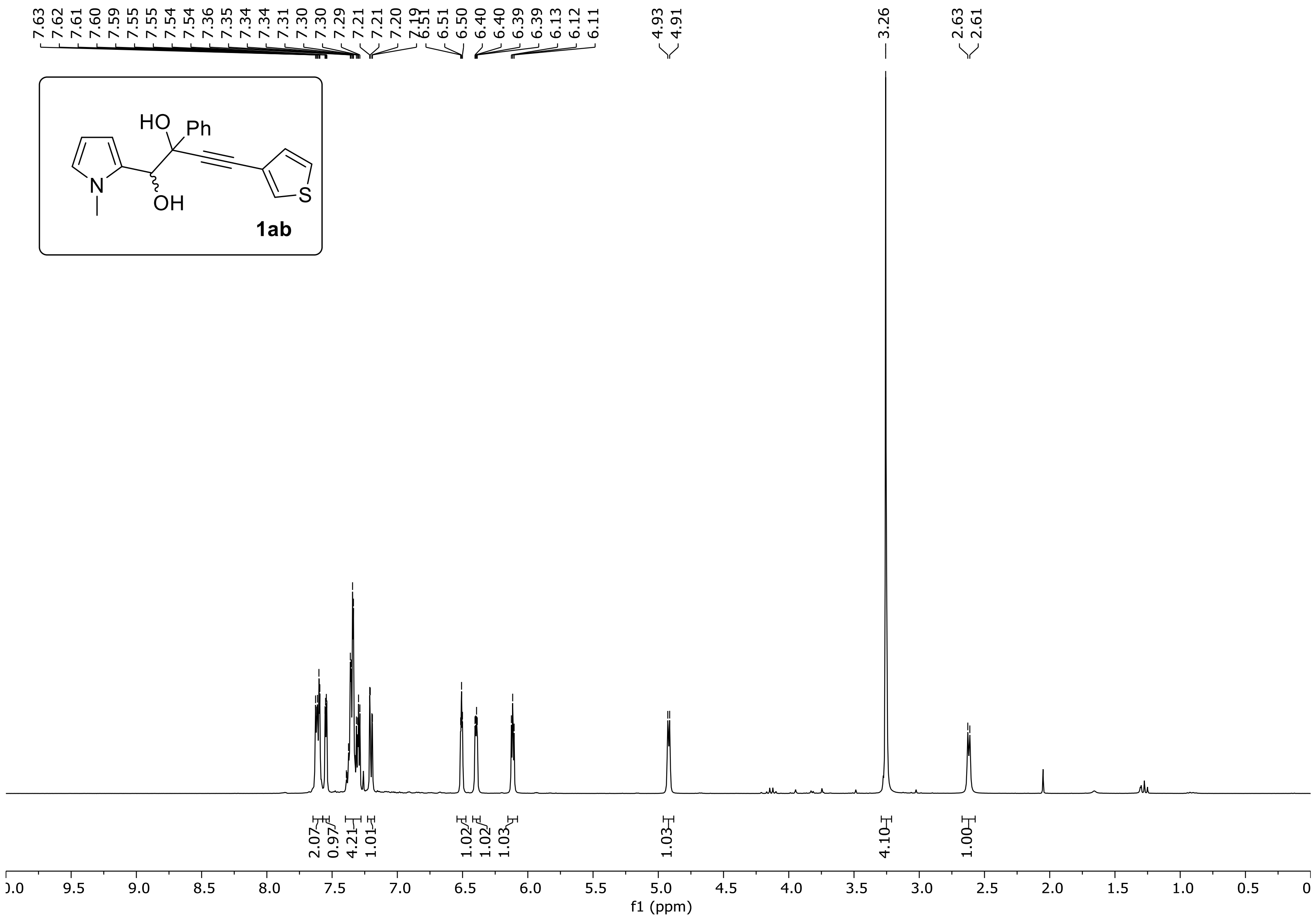

Figure S38: ^{13}C NMR of compound **1ab** in $CDCl_3$ at 75.4 MHz.

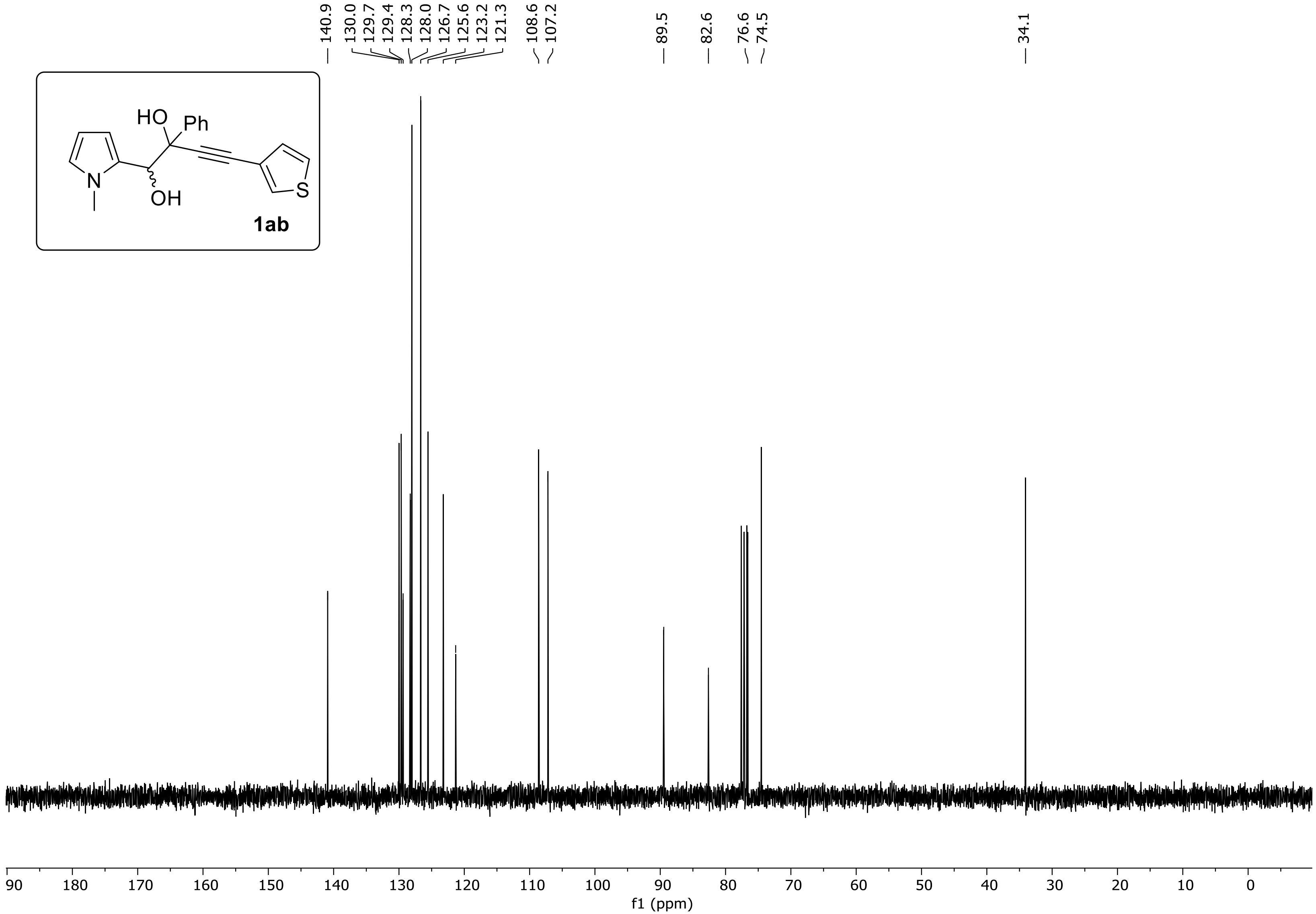

Figure S39: ^{1}H NMR of compound **1af** in $CDCl_3$ at 300 MHz.

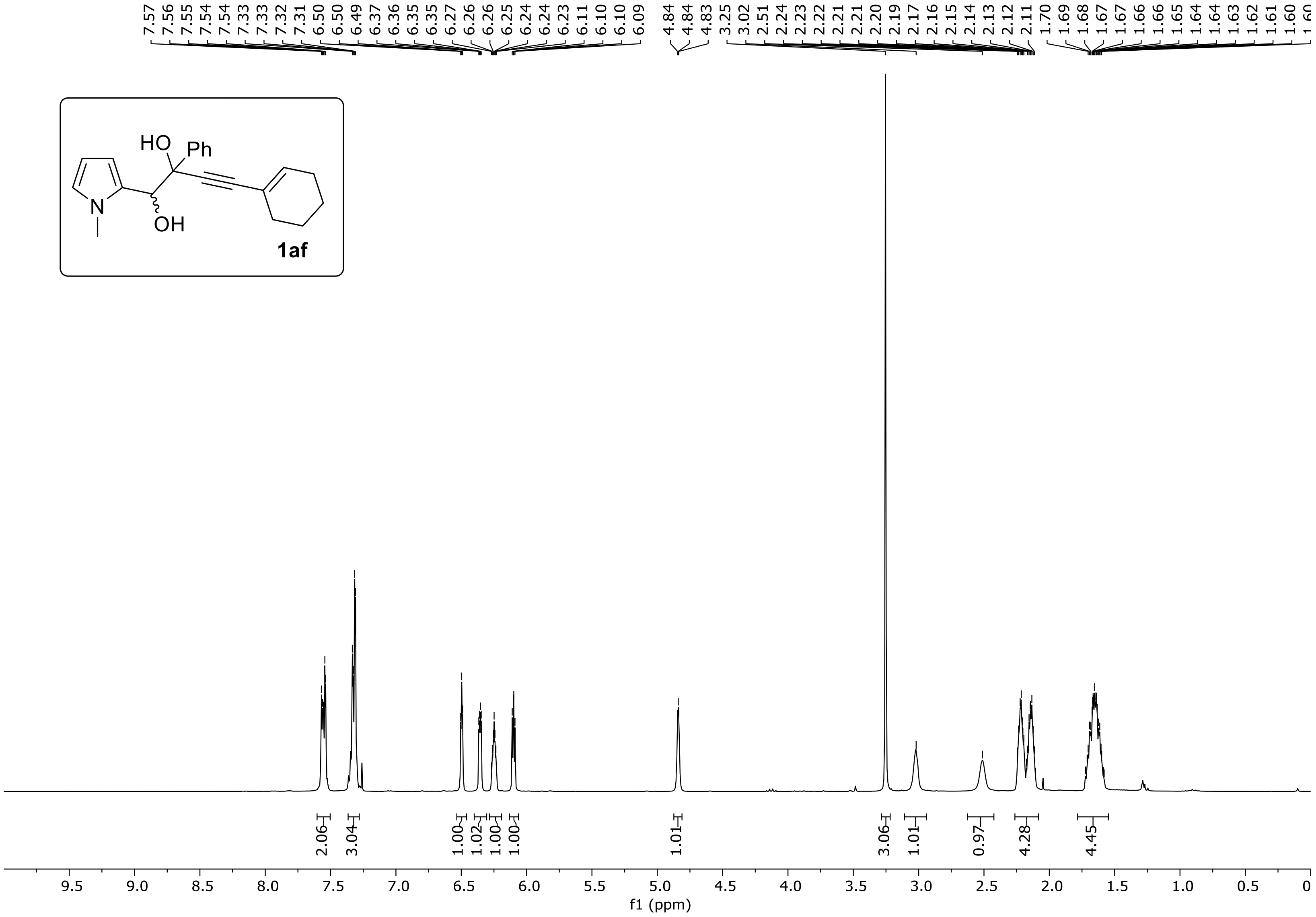

Figure S40: ^{13}C NMR of compound **1af** in $CDCl_3$ at 75.4 MHz.

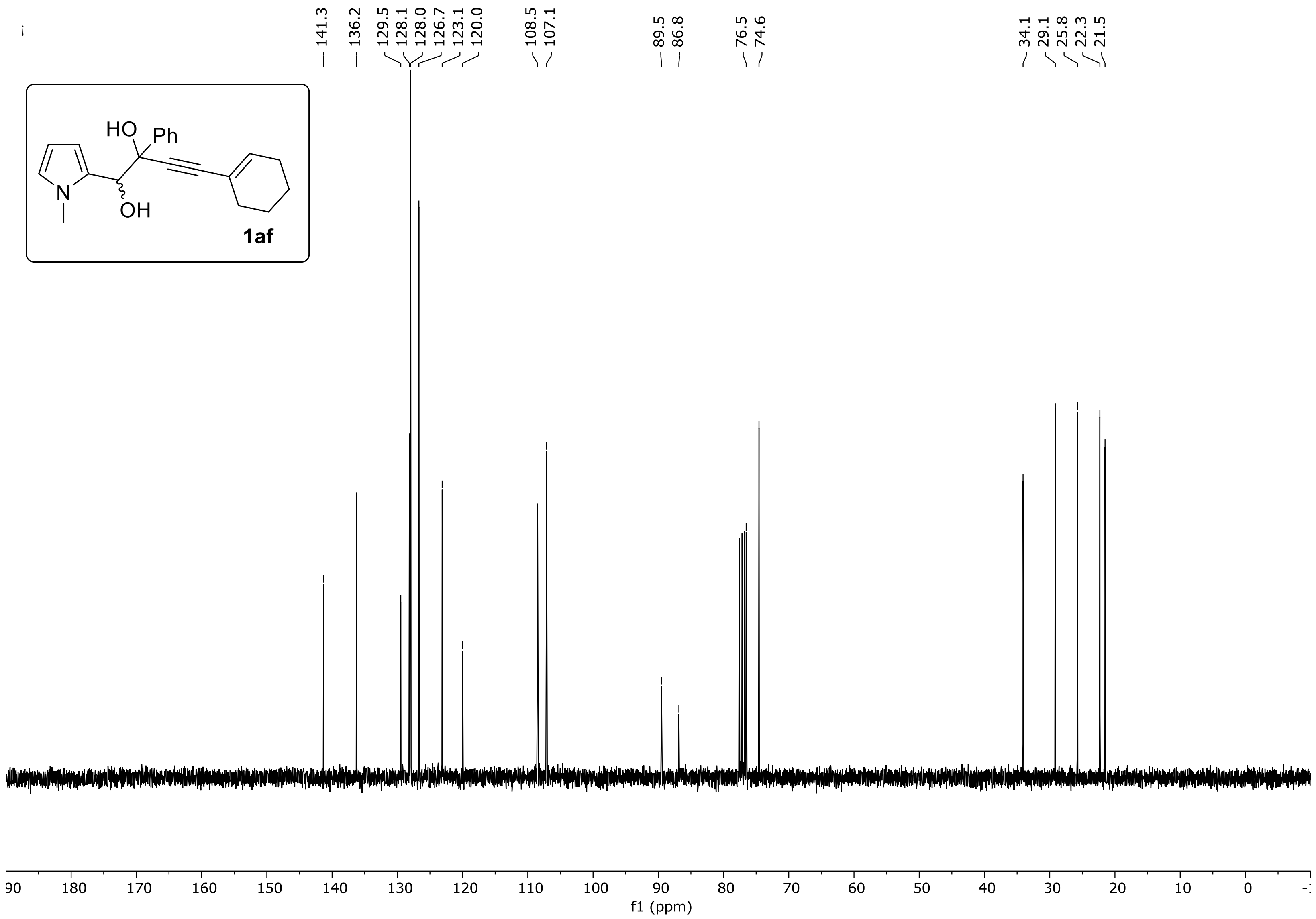

Figure S41: ^{1}H NMR of compound **1ah** in $CDCl_3$ at 300 MHz.

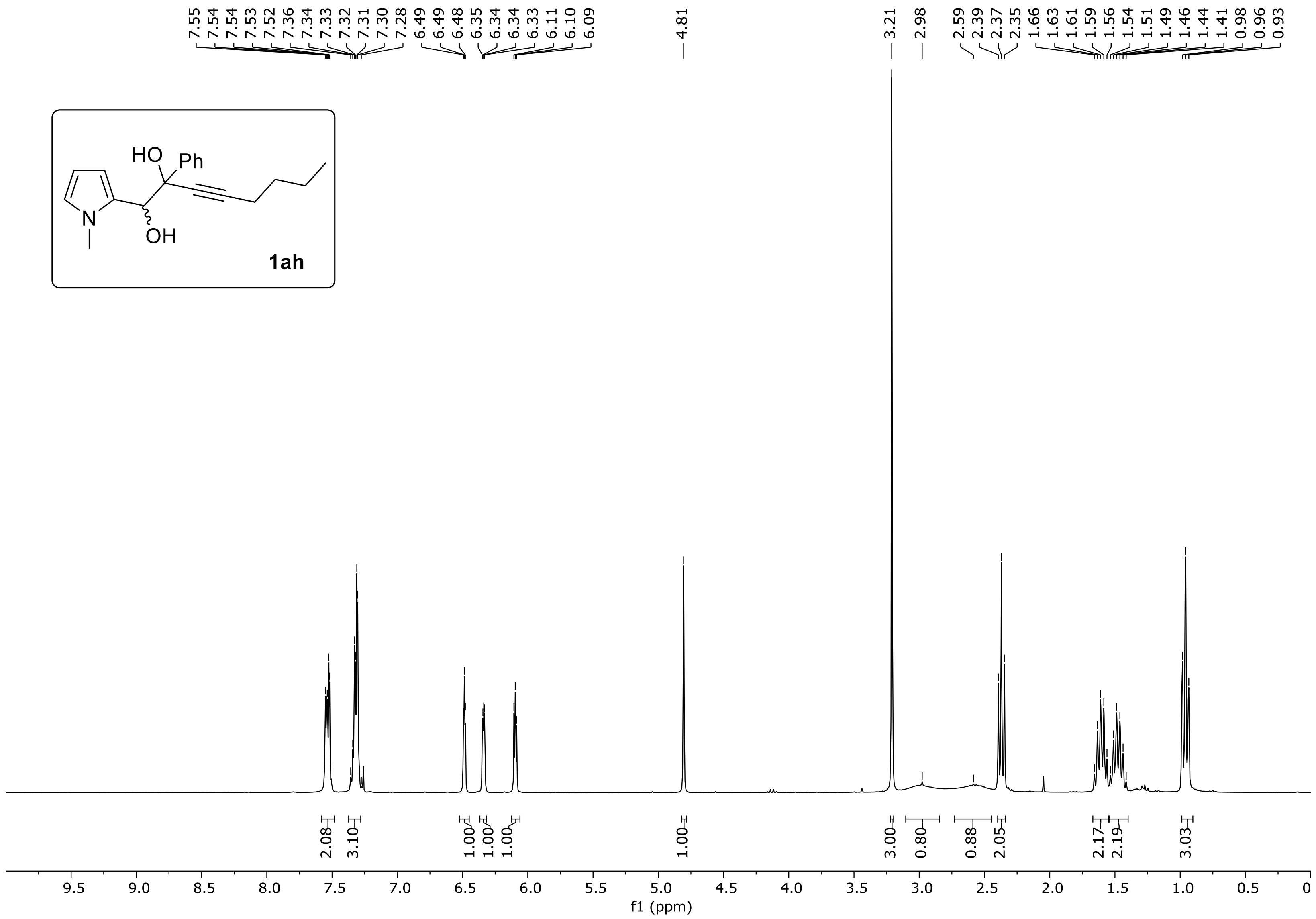

Figure S42: ^{13}C NMR of compound **1ah** in $CDCl_3$ at 75.4 MHz.

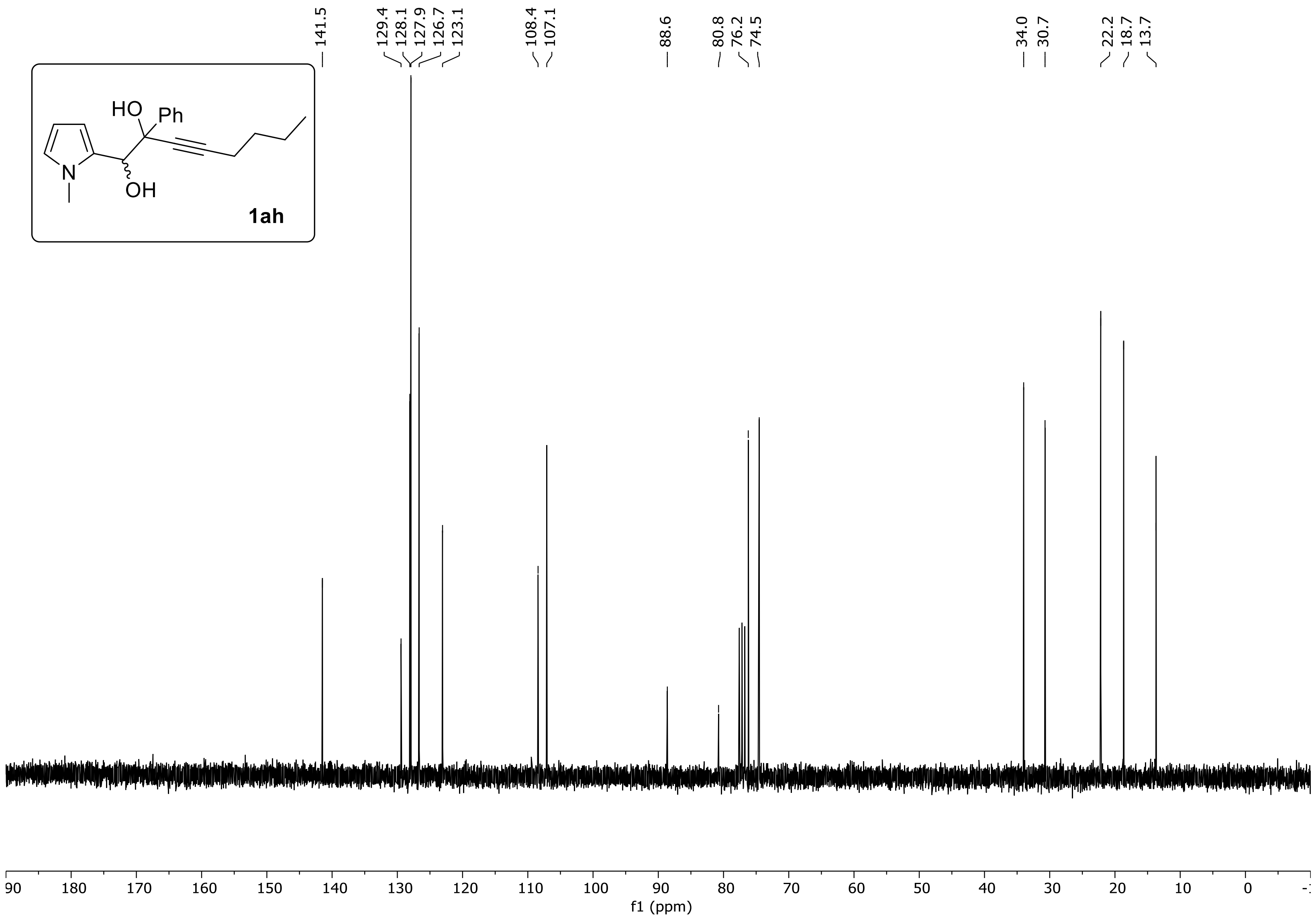

Figure S43: ^{1}H NMR of compound **1ai** in $CDCl_3$ at 300 MHz.

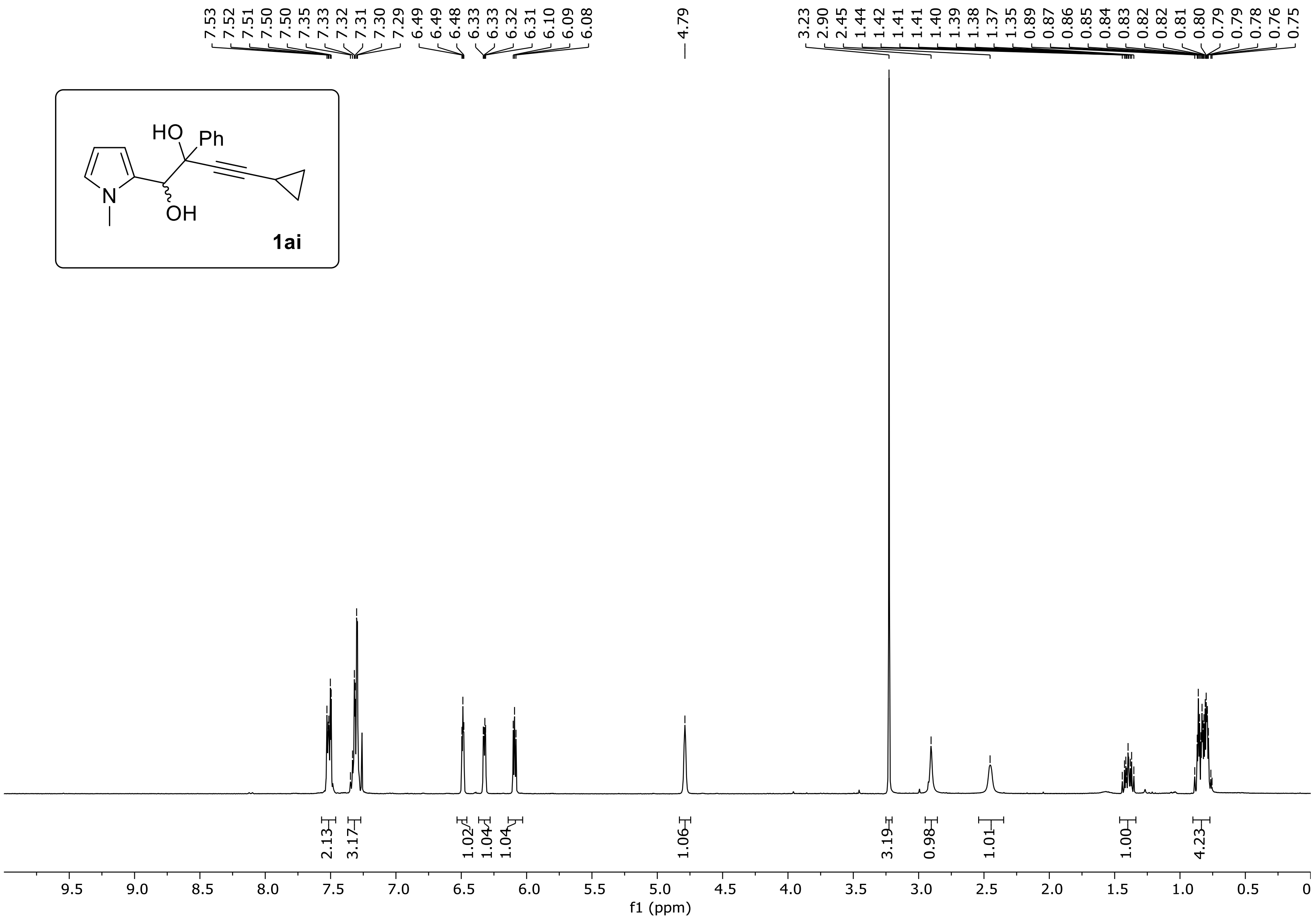

Figure S44: ^{13}C NMR of compound **1ai** in $CDCl_3$ at 75.4 MHz.

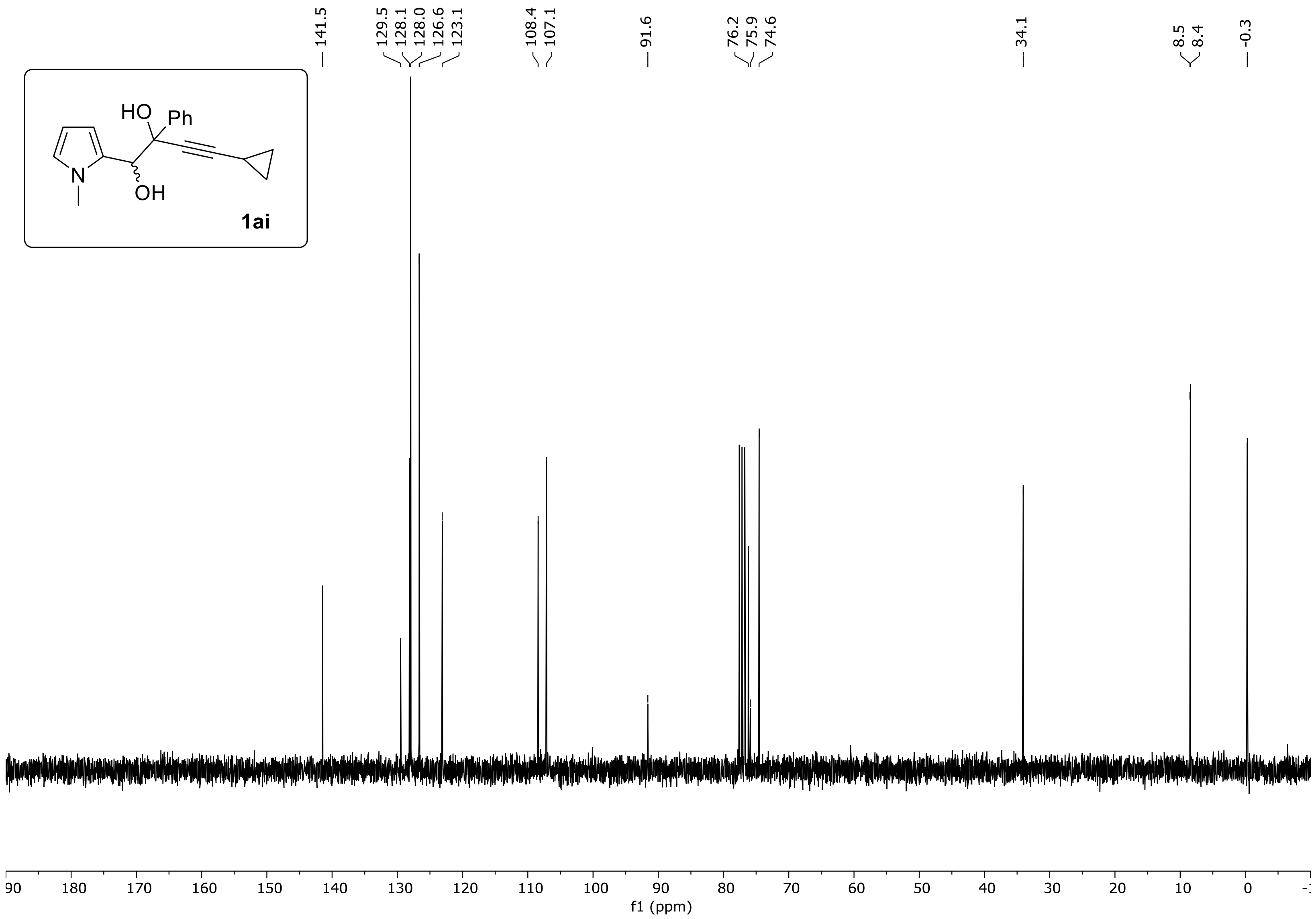

Figure S45: ^{1}H NMR of compound **1aj** in $CDCl_3$ at 300 MHz.

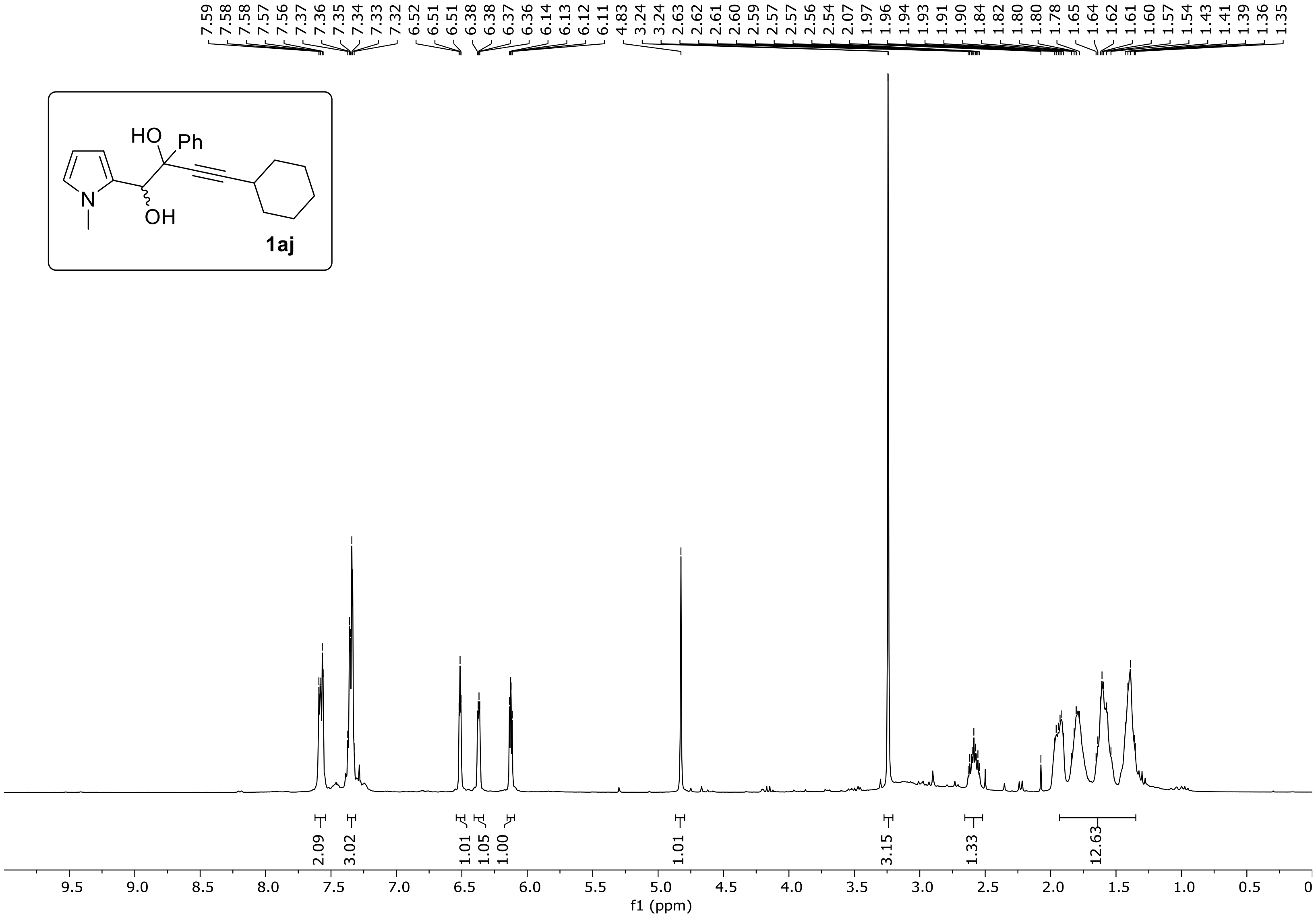

Figure S46: ^{13}C NMR of compound **1aj** in $CDCl_3$ at 75.4 MHz.

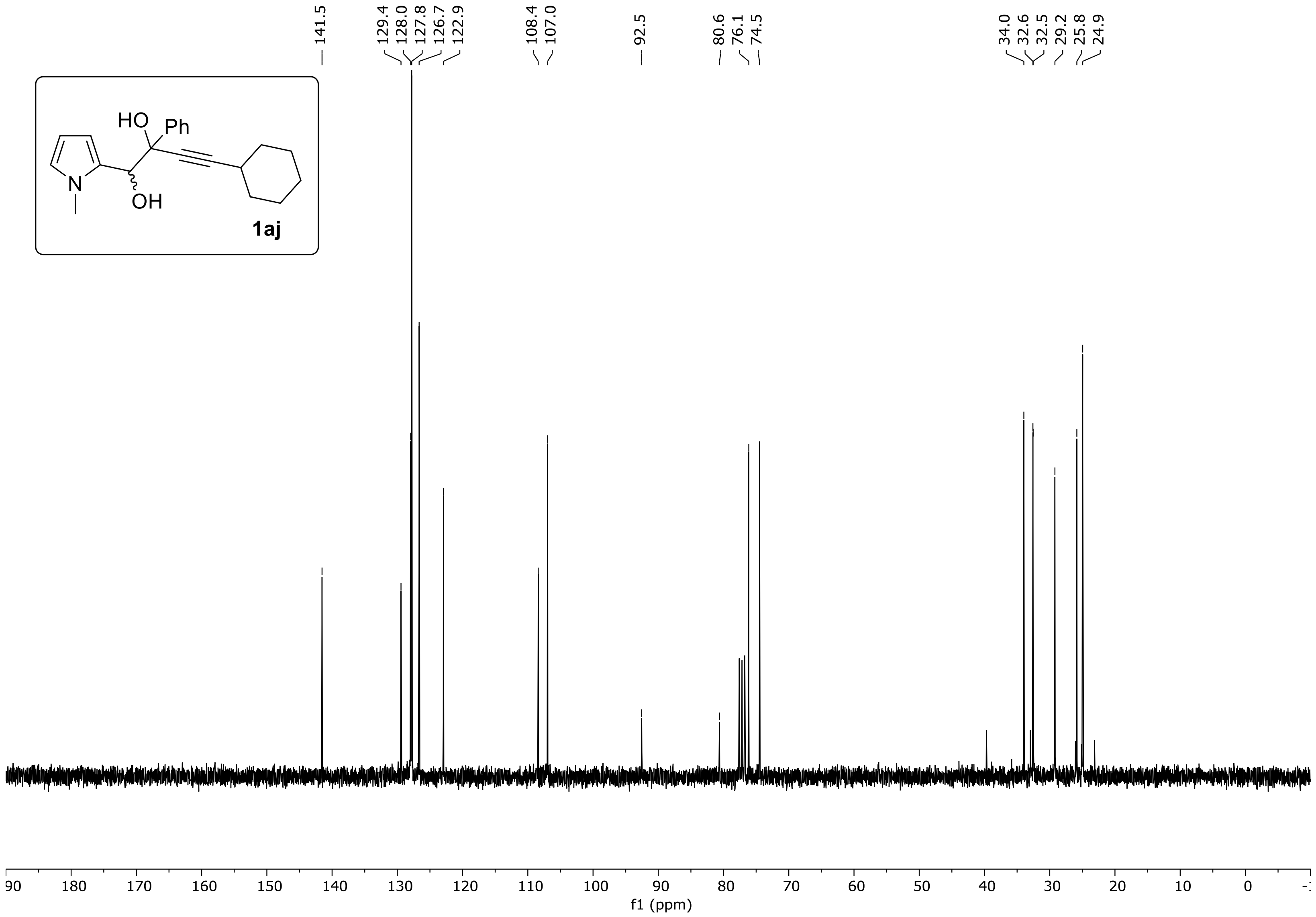

Figure S47: ^{1}H NMR of compound **1ha** in $CDCl_3$ at 300 MHz.

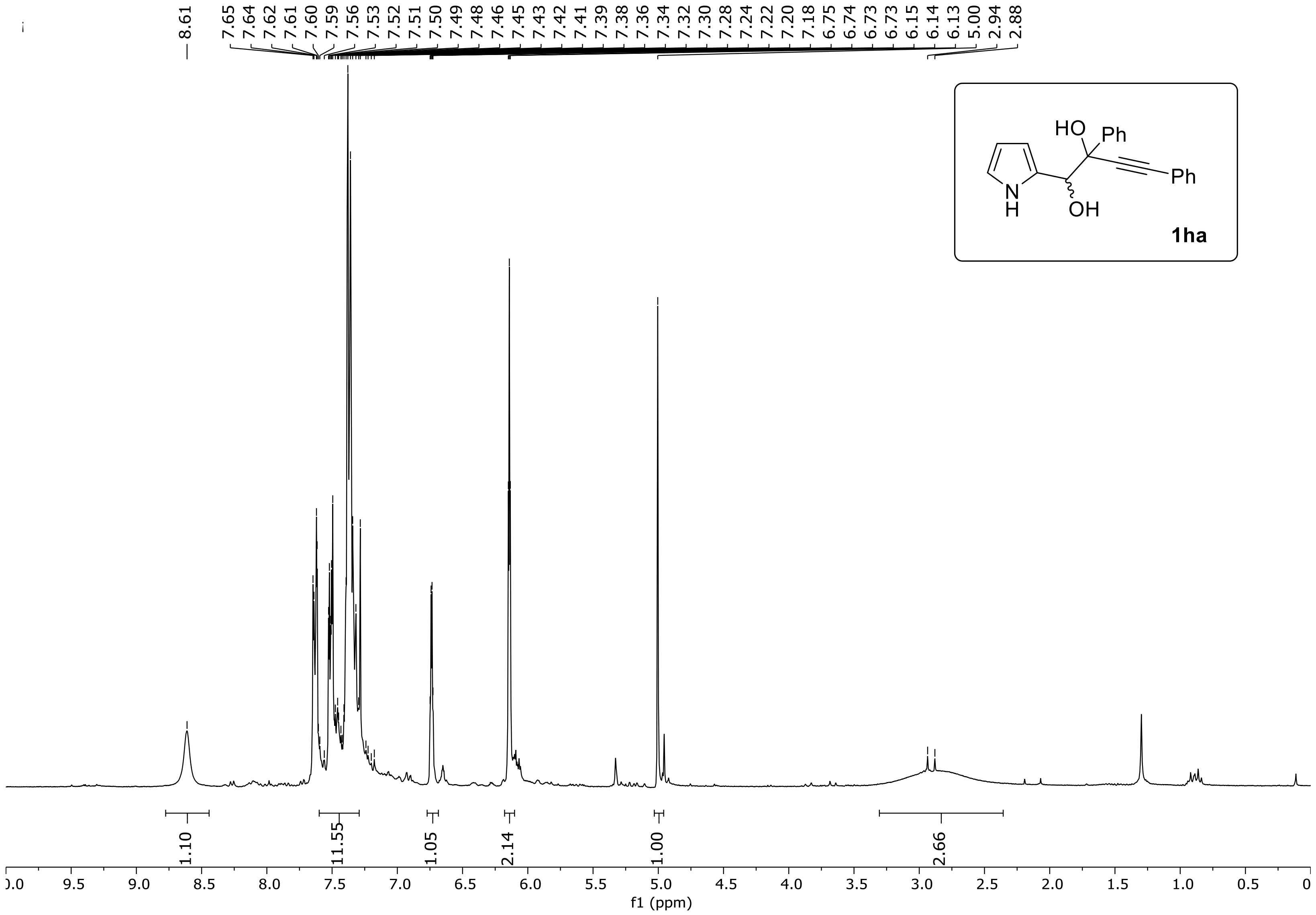

Figure S48: ^{13}C NMR of compound **1ha** in $CDCl_3$ at 75.4 MHz.

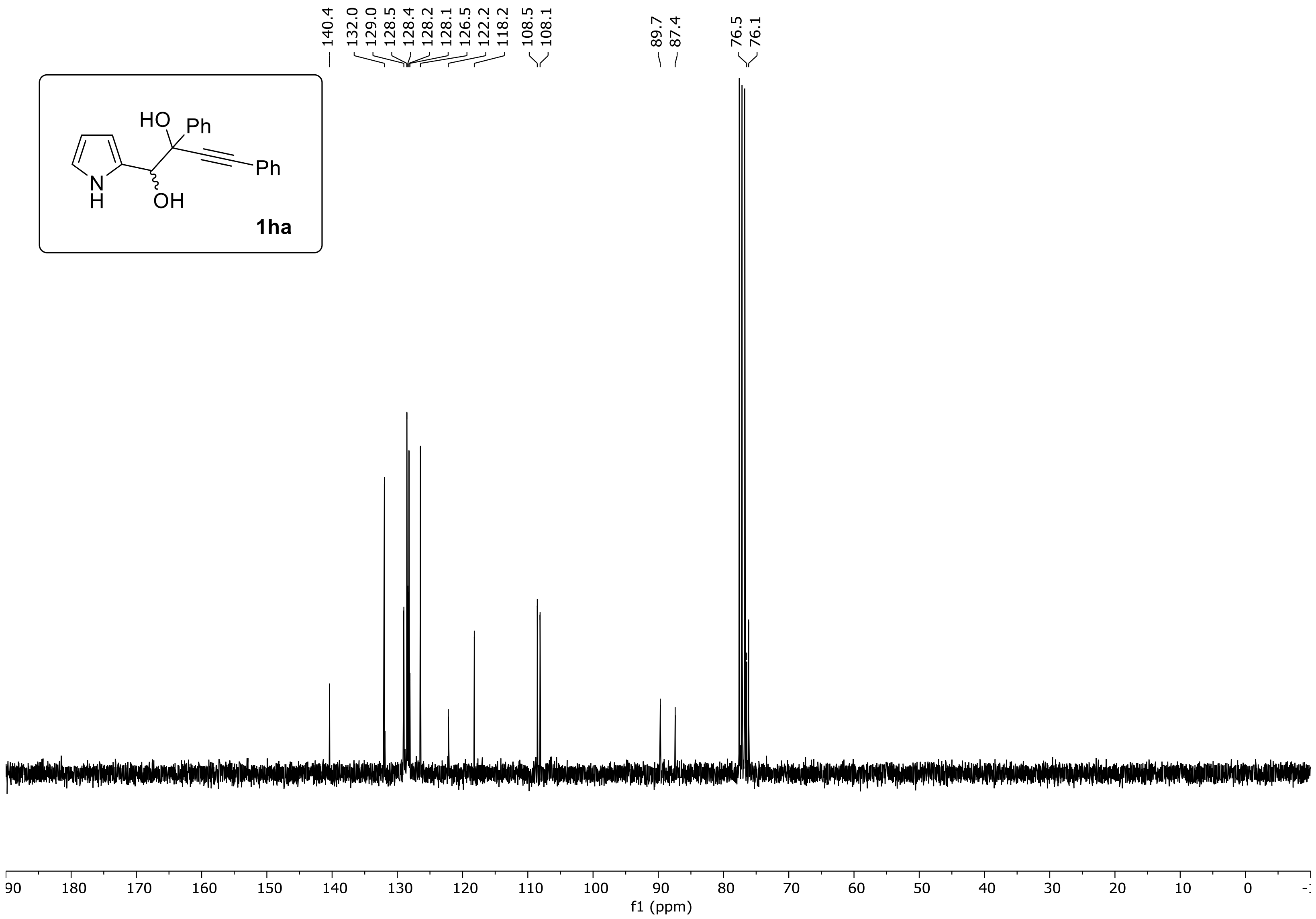

Figure S49: ^{1}H NMR of compound **1hb** in acetone-*d6* at 300 MHz.

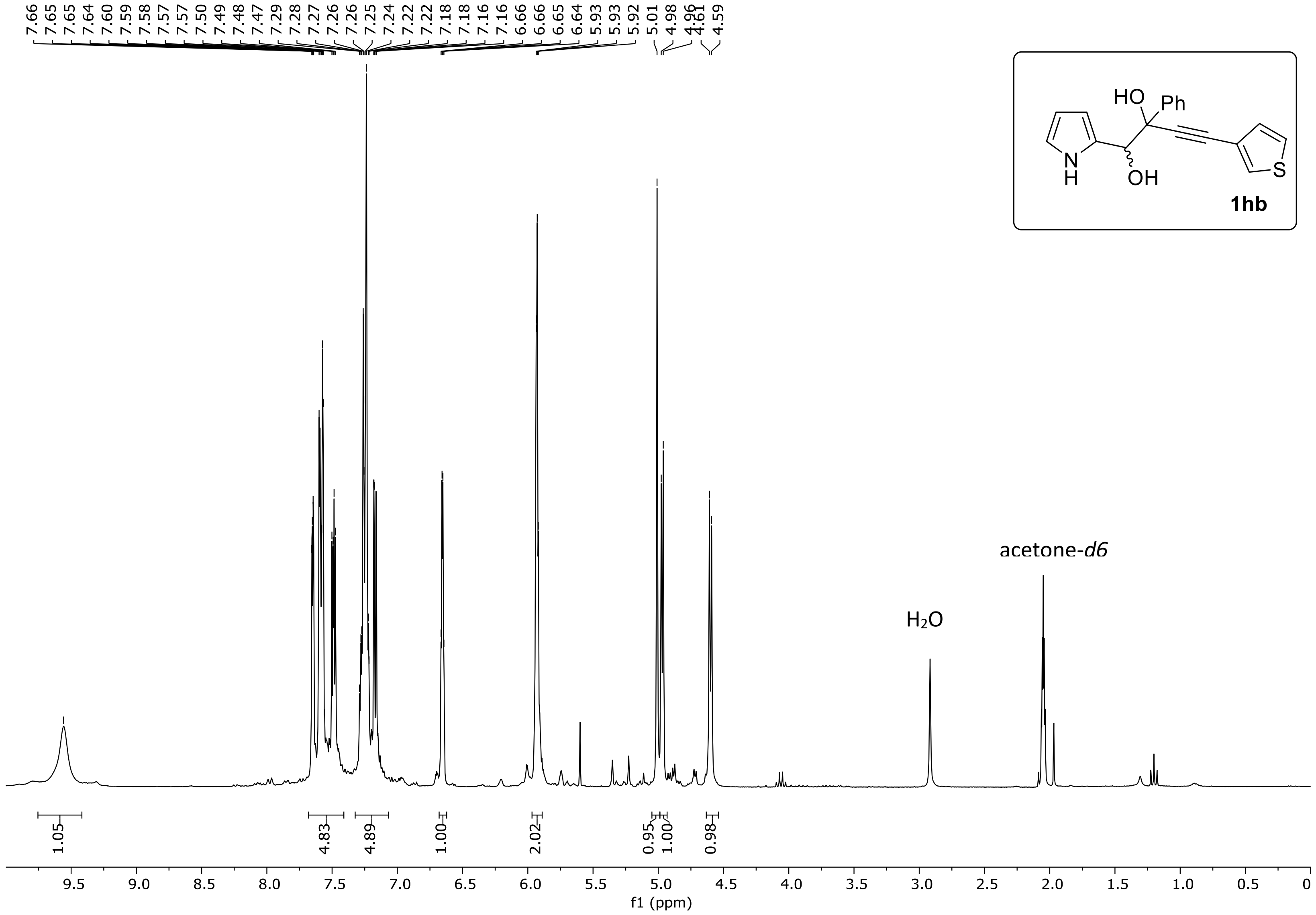

Figure S50: ^{13}C NMR of compound **1hb** in acetone-*d6* at 75.4 MHz.

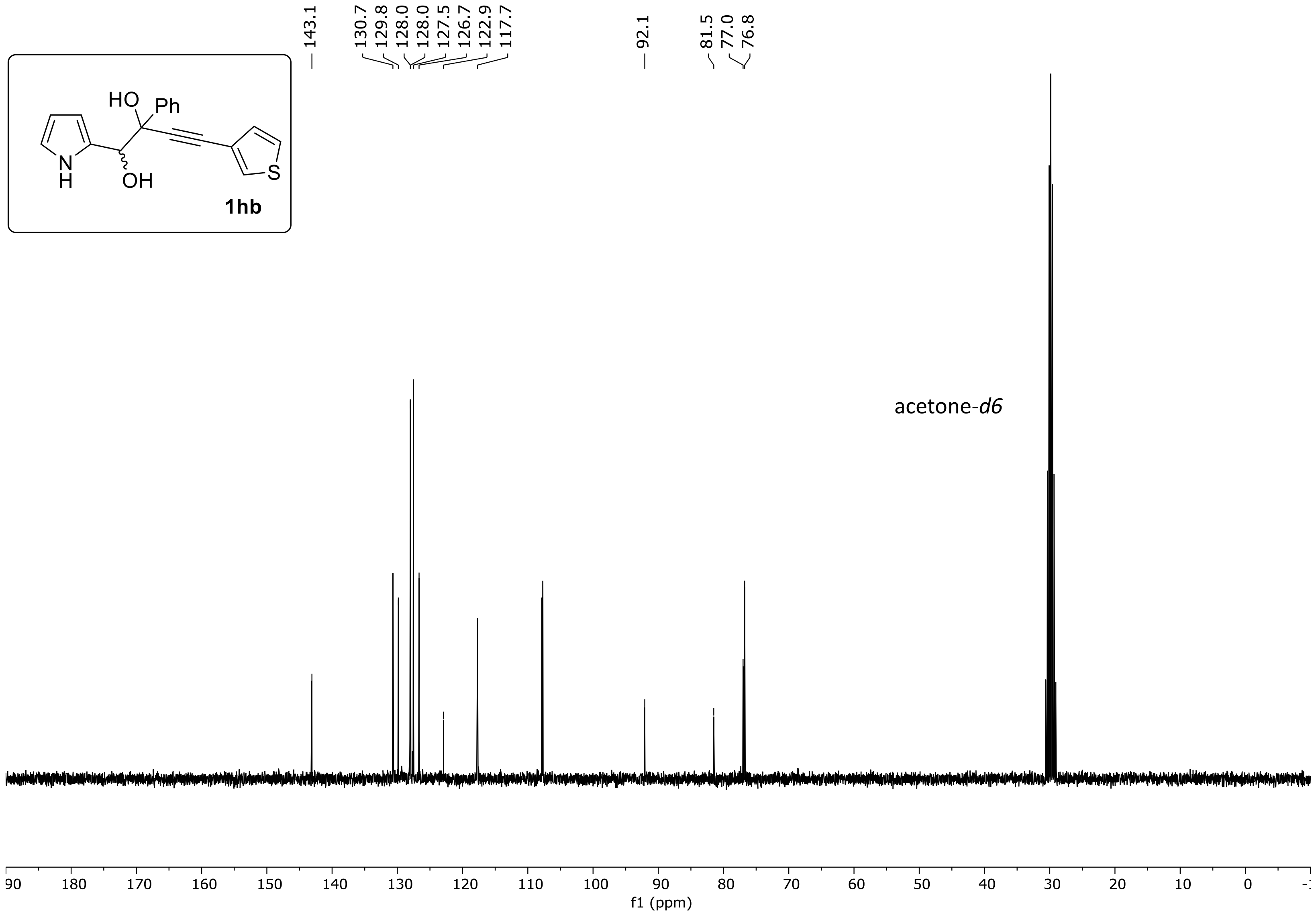

Figure S51: ^{1}H NMR of compound **1hh** in $CDCl_3$ at 300 MHz.

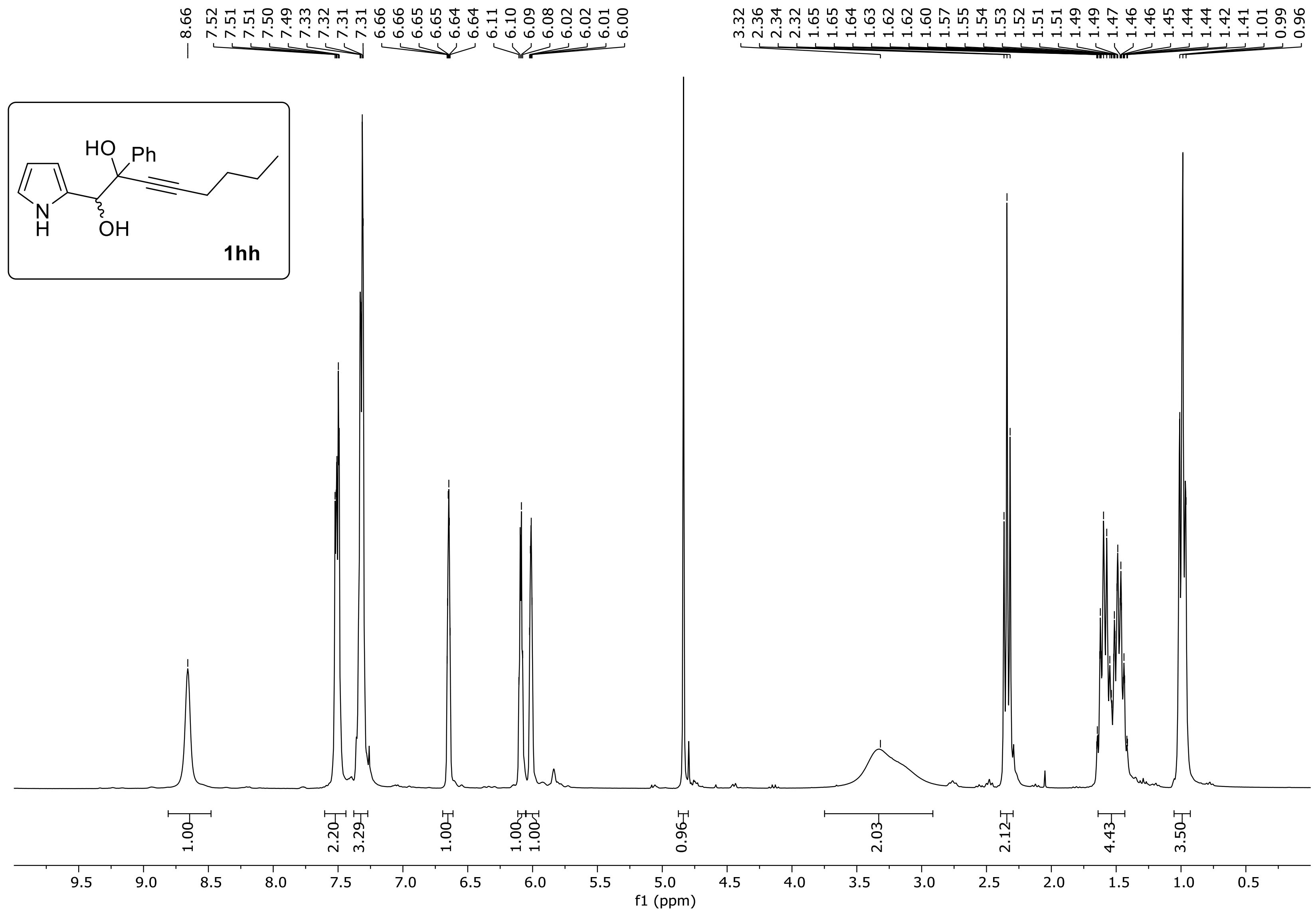

Figure S52: ^{13}C NMR of compound **1hh** in $CDCl_3$ at 75.4 MHz.

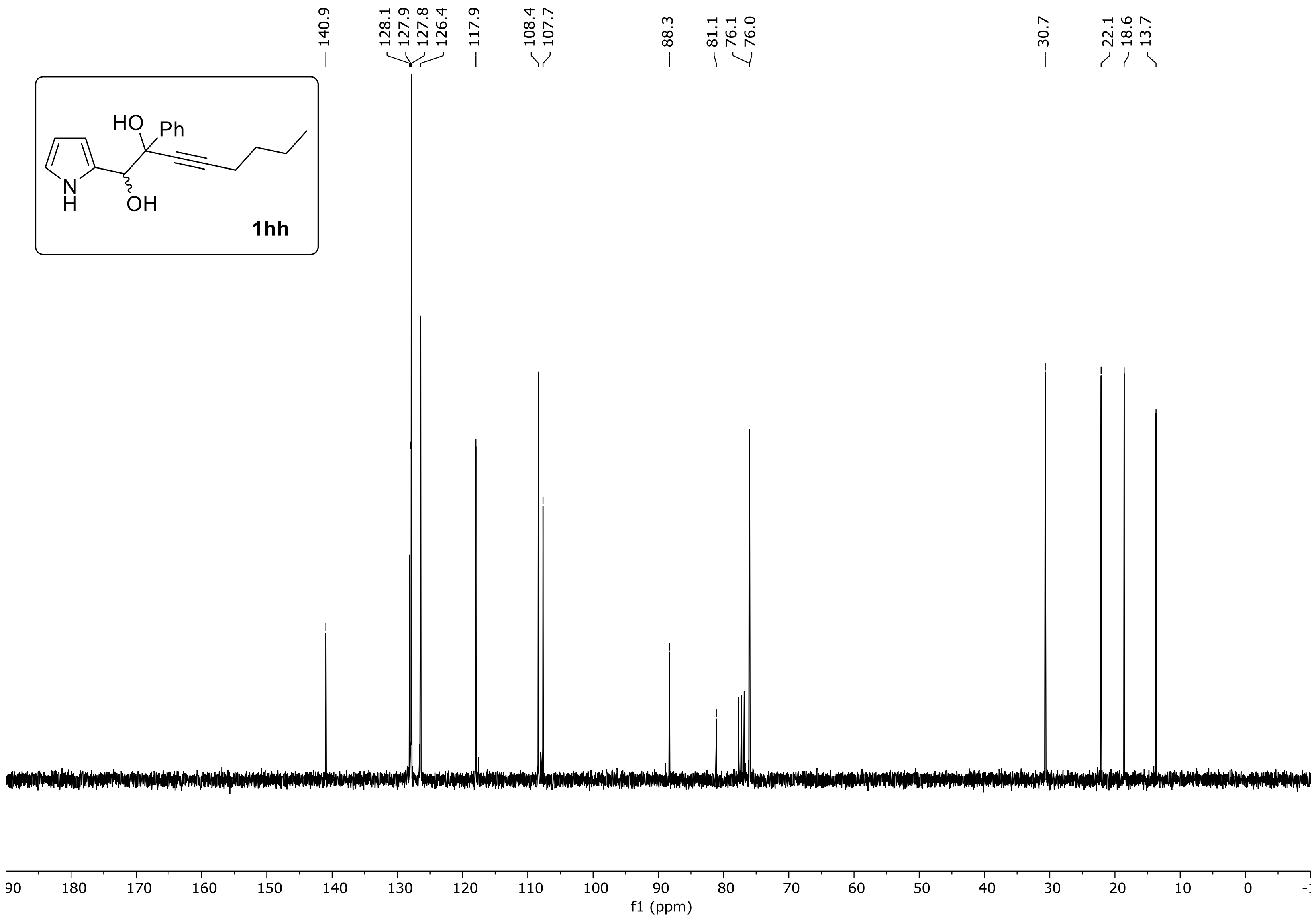

Figure S53: ^{1}H NMR of compound **1ia** in $CDCl_3$ at 300 MHz.

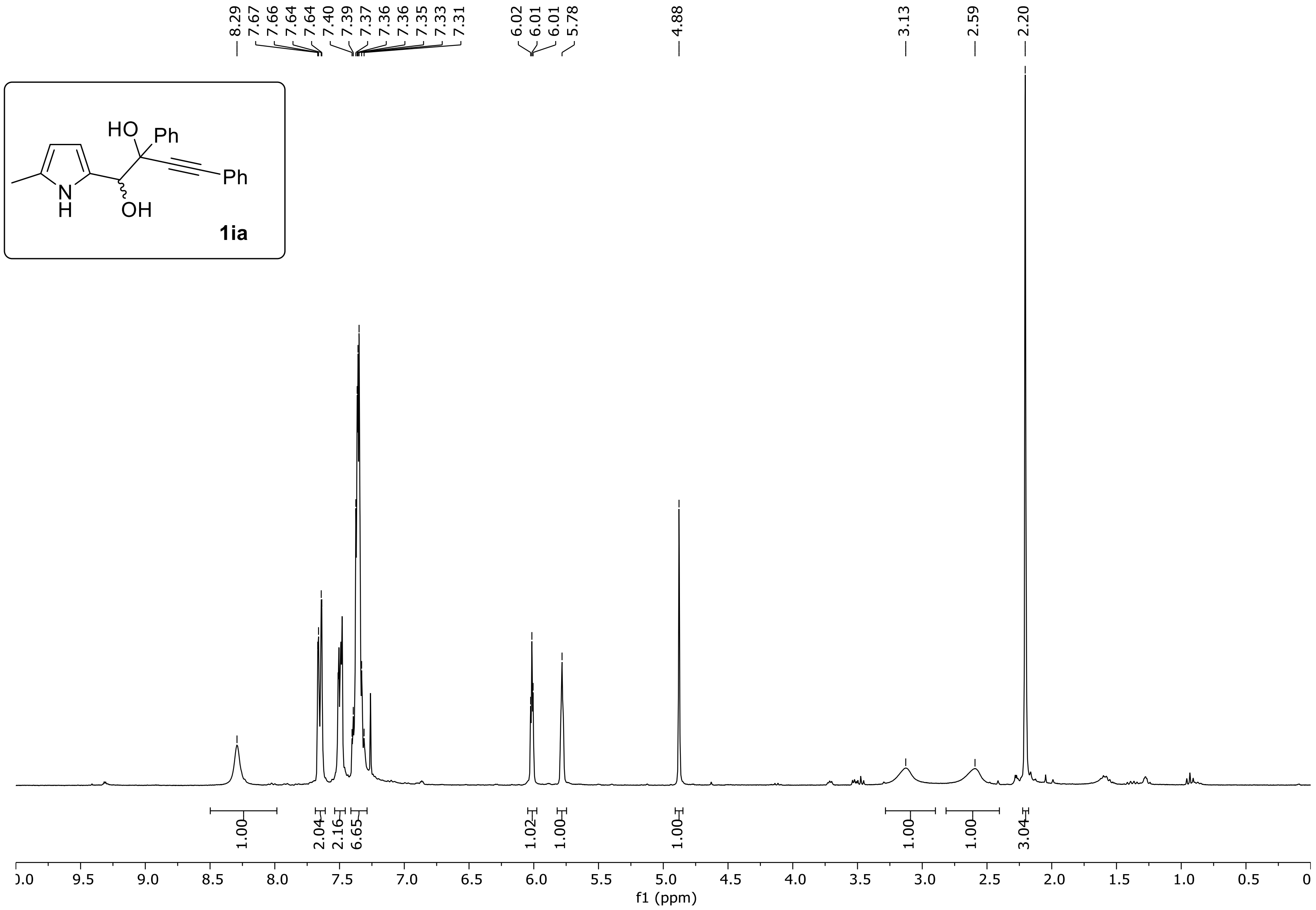

Figure S54: ^{13}C NMR of compound **1ia** in $CDCl_3$ at 75.4 MHz.

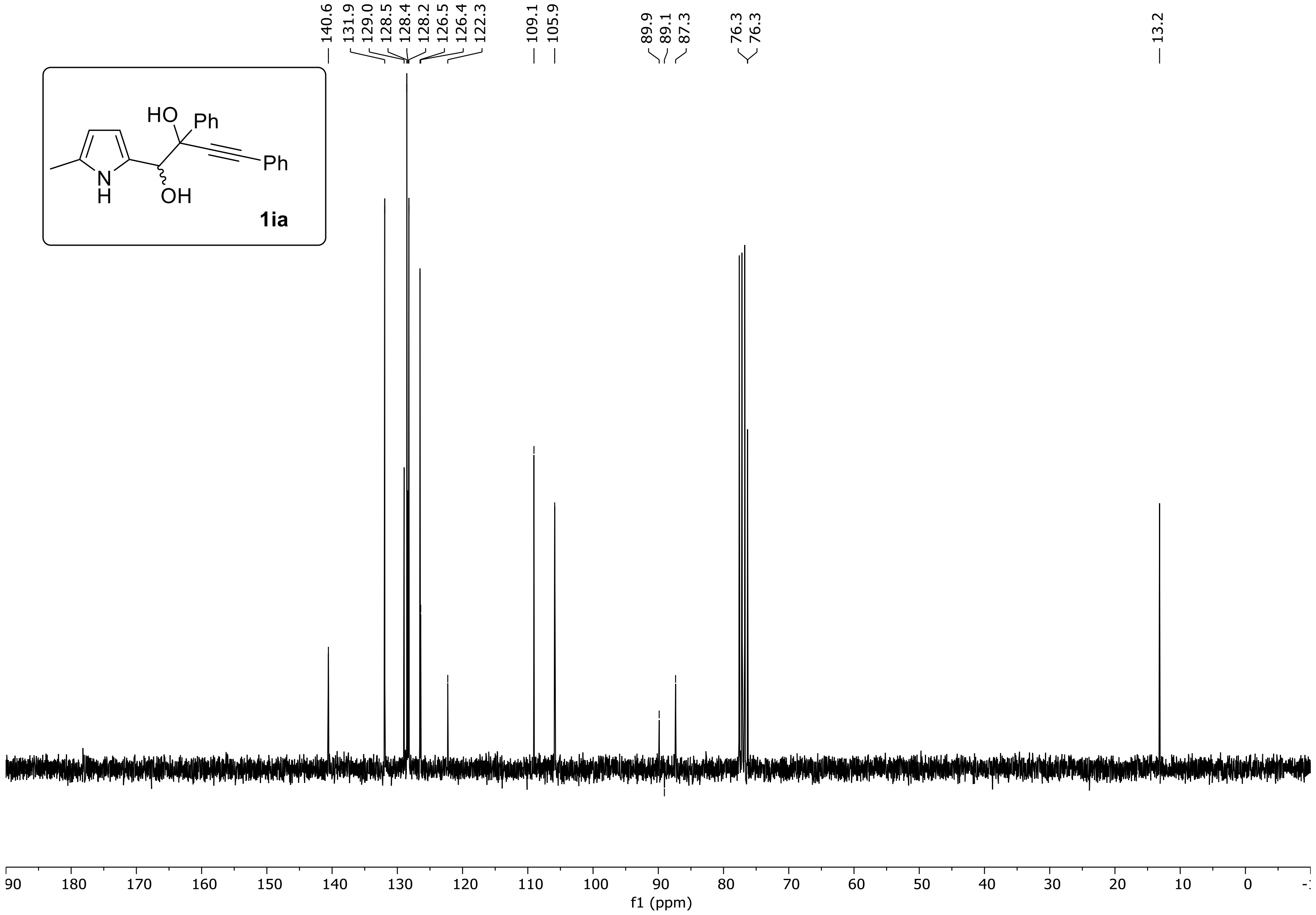

Figure S55: ^{1}H NMR of compound **1ib** in $CDCl_3$ at 300 MHz.

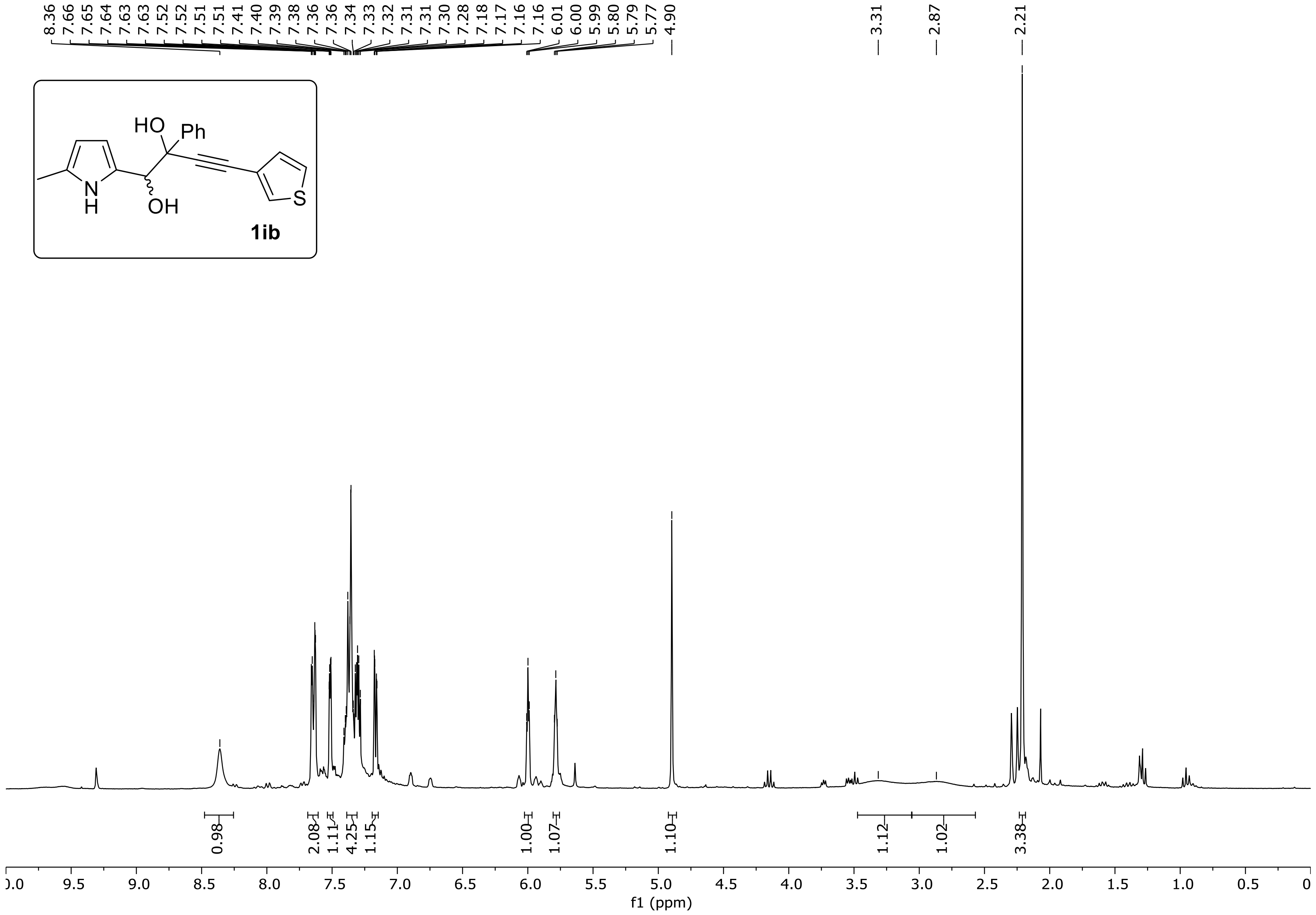

Figure S56: ^{13}C NMR of compound **1ib** in $CDCl_3$ at 75.4 MHz.

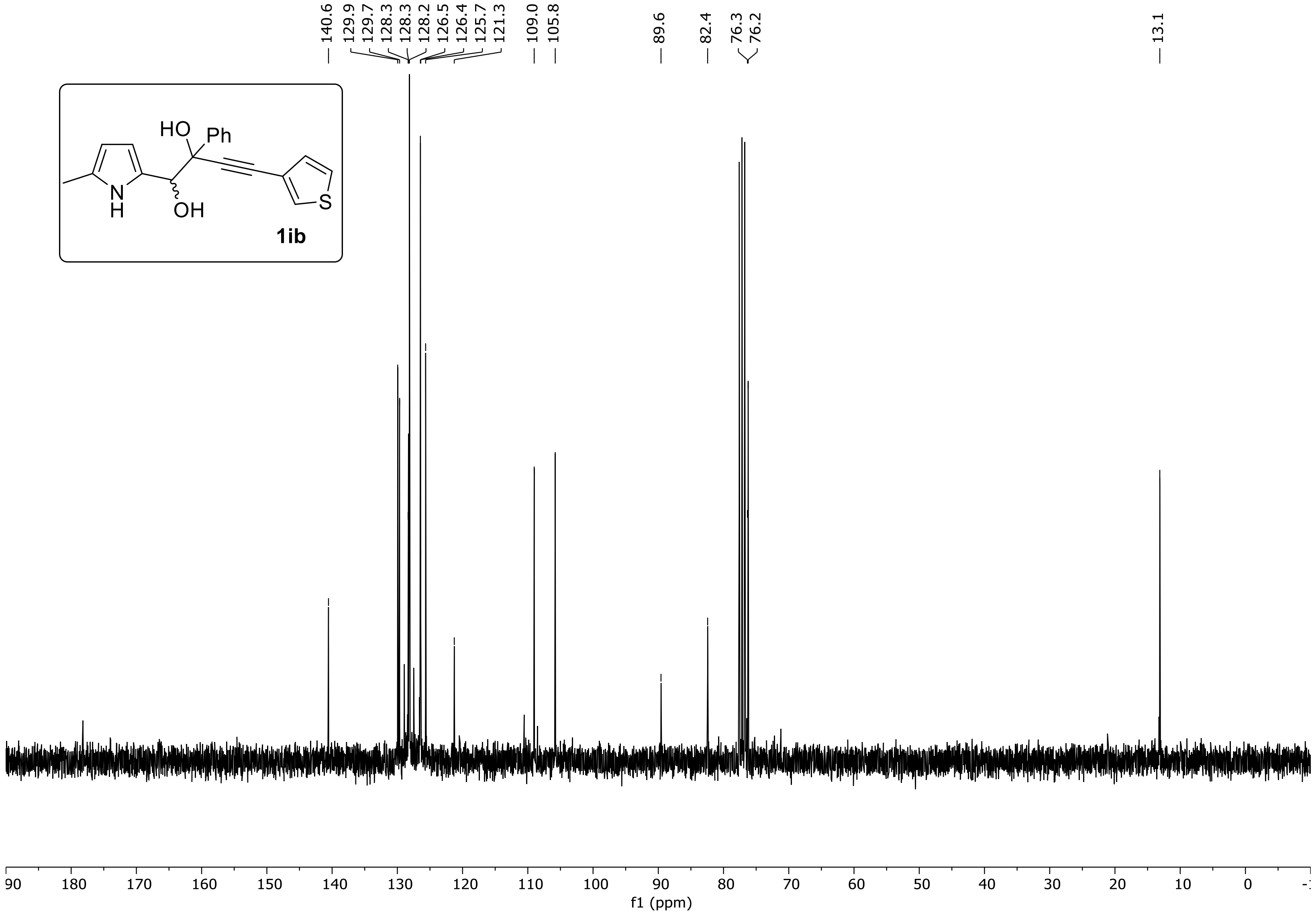

Figure S57: ^{1}H NMR of compound **1jh** in $CDCl_3$ at 300 MHz.

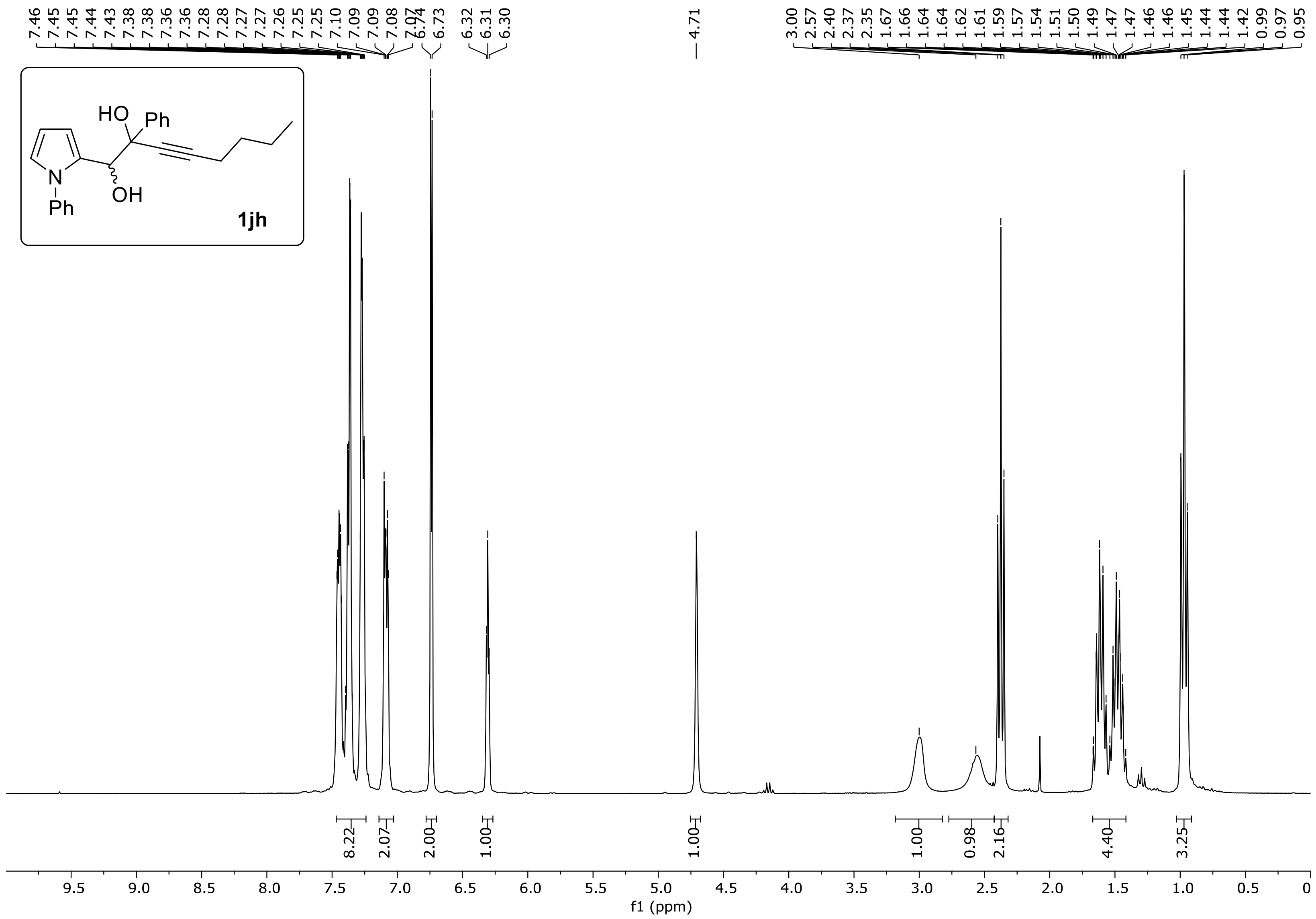

Figure S58: ^{13}C NMR of compound **1jh** in $CDCl_3$ at 75.4 MHz.

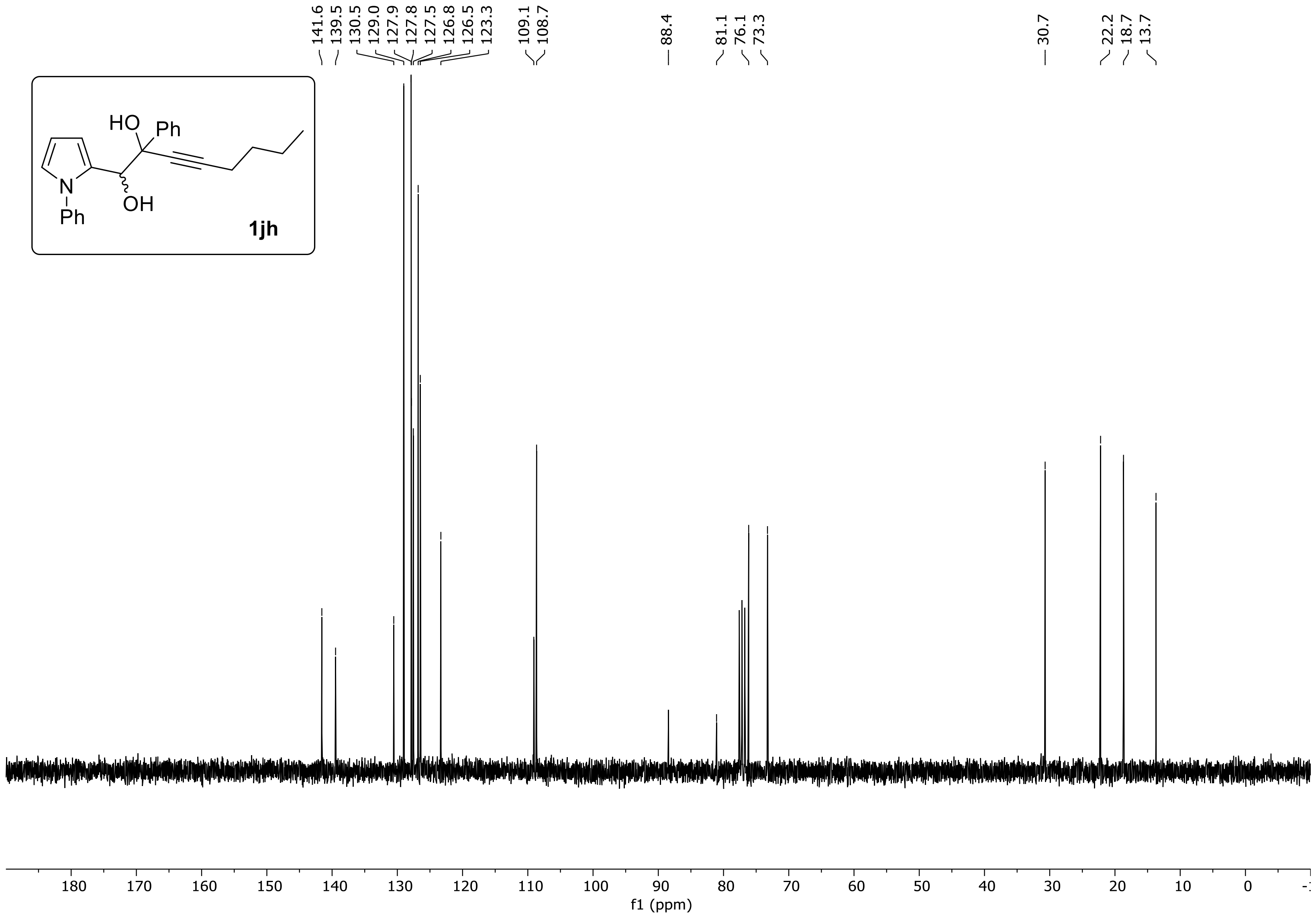

Figure S59: ^{1}H NMR of compound **2aa** in $CDCl_3$ at 300 MHz.

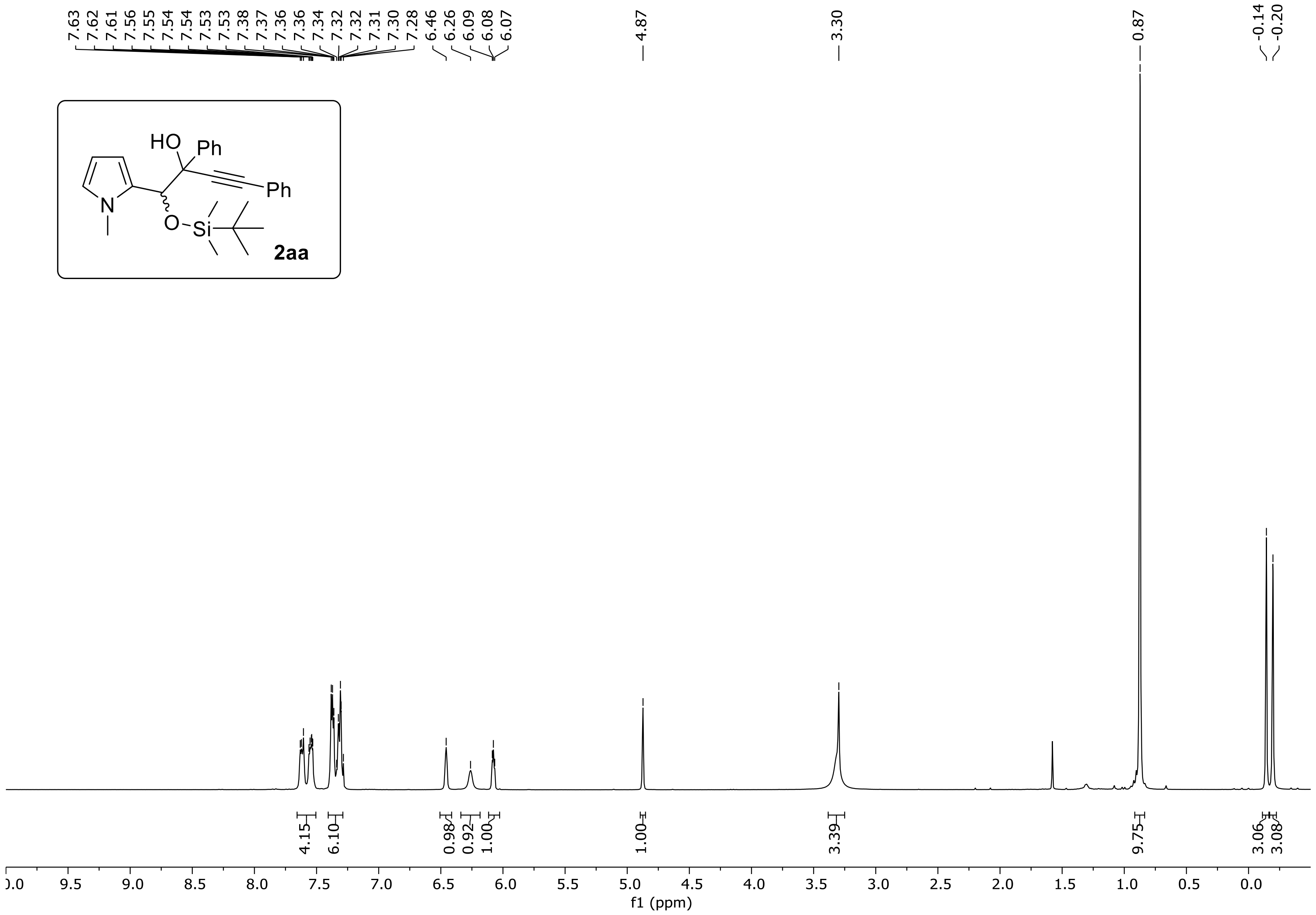

Figure S60: ^{13}C NMR of compound **2aa** in $CDCl_3$ at 75.4 MHz.

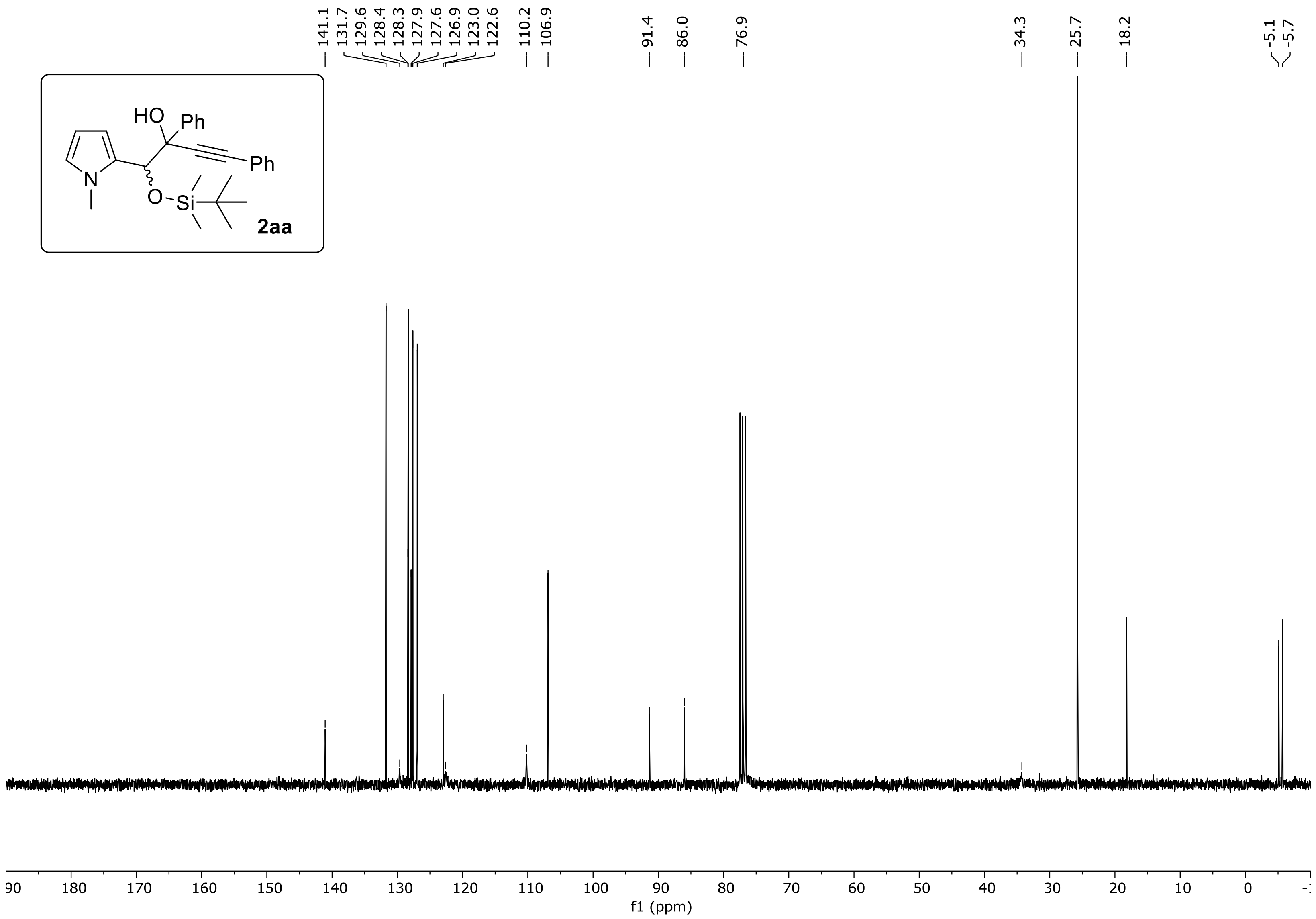

Figure S61: ^{1}H NMR of compound **2´aa** in $CDCl_3$ at 300 MHz.

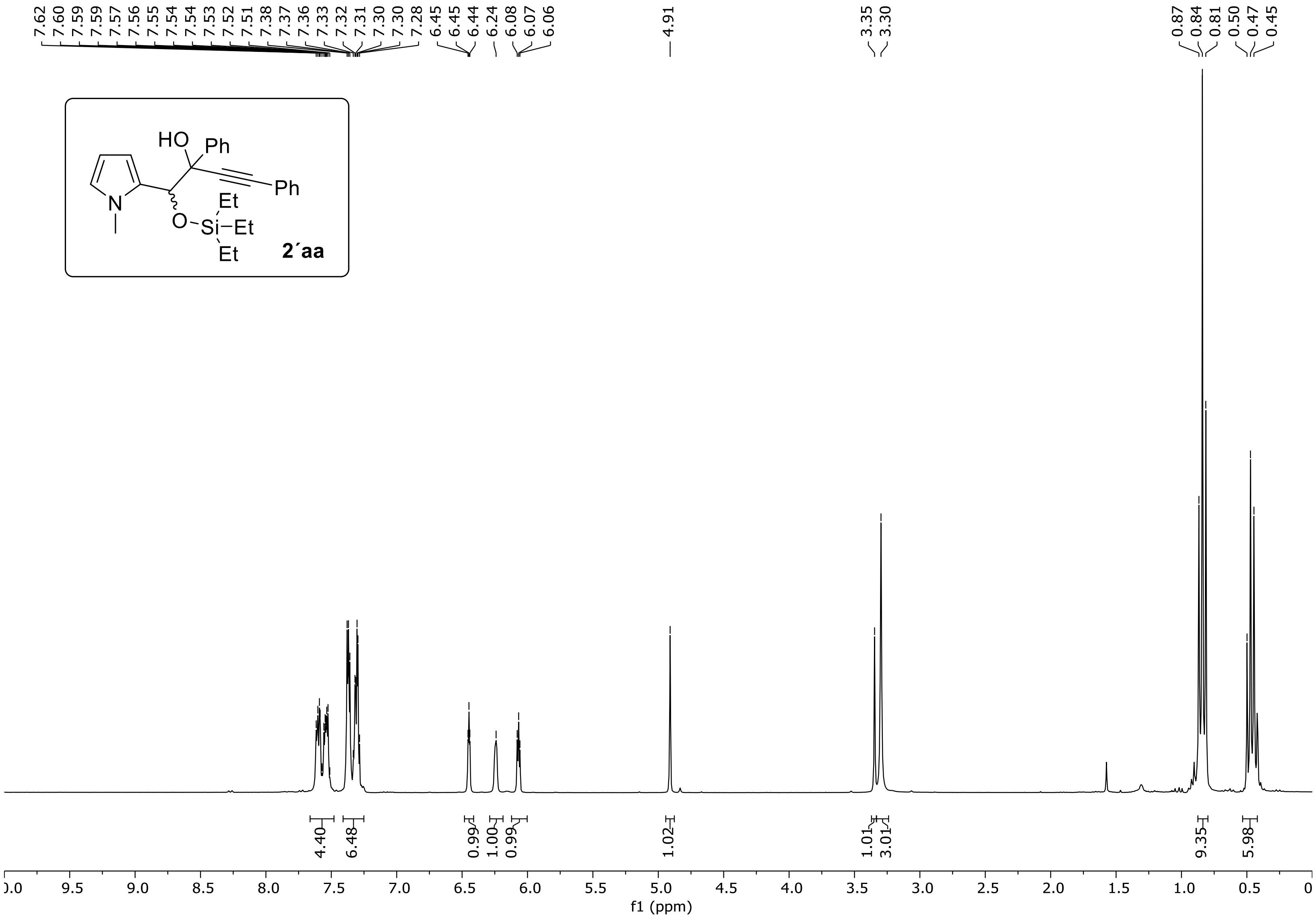

Figure S62: ^{13}C NMR of compound **2´aa** in $CDCl_3$ at 75.4 MHz.

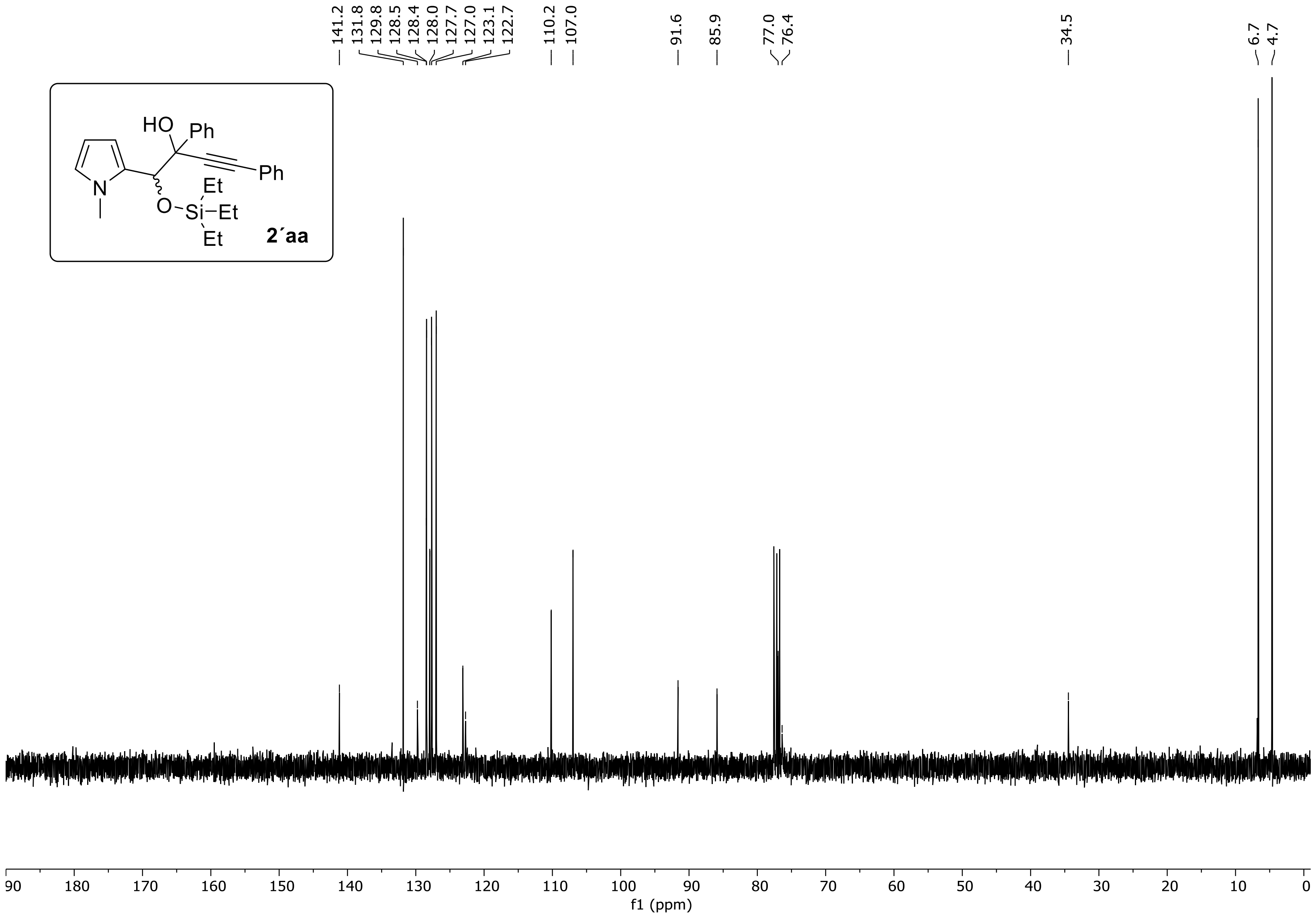

Figure S63: ^{1}H NMR of compound **2´´aa** in $CDCl_3$ at 300 MHz.

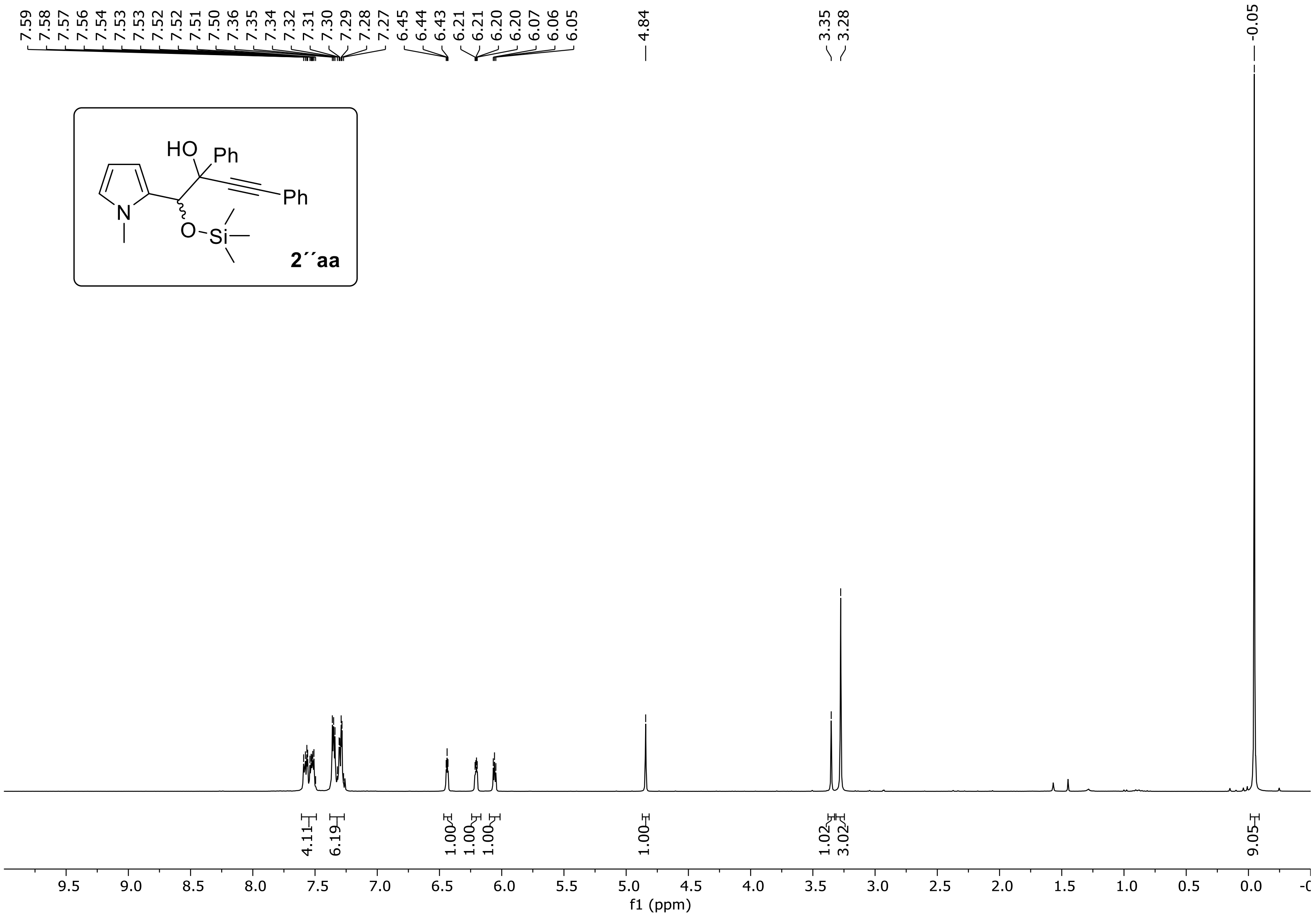

Figure S64: ^{13}C NMR of compound **2´´aa** in $CDCl_3$ at 75.4 MHz.

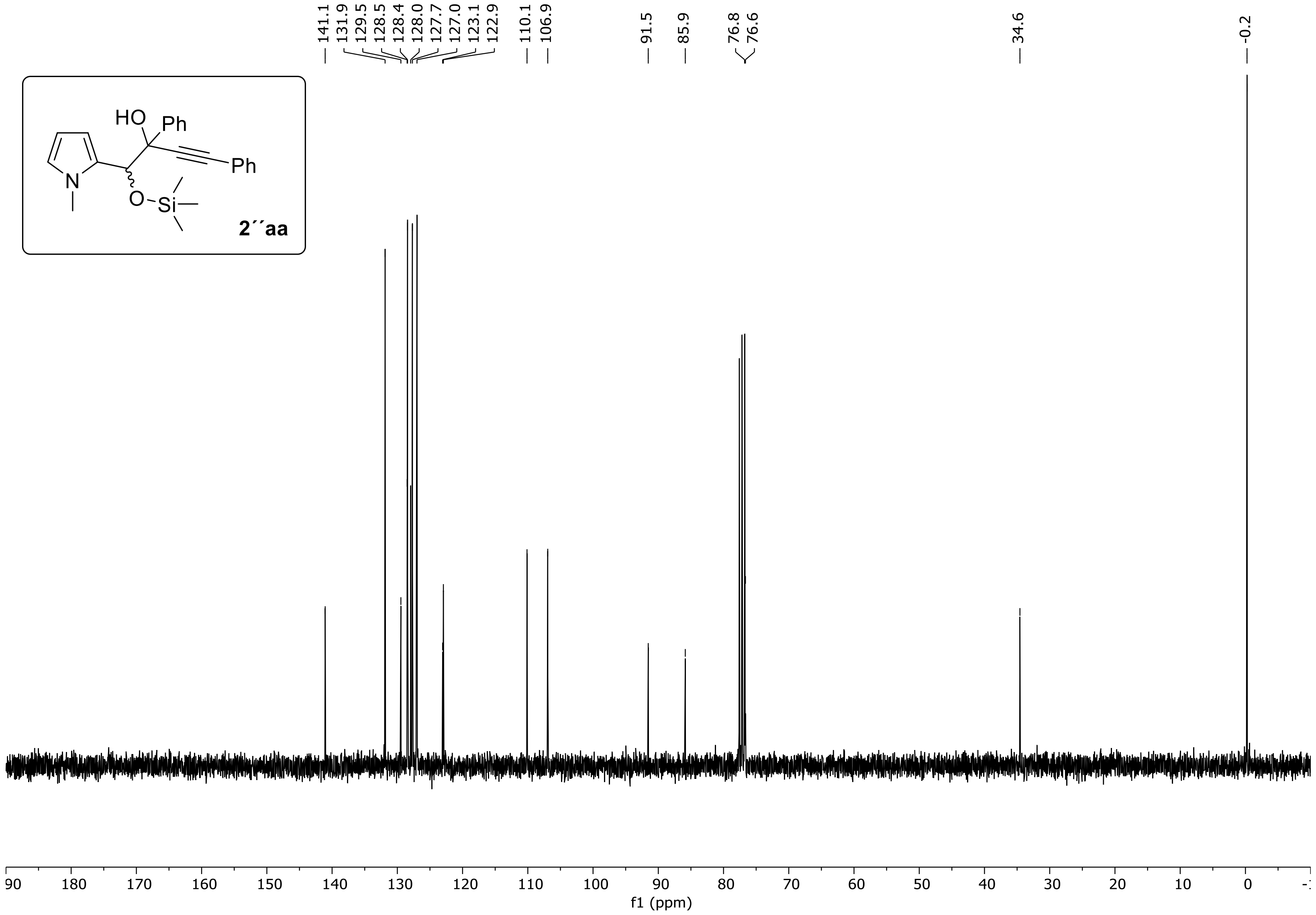

Figure S65: ^{1}H NMR of compound **2ab** in $CDCl_3$ at 300 MHz.

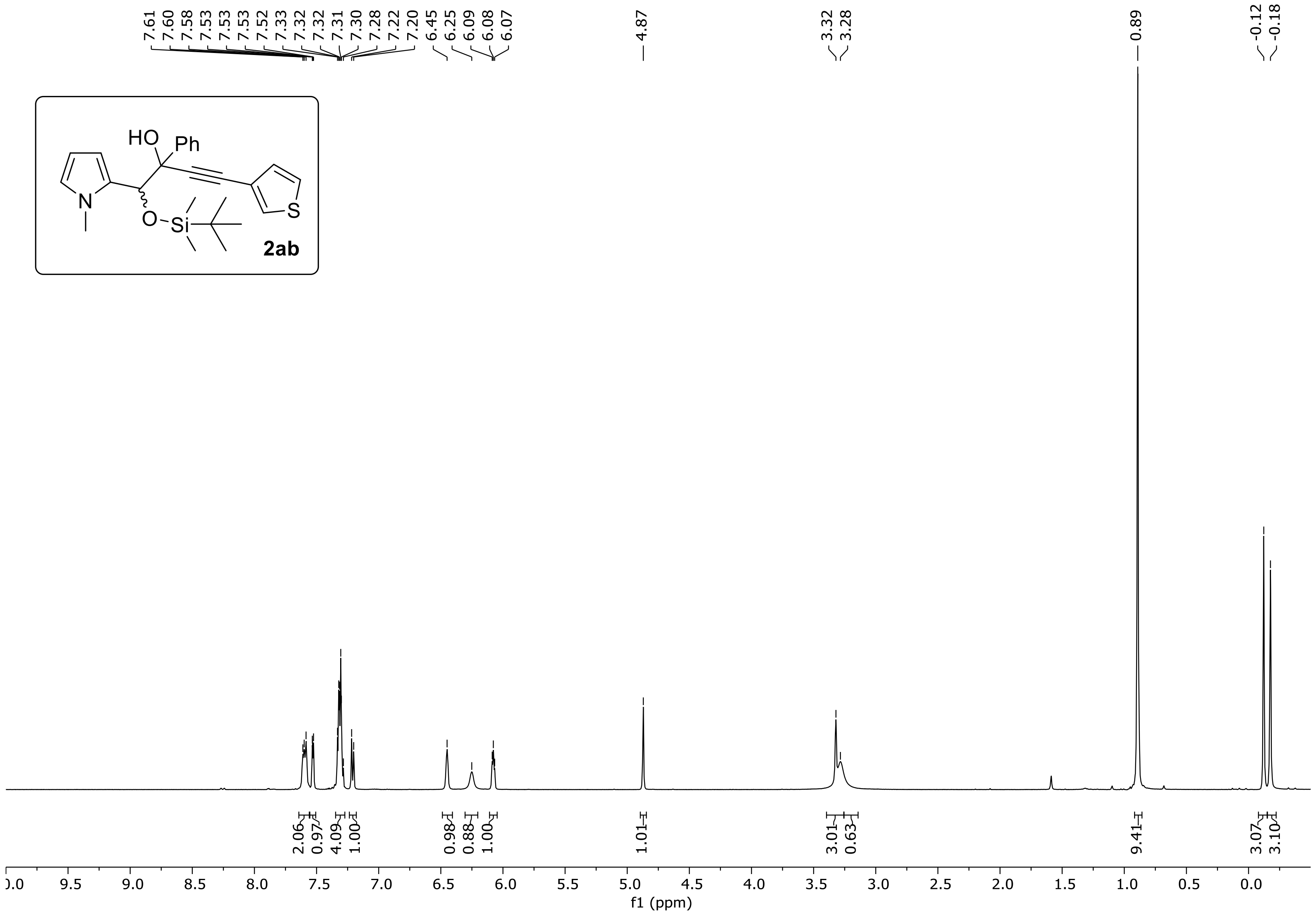

Figure S66: ^{13}C NMR of compound **2ab** in $CDCl_3$ at 75.4 MHz.

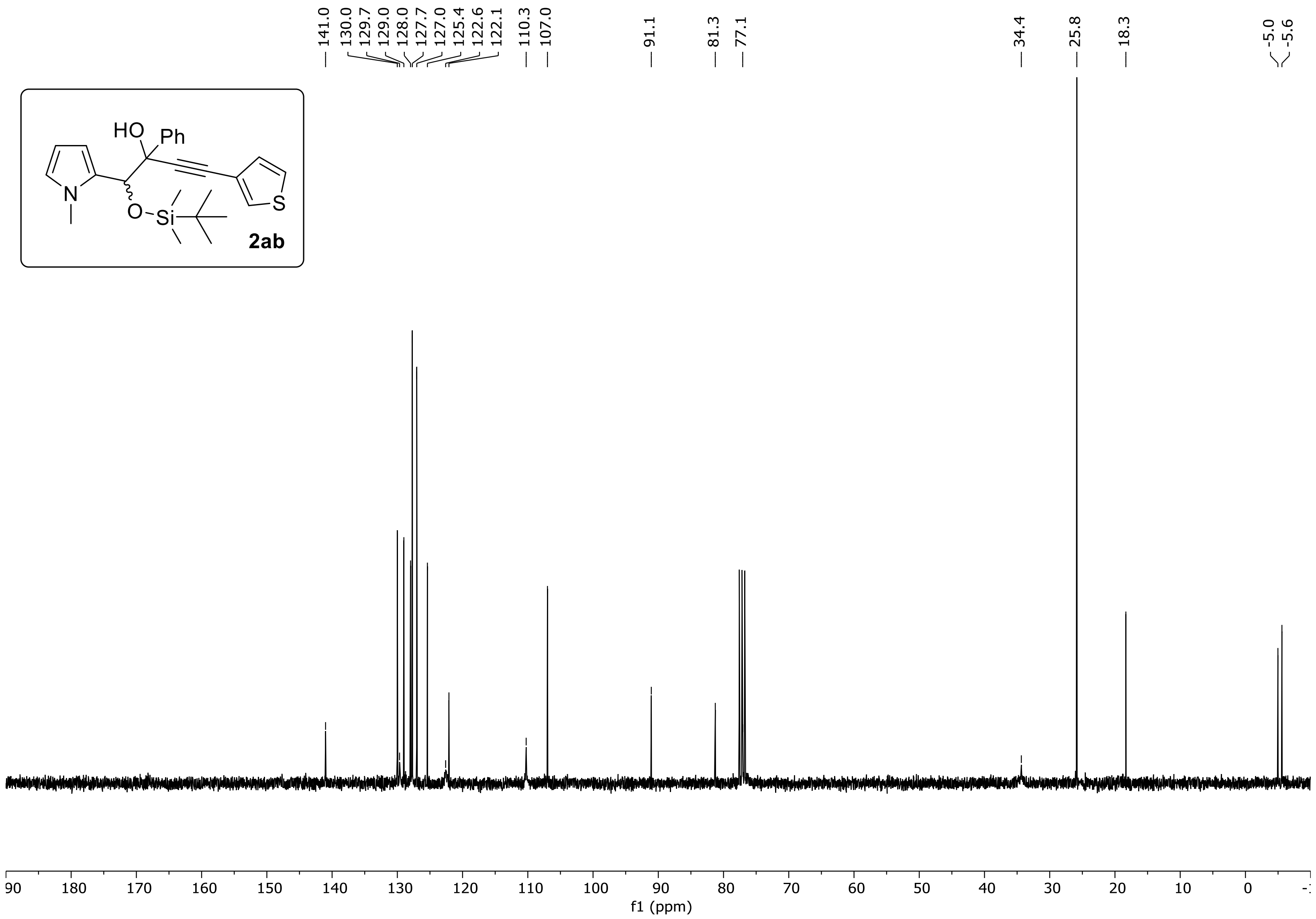

Figure S67: ^{1}H NMR of compound **2ac** in $CDCl_3$ at 300 MHz.

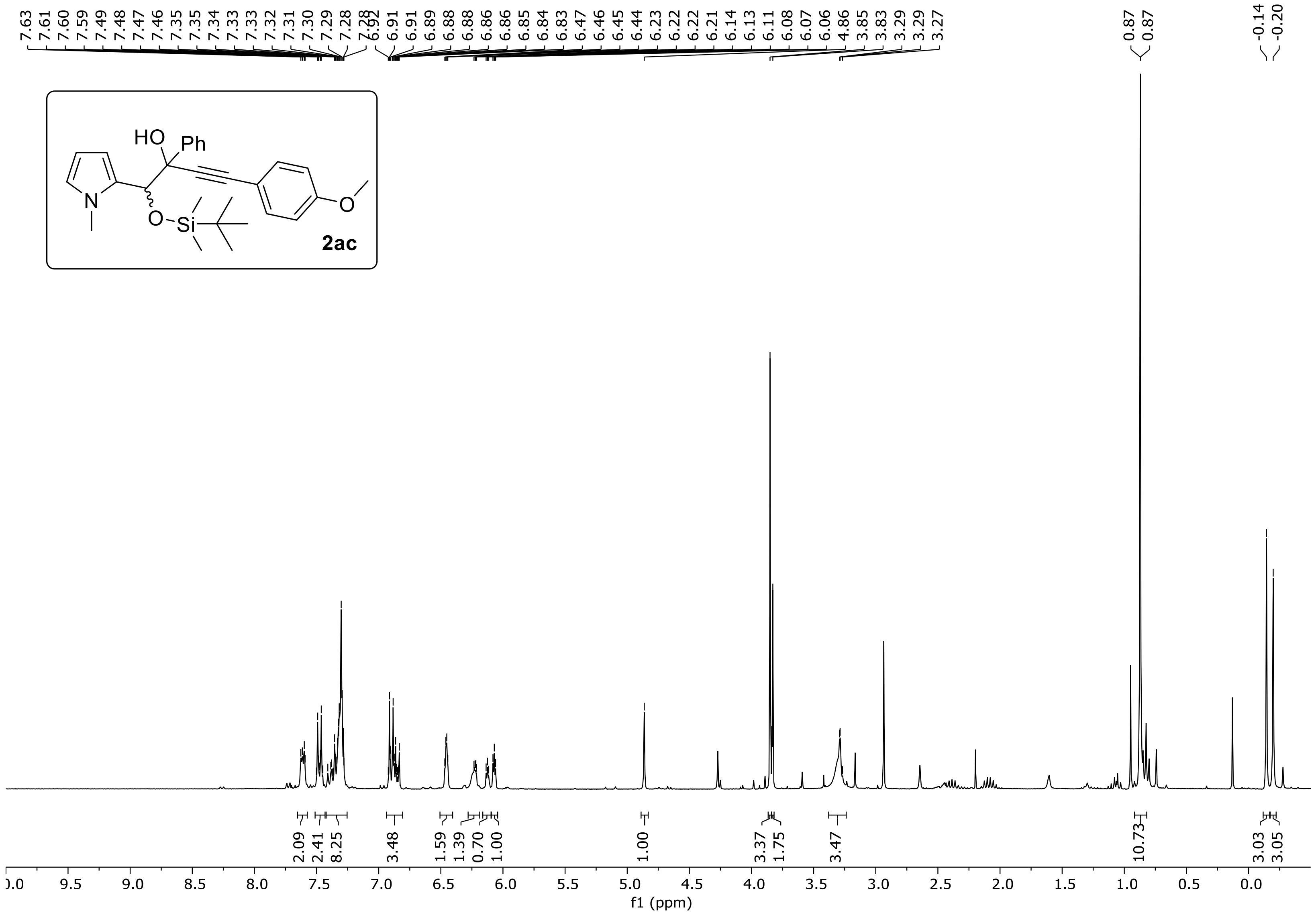

Figure S68: ^{13}C NMR of compound **2ac** in $CDCl_3$ at 75.4 MHz.

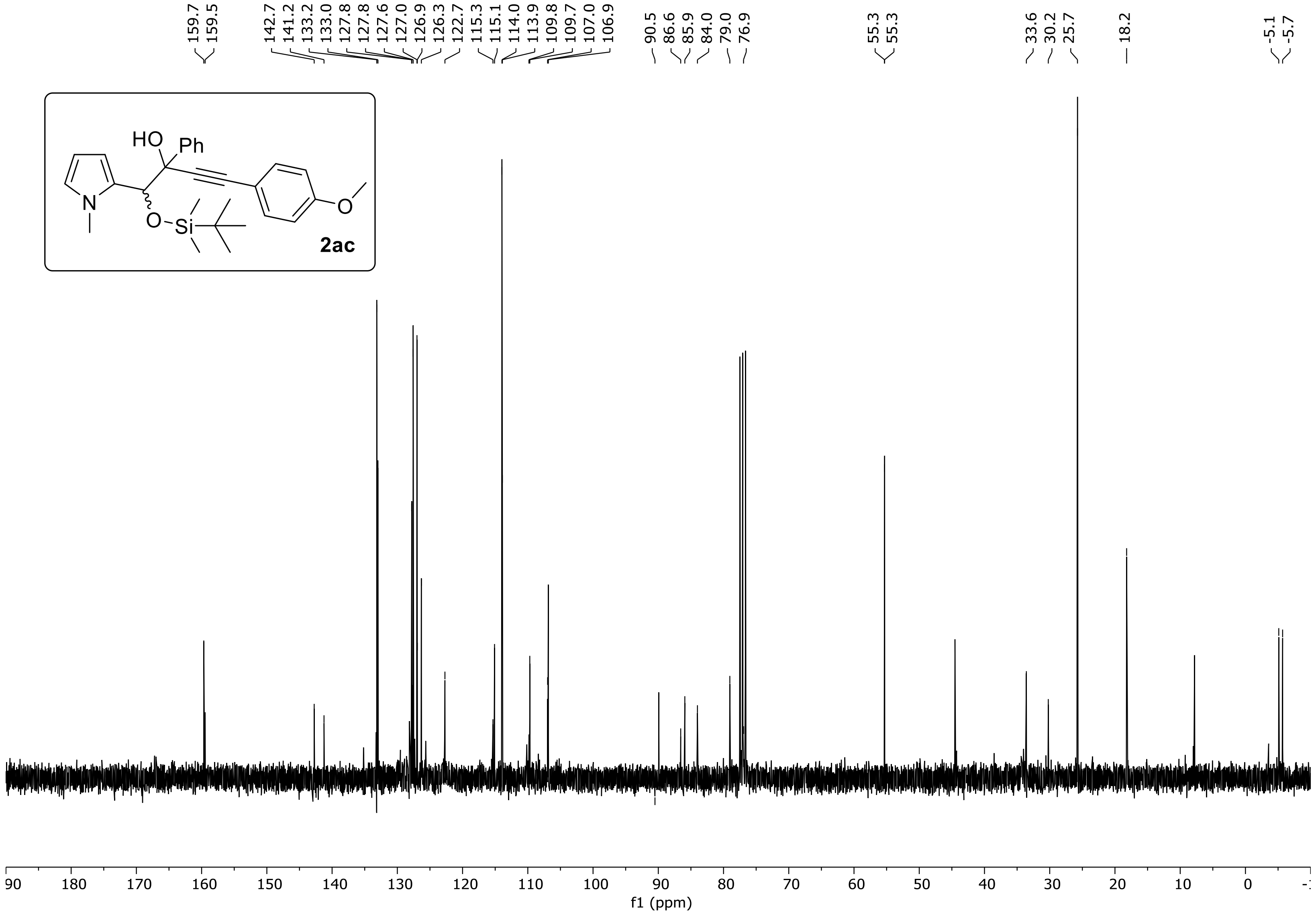

Figure S69: ^{1}H NMR of compound **2ad** in $CDCl_3$ at 300 MHz.

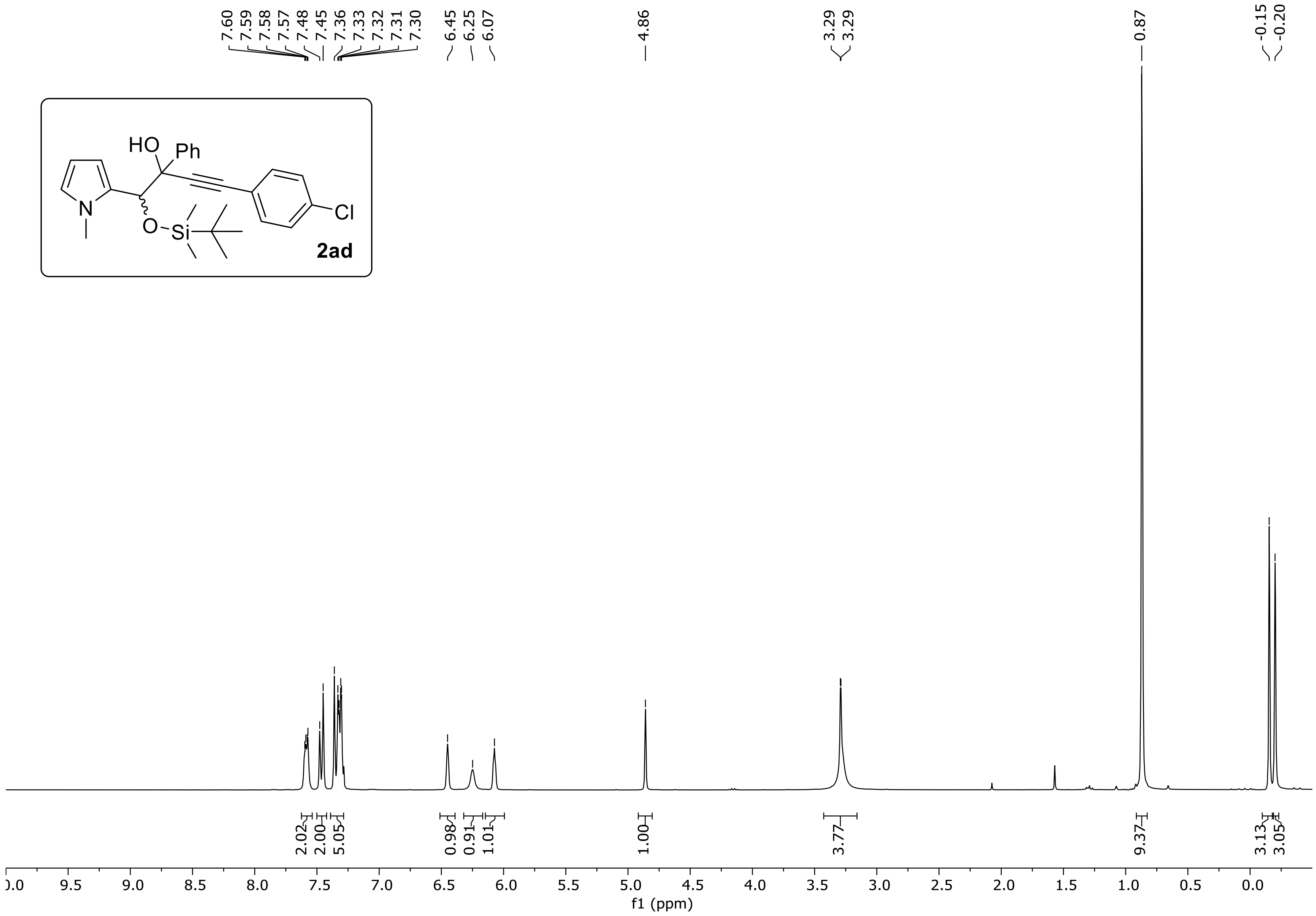

Figure S70: ^{13}C NMR of compound **2ad** in $CDCl_3$ at 75.4 MHz.

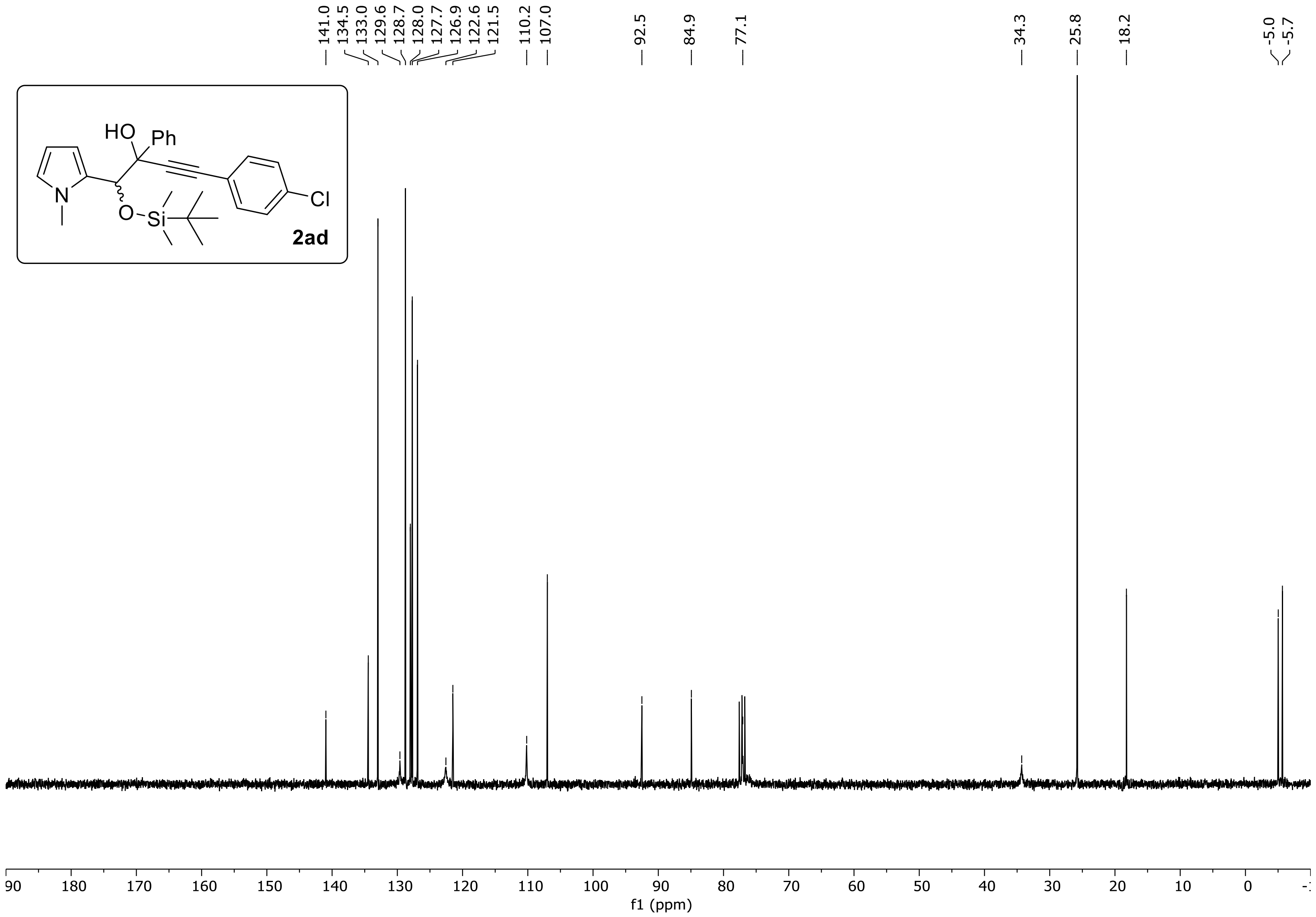

Figure S71: ^{1}H NMR of compound **2ae** in $CDCl_3$ at 300 MHz.

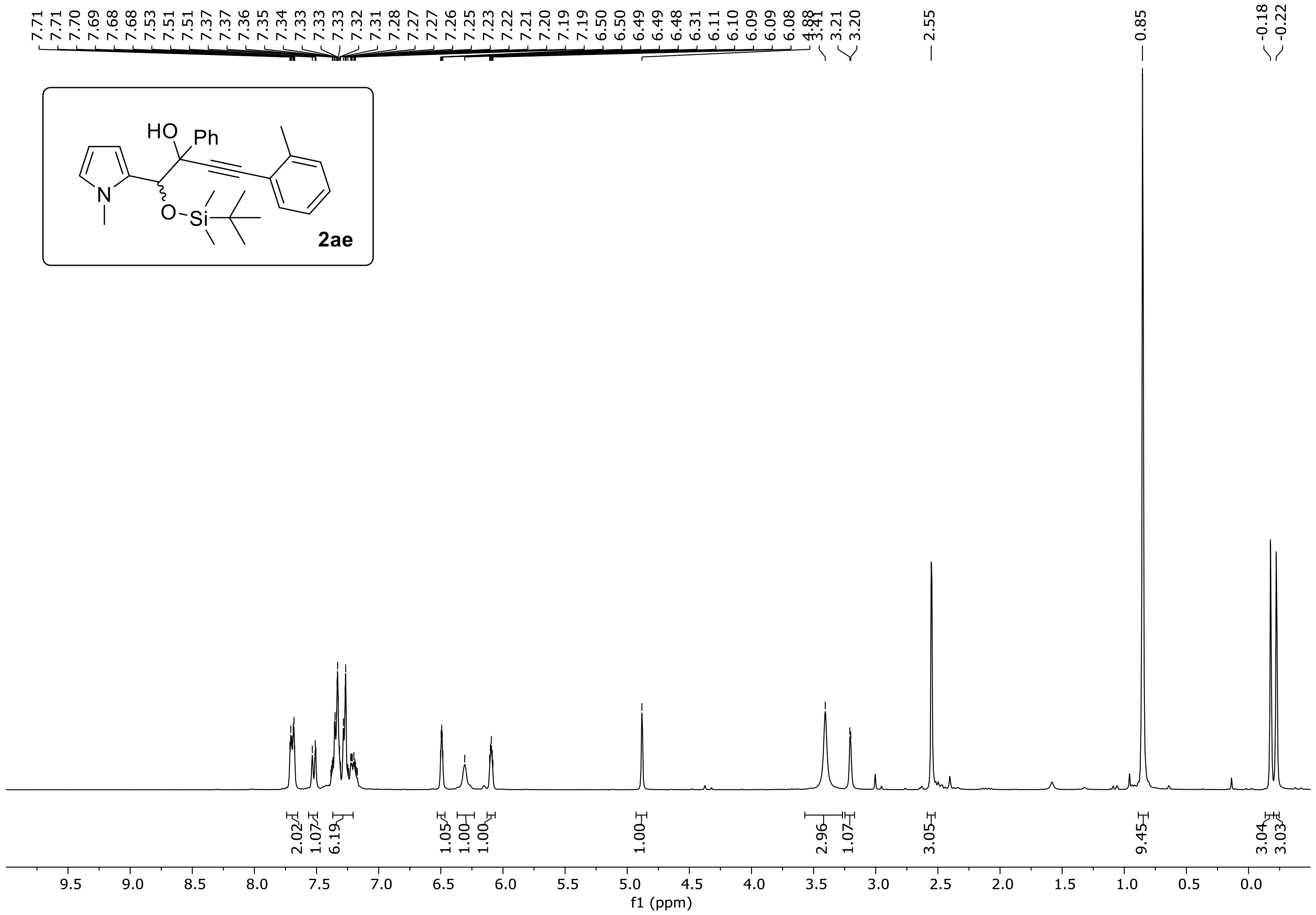

Figure S72: ^{13}C NMR of compound **2ae** in $CDCl_3$ at 75.4 MHz.

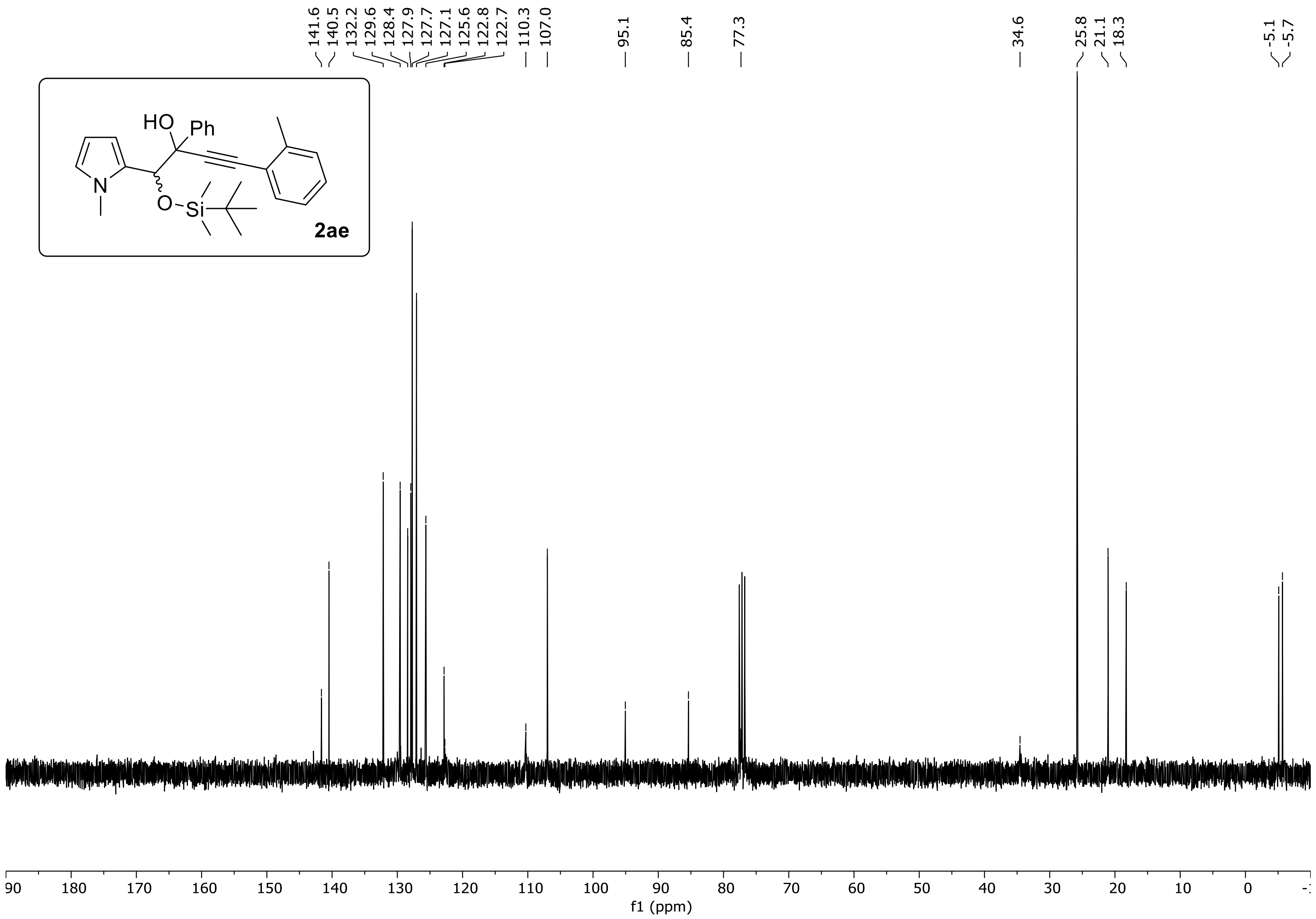

Figure S73: ^{1}H NMR of compound **2af** in $CDCl_3$ at 300 MHz.

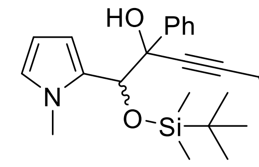

2af

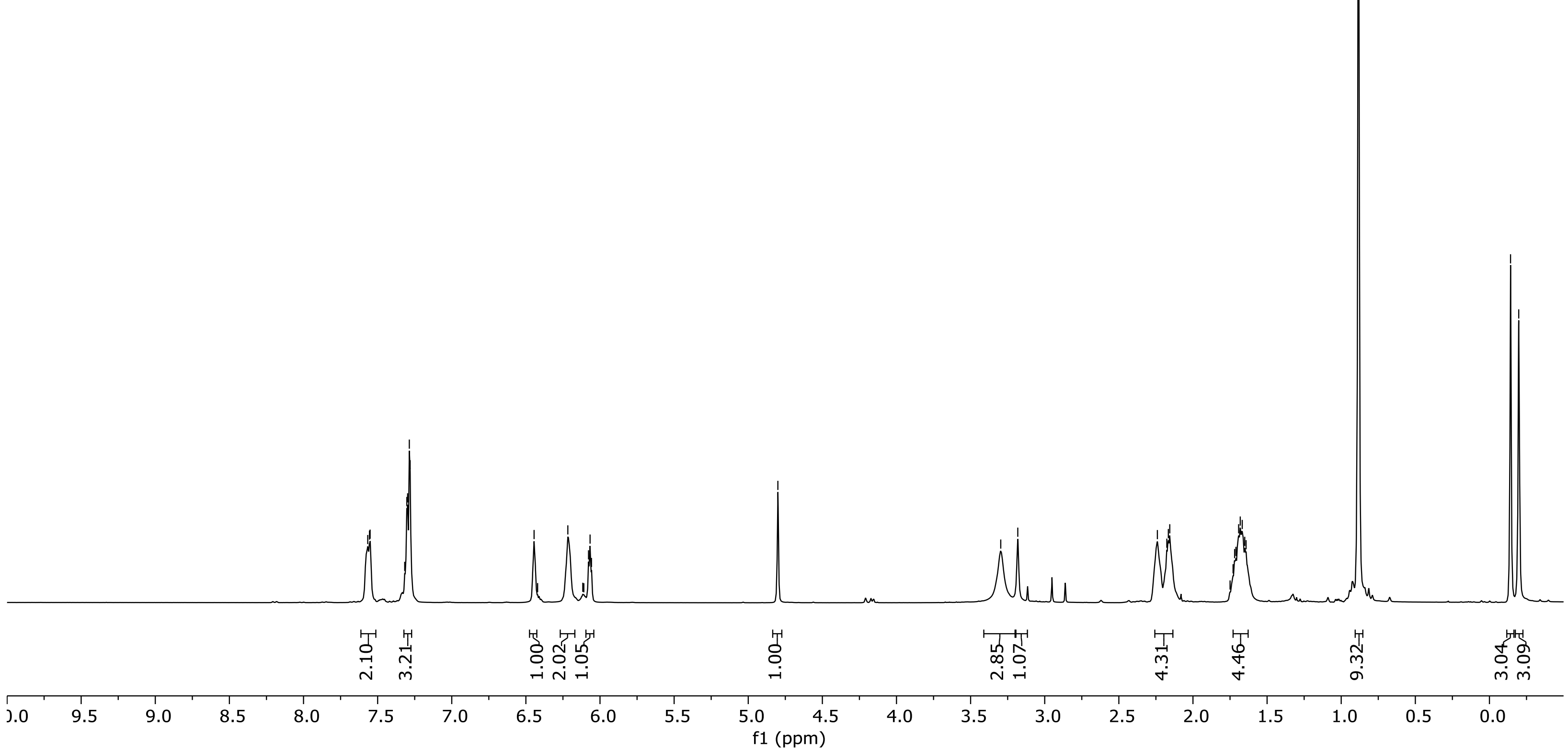

Figure S74: ^{13}C NMR of compound **2af** in $CDCl_3$ at 75.4 MHz.

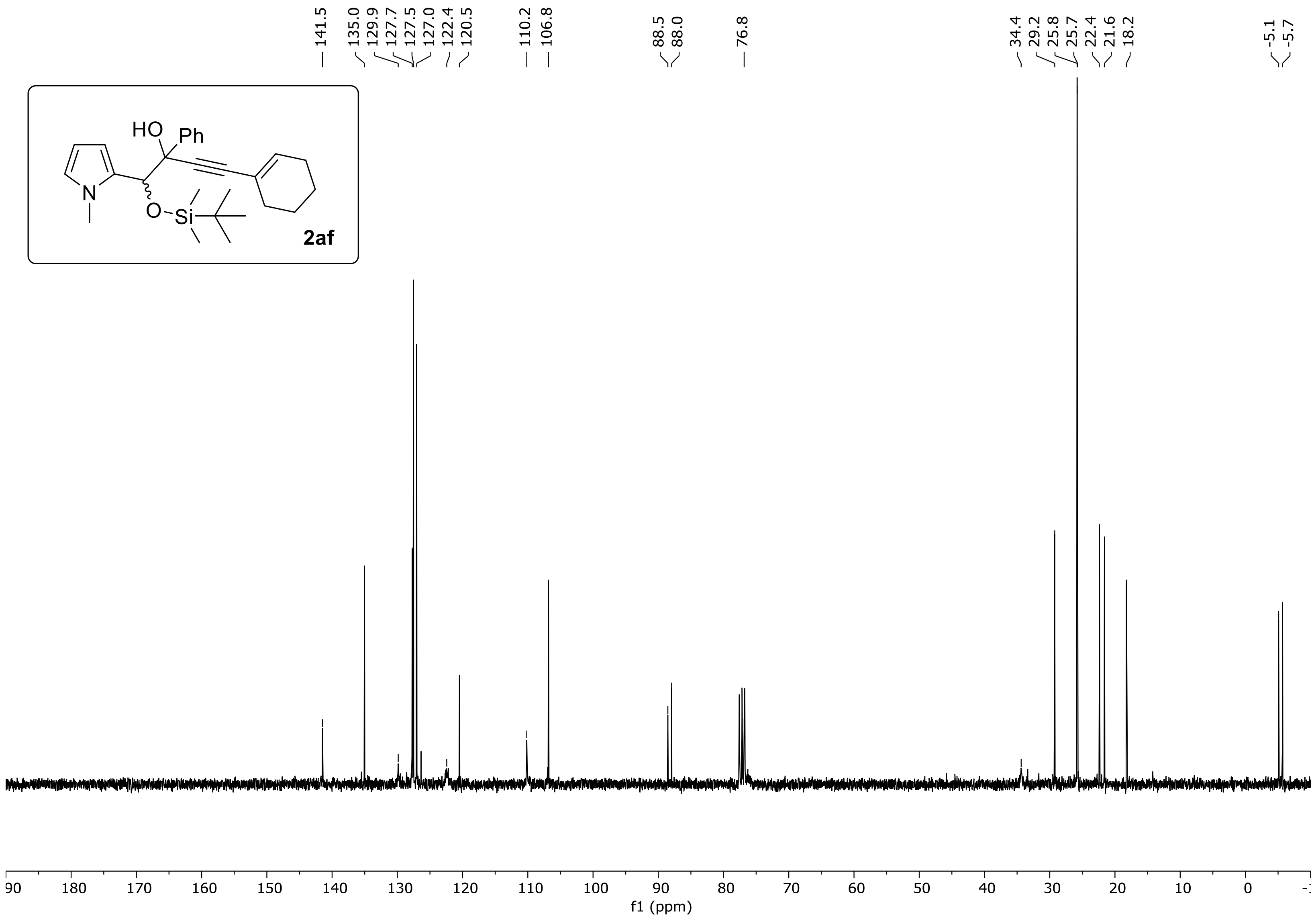

Figure S75: ^{1}H NMR of compound **2ag** in $CDCl_3$ at 300 MHz.

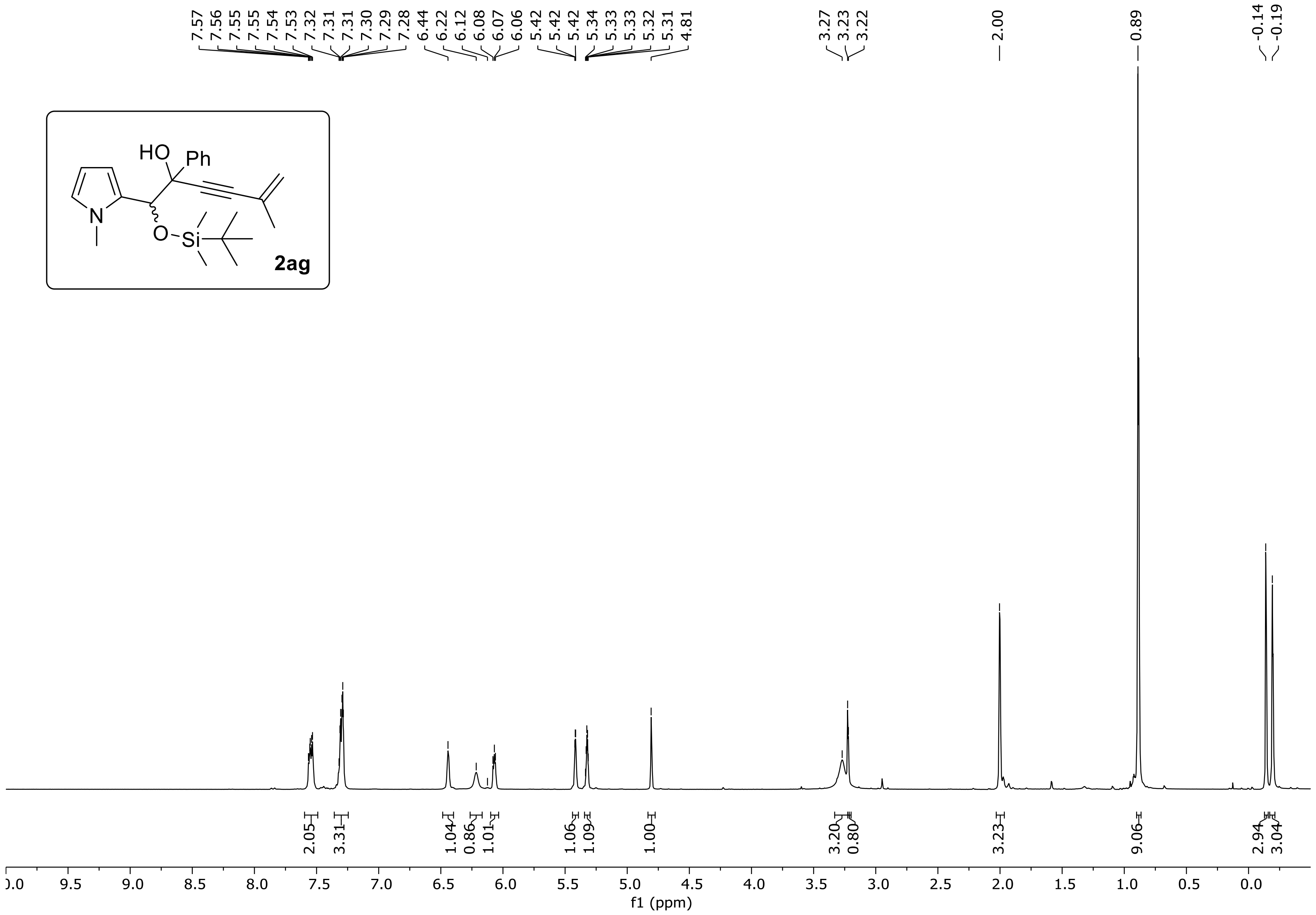

Figure S76: ^{13}C NMR of compound **2ag** in $CDCl_3$ at 75.4 MHz.

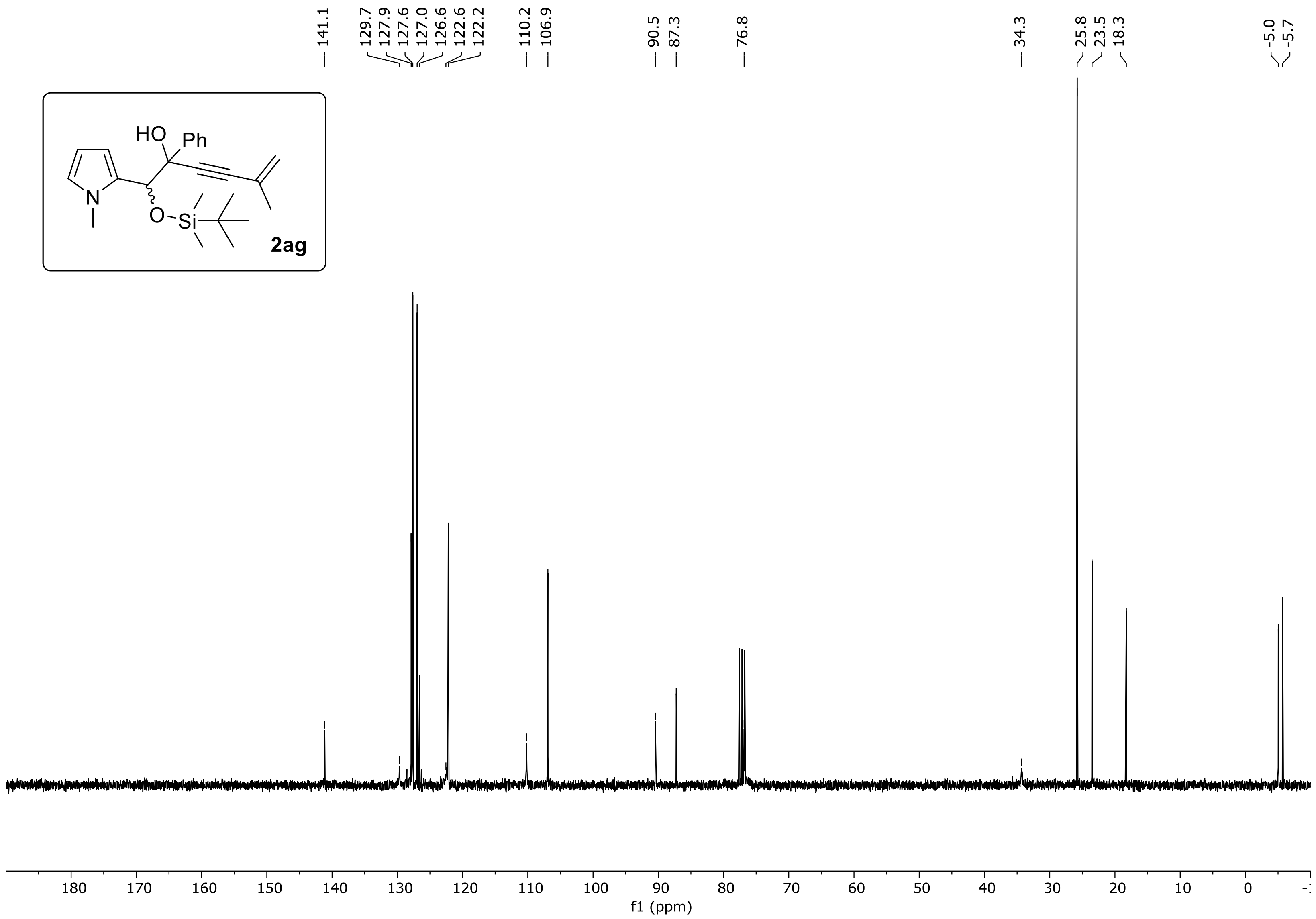

Figure S77: ^{1}H NMR of compound **2ah** in $CDCl_3$ at 300 MHz.

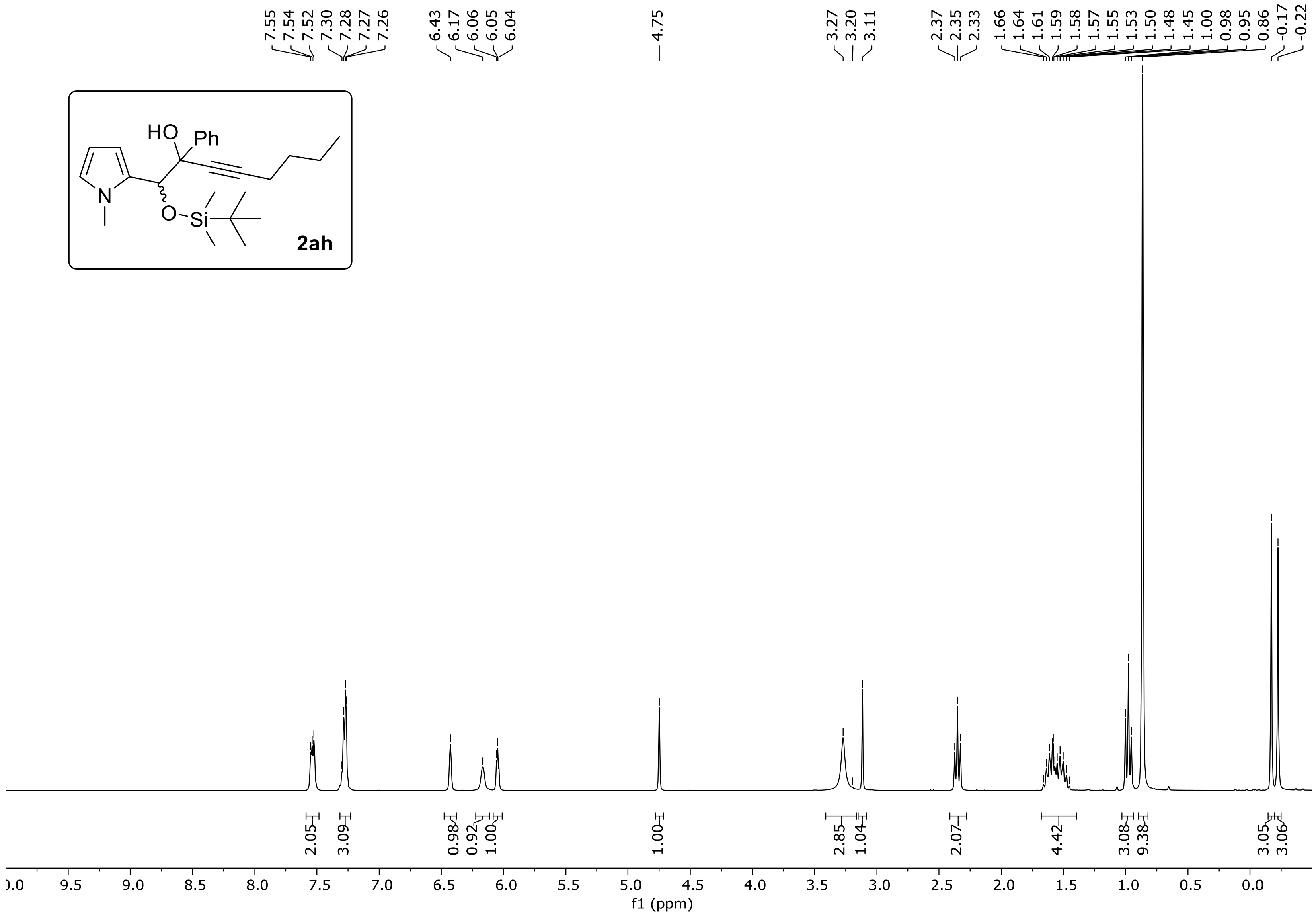

Figure S78: ^{13}C NMR of compound **2ah** in $CDCl_3$ at 75.4 MHz.

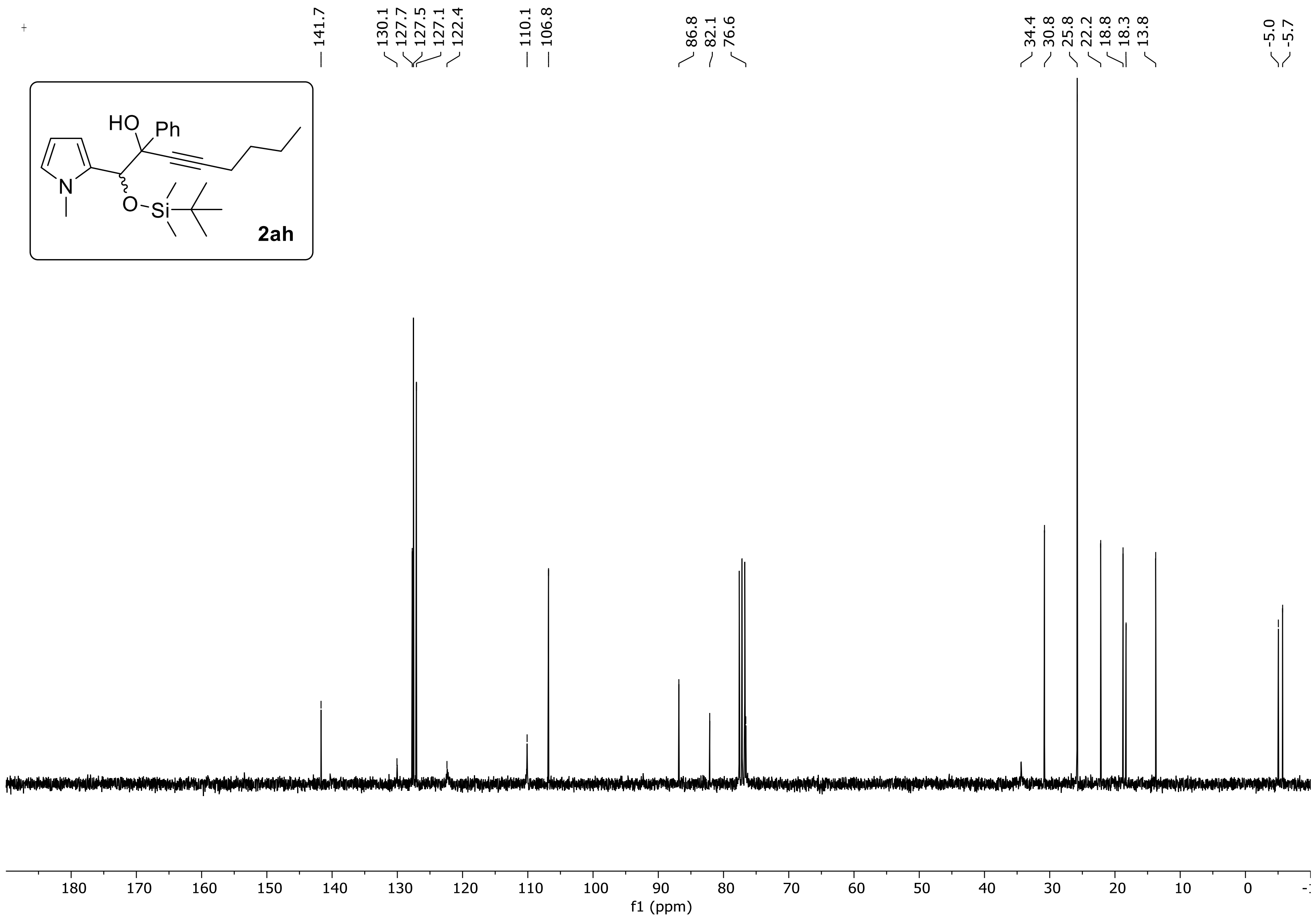

Figure S79: ^{1}H NMR of compound **2ai** in $CDCl_3$ at 300 MHz.

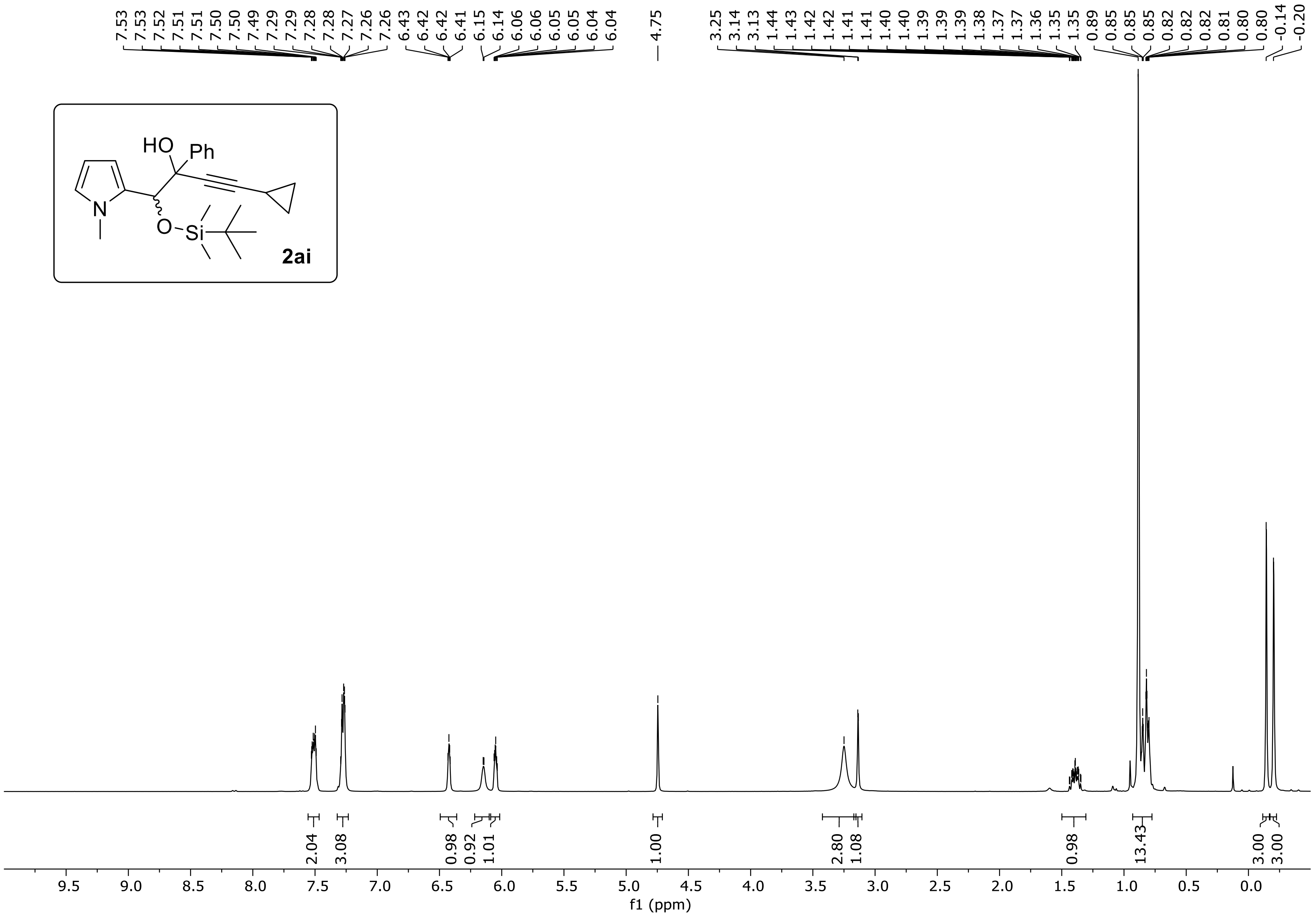

Figure S80: ^{13}C NMR of compound **2ai** in $CDCl_3$ at 75.4 MHz.

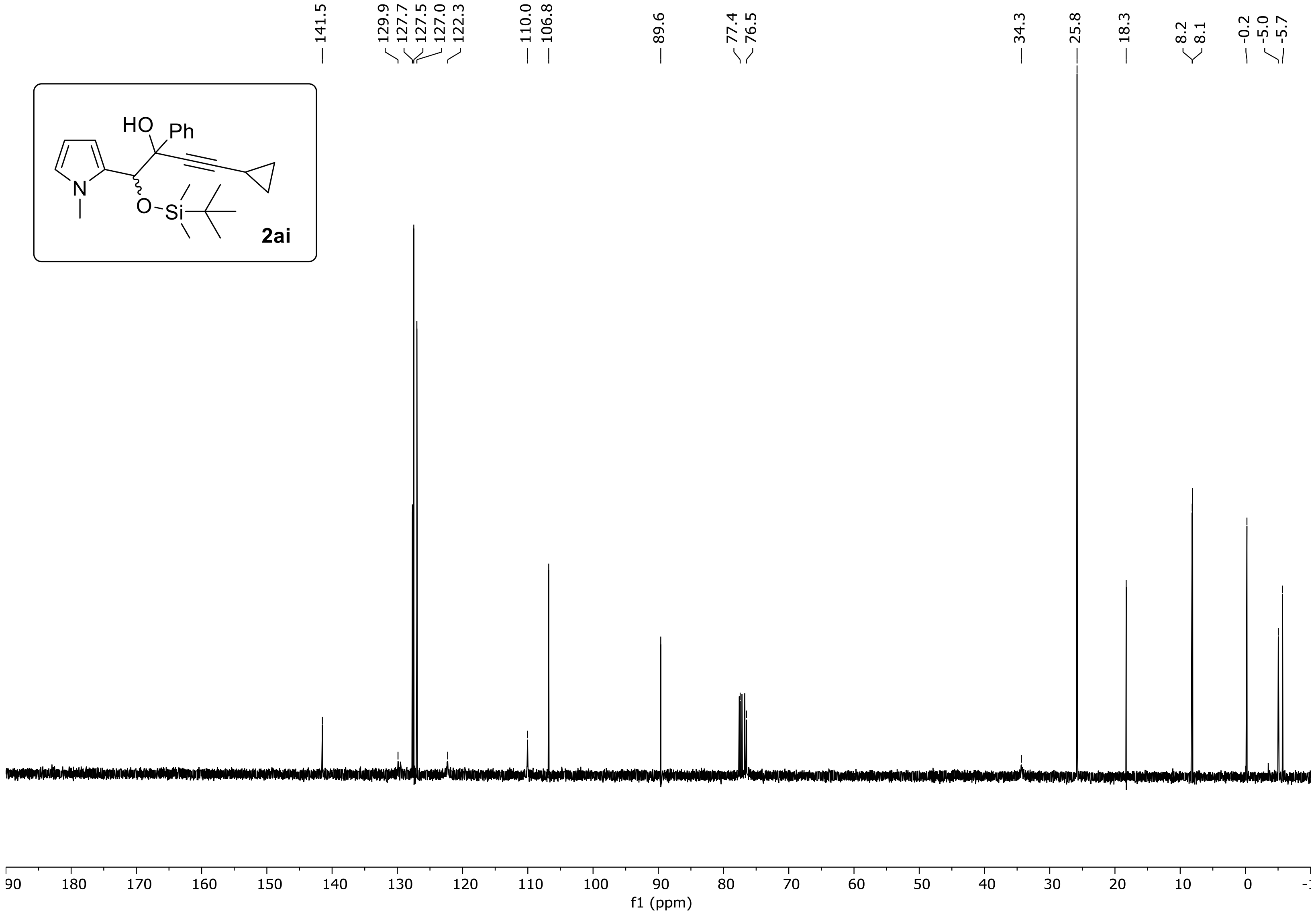

Figure S81: ^{1}H NMR of compound **2aj** in $CDCl_3$ at 300 MHz.

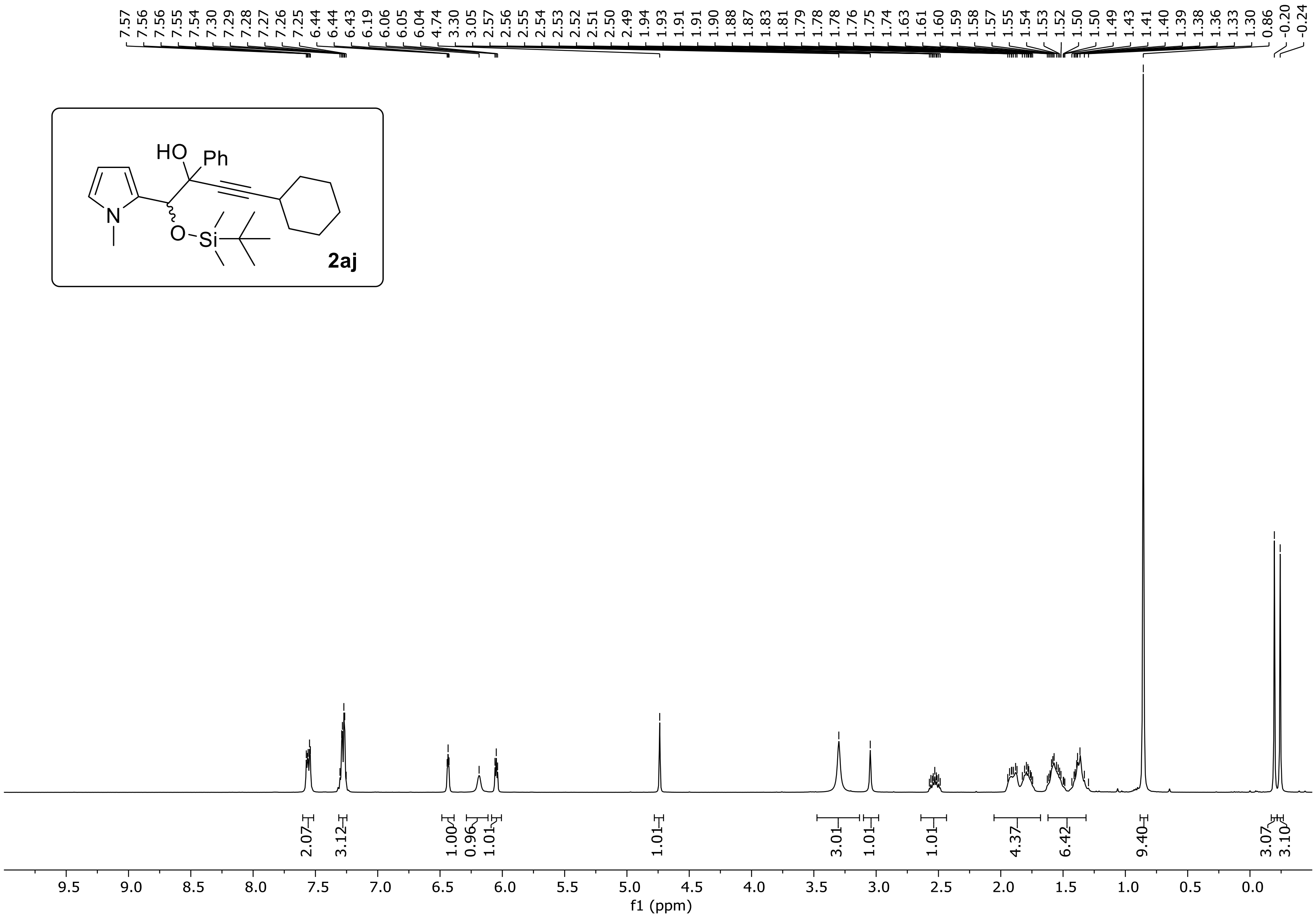

Figure S82: ^{13}C NMR of compound **2aj** in $CDCl_3$ at 75.4 MHz.

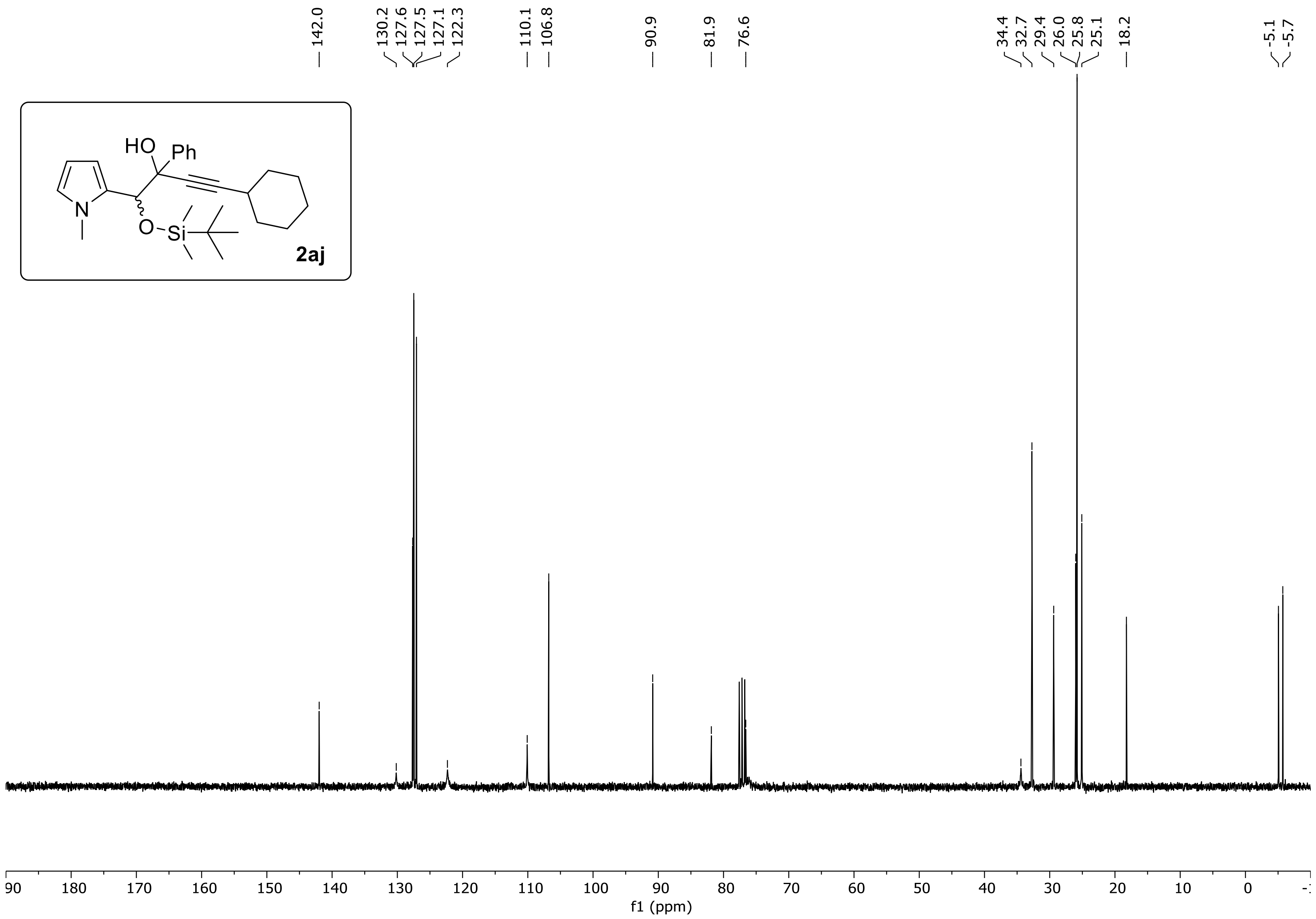

Figure S83: ^{1}H NMR of compound **2ak** in $CDCl_3$ at 300 MHz.

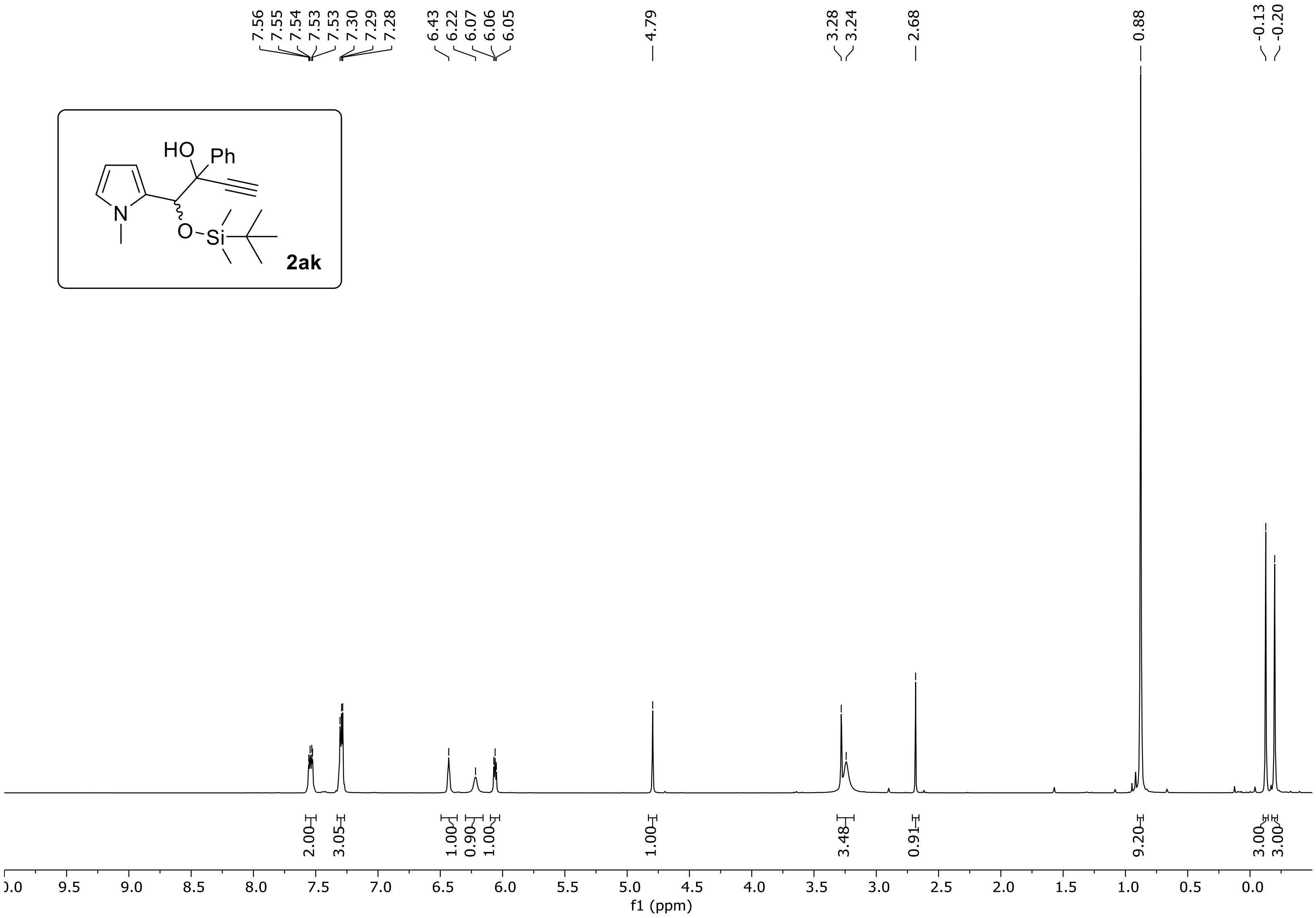

Figure S84: ^{13}C NMR of compound **2ak** in $CDCl_3$ at 75.4 MHz.

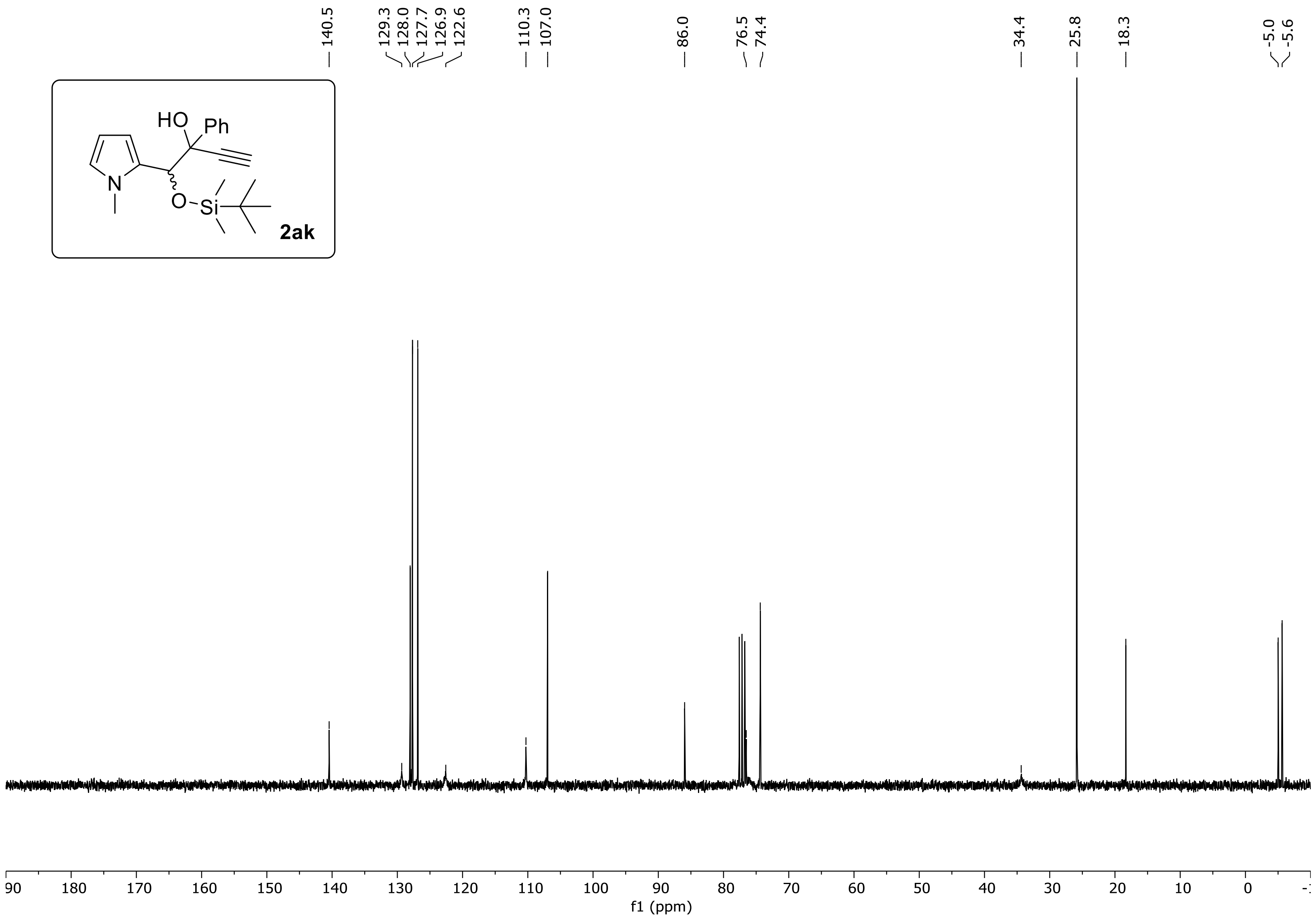

Figure S85: ^{1}H NMR of compound **2ba** in $CDCl_3$ at 300 MHz.

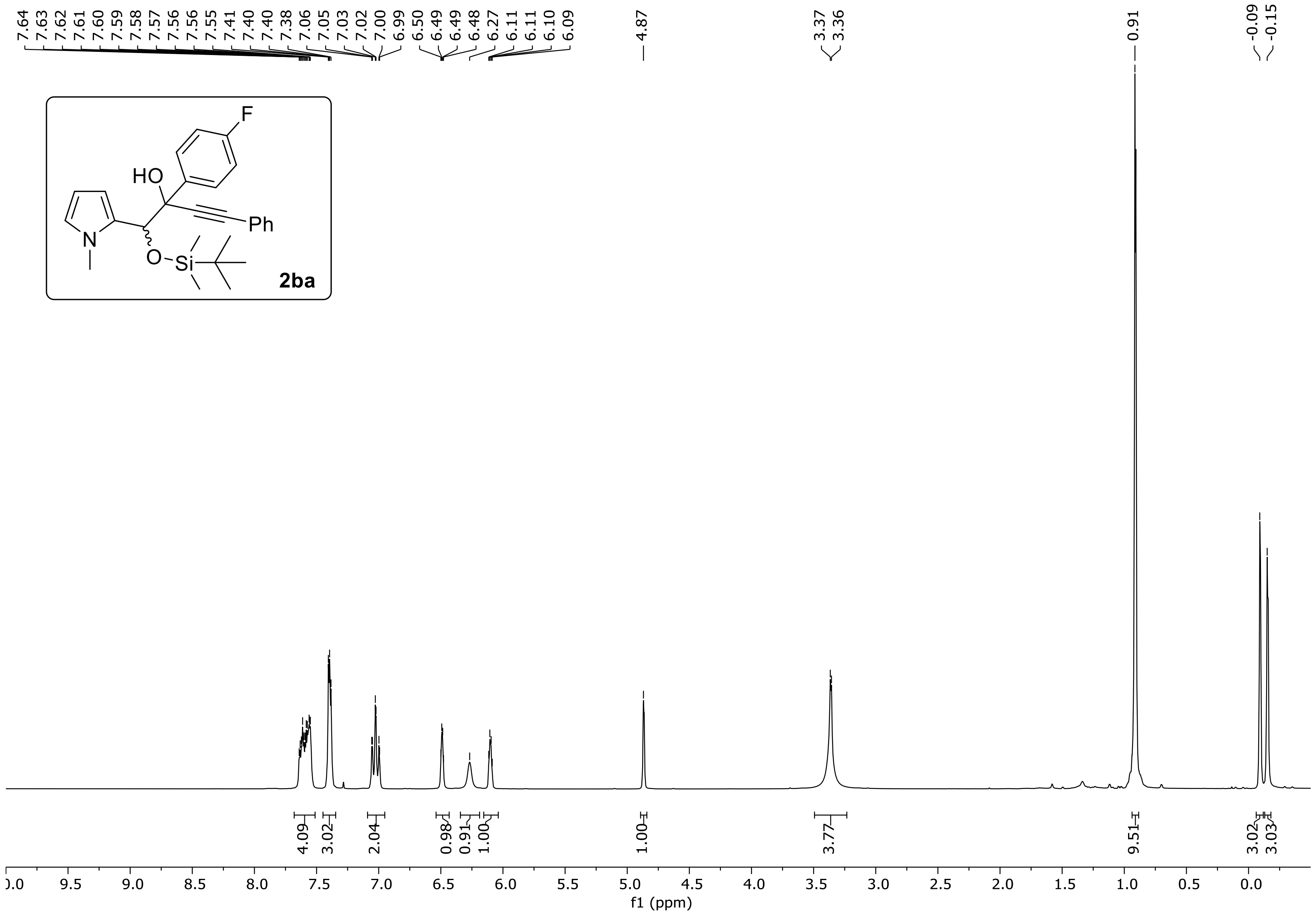

Figure S86: ^{13}C NMR of compound **2ba** in $CDCl_3$ at 75.4 MHz.

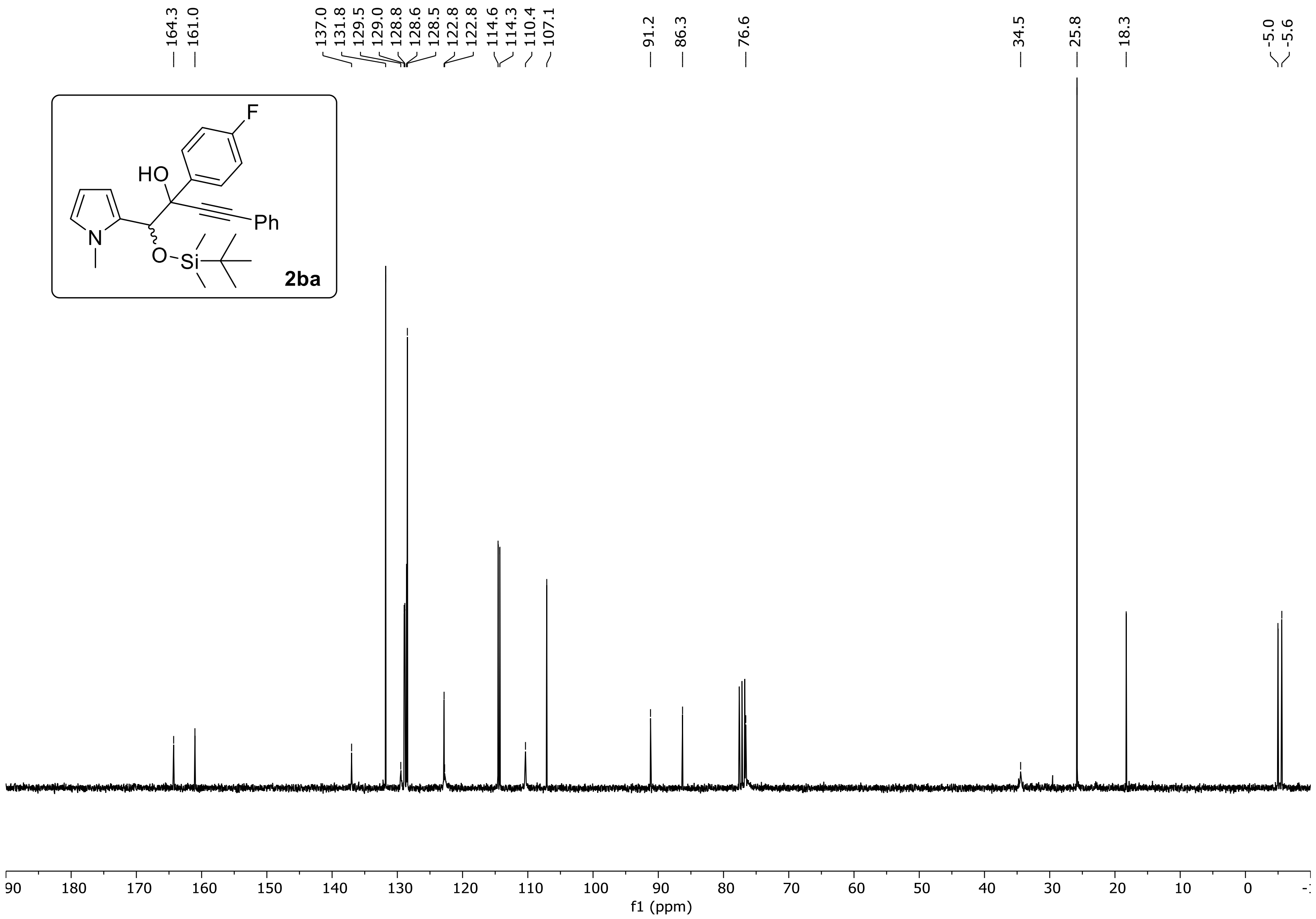

Figure S87: ^{1}H NMR of compound **2bf** in $CDCl_3$ at 300 MHz.

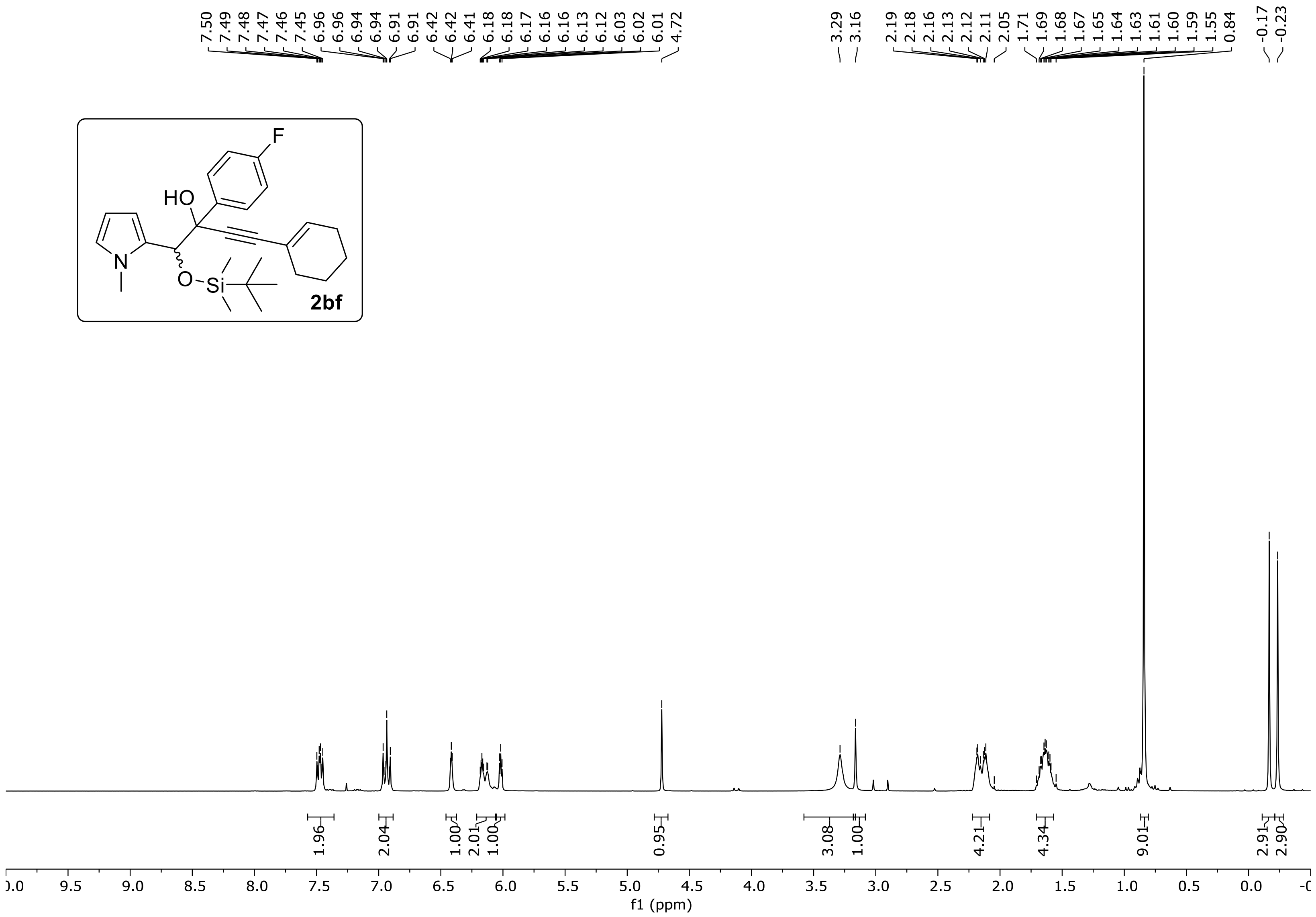

Figure S88: ^{13}C NMR of compound **2bf** in $CDCl_3$ at 75.4 MHz.

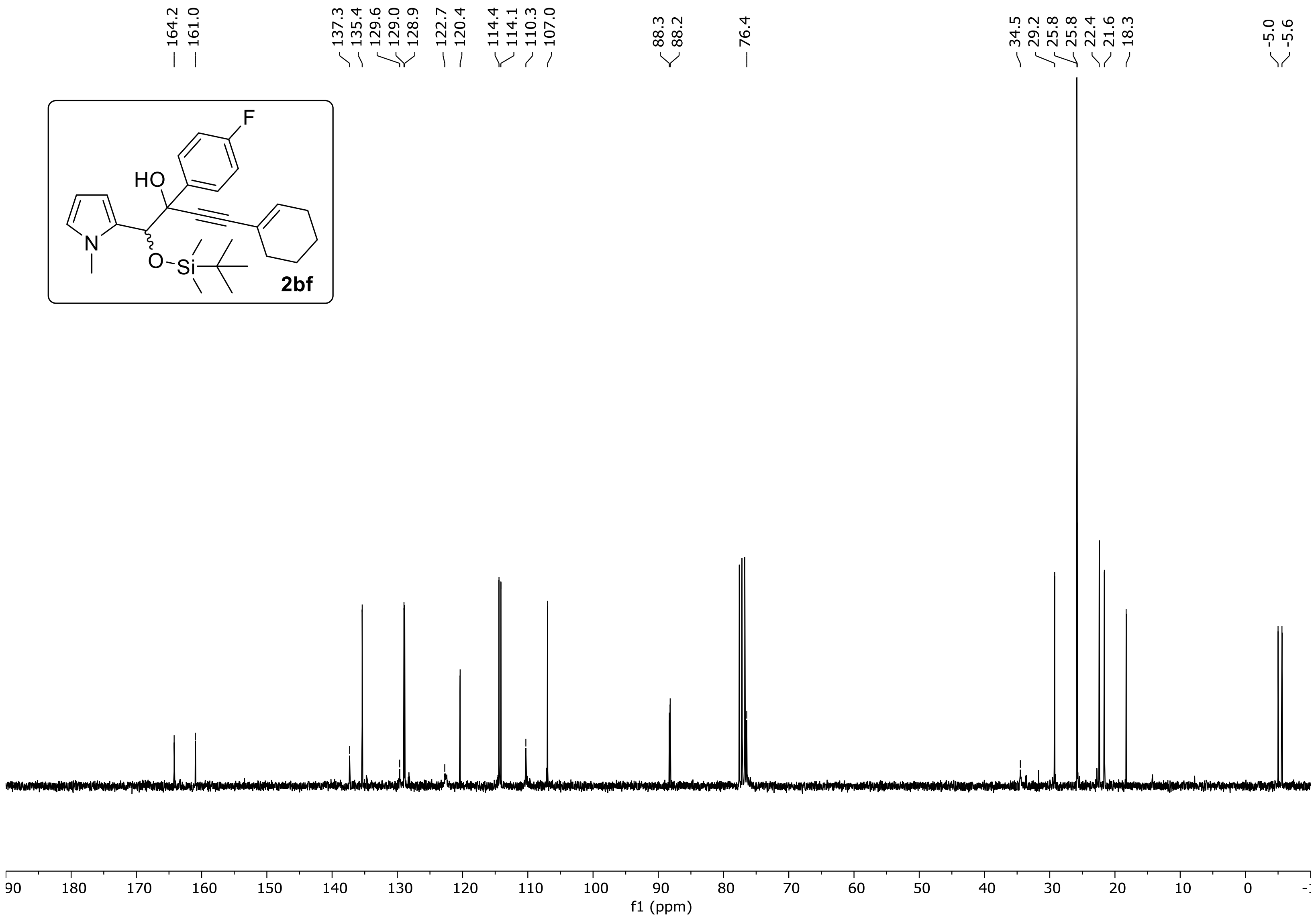

Figure S89: ^{1}H NMR of compound **2ca** in $CDCl_3$ at 300 MHz.

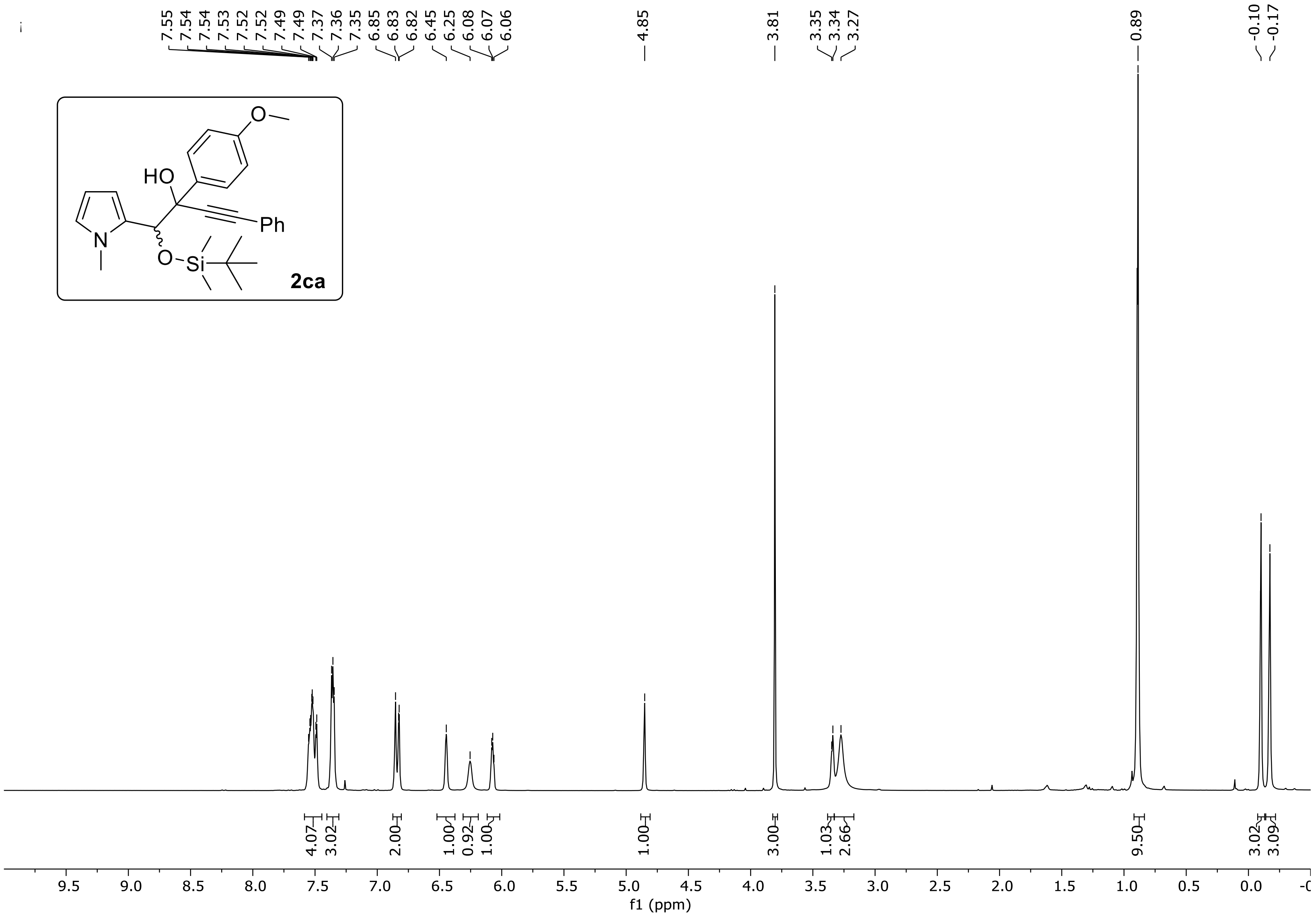

Figure S90: ^{13}C NMR of compound **2ca** in $CDCl_3$ at 75.4 MHz.

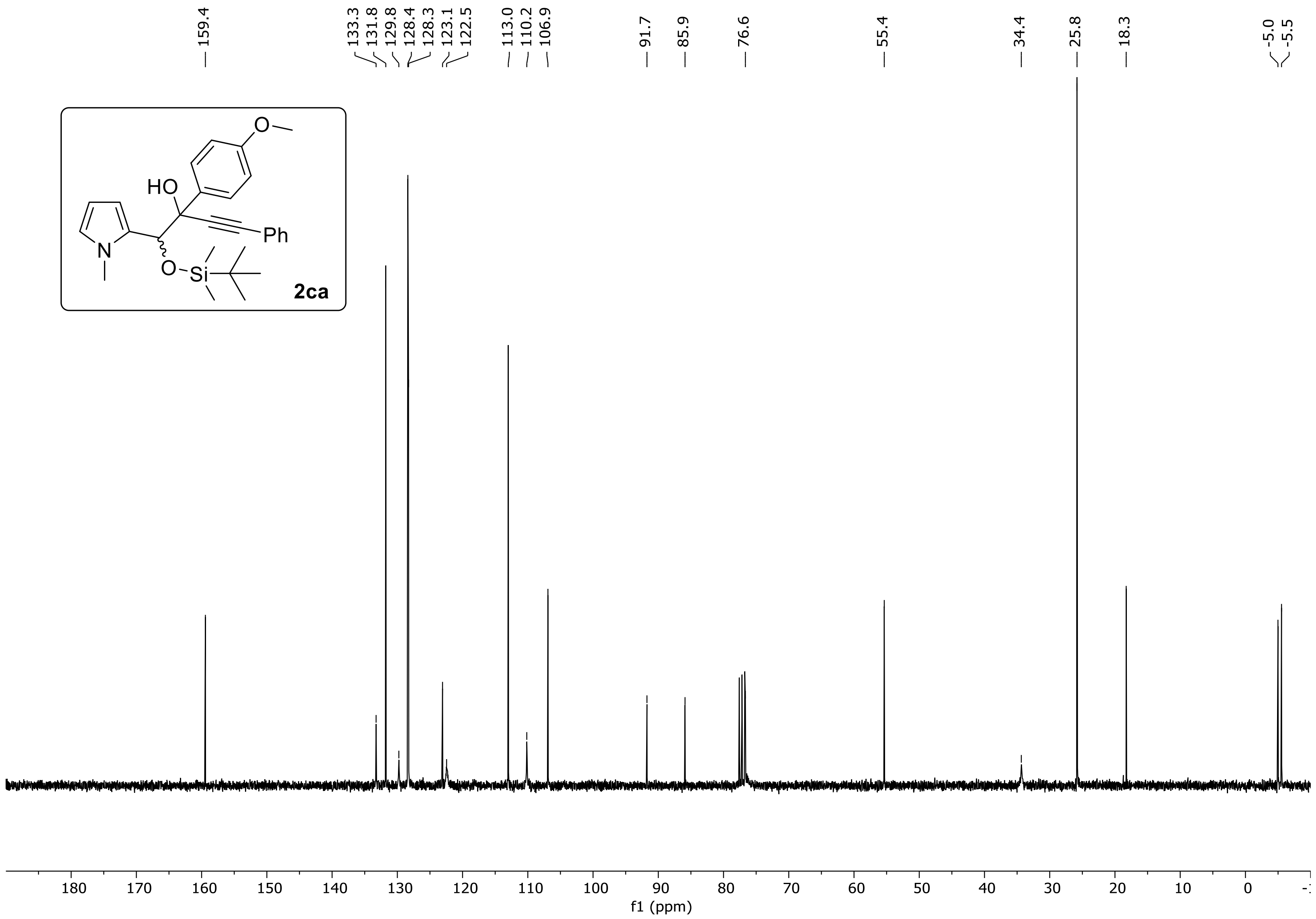

Figure S91: ^{1}H NMR of compound **2ea** in $CDCl_3$ at 300 MHz.

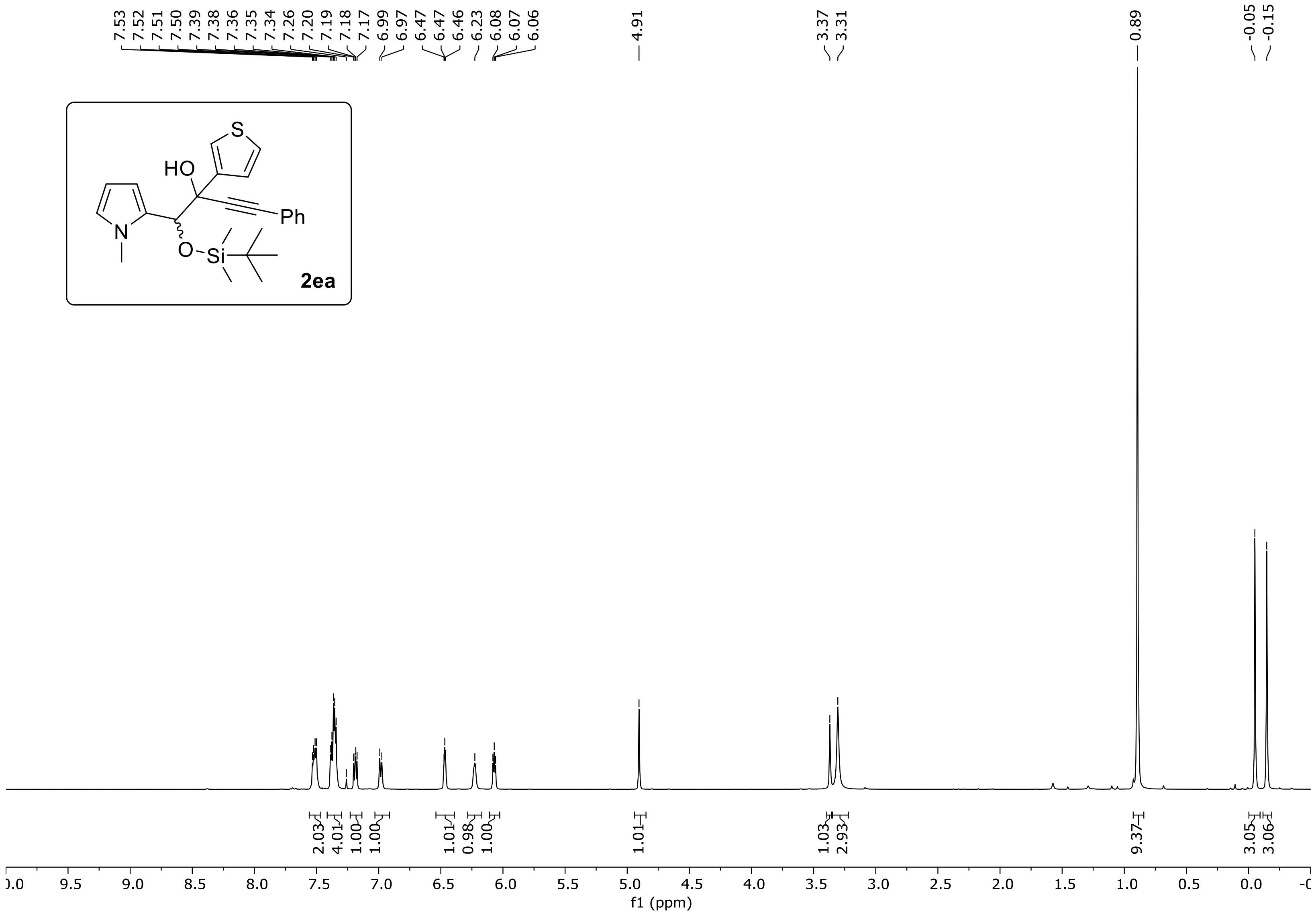

Figure S92: ^{13}C NMR of compound **2ea** in $CDCl_3$ at 75.4 MHz.

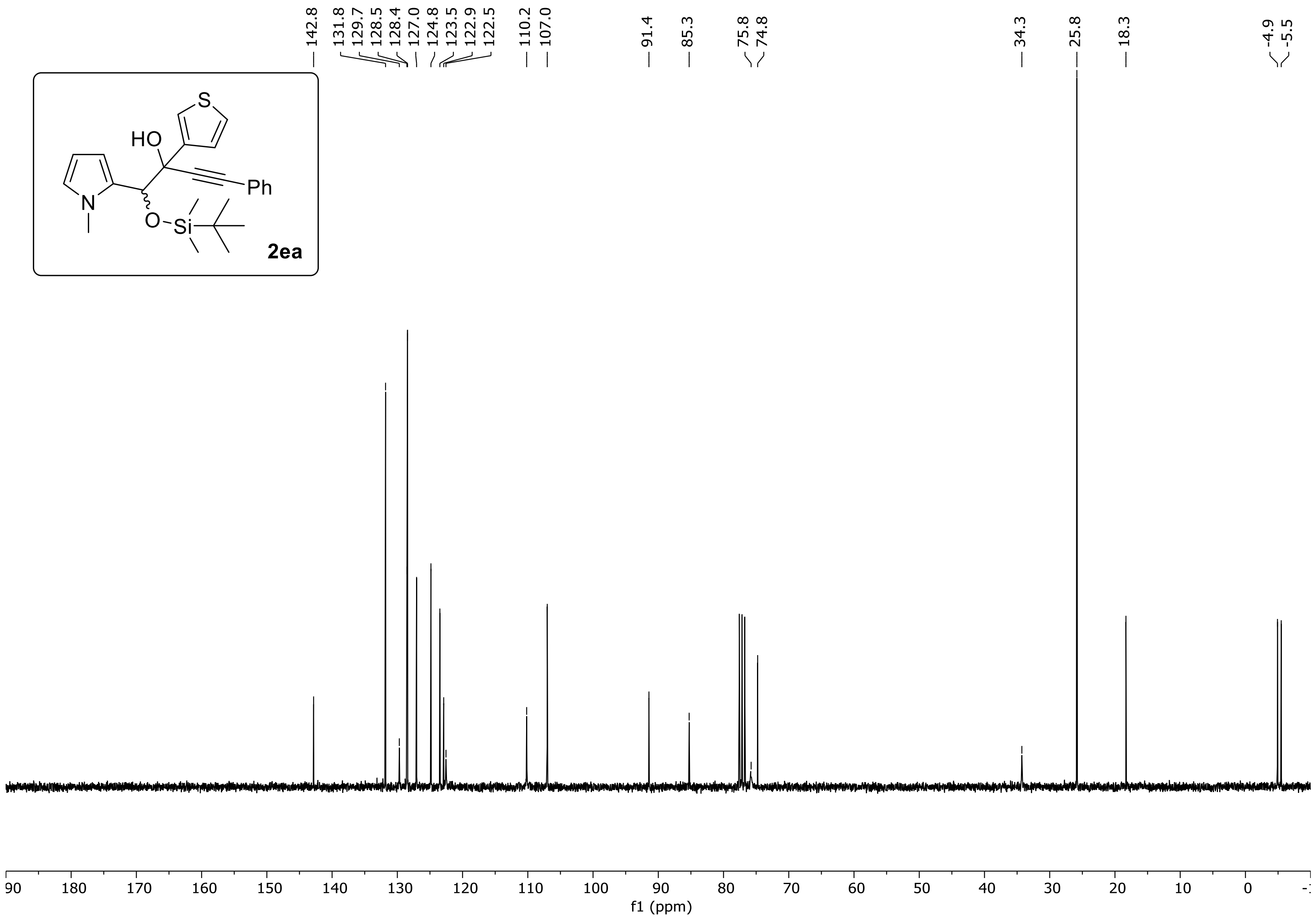

Figure S93: ^{1}H NMR of compound **2fa** in $CDCl_3$ at 300 MHz.

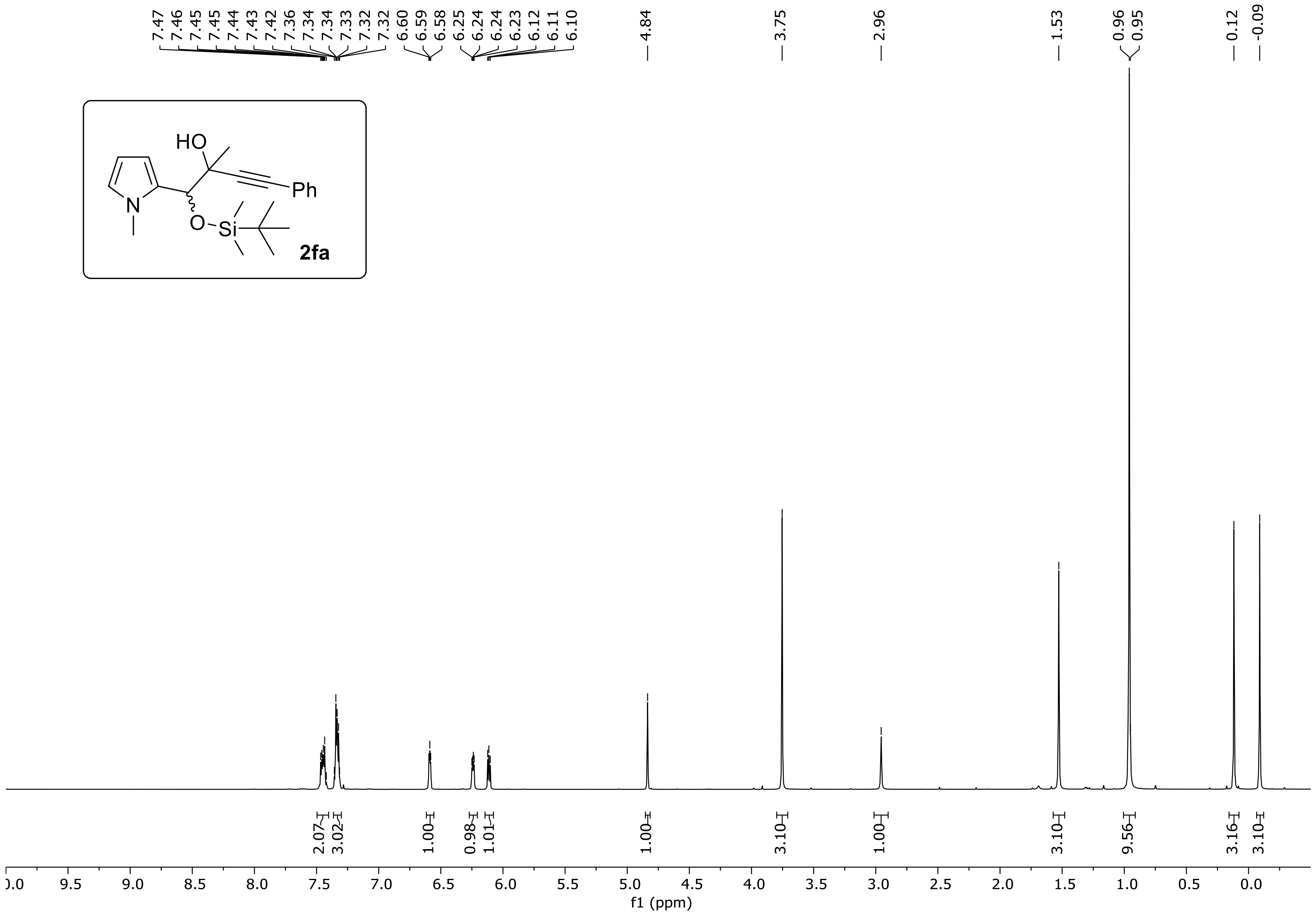

Figure S94: ^{13}C NMR of compound **2fa** in $CDCl_3$ at 75.4 MHz.

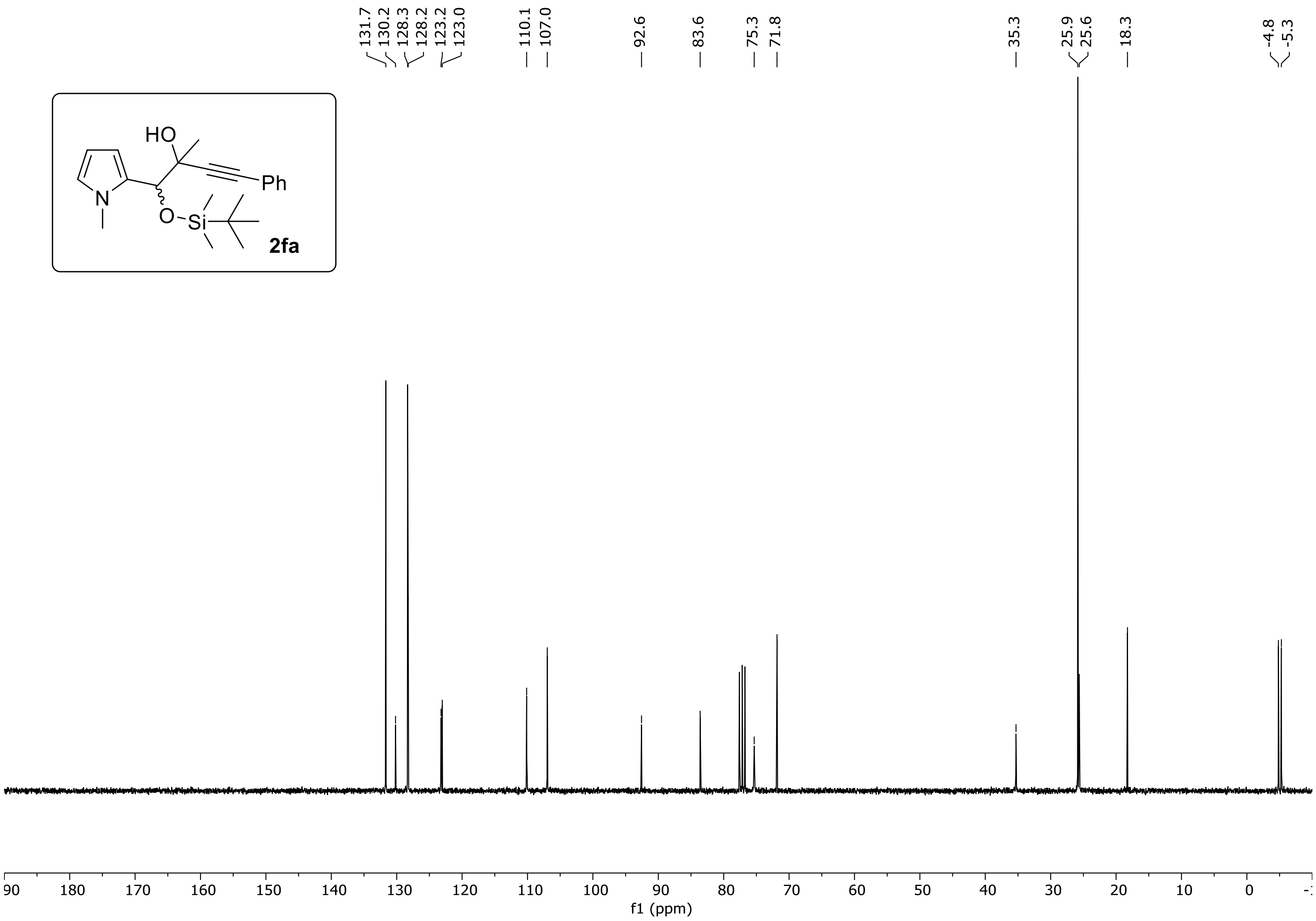

Figure S95: ^{1}H NMR of compound **2fb** in $CDCl_3$ at 300 MHz.

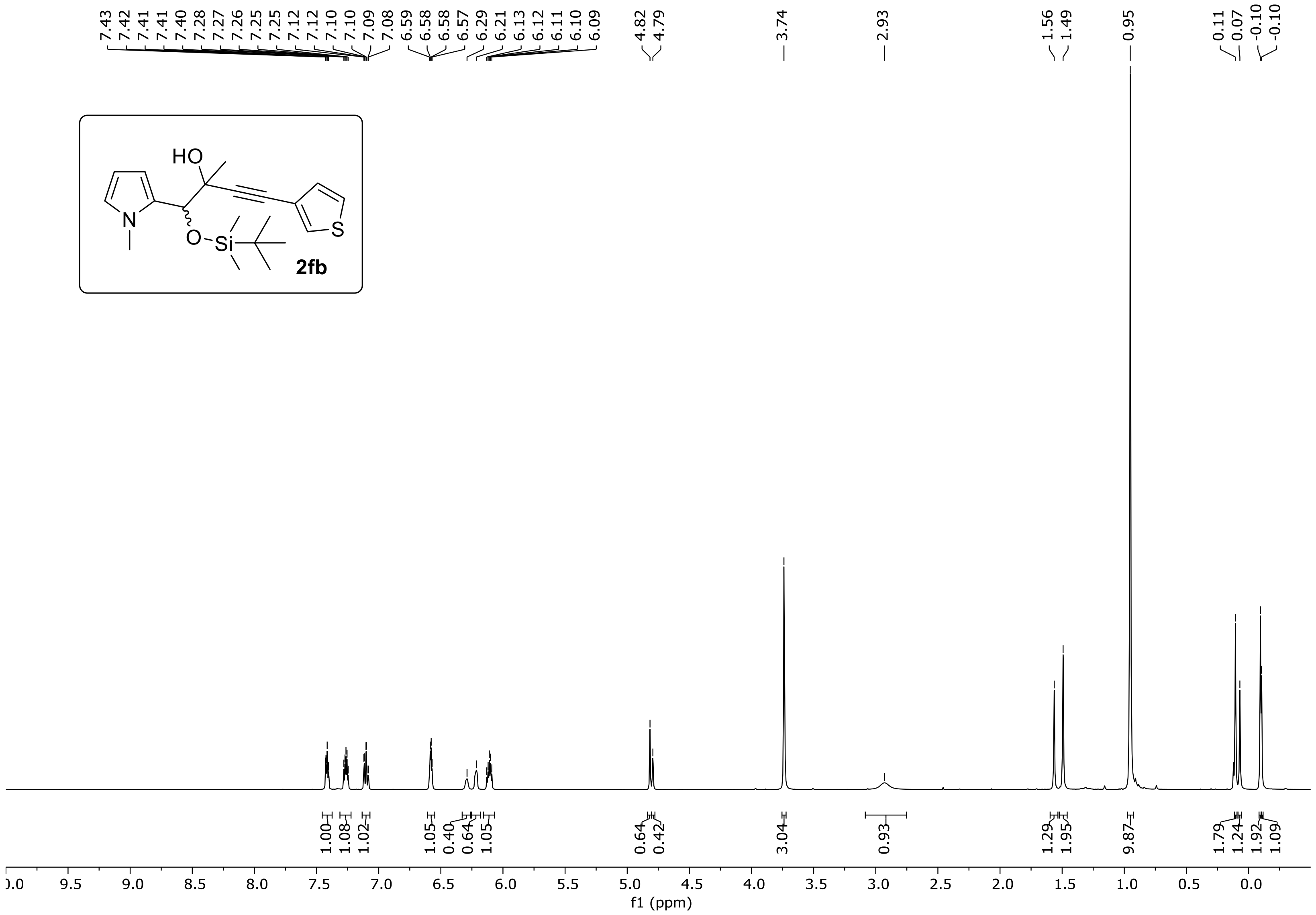

Figure S96: ^{13}C NMR of compound **2fb** in $CDCl_3$ at 75.4 MHz.

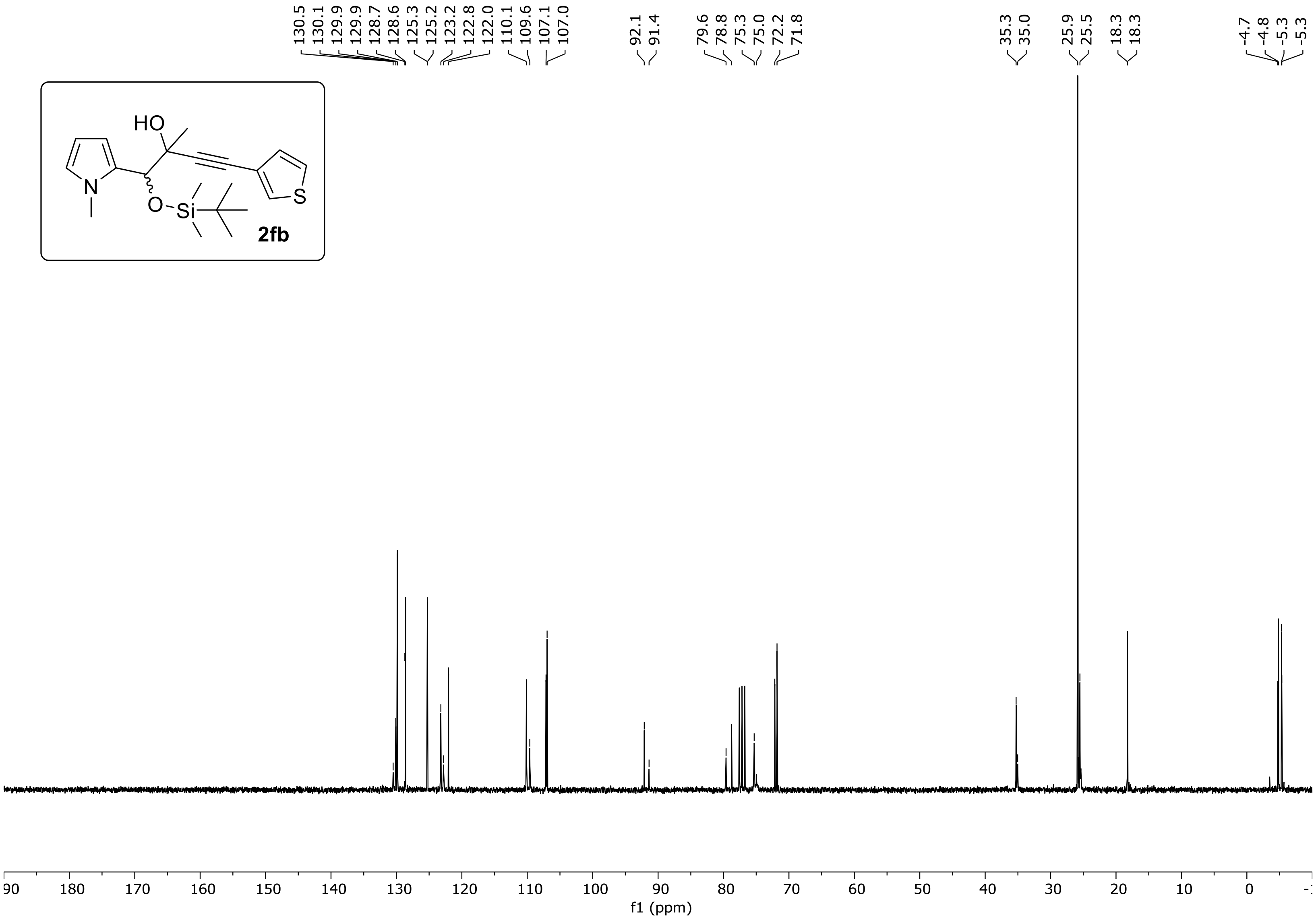

Figure S97: [1] H NMR of compound **2ff** in $CDCl_3$ at 300 MHz.

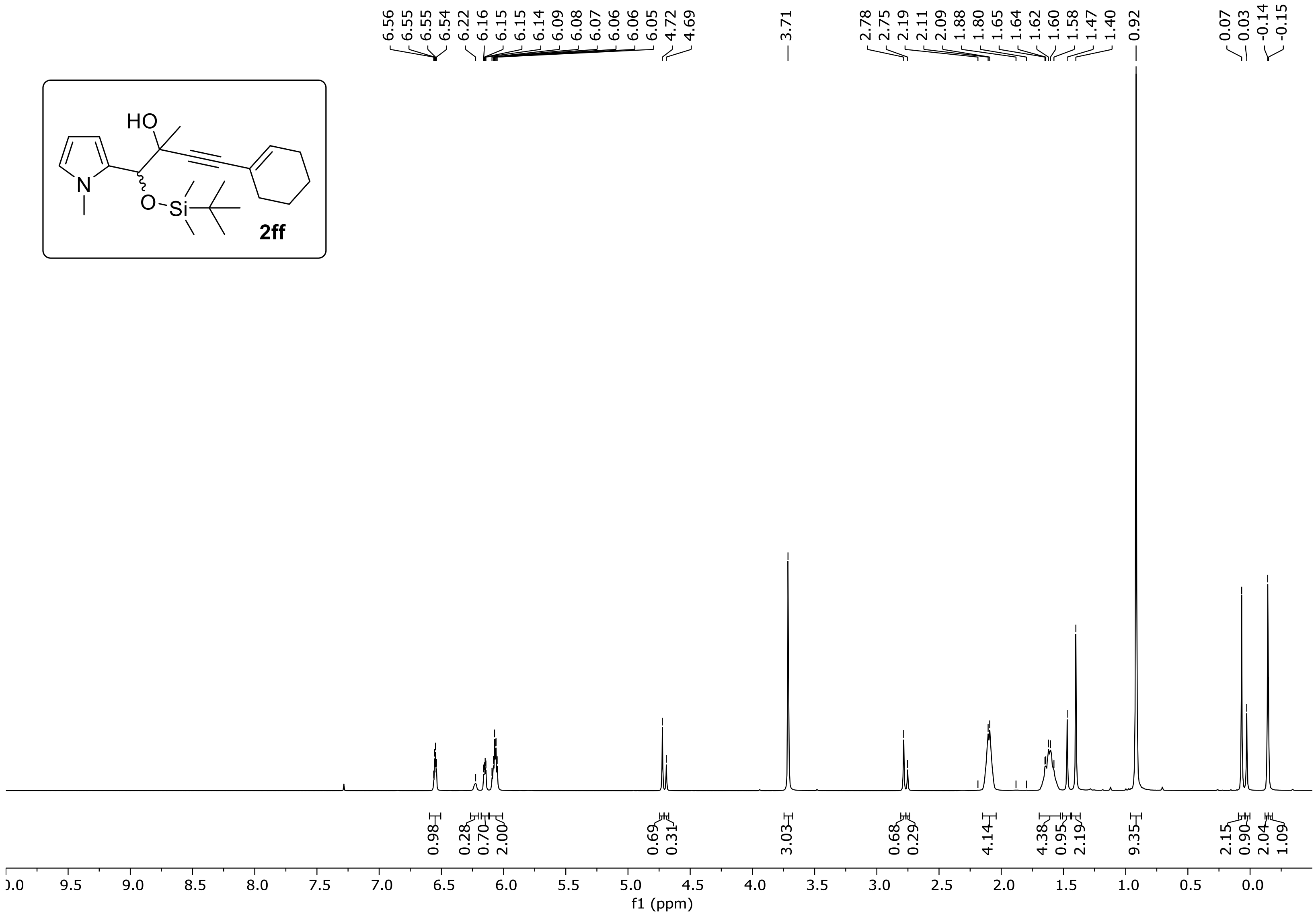

Figure S98: ^{13}C NMR of compound **2ff** in $CDCl_3$ at 75.4 MHz.

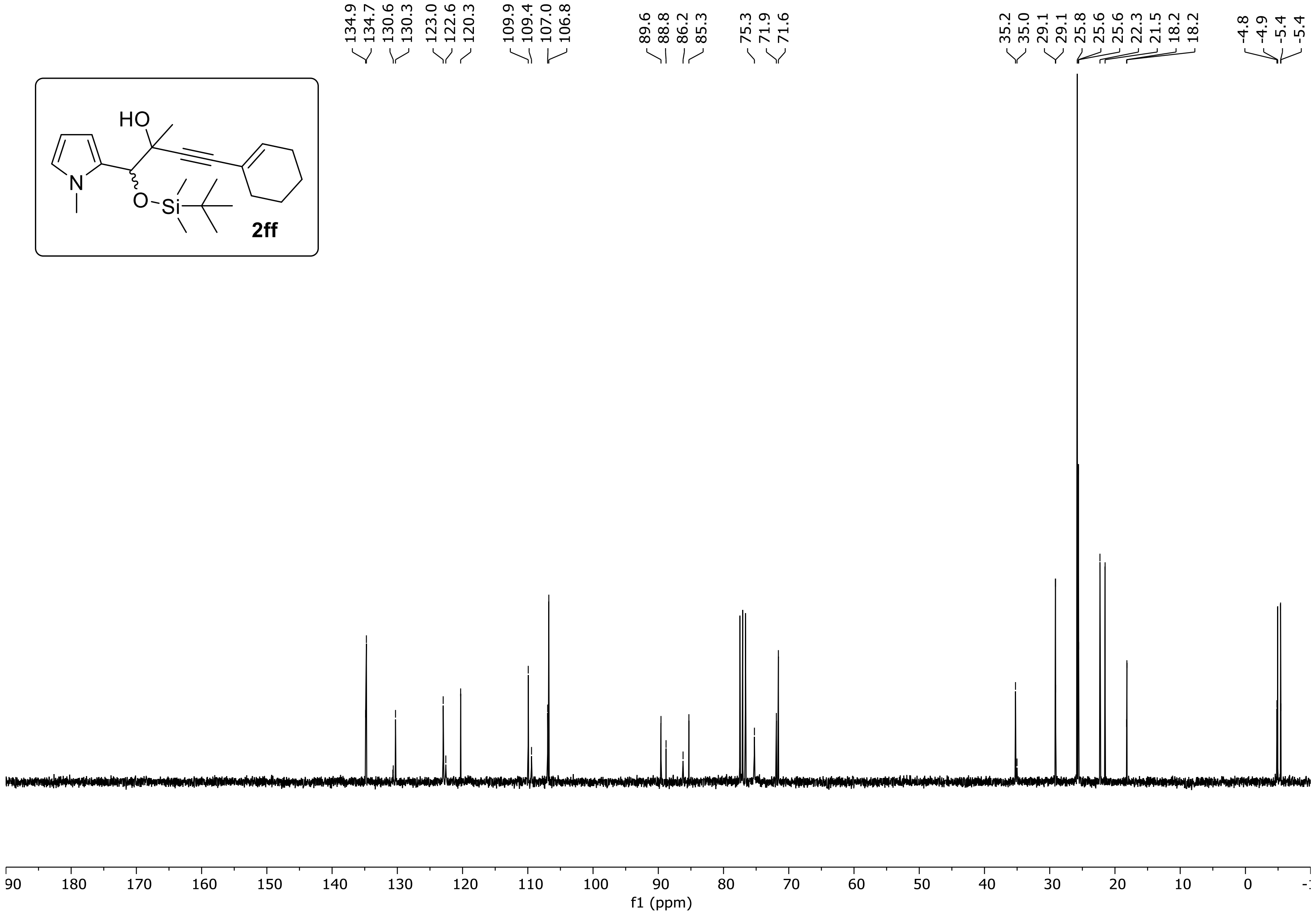

Figure S99: ^{1}H NMR of compound **2ga** in $CDCl_3$ at 300 MHz.

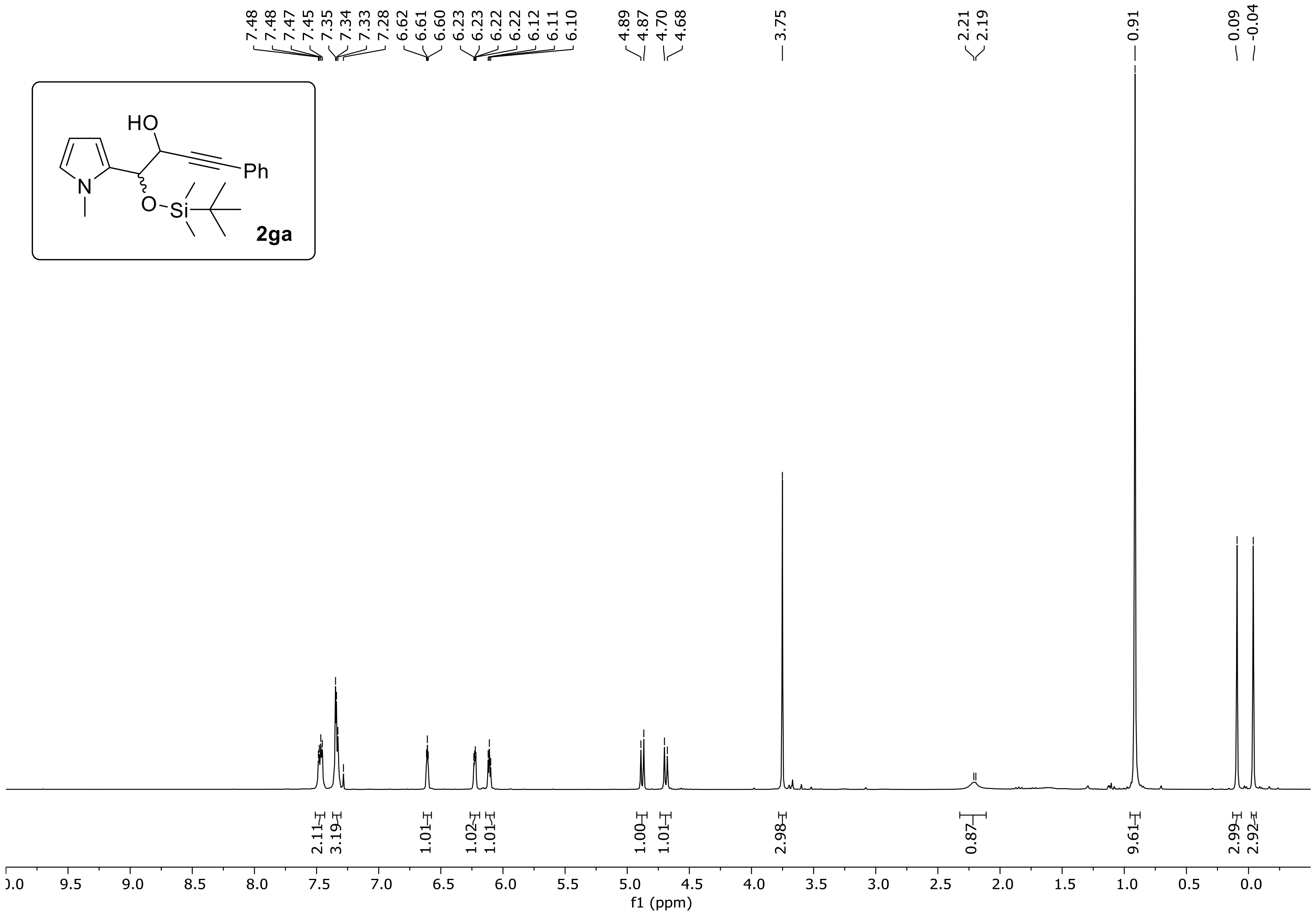

Figure S100: ^{13}C NMR of compound **2ga** in $CDCl_3$ at 75.4 MHz.

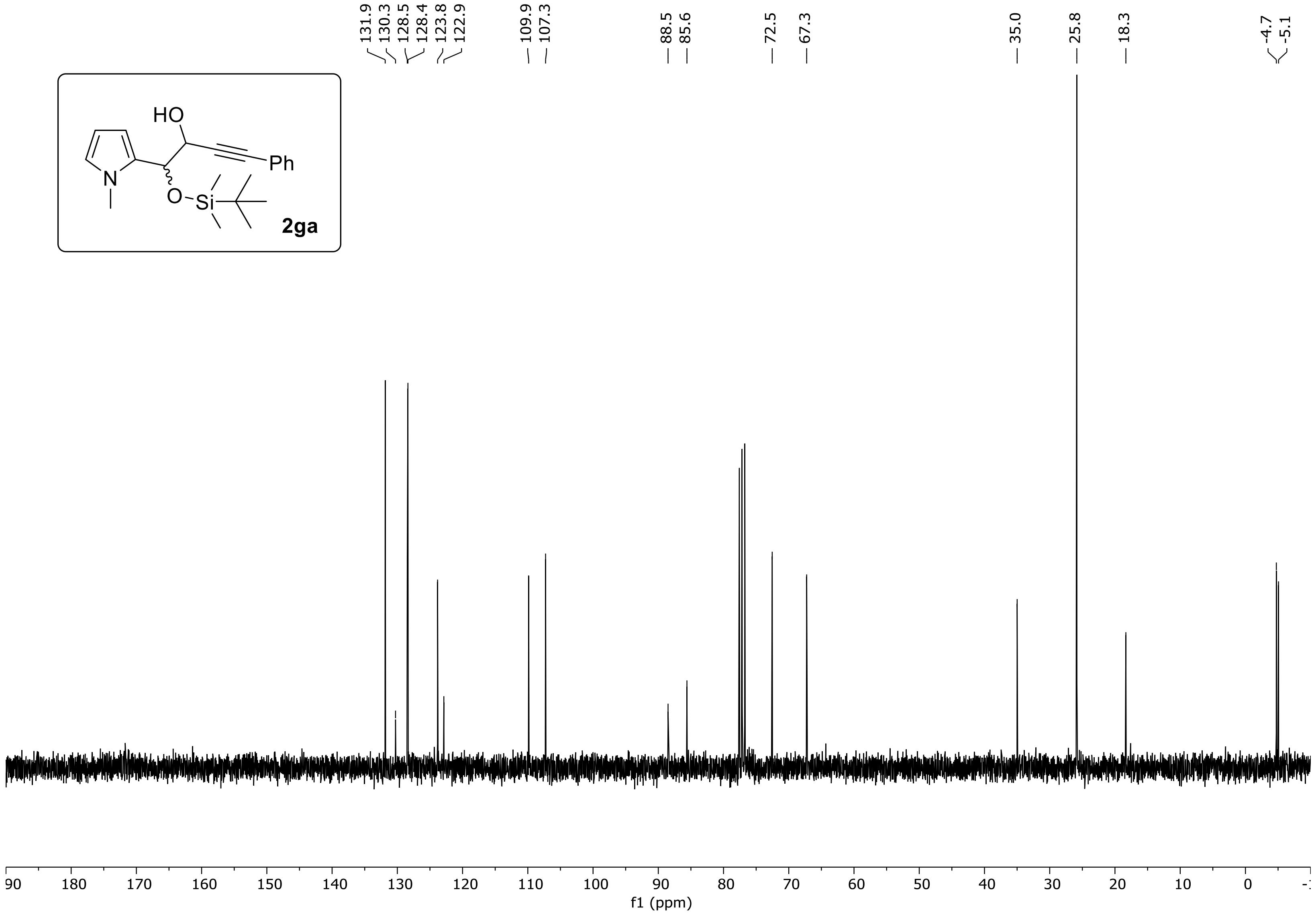

Figure S101: ^{1}H NMR of compound **2gb** in $CDCl_3$ at 300 MHz.

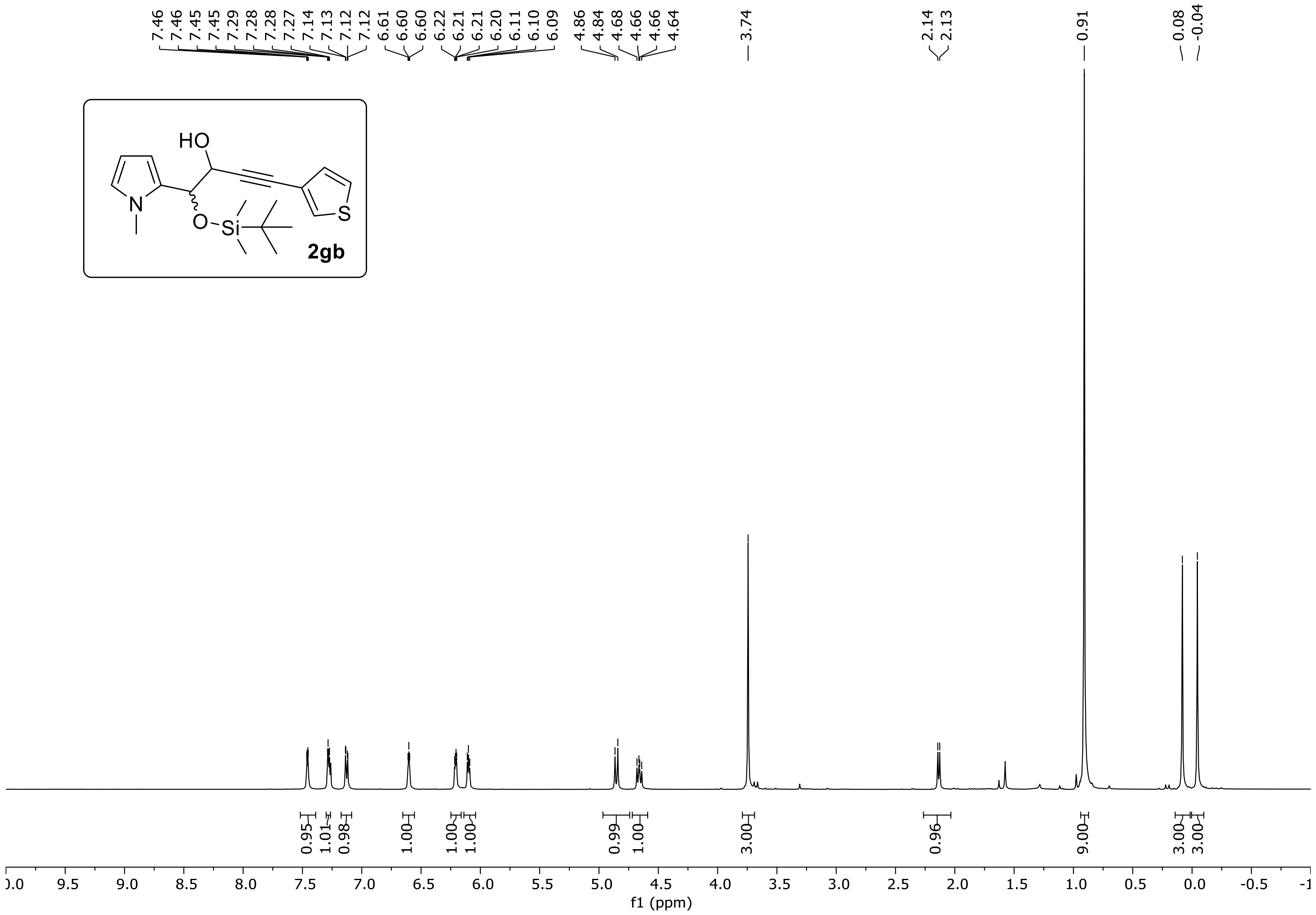

Figure S102: ^{13}C NMR of compound **2gb** in $CDCl_3$ at 75.4 MHz.

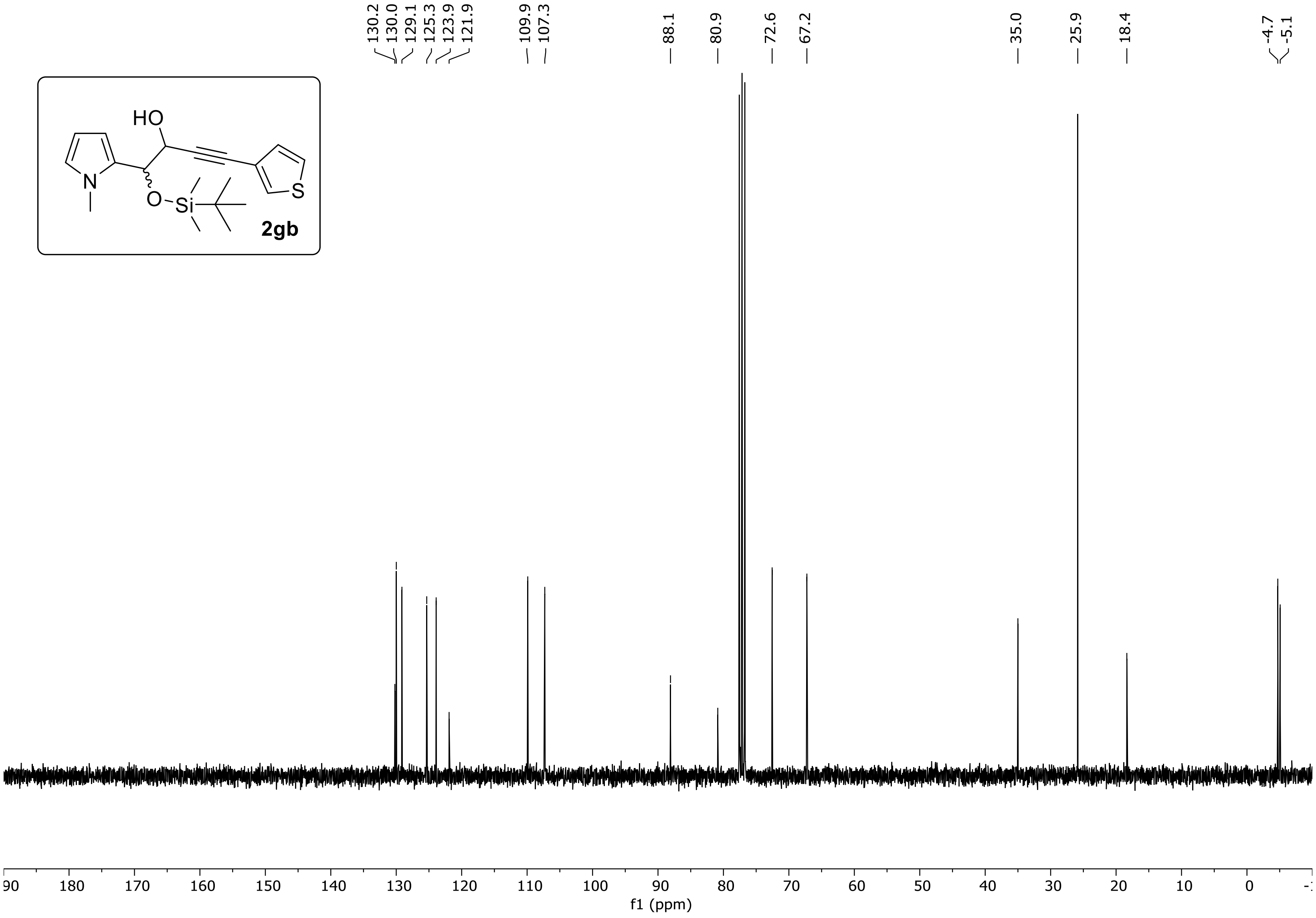

Figure S103: ^{1}H NMR of compound **2ha** in $CDCl_3$ at 300 MHz.

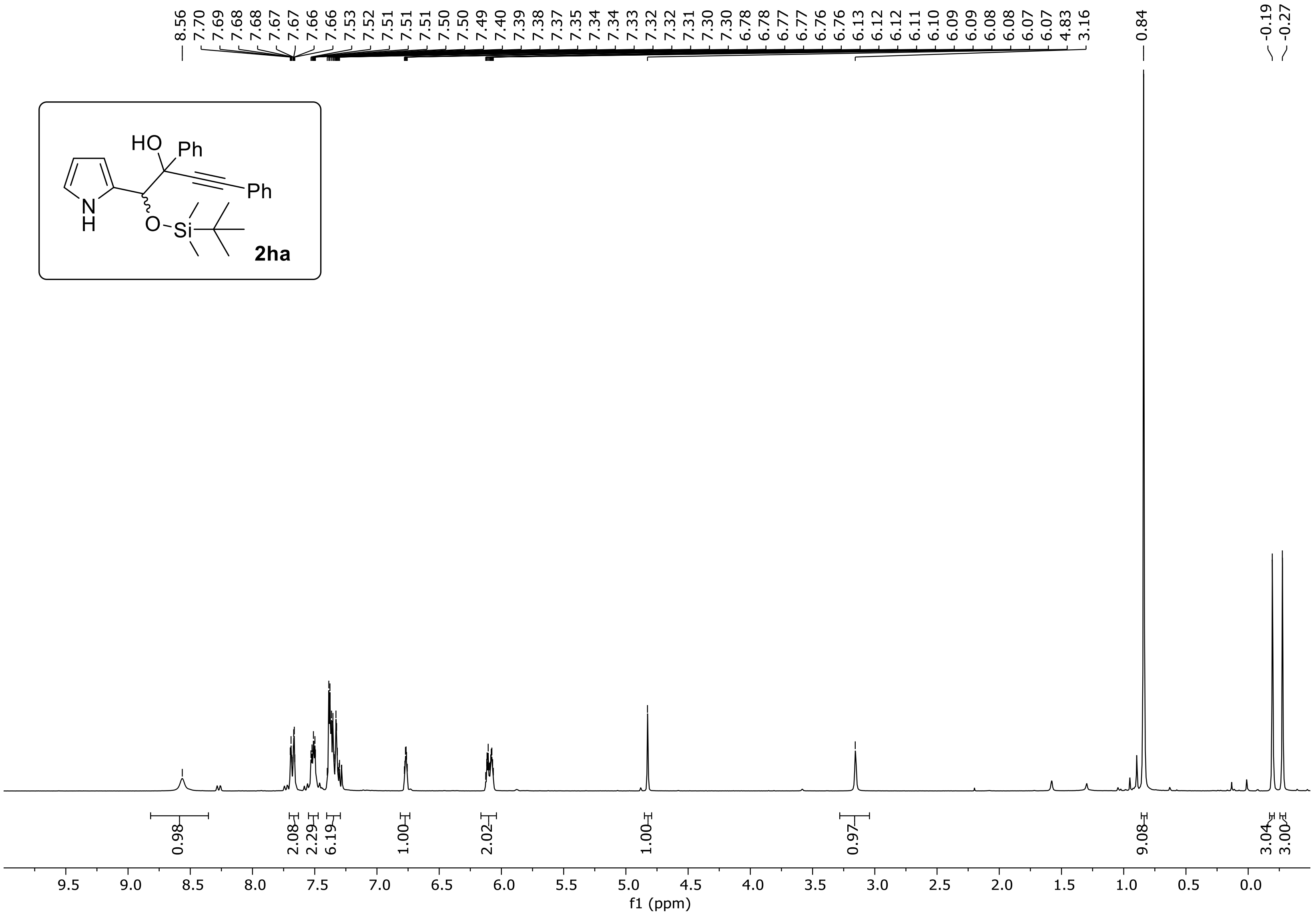

Figure S104: ^{13}C NMR of compound **2ha** in $CDCl_3$ at 75.4 MHz.

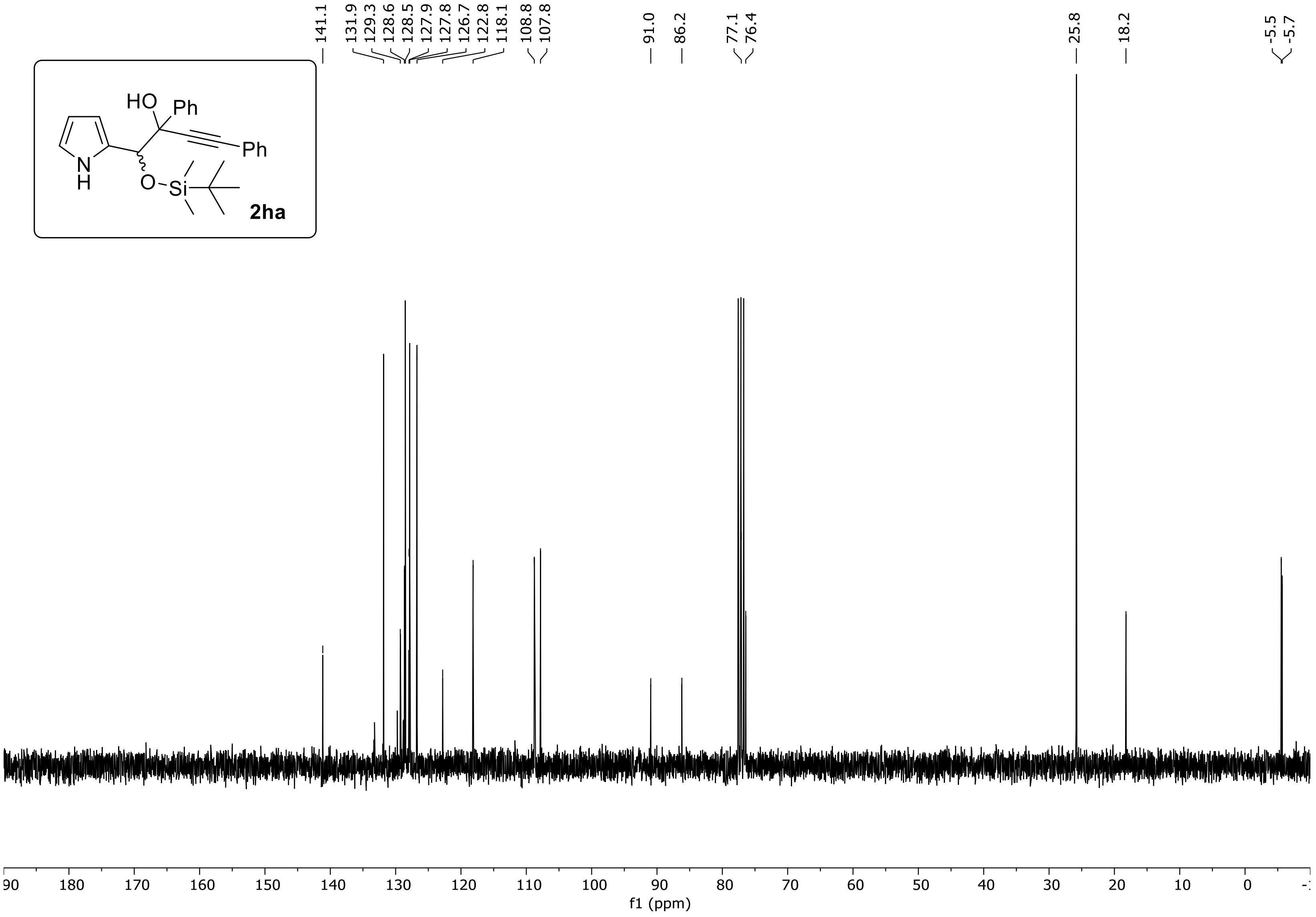

Figure S105: ^{1}H NMR of compound **2hb** in $CDCl_3$ at 300 MHz.

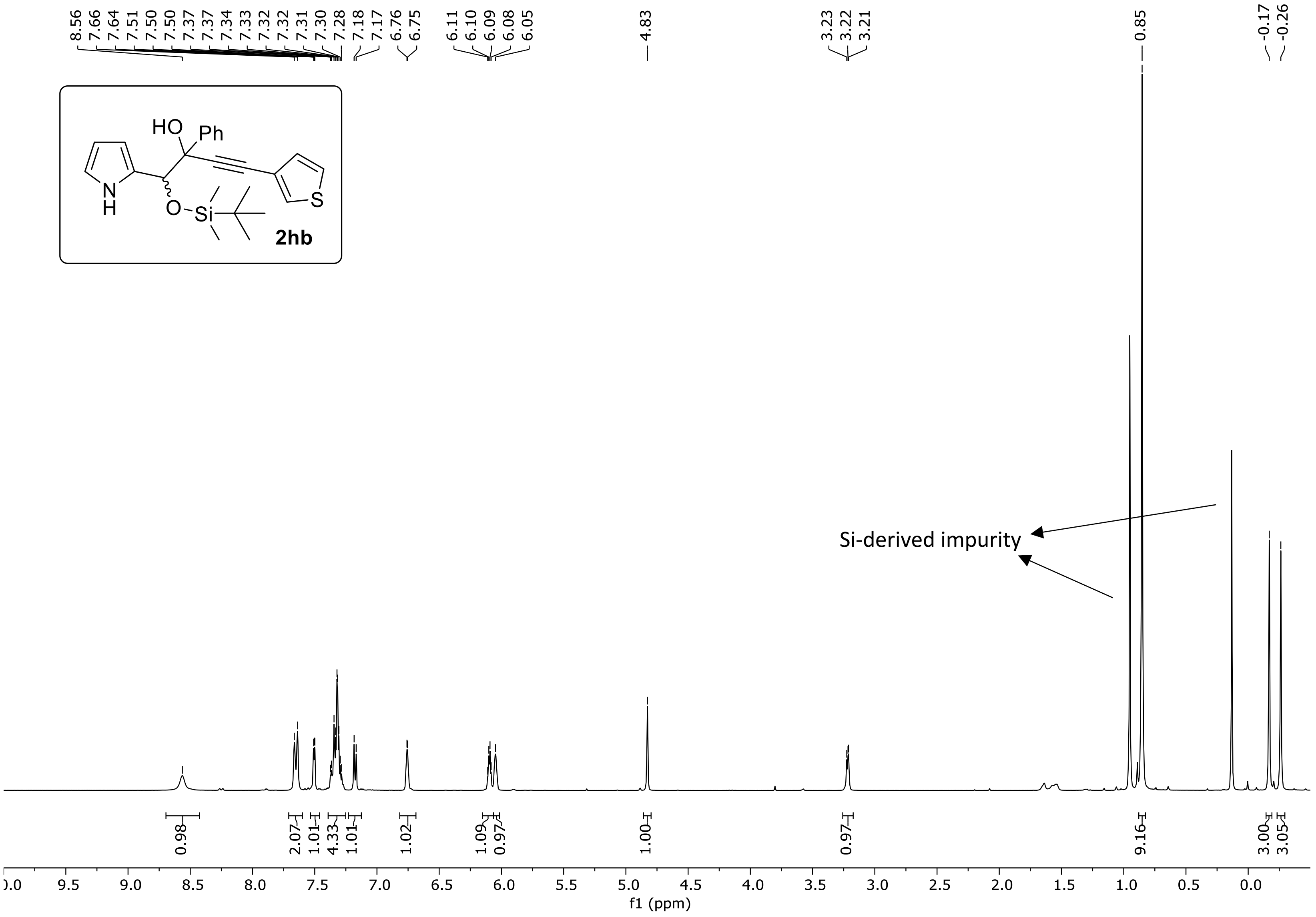

Figure S106: ^{13}C NMR of compound **2hb** in $CDCl_3$ at 75.4 MHz.

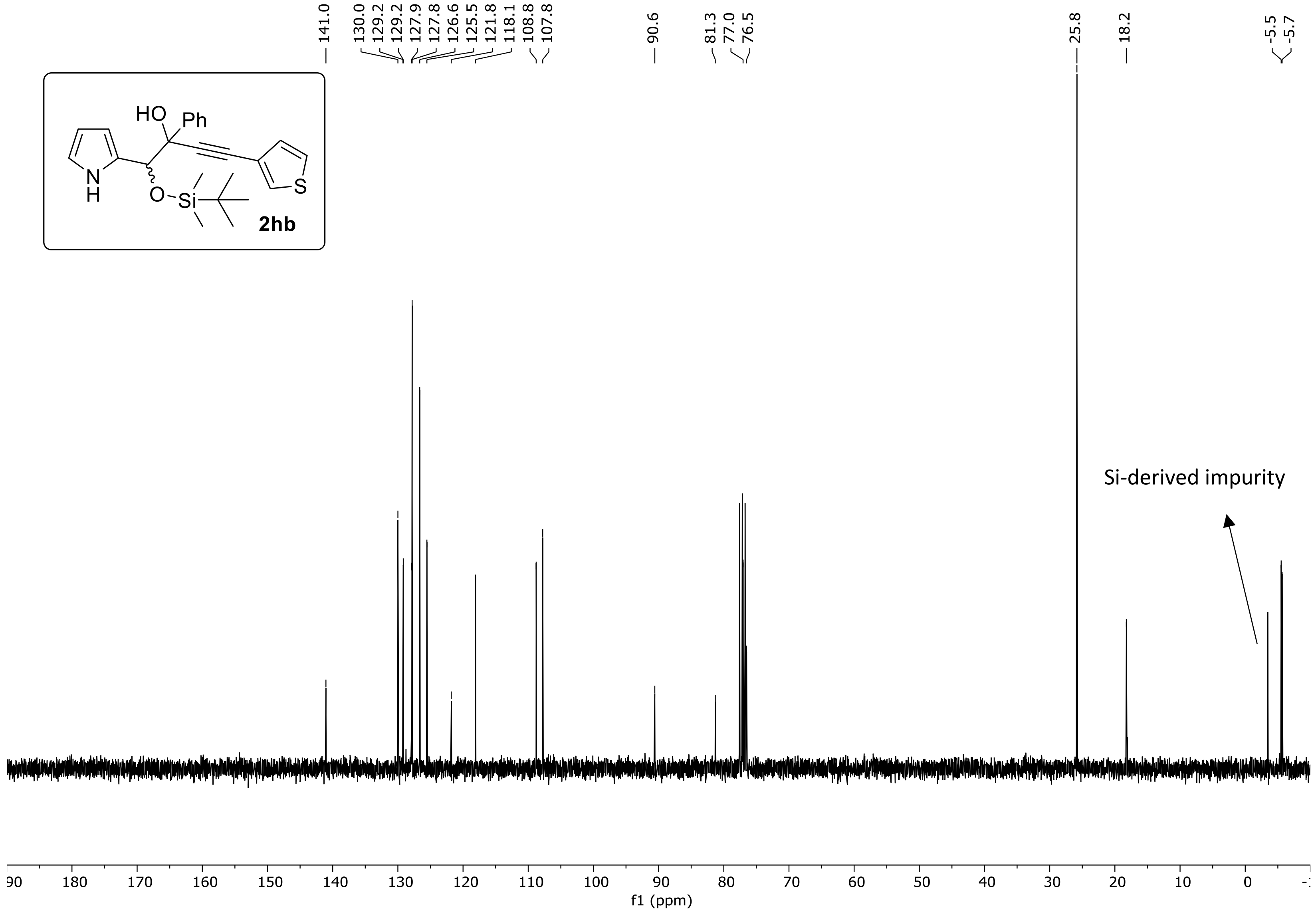

Figure S107: ^{1}H NMR of compound **2hh** in $CDCl_3$ at 300 MHz.

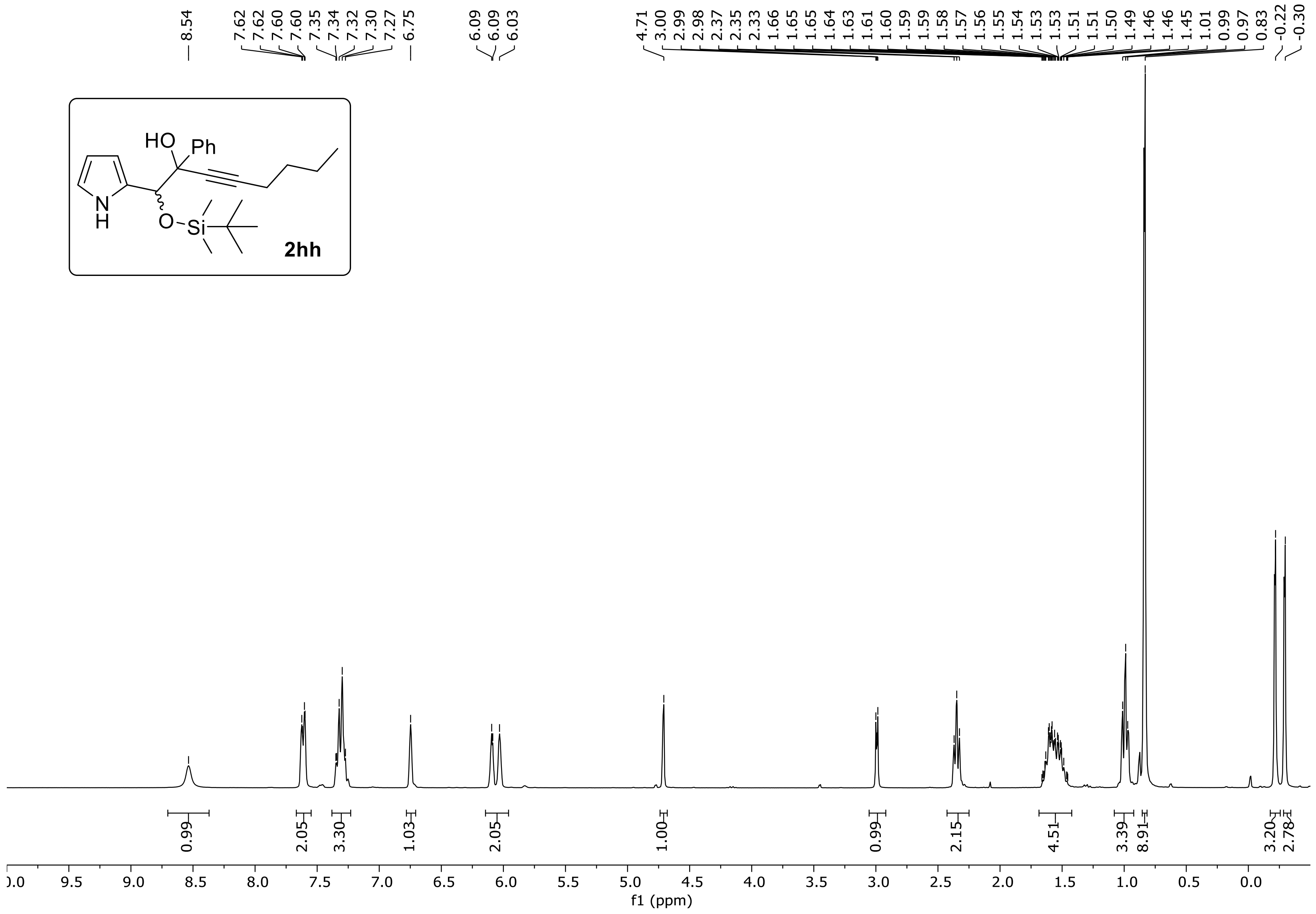

Figure S108: ^{13}C NMR of compound **2hh** in $CDCl_3$ at 75.4 MHz.

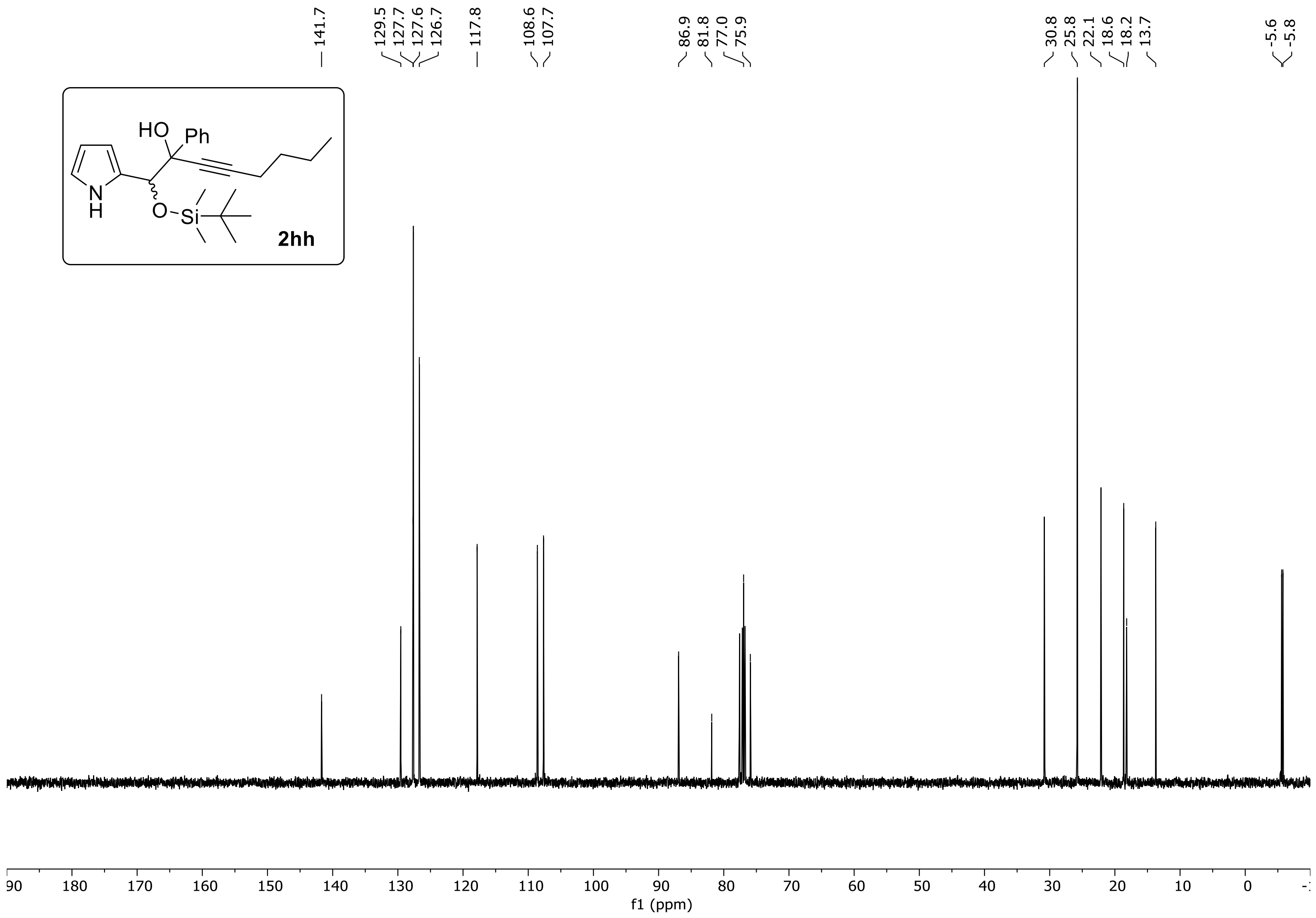

Figure S109: ^{1}H NMR of compound **2ia** in $CDCl_3$ at 300 MHz.

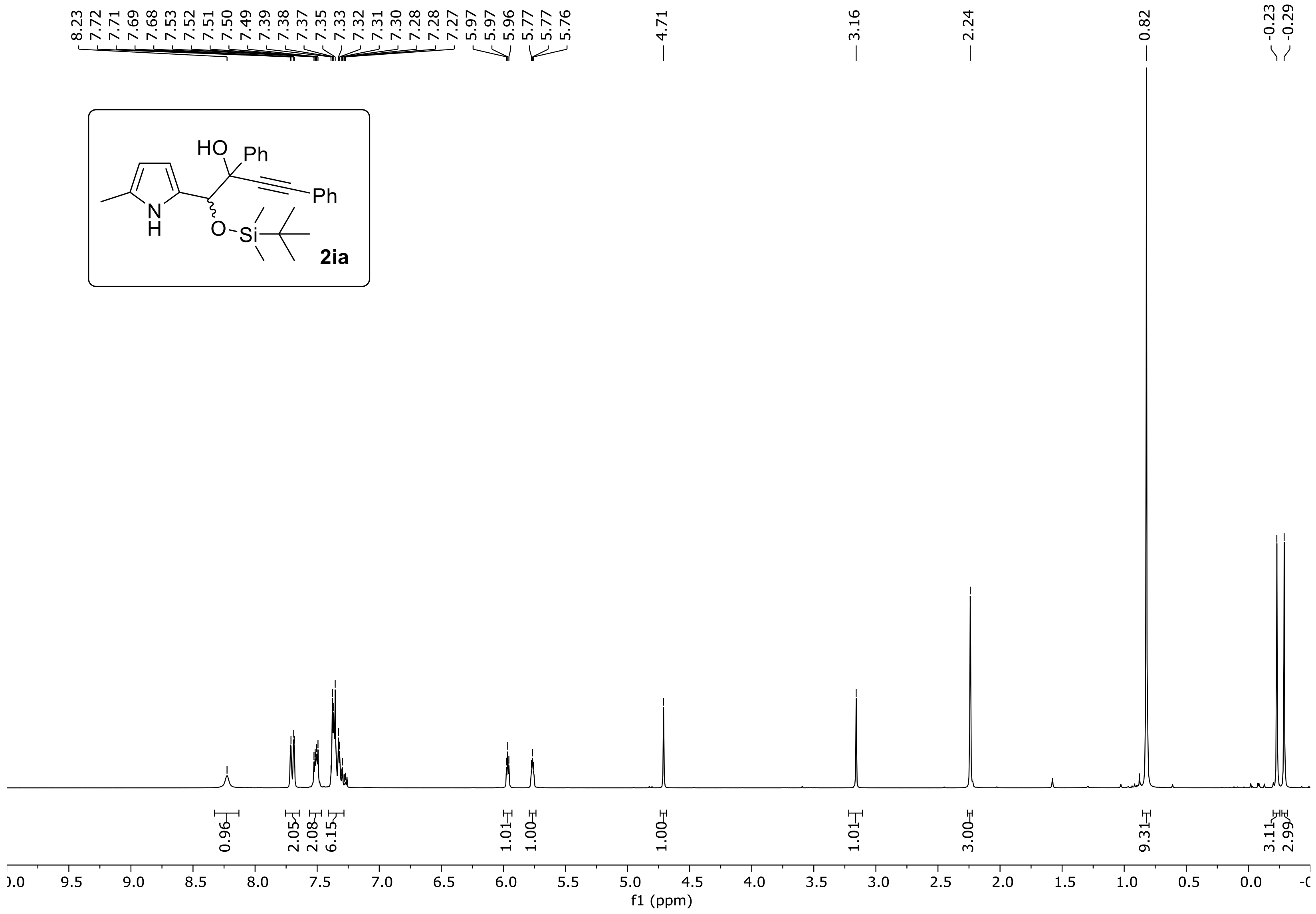

Figure S110: ^{13}C NMR of compound **2ia** in $CDCl_3$ at 75.4 MHz.

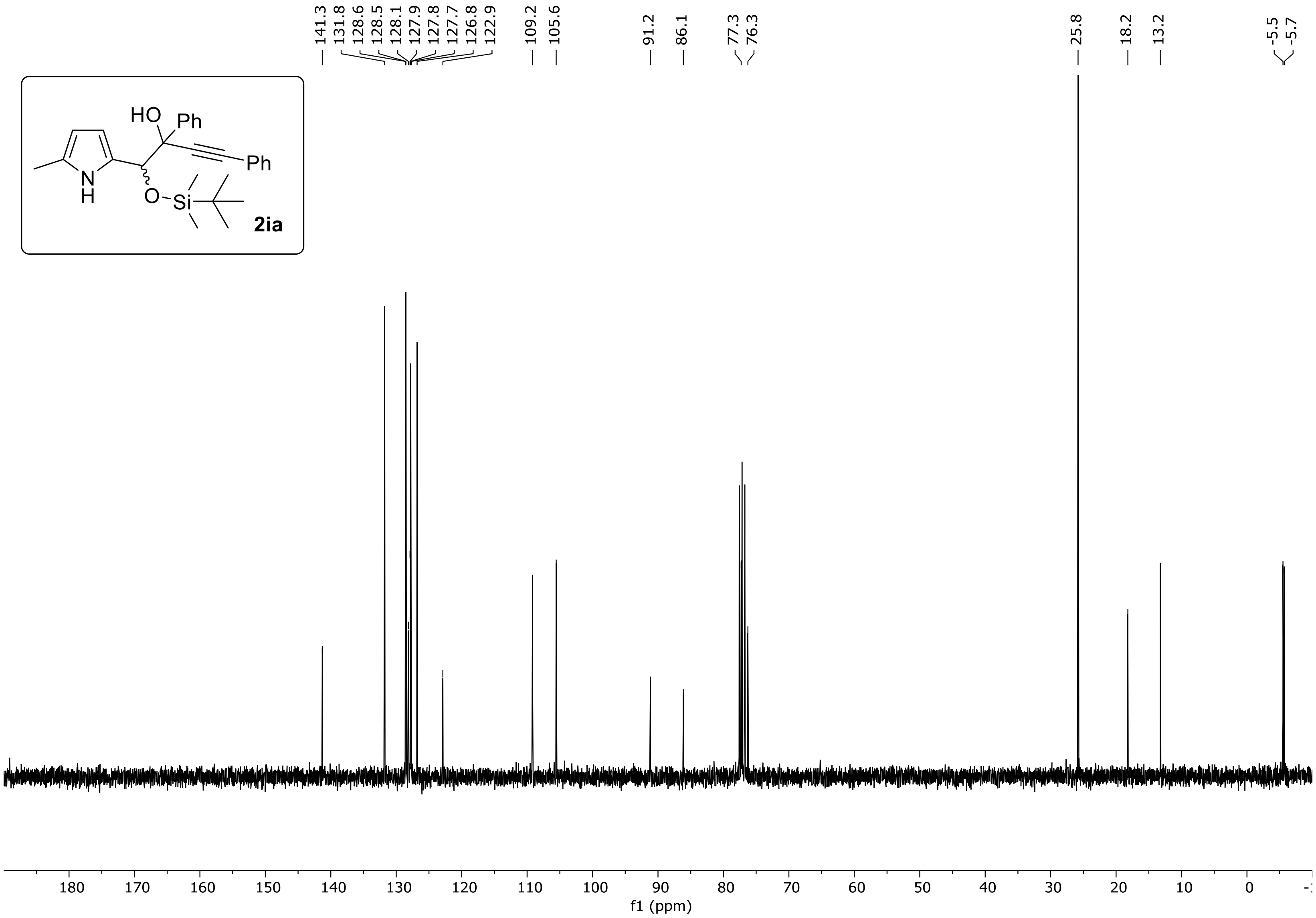

Figure S111: ^{1}H NMR of compound **2ib** in $CDCl_3$ at 300 MHz.

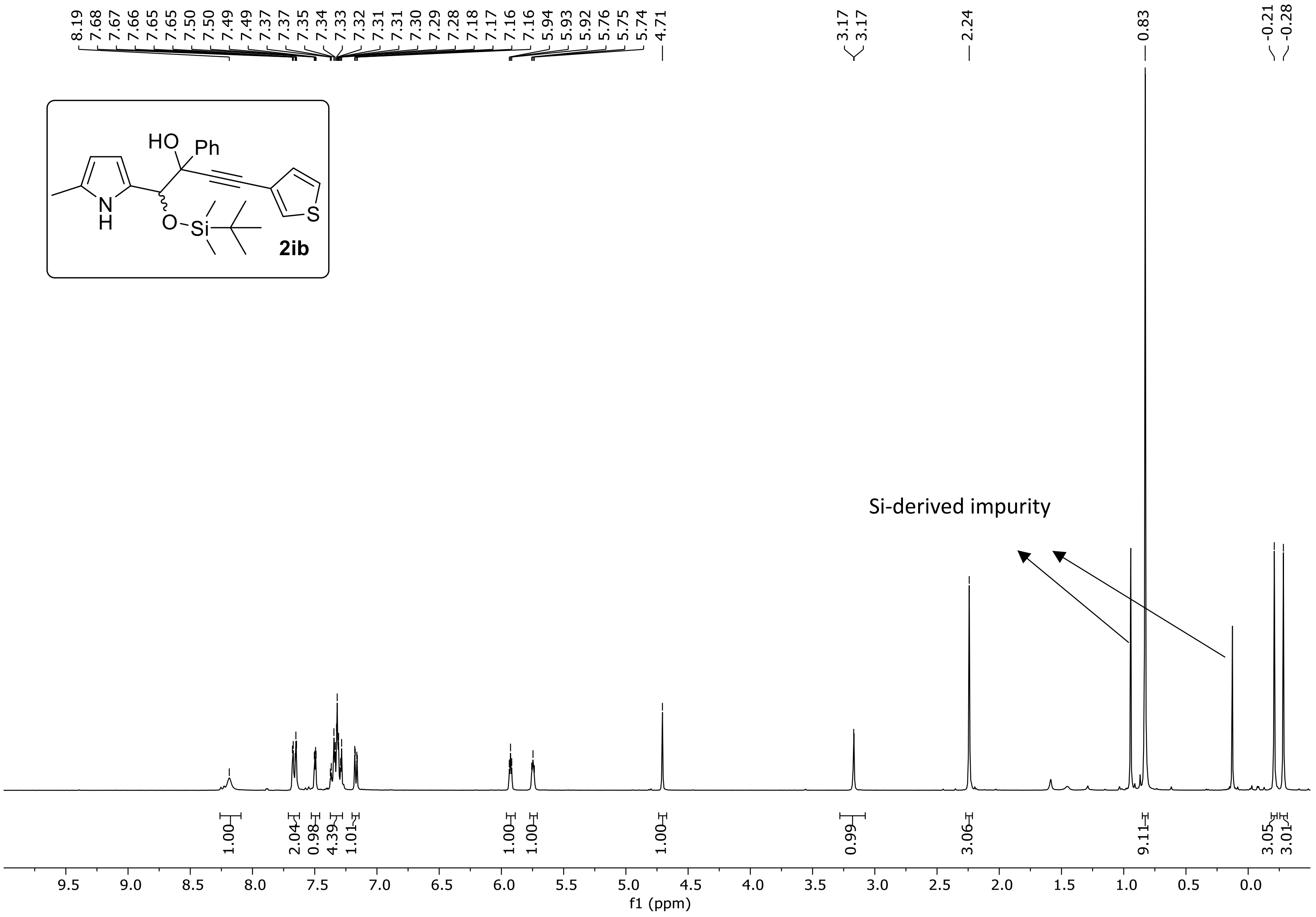

Figure S112: ^{13}C NMR of compound **2ib** in $CDCl_3$ at 75.4 MHz.

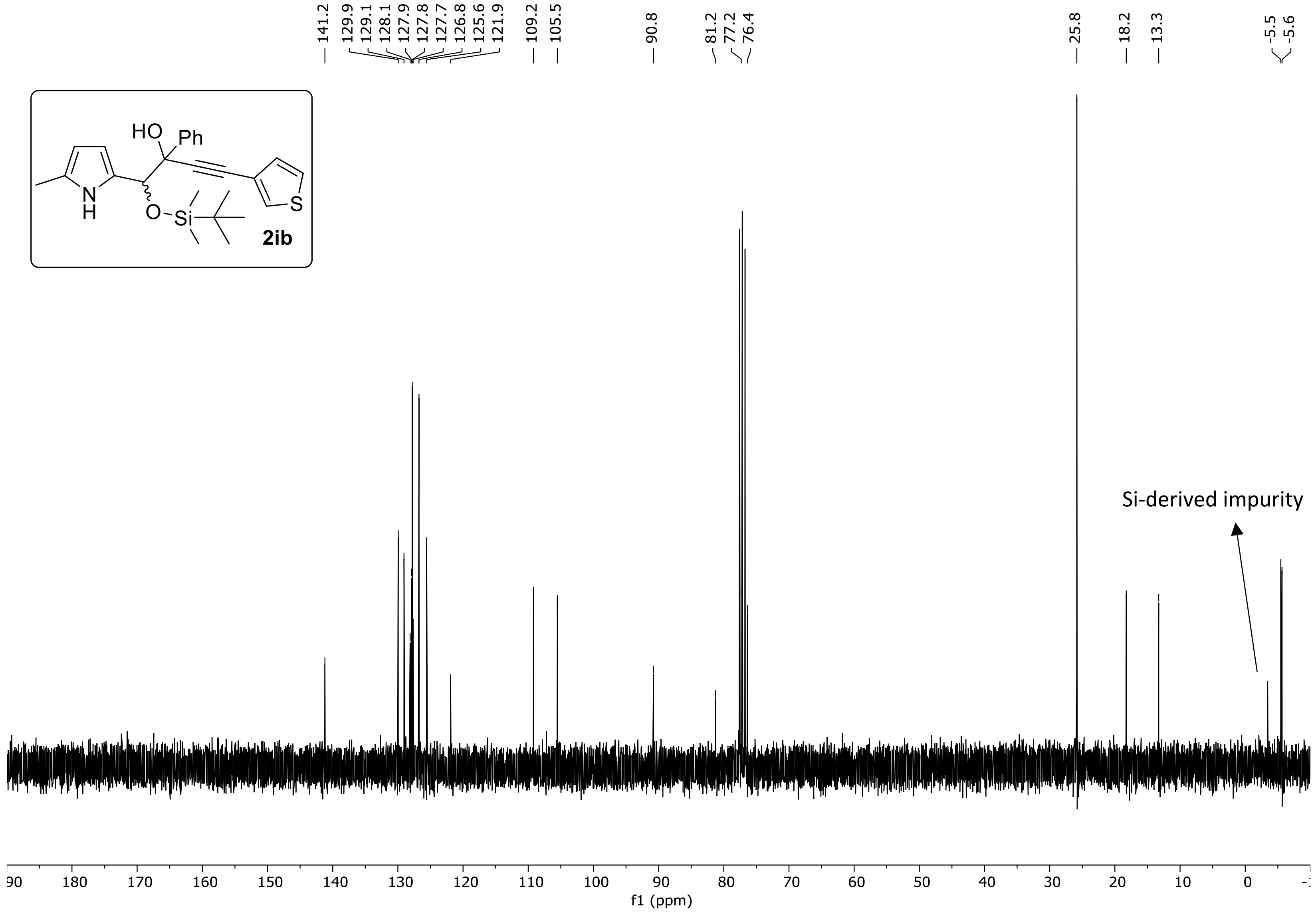

Figure S113: ^{1}H NMR of compound **2ja** in CDCl$_3$ at 300 MHz.

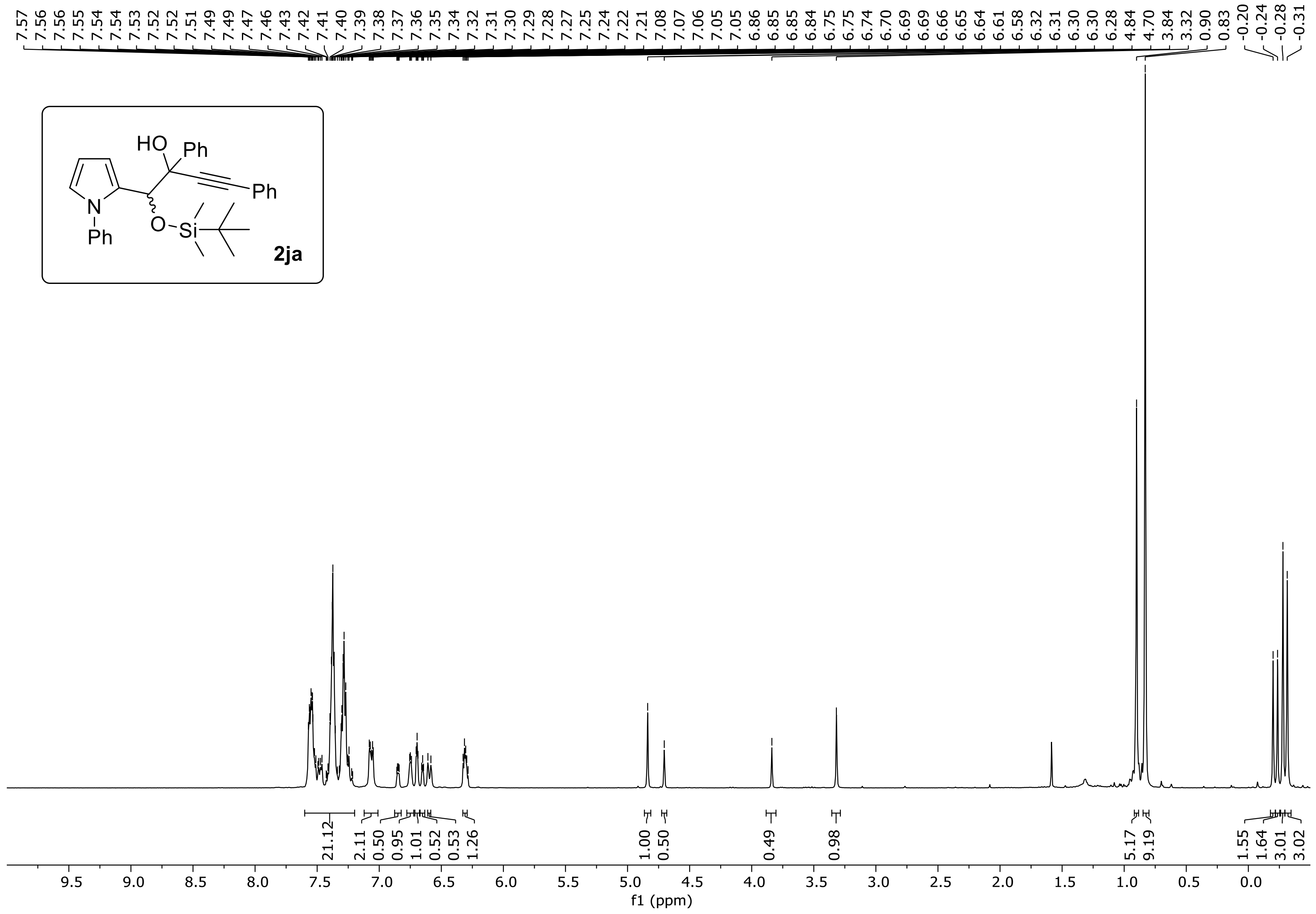

Figure S114: ^{13}C NMR of compound **2ja** in $CDCl_3$ at 75.4 MHz.

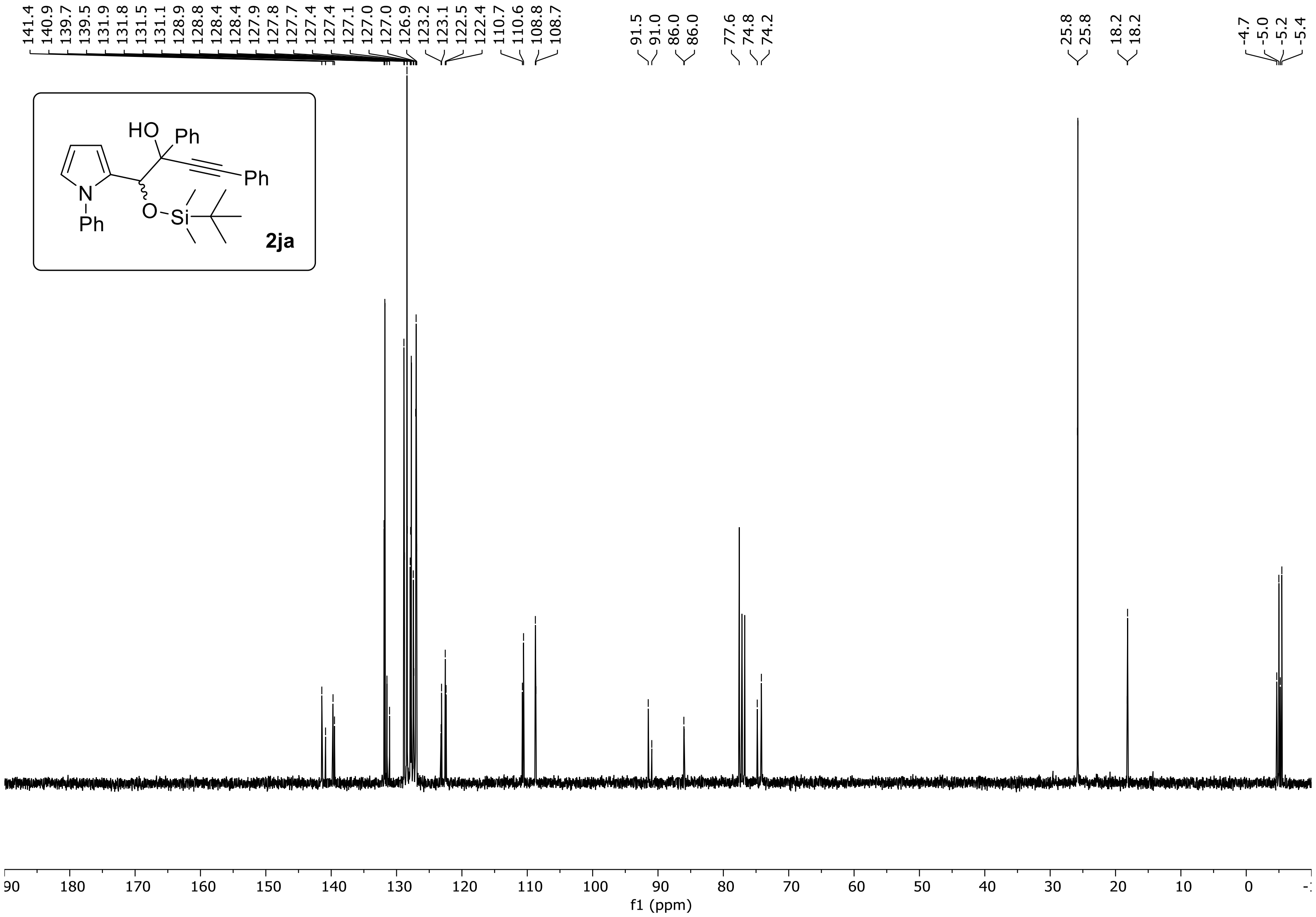

Figure S115: ^{1}H NMR of compound **2jh** in $CDCl_3$ at 300 MHz.

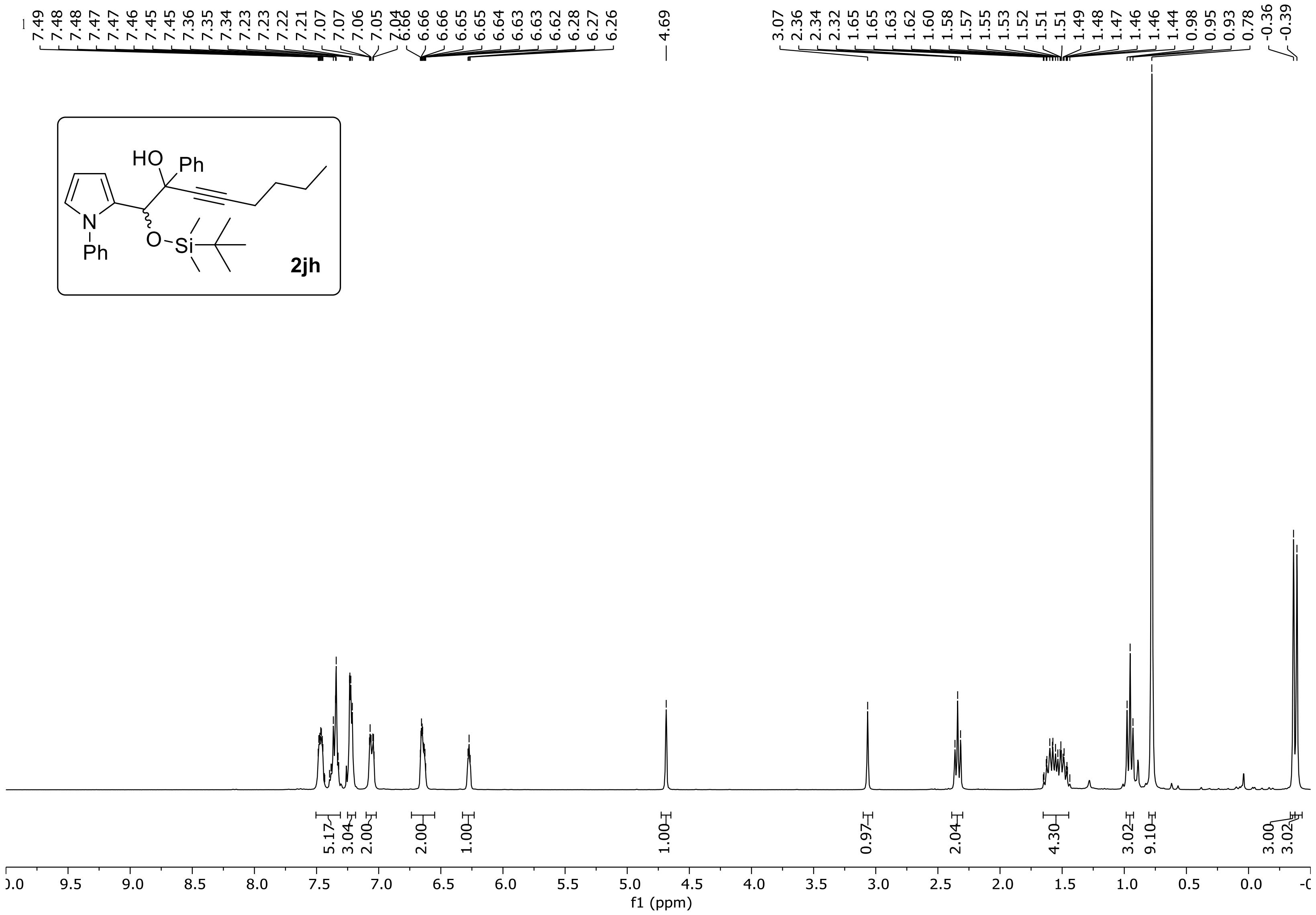

Figure S116: ^{13}C NMR of compound **2jh** in $CDCl_3$ at 75.4 MHz.

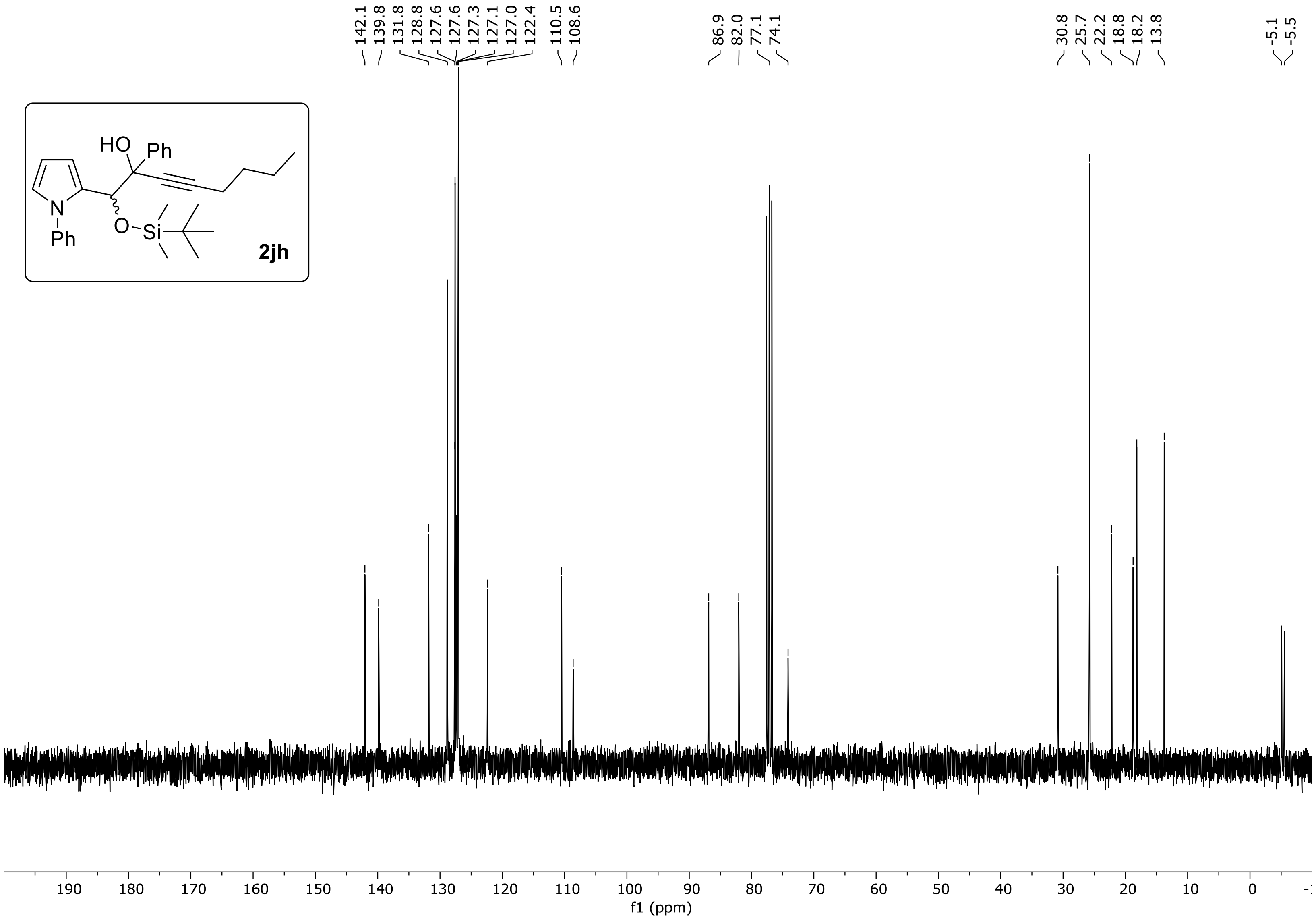

Figure S117: ^{1}H NMR of compound **3aa** in $CDCl_3$ at 300 MHz.

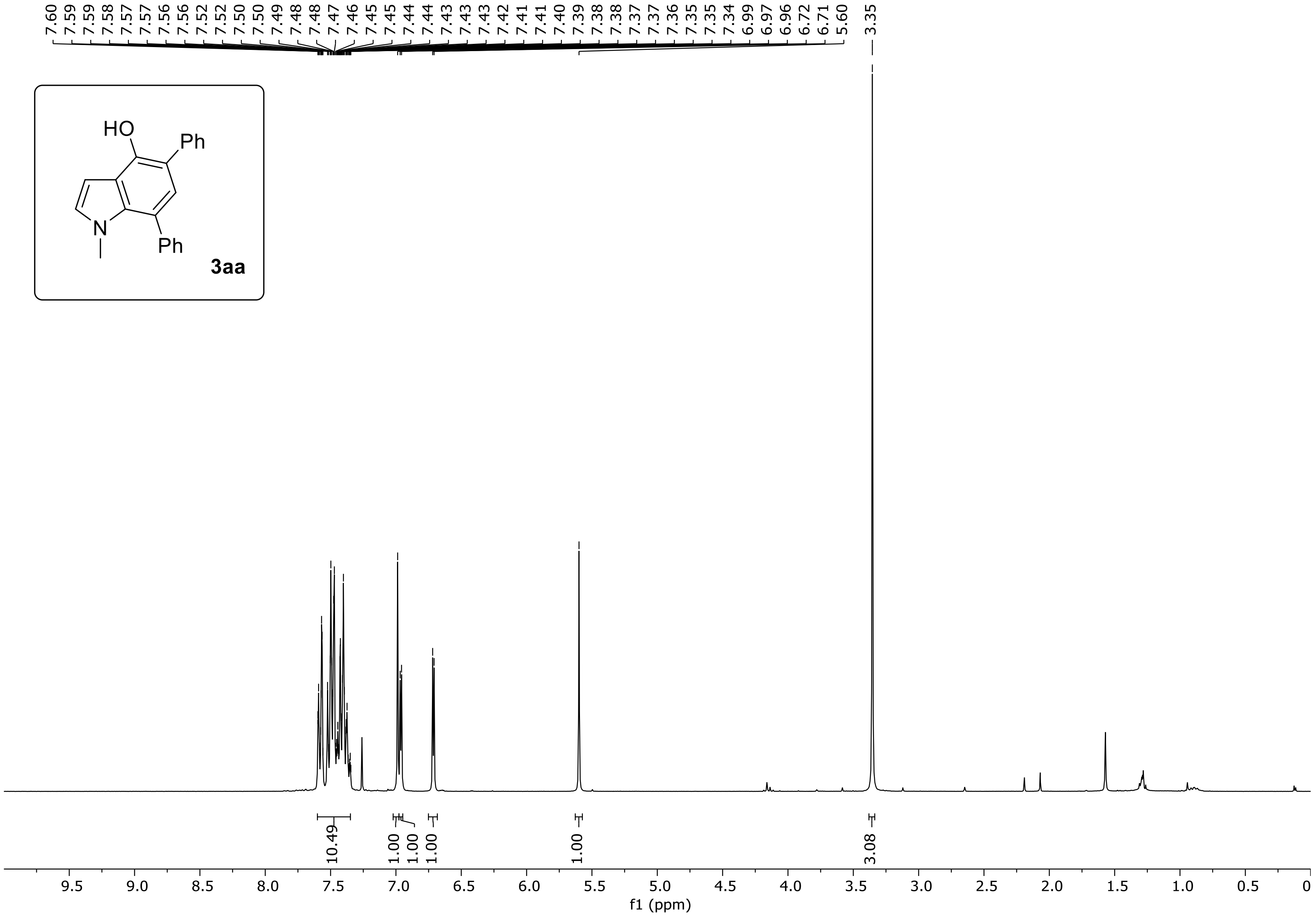

Figure S118: ^{13}C NMR of compound **3aa** in $CDCl_3$ at 75.4 MHz.

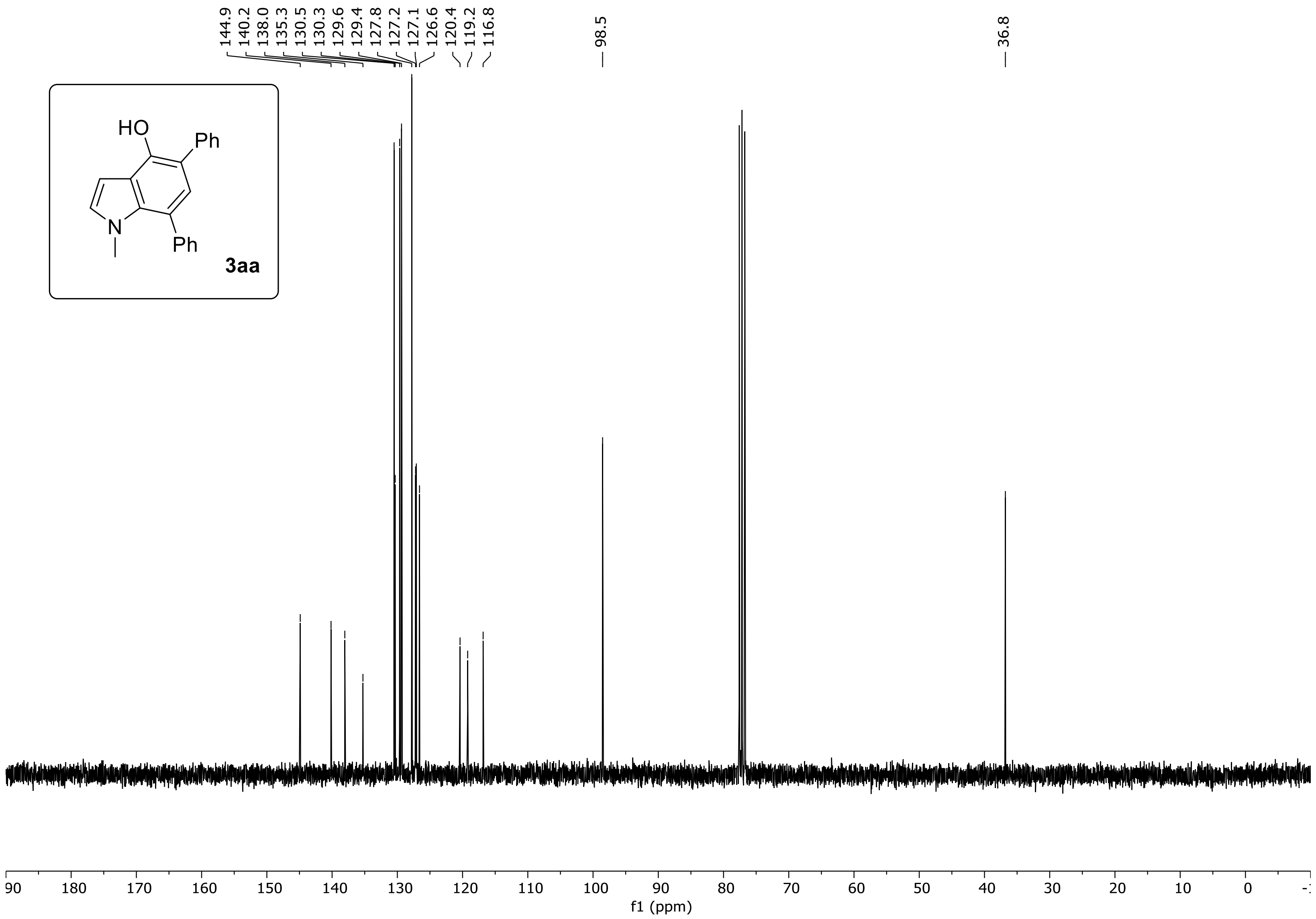

Figure S119: ^{1}H NMR of compound **3ab** in $CDCl_3$ at 75.4 MHz.

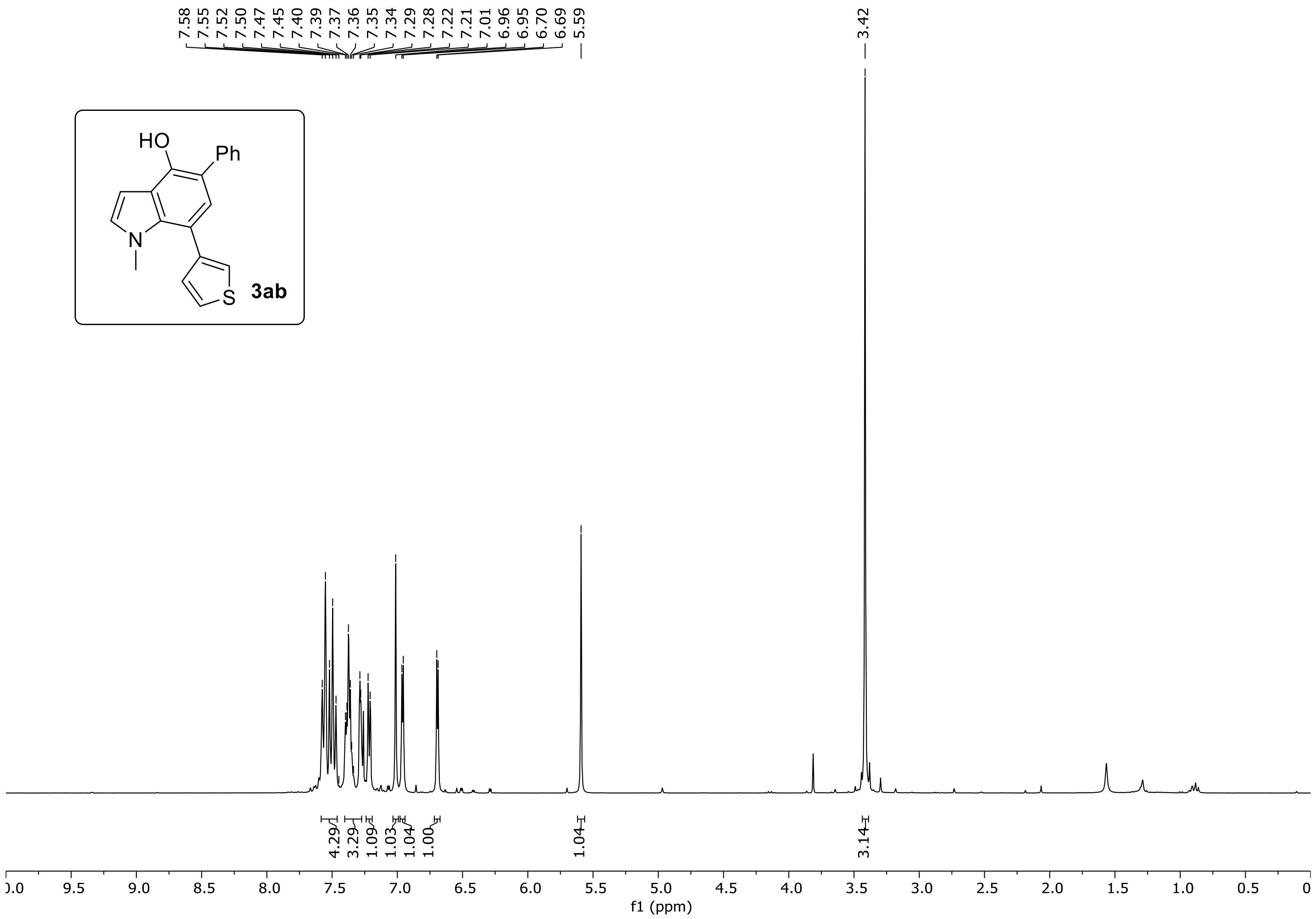

Figure S120: ^{13}C NMR of compound **3ab** in $CDCl_3$ at 75.4 MHz.

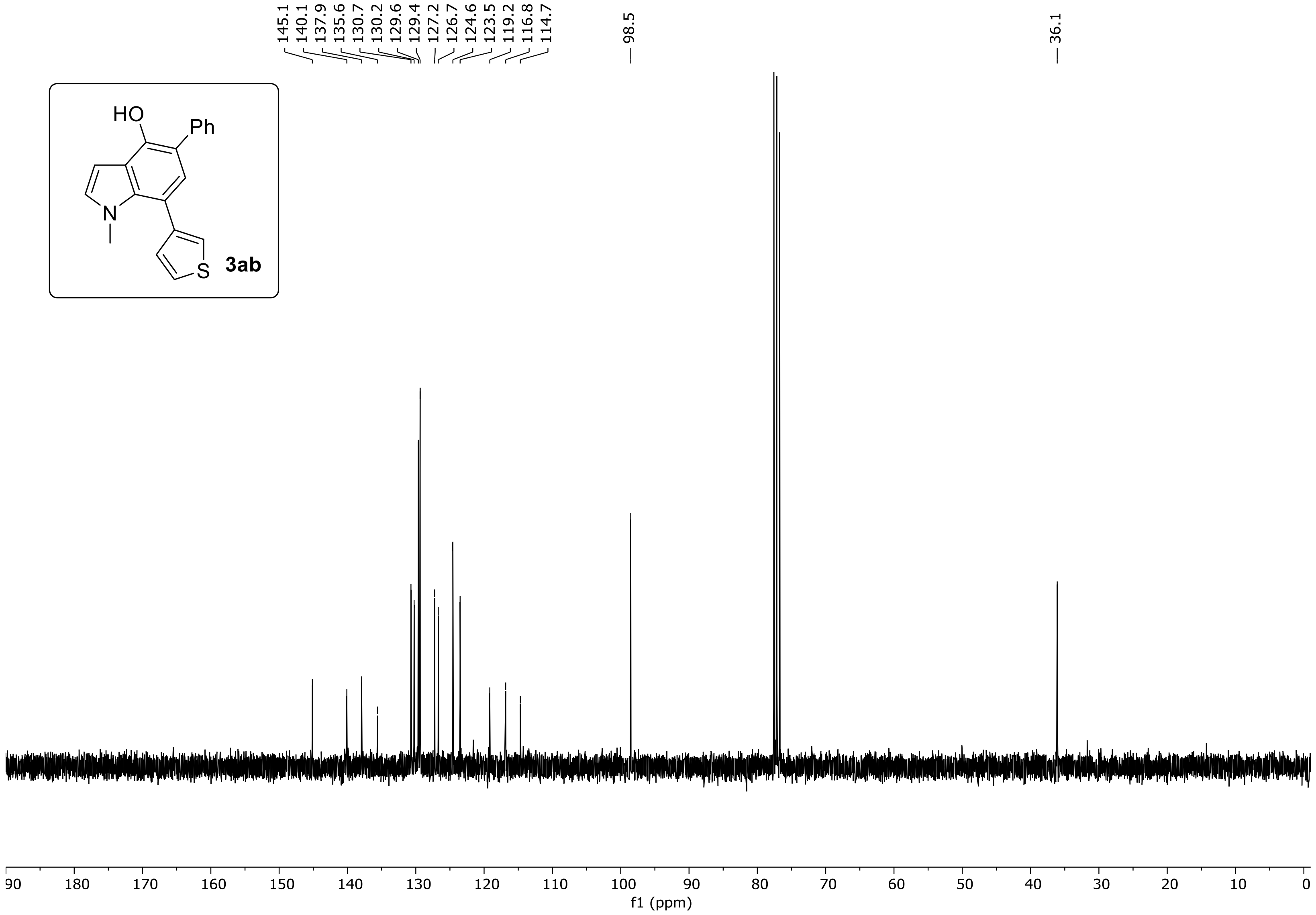

Figure S121: 1D NOE NMR of compound **3ab** in $CDCl_3$ at 300 MHz.

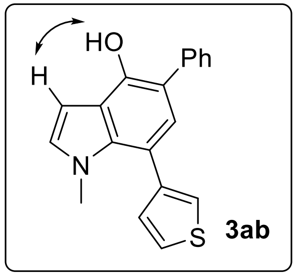

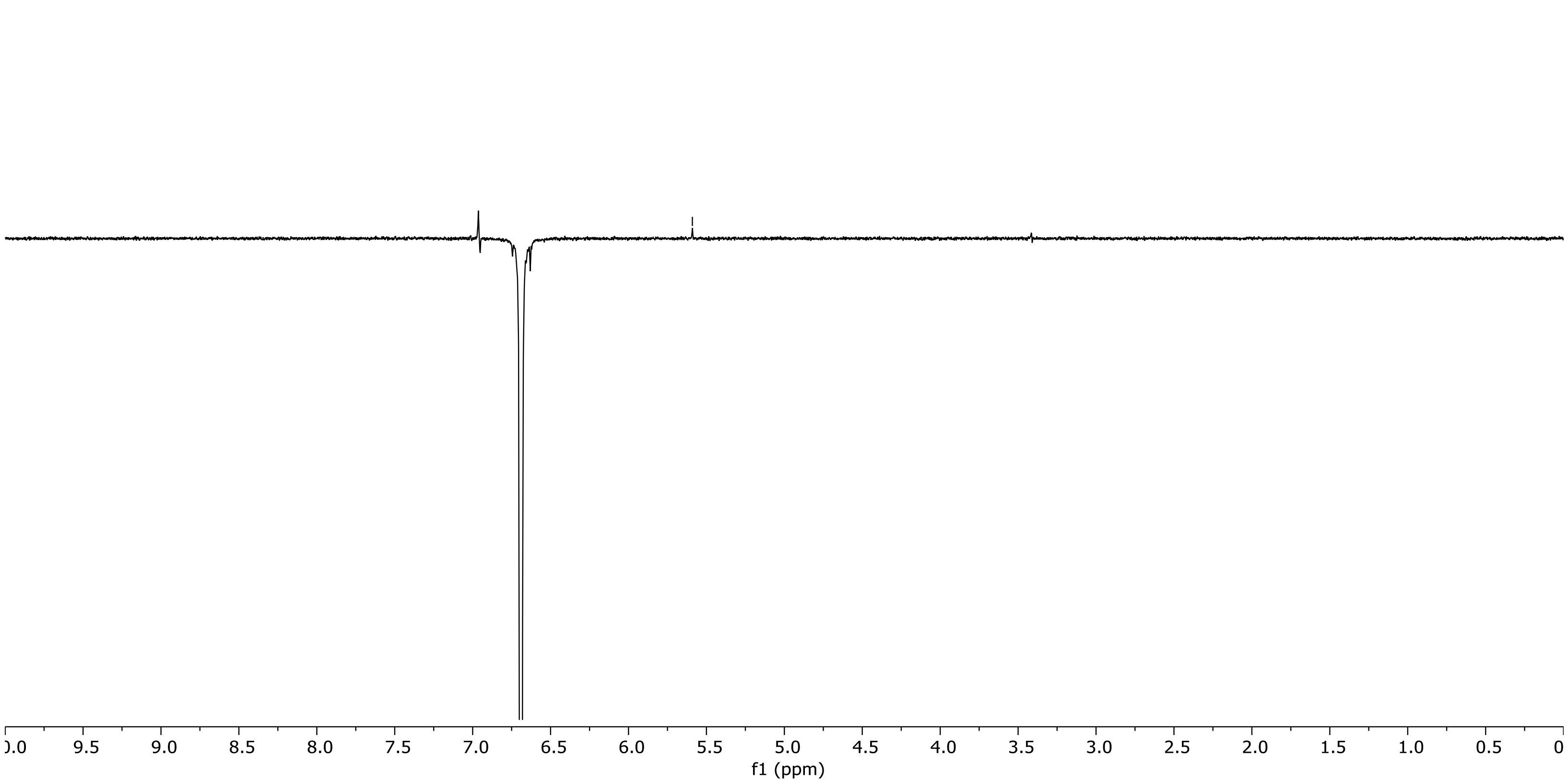

Figure S122: ^{1}H NMR of compound **4ab** in $CDCl_3$ at 300 MHz.

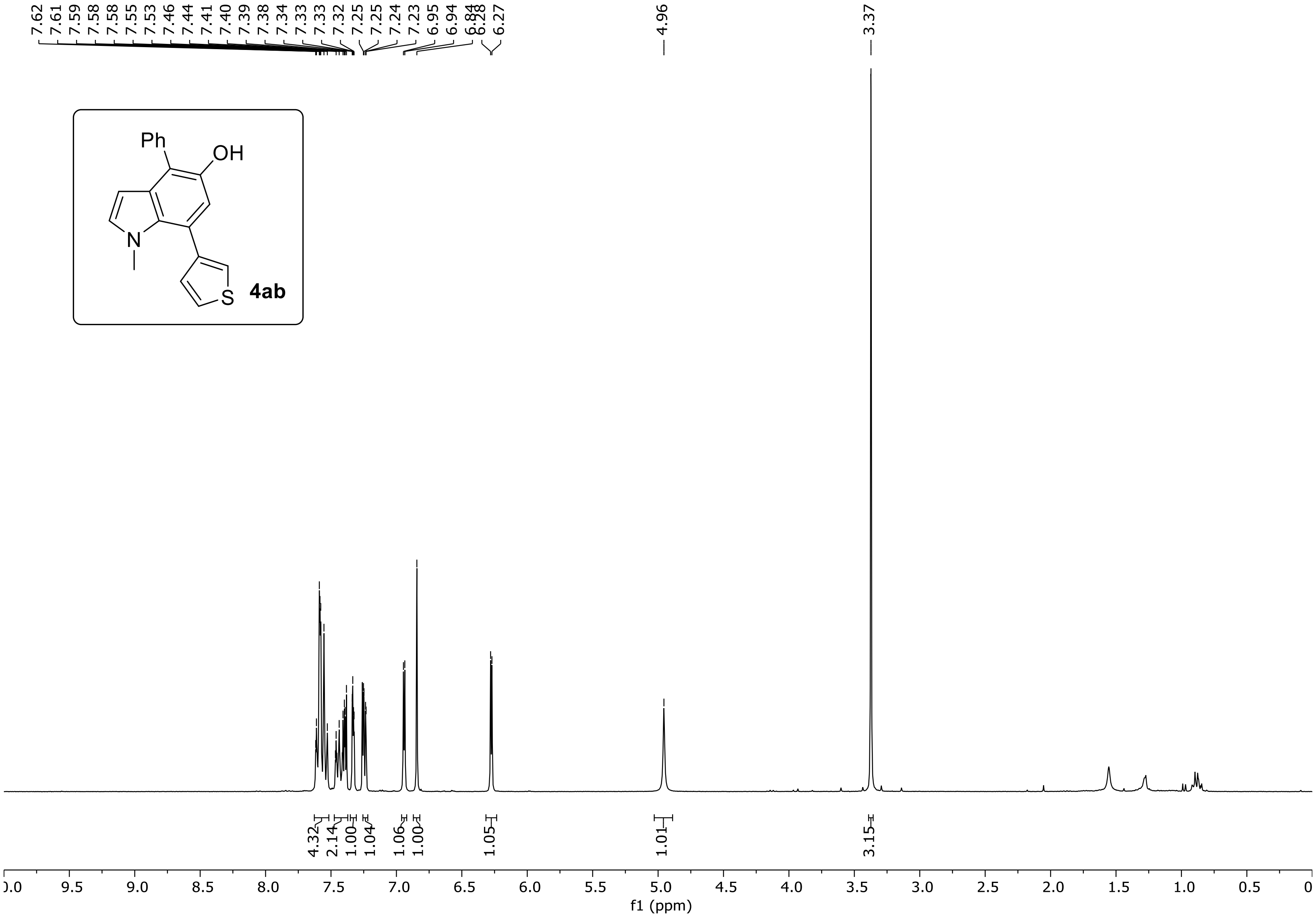

Figure S123: ^{13}C NMR of compound **4ab** in $CDCl_3$ at 125.7 MHz.

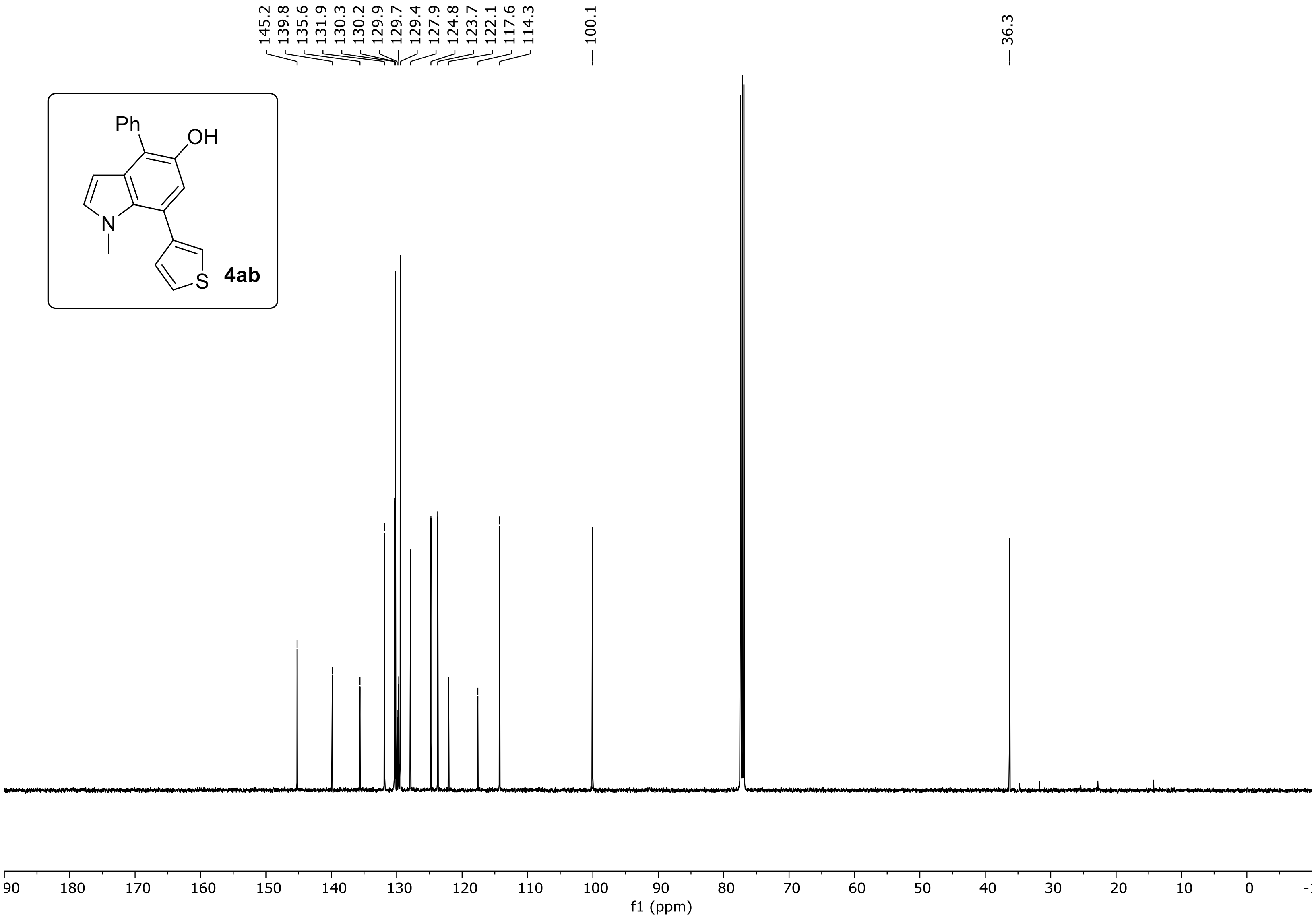

Figure S124: 1D NOE NMR of compound **4ab** in $CDCl_3$ at 300 MHz.

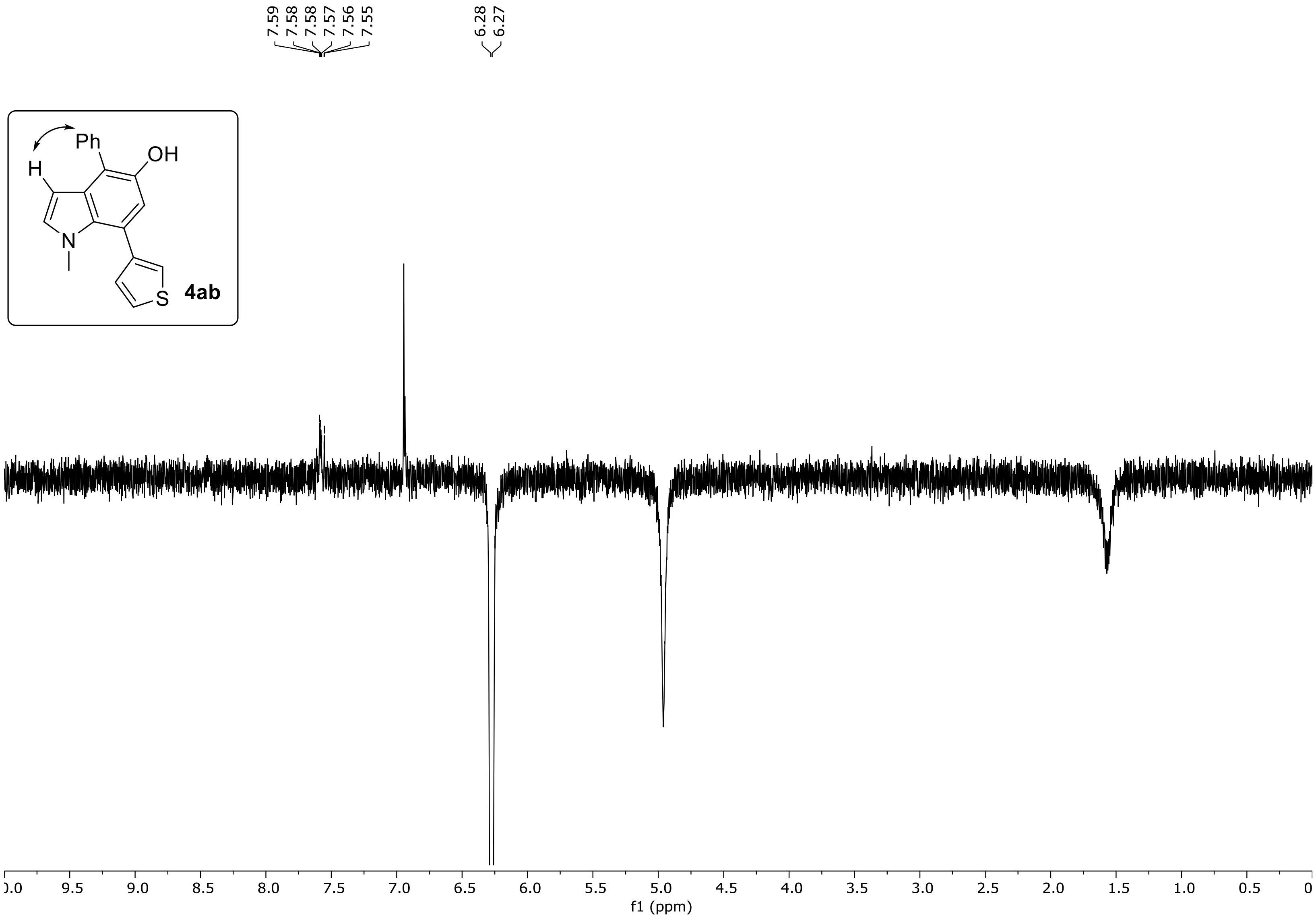

Figure S125: ^{1}H NMR of compound **3af** in $CDCl_3$ at 300 MHz.

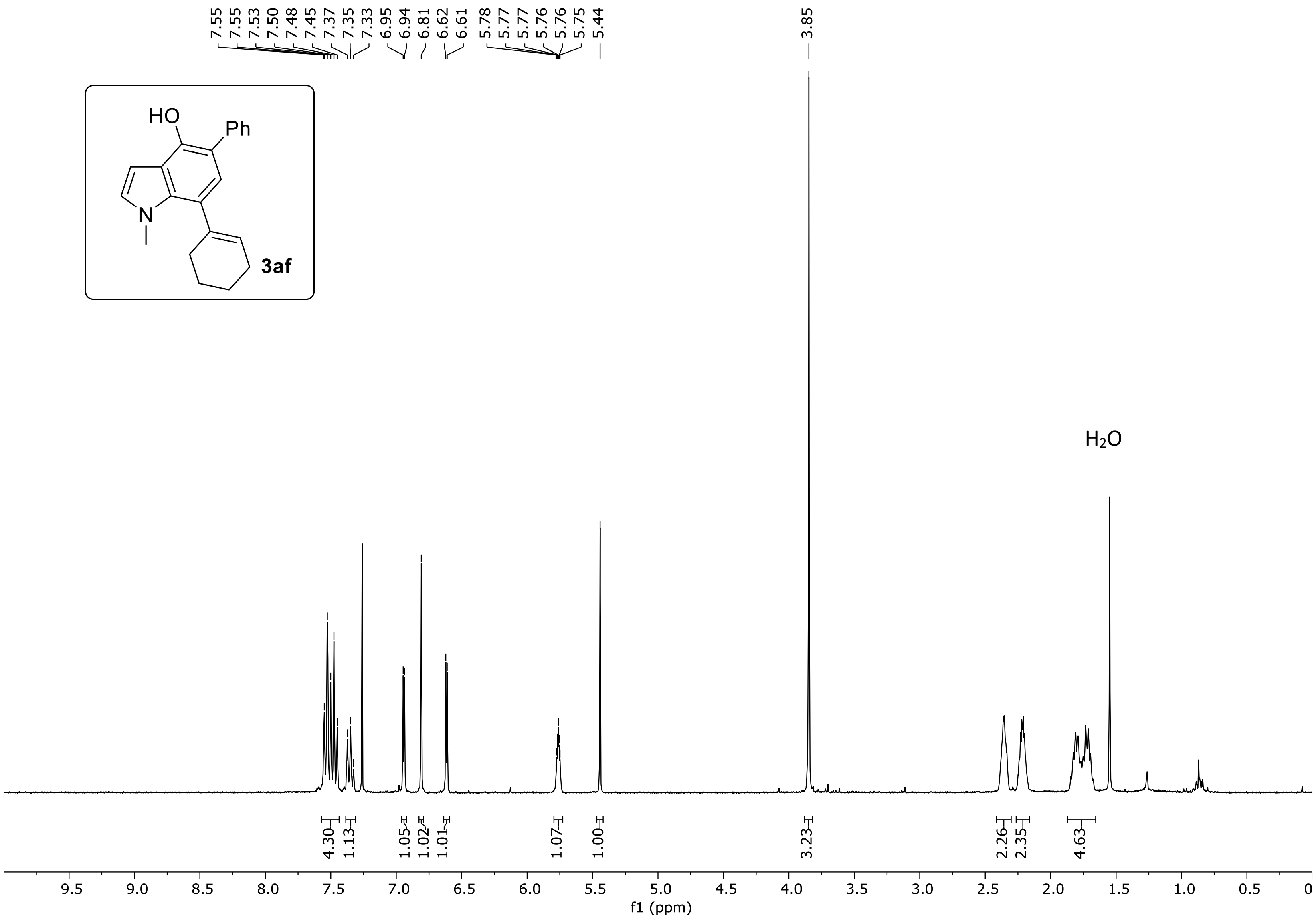

Figure S126: ^{13}C NMR of compound **3af** in $CDCl_3$ at 75.4 MHz.

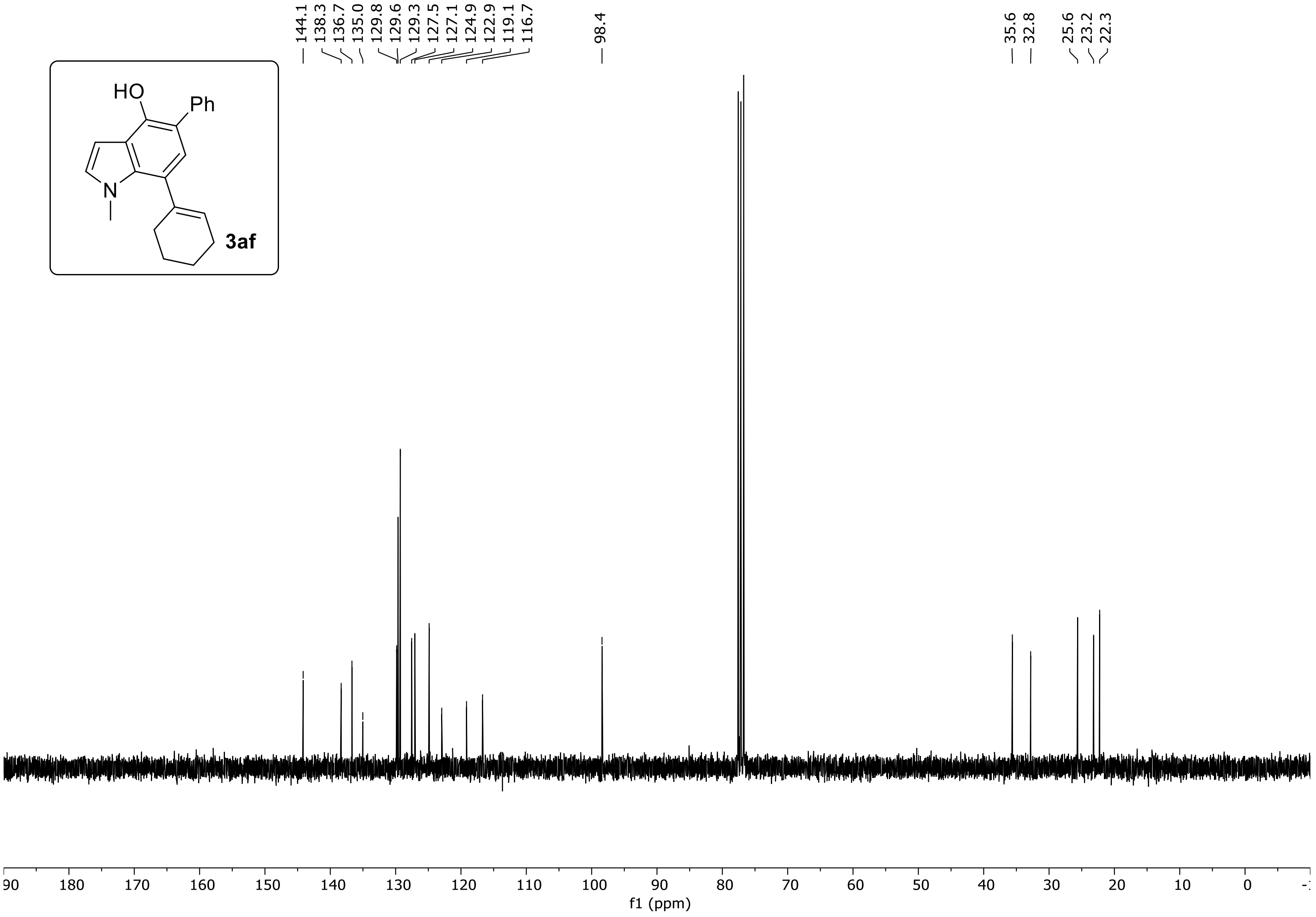

Figure S127a: 1D NOE NMR of compound **3af** in $CDCl_3$ at 300 MHz.

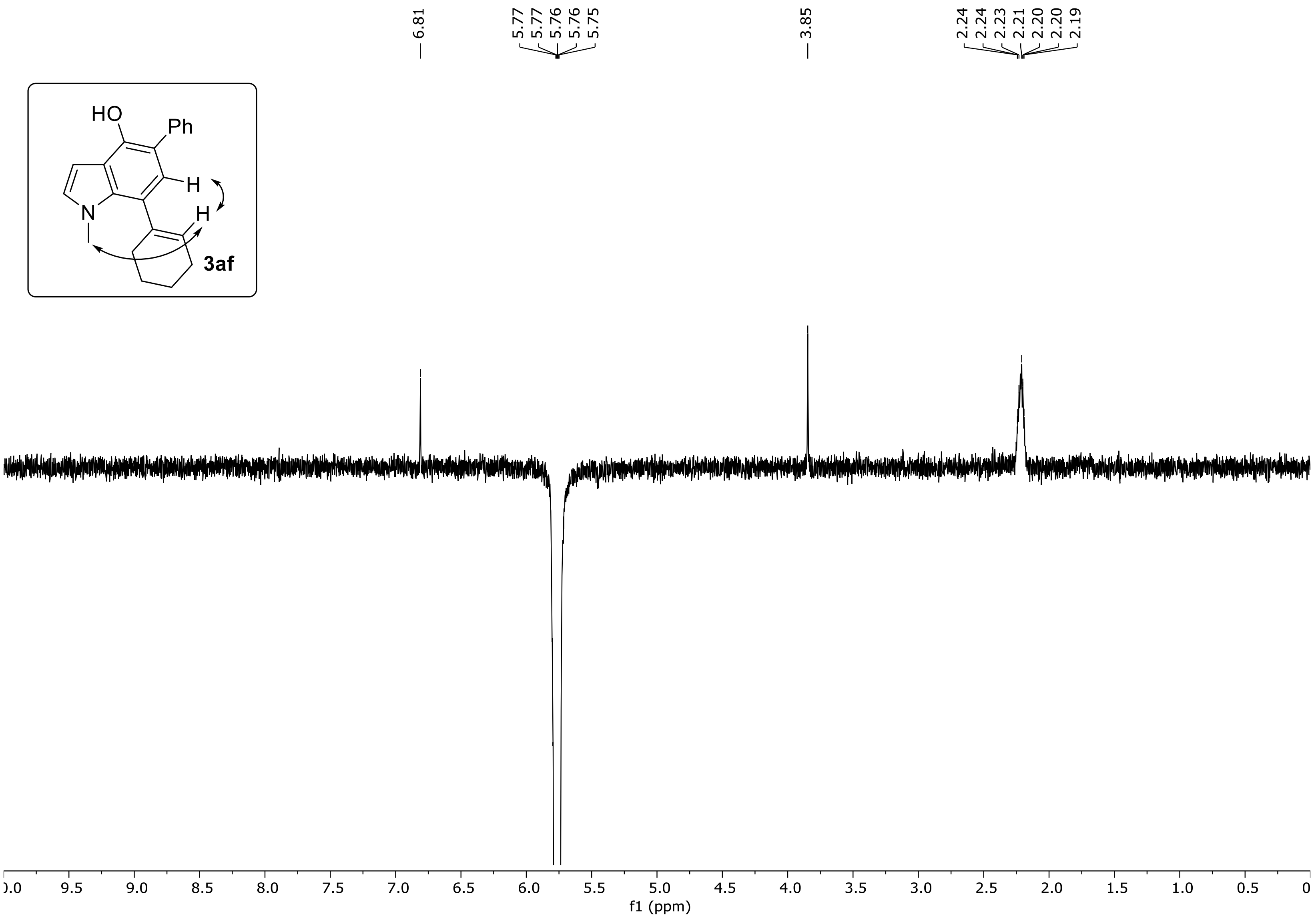

Figure S127b: 1D NOE NMR of compound **3af** in $CDCl_3$ at 300 MHz.

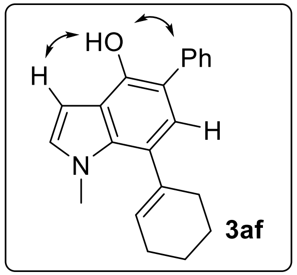

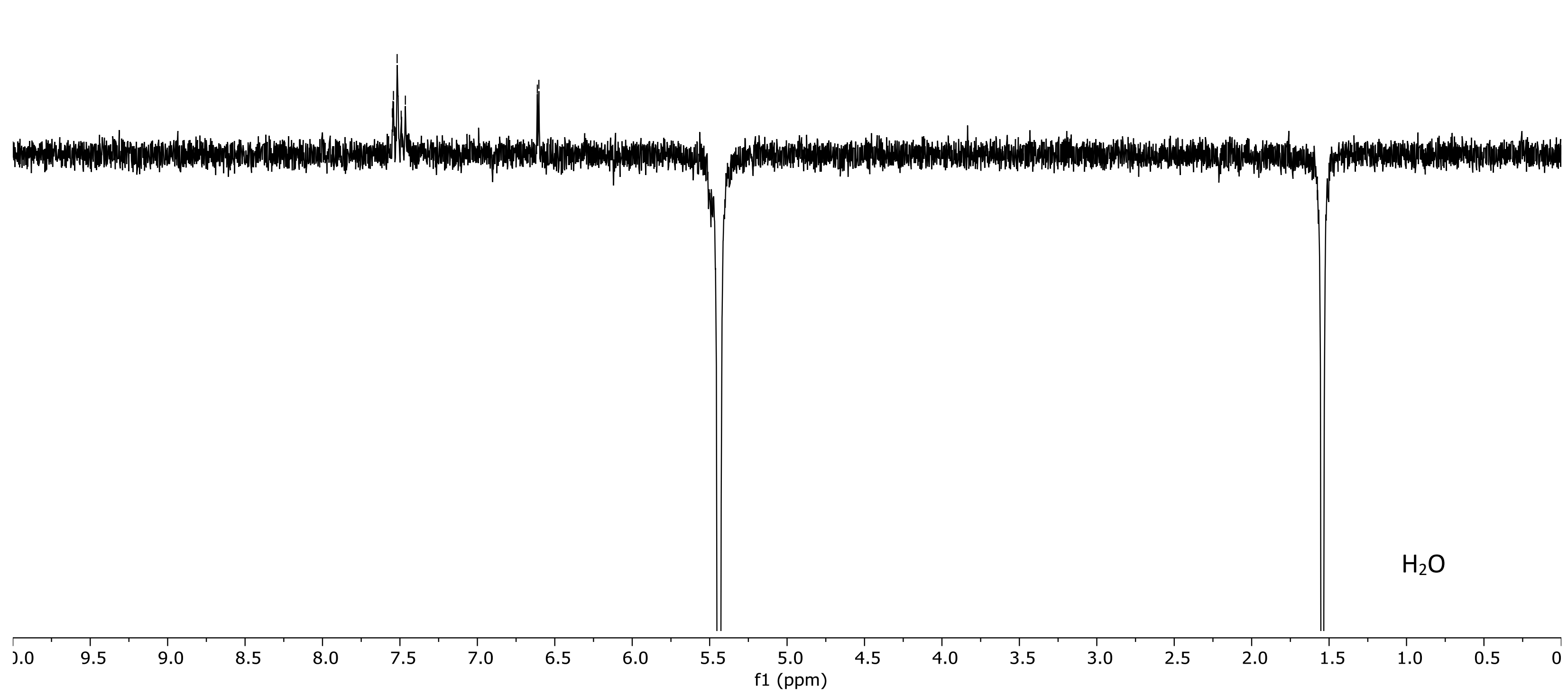

Figure S128: ^{1}H NMR of compound **4af** in $CDCl_3$ at 300 MHz.

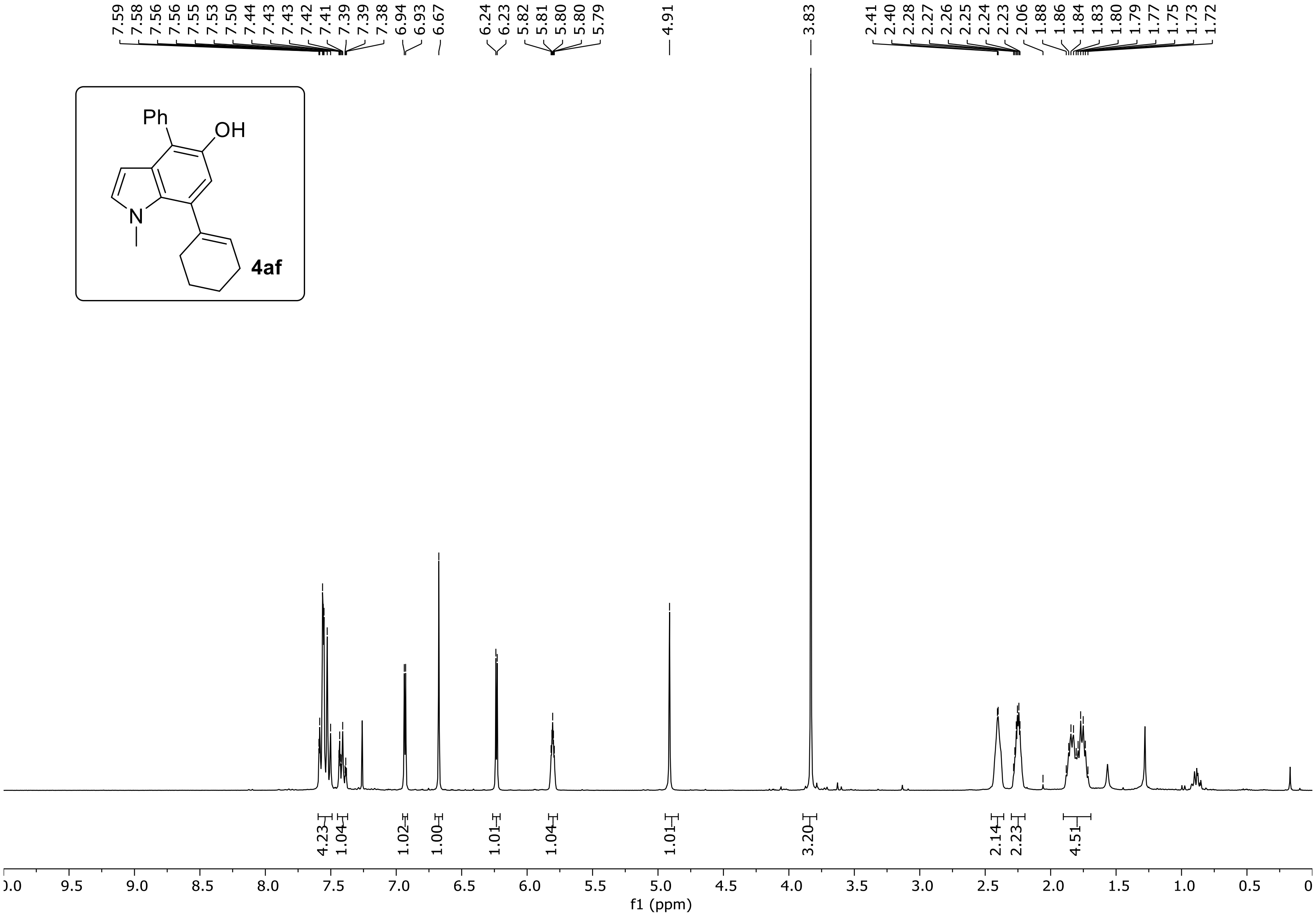

Figure S129: ^{13}C NMR of compound **4af** in $CDCl_3$ at 75.4 MHz.

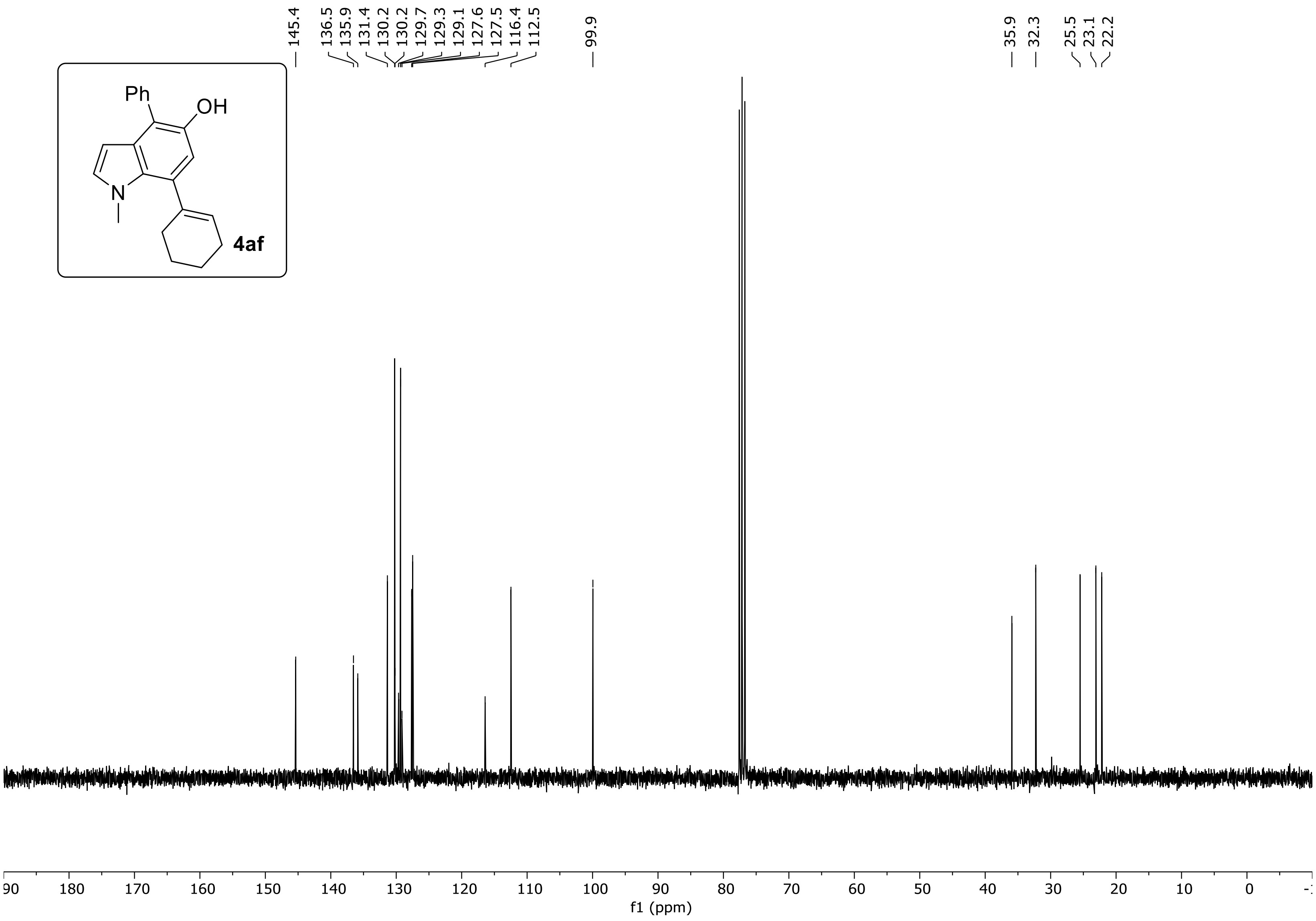

Figure S130: 1D NOE NMR of compound **4af** in $CDCl_3$ at 300 MHz.

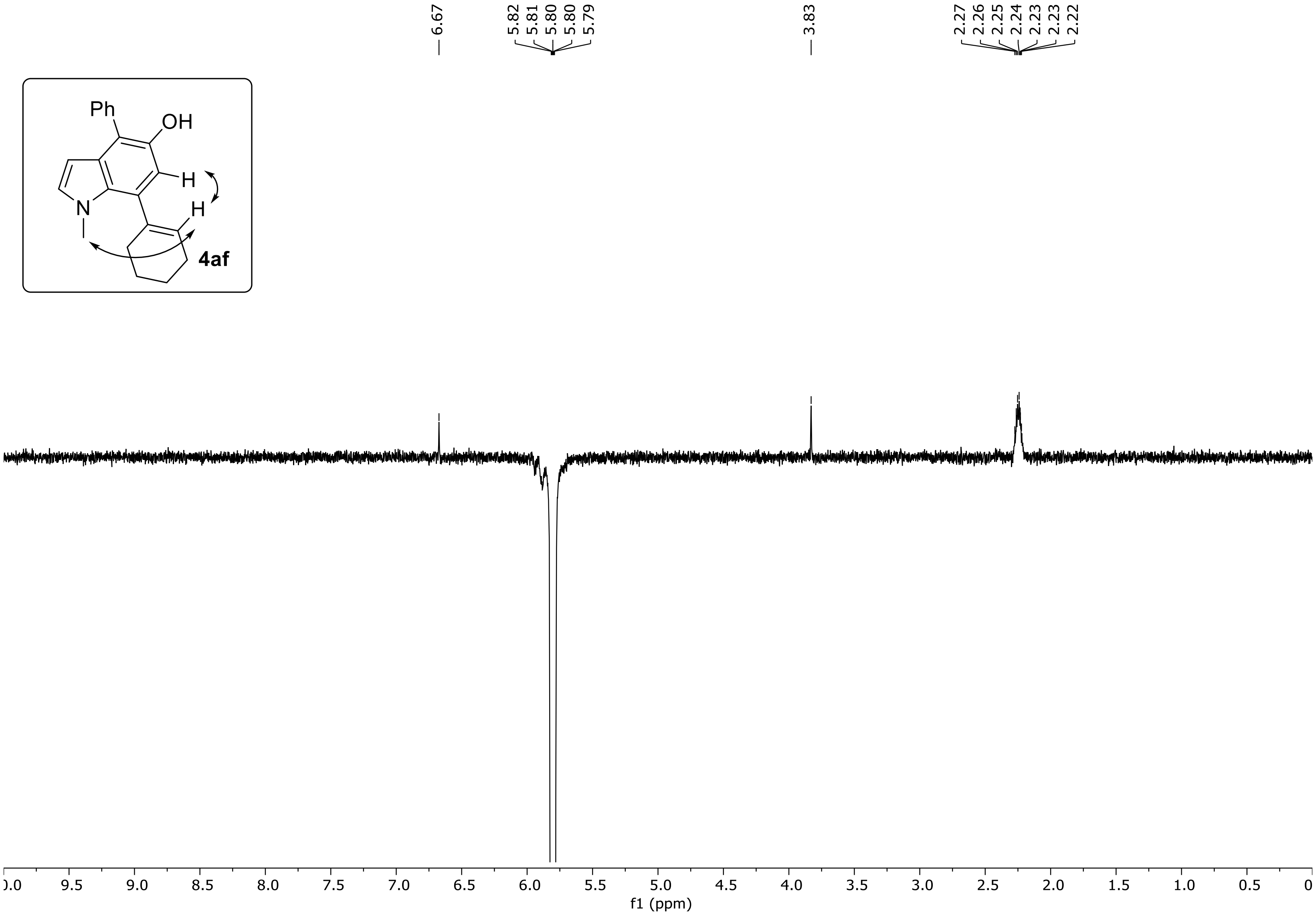

Figure S131: ^{1}H NMR of compound **3ah** in $CDCl_3$ at 500 MHz.

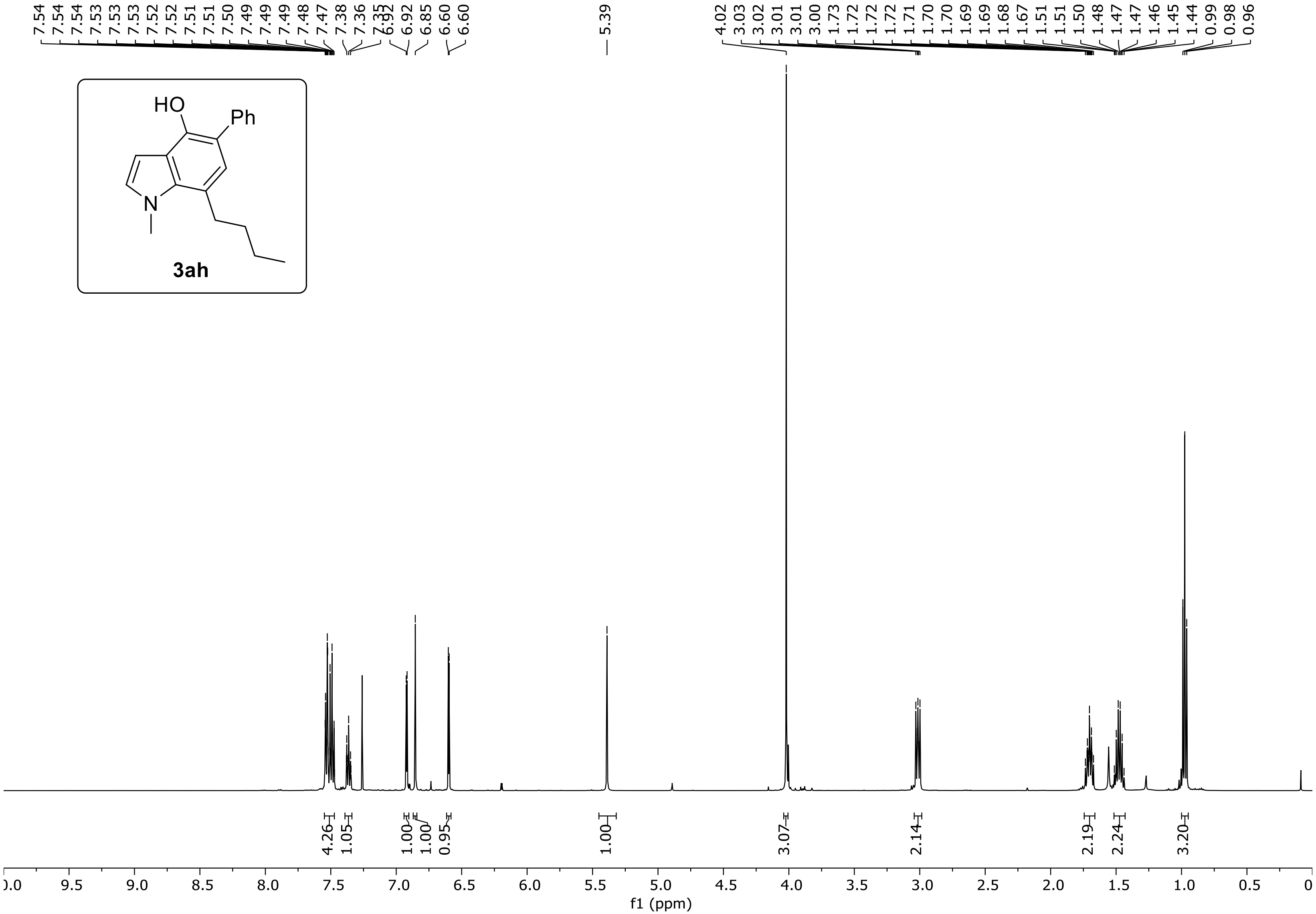

Figure S132: ^{13}C NMR of compound **3ah** in $CDCl_3$ at 75.4 MHz.

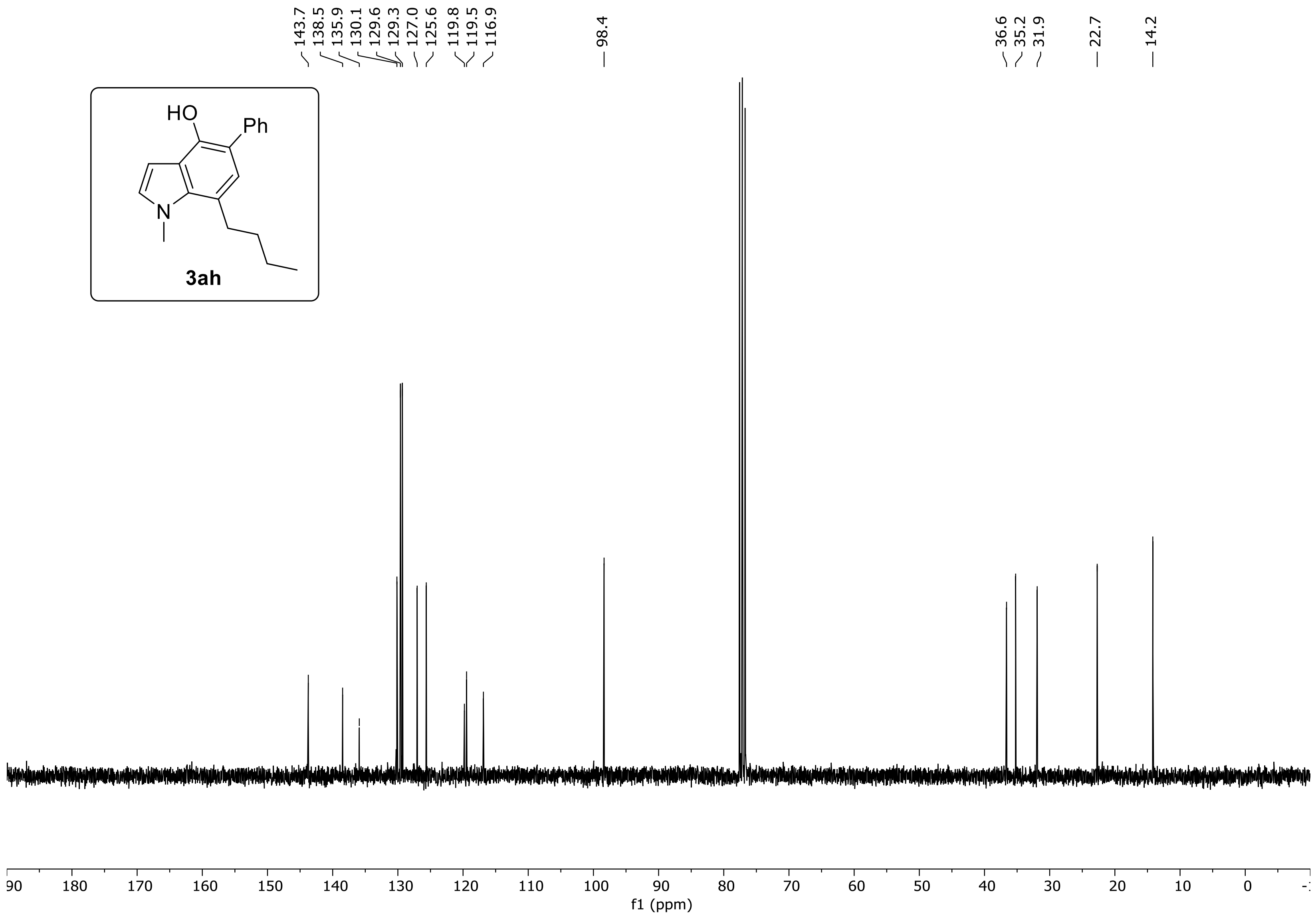

Figure S133: 1D NOE NMR of compound **3ah** in $CDCl_3$ at 500 MHz.

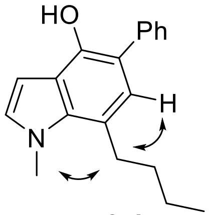

3ah

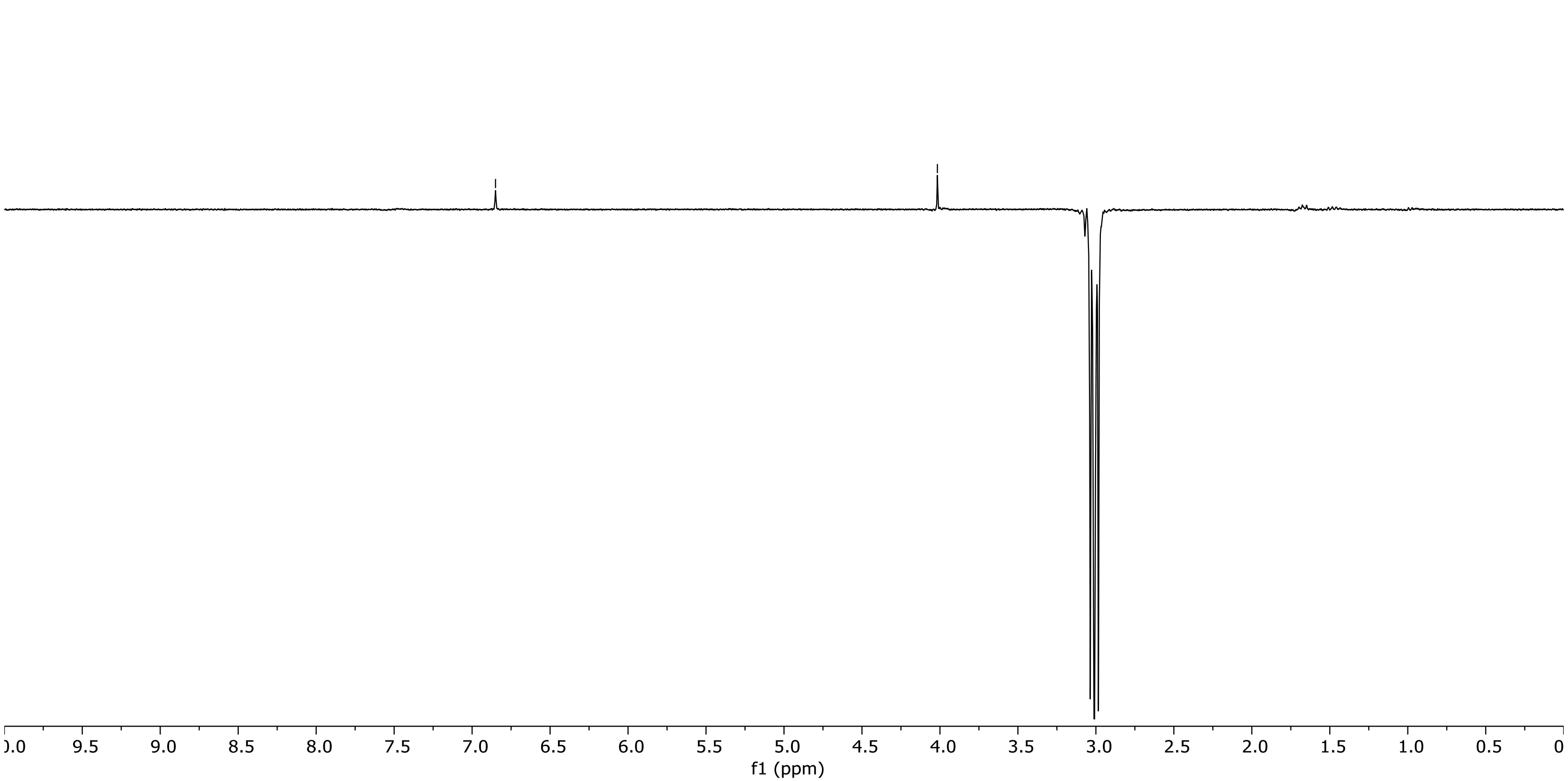

Figure S134: ^{1}H NMR of compound **4ah** in $CDCl_3$ at 500 MHz.

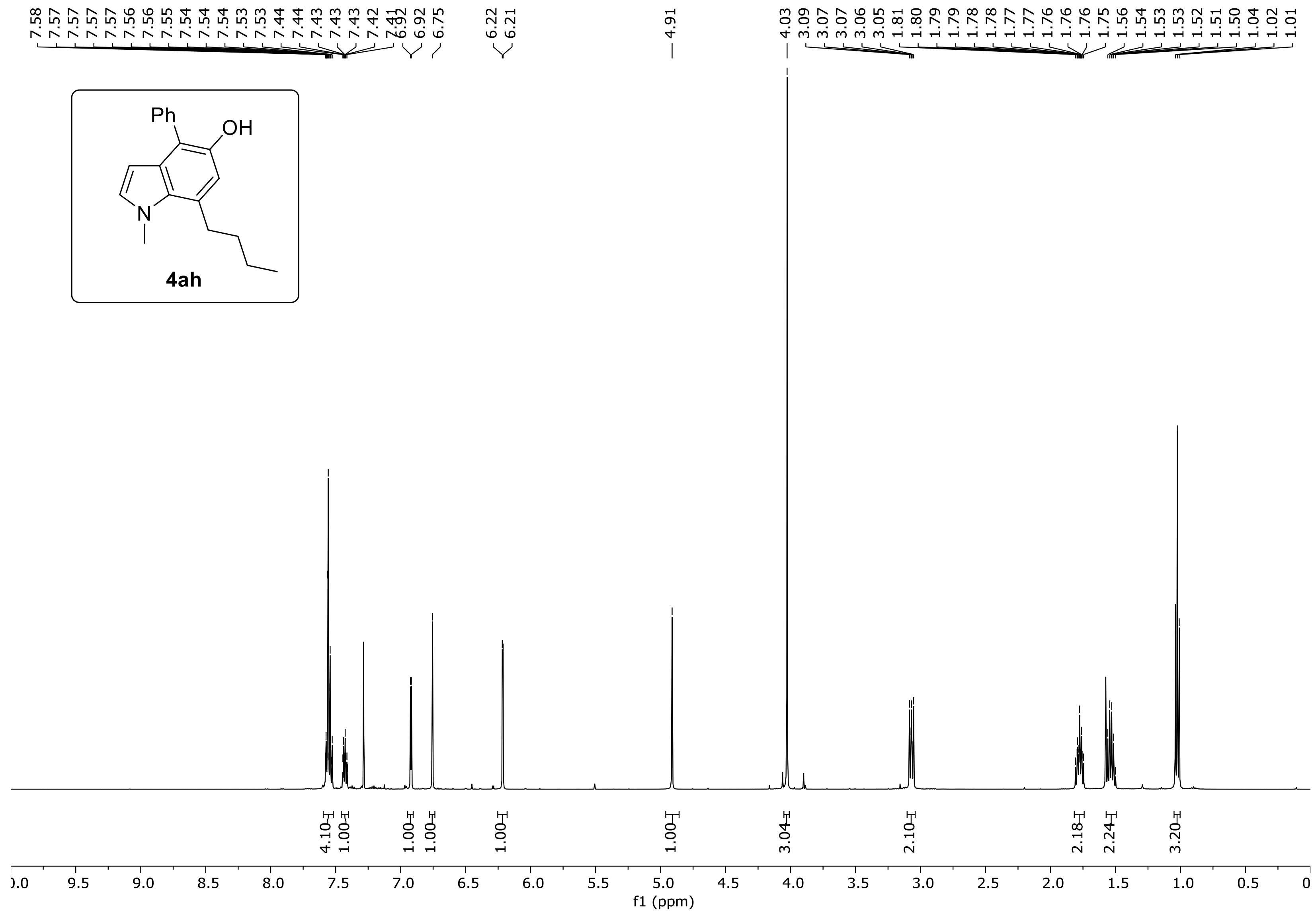

Figure S135: ^{13}C NMR of compound **4ah** in $CDCl_3$ at 125.7 MHz.

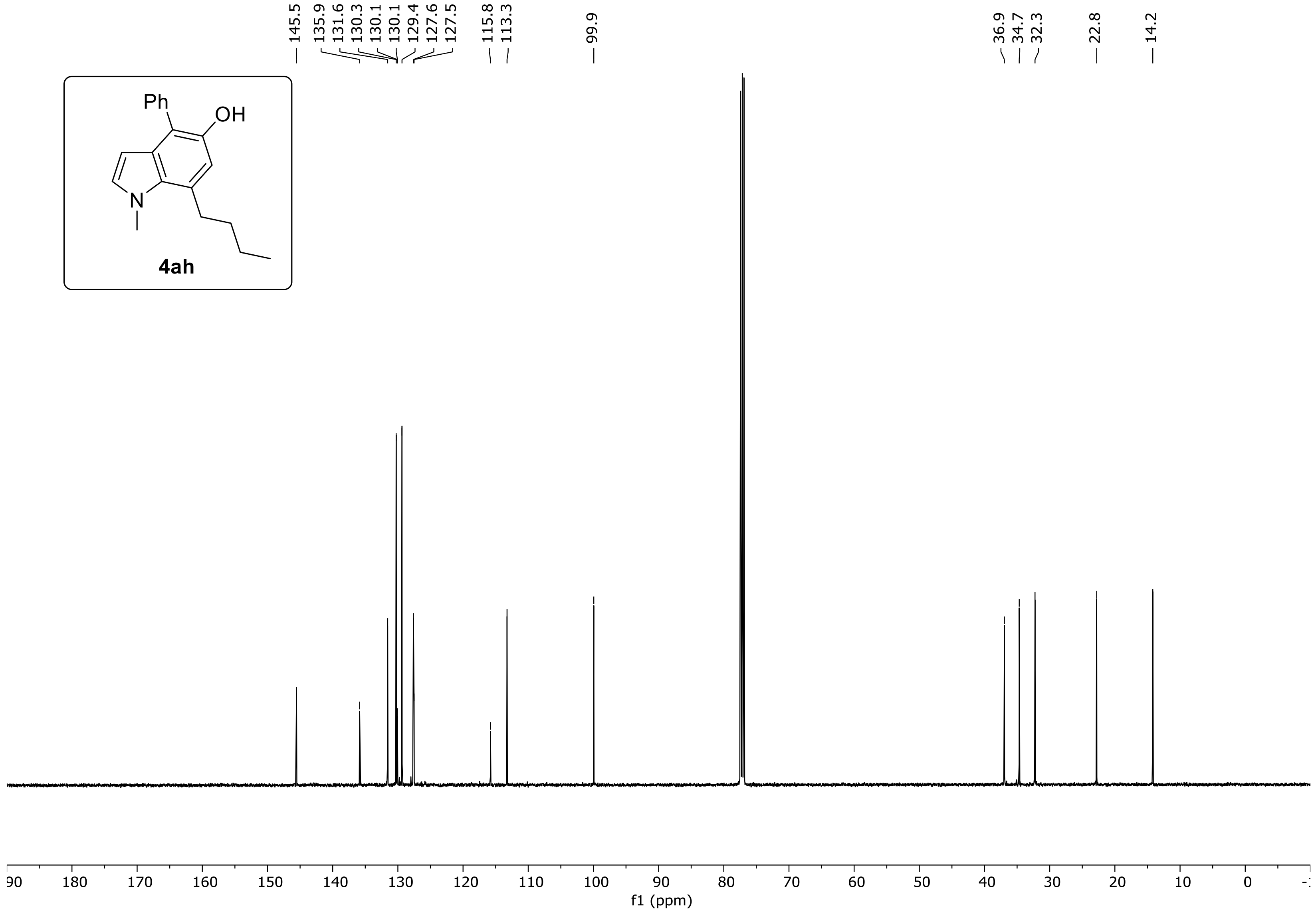

Figure S136: 1D NOE NMR of compound **4ah** in $CDCl_3$ at 500 MHz.

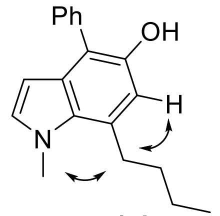

4ah

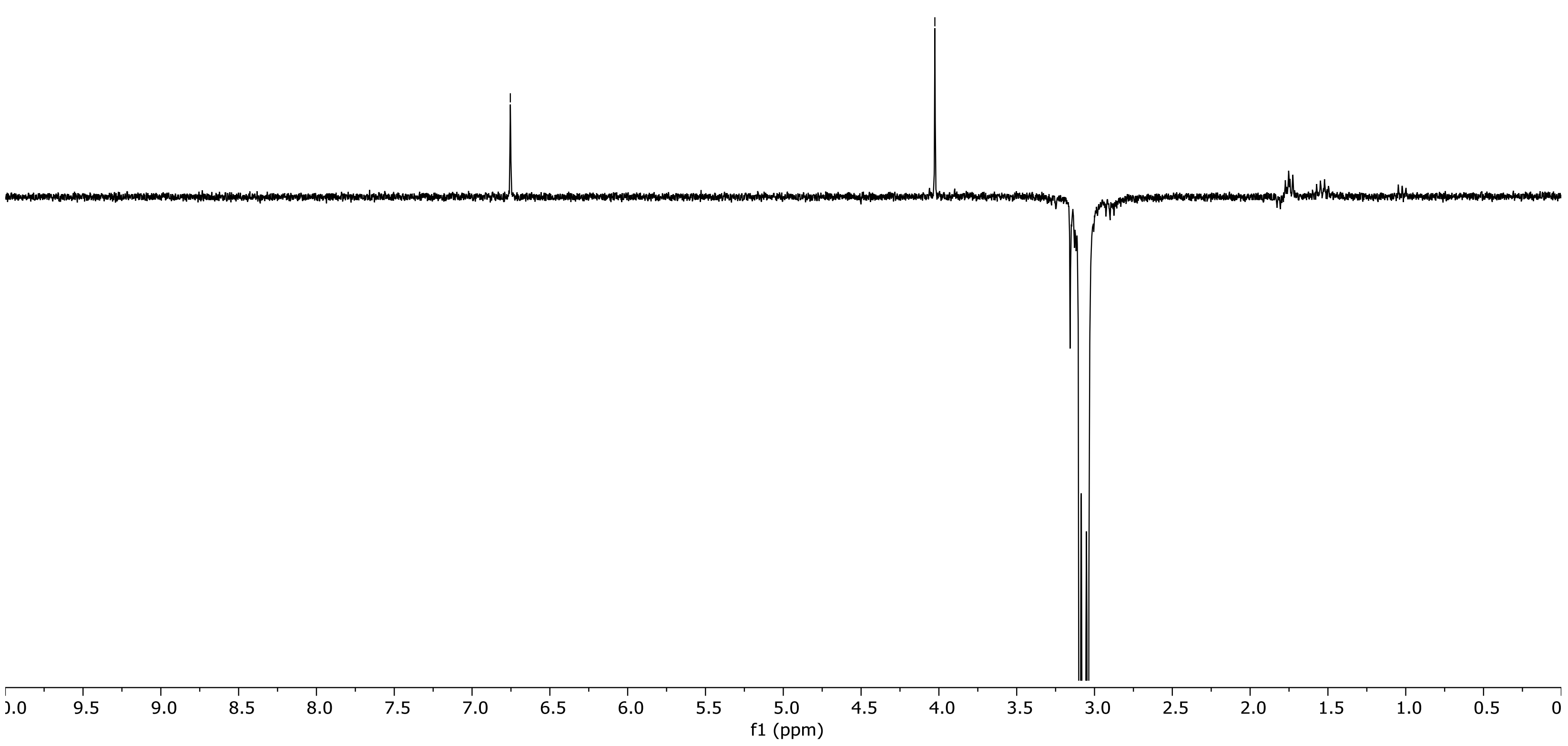

Figure S137: ^{1}H NMR of compound **6ah** in $CDCl_3$ at 300 MHz.

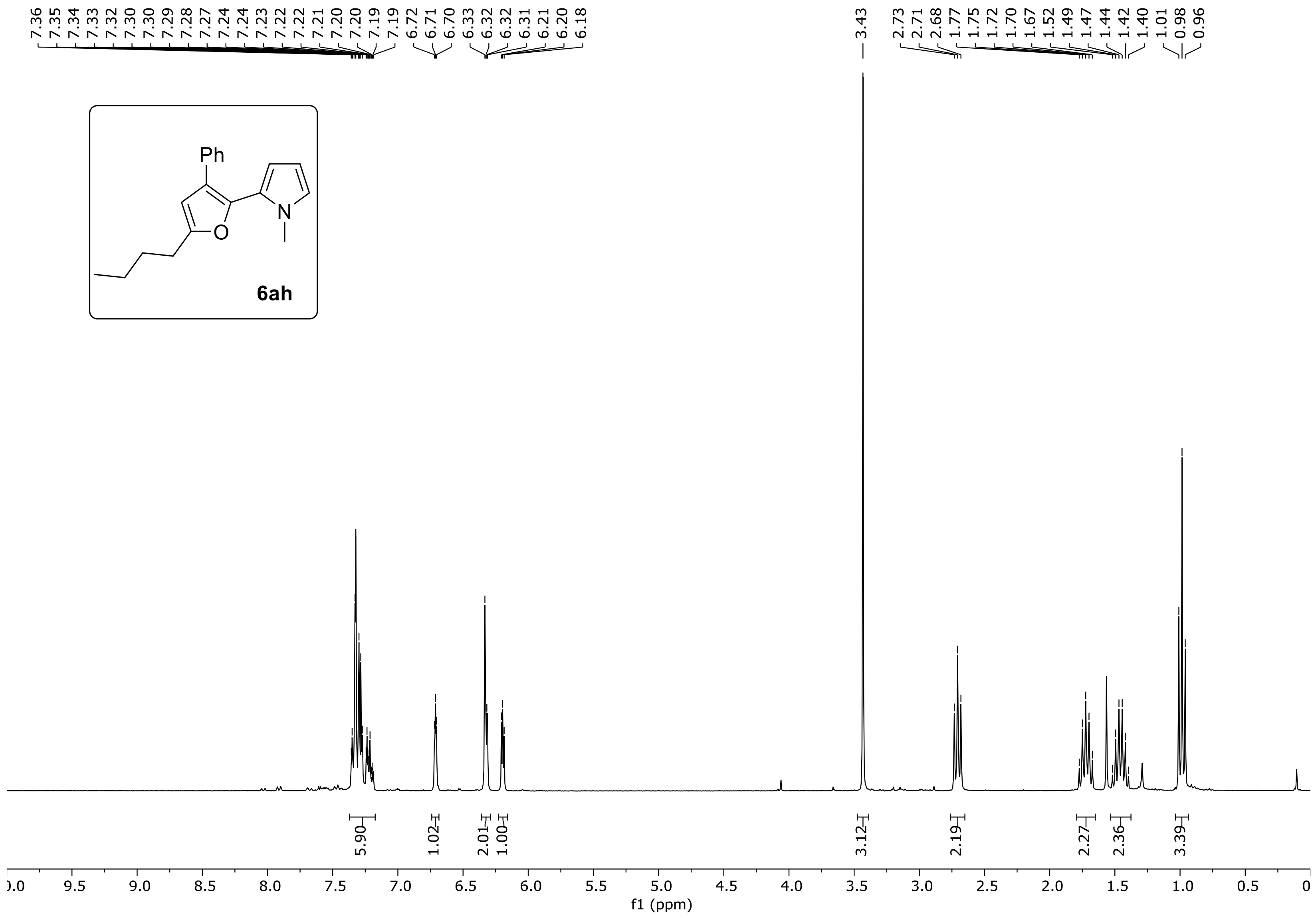

Figure S138: ^{13}C NMR of compound **6ah** in $CDCl_3$ at 75.4 MHz.

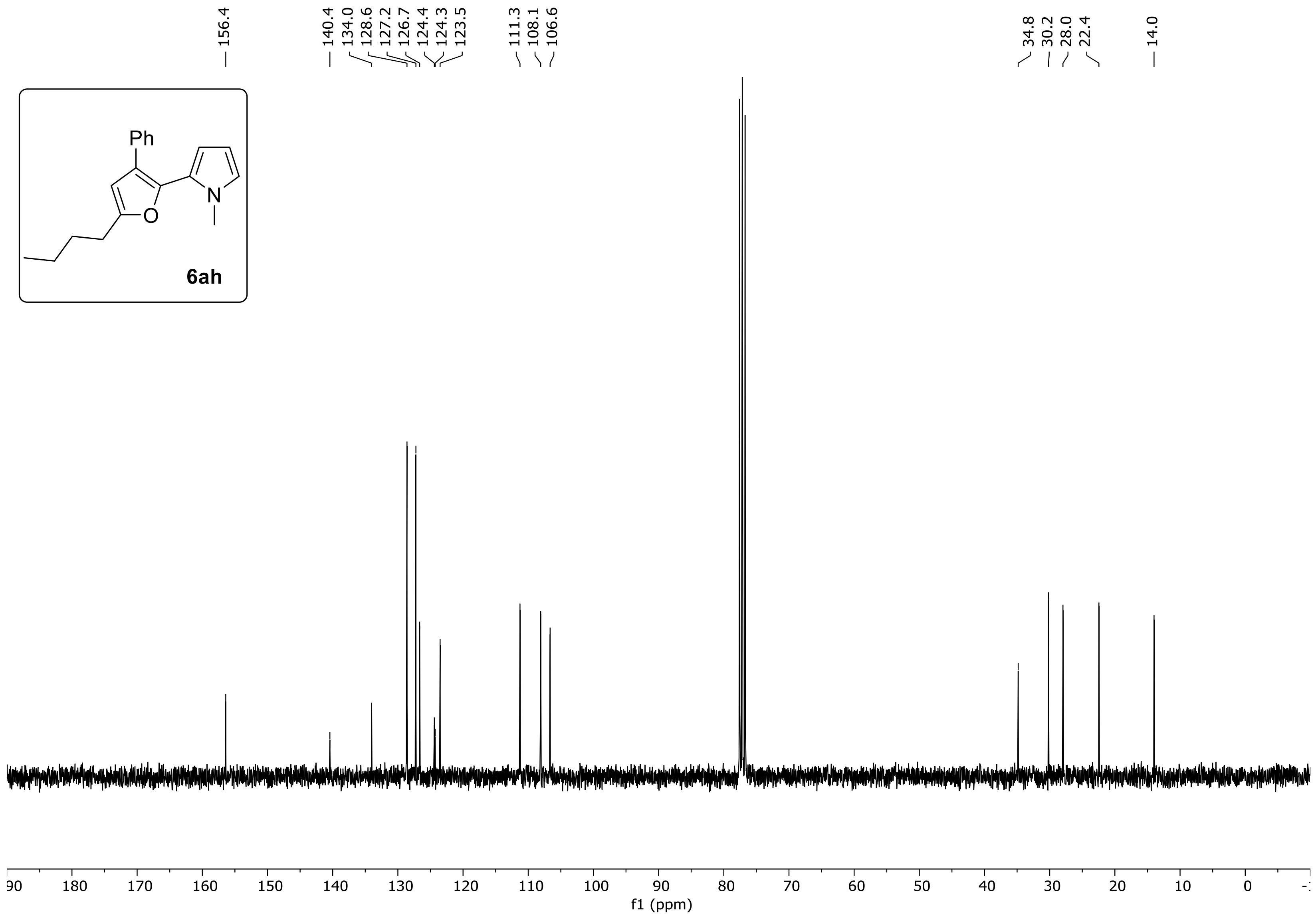

Figure S139: ^{3}H NMR of compound **7aa** in $CDCl_3$ at 300 MHz.

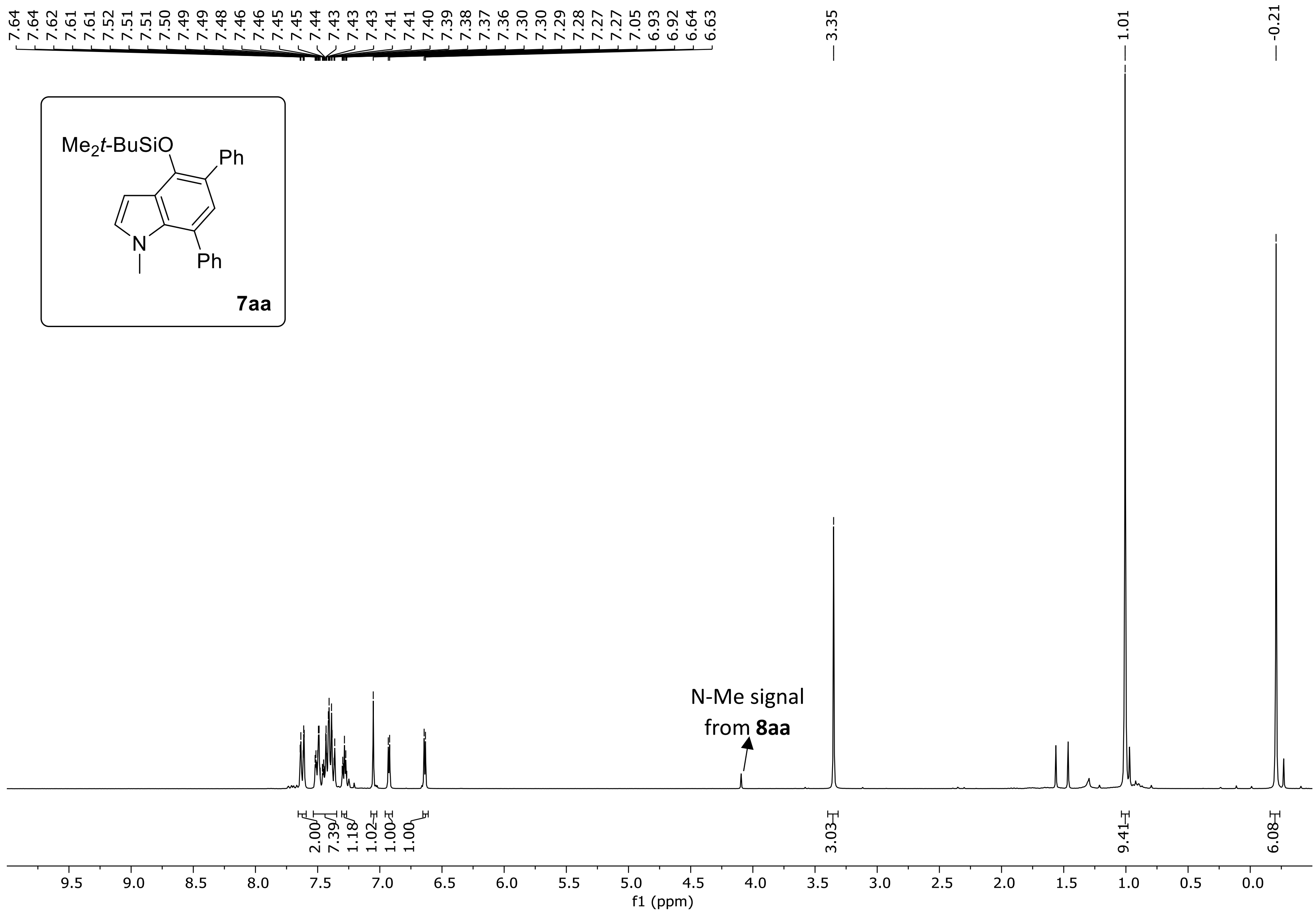

Figure S140: ^{13}C NMR of compound **7aa** in $CDCl_3$ at 75.4 MHz.

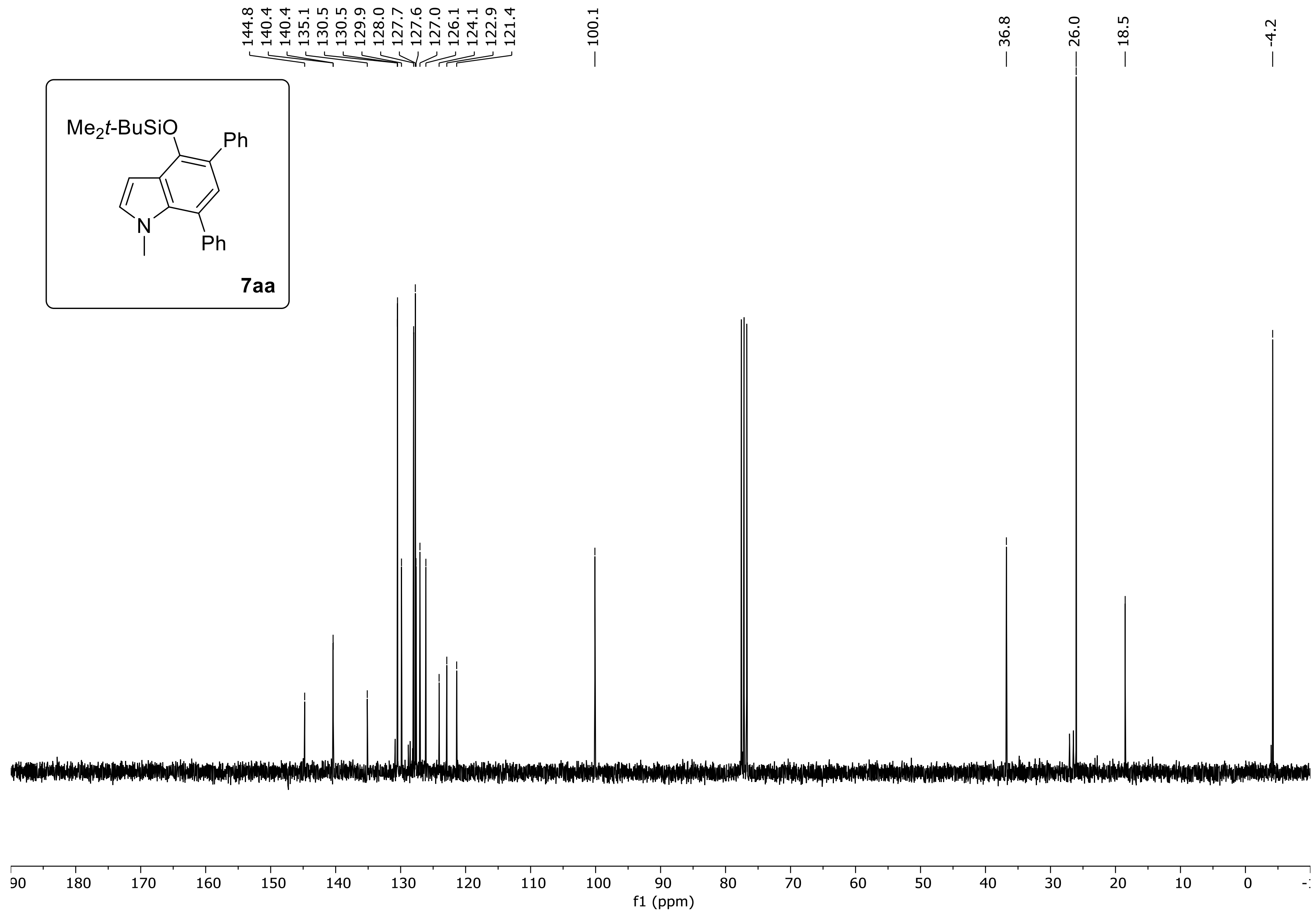

Figure S141: 1D NOE NMR of compound **7aa** in $CDCl_3$ at 300 MHz.

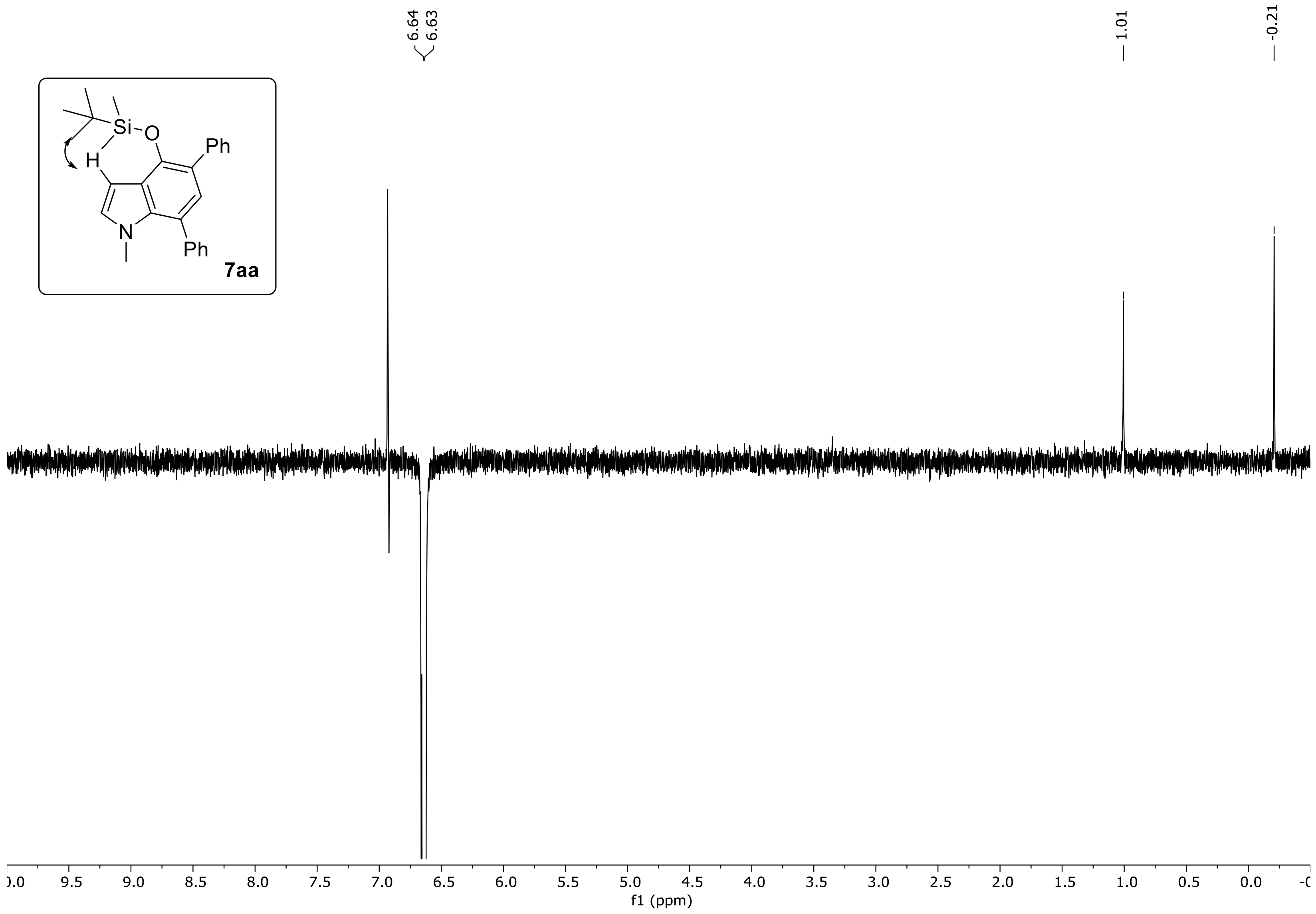

Figure S142: ^{1}H NMR of compound **7´aa** in $CDCl_3$ at 300 MHz.

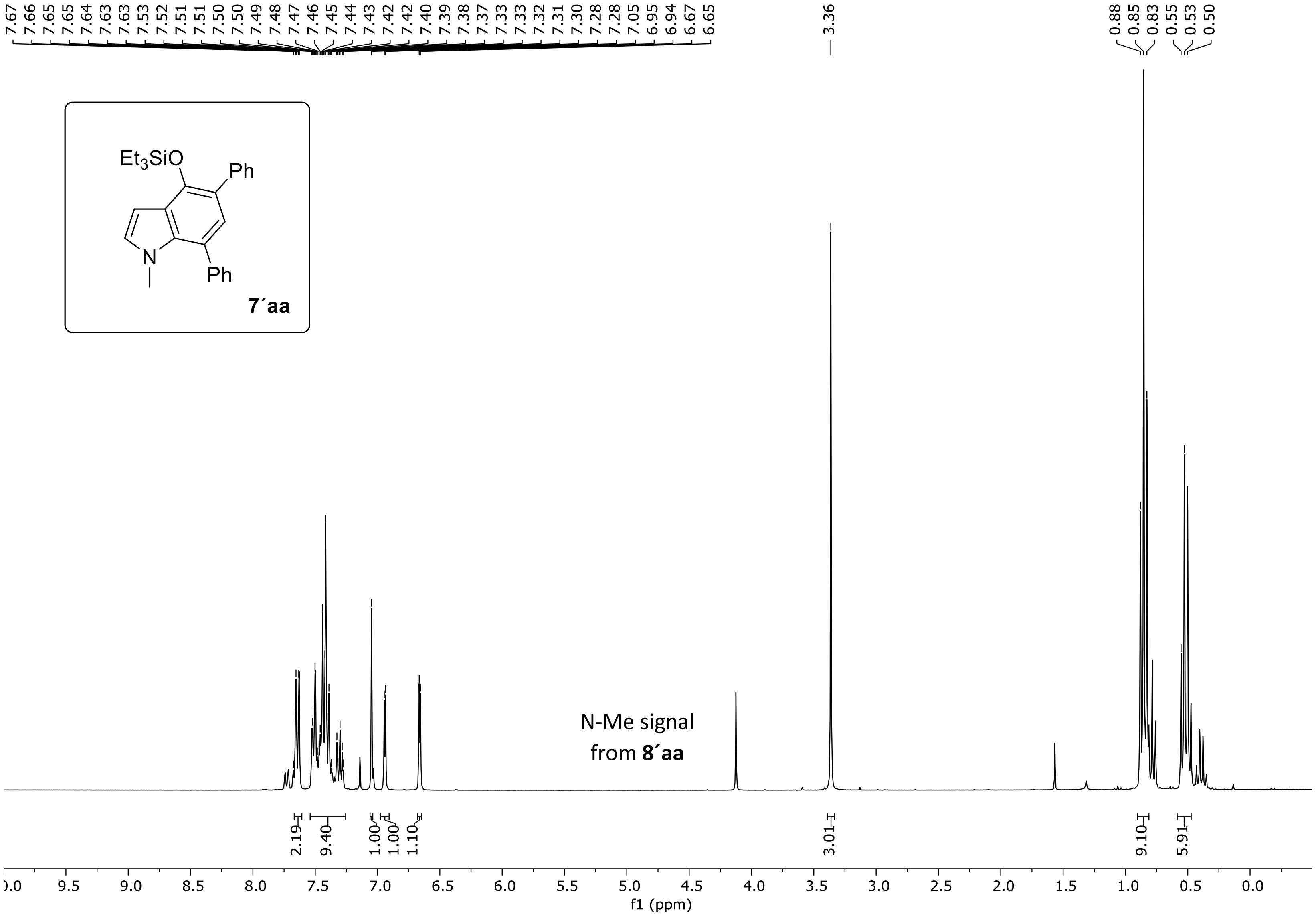

Figure S143: ^{13}C NMR of compound **7´aa** in $CDCl_3$ at 75.4 MHz.

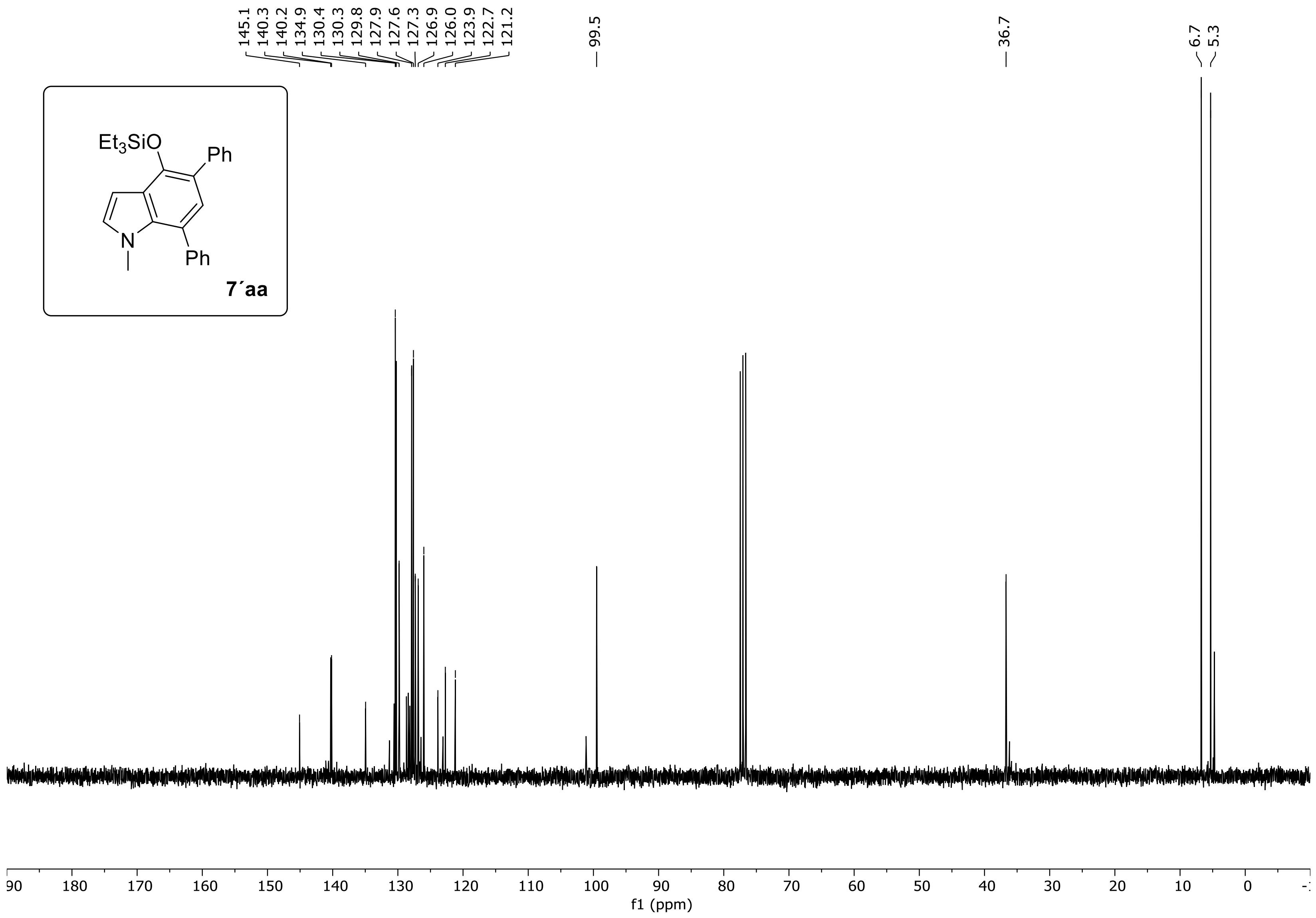

Figure S144: ^{1}H NMR of compound **7´´aa/8´´aa** in $CDCl_3$ at 300 MHz.

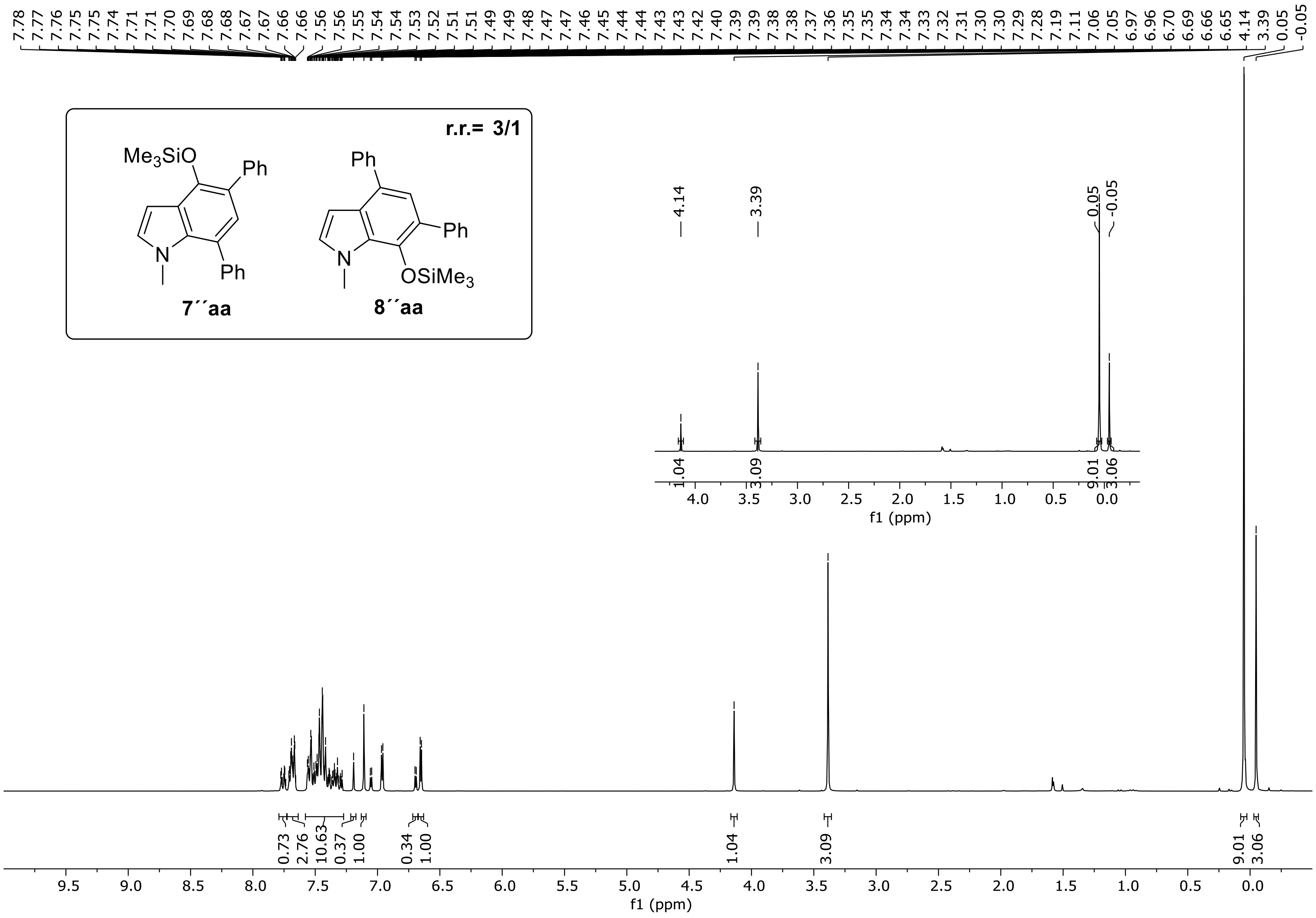

Figure S145: ^{13}C NMR of compound **7´´aa**/**8´´aa** in $CDCl_3$ at 75.4 MHz.

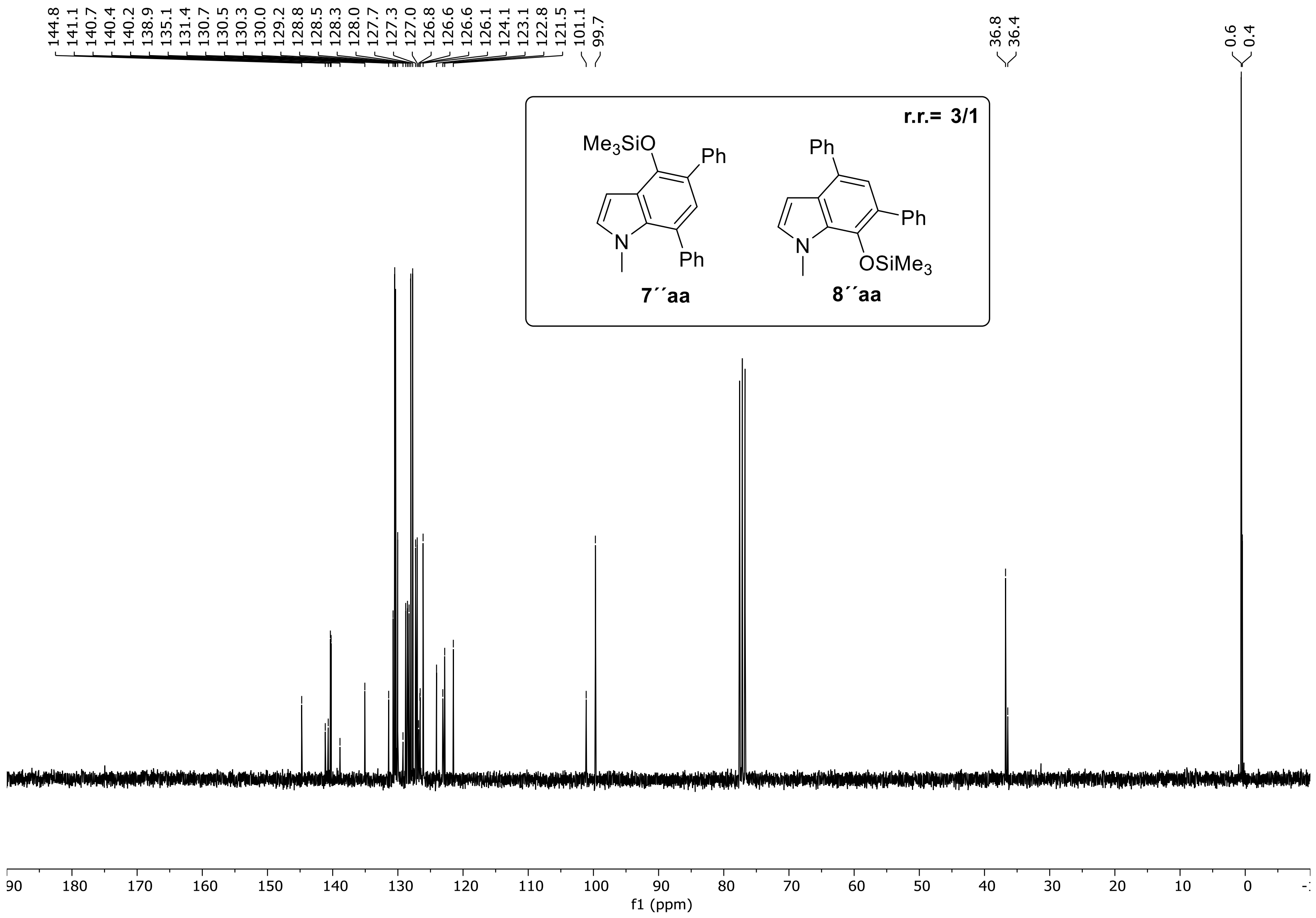

Figure S146: 1D NOE NMR of compound **7´´aa** in $CDCl_3$ at 300 MHz.

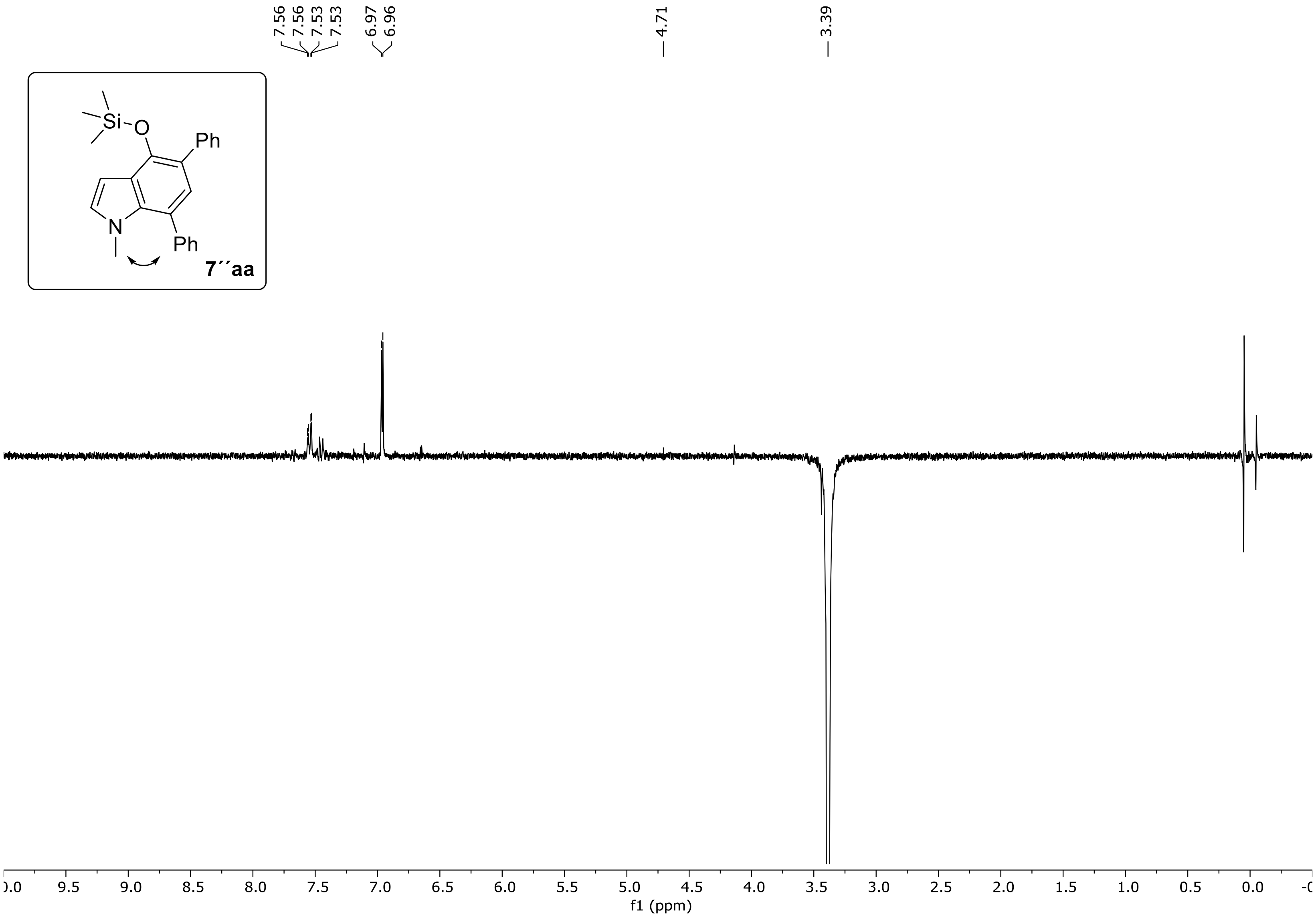

Figure S147: 1D NOE NMR of compound **8´´aa** in $CDCl_3$ at 300 MHz.

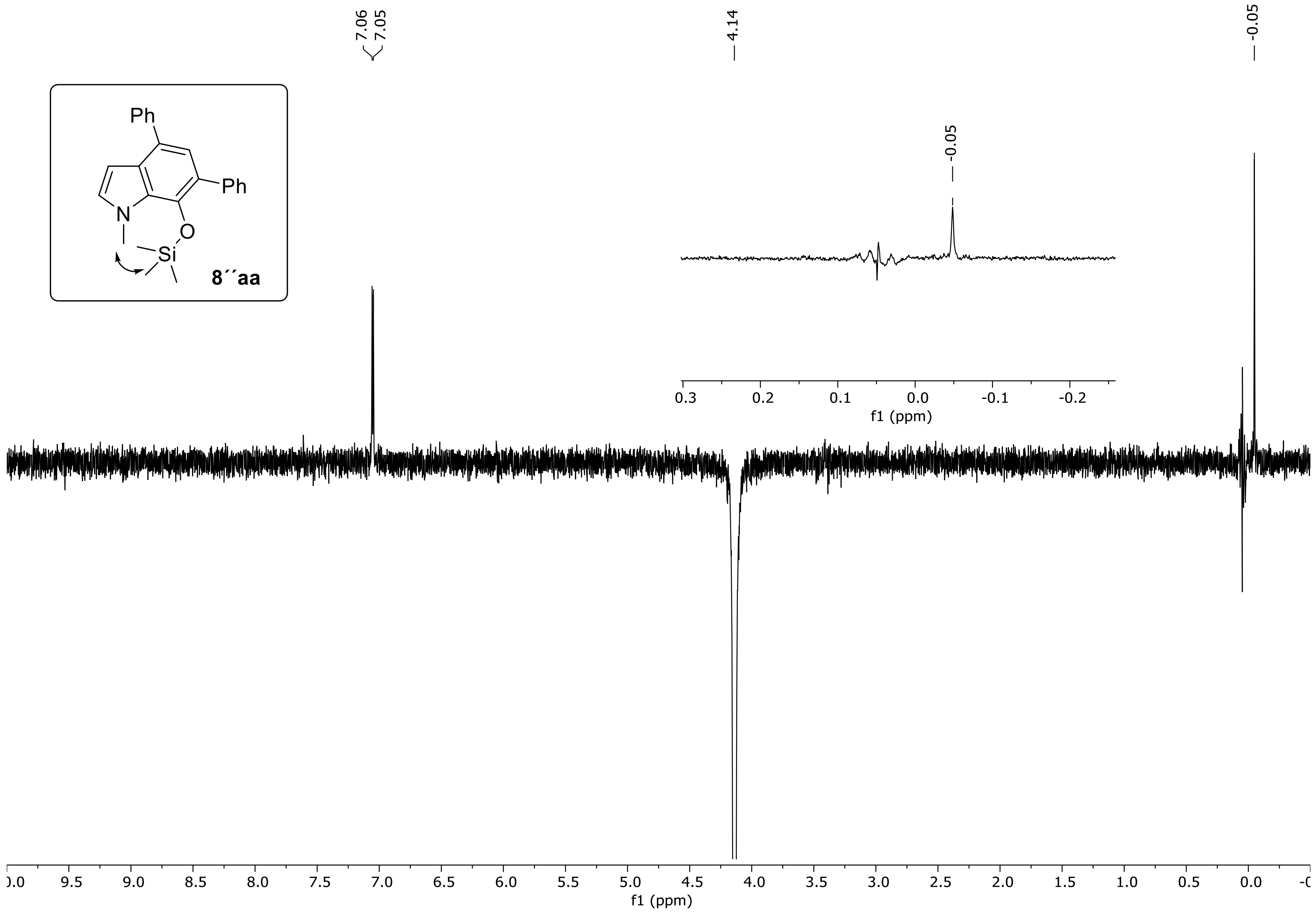

Figure S148: ^{1}H NMR of compound **7ab** in $CDCl_3$ at 300 MHz.

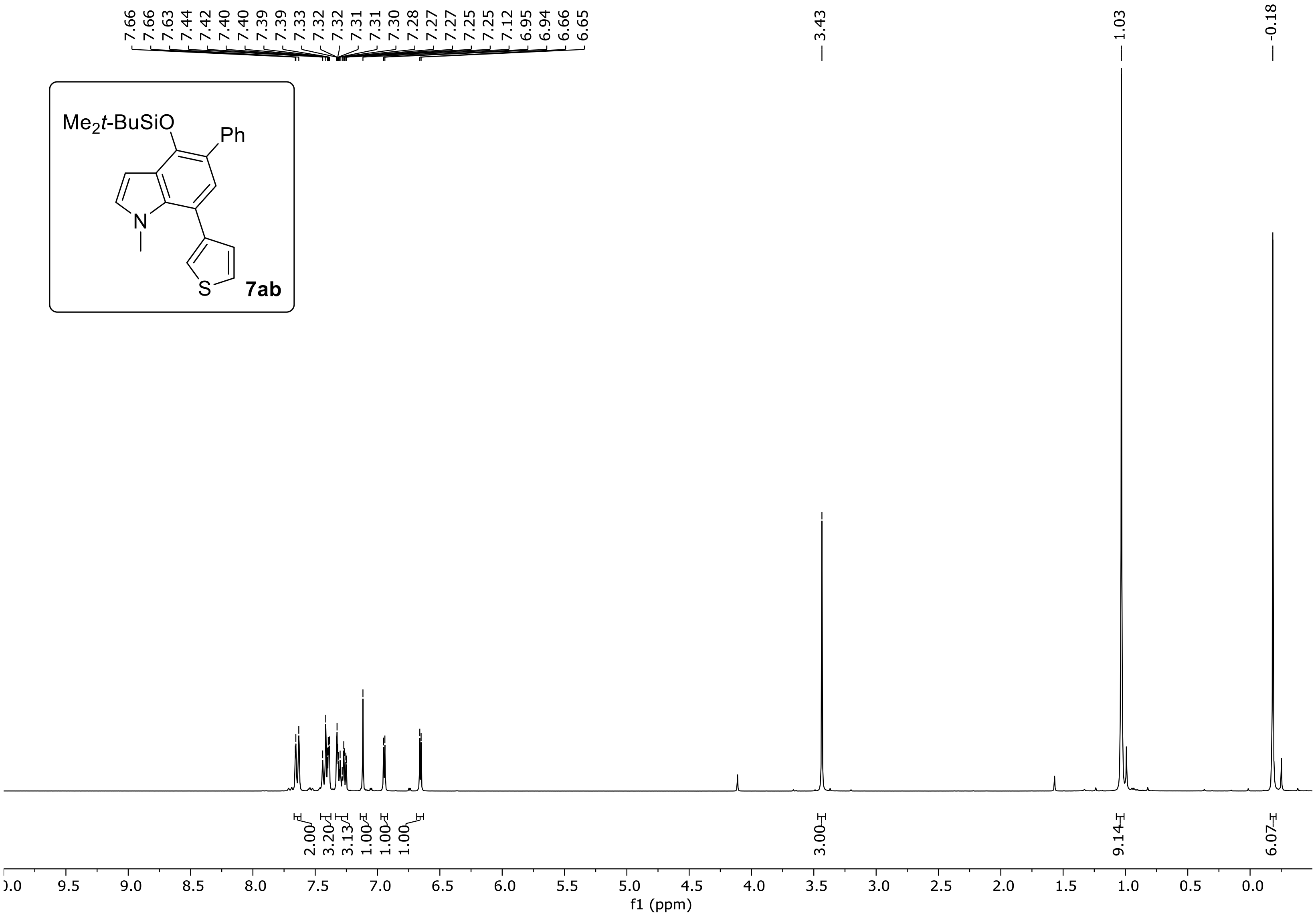

Figure S149: ^{13}C NMR of compound **7ab** in $CDCl_3$ at 75.4 MHz.

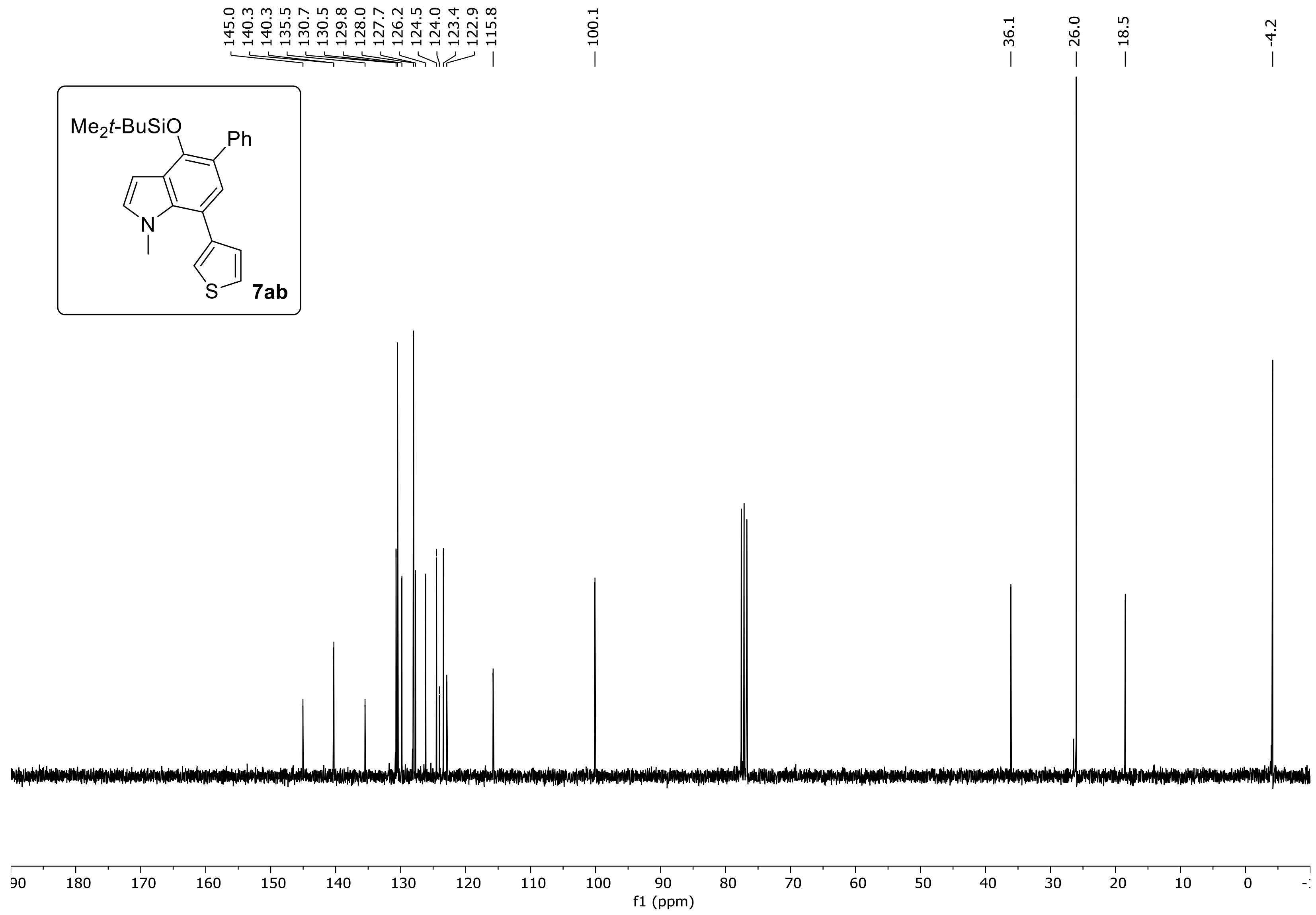

Figure S150: 1D NOE NMR of compound **7ab** in $CDCl_3$ at 300 MHz.

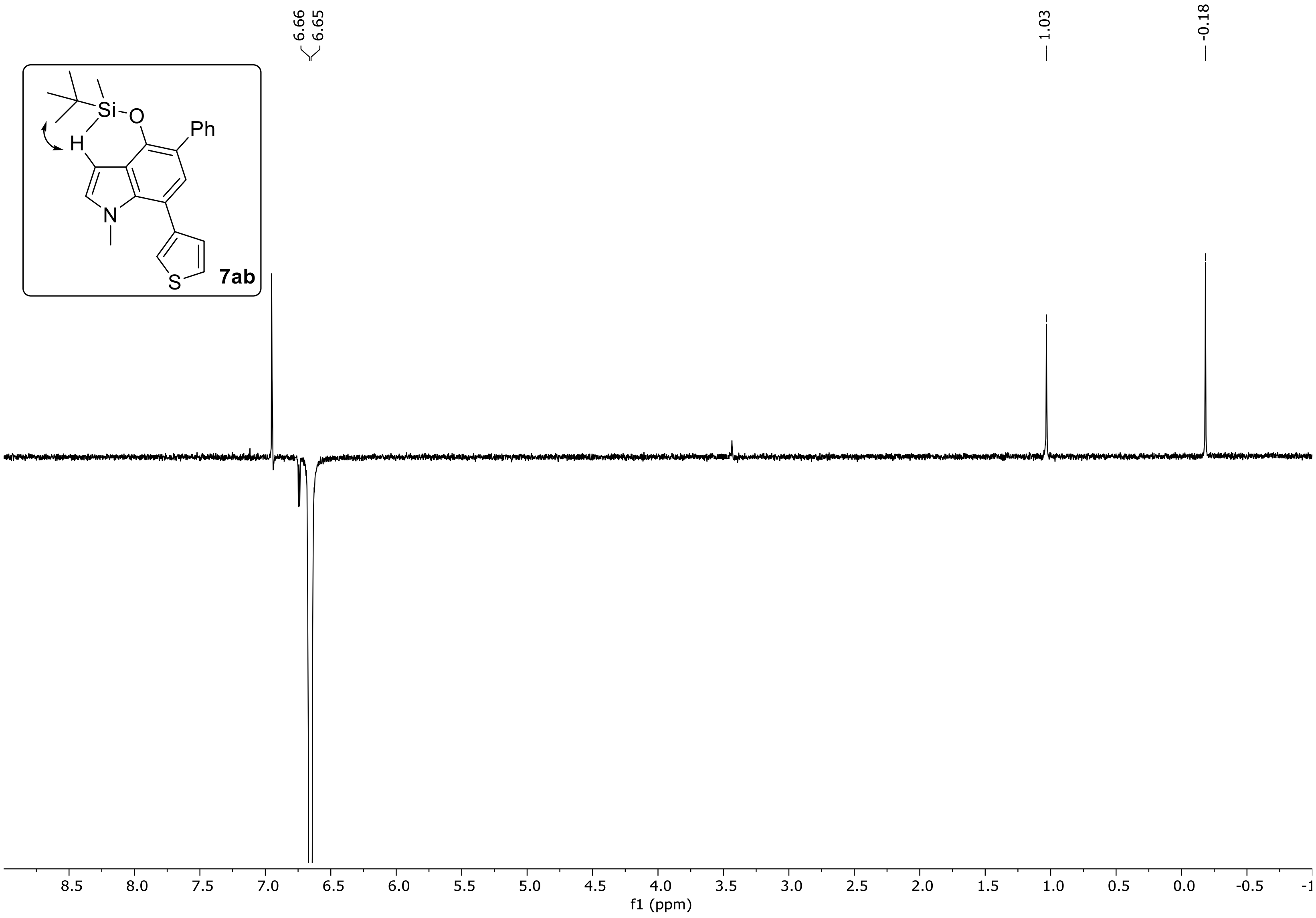

Figure S151: ^{1}H NMR of compound **7ac** in $CDCl_3$ at 300 MHz.

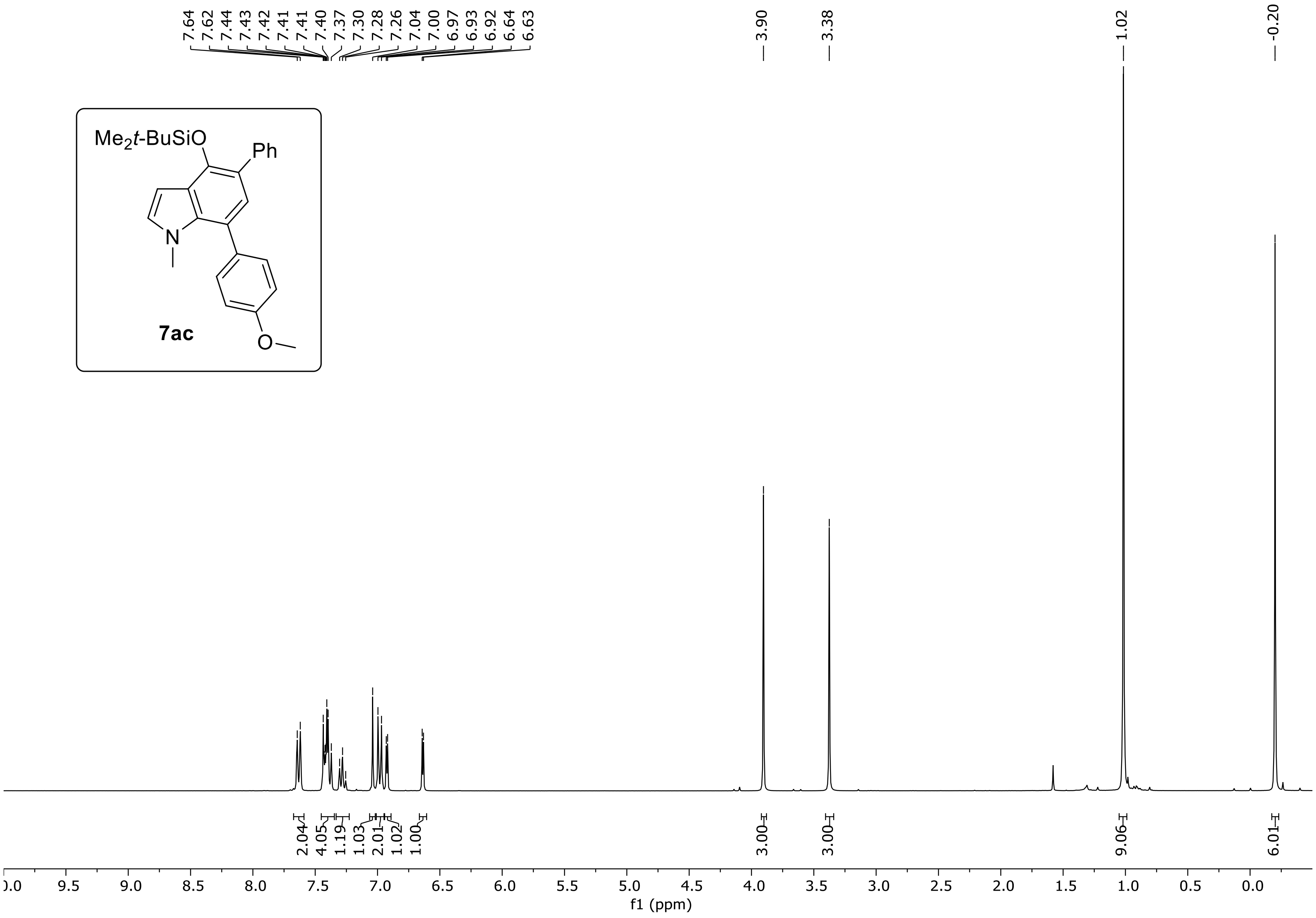

Figure S152: ^{13}C NMR of compound **7ac** in $CDCl_3$ at 75.4 MHz.

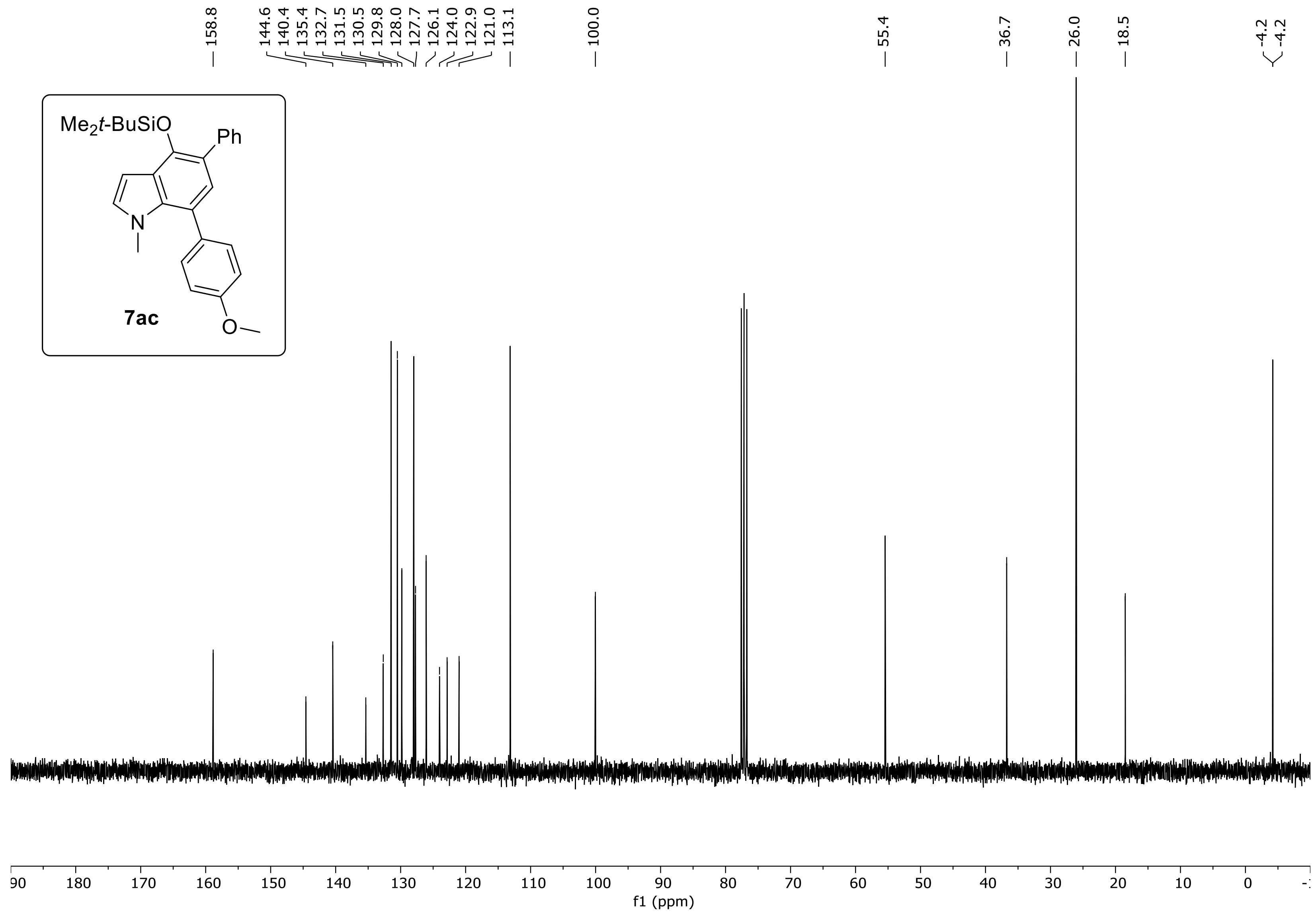

Figure S153: 1D NOE NMR of compound **7ac** in $CDCl_3$ at 300 MHz.

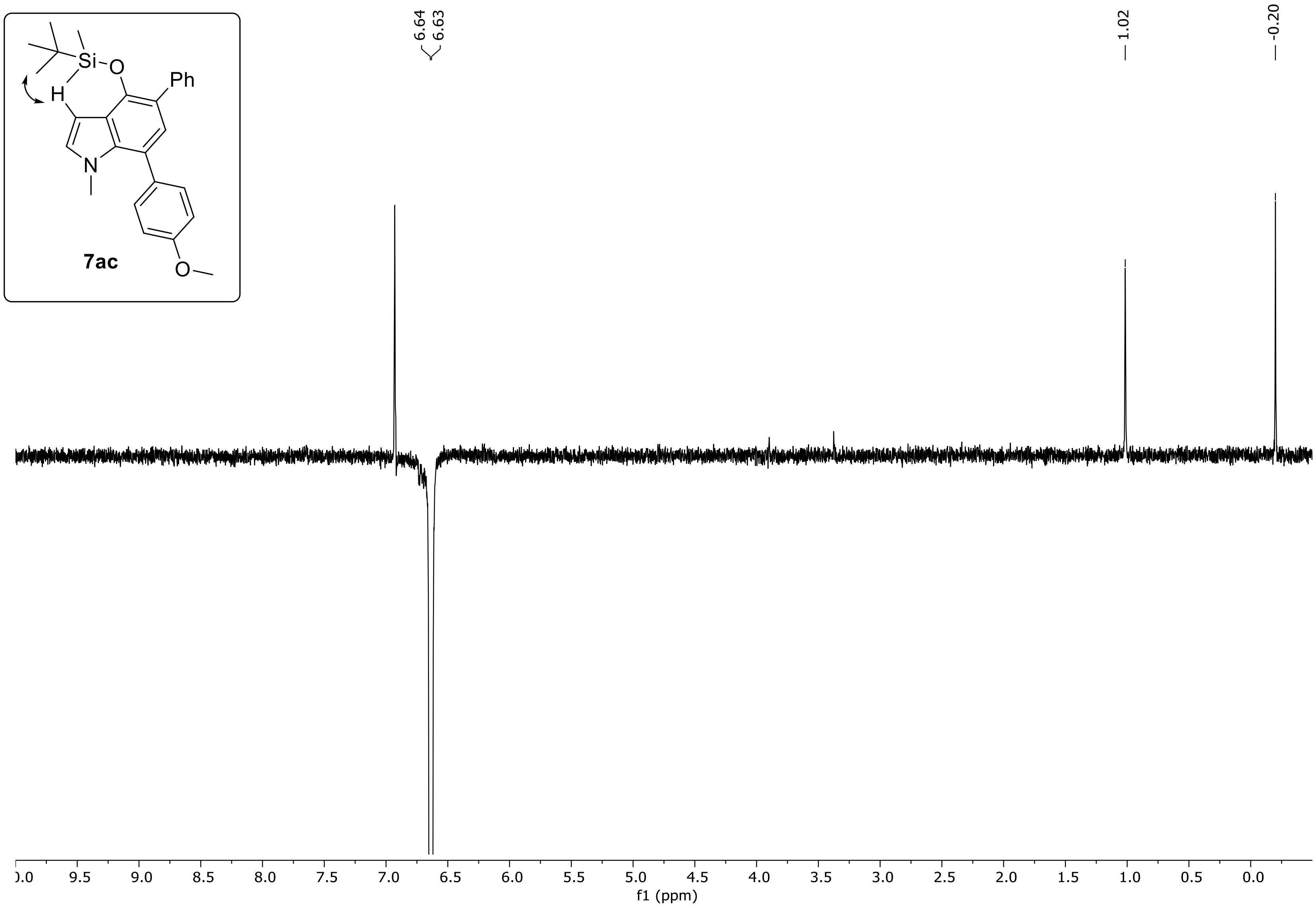

Figure S154: ^{1}H NMR of compound **7ad** in $CDCl_3$ at 300 MHz.

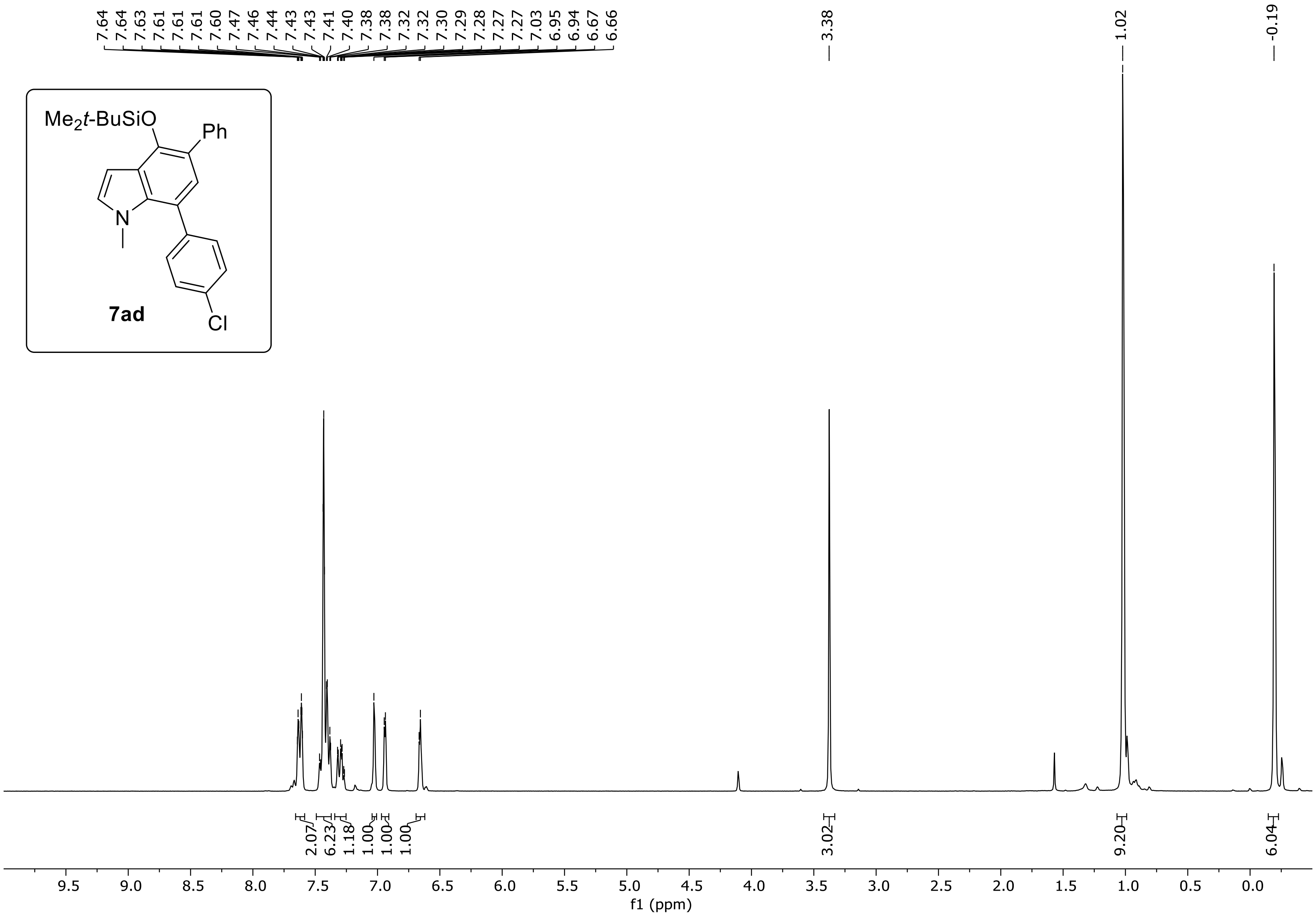

Figure S155: ^{13}C NMR of compound **7ad** in $CDCl_3$ at 75.4 MHz.

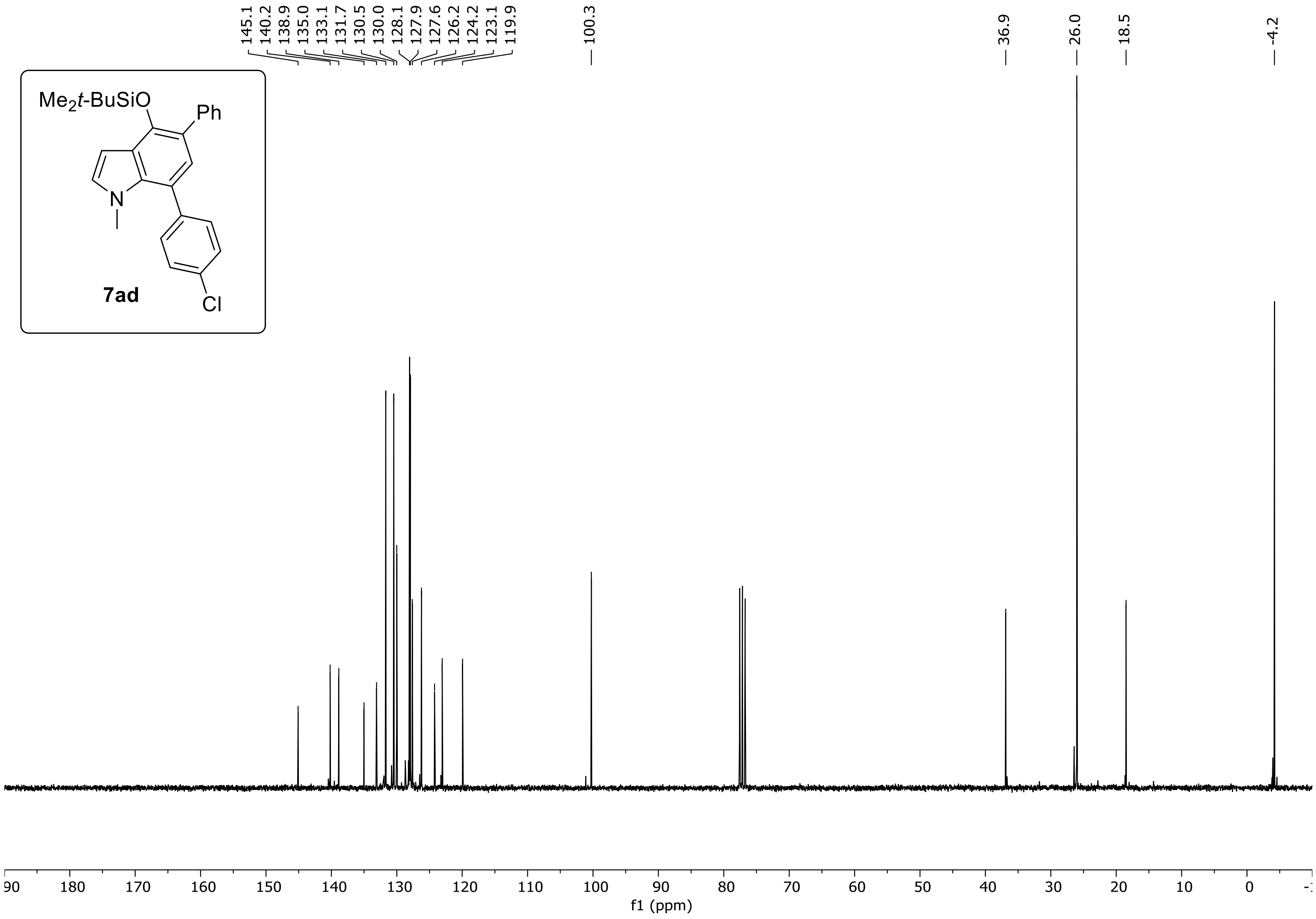

Figure S156: ^{1}H NMR of compound **7ae** in $CDCl_3$ at 300 MHz.

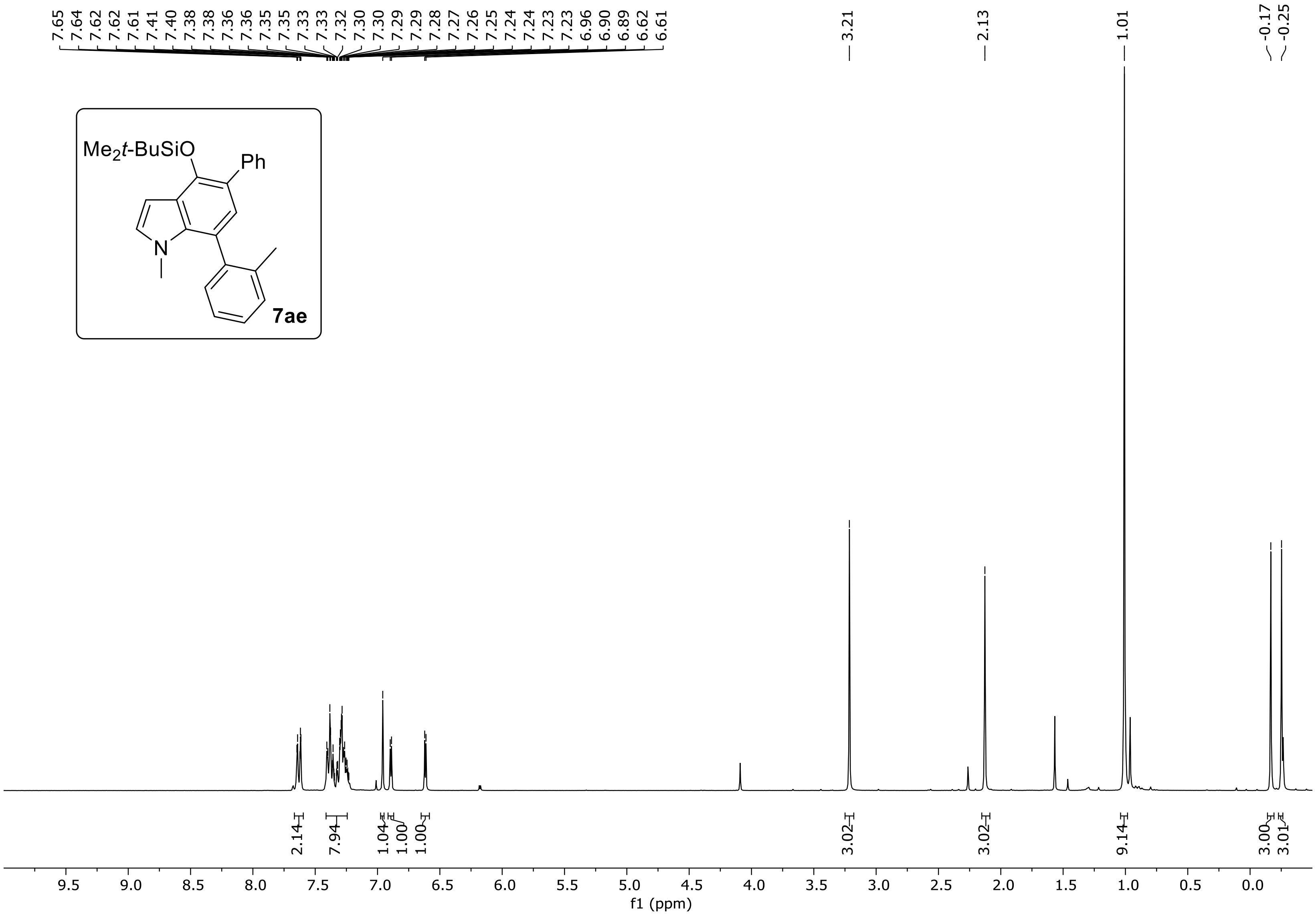

Figure S157: ^{13}C NMR of compound **7ae** in $CDCl_3$ at 75.4 MHz.

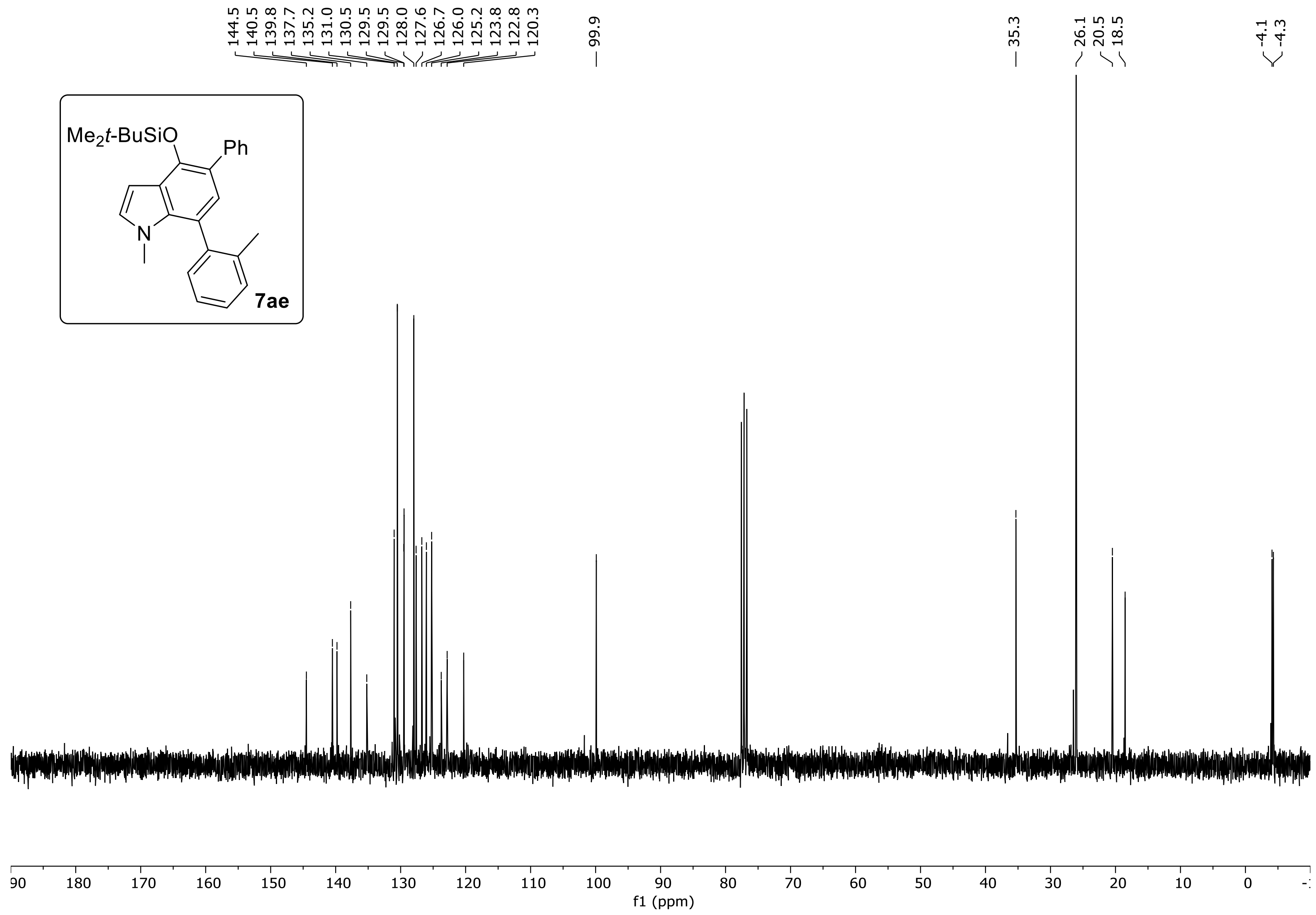

Figure S158: 1D NOE NMR of compound **7ae** in $CDCl_3$ at 300 MHz.

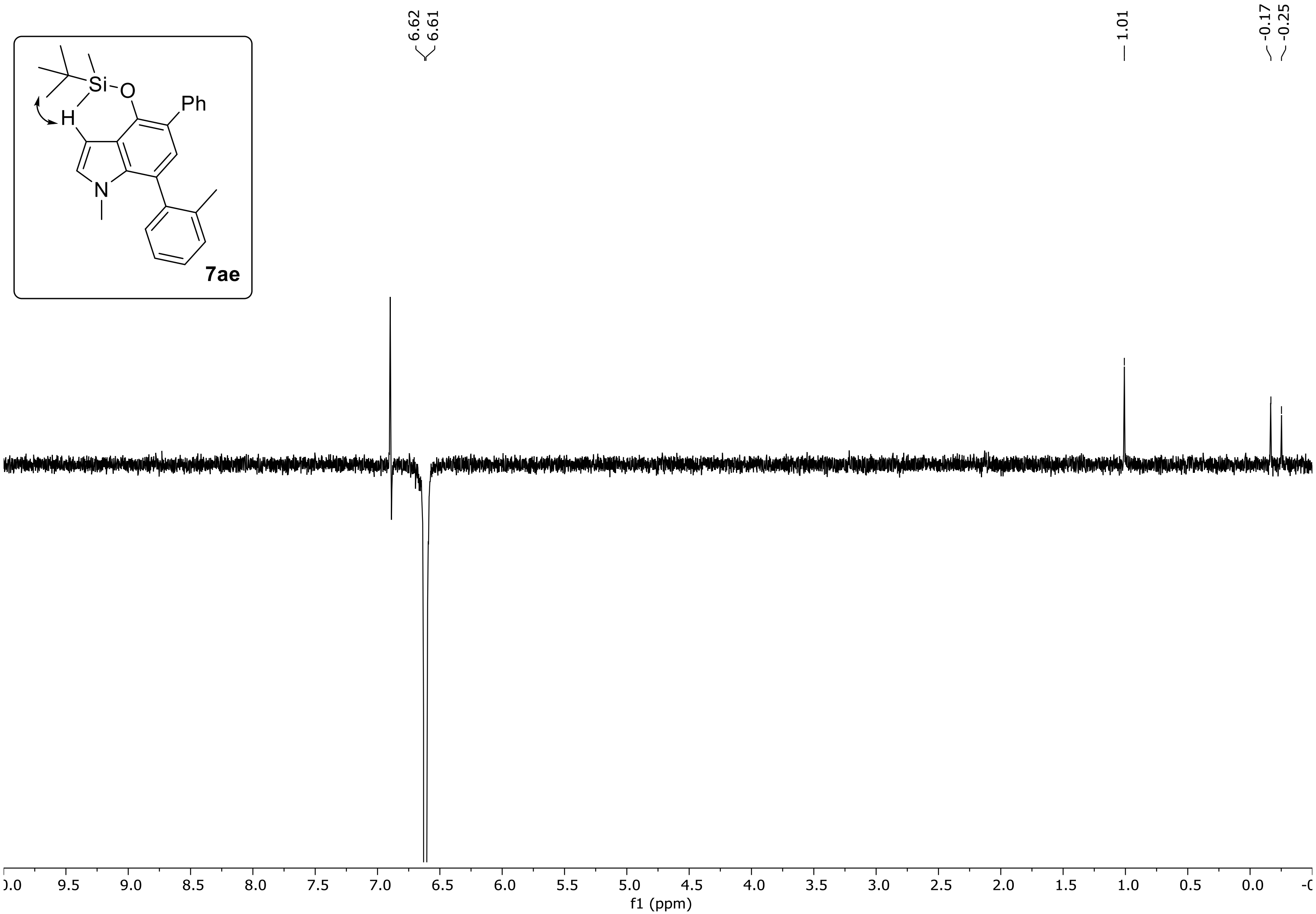

Figure S159: ^{1}H NMR of compound **7af** in $CDCl_3$ at 300 MHz.

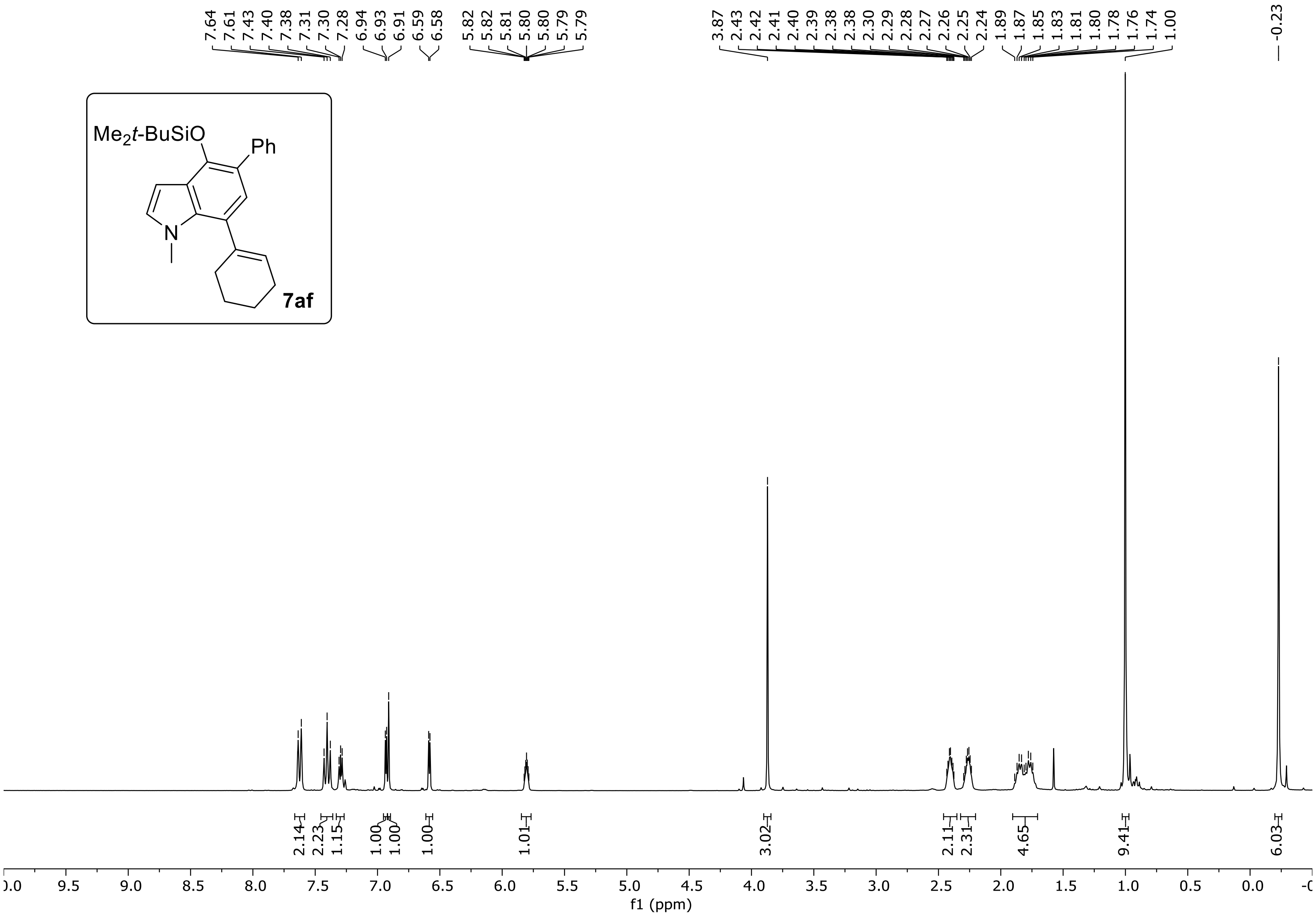

Figure S160: ^{13}C NMR of compound **7af** in $CDCl_3$ at 75.4 MHz.

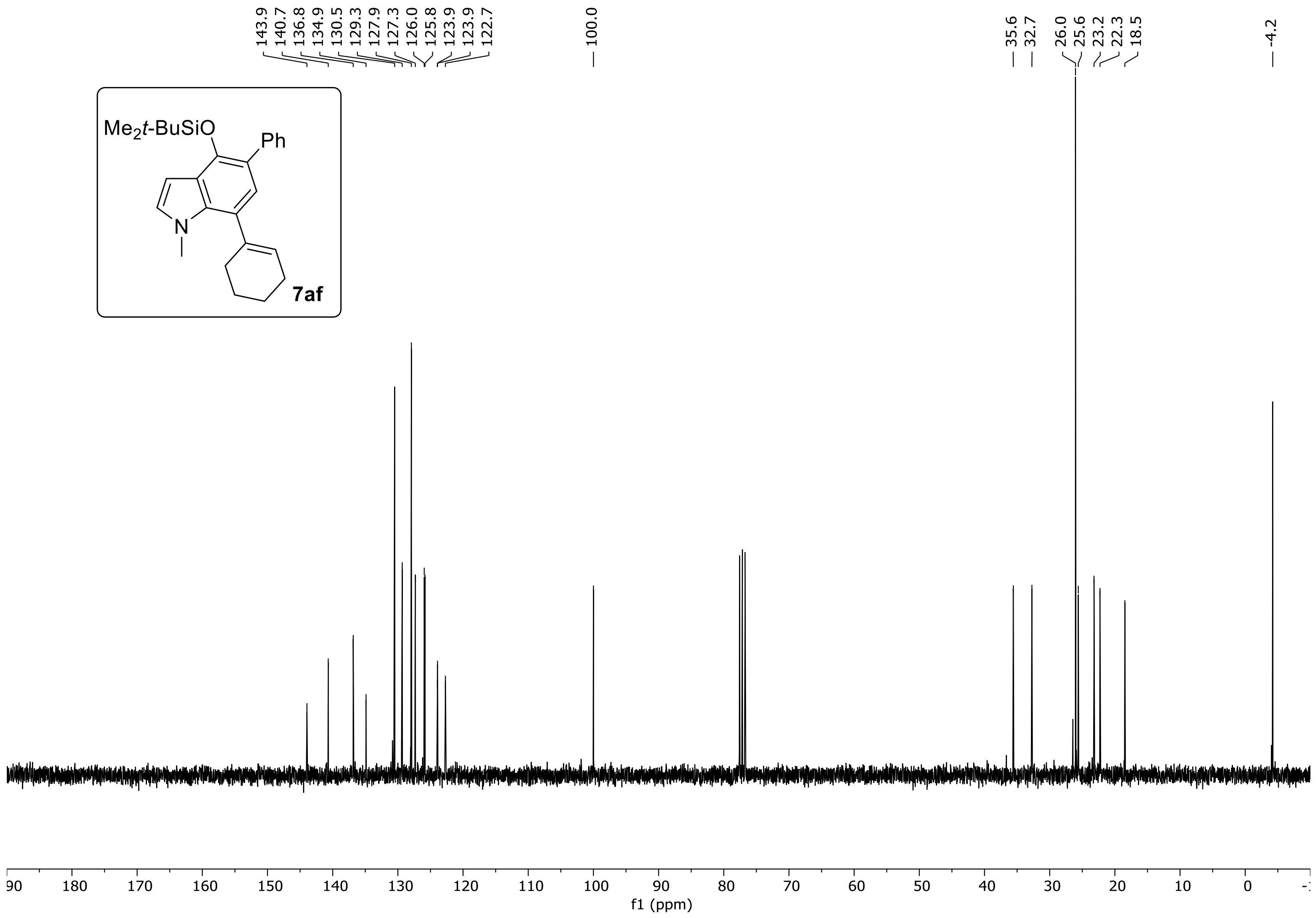

Figure S161: ^{1}H NMR of compound **7ag** in $CDCl_3$ at 300 MHz.

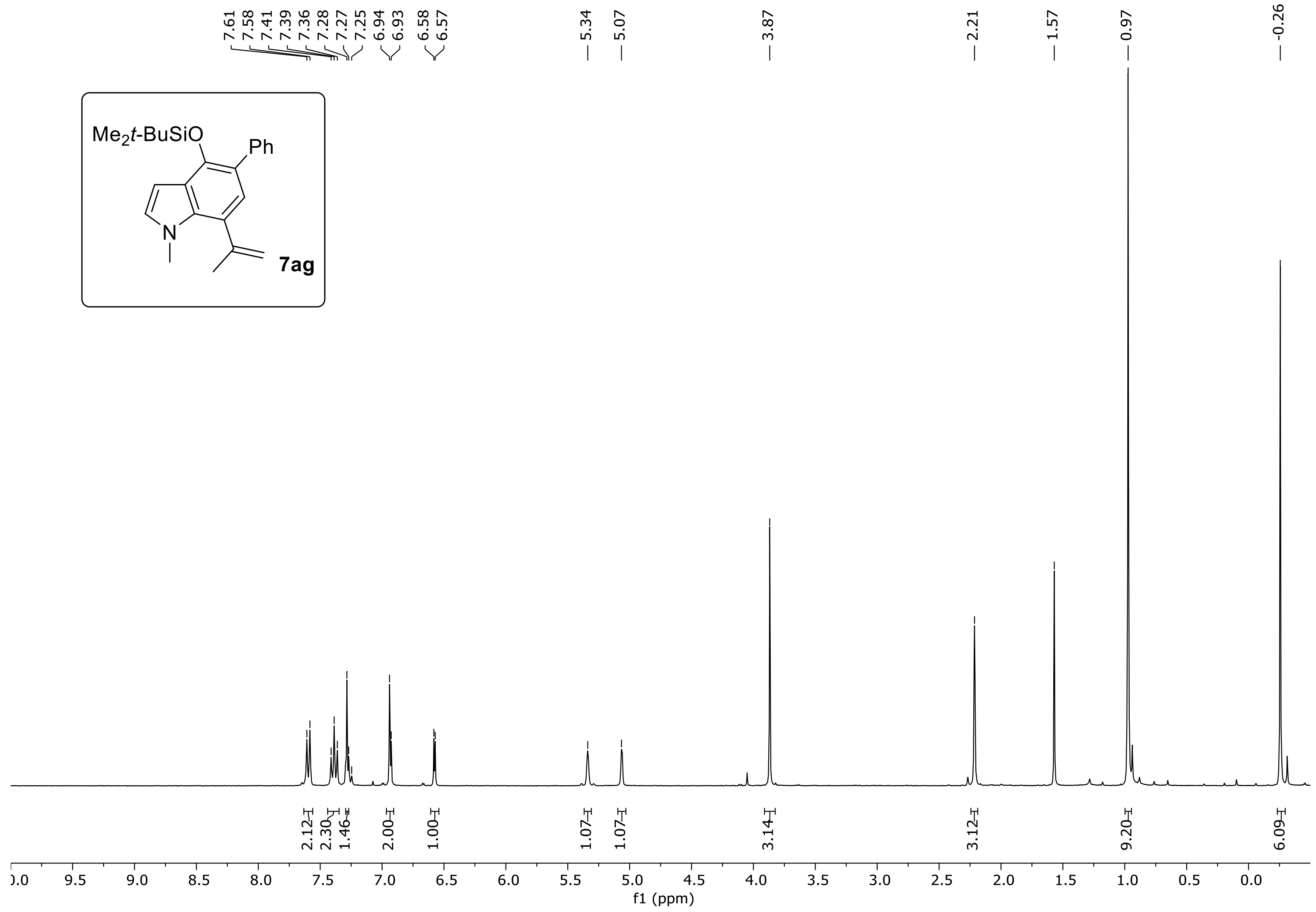

Figure S162: ^{13}C NMR of compound **7ag** in $CDCl_3$ at 75.4 MHz.

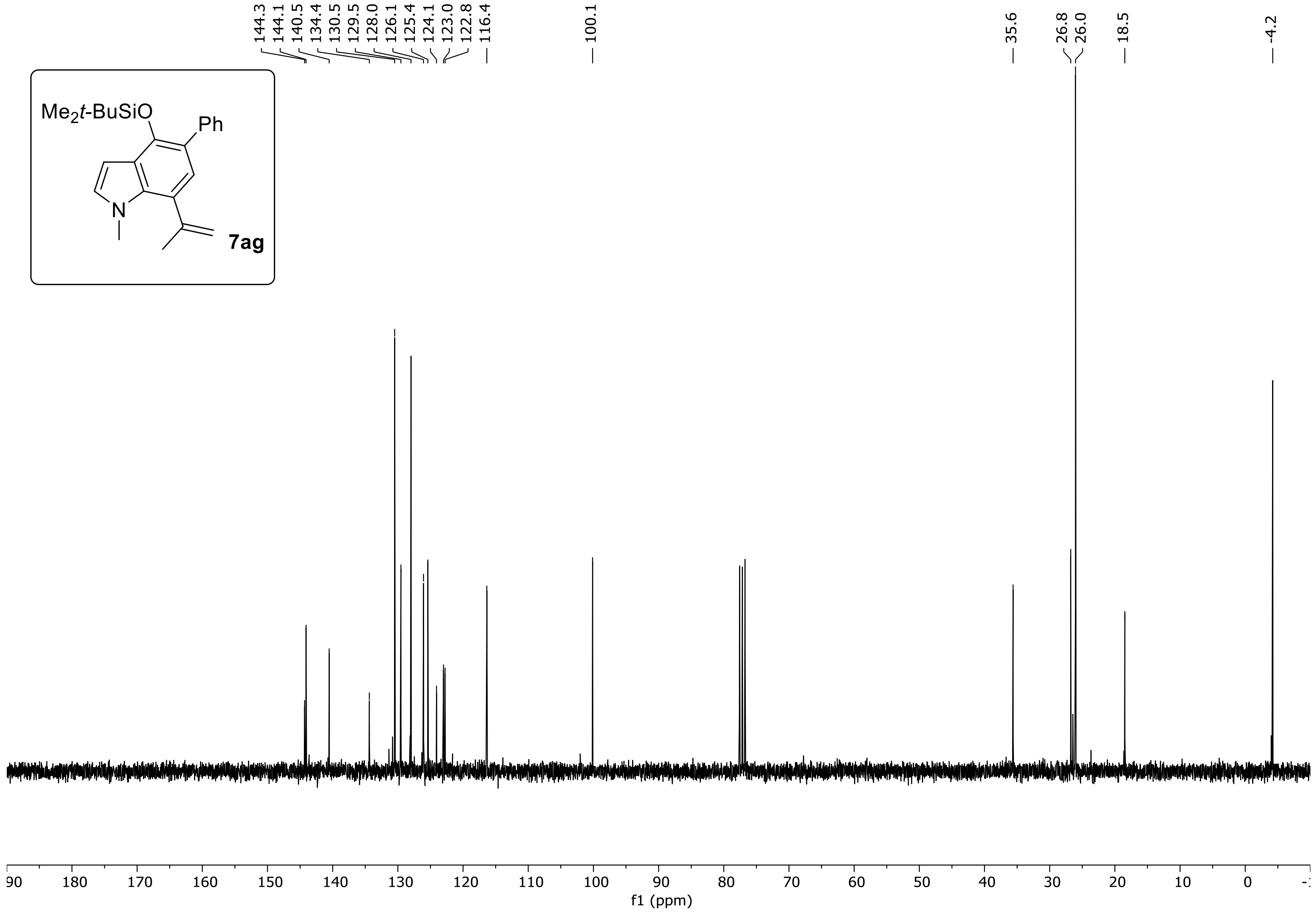

Figure S163: 1D NOE NMR of compound **7ag** in $CDCl_3$ at 300 MHz.

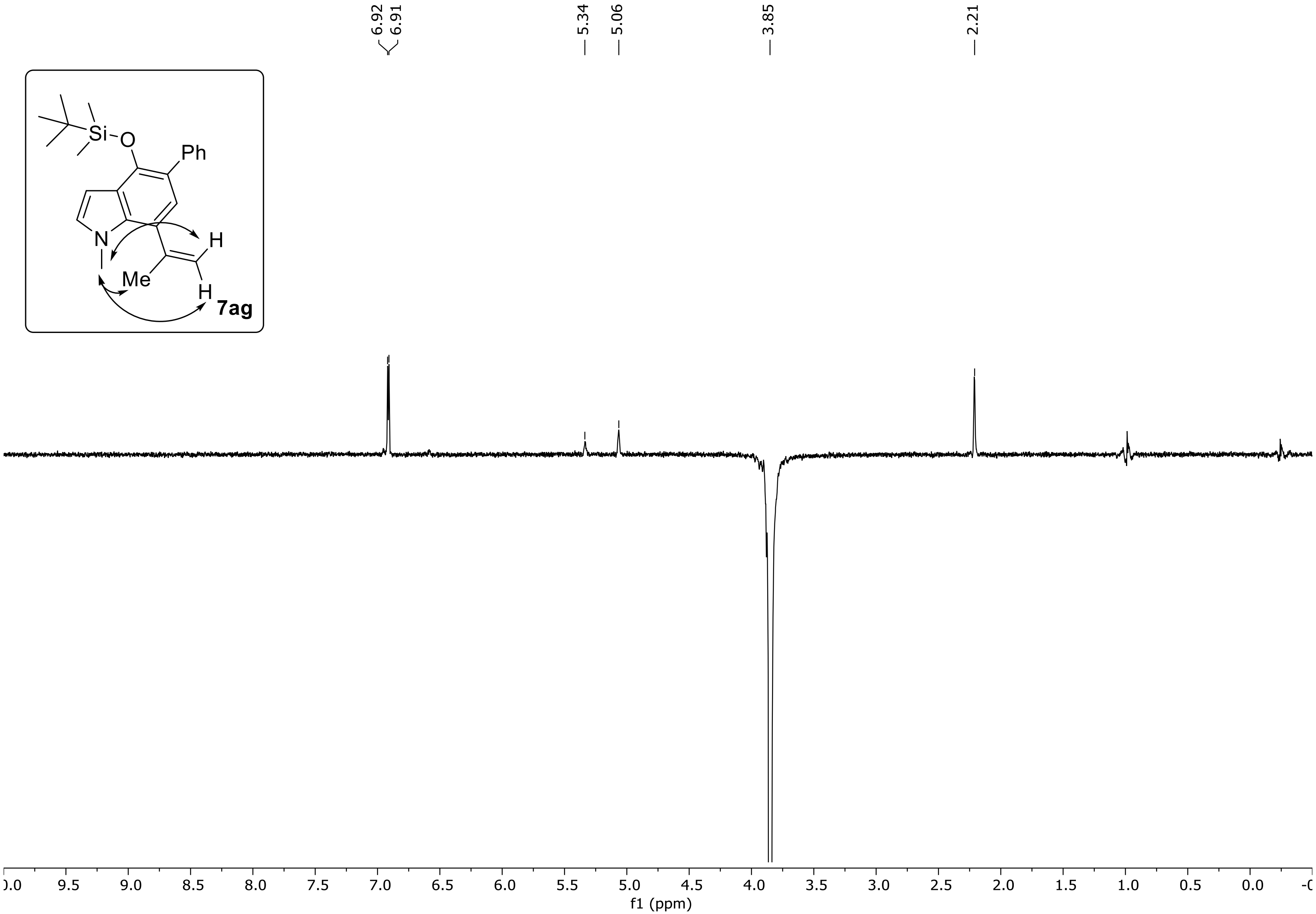

Figure S164: ^{1}H NMR of compound **7ah** in $CDCl_3$ at 300 MHz.

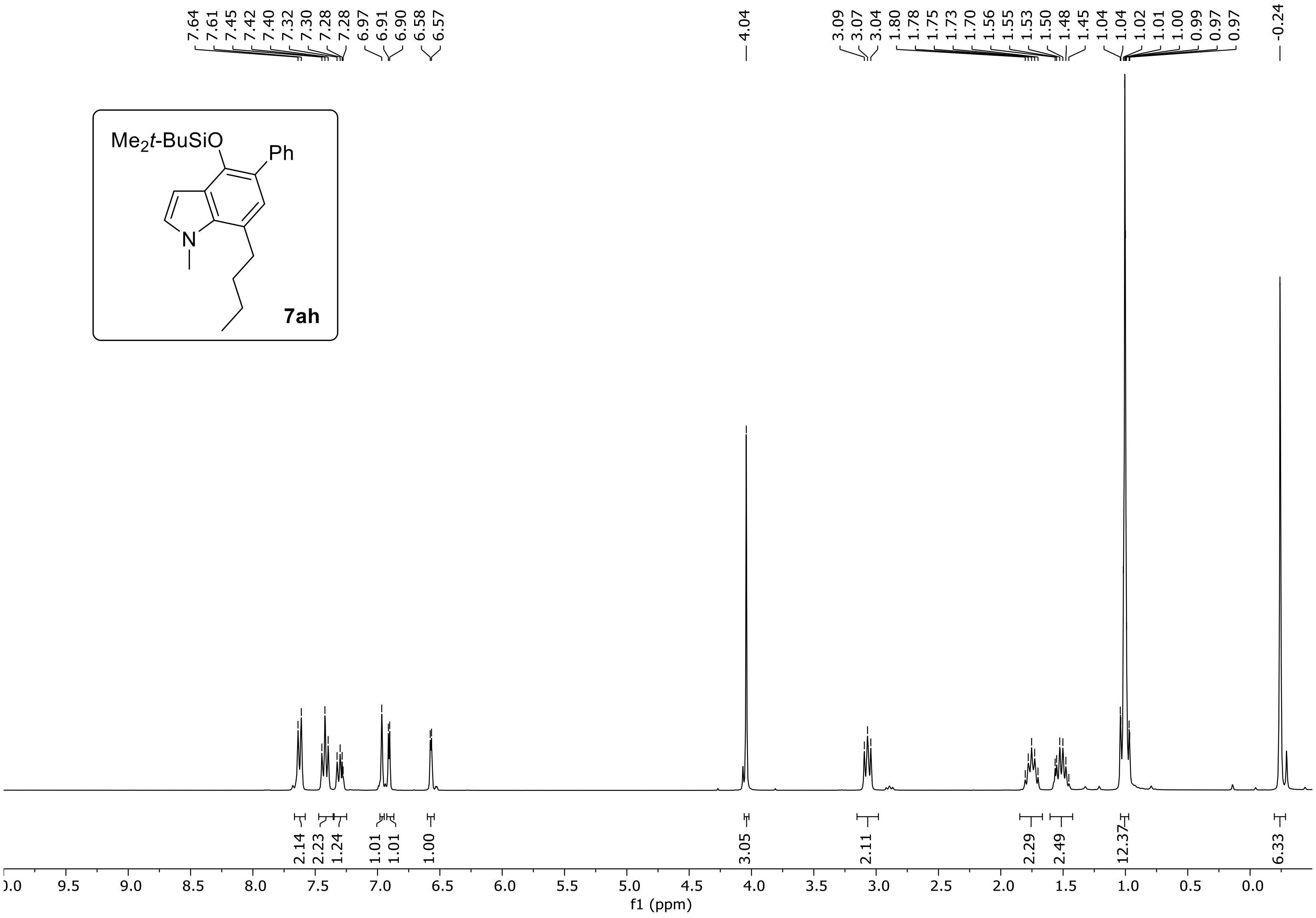

Figure S165: ^{13}C NMR of compound **7ah** in $CDCl_3$ at 75.4 MHz.

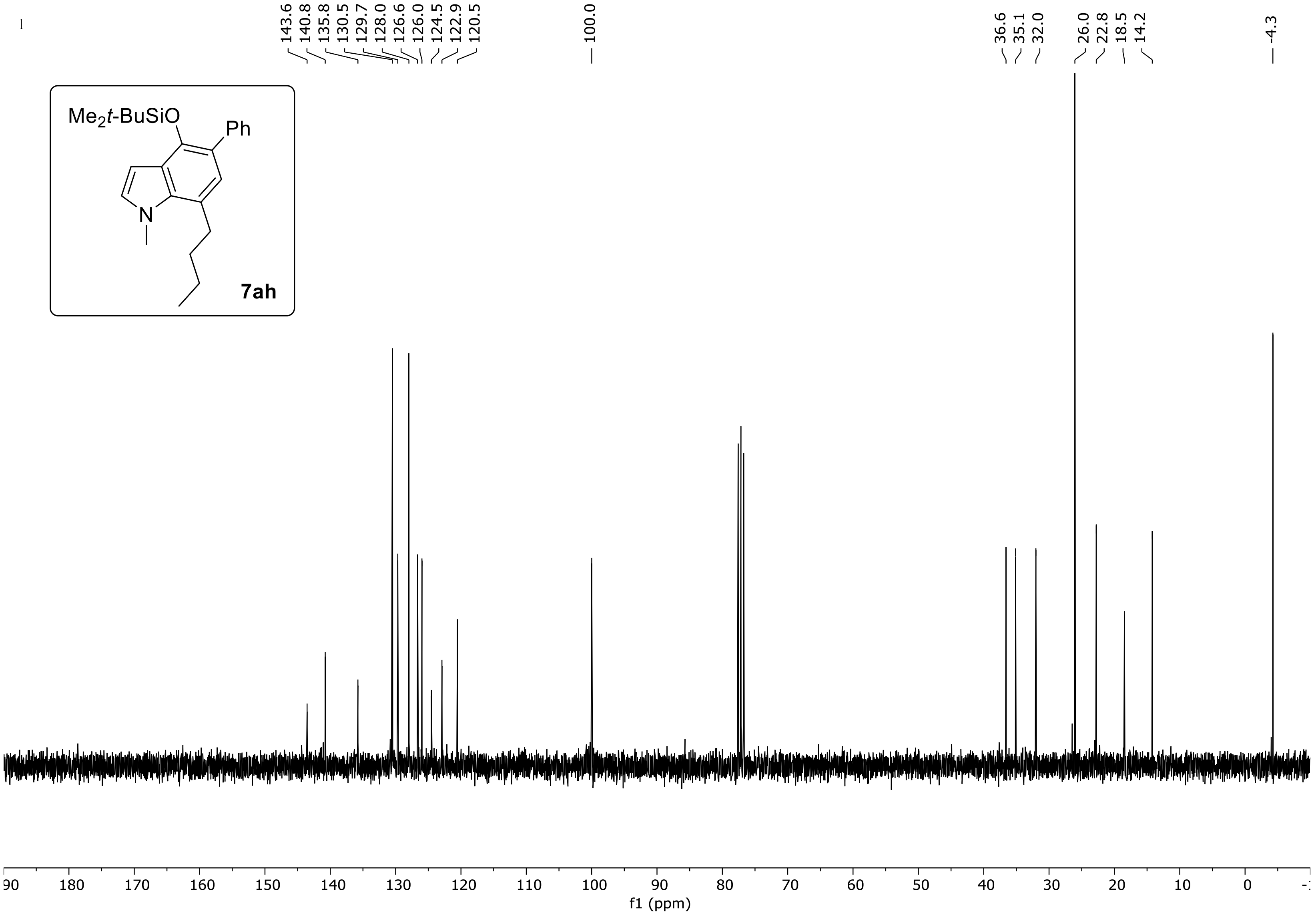

Figure S166: 1D NOE NMR of compound **7ah** in $CDCl_3$ at 300 MHz.

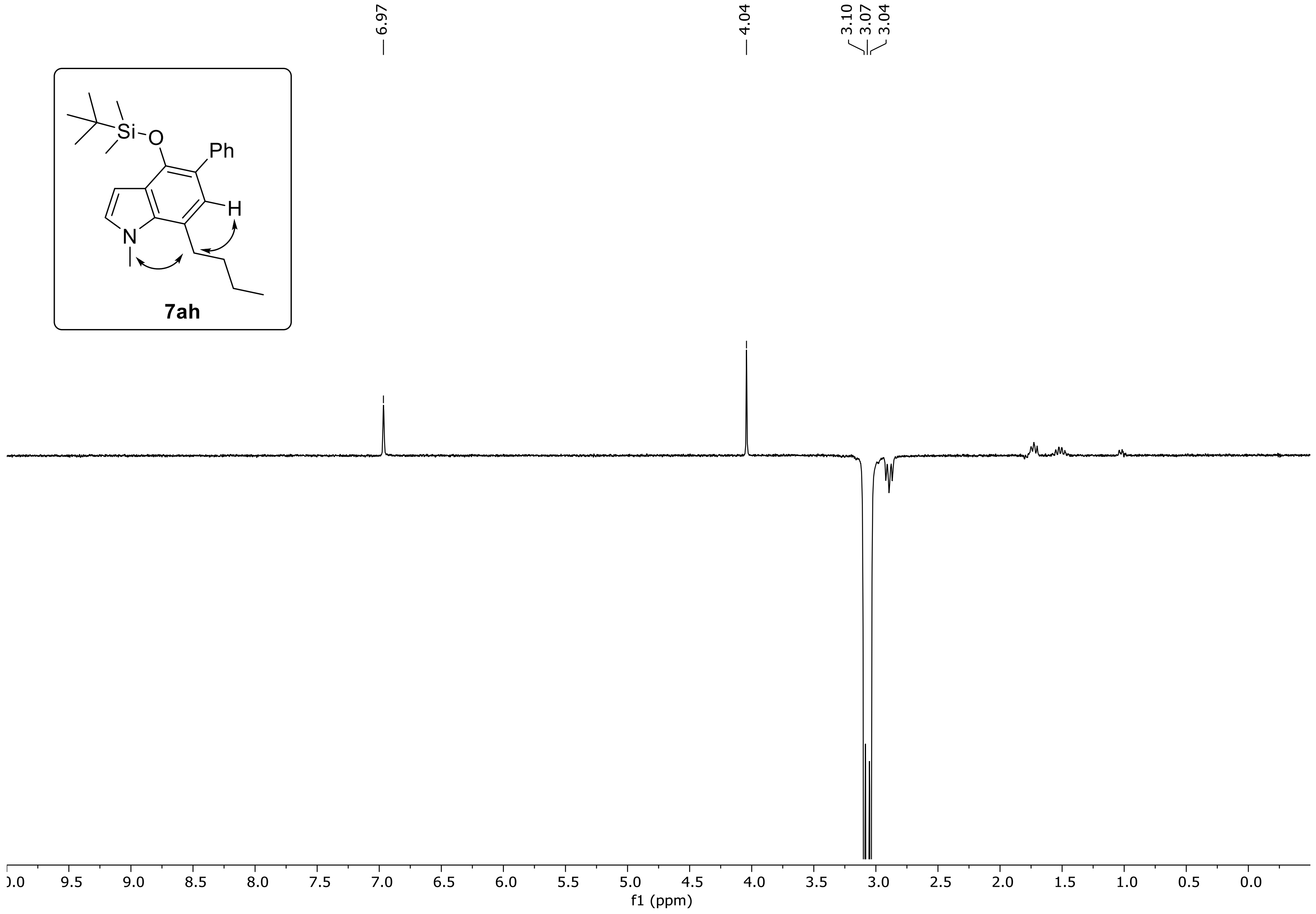

Figure S167: ^{1}H NMR of compound **7ai** in $CDCl_3$ at 300 MHz.

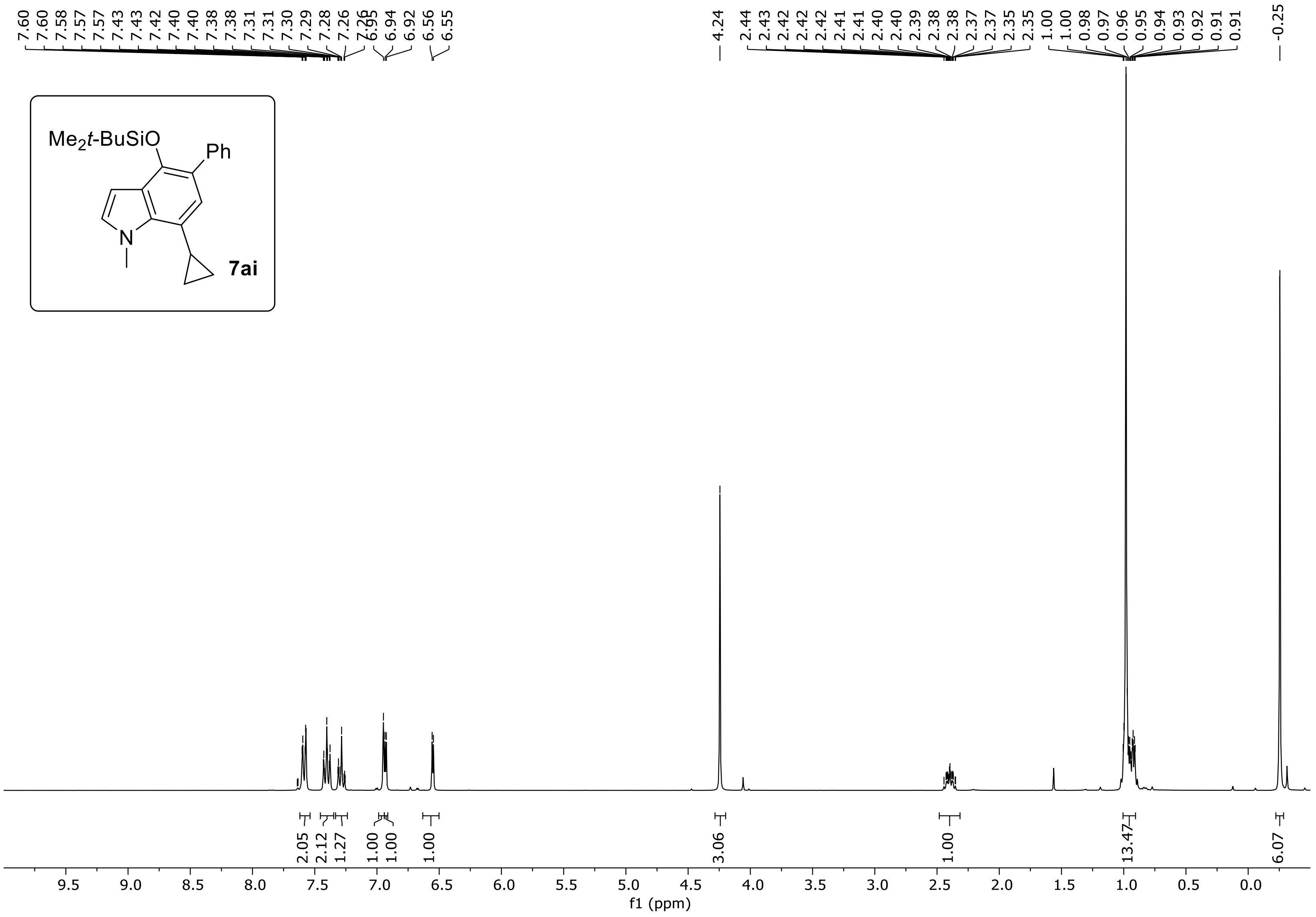

Figure S168: ^{13}C NMR of compound **7ai** in $CDCl_3$ at 75.4 MHz.

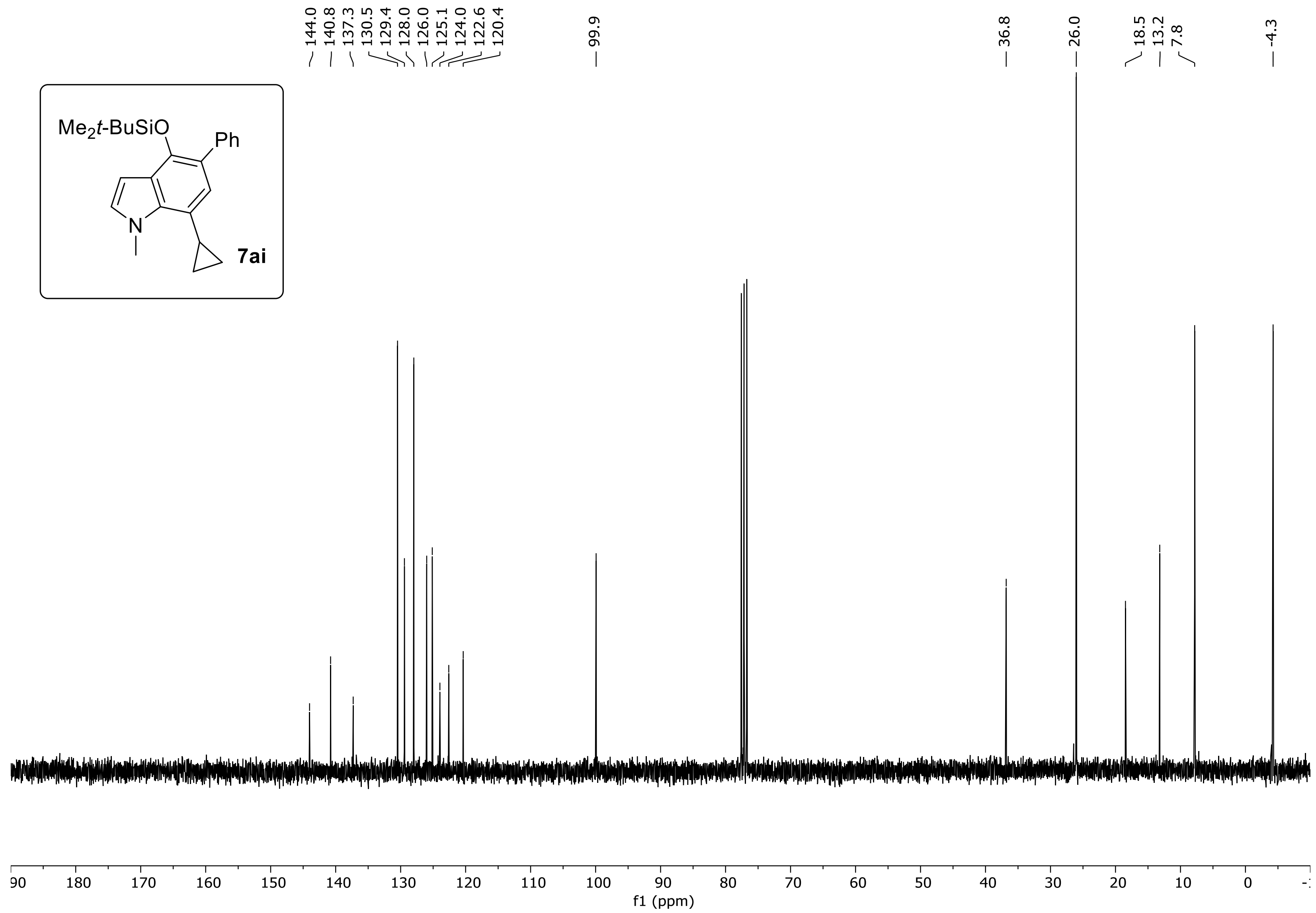

Figure S169: 1D NOE NMR of compound **7ai** in $CDCl_3$ at 300 MHz.

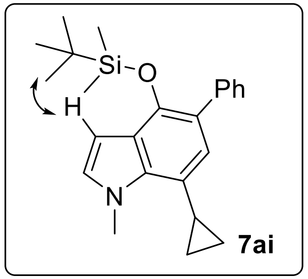

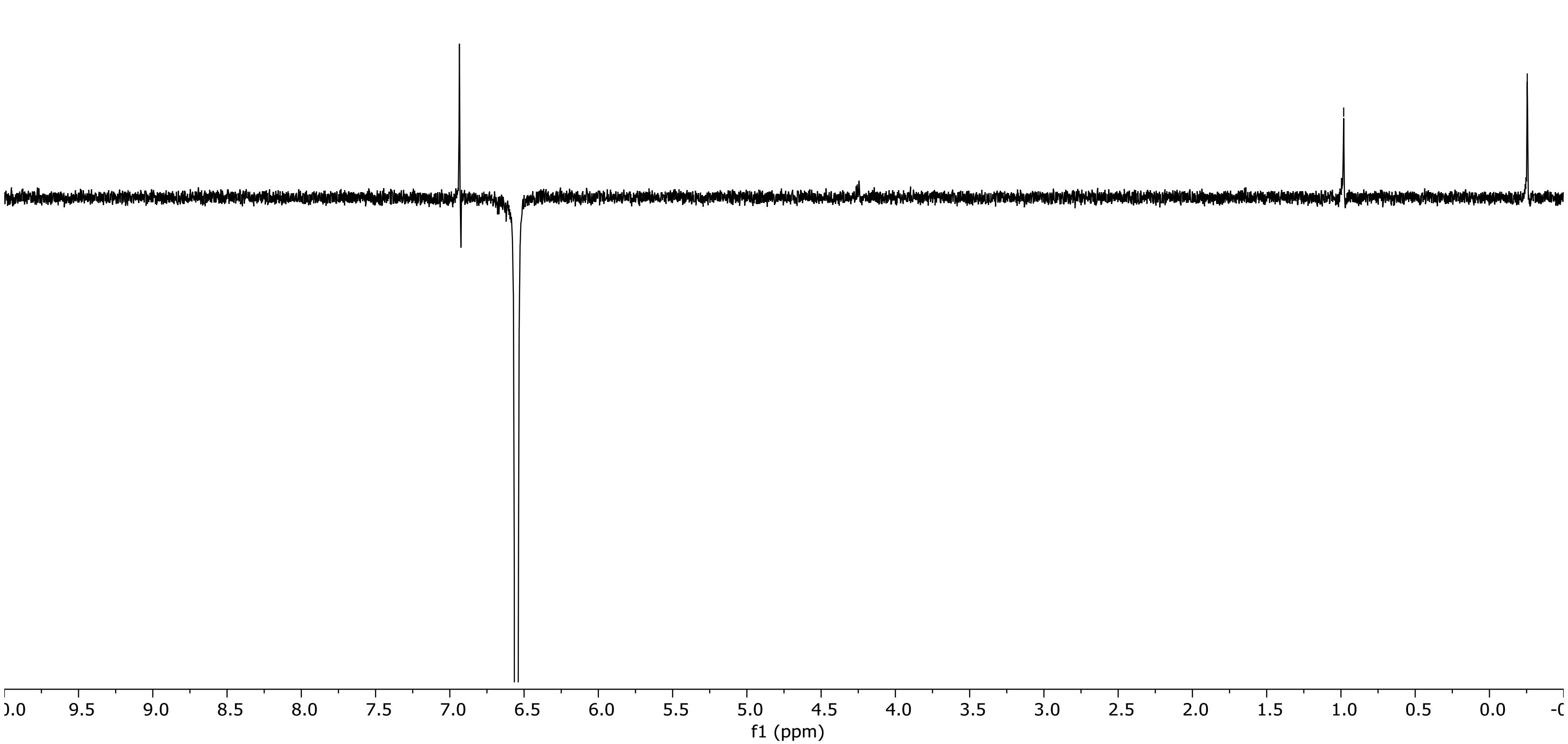

Figure S170: ^{1}H NMR of compound **7aj** in $CDCl_3$ at 300 MHz.

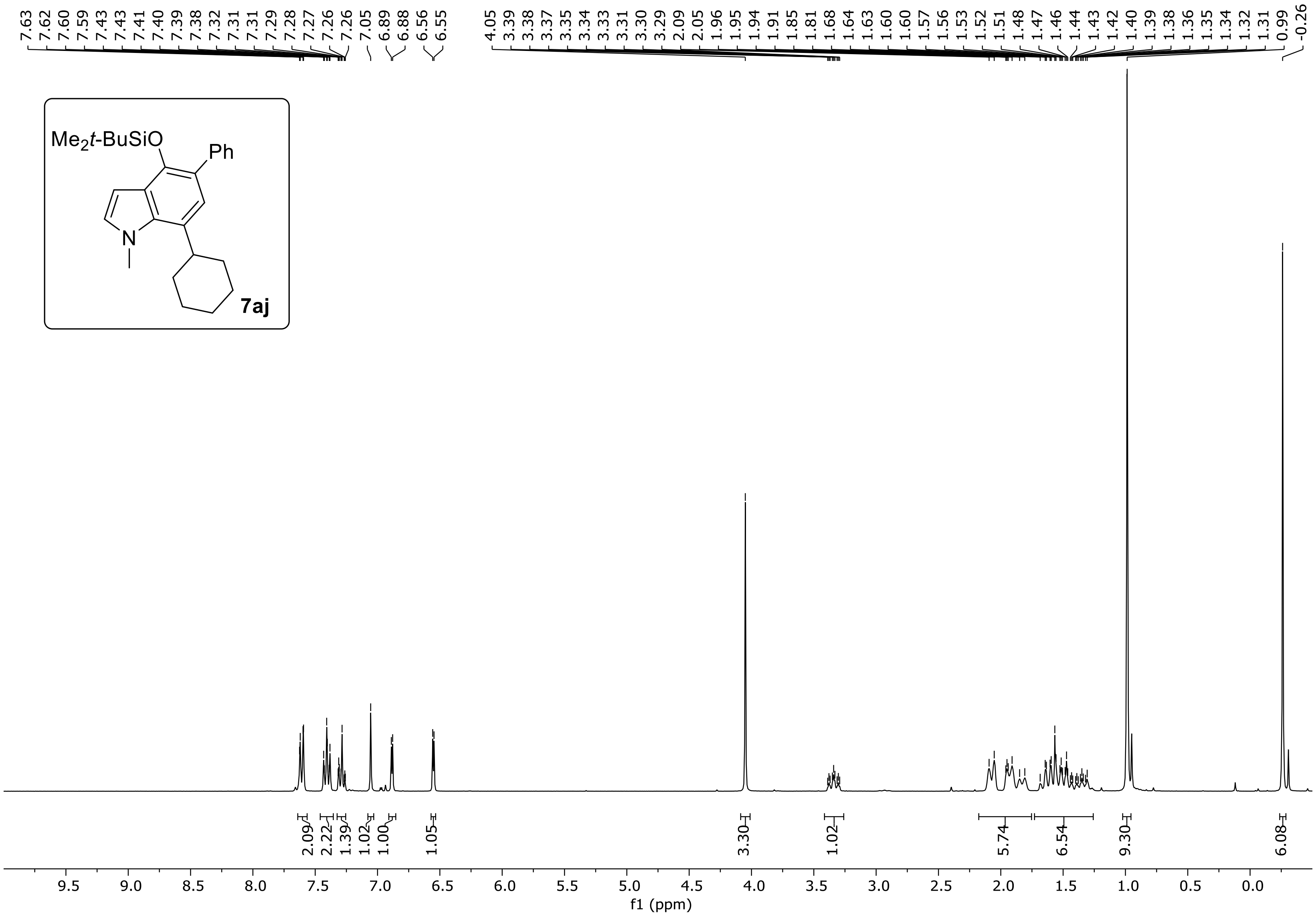

Figure S171: ^{13}C NMR of compound **7aj** in $CDCl_3$ at 75.4 MHz.

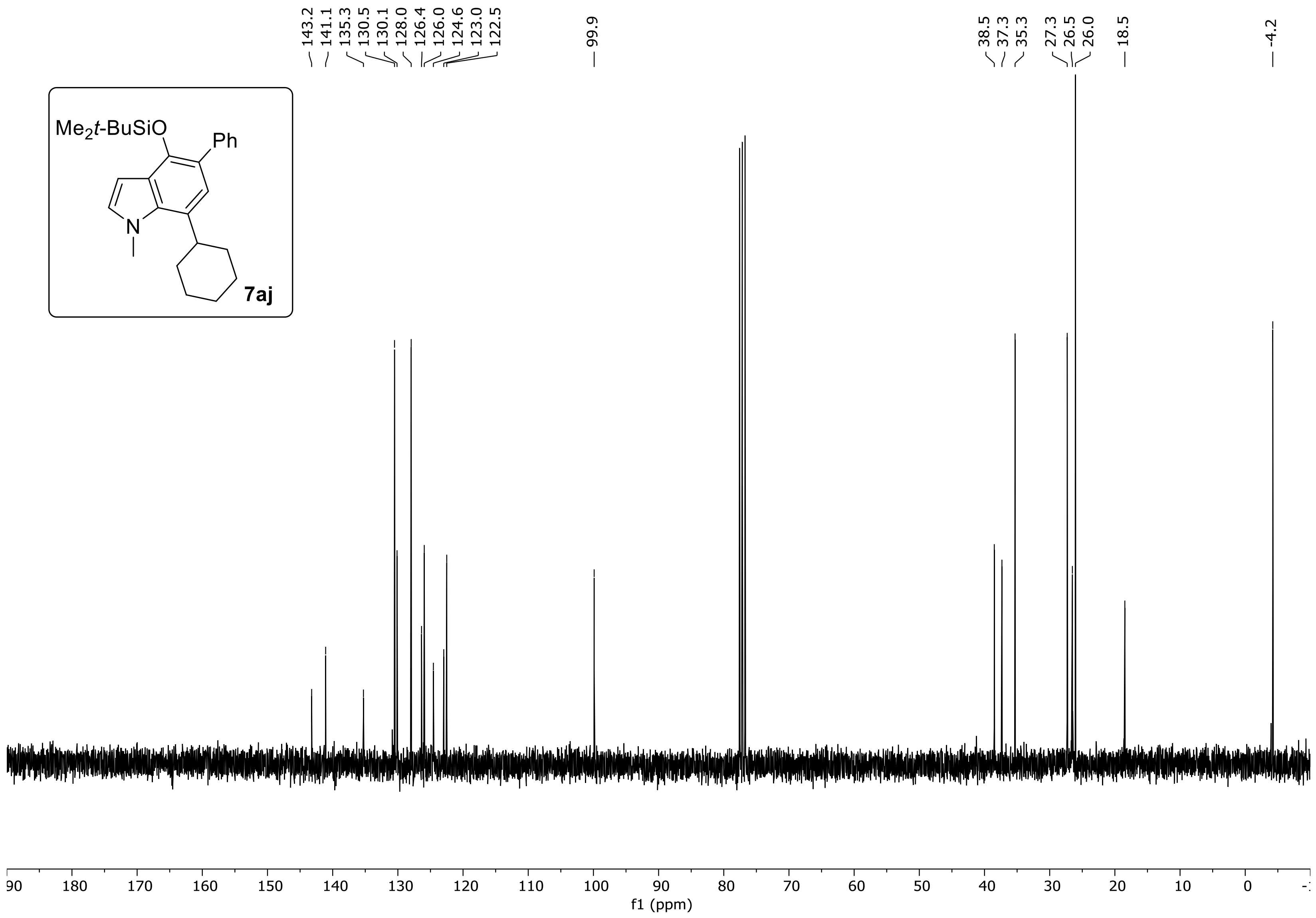

Figure S172: 1D NOE NMR of compound **7aj** in $CDCl_3$ at 300 MHz.

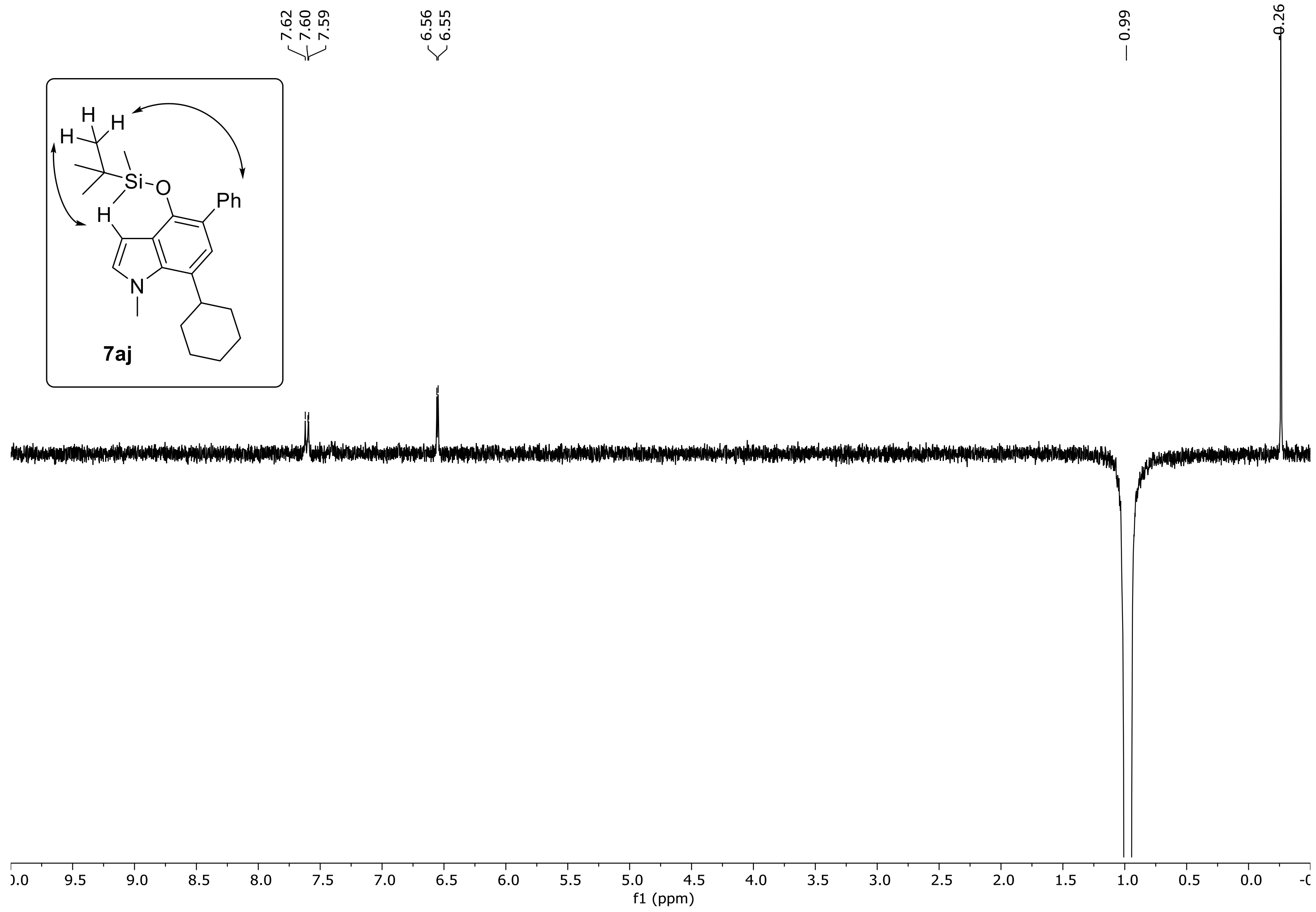

Figure S173: ^{1}H NMR of compound **7ak** in $CDCl_3$ at 300 MHz.

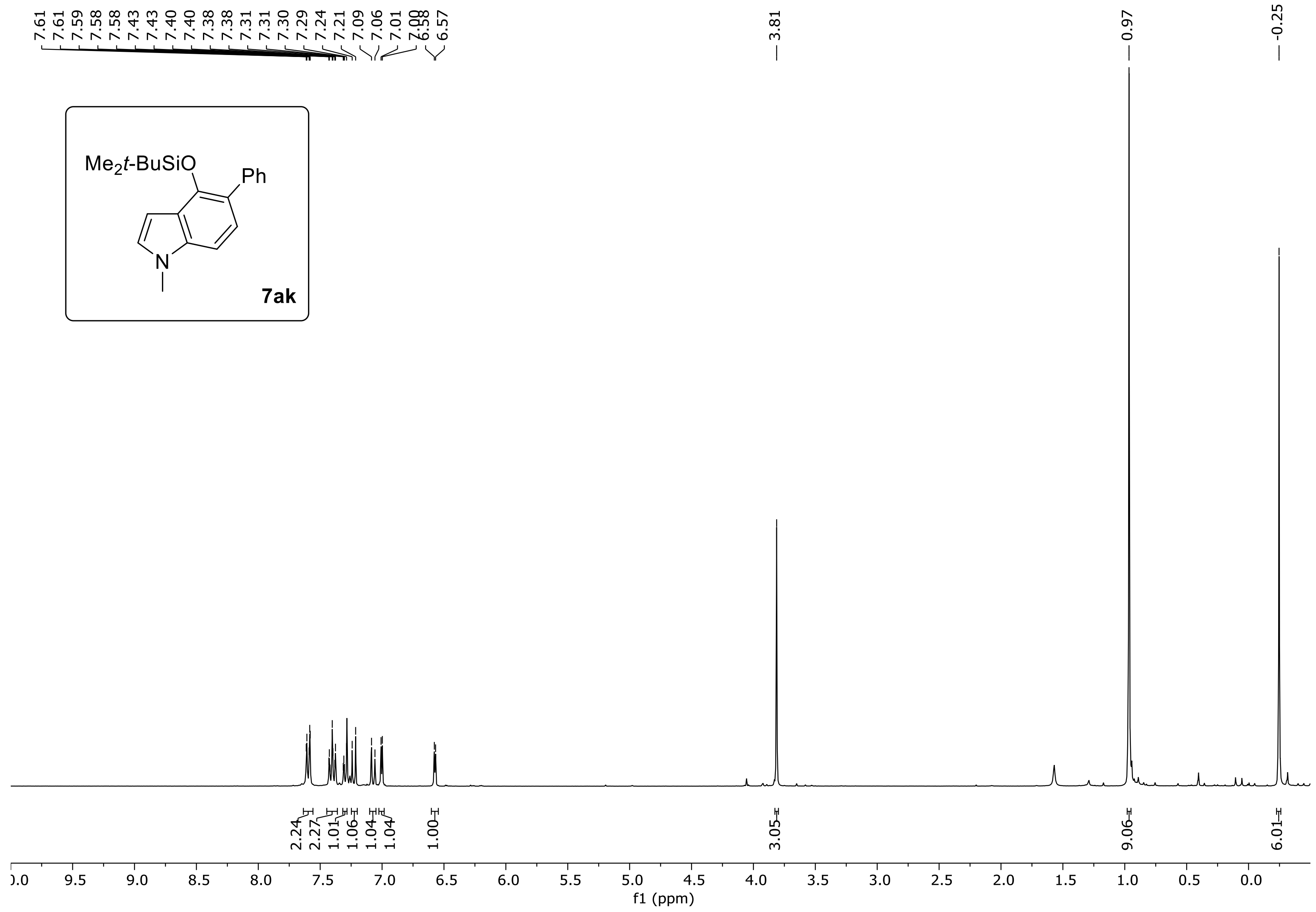

Figure S174: ^{13}C NMR of compound **7ak** in $CDCl_3$ at 75.4 MHz.

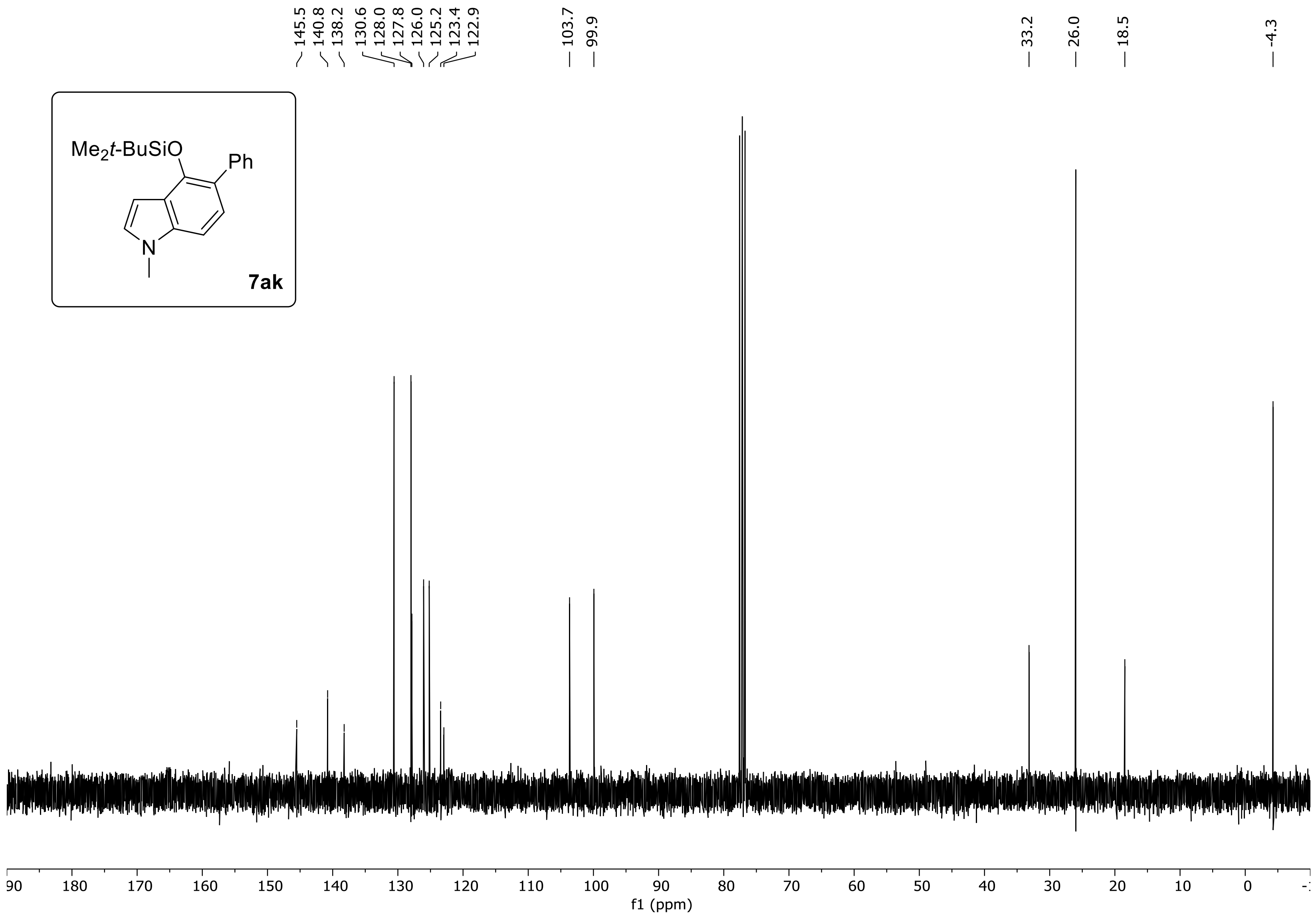

Figure S175a: 1D NOE NMR of compound **7ak** in $CDCl_3$ at 300 MHz.

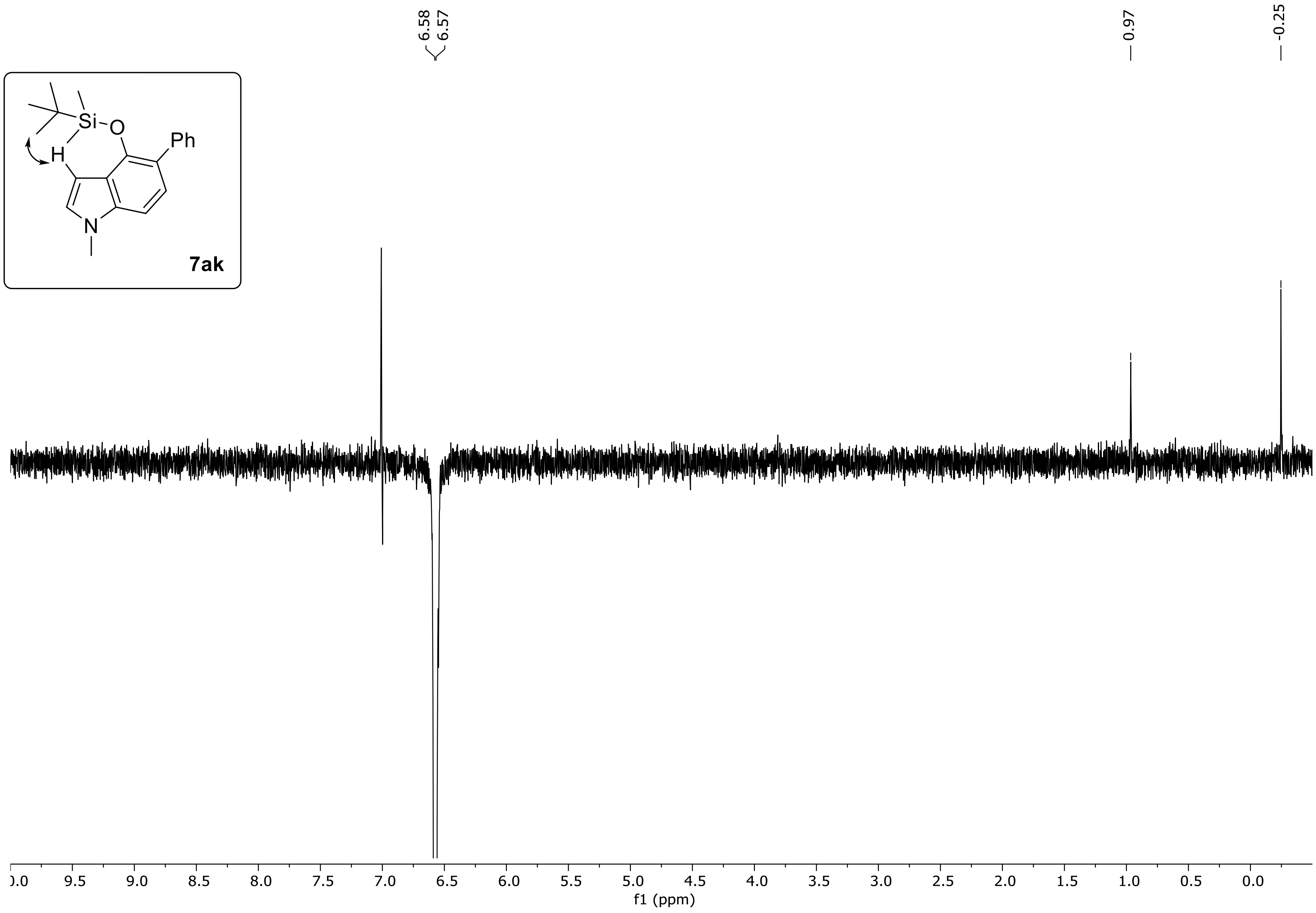

Figure S175b: 1D NOE NMR of compound **7ak** in $CDCl_3$ at 300 MHz.

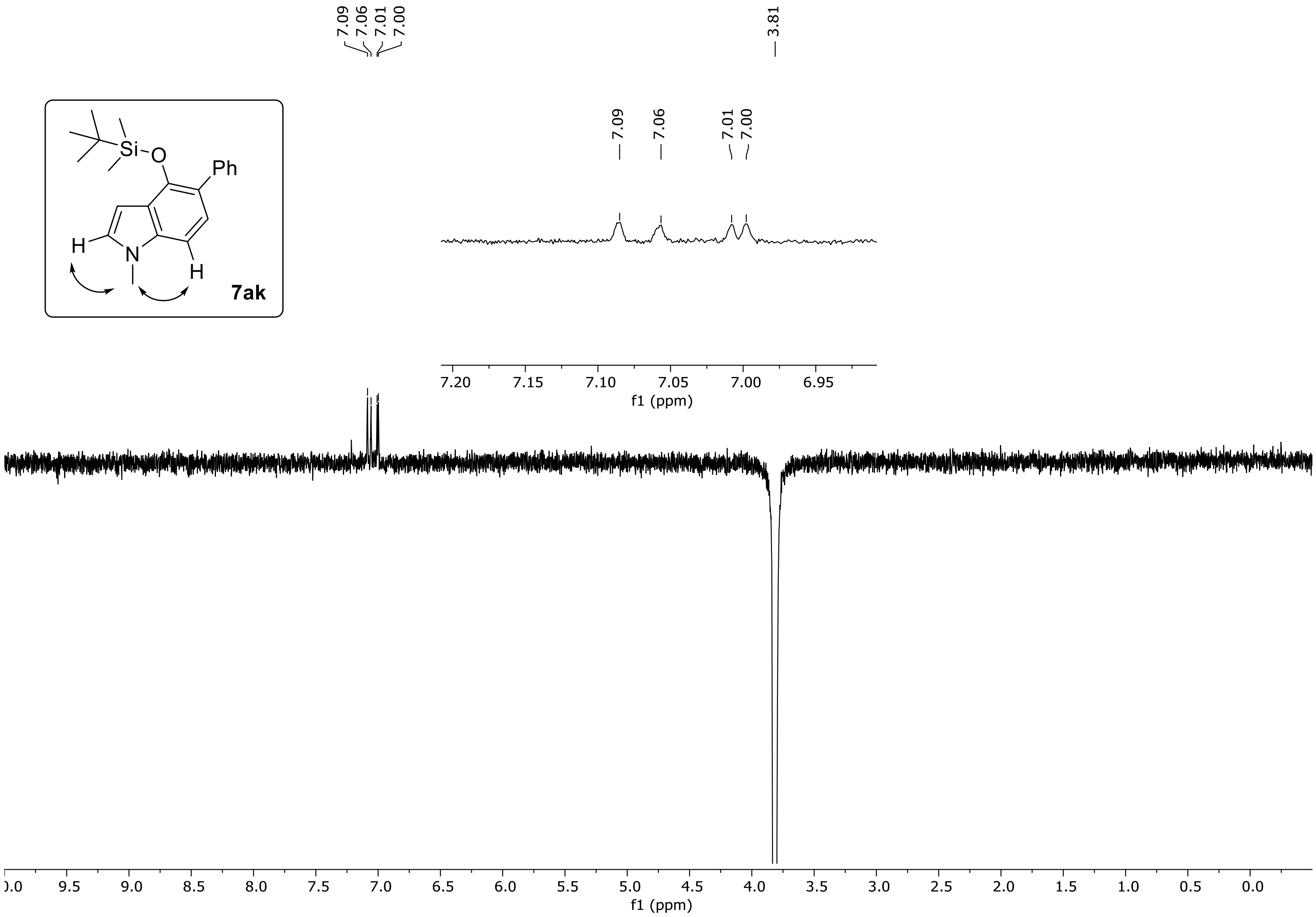

Figure S176: ^{1}H NMR of compound **7ba** in $CDCl_3$ at 300 MHz.

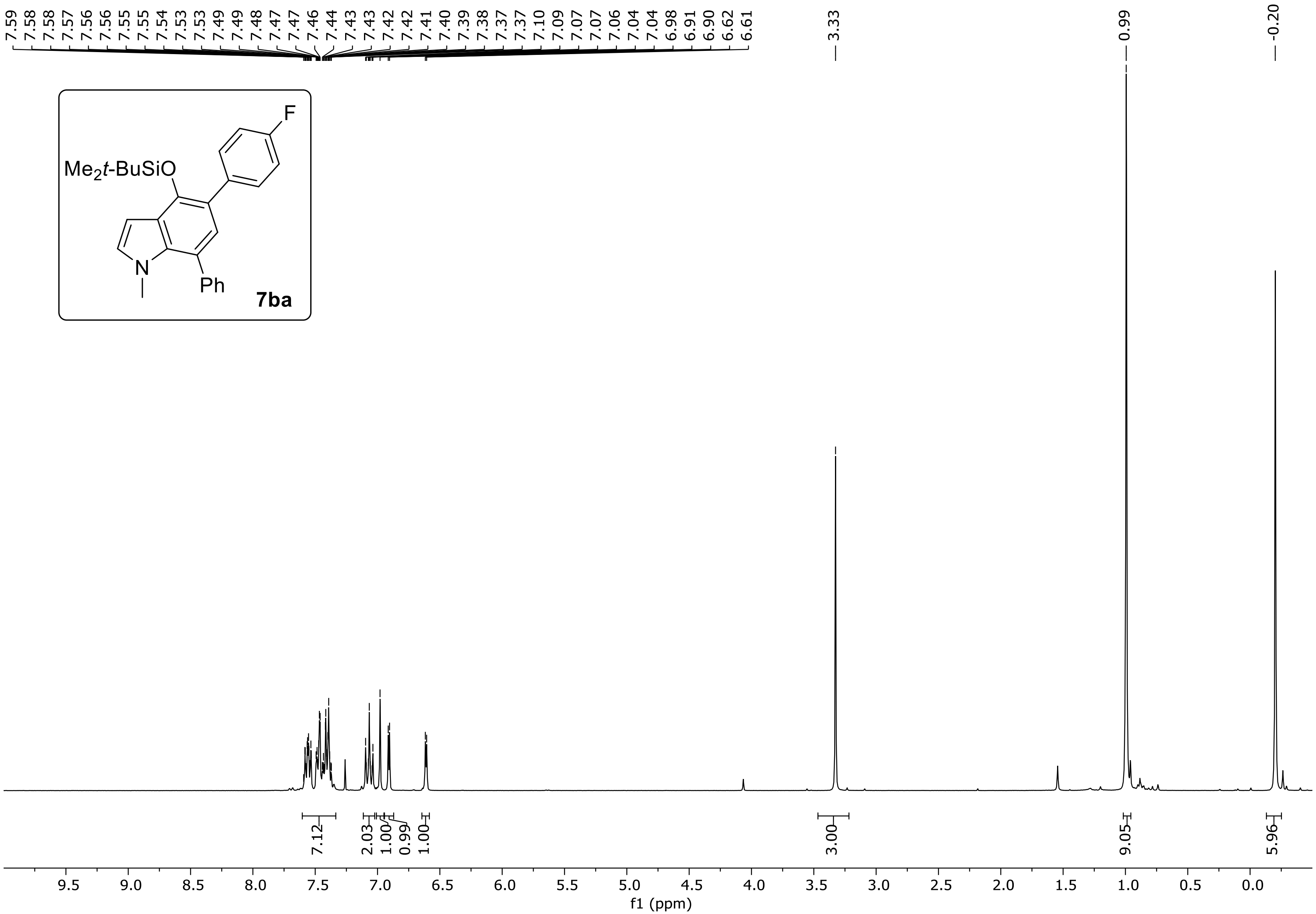

Figure S177: ^{13}C NMR of compound **7ba** in $CDCl_3$ at 75.4 MHz.

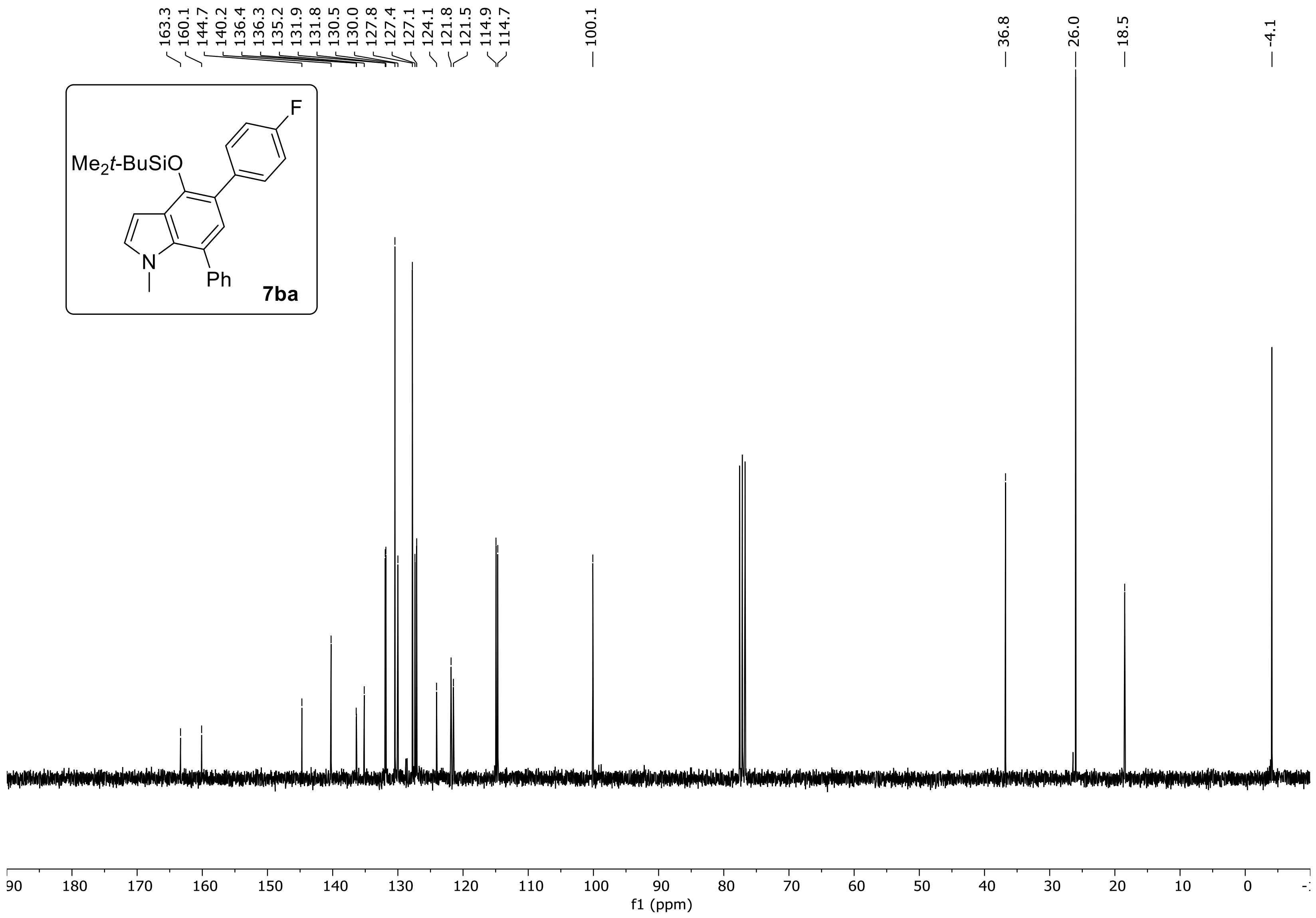

Figure S178: ^{1}H NMR of compound **7bf** in $CDCl_3$ at 300 MHz.

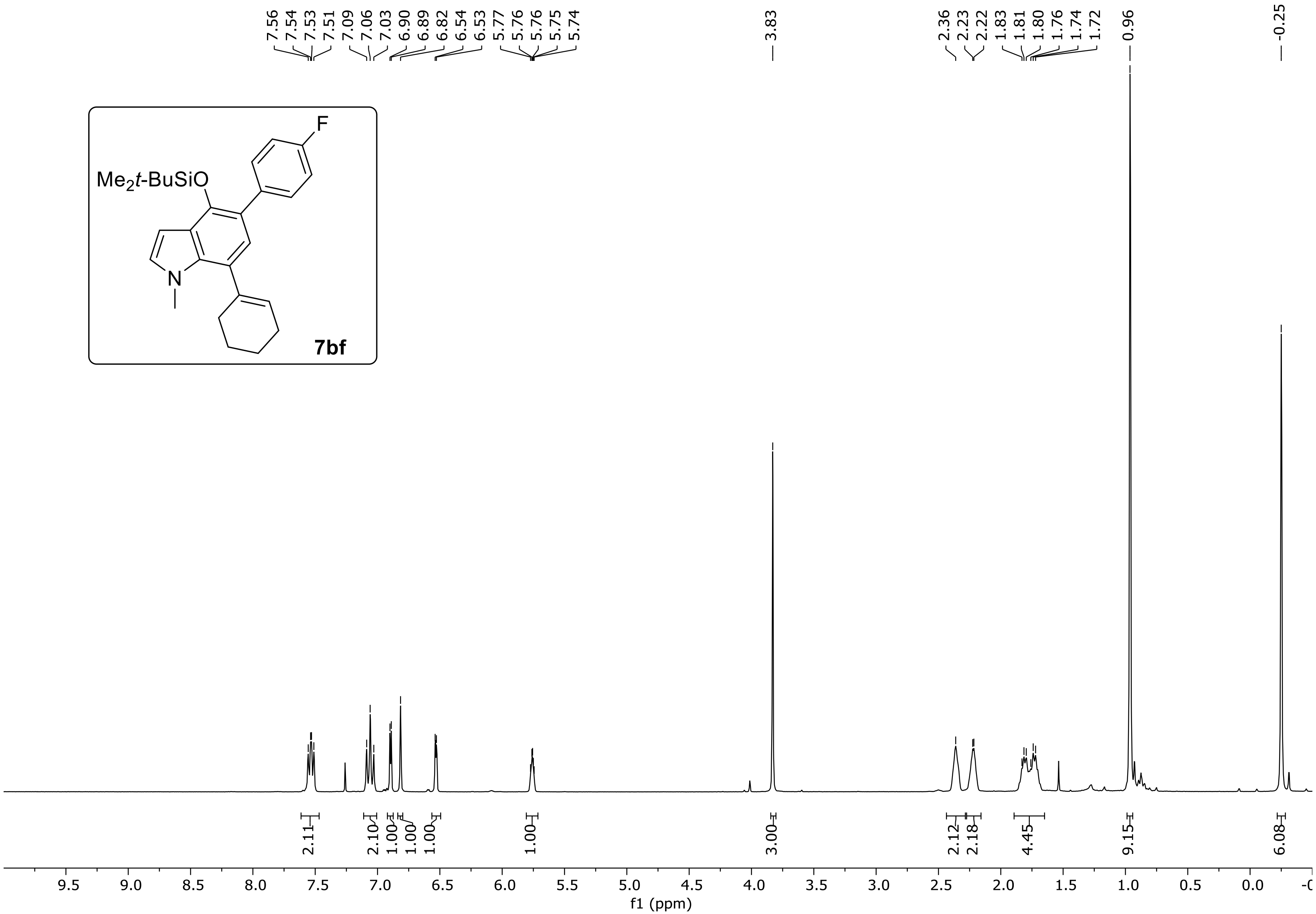

Figure S179: ^{13}C NMR of compound **7bf** in $CDCl_3$ at 75.4 MHz.

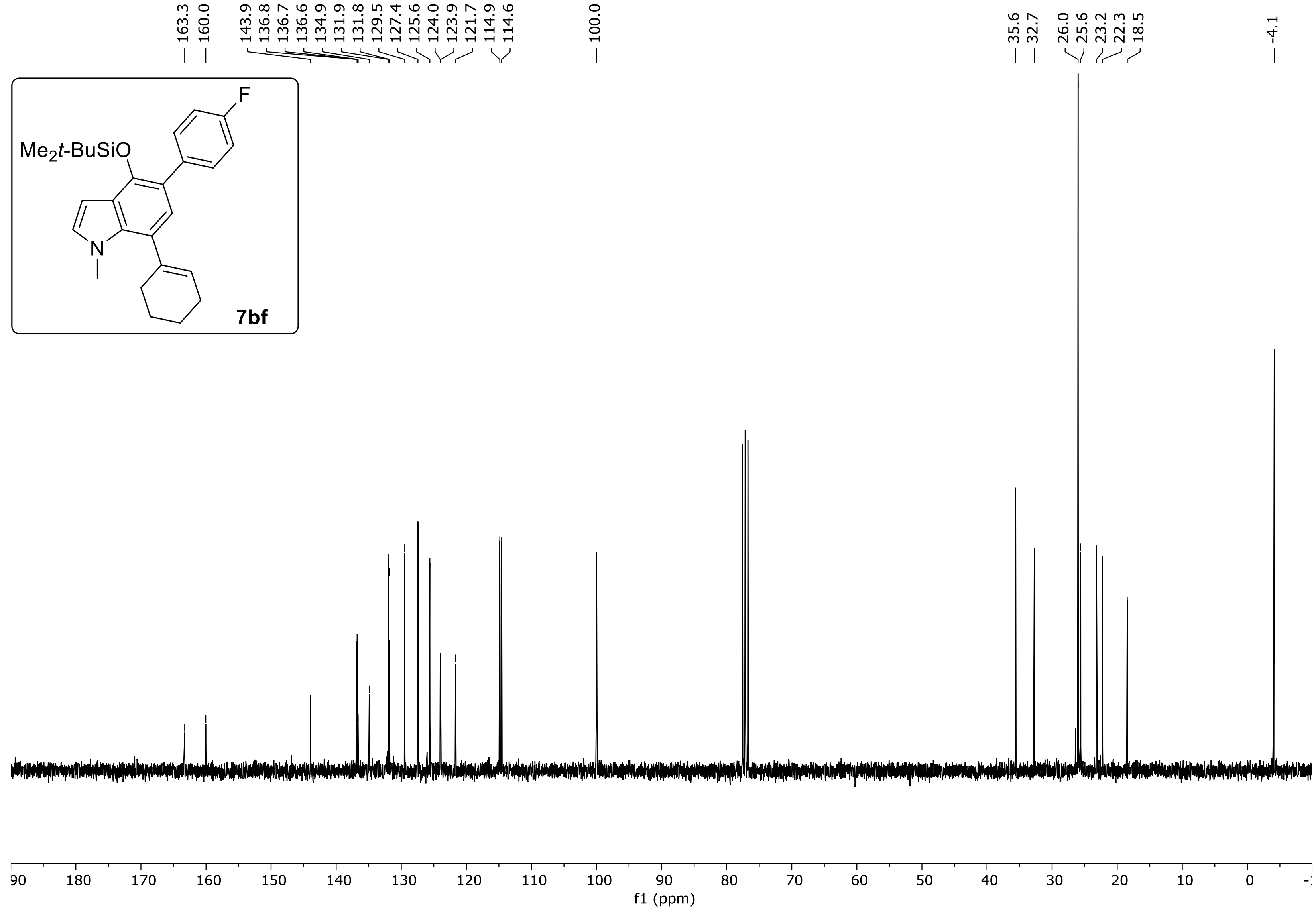

Figure S180a: 1D NOE NMR of compound **7bf** in $CDCl_3$ at 300 MHz.

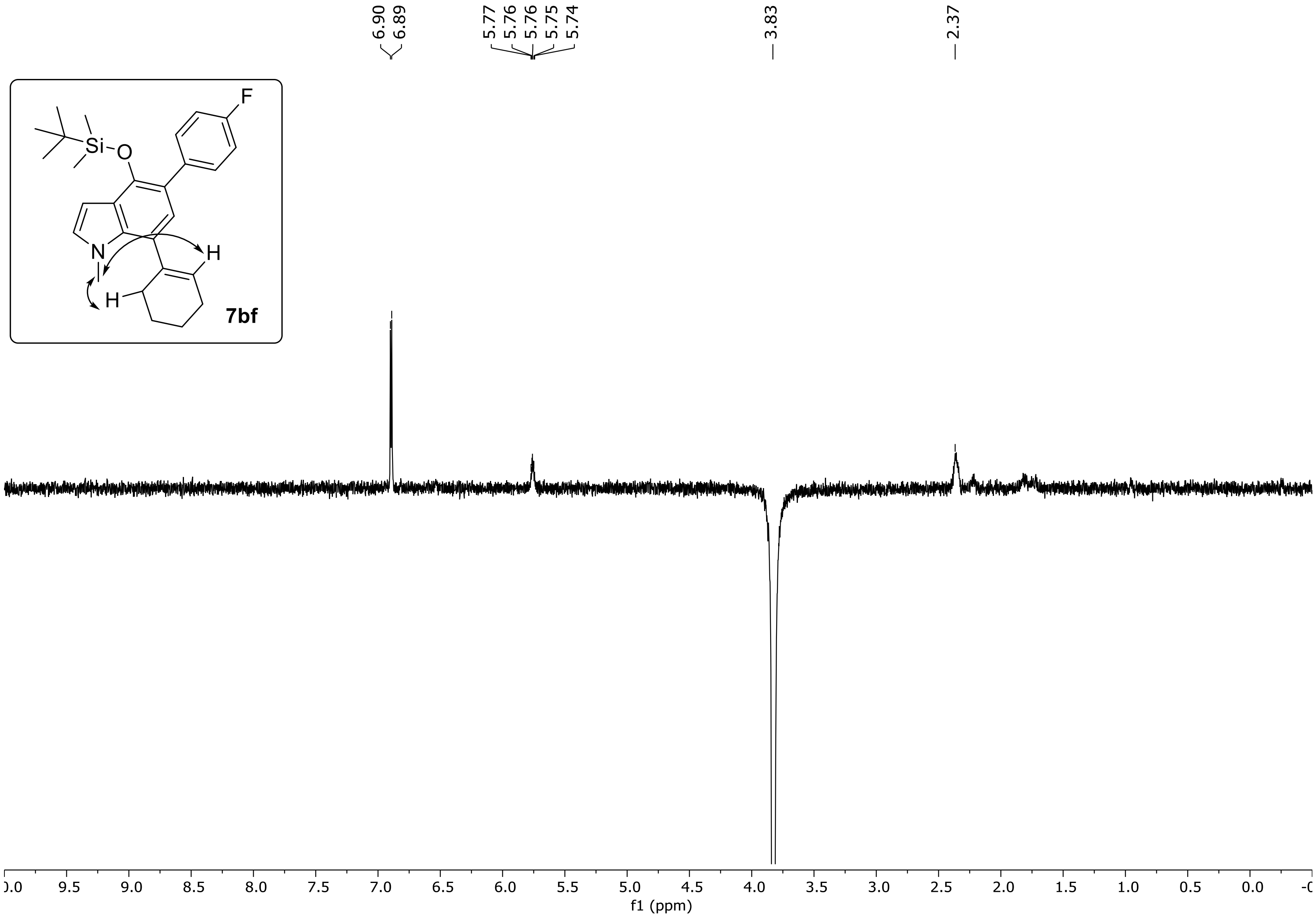

Figure S180b: 1D NOE NMR of compound **7bf** in $CDCl_3$ at 300 MHz.

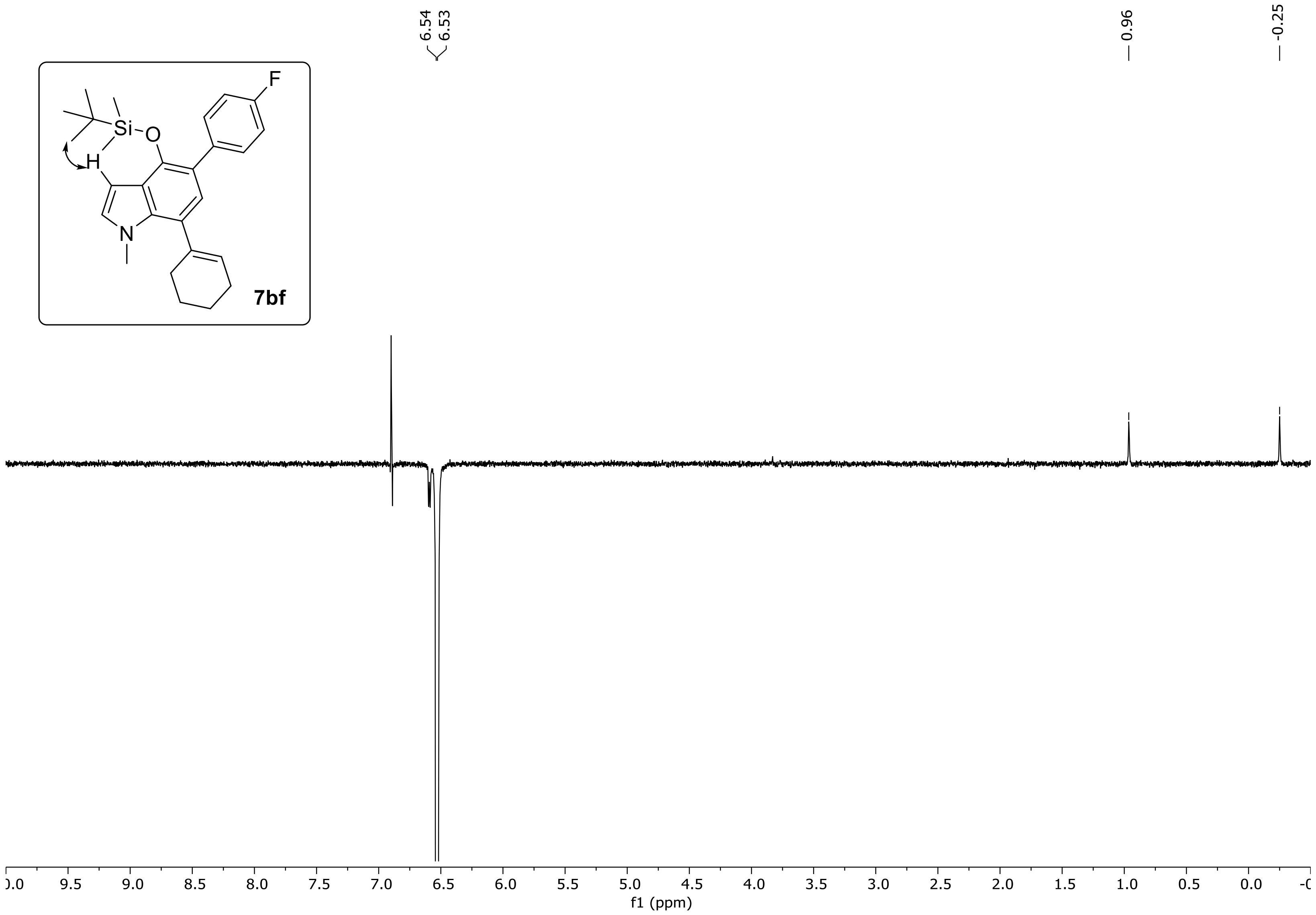

Figure S181: ^{1}H NMR of compound **7ca** in $CDCl_3$ at 300 MHz.

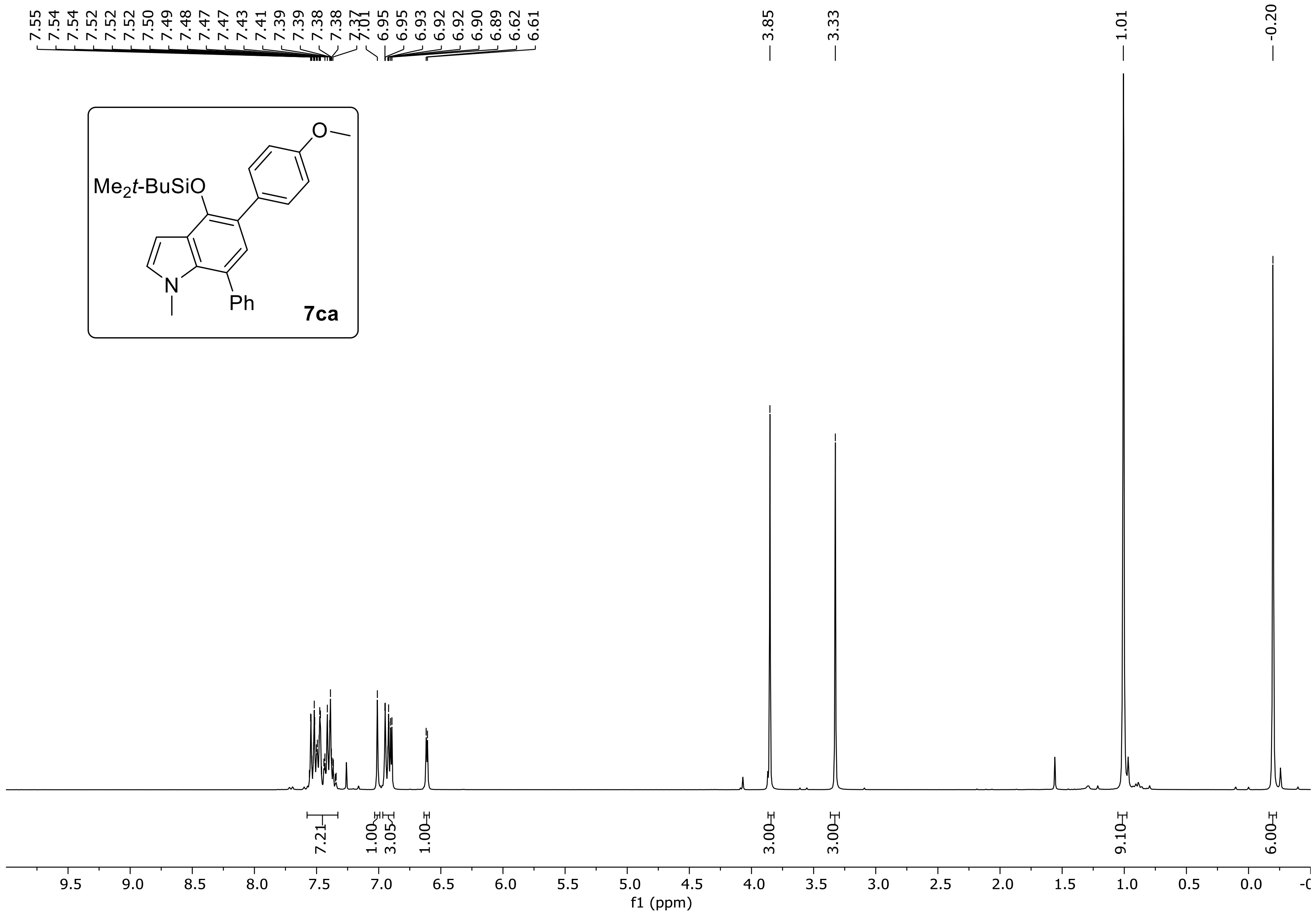

Figure S182: ^{13}C NMR of compound **7ca** in $CDCl_3$ at 75.4 MHz.

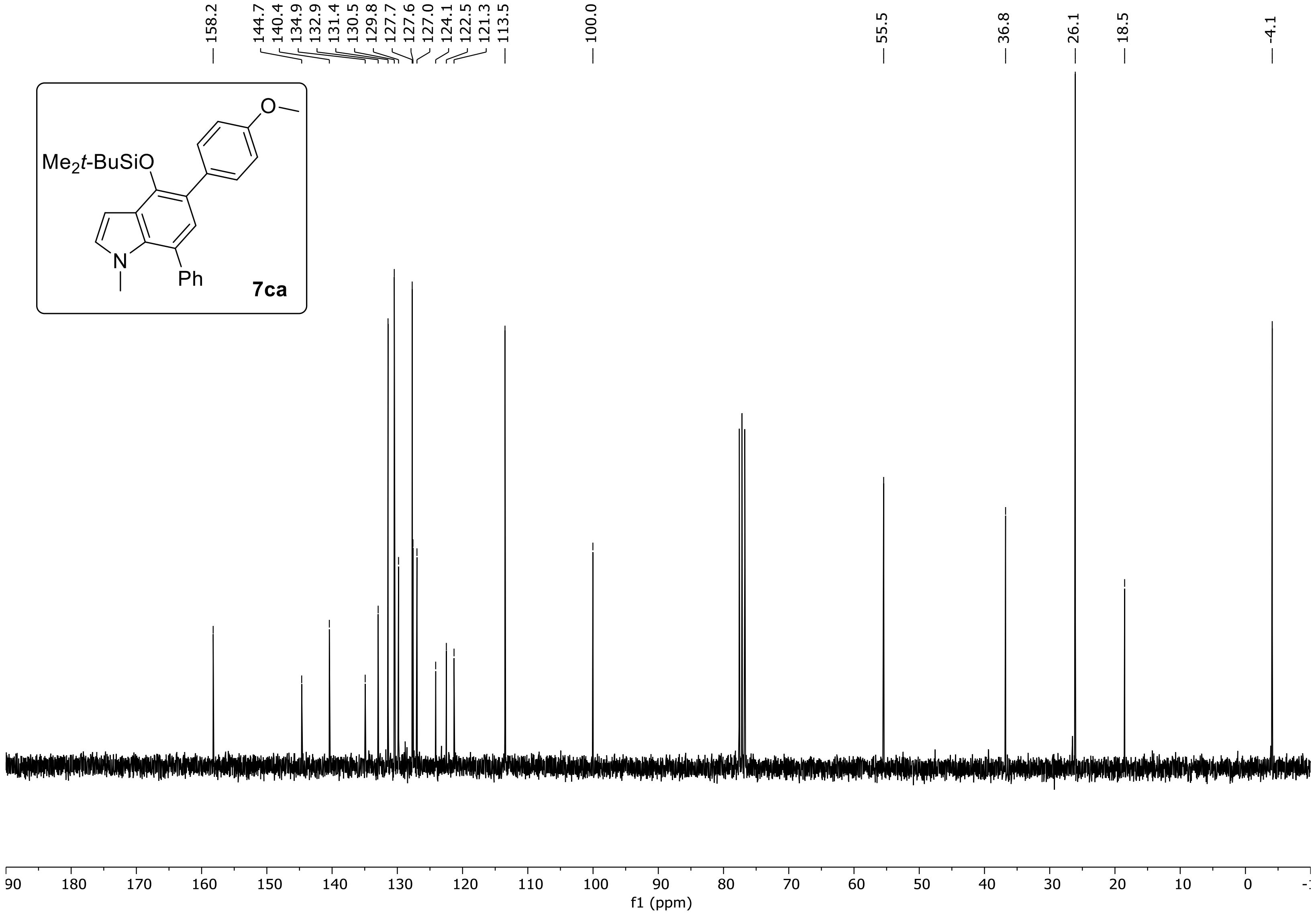

Figure S183: 1D NOE NMR of compound **7ca** in $CDCl_3$ at 300 MHz.

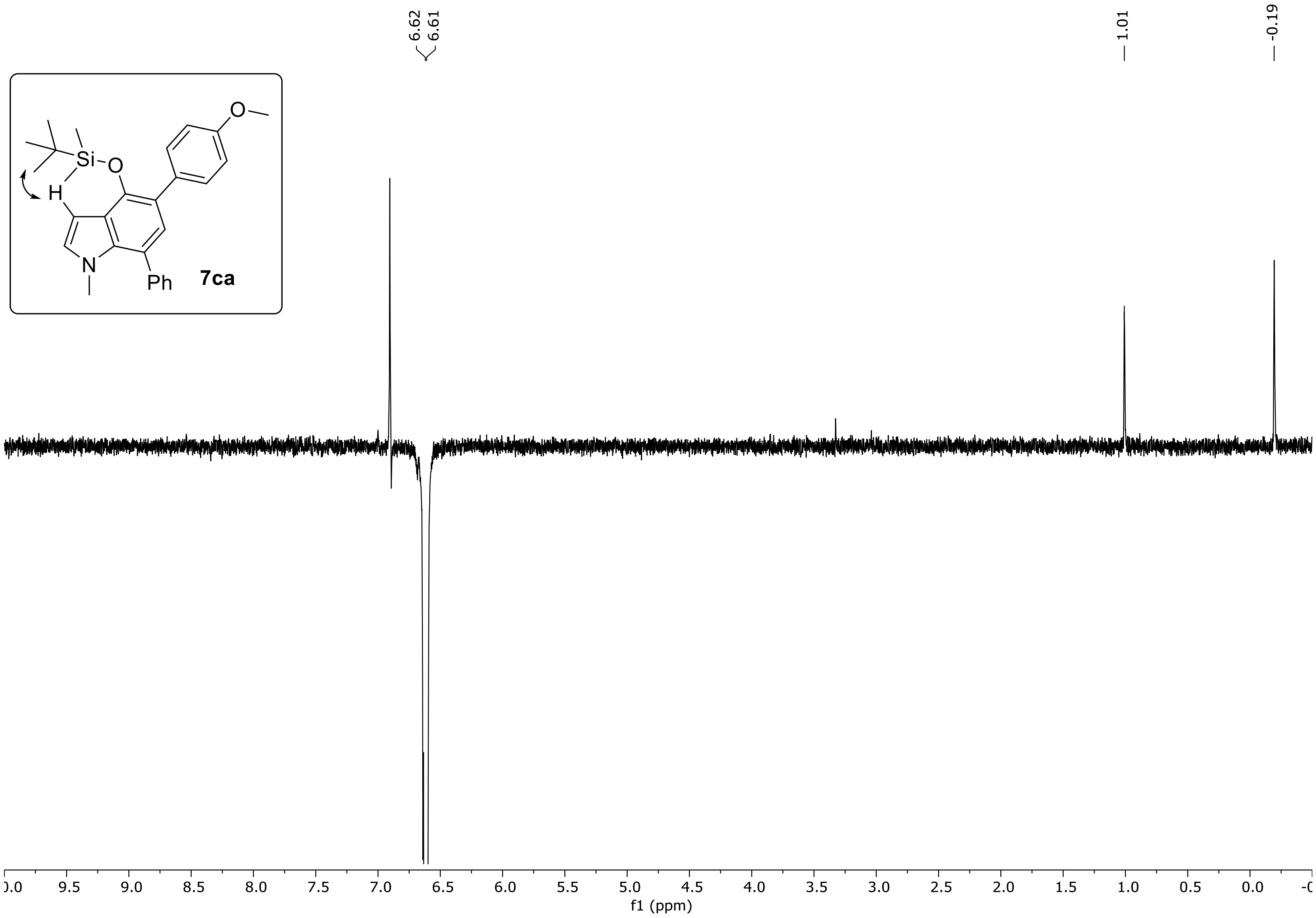

Figure S184: ^{1}H NMR of compound **7da** in $CDCl_3$ at 300 MHz.

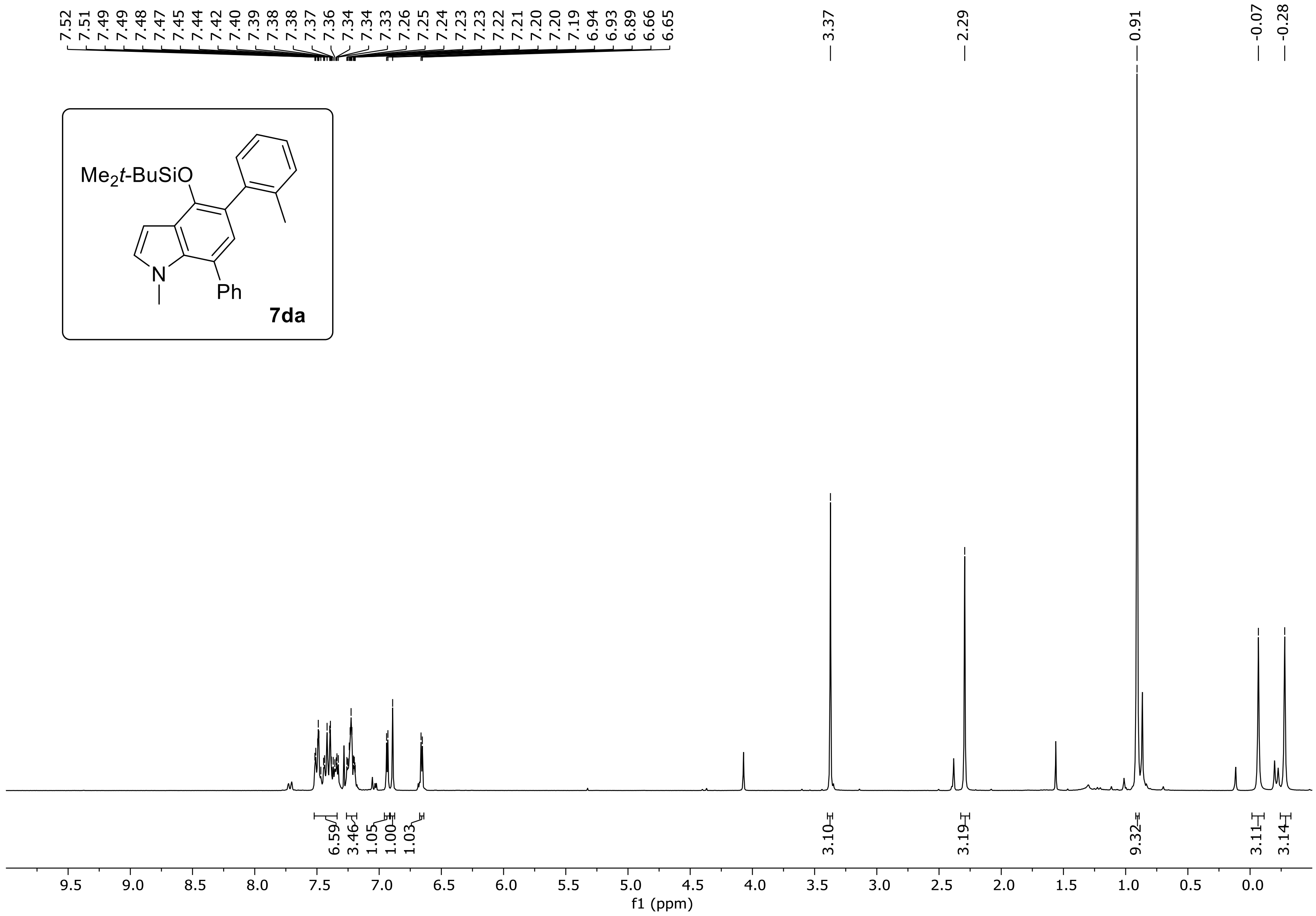

Figure S185: ^{13}C NMR of compound **7da** in $CDCl_3$ at 75.4 MHz.

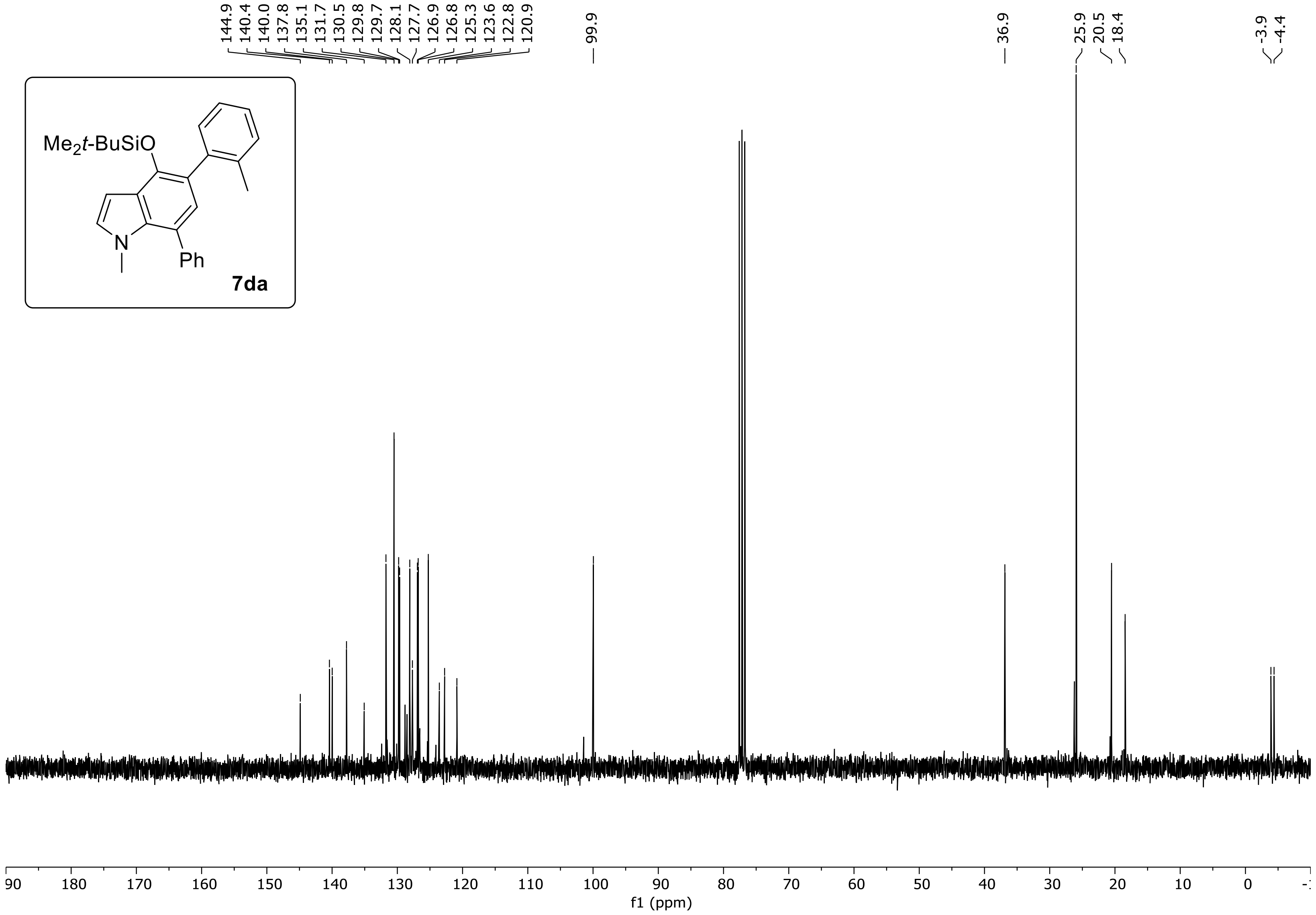

Figure S186: ^{1}H NMR of compound **7ea** in $CDCl_3$ at 300 MHz.

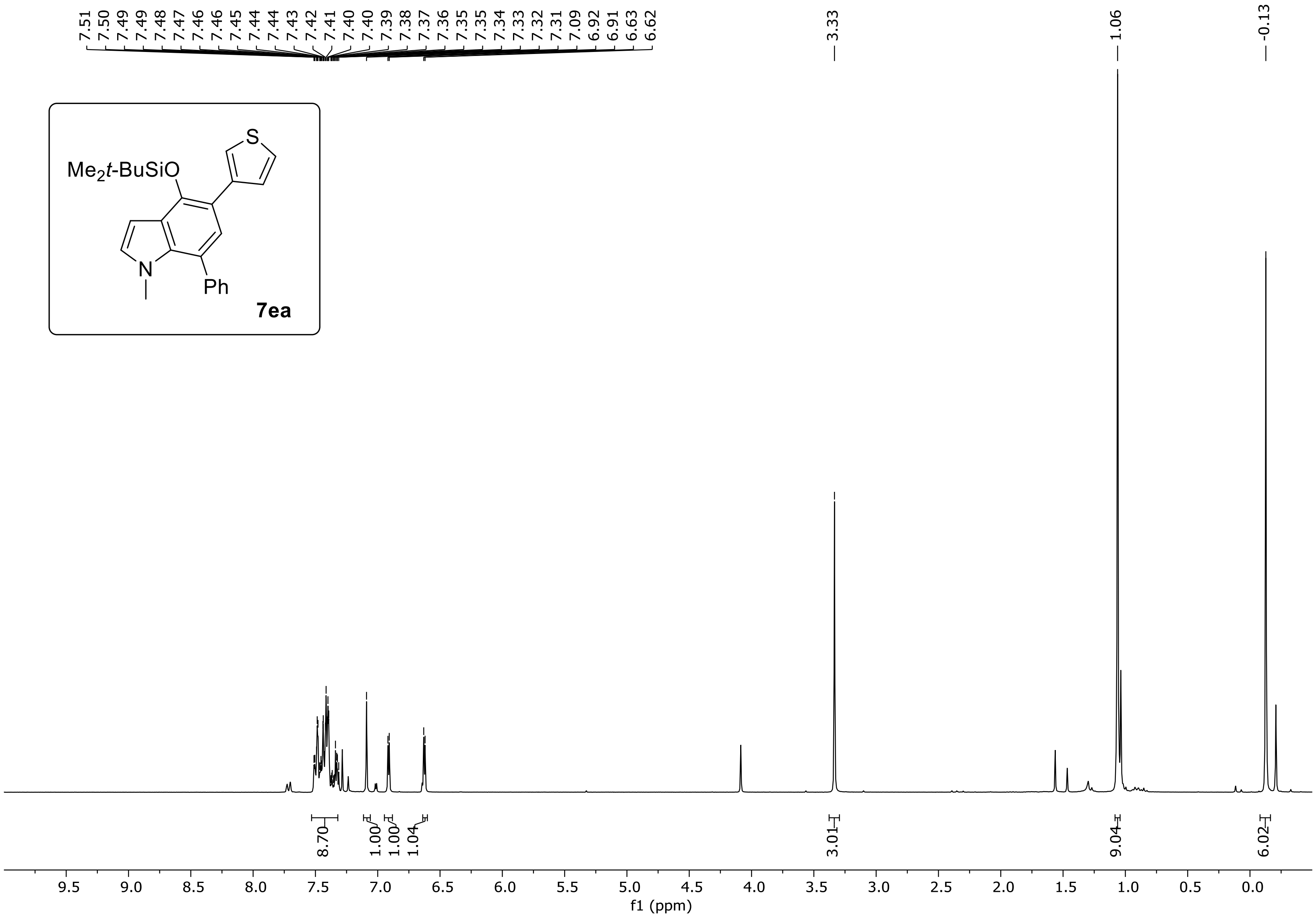

Figure S187: ^{13}C NMR of compound **7ea** in $CDCl_3$ at 75.4 MHz.

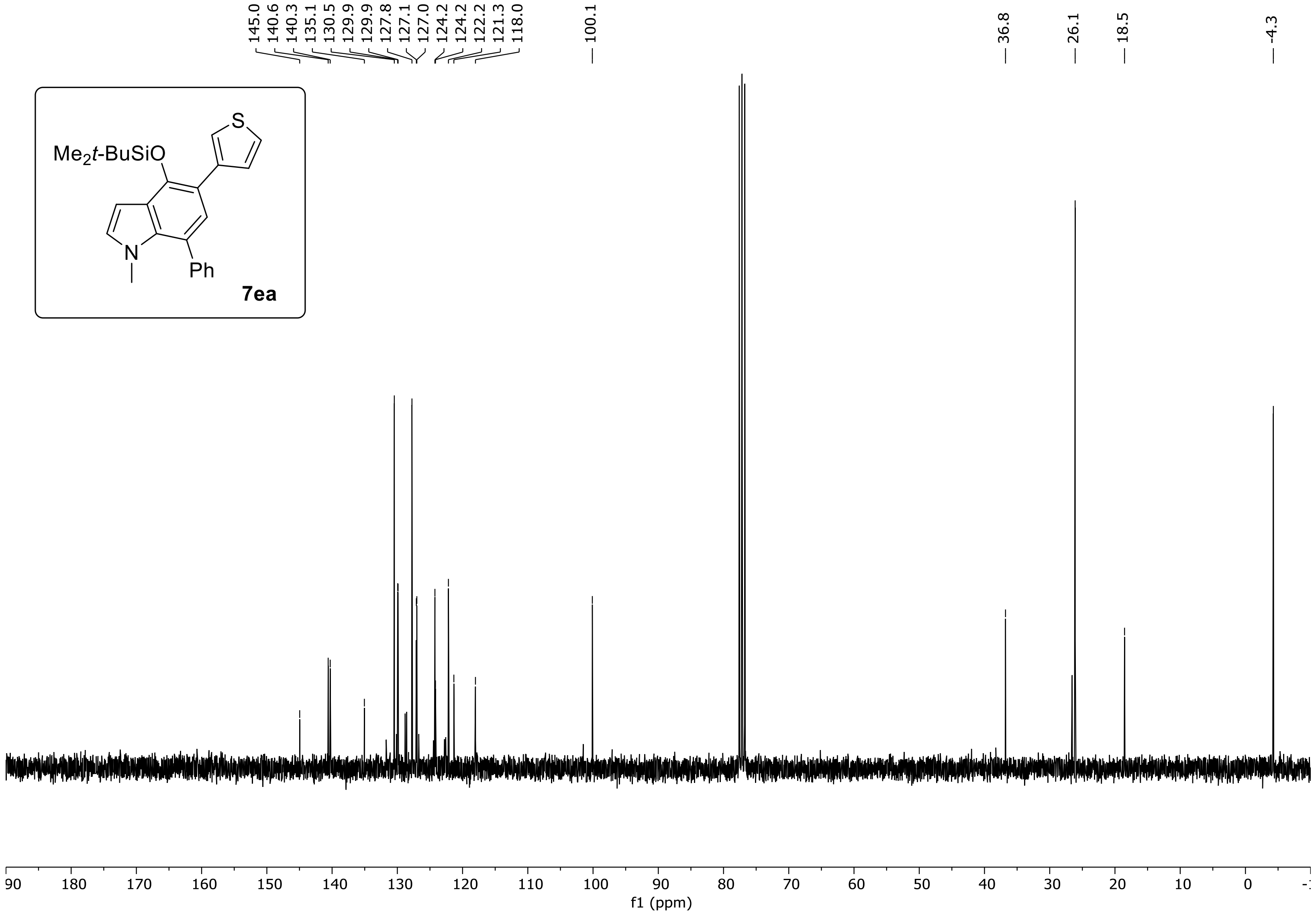

Figure S188: 1D NOE NMR of compound **7ea** in $CDCl_3$ at 300 MHz.

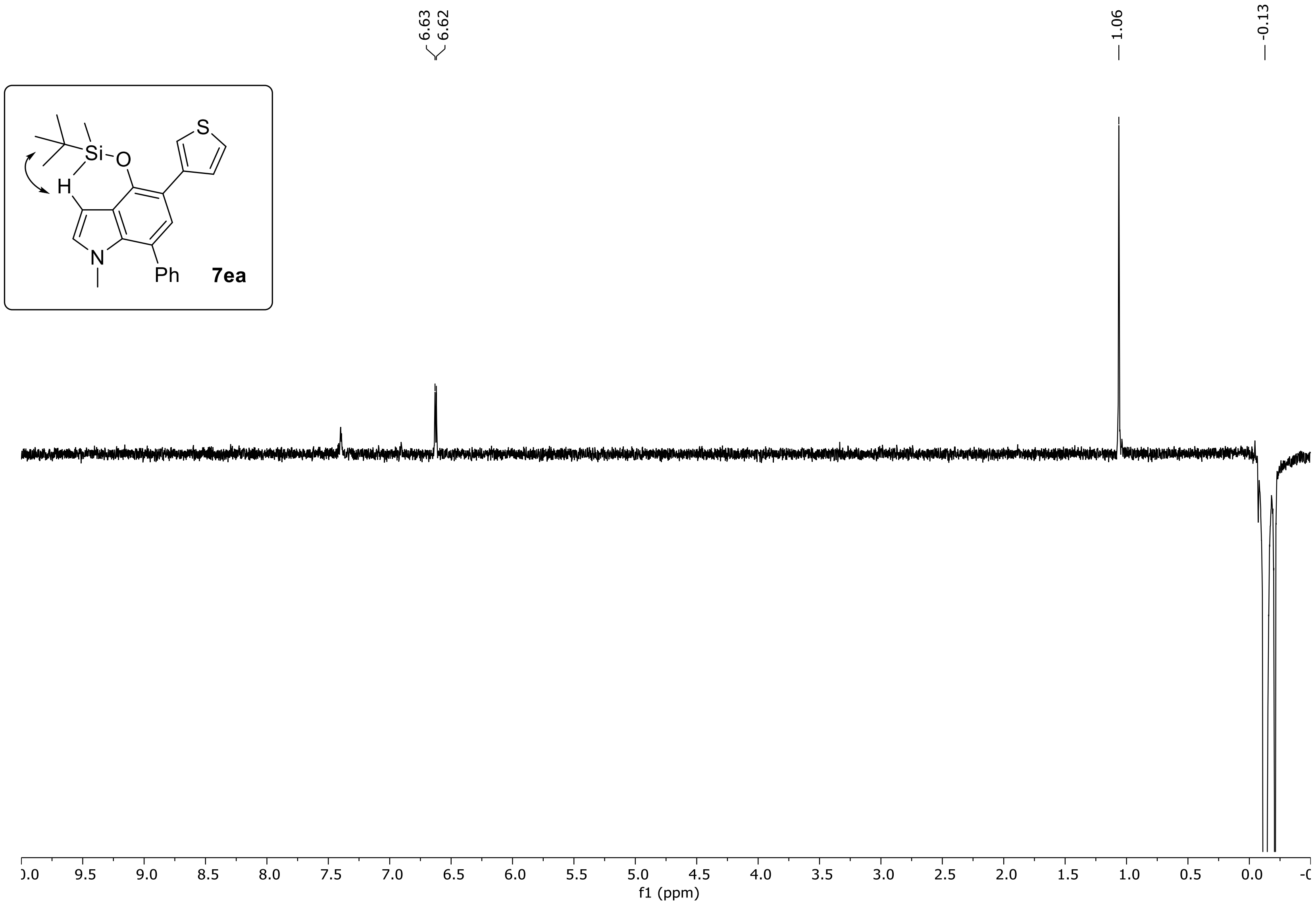

Figure S189: ^{1}H NMR of compound **7fa** in $CDCl_3$ at 300 MHz.

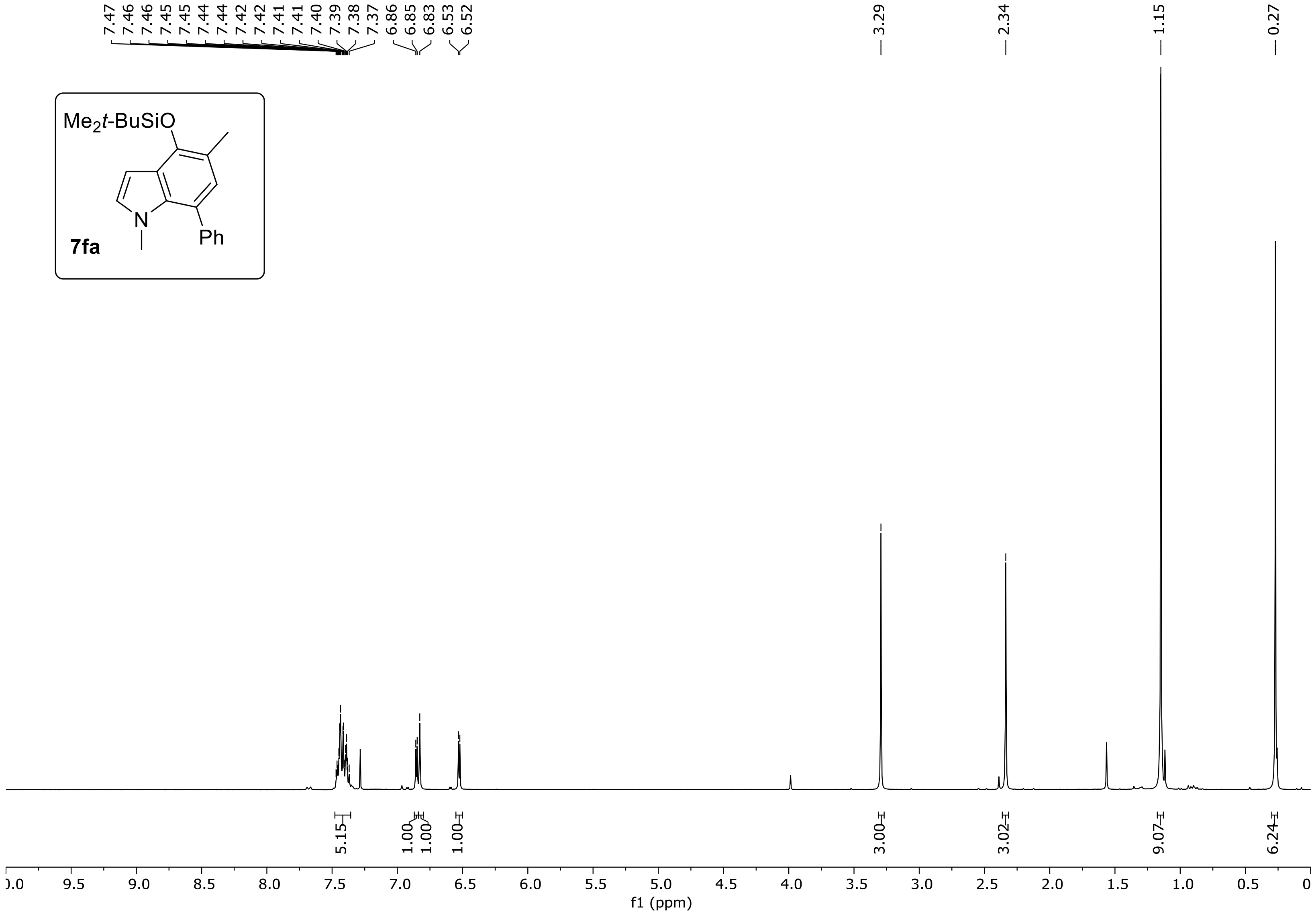

Figure S190: ^{13}C NMR of compound **7fa** in $CDCl_3$ at 75.4 MHz.

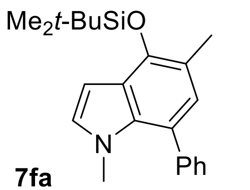

7fa

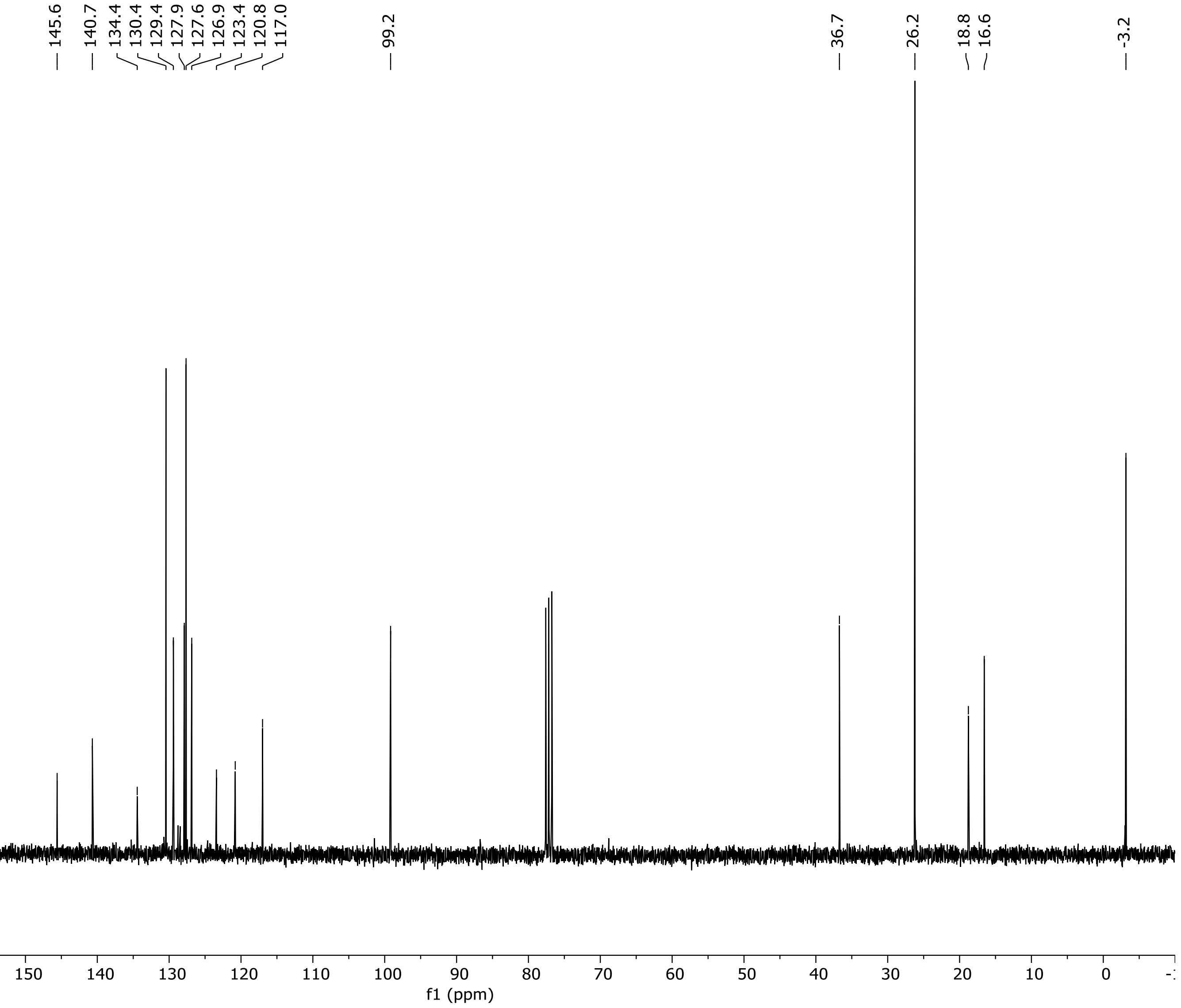

Figure S191: 1D NOE NMR of compound **7fa** in $CDCl_3$ at 300 MHz.

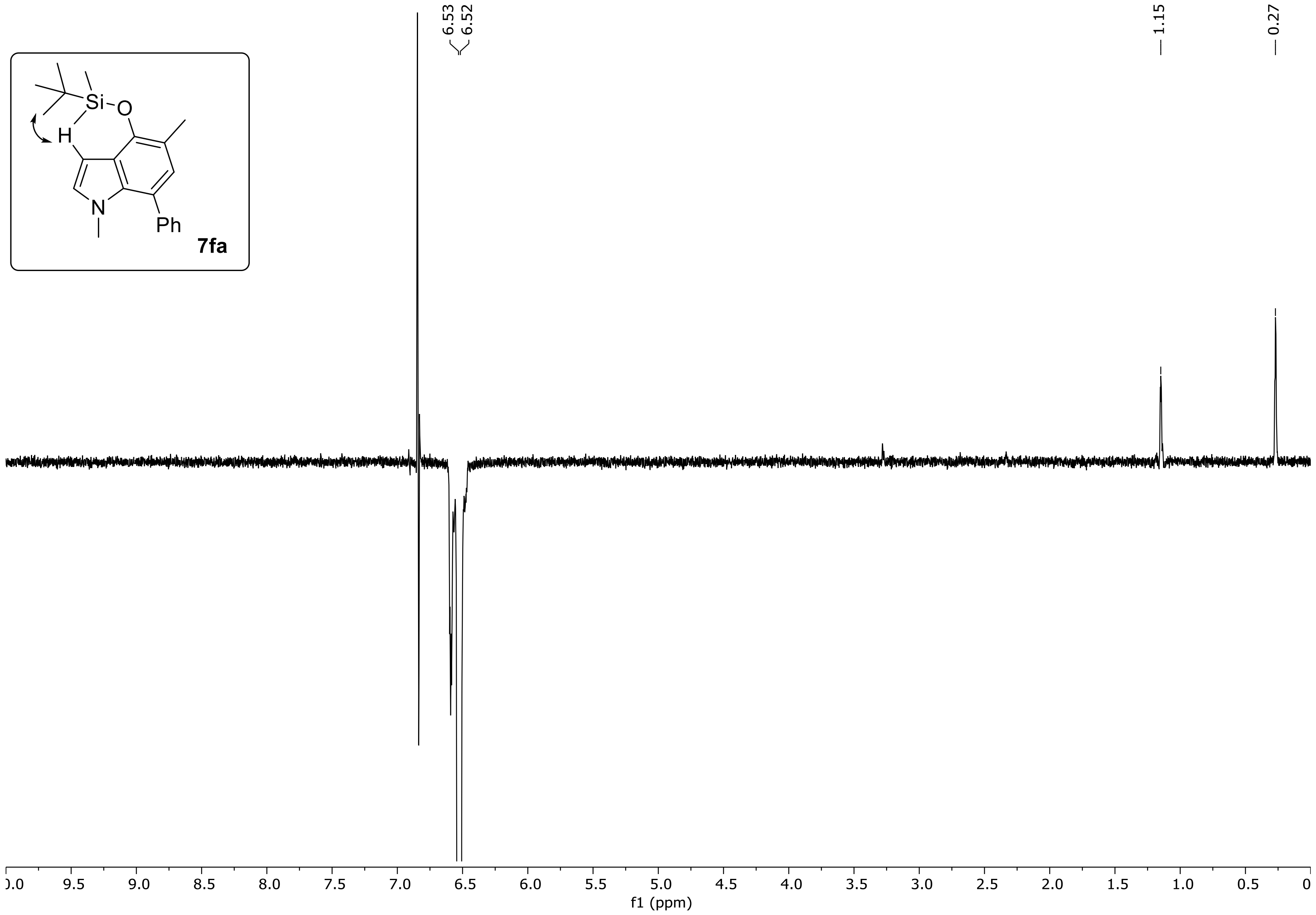

Figure S192: ^{1}H NMR of compound **7fb** in $CDCl_3$ at 300 MHz.

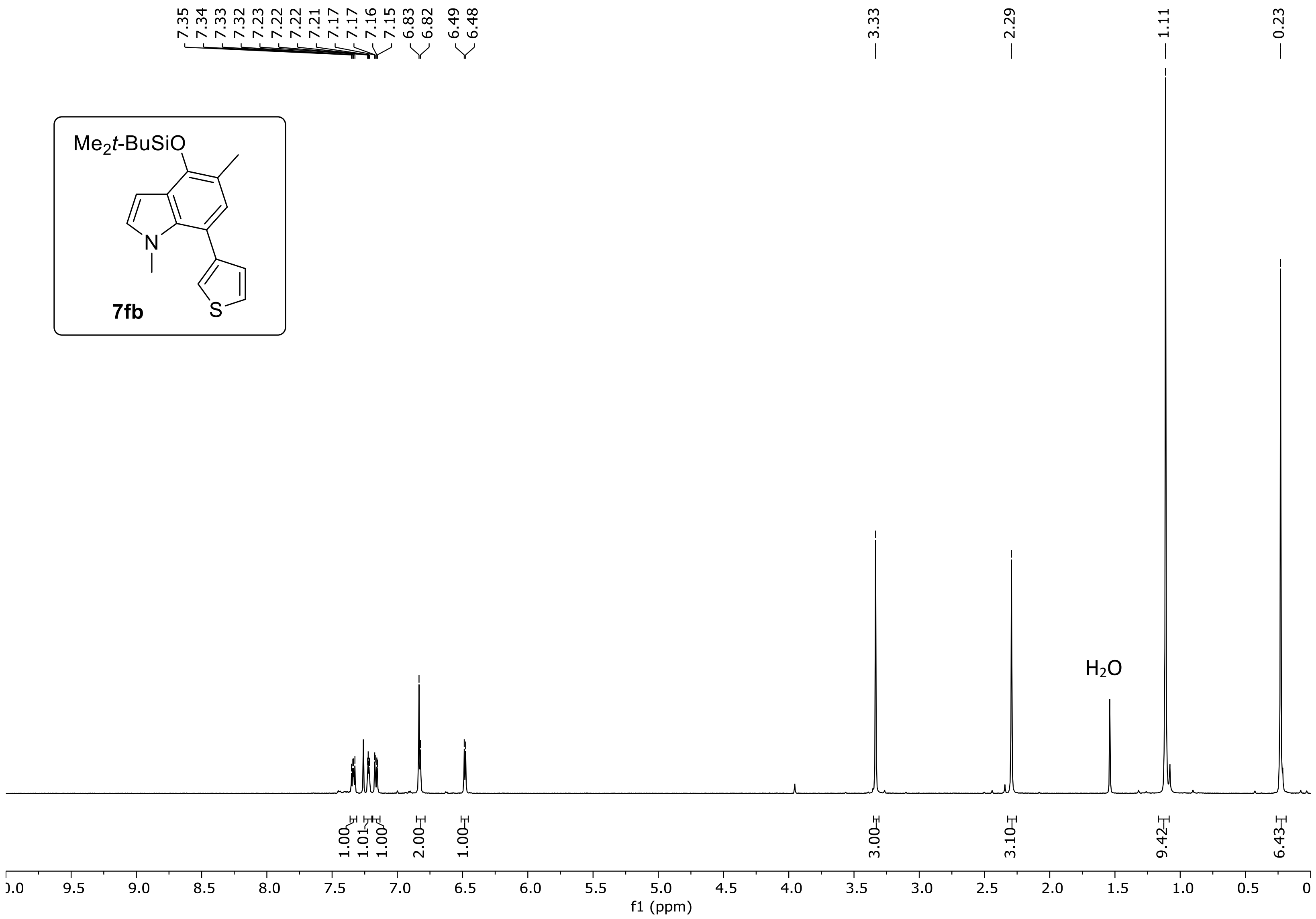

Figure S193: ^{13}C NMR of compound **7fb** in $CDCl_3$ at 75.4 MHz.

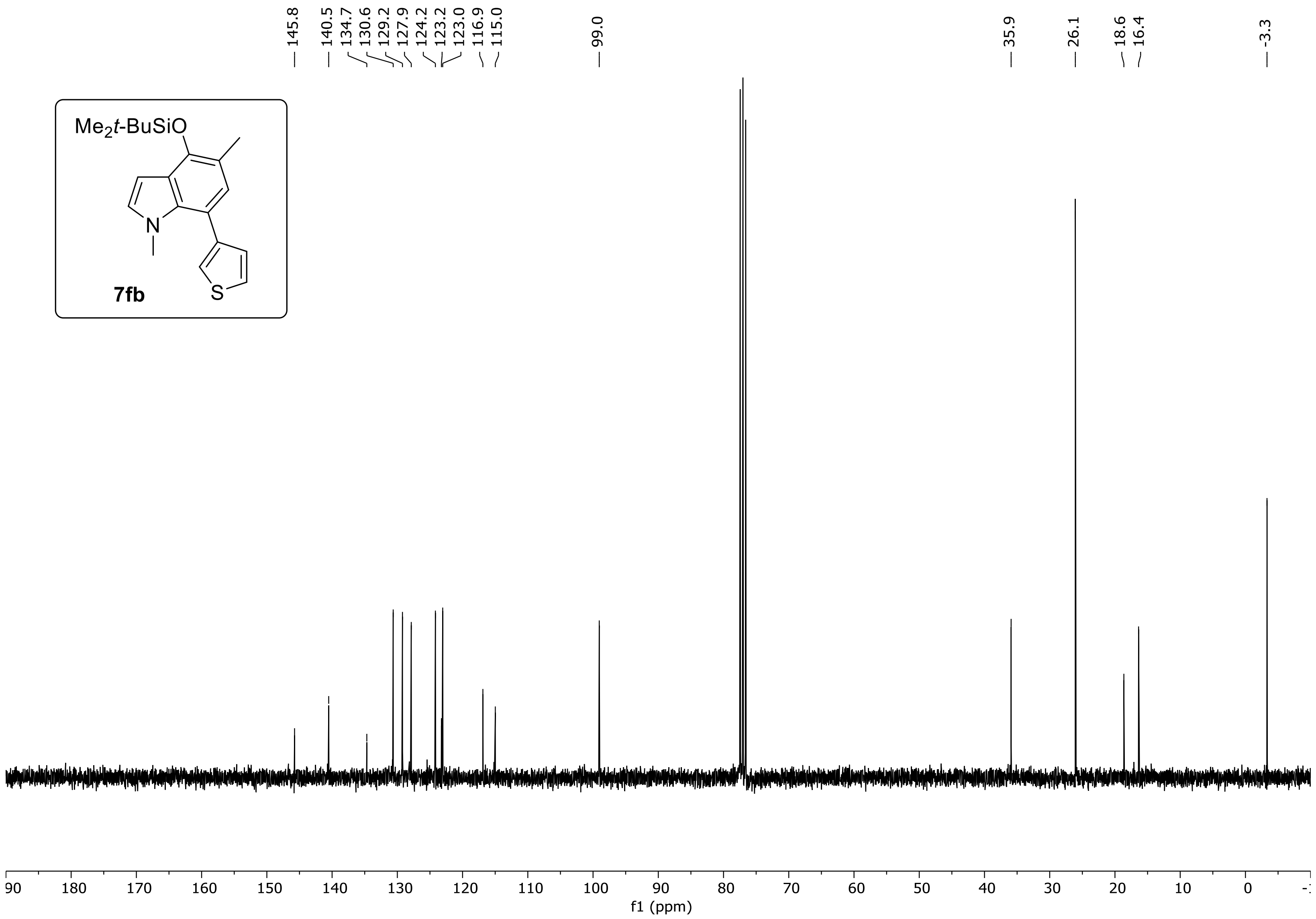

Figure S194: ^{1}H NMR of compound **7ff** in $CDCl_3$ at 300 MHz.

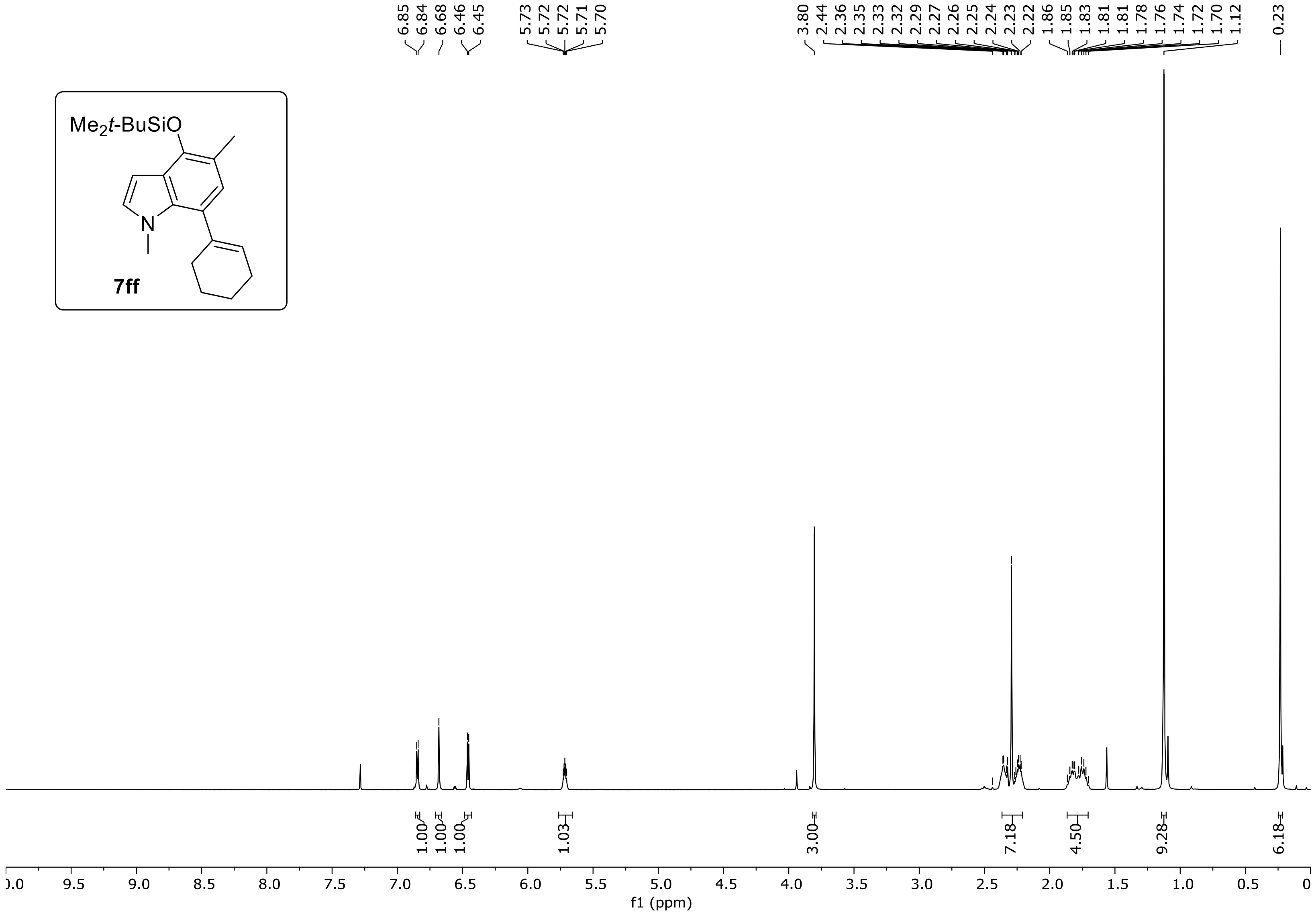

Figure S195: ^{13}C NMR of compound **7ff** in $CDCl_3$ at 75.4 MHz.

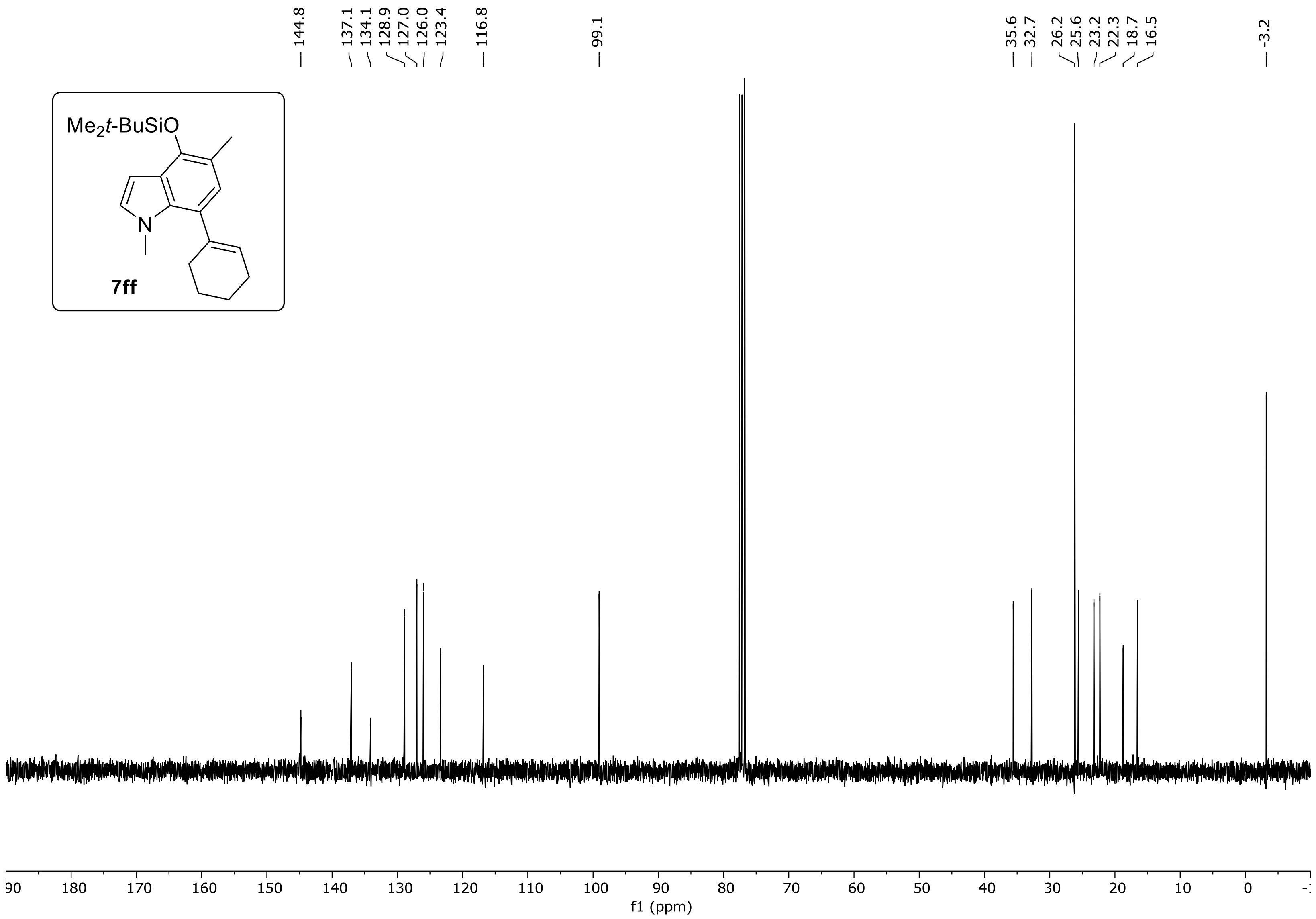

Figure S196: ^{1}H NMR of compound **3ga** in $CDCl_3$ at 300 MHz.

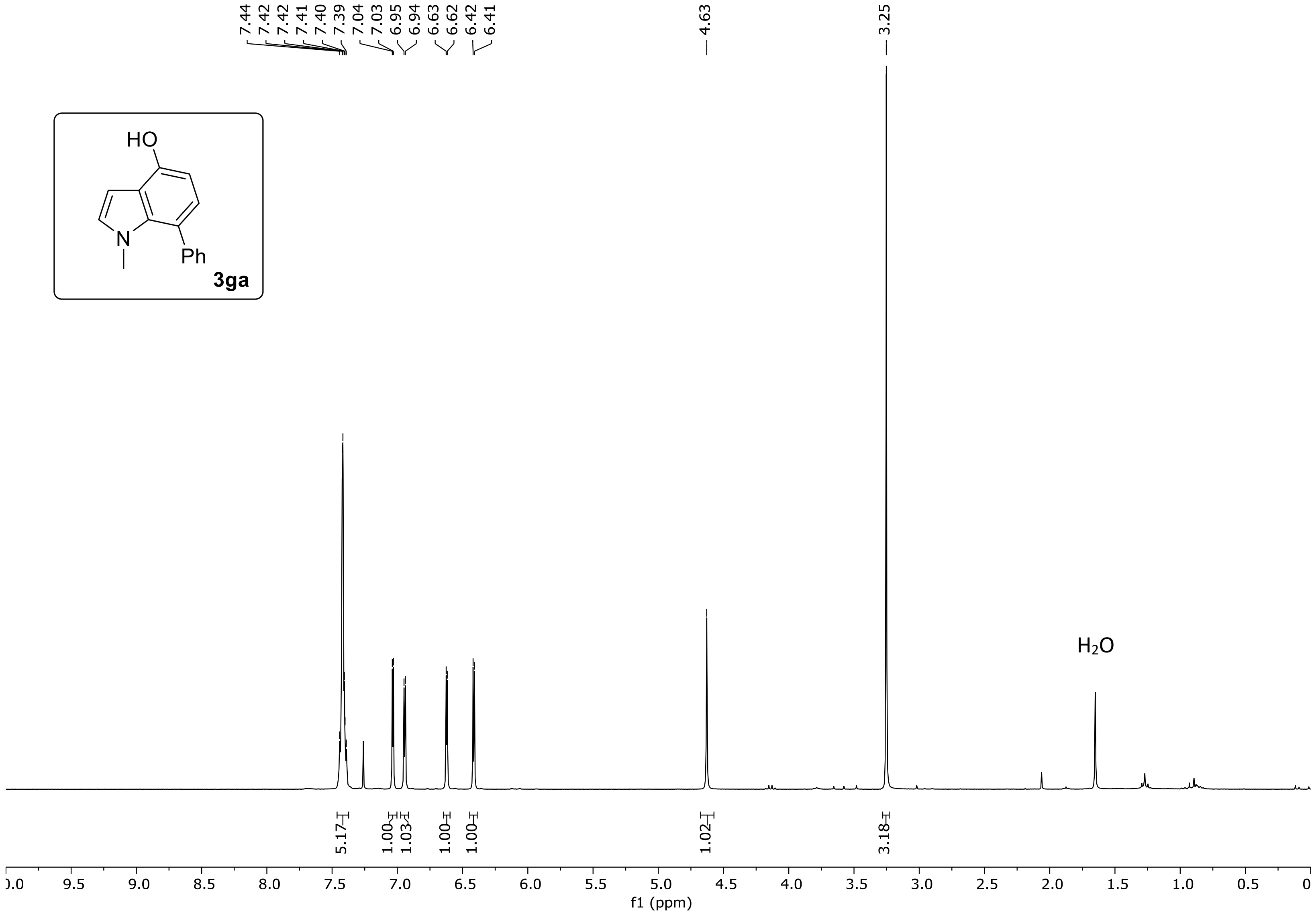

Figure S197: ^{13}C NMR of compound **3ga** in $CDCl_3$ at 75.4 MHz.

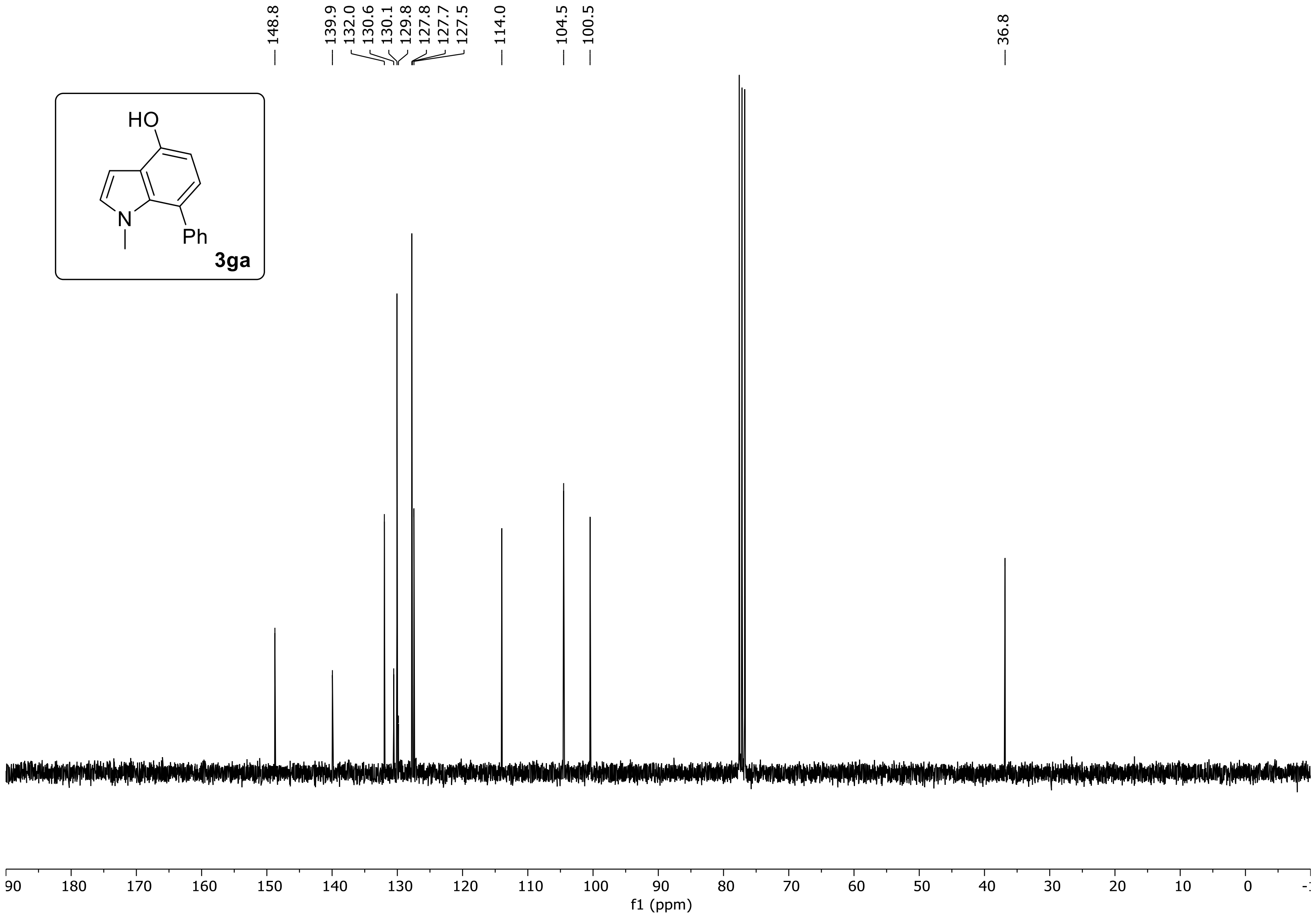

Figure S198: 1D NOE NMR of compound **3ga** in $CDCl_3$ at 300 MHz.

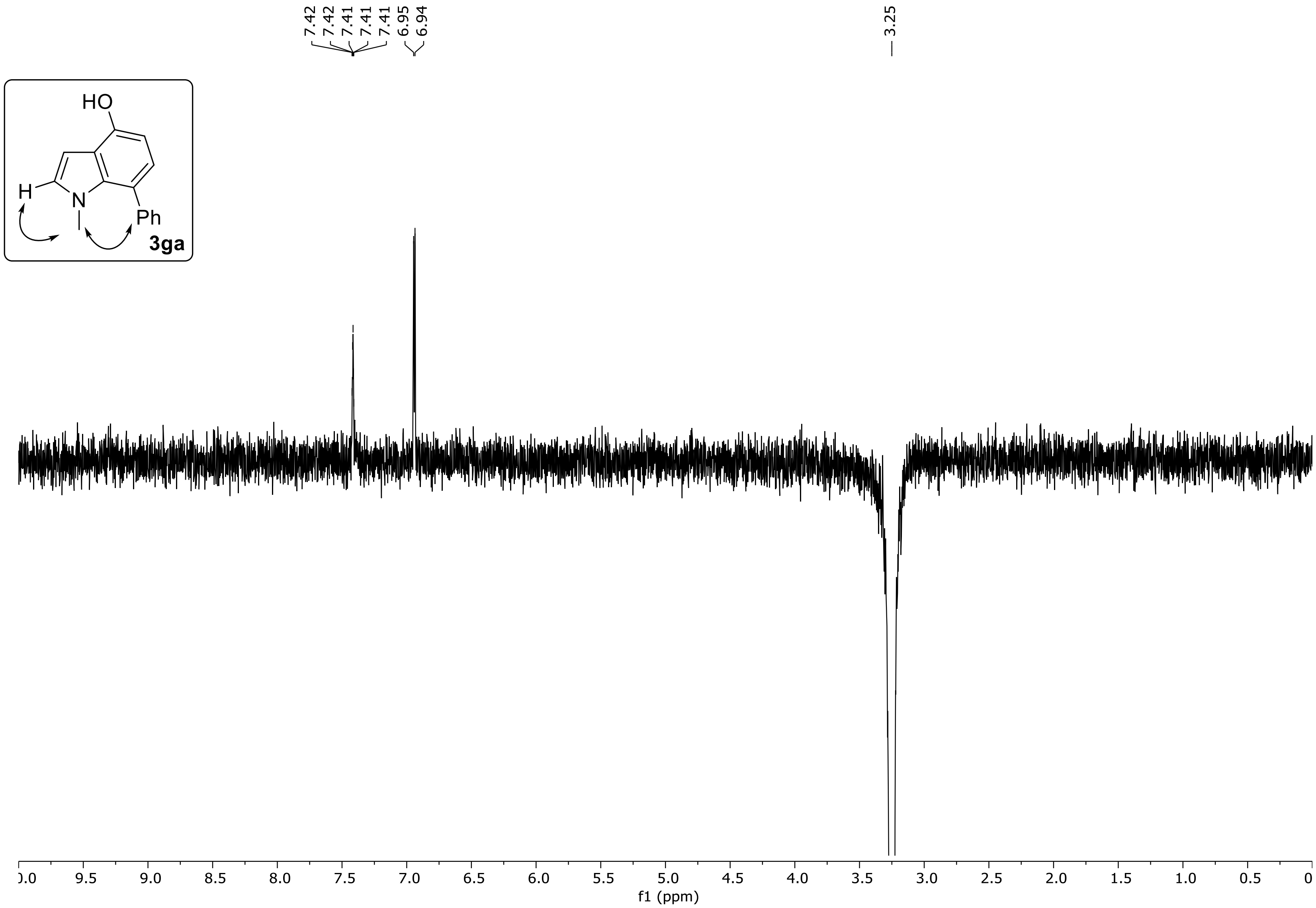

Figure S199: ^{1}H NMR of compound **3gb** in $CDCl_3$ at 300 MHz.

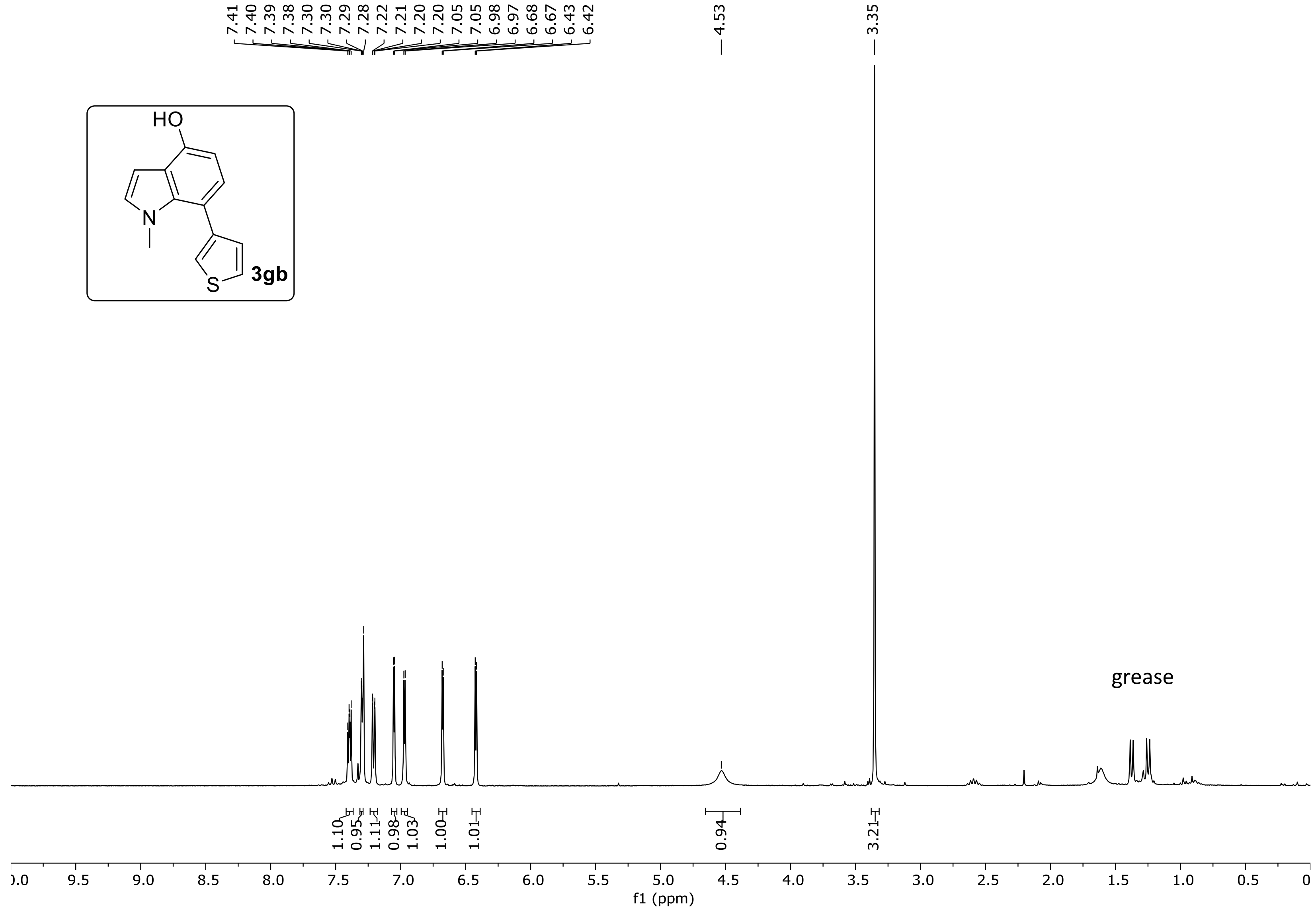

Figure S200: ^{13}C NMR of compound **3gb** in $CDCl_3$ at 75.4 MHz.

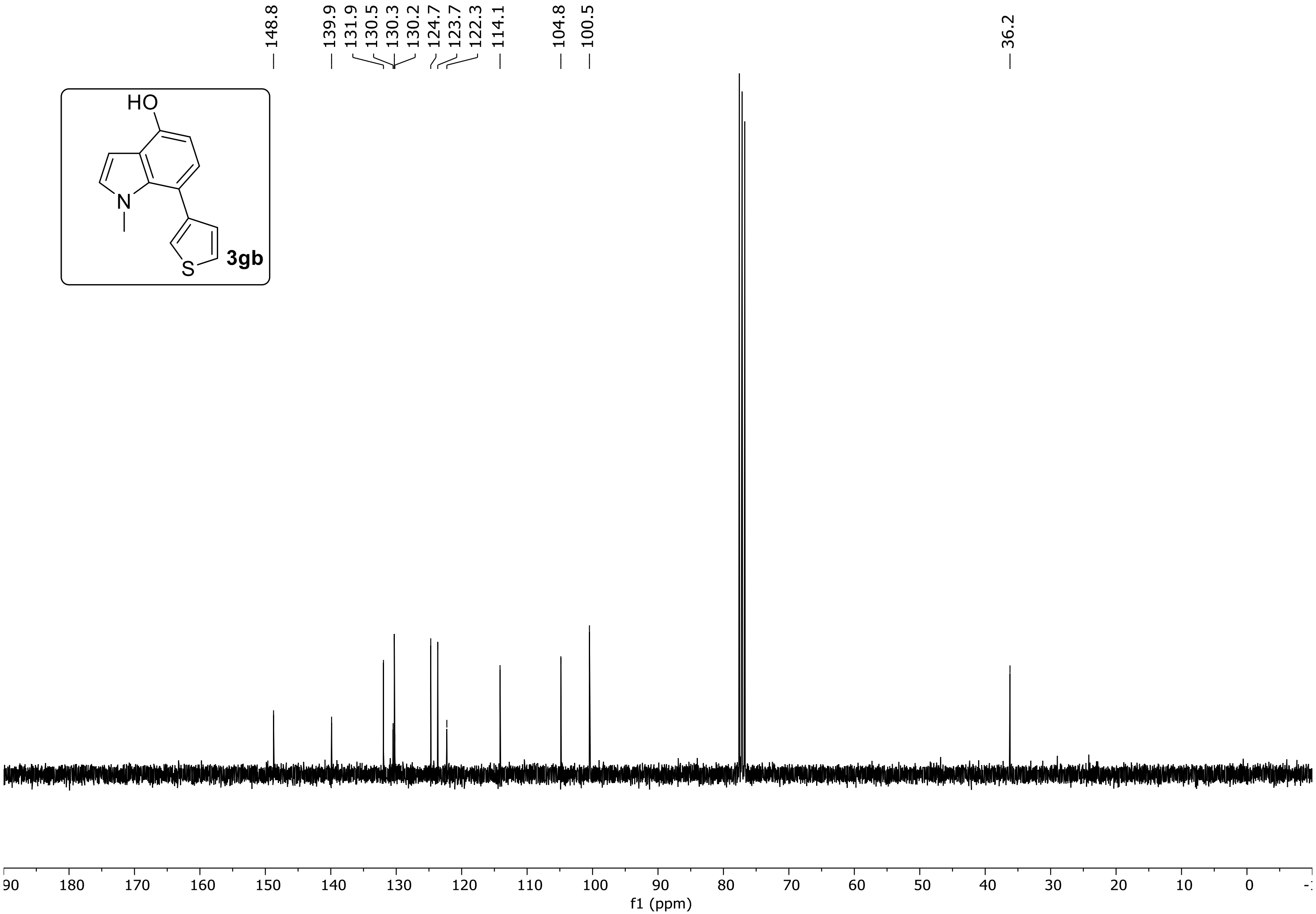

Figure S201: ^{1}H NMR of compound **7ha** in $CDCl_3$ at 300 MHz.

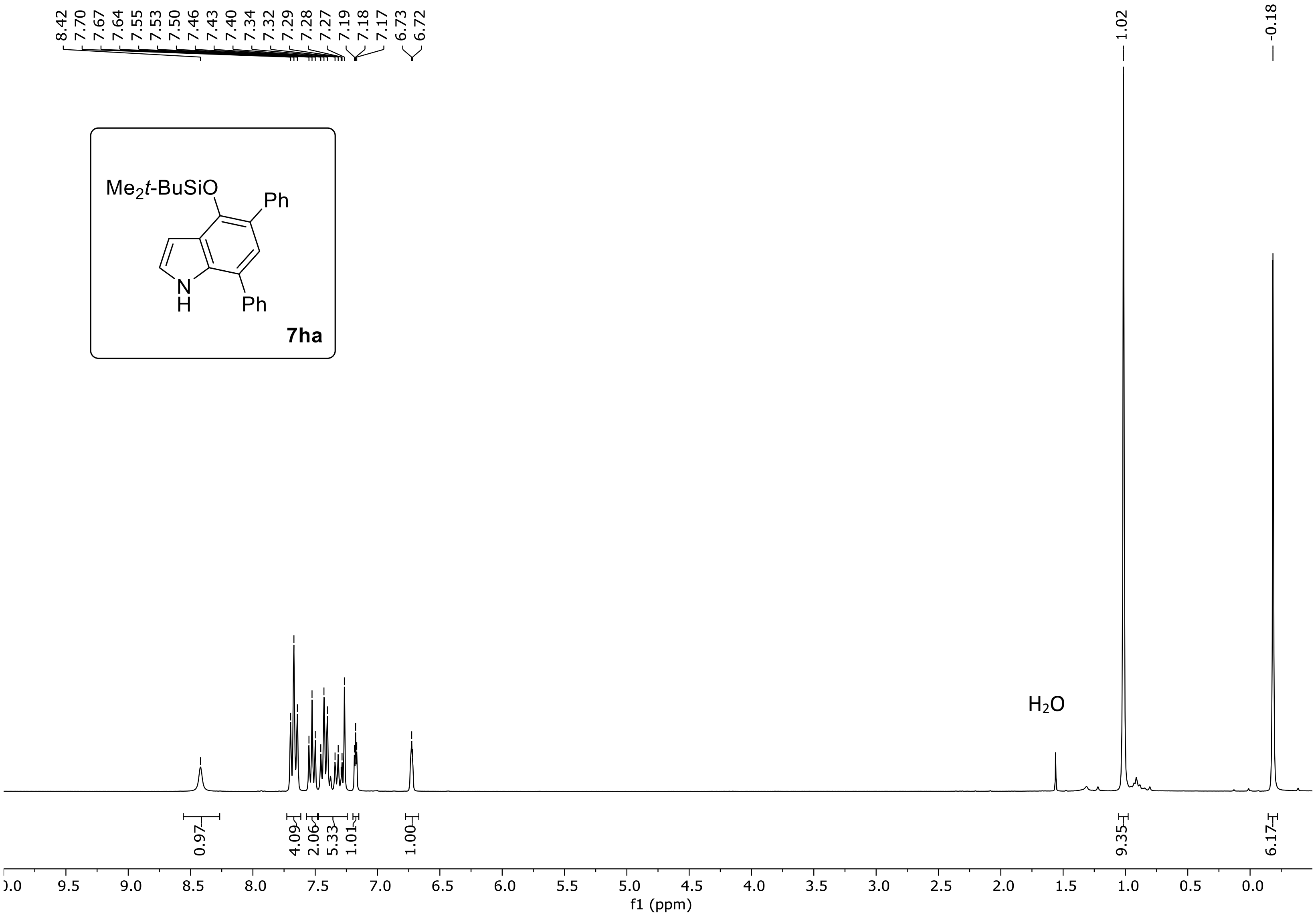

Figure S202: ^{13}C NMR of compound **7ha** in $CDCl_3$ at 75.4 MHz.

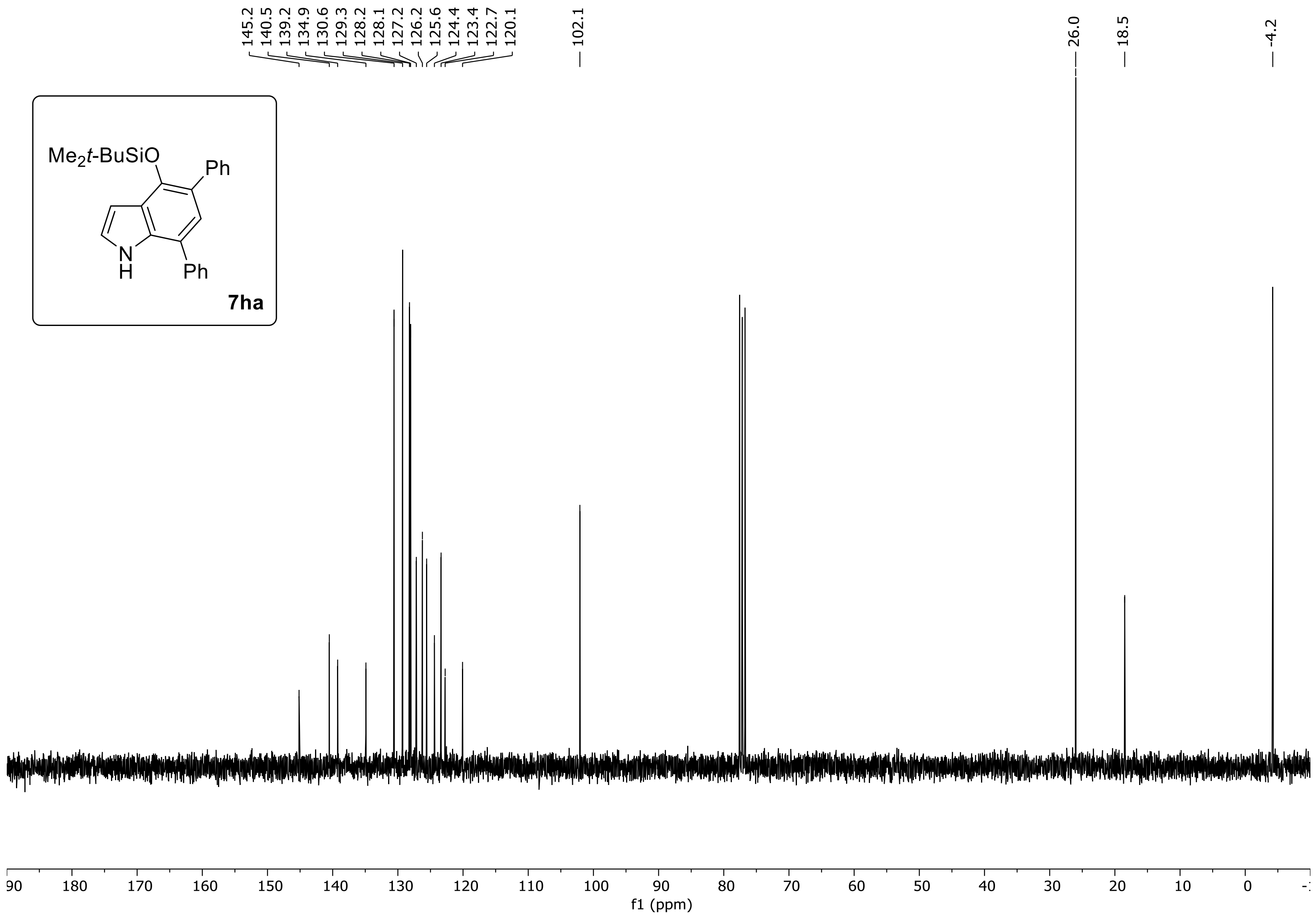

Figure S203: 1D NOE NMR of compound **7ha** in $CDCl_3$ at 300 MHz.

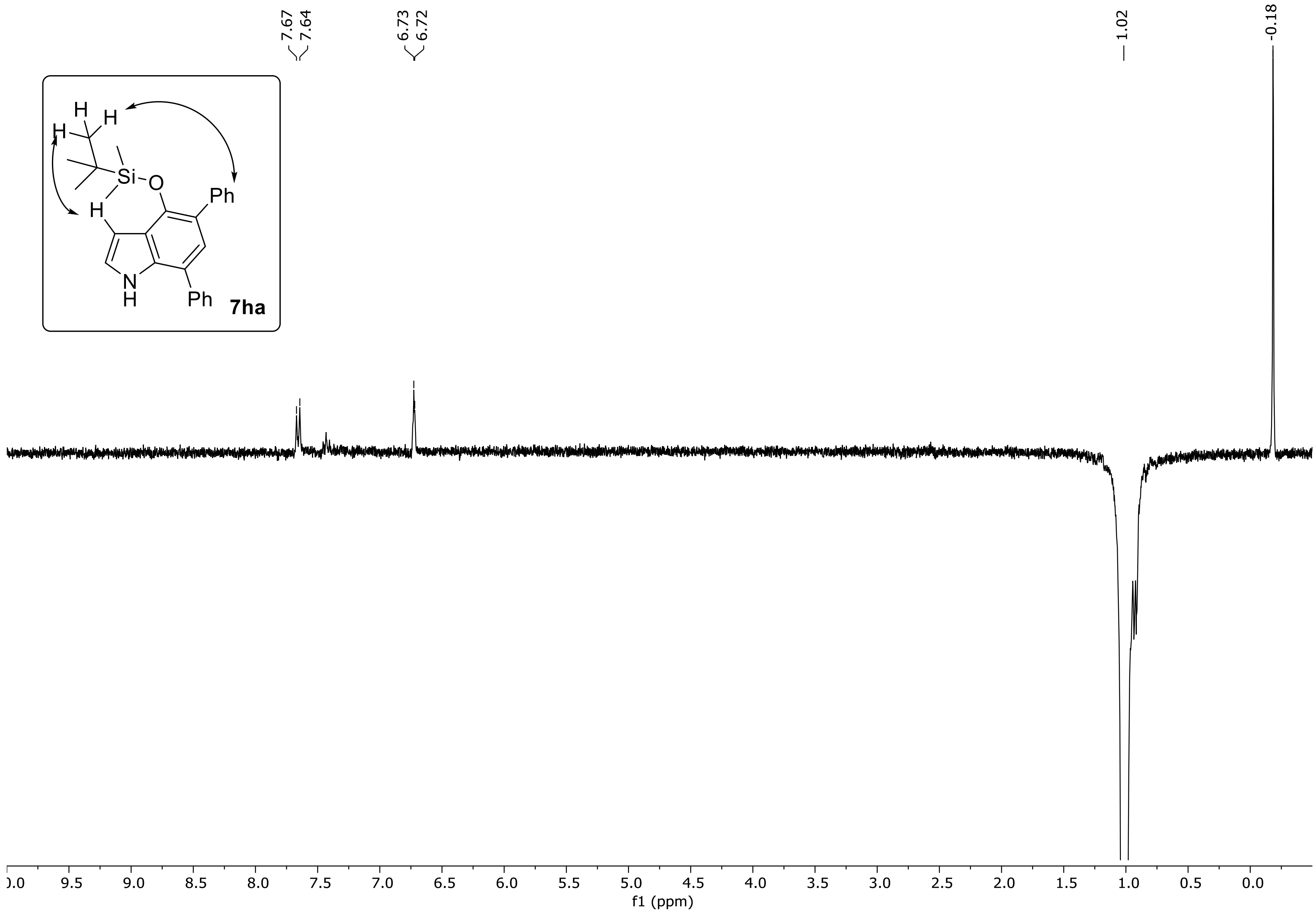

Figure S204: ^{1}H NMR of compound **7hb** in $CDCl_3$ at 300 MHz.

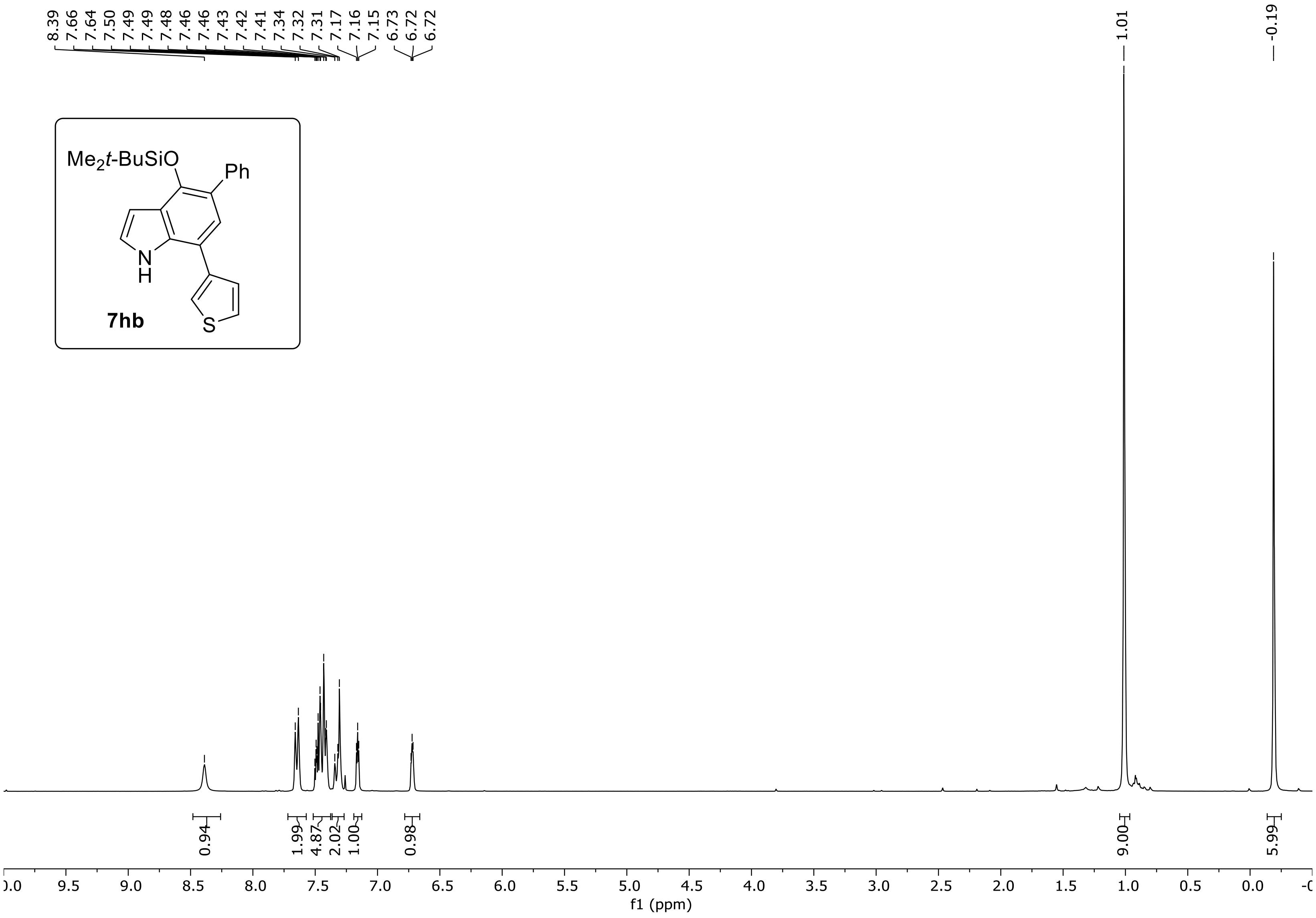

Figure S205: ^{13}C NMR of compound **7hb** in $CDCl_3$ at 75.4 MHz.

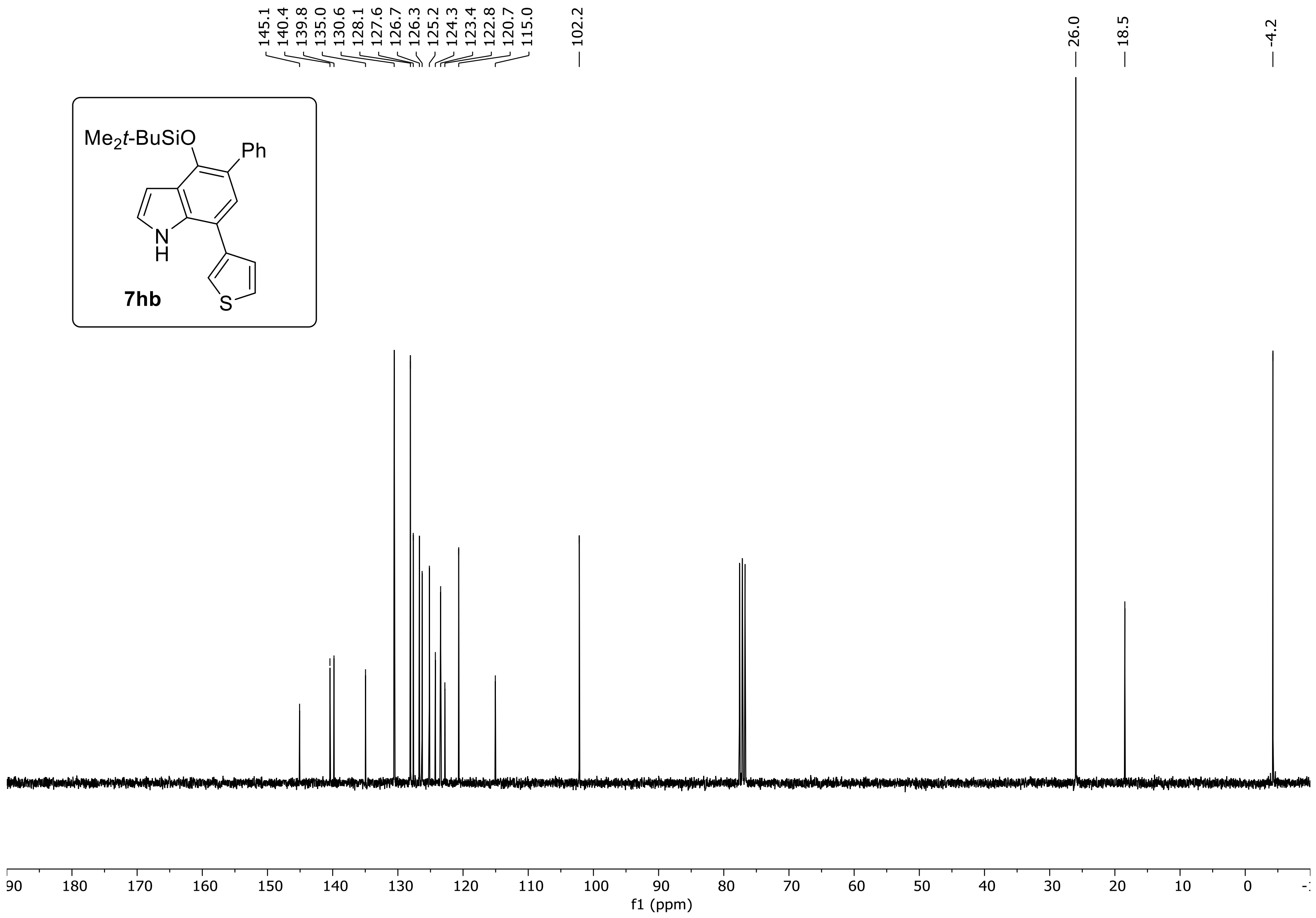

Figure S206: 1D NOE NMR of compound **7hb** in $CDCl_3$ at 300 MHz.

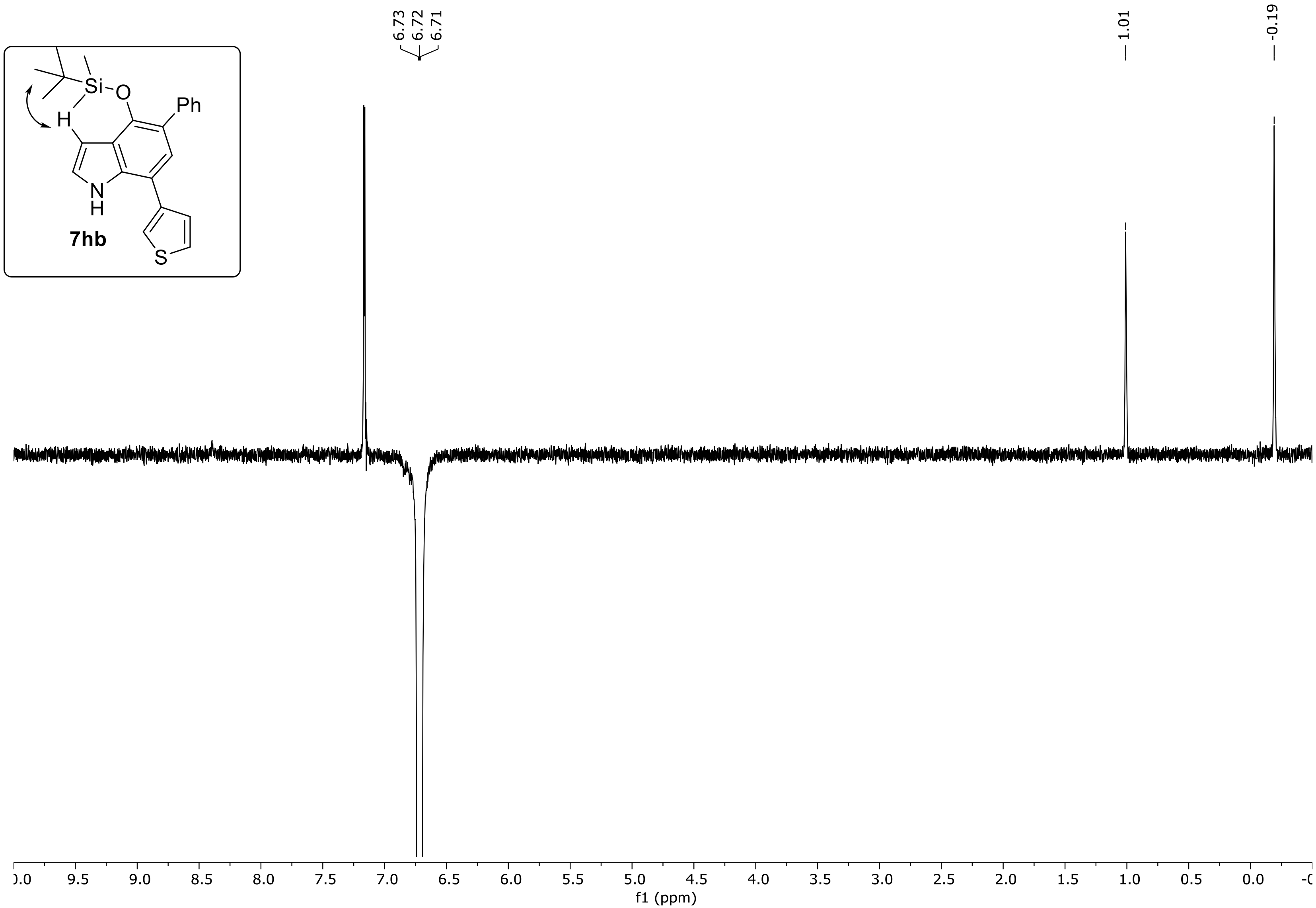

Figure S207: ^{1}H NMR of compound **7hh** in $CDCl_3$ at 300 MHz.

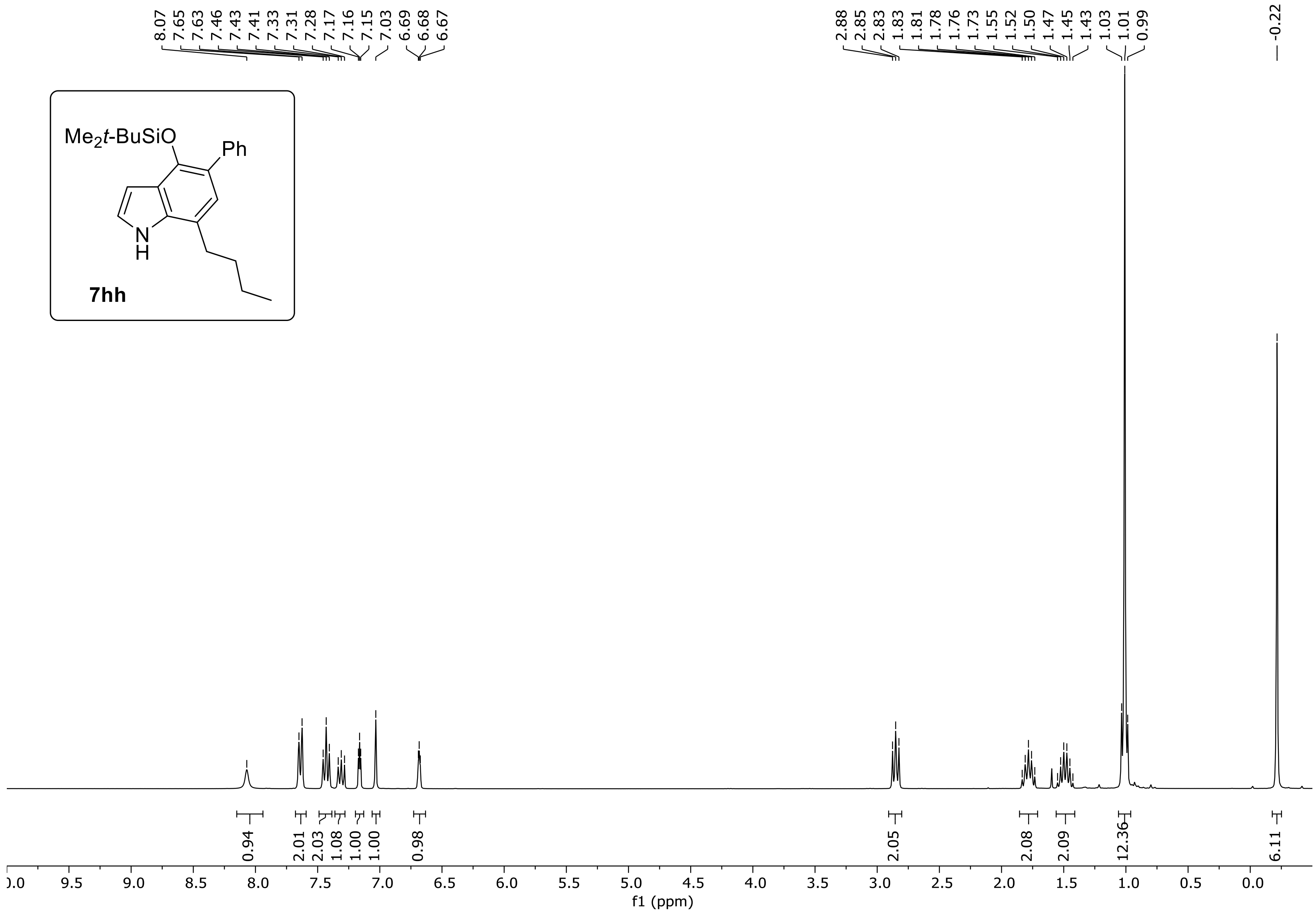

Figure S208: ^{13}C NMR of compound **7hh** in $CDCl_3$ at 75.4 MHz.

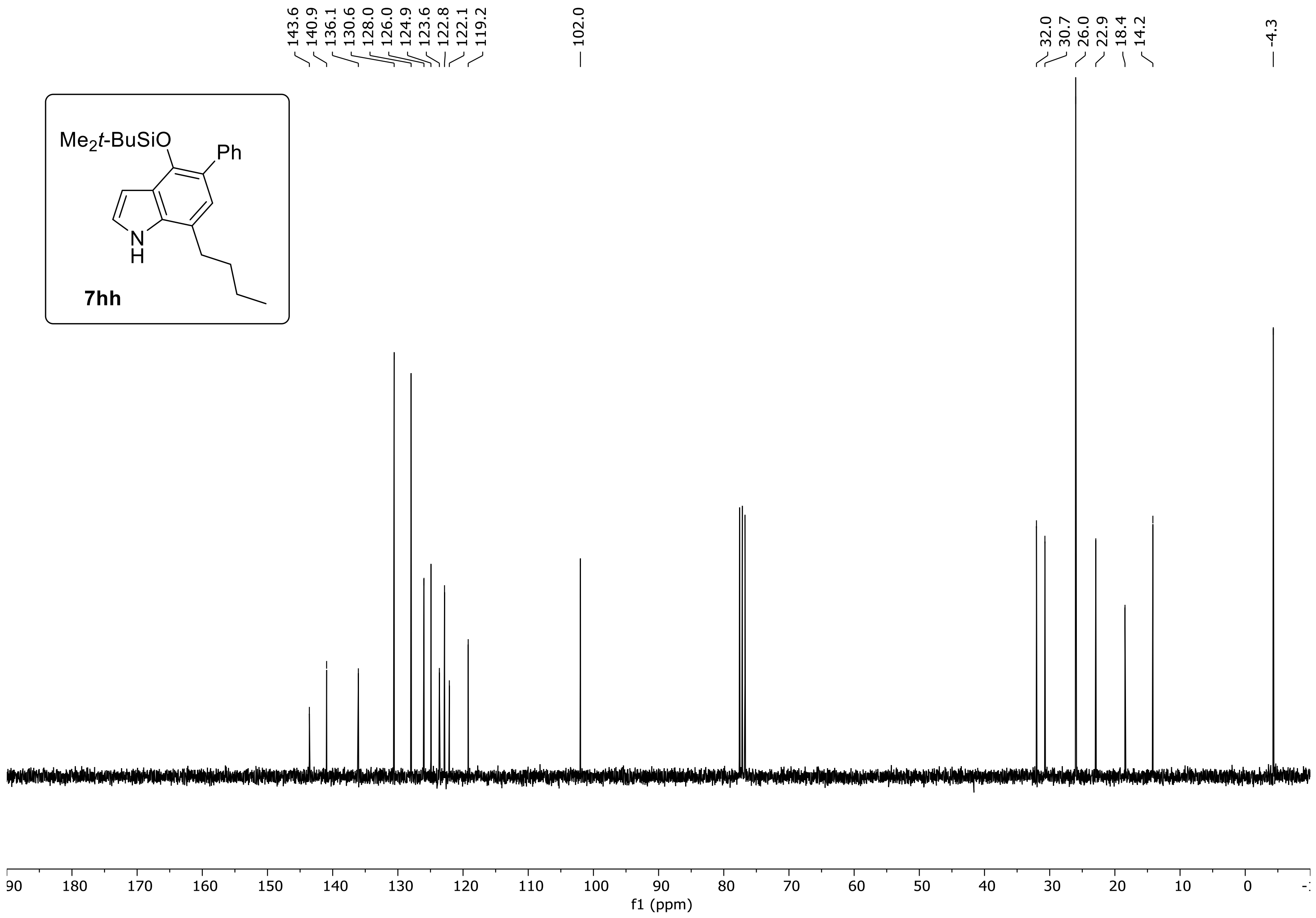

Figure S209: 1D NOE NMR of compound **7hh** in $CDCl_3$ at 300 MHz.

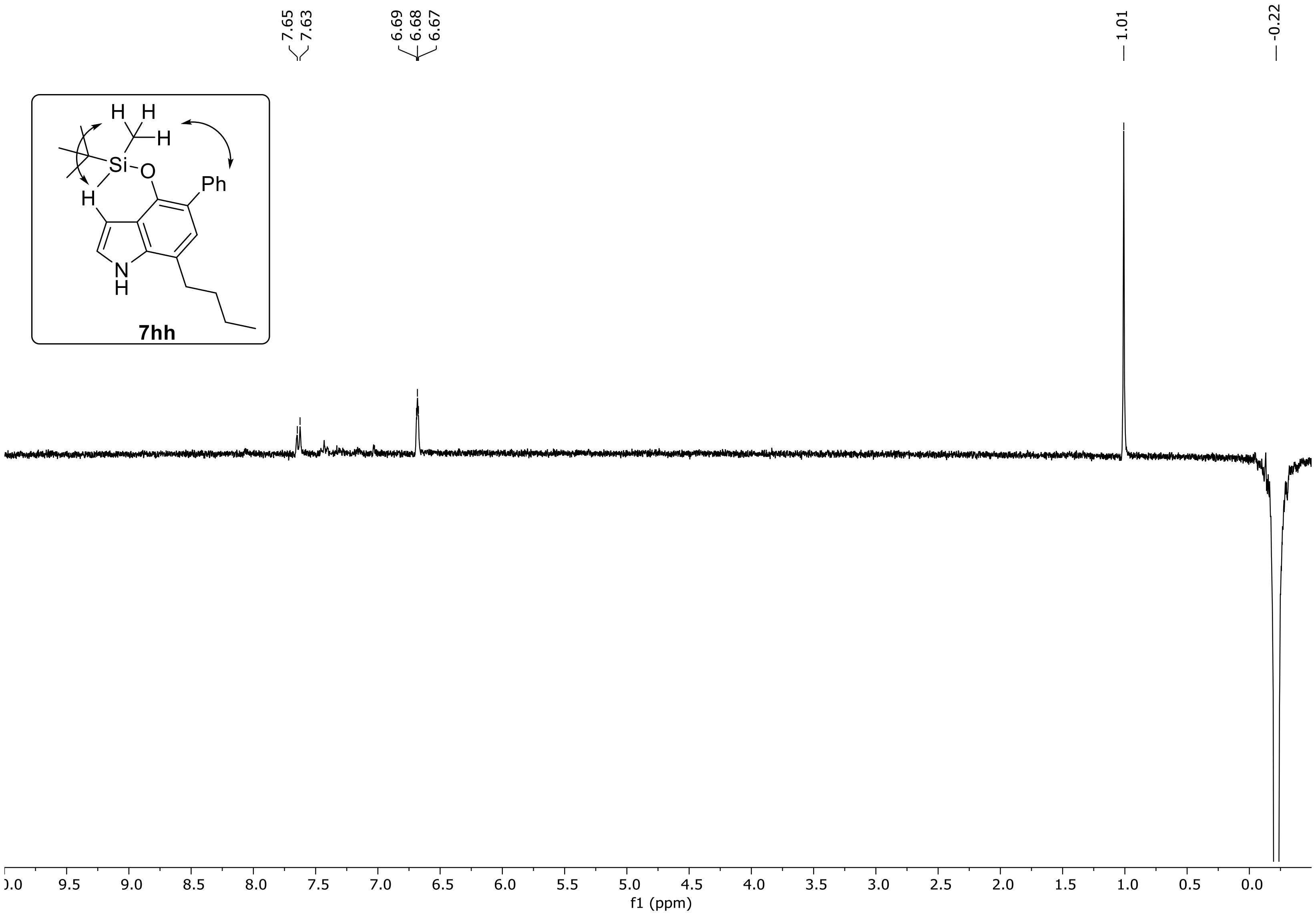

Figure S210: ^{1}H NMR of compound **7ia** in $CDCl_3$ at 300 MHz.

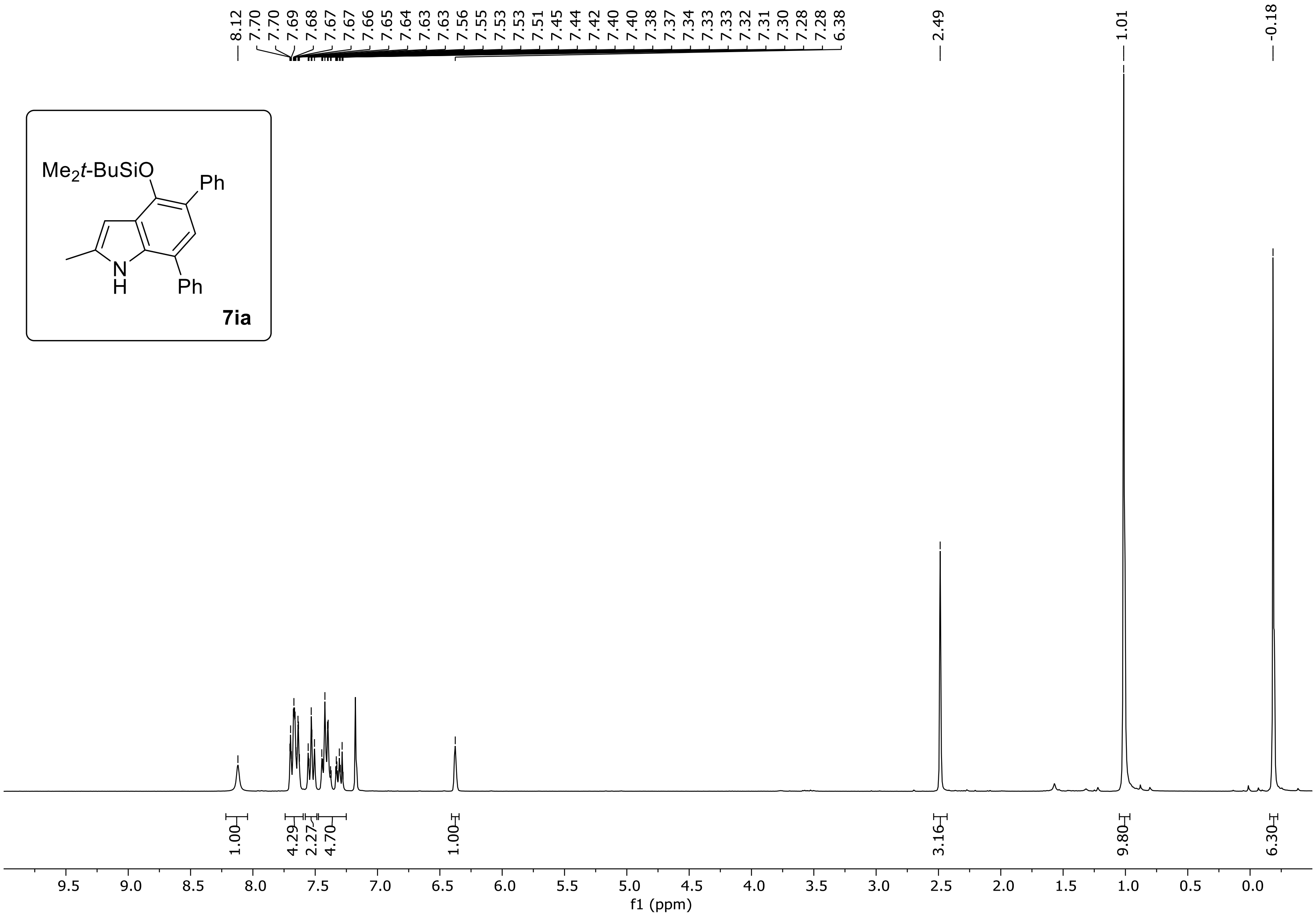

Figure S211: ^{13}C NMR of compound **7ia** in $CDCl_3$ at 75.4 MHz.

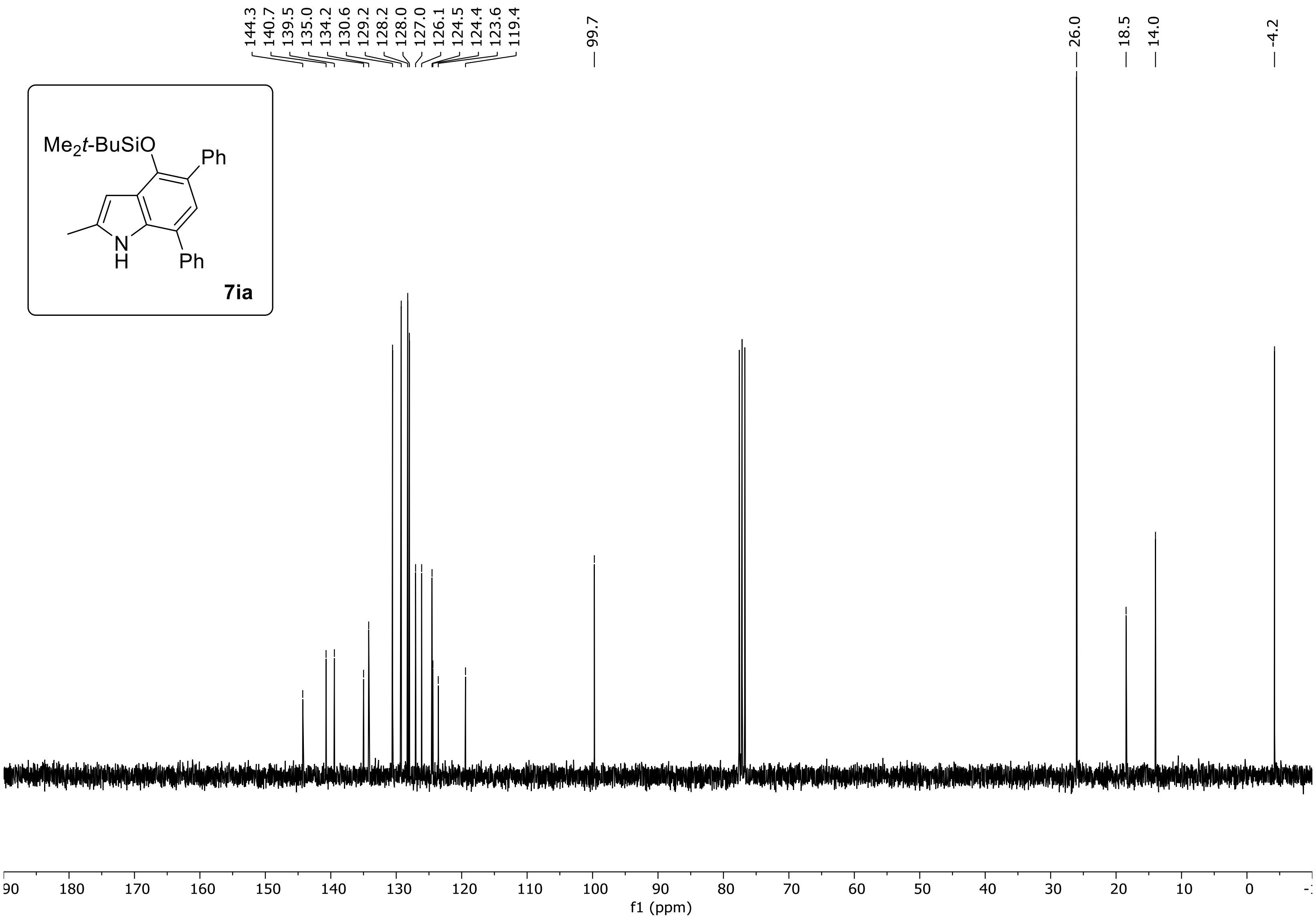

Figure S212: 1D NOE NMR of compound **7ia** in $CDCl_3$ at 300 MHz.

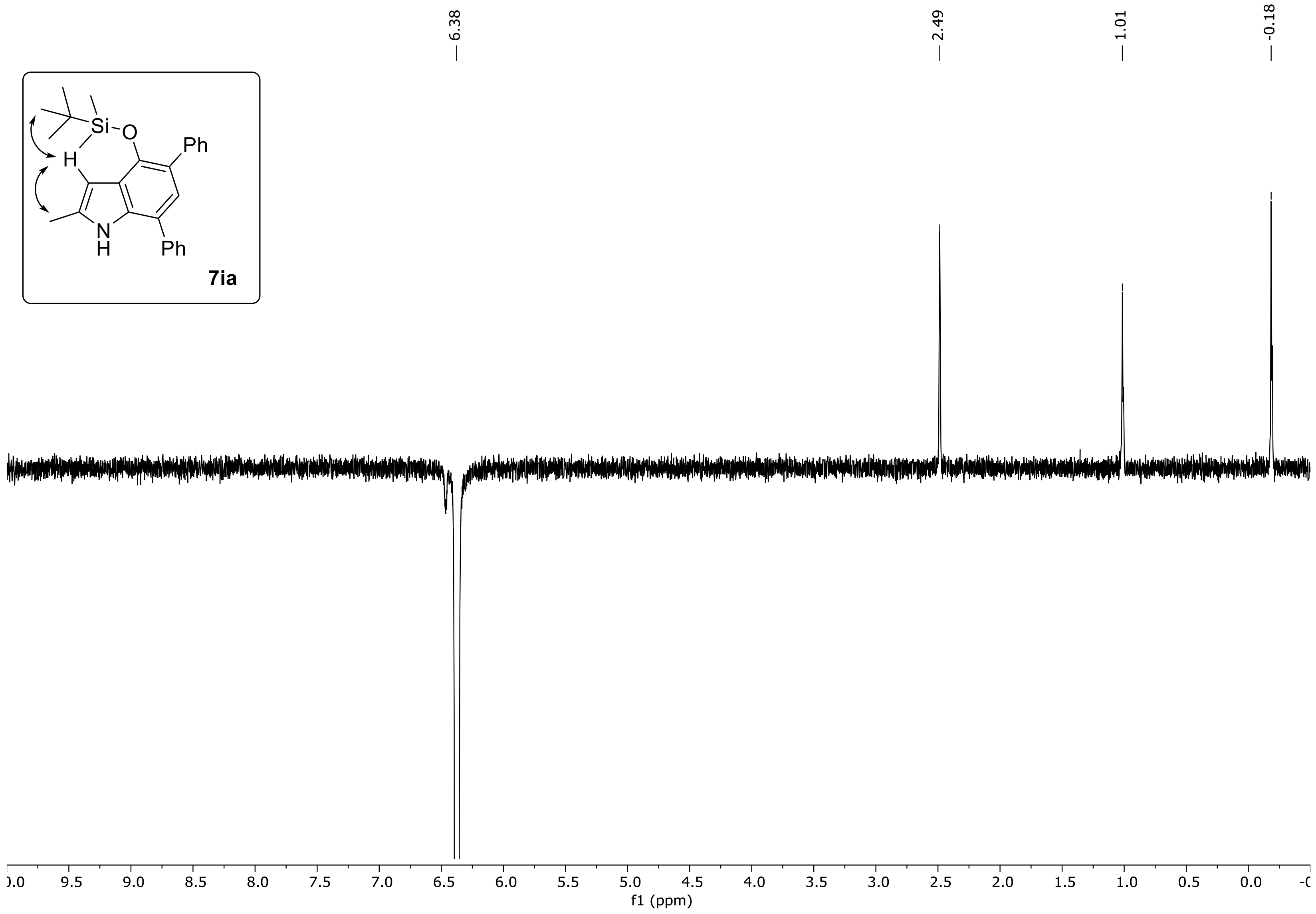

Figure S213: ^{1}H NMR of compound **7ib** in $CDCl_3$ at 300 MHz.

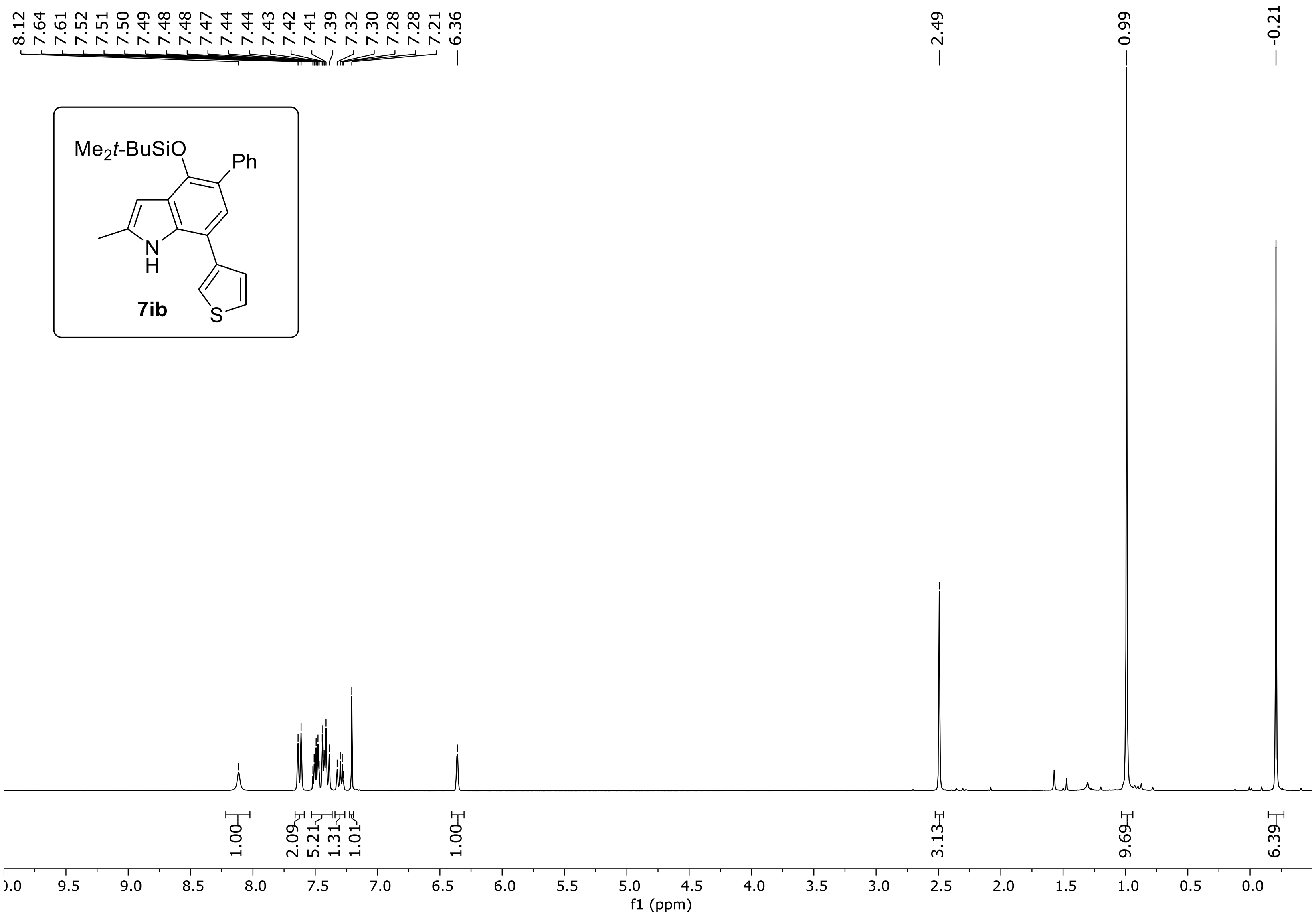

Figure S214: ^{13}C NMR of compound **7ib** in $CDCl_3$ at 75.4 MHz.

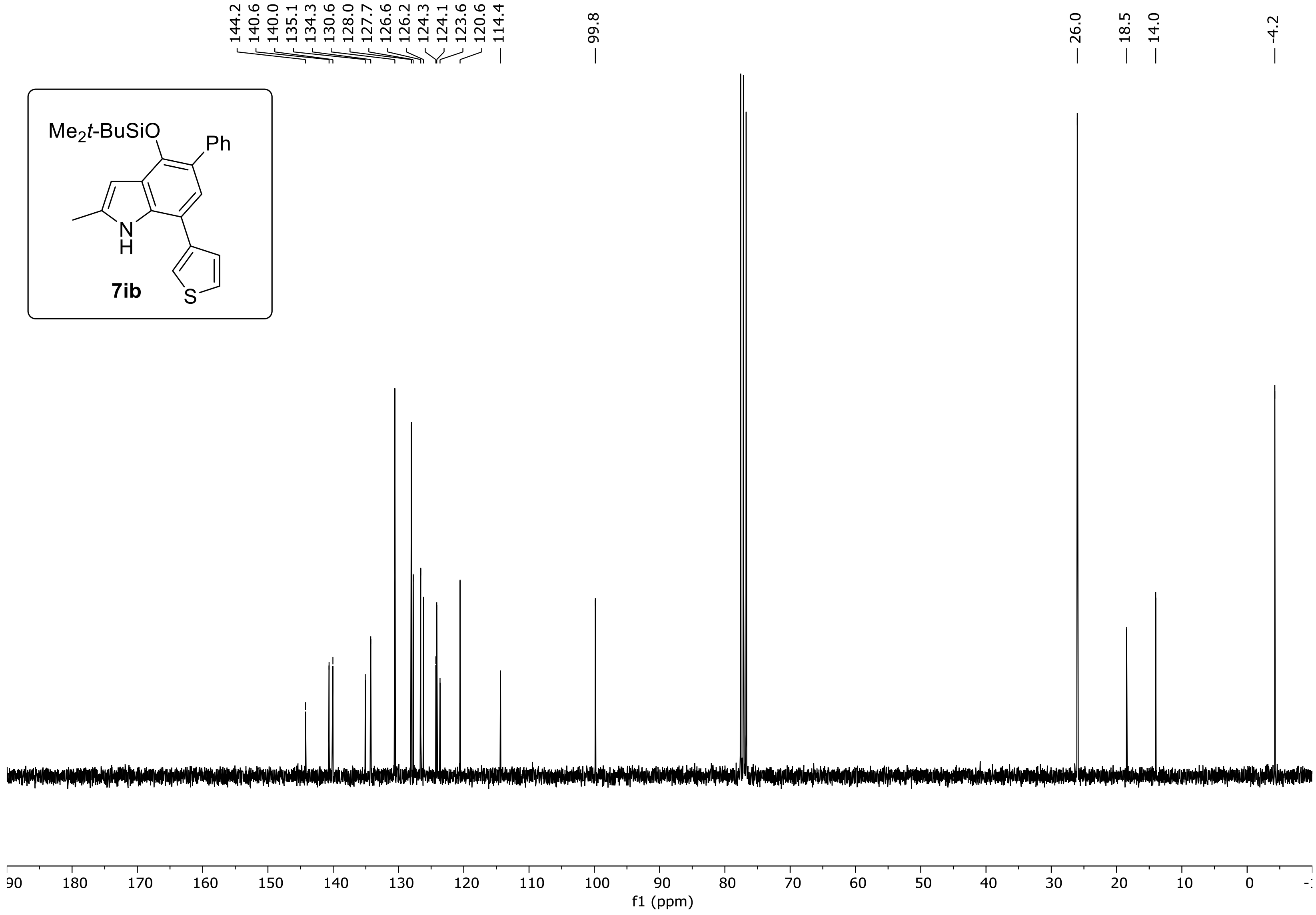

Figure S215: ^{1}H NMR of compound **7ja** in $CDCl_3$ at 300 MHz.

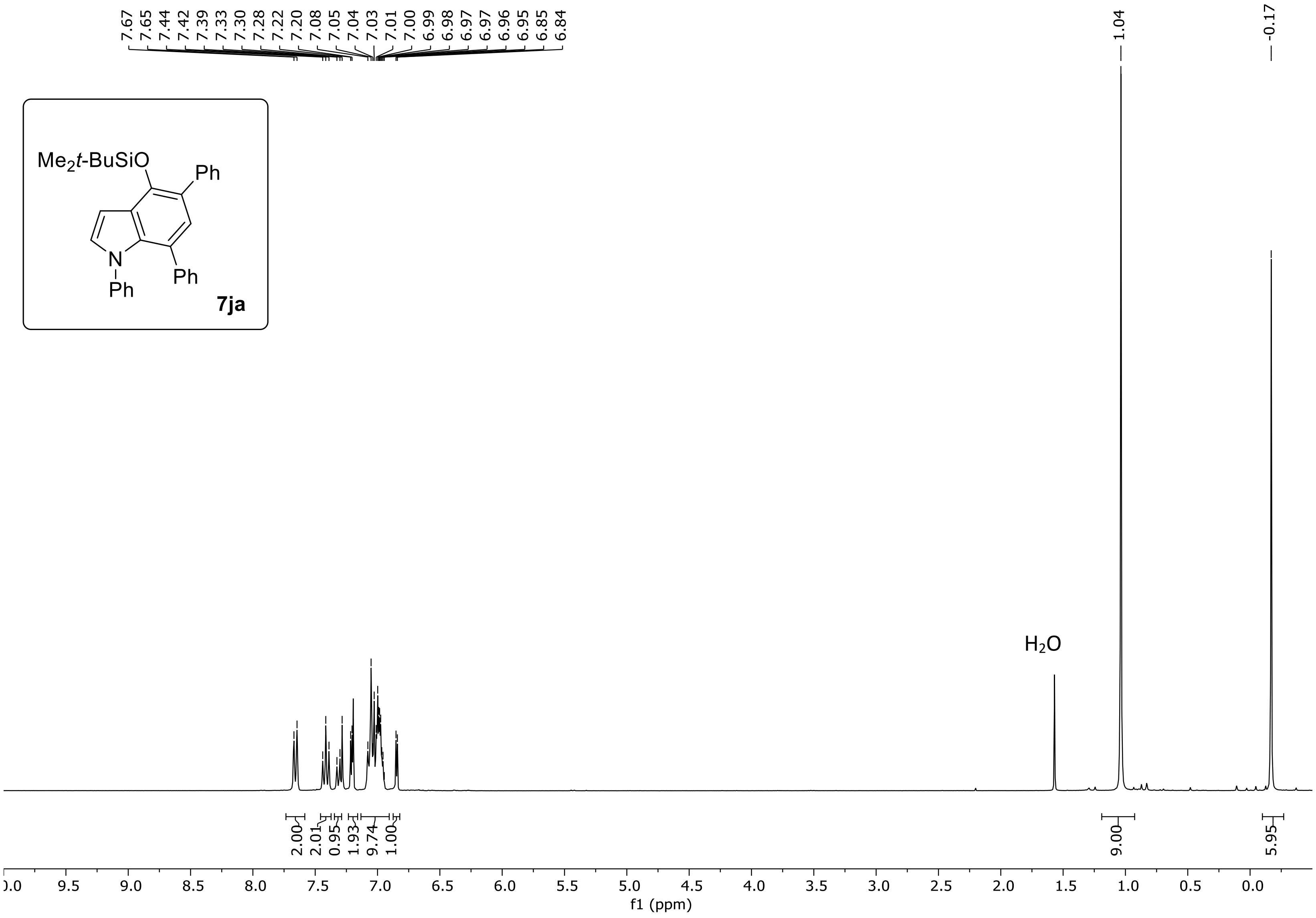

Figure S216: ^{13}C NMR of compound **7ja** in $CDCl_3$ at 75.4 MHz.

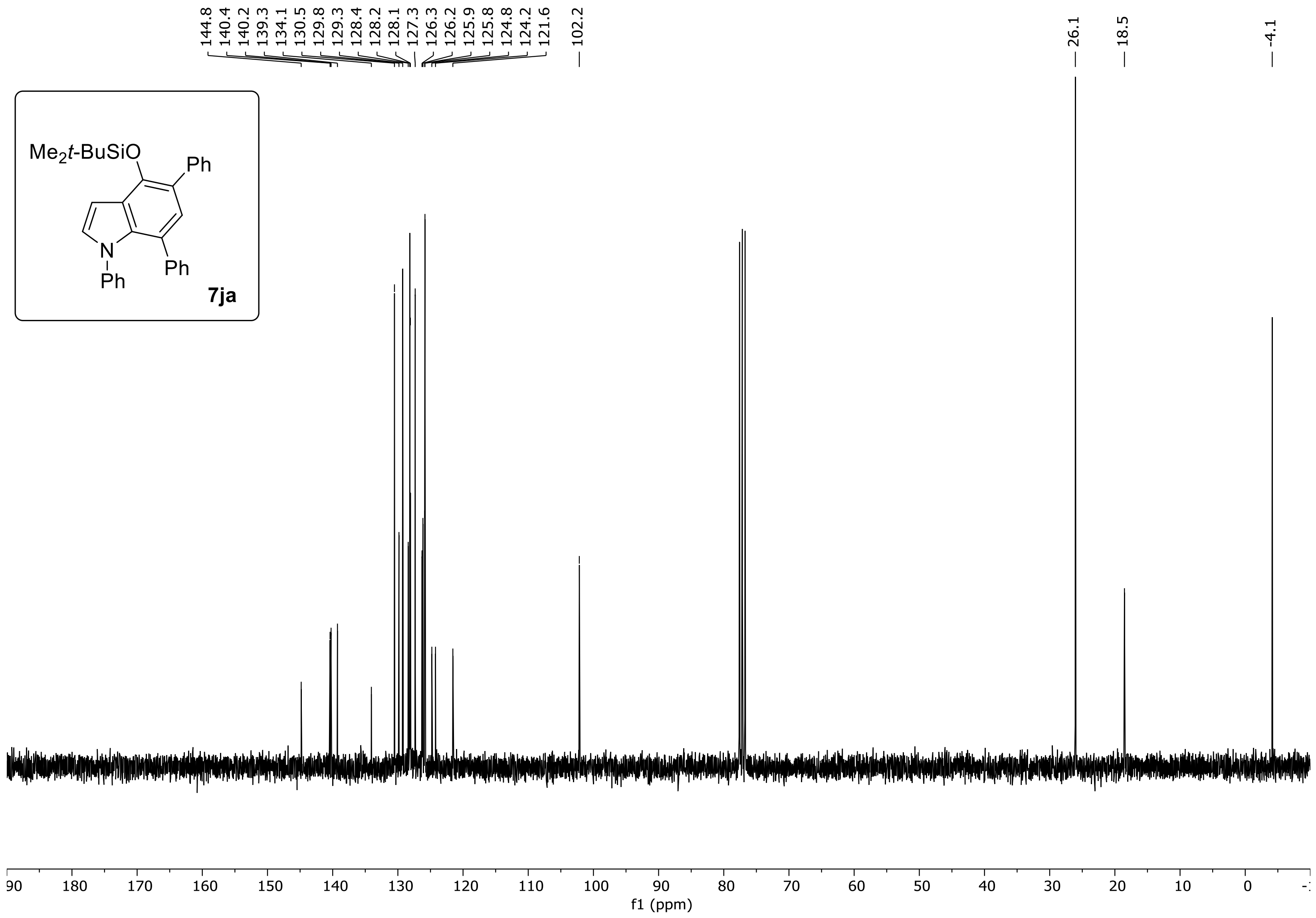

Figure S217: 1D NOE NMR of compound **7ja** in $CDCl_3$ at 300 MHz.

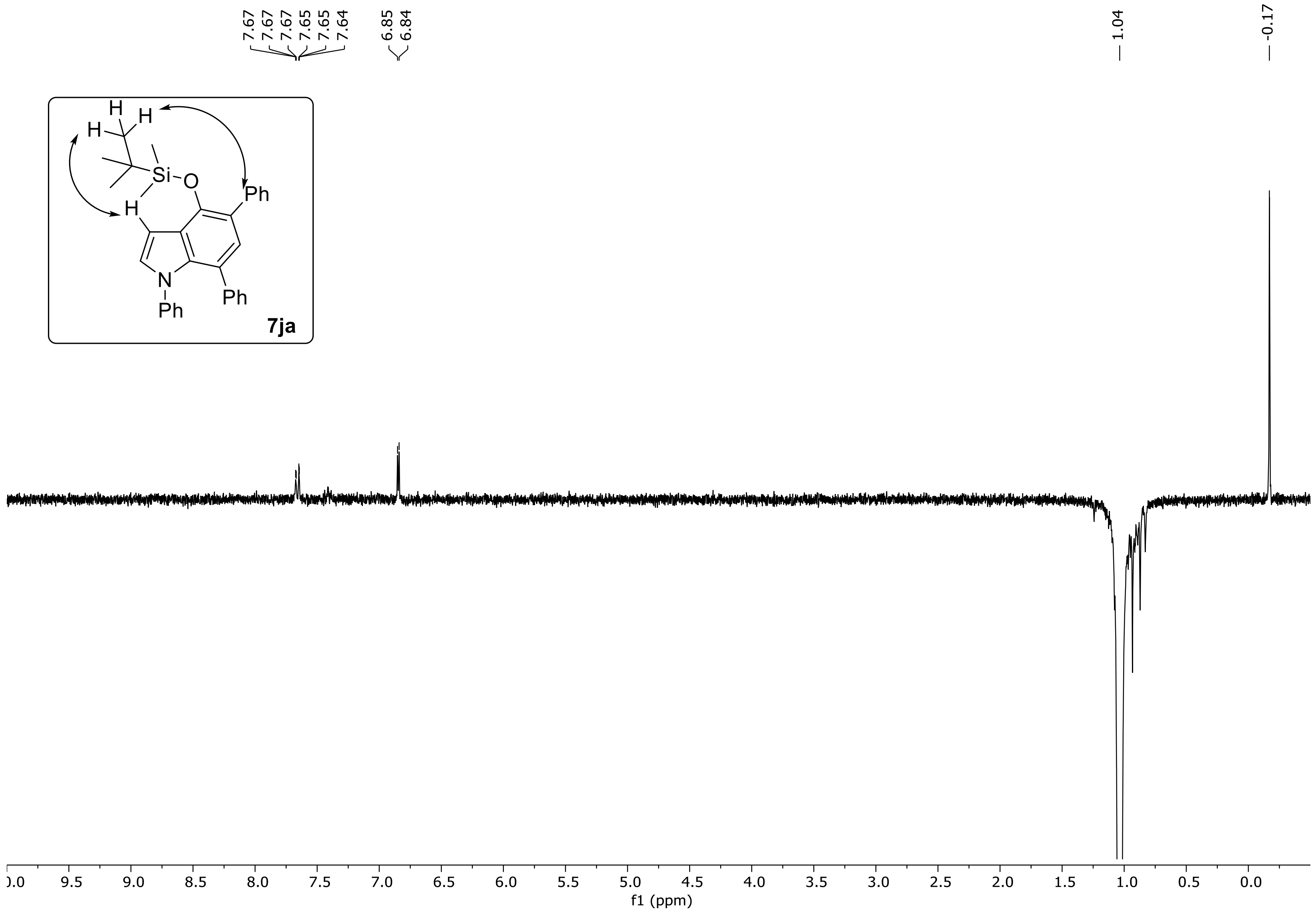

Figure S218: ^{1}H NMR of compound **7jh** in $CDCl_3$ at 300 MHz.

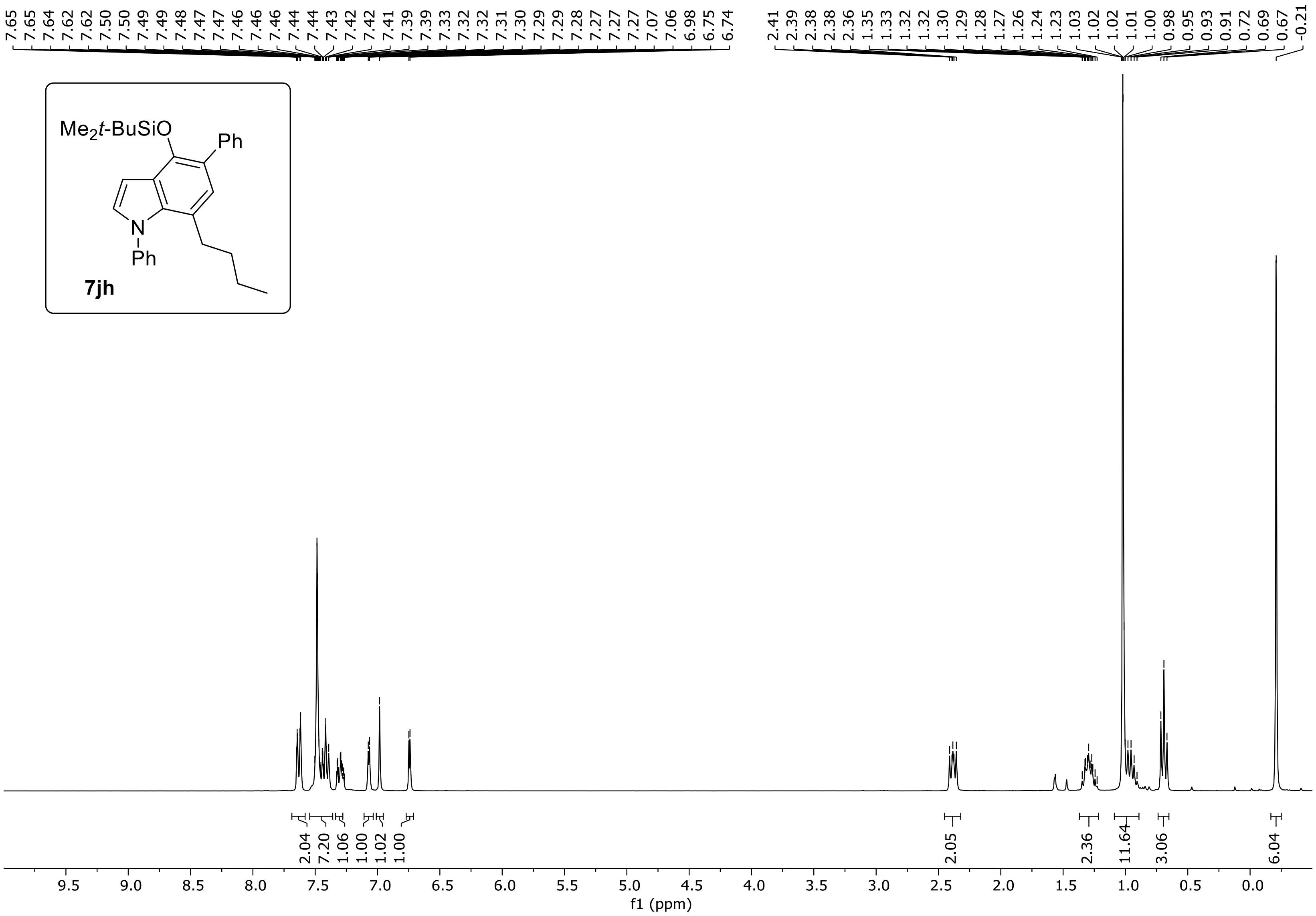

Figure S219: ^{13}C NMR of compound **7jh** in $CDCl_3$ at 75.4 MHz.

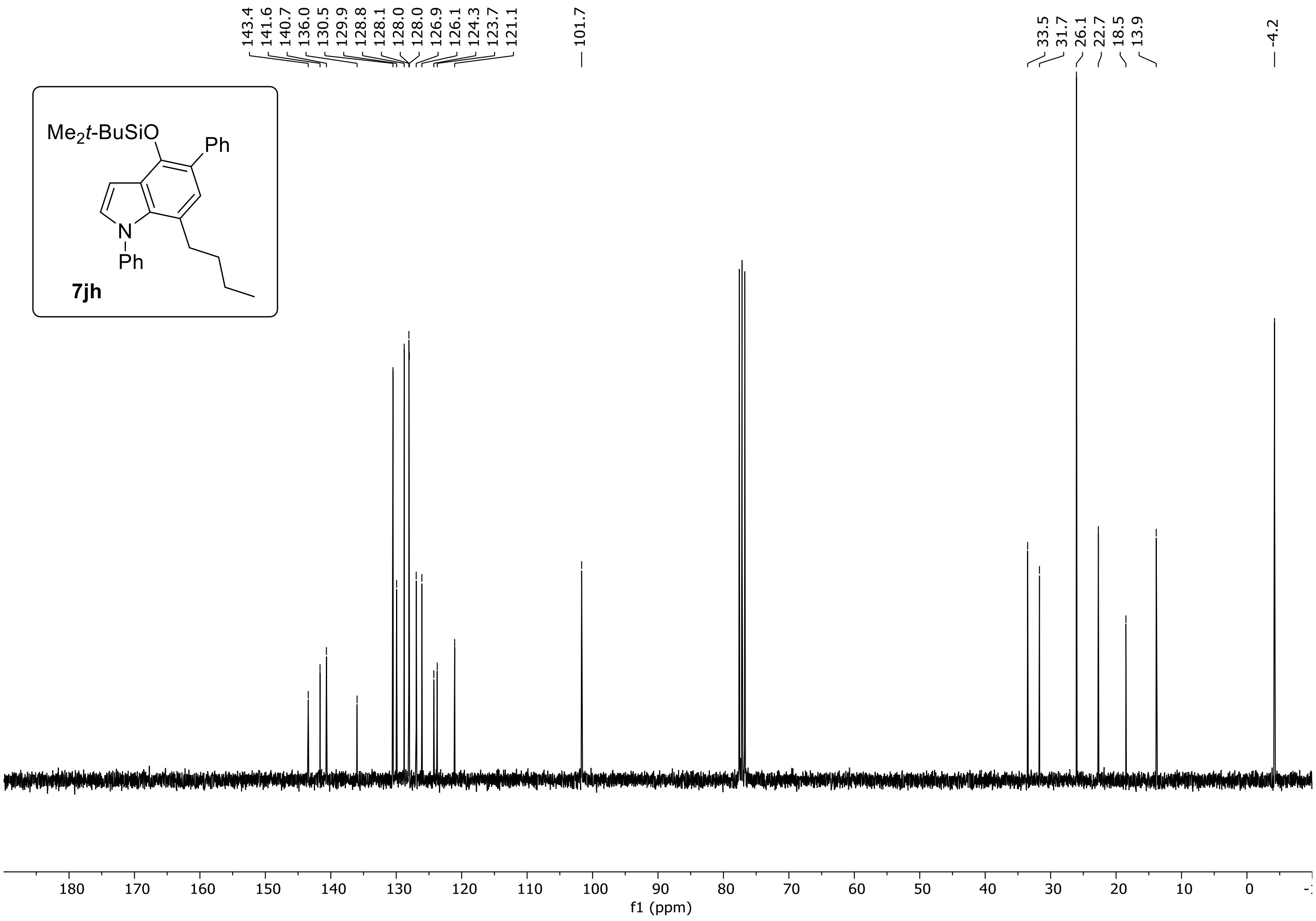

Figure S220: ^{1}H NMR of compound **3ac** in $CDCl_3$ at 300 MHz.

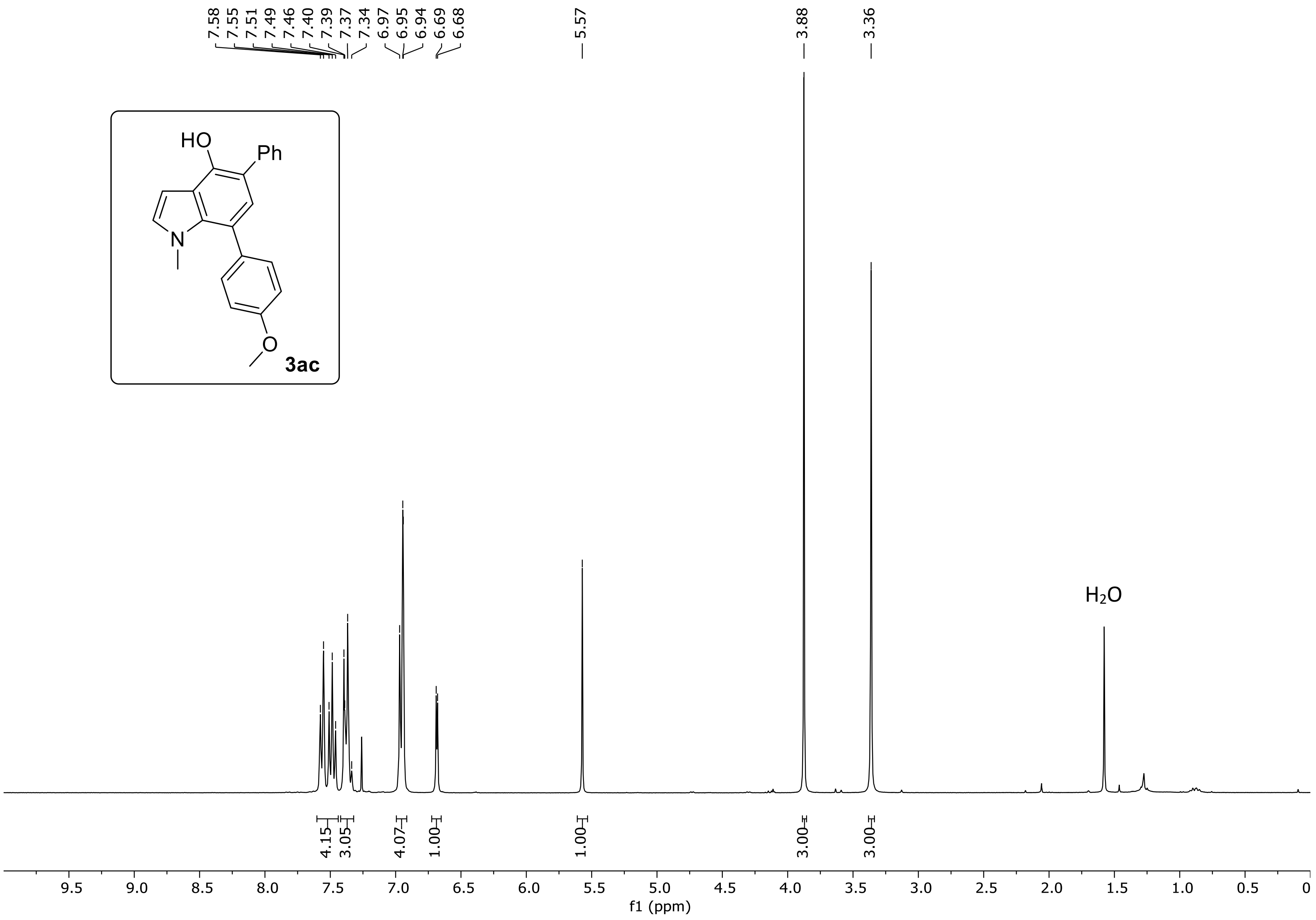

Figure S221: ^{13}C NMR of compound **3ac** in $CDCl_3$ at 75.4 MHz.

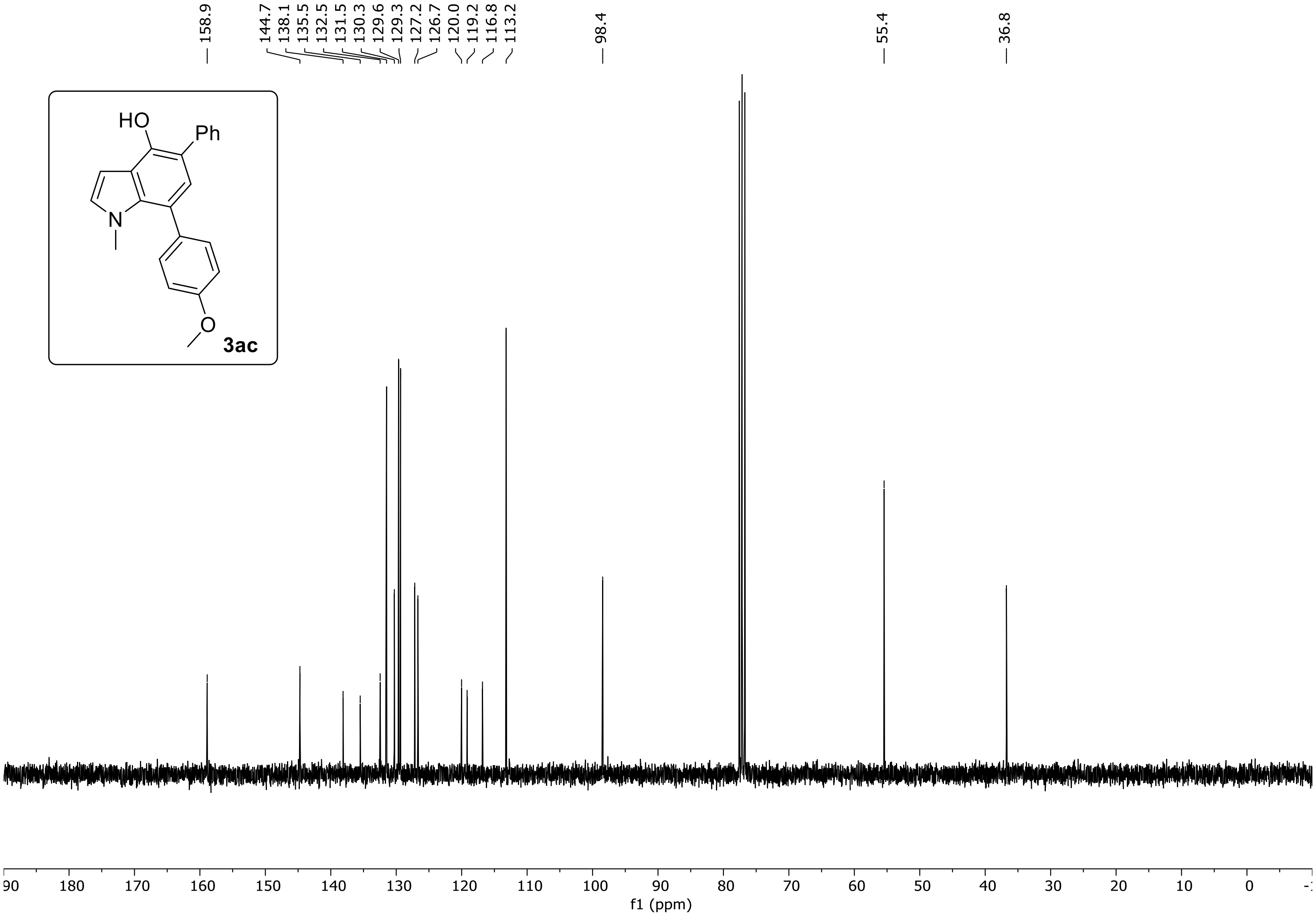

Figure S222: ^{1}H NMR of compound **3ad** in $CDCl_3$ at 300 MHz.

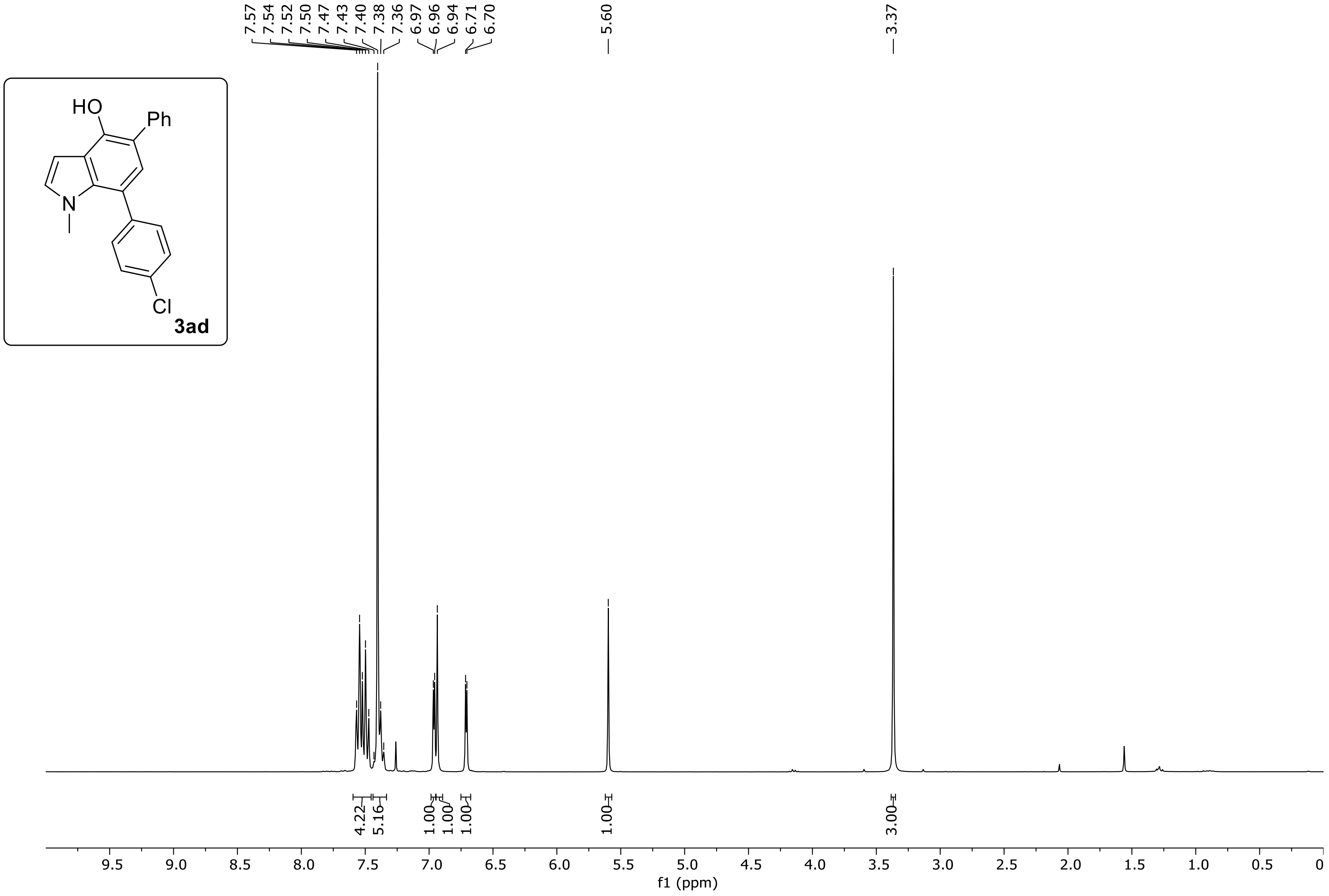

Figure S223: ^{13}C NMR of compound **3ad** in $CDCl_3$ at 75.4 MHz.

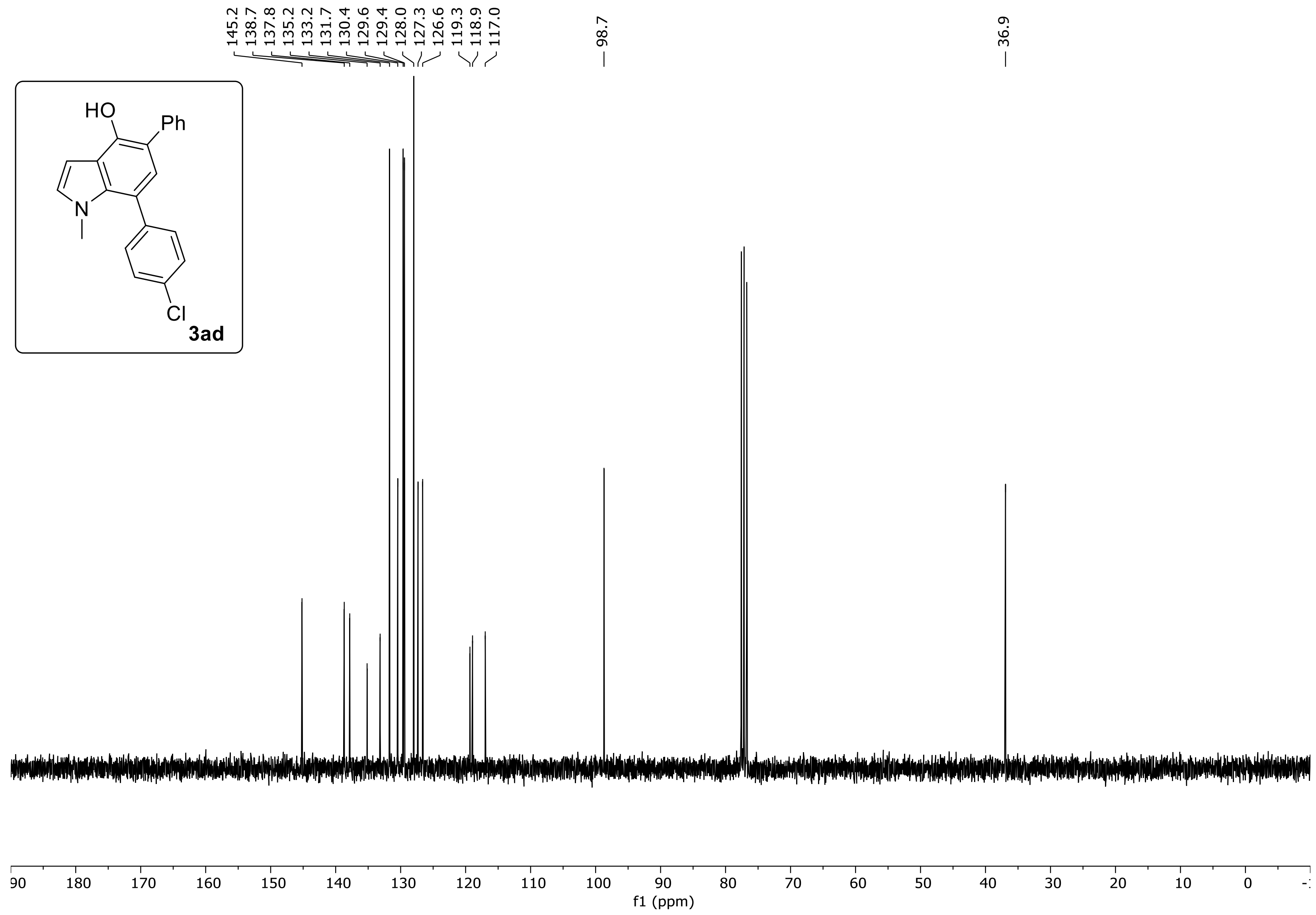

Figure S224: ^{1}H NMR of compound **3ag** in $CDCl_3$ at 300 MHz.

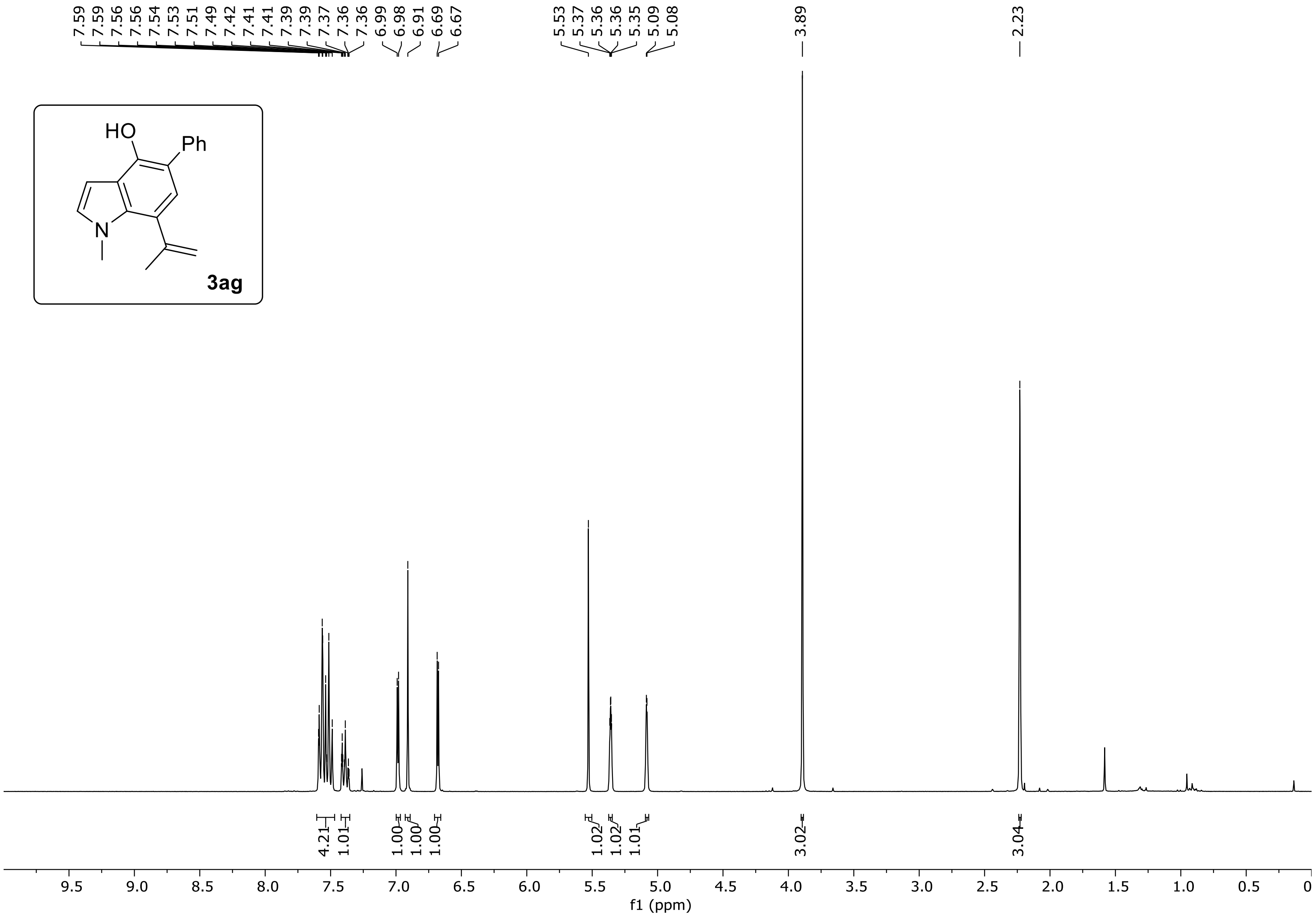

Figure S225: ^{13}C NMR of compound **3ag** in $CDCl_3$ at 75.4 MHz.

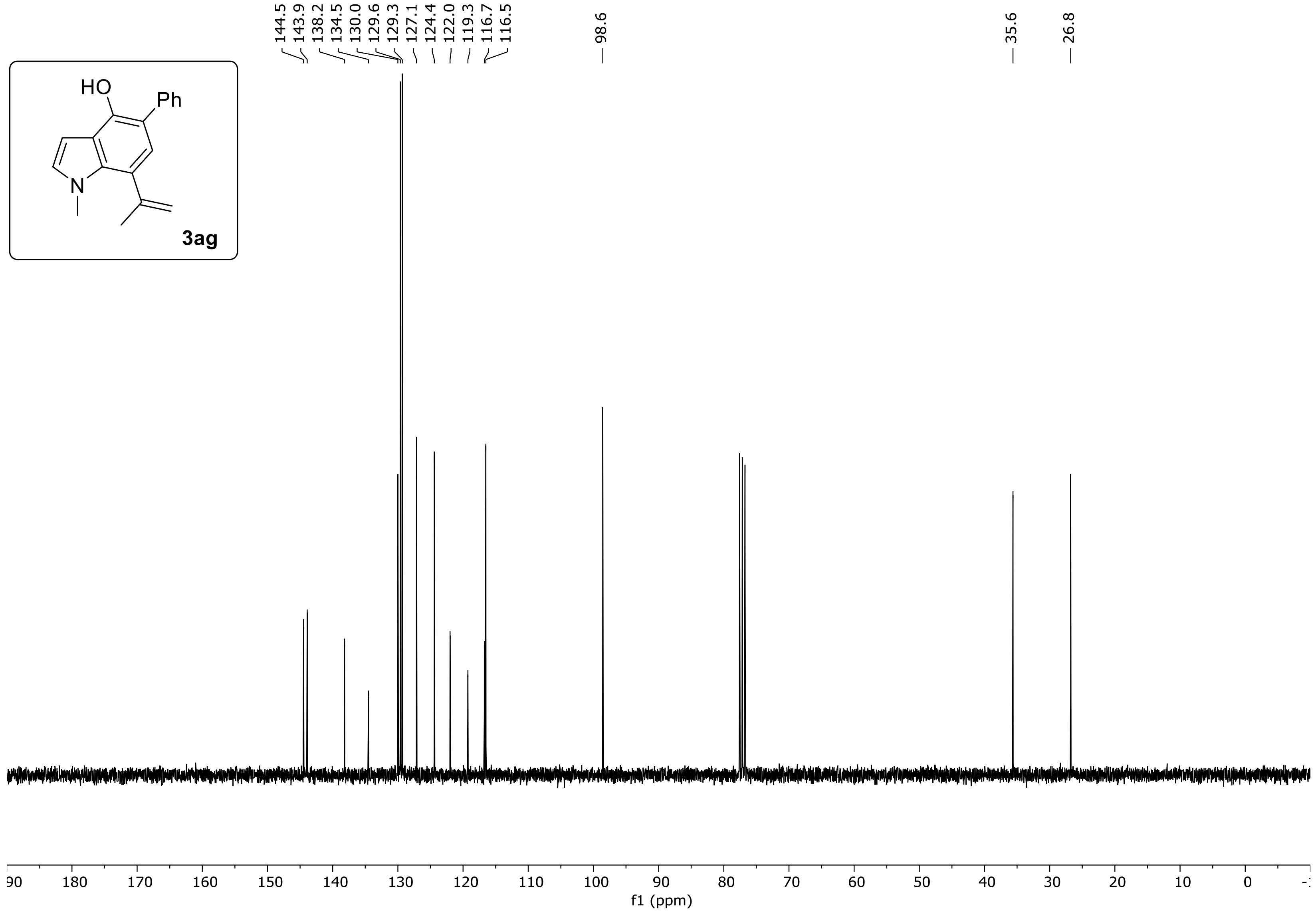

Figure S226: ^{1}H NMR of compound **3ai** in $CDCl_3$ at 300 MHz.

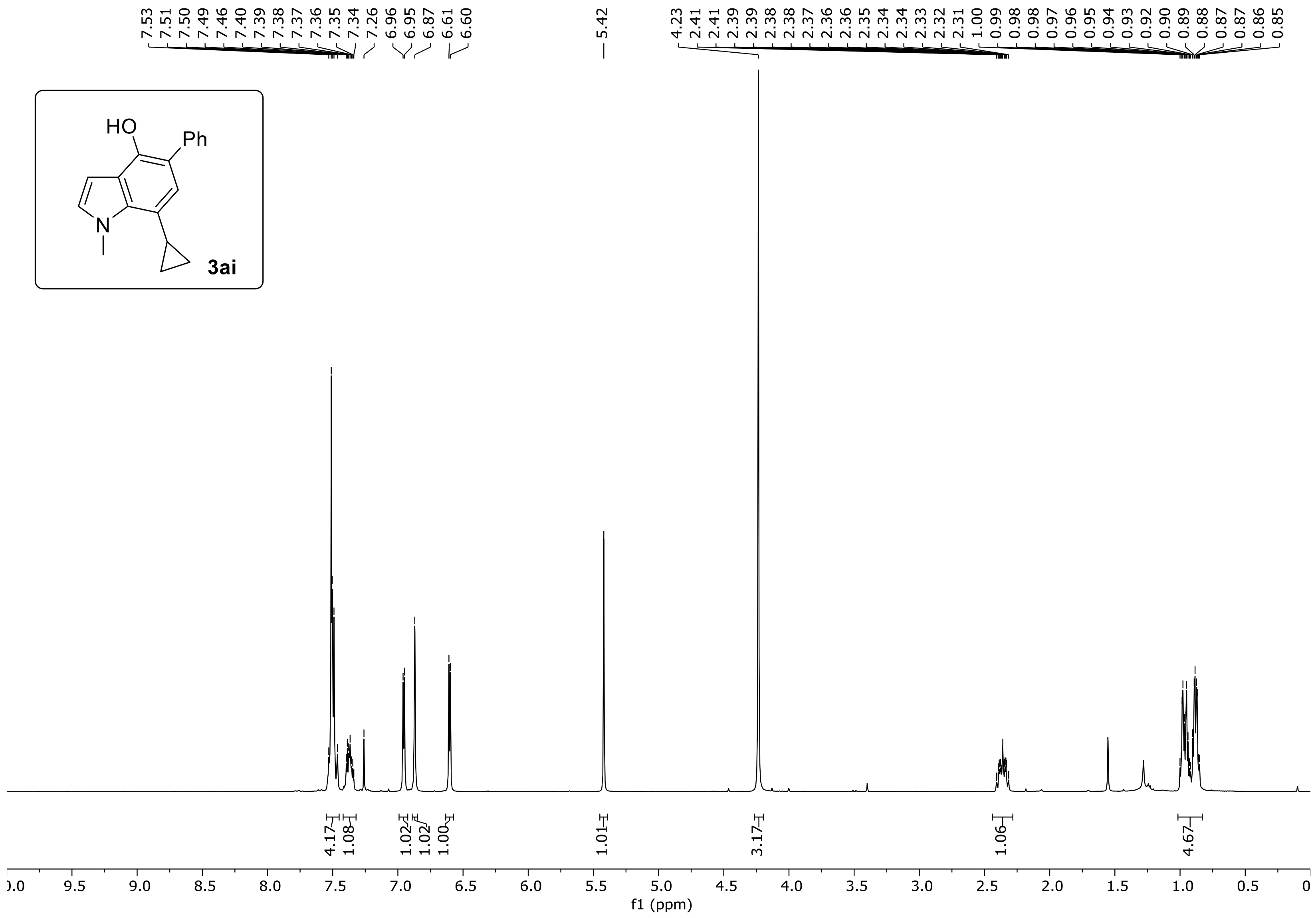

Figure S227: ^{13}C NMR of compound **3ai** in $CDCl_3$ at 75.4 MHz.

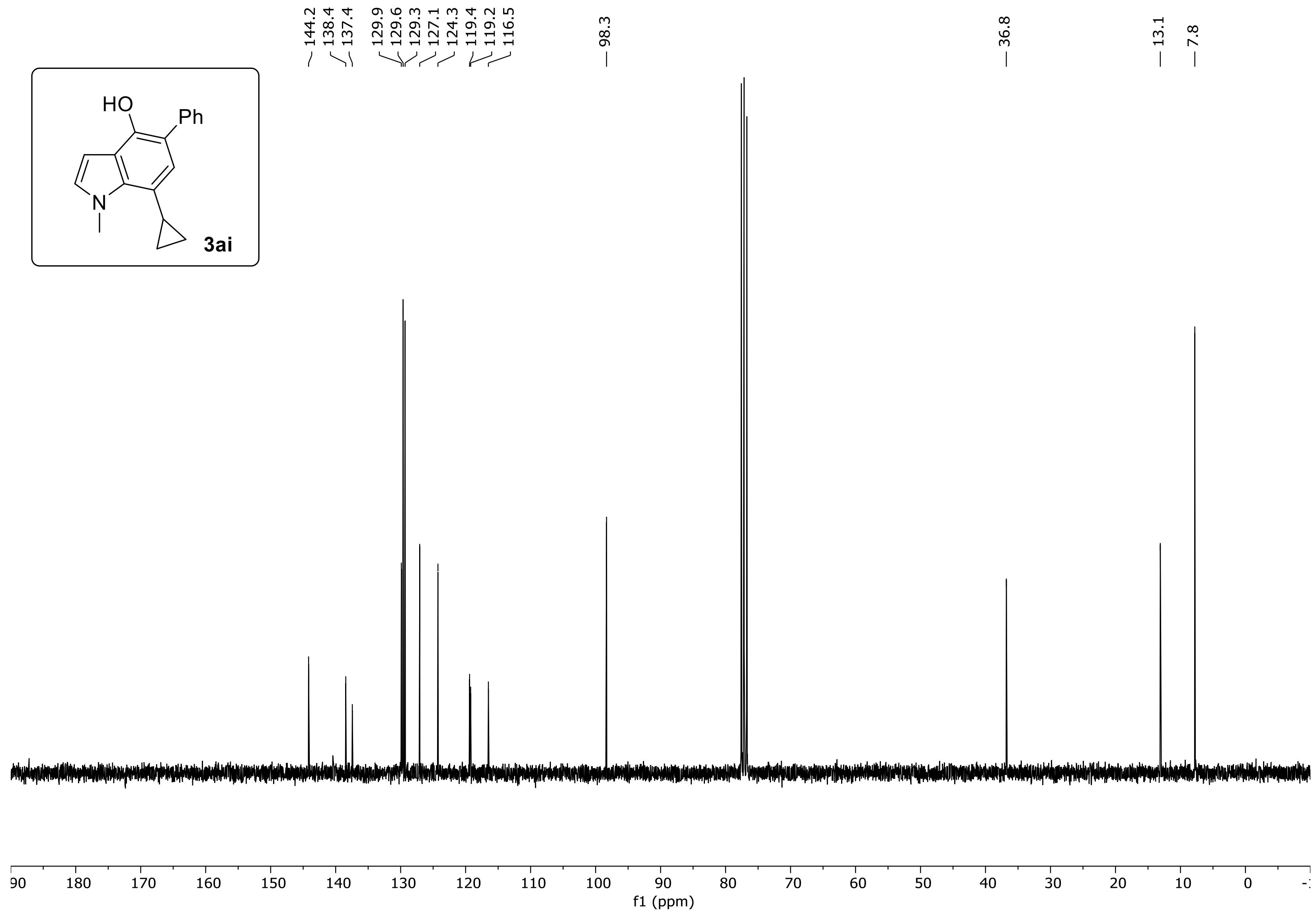

Figure S228: ^{1}H NMR of compound **3ak** in $CDCl_3$ at 300 MHz.

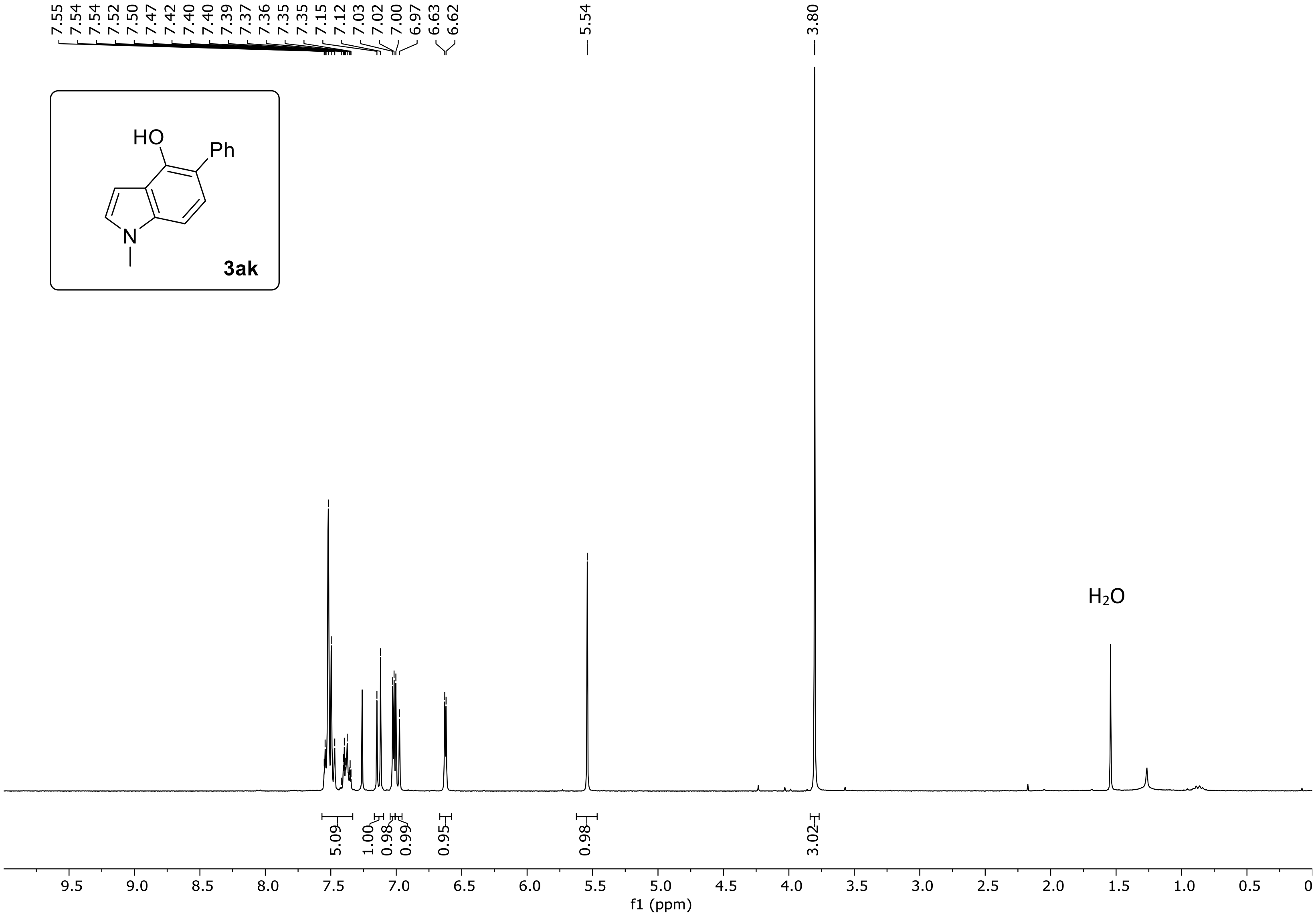

Figure S229: ^{13}C NMR of compound **3ak** in $CDCl_3$ at 75.4 MHz.

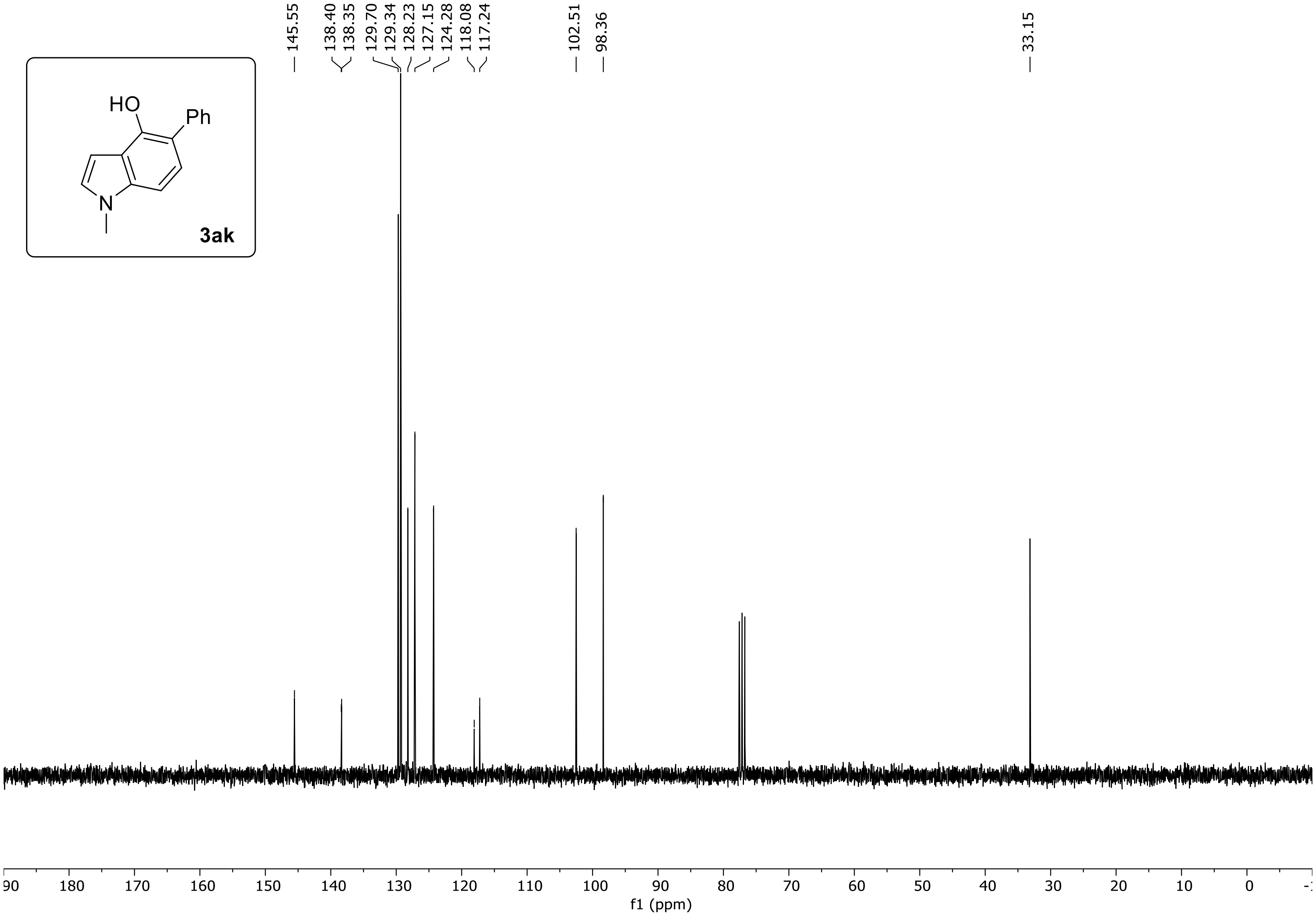

Figure S230: ^{1}H NMR of compound **3ba** in $CDCl_3$ at 300 MHz.

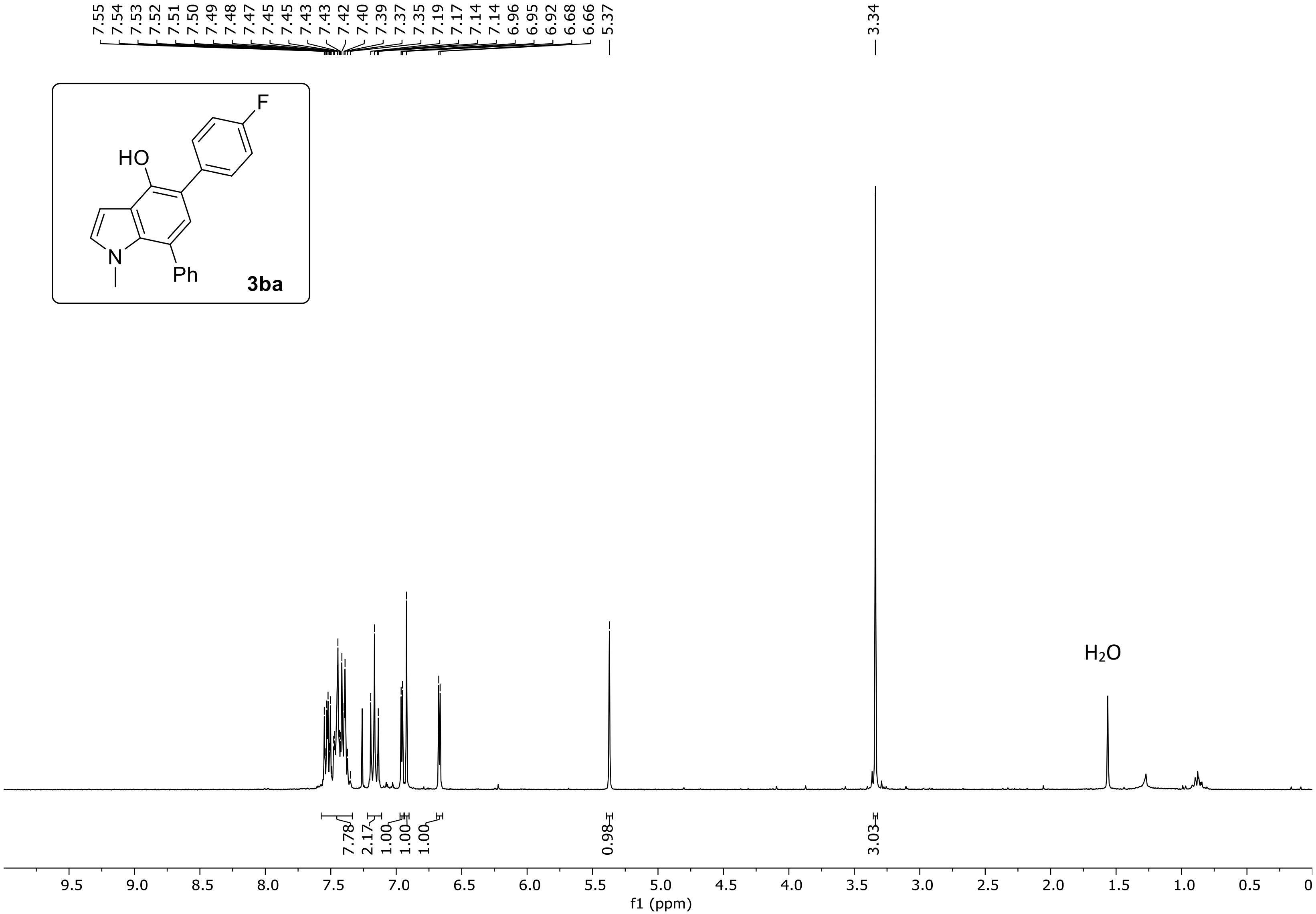

Figure S231: ^{13}C NMR of compound **3ba** in $CDCl_3$ at 75.4 MHz.

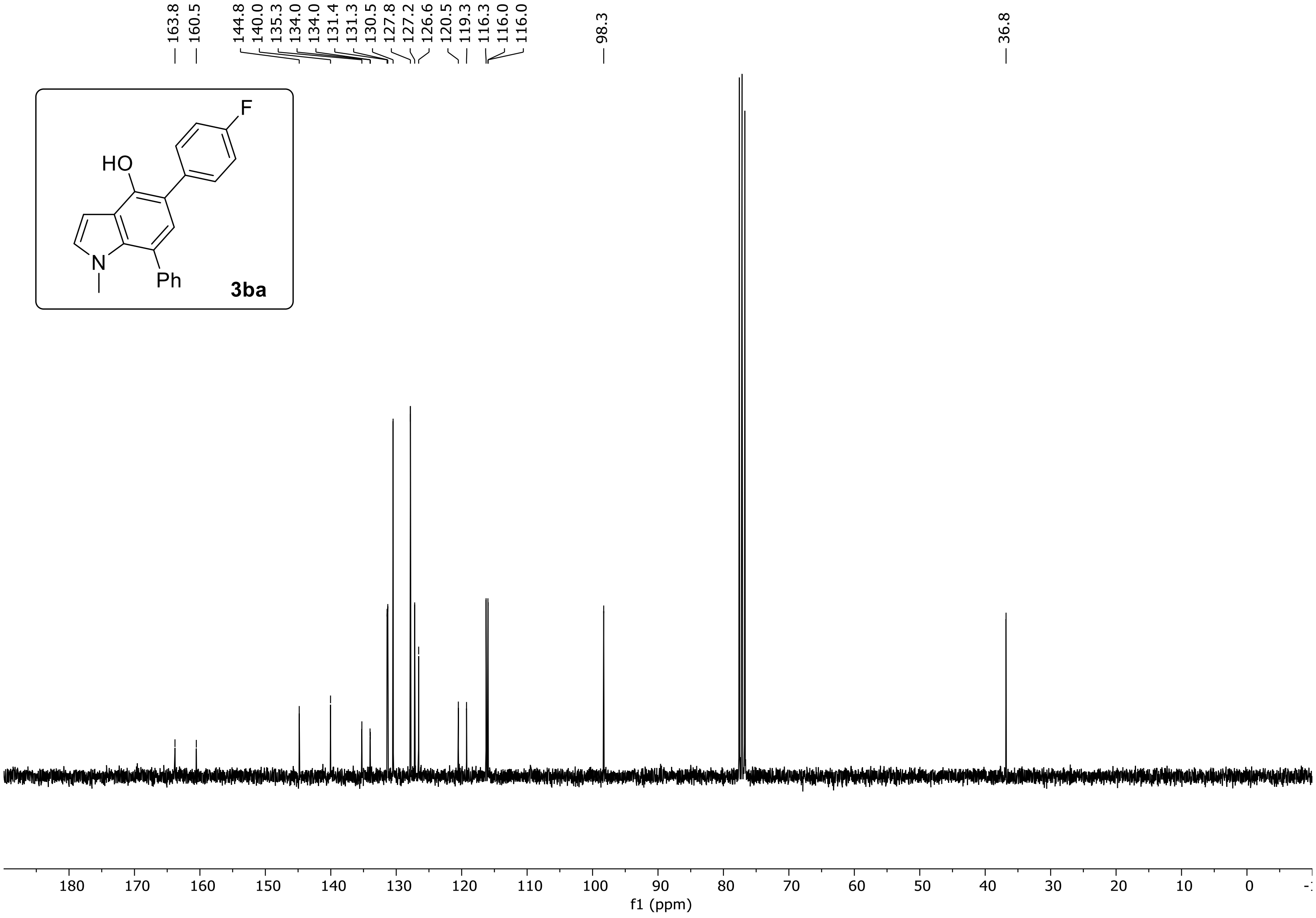

Figure S232: ^{1}H NMR of compound **3bf** in $CDCl_3$ at 300 MHz.

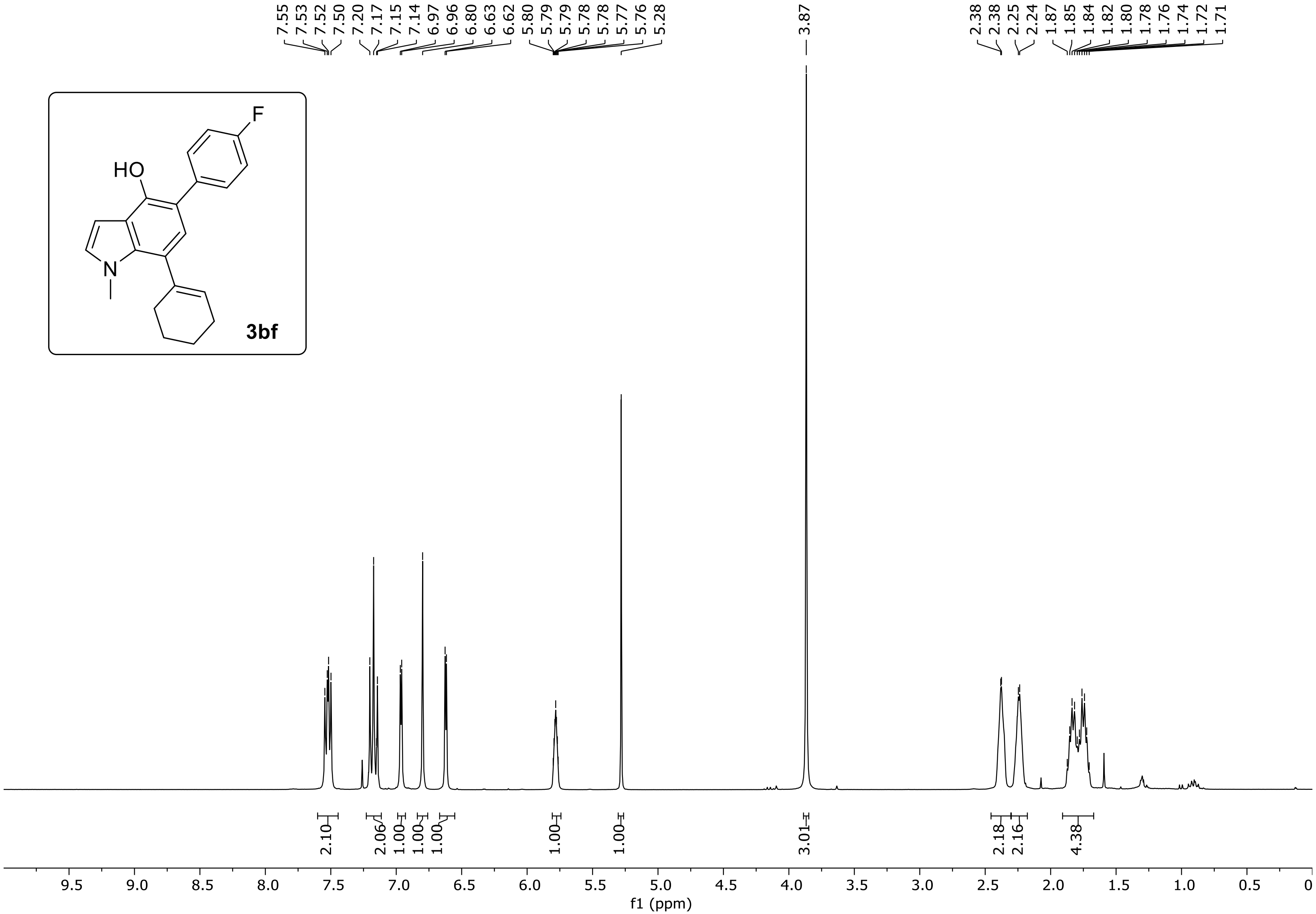

Figure S233: ^{13}C NMR of compound **3bf** in $CDCl_3$ at 75.4 MHz.

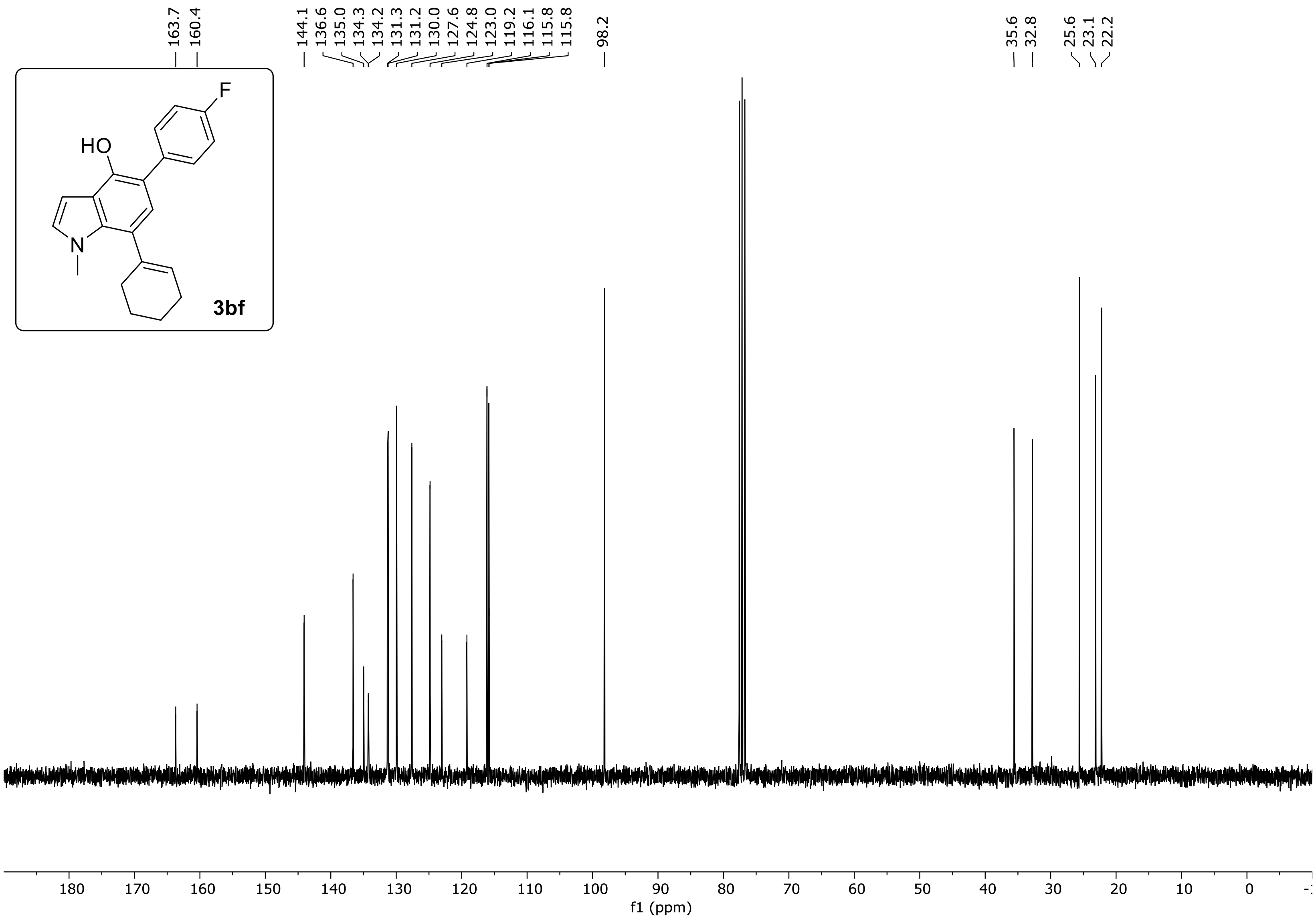

Figure S234: ^{1}H NMR of compound **3ca** in $CDCl_3$ at 300 MHz.

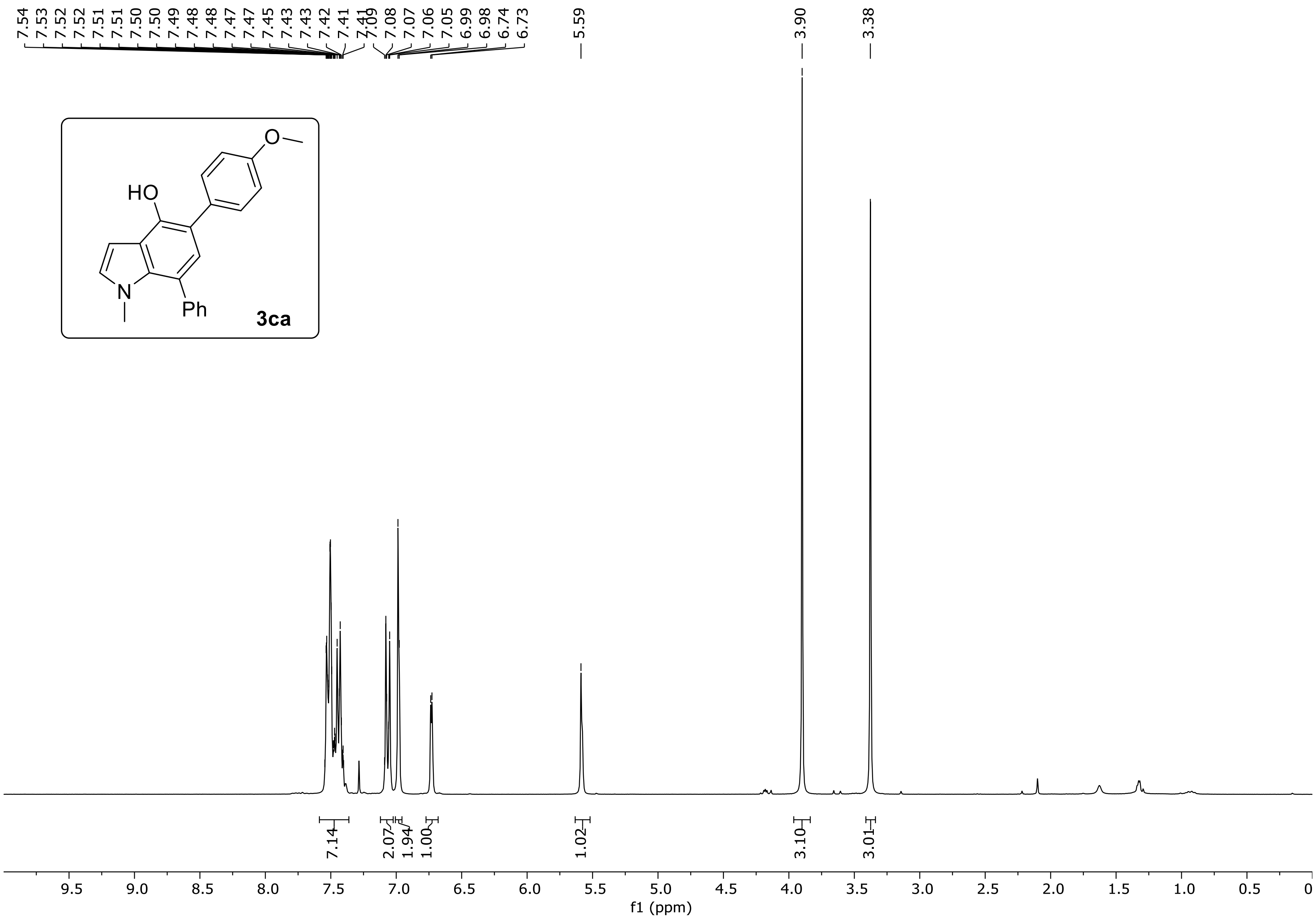

Figure S235: ^{13}C NMR of compound **3ca** in $CDCl_3$ at 75.4 MHz.

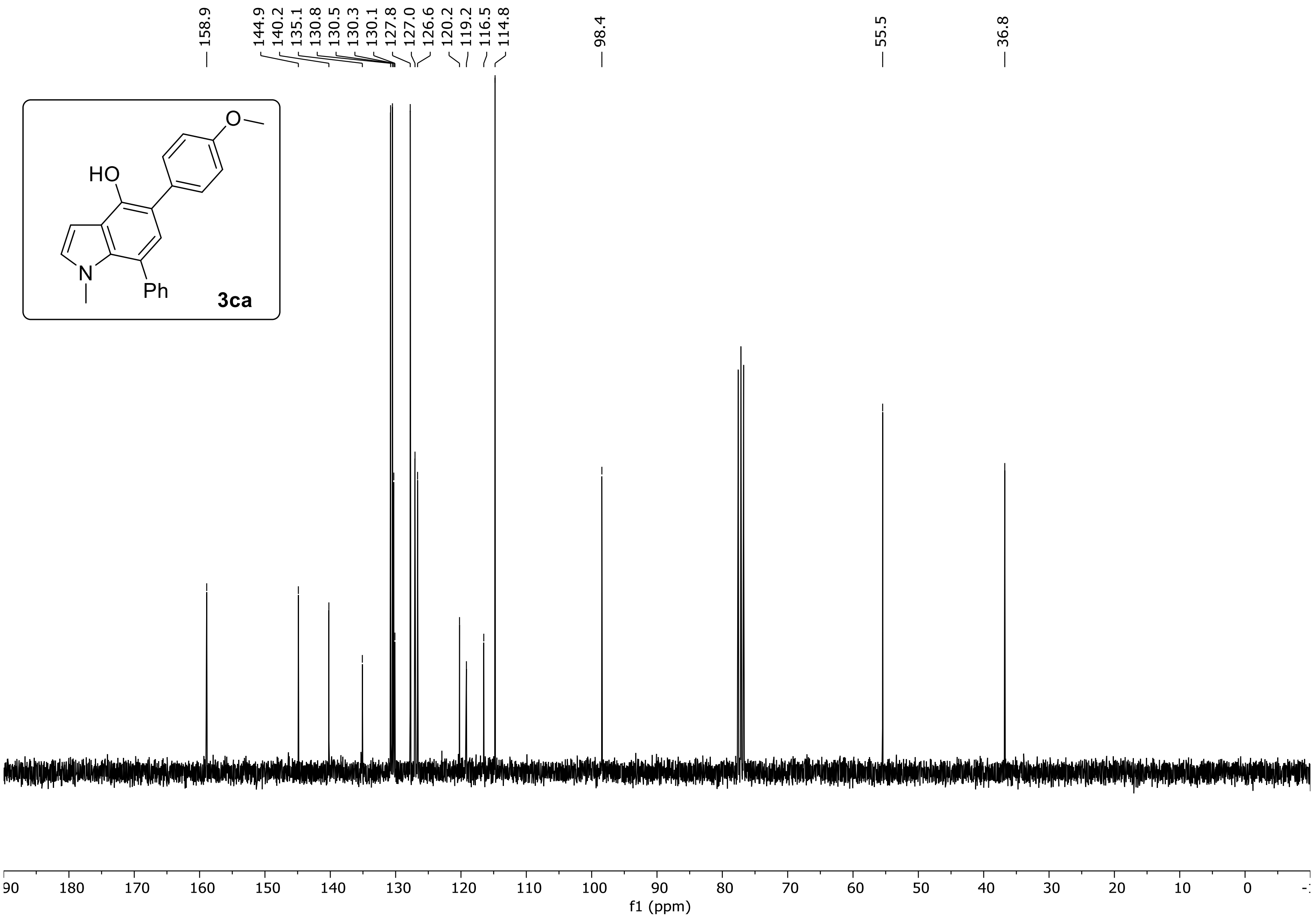

Figure S236: ^{1}H NMR of compound **3fa** in $CDCl_3$ at 300 MHz.

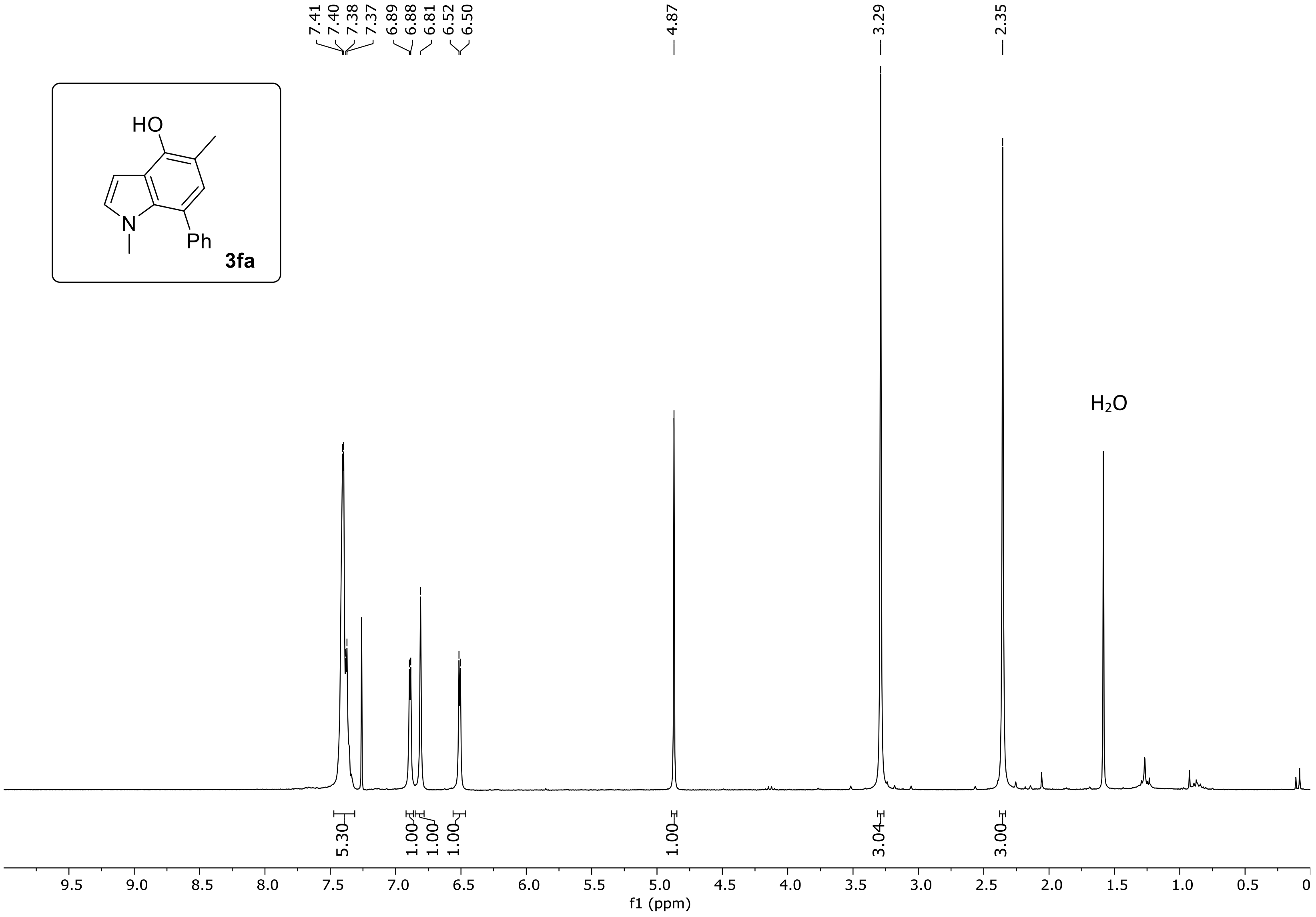

Figure S237: ^{13}C NMR of compound **3fa** in $CDCl_3$ at 75.4 MHz.

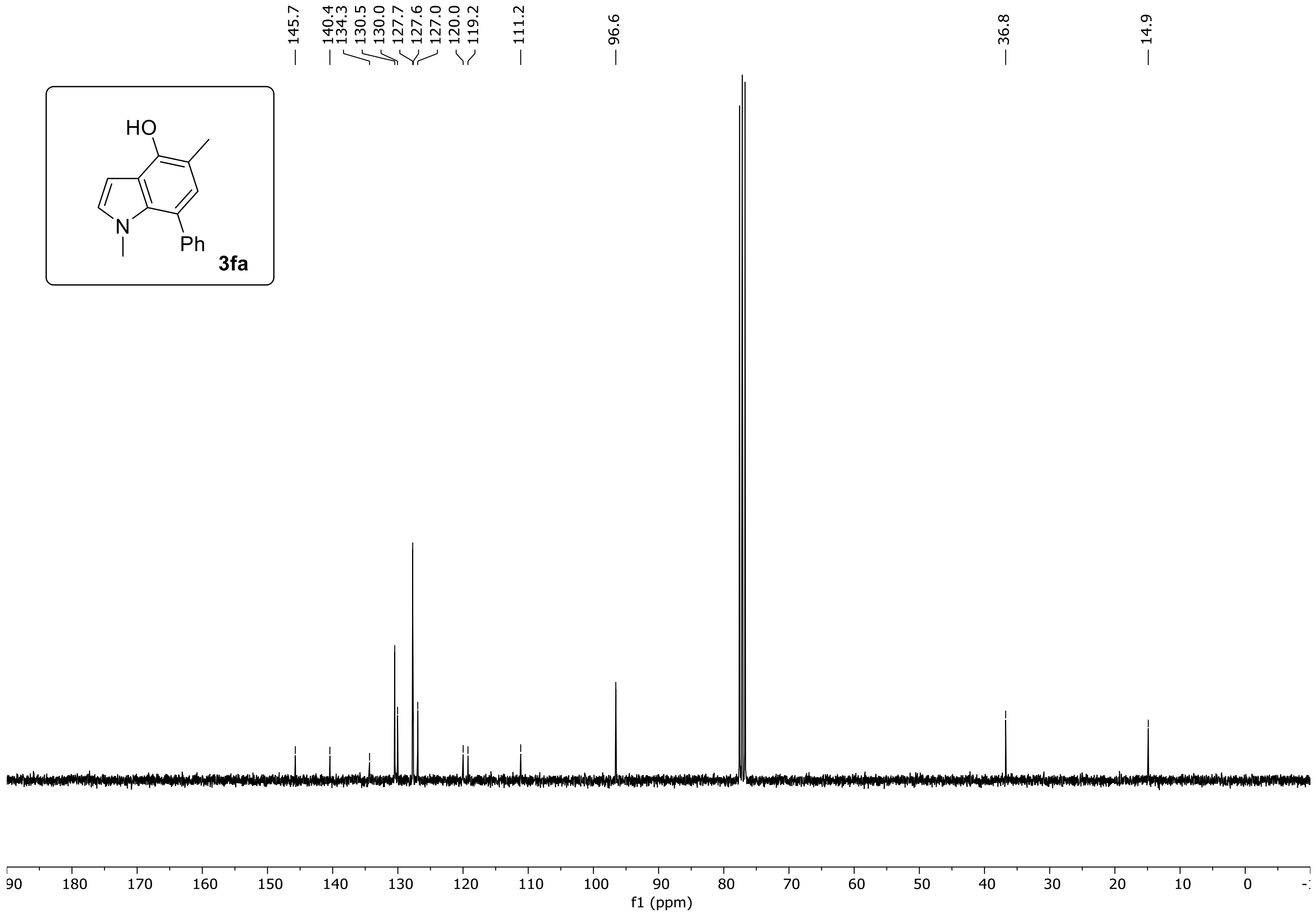

Figure S238: ¹H NMR of compound **3fb** in $CDCl_3$ at 300 MHz.

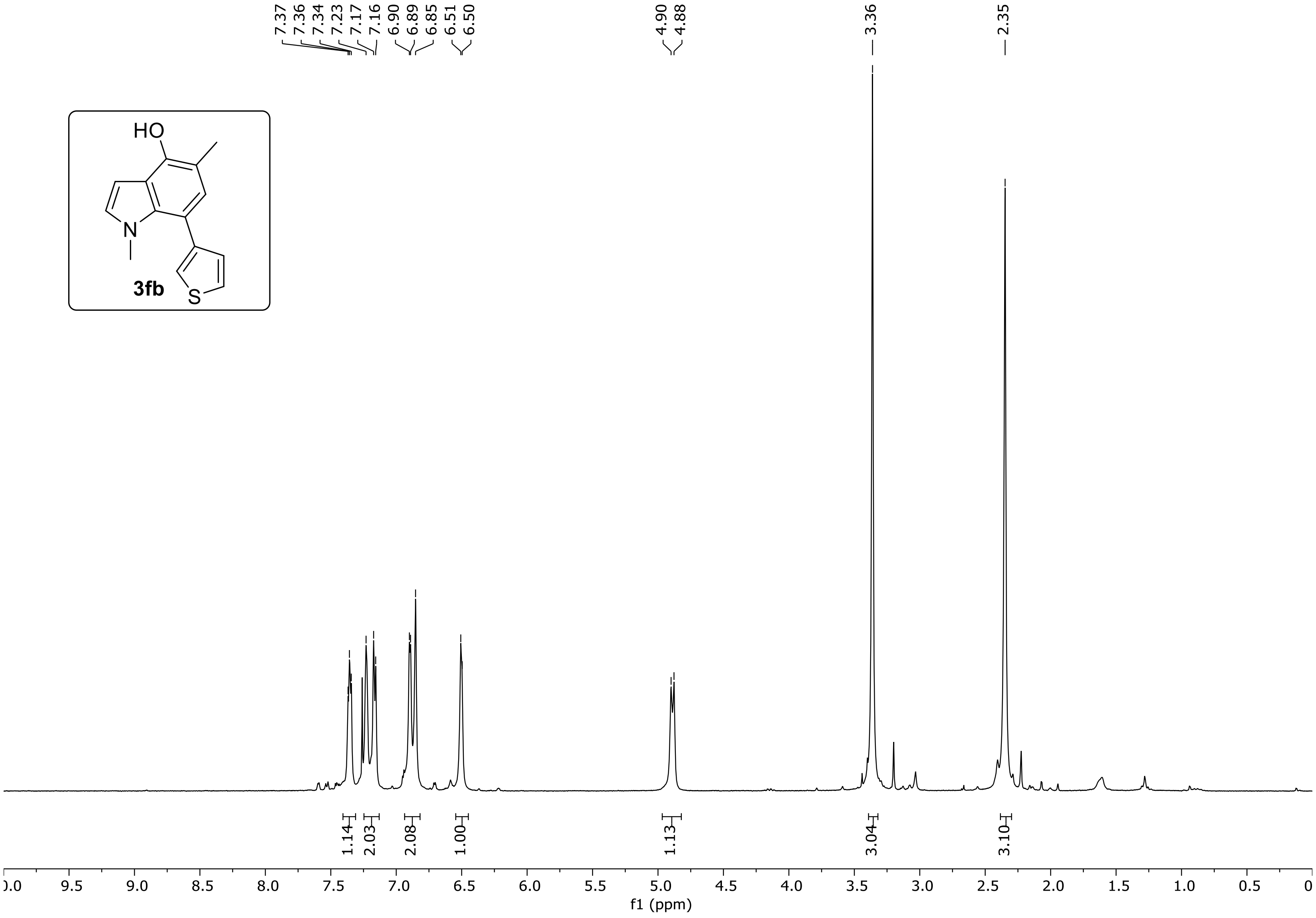

Figure S239: ^{13}C NMR of compound **3fb** in $CDCl_3$ at 75.4 MHz.

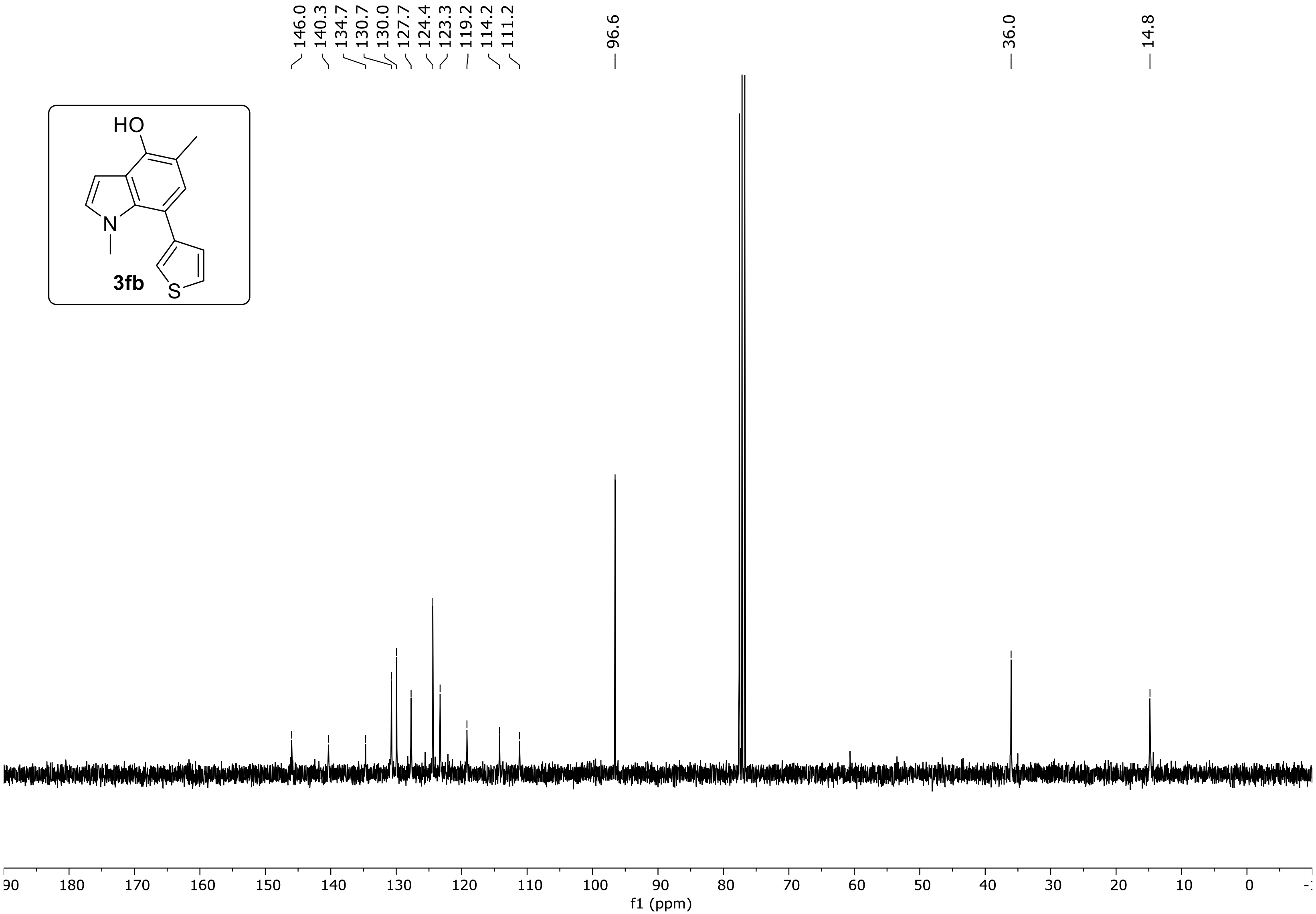

Figure S240: ^{1}H NMR of compound **3ha** in $CDCl_3$ at 300 MHz.

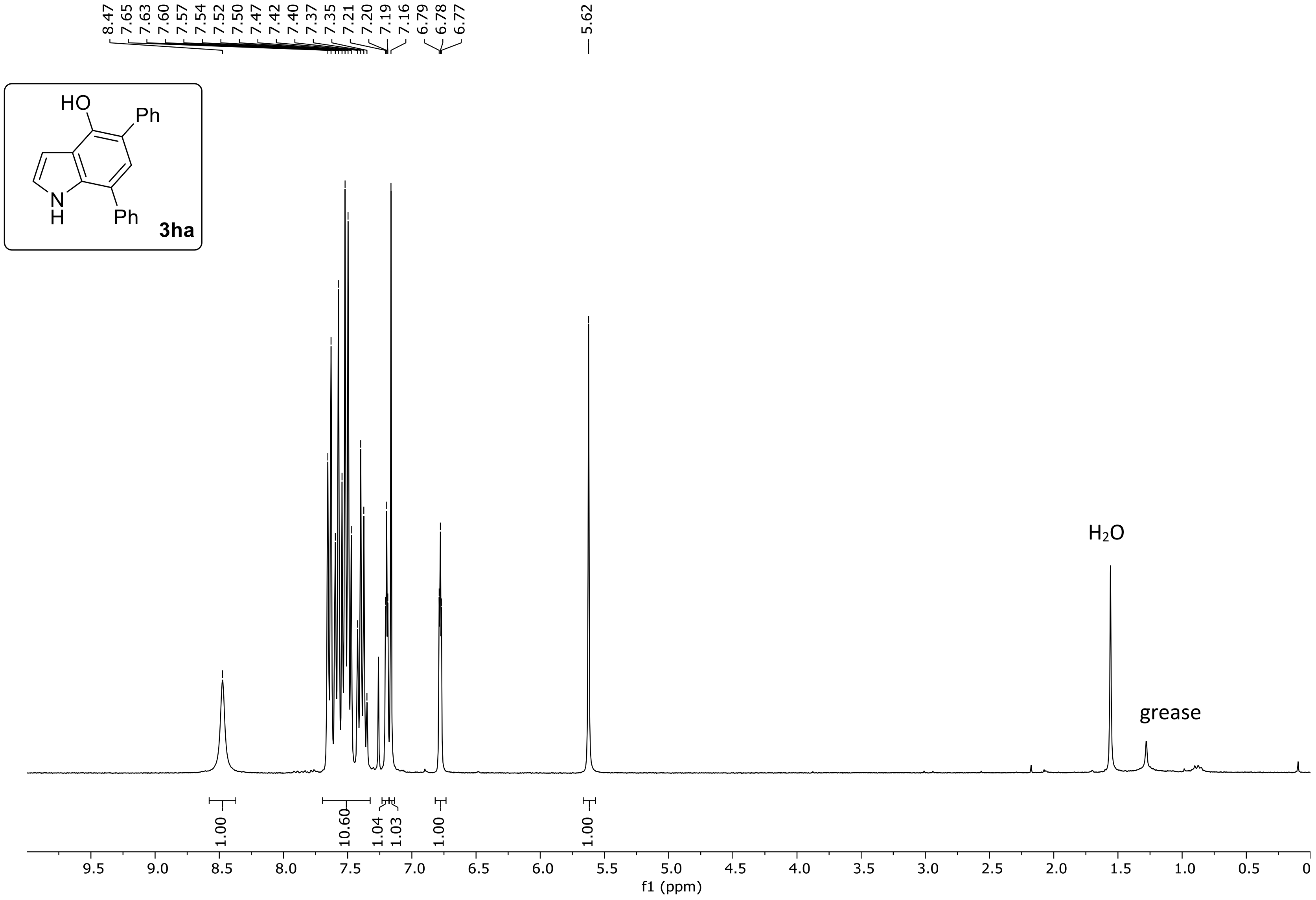

Figure S241: ^{13}C NMR of compound **3ha** in $CDCl_3$ at 75.4 MHz.

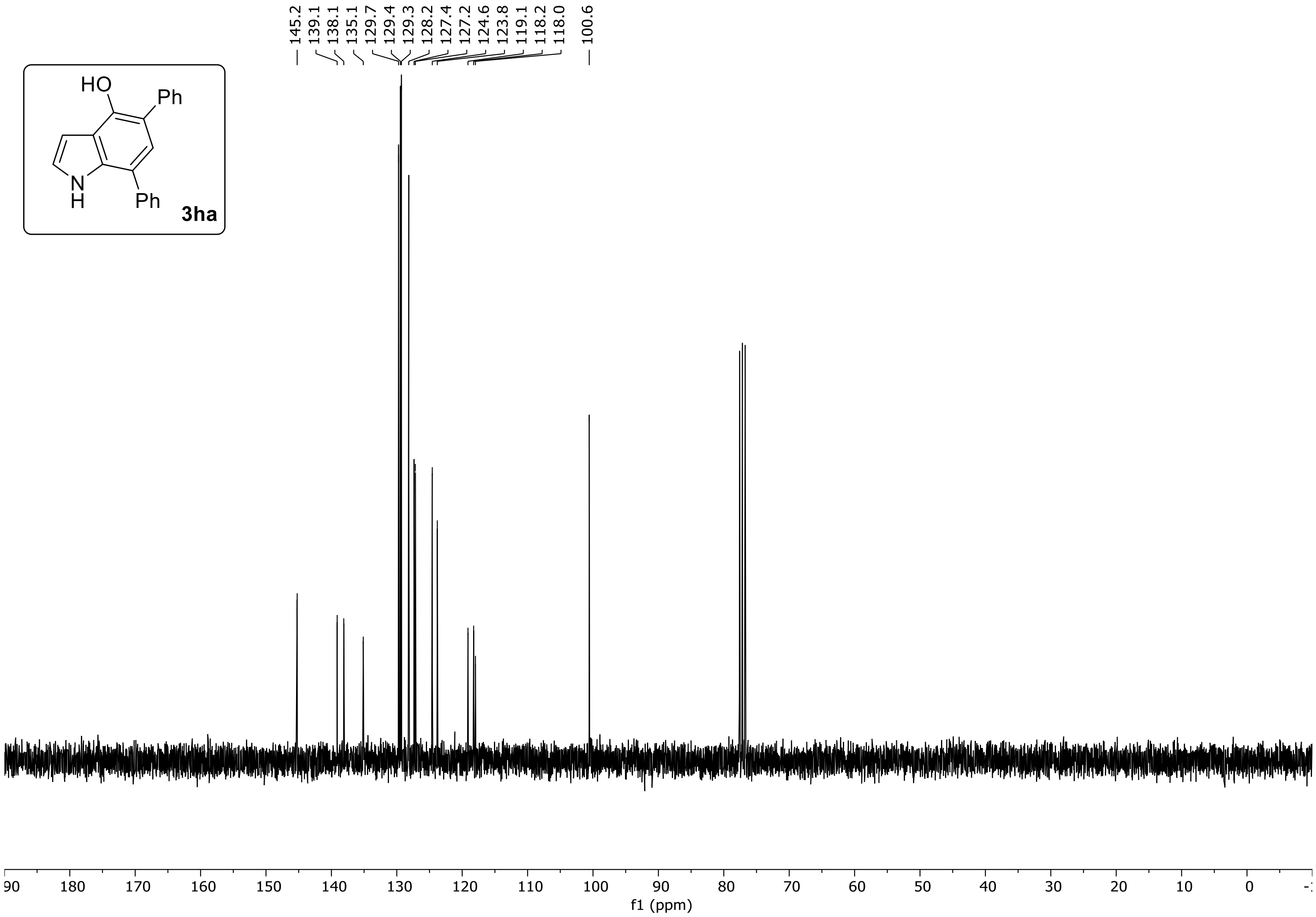

Figure S242: ^{1}H NMR of compound **3hb** in $CDCl_3$ at 300 MHz.

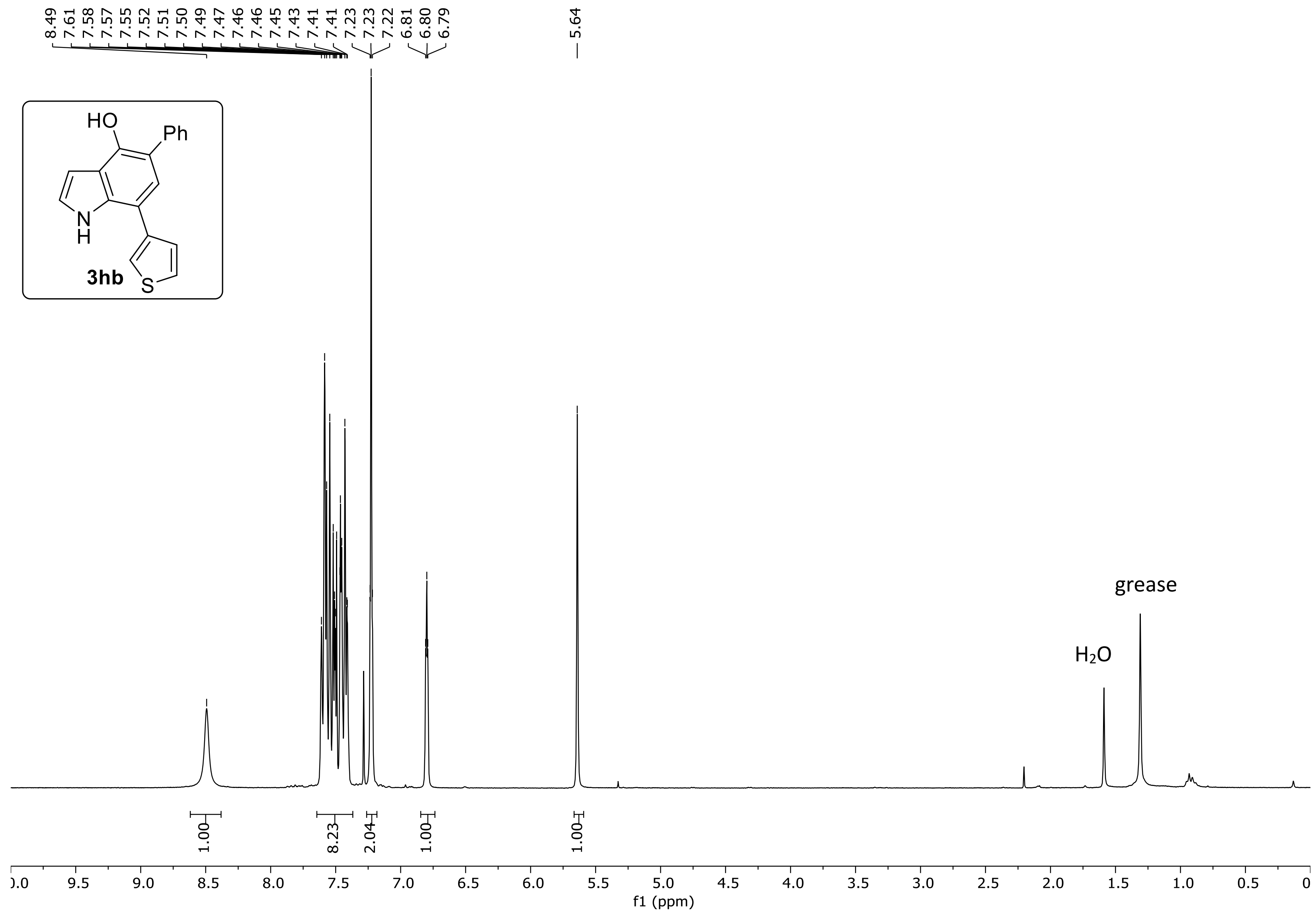

Figure S243: ^{13}C NMR of compound **3hb** in $CDCl_3$ at 75.4 MHz.

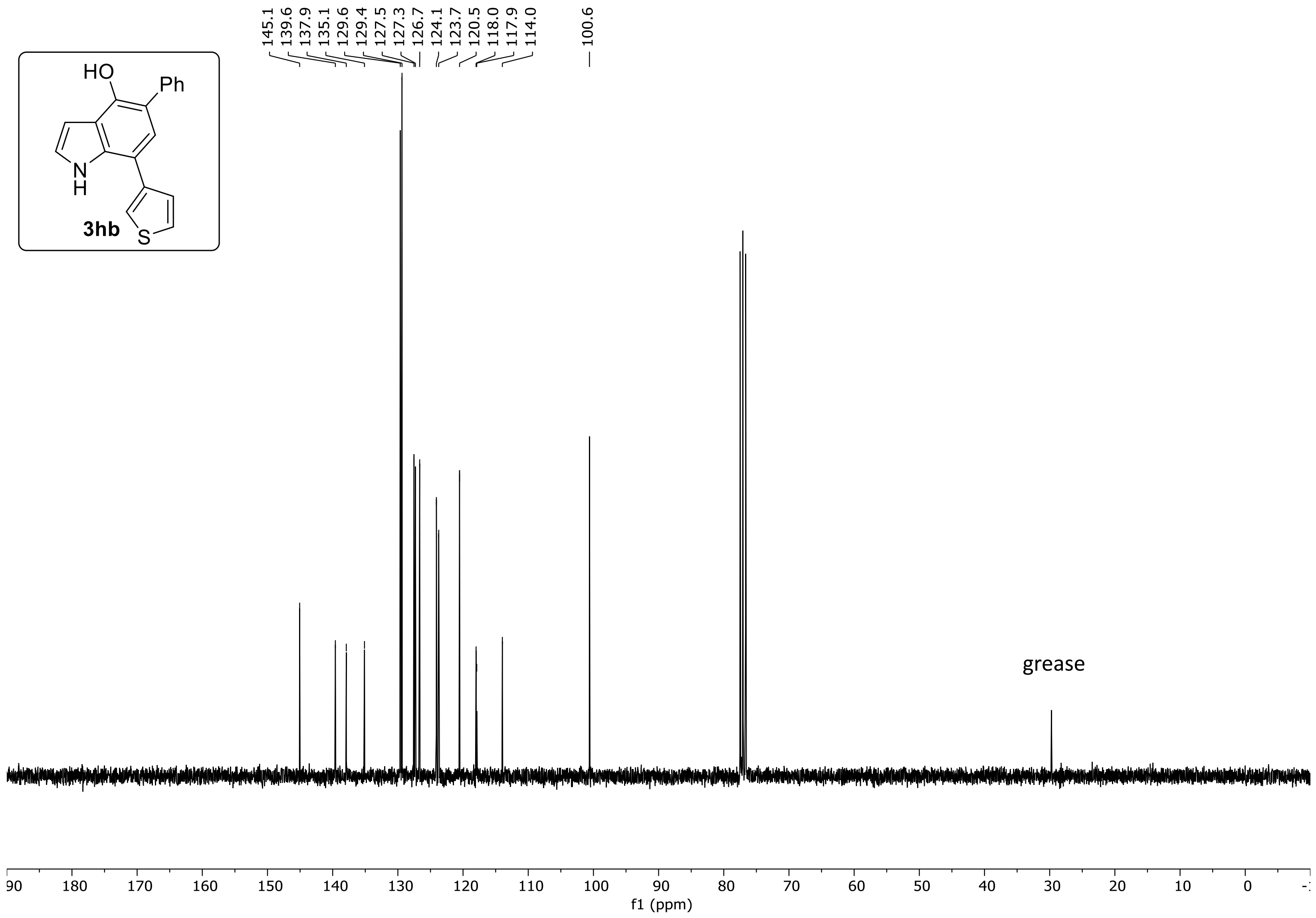

Figure S244: ^{1}H NMR of compound **3hh** in $CDCl_3$ at 300 MHz.

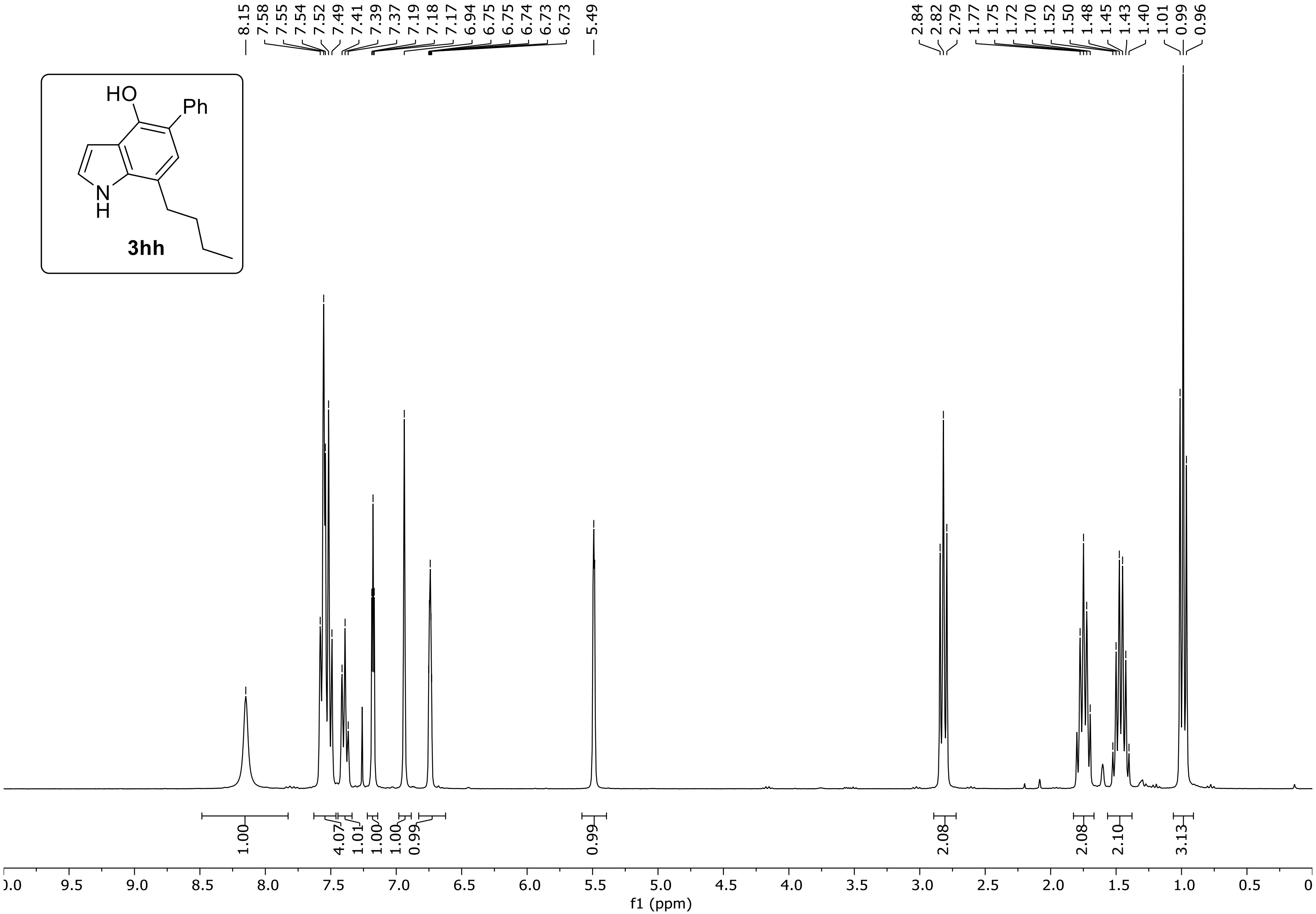

Figure S245: ^{13}C NMR of compound **3hh** in $CDCl_3$ at 75.4 MHz.

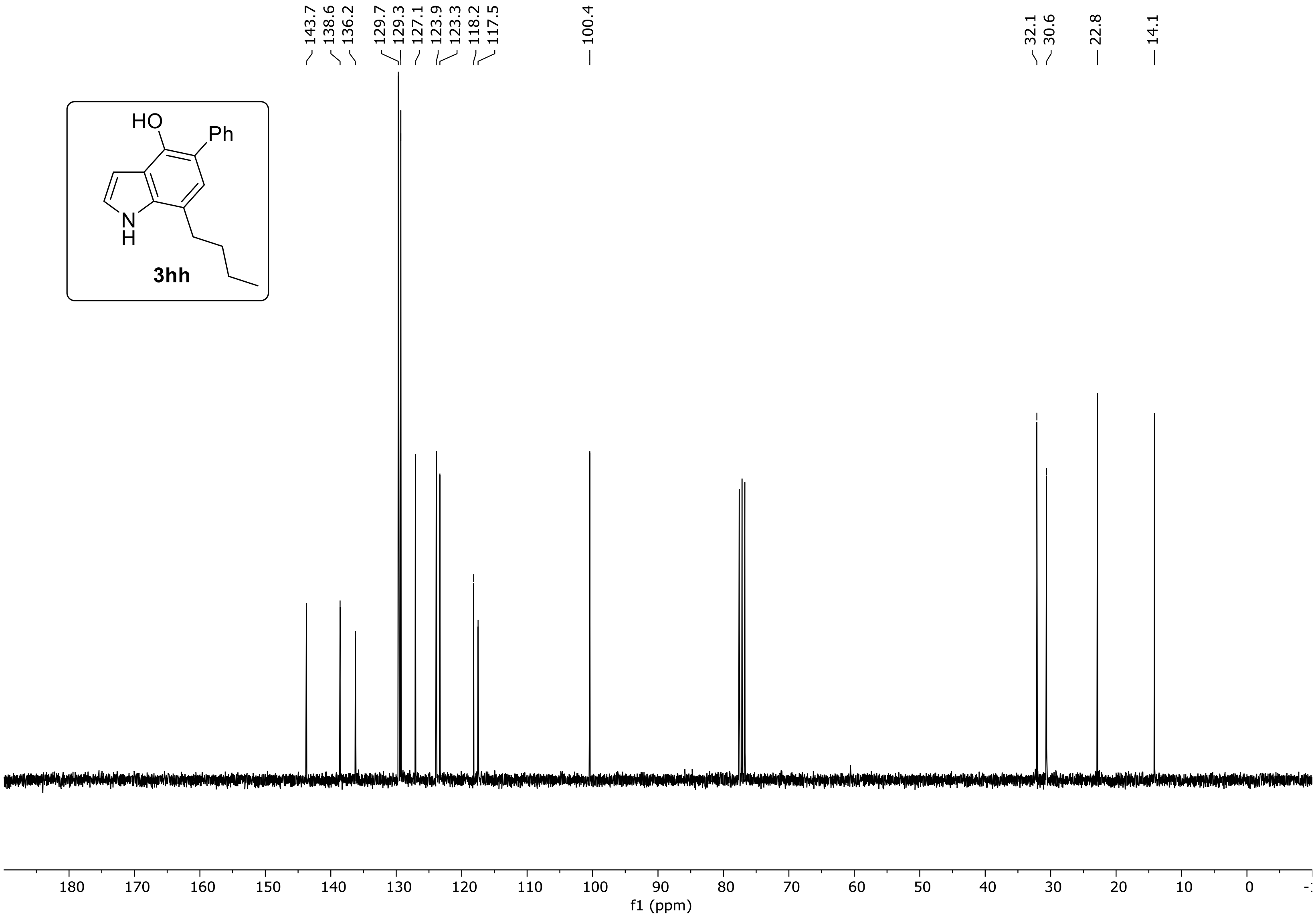

Figure S246: ^{1}H NMR of compound **3ia** in $CDCl_3$ at 300 MHz.

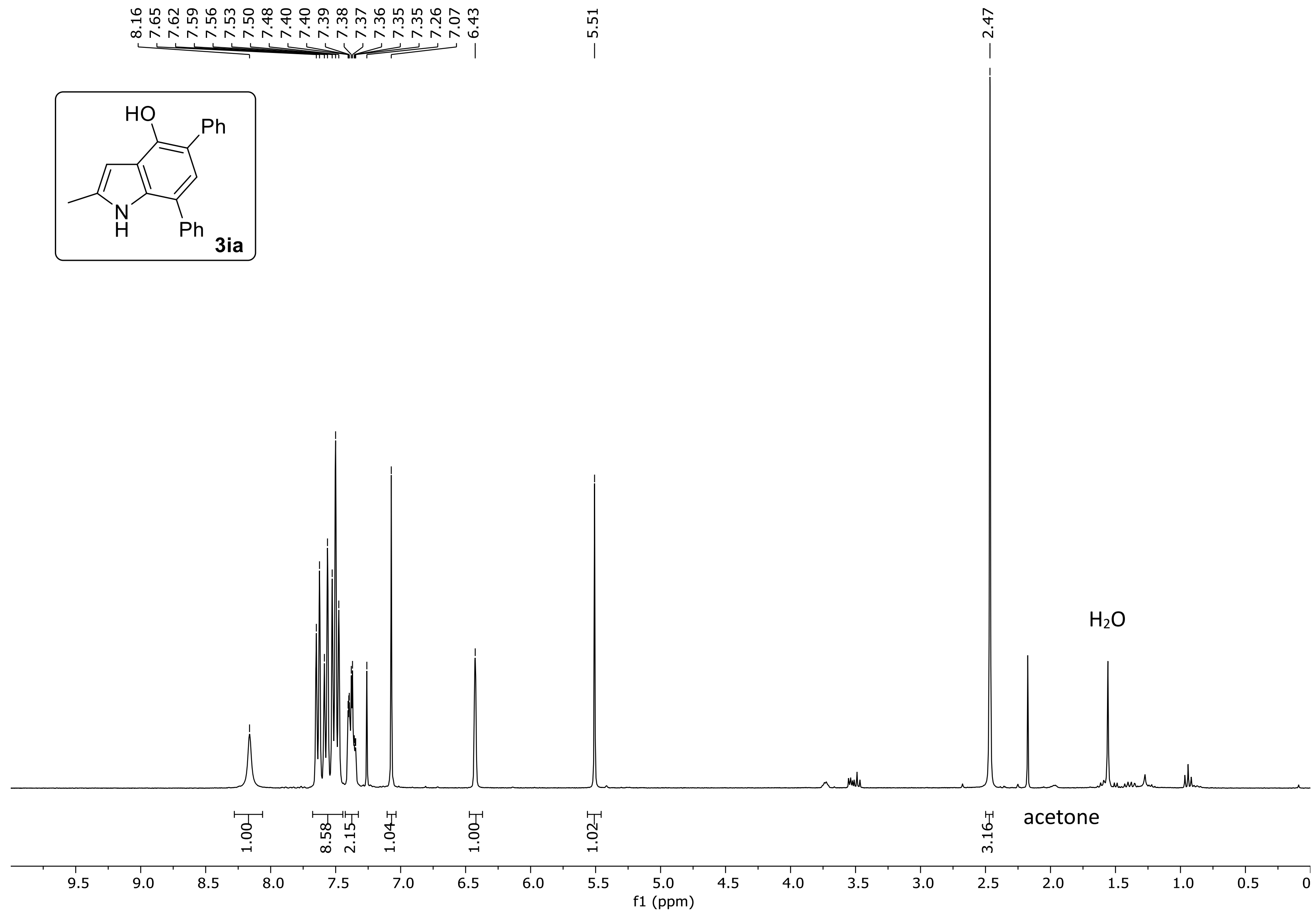

Figure S247: ^{13}C NMR of compound **3ia** in $CDCl_3$ at 75.4 MHz.

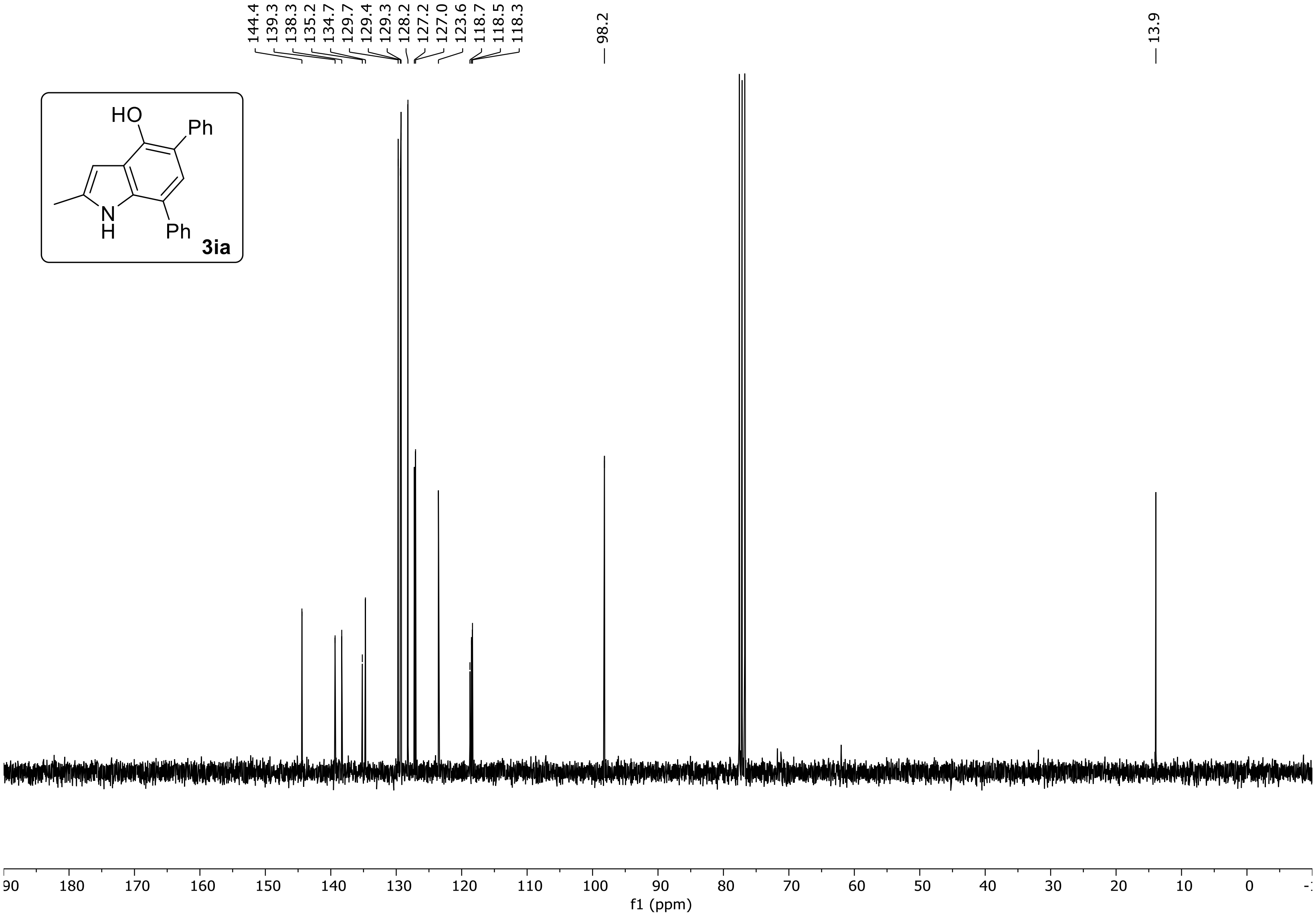

Figure S248: ^{1}H NMR of compound **9** in $CDCl_3$ at 300 MHz.

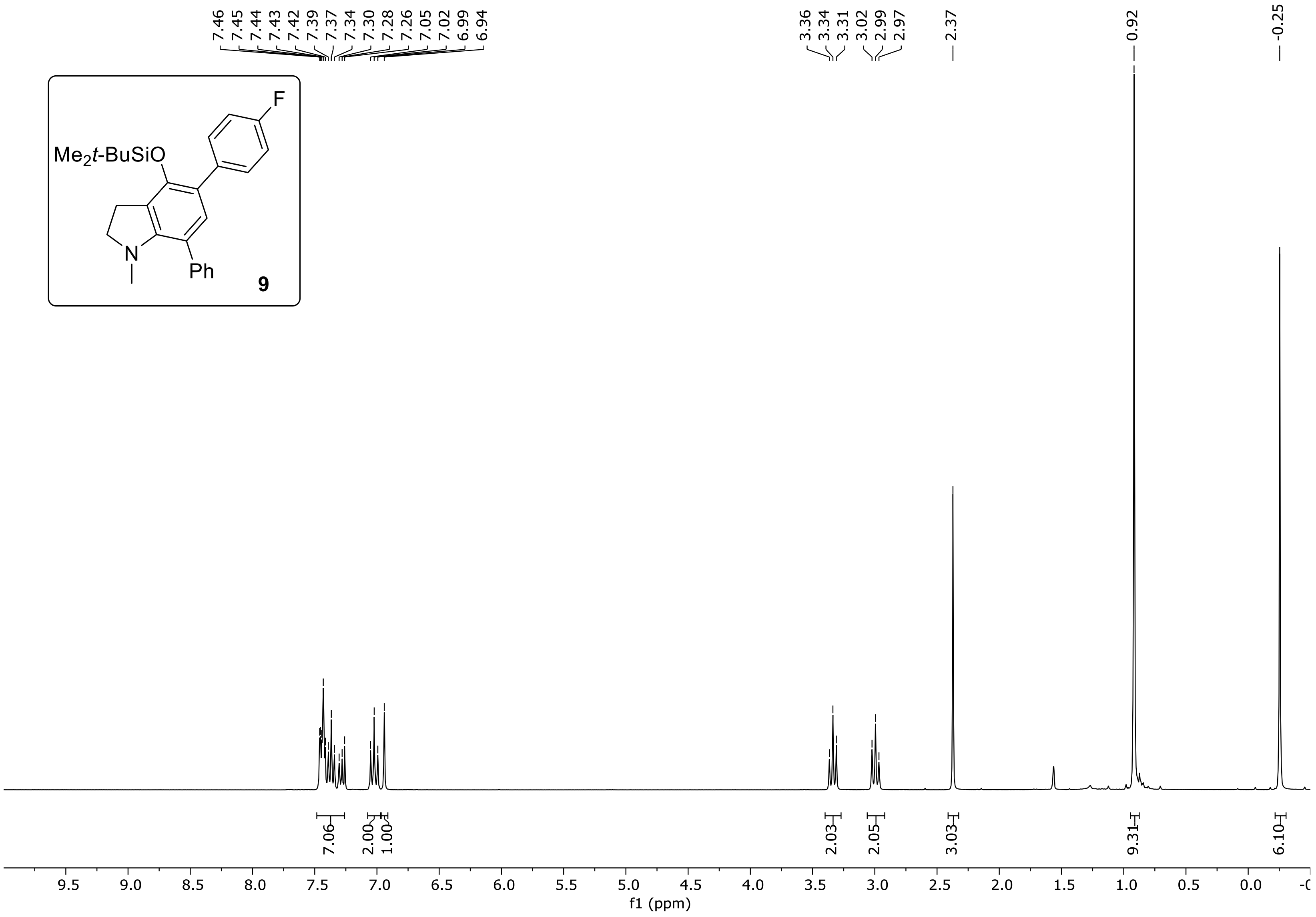

Figure S249: ^{13}C NMR of compound **9** in $CDCl_3$ at 75.4 MHz.

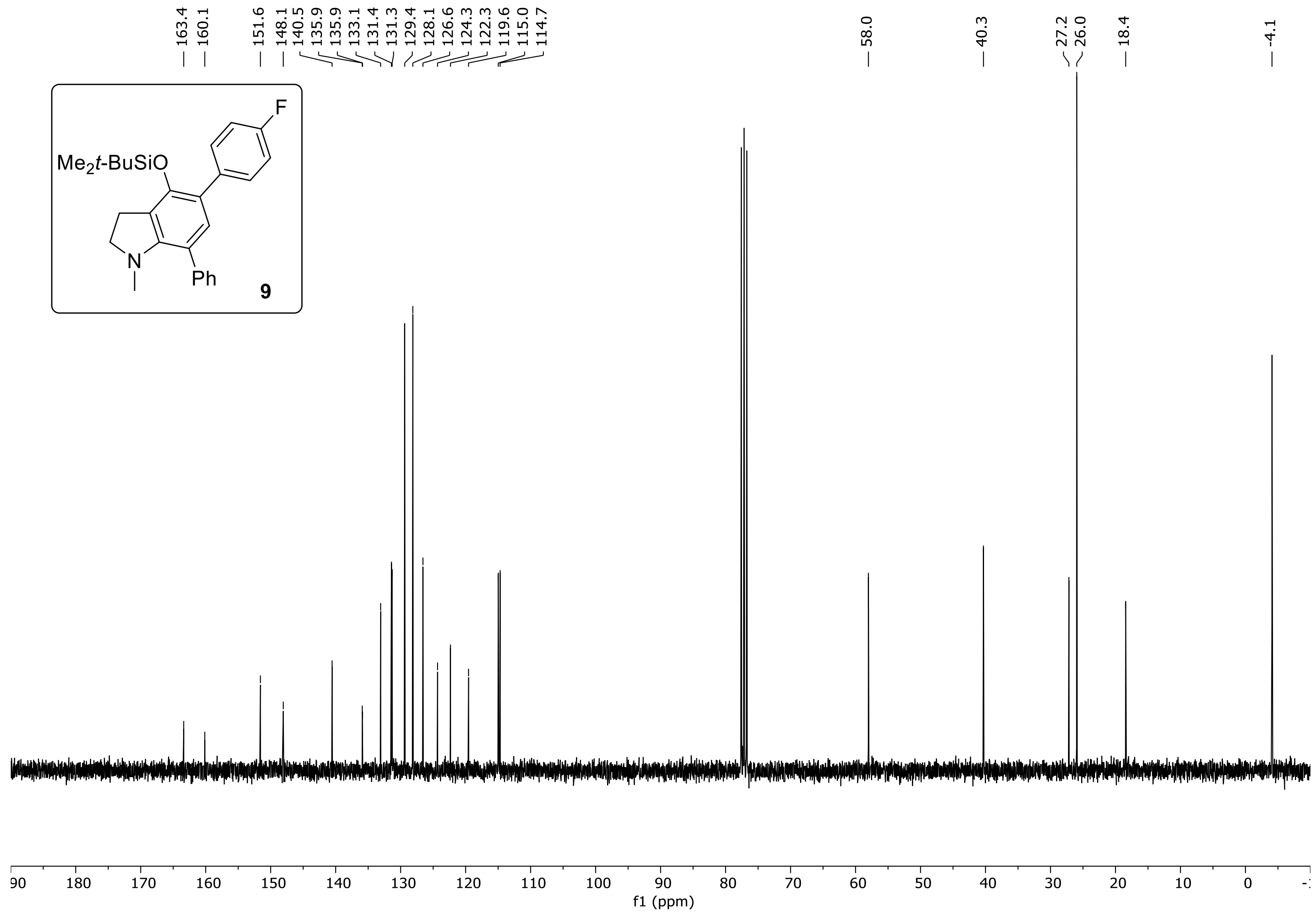

Figure S250: ^{1}H NMR of compound **10** in $CDCl_3$ at 300 MHz.

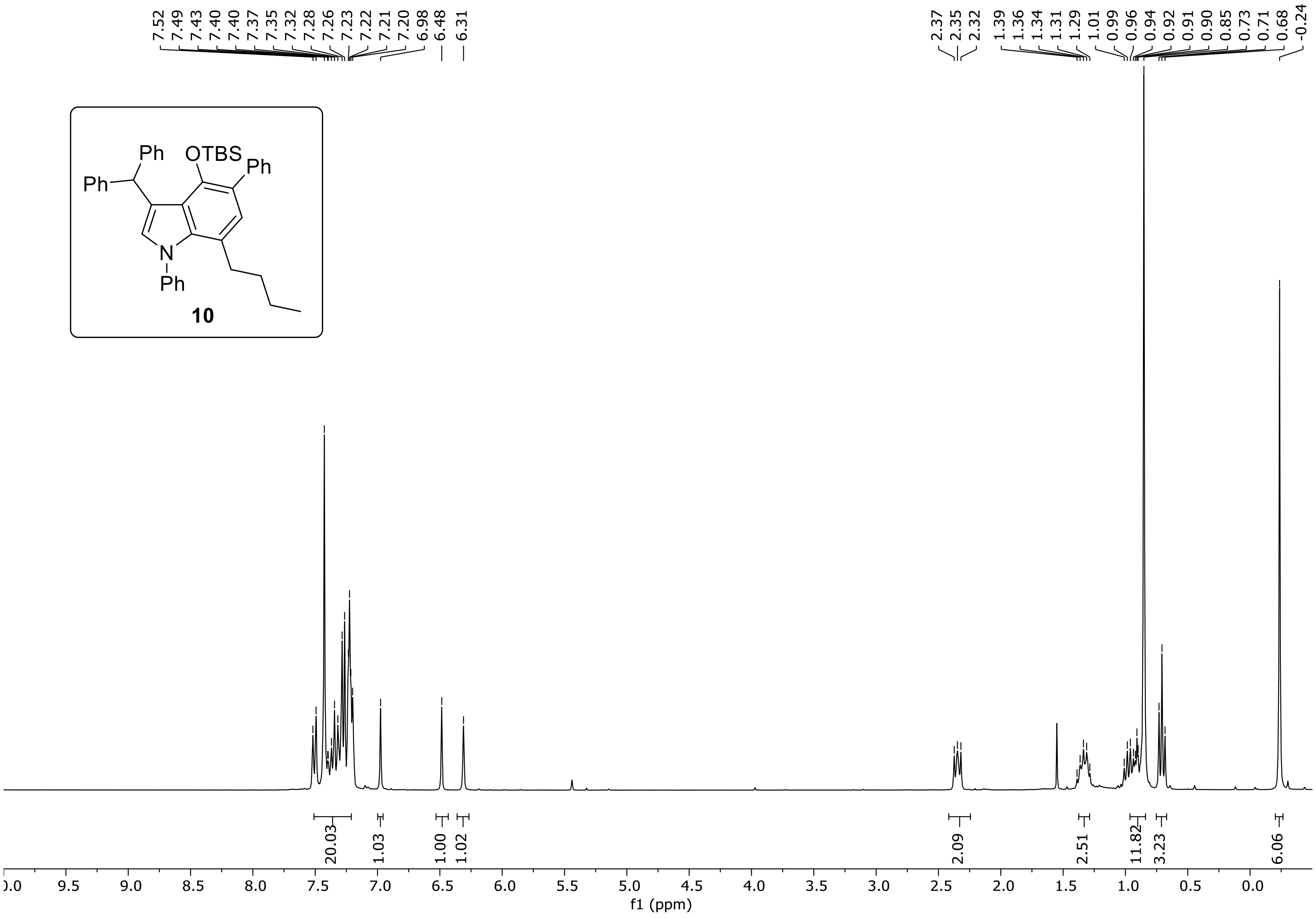

Figure S251: ^{13}C NMR of compound **10** in $CDCl_3$ at 75.4 MHz.

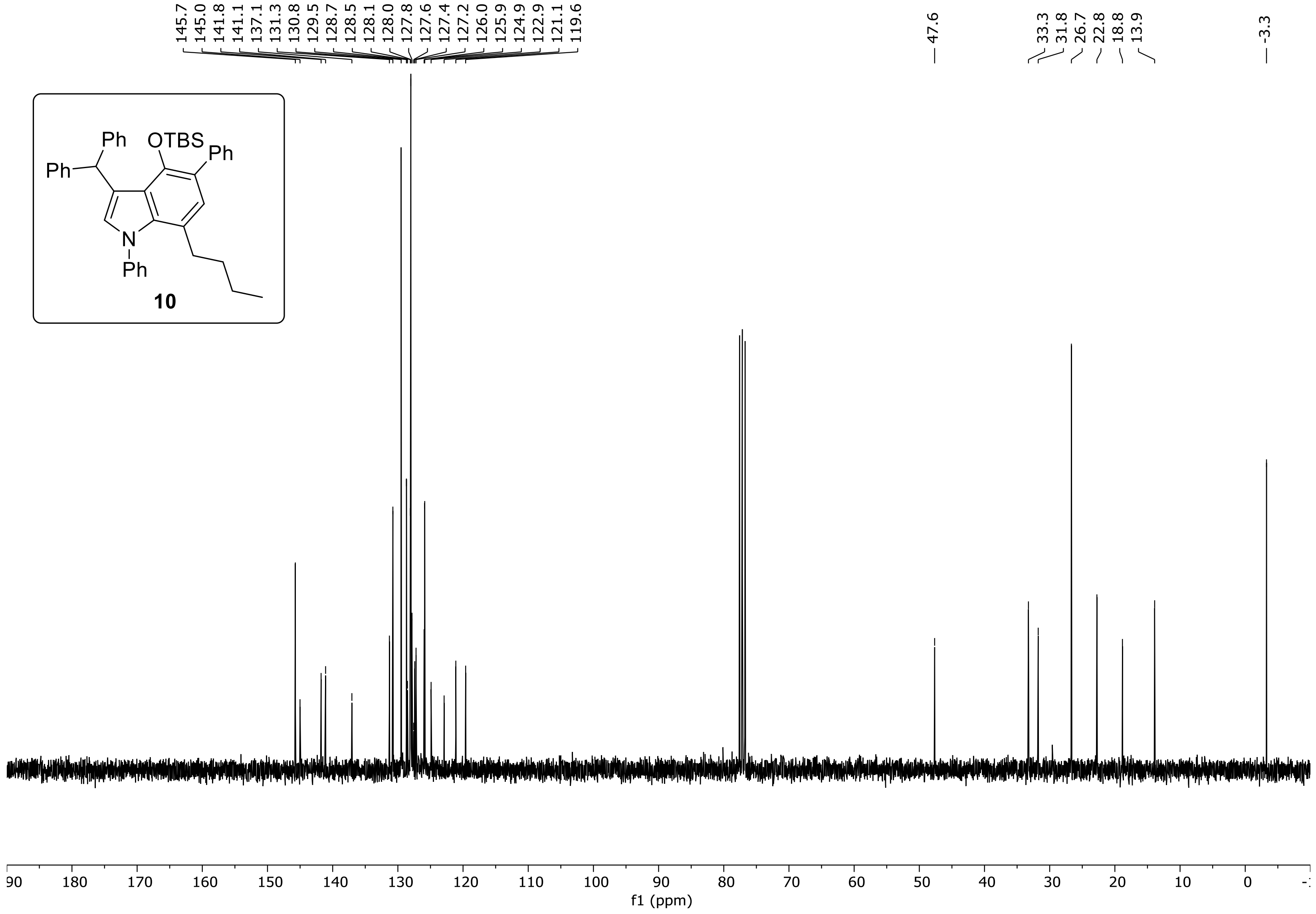

Supplement: Supplementary file 1 — ol4c01581_si_001.pdf [file ol4c01581_si_001.pdf]
