# Supplementary figures and images for: Gold-Catalyzed “Back-to-Front” Synthesis of 4-Silyloxyindoles
Source: Org Lett. 2024 Jun 5;26(23):4969–74. doi: 10.1021/acs.orglett.4c01581 (PMC11187626; doi:10.1021/acs.orglett.4c01581)

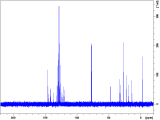

Supplement: Supplementary file 2 — ol4c01581_si_002.zip [file ol4c01581_si_002.zip › 10/C NMR/pdata/1/thumb.png]

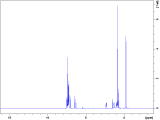

Supplement: Supplementary file 2 — ol4c01581_si_002.zip [file ol4c01581_si_002.zip › 10/H NMR/pdata/1/thumb.png]

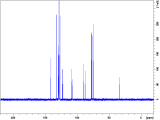

Supplement: Supplementary file 2 — ol4c01581_si_002.zip [file ol4c01581_si_002.zip › 1aa/C NMR/pdata/1/thumb.png]

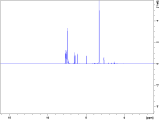

Supplement: Supplementary file 2 — ol4c01581_si_002.zip [file ol4c01581_si_002.zip › 1aa/H NMR/pdata/1/thumb.png]

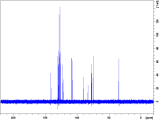

Supplement: Supplementary file 2 — ol4c01581_si_002.zip [file ol4c01581_si_002.zip › 1ab/C NMR/pdata/1/thumb.png]

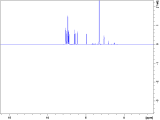

Supplement: Supplementary file 2 — ol4c01581_si_002.zip [file ol4c01581_si_002.zip › 1ab/H NMR/pdata/1/thumb.png]

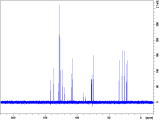

Supplement: Supplementary file 2 — ol4c01581_si_002.zip [file ol4c01581_si_002.zip › 1af/C NMR/pdata/1/thumb.png]

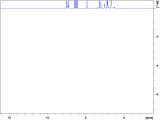

Supplement: Supplementary file 2 — ol4c01581_si_002.zip [file ol4c01581_si_002.zip › 1af/H NMR/pdata/1/thumb.png]

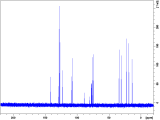

Supplement: Supplementary file 2 — ol4c01581_si_002.zip [file ol4c01581_si_002.zip › 1ah/C NMR/pdata/1/thumb.png]

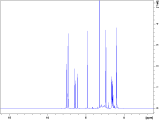

Supplement: Supplementary file 2 — ol4c01581_si_002.zip [file ol4c01581_si_002.zip › 1ah/H NMR/pdata/1/thumb.png]
