# Supplementary material for: Gold-Catalyzed “Back-to-Front” Synthesis of 4-Silyloxyindoles
Source: Org Lett. 2024 Jun 5;26(23):4969–74. doi: 10.1021/acs.orglett.4c01581 (PMC11187626; doi:10.1021/acs.orglett.4c01581)
Supplement: Supplementary file 2 — ol4c01581_si_002.zip [file ol4c01581_si_002.zip › 3ah/H NMR/pdata/1/email_MAM-859-F3_10_1.pdf]

UNIVERSIDAD DE BURGOS | Vicerrectorado de Investigación, Transferencia e Innovación

PARQUE CIENTÍFICO TECNOLÓGICO PCT

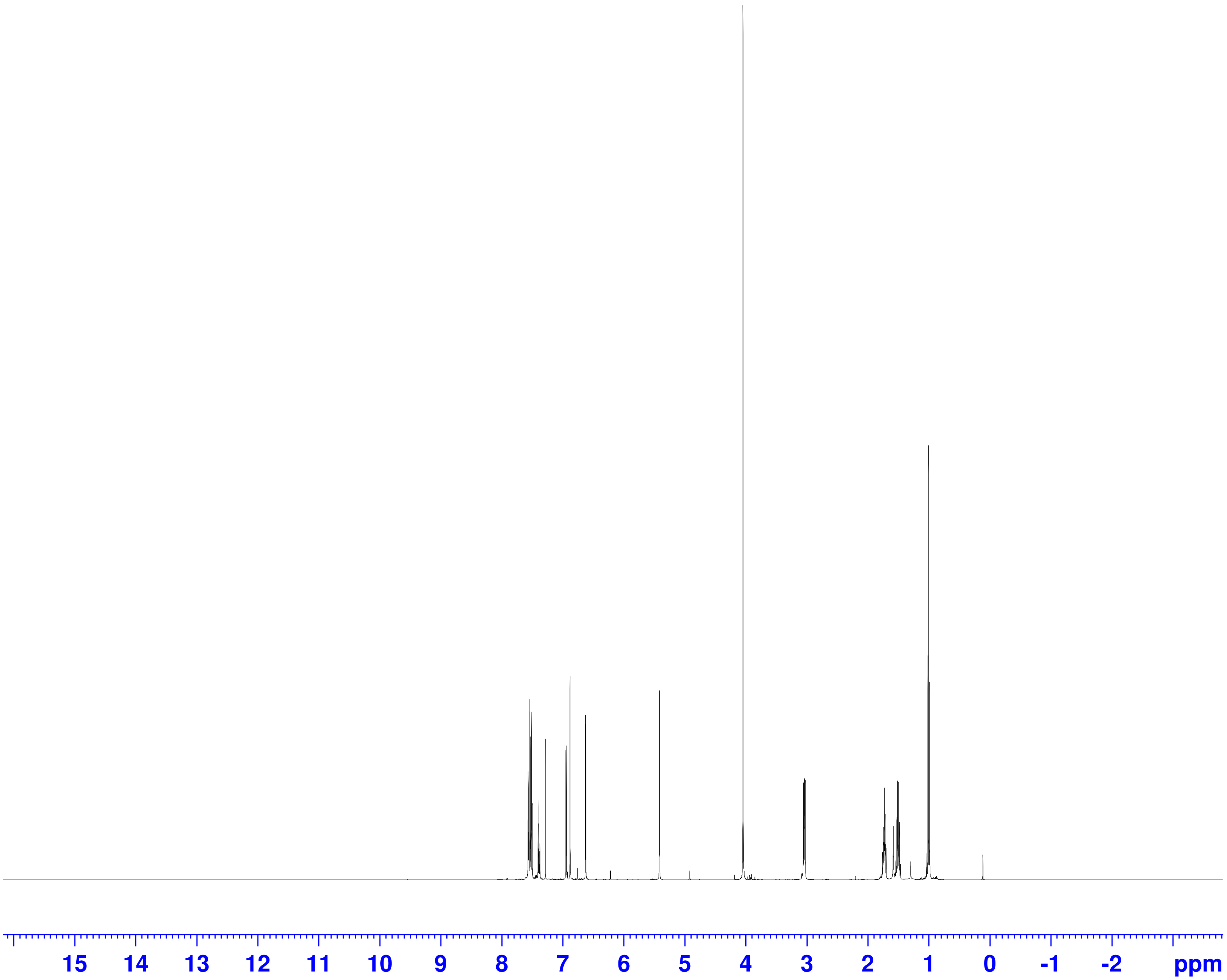

Current Data Parameters

NAME	MAM-859-F3
EXPNO	10
PROCNO	1

F2 - Acquisition Parameters

Date_	20230512
Time	2.58 h
INSTRUM	AV NEO
PROBHD	Z151574_0146 (
PULPROG	zg30
TD	65536
SOLVENT	CDCl3
NS	64
DS	2
SWH	10000.000 Hz
FIDRES	0.305176 Hz
AQ	3.2767999 sec
RG	101
DW	50.000 usec
DE	12.17 usec
TE	298.0 K
D1	1.00000000 sec
TD0	1
SFO1	500.1630885 MHz
NUC1	1H
P0	2.60 usec
P1	7.81 usec
PLW1	26.31999969 W

F2 - Processing parameters

SI	65536
SF	500.1600000 MHz
WDW	EM
SSB	0
LB	0.30 Hz
GB	0
PC	1.00
