# Supplementary material for: Gold-Catalyzed “Back-to-Front” Synthesis of 4-Silyloxyindoles
Source: Org Lett. 2024 Jun 5;26(23):4969–74. doi: 10.1021/acs.orglett.4c01581 (PMC11187626; doi:10.1021/acs.orglett.4c01581)
Supplement: Supplementary file 2 — ol4c01581_si_002.zip [file ol4c01581_si_002.zip › 4ah/H NMR/pdata/1/email_MAM-859-F2_10_1.pdf]

UNIVERSIDAD DE BURGOS | Vicerrectorado de Investigación, Transferencia e Innovación

PARQUE CIENTÍFICO TECNOLÓGICO PCT

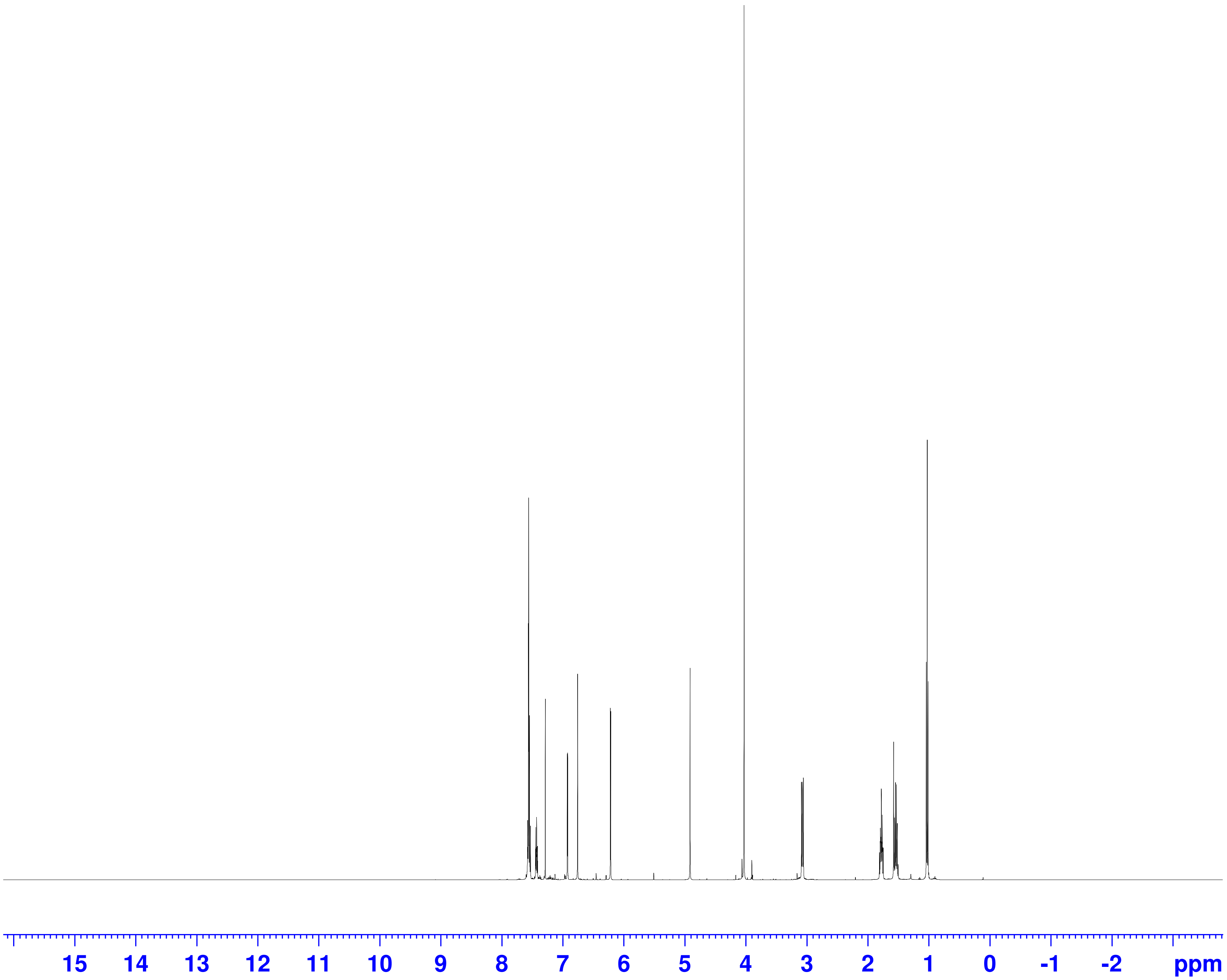


Current Data Parameters
NAME MAM-859-F2
EXPNO 10
PROCNO 1

F2 - Acquisition Parameters
Date_ 20230511
Time 17.44 h
INSTRUM AV NEO
PROBHD Z151574_0146 (
PULPROG zg30
TD 65536
SOLVENT CDCl3
NS 64
DS 2
SWH 10000.000 Hz
FIDRES 0.305176 Hz
AQ 3.2767999 sec
RG 101
DW 50.000 usec
DE 12.17 usec
TE 298.0 K
D1 1.00000000 sec
TD0 1
SFO1 500.1630885 MHz
NUC1 1H
P0 2.60 usec
P1 7.81 usec
PLW1 26.31999969 W

F2 - Processing parameters
SI 65536
SF 500.1600000 MHz
WDW EM
SSB 0
LB 0.30 Hz
GB 0
PC 1.00
