# Supplementary material for: Gold-Catalyzed “Back-to-Front” Synthesis of 4-Silyloxyindoles
Source: Org Lett. 2024 Jun 5;26(23):4969–74. doi: 10.1021/acs.orglett.4c01581 (PMC11187626; doi:10.1021/acs.orglett.4c01581)
Supplement: Supplementary file 2 — ol4c01581_si_002.zip [file ol4c01581_si_002.zip › S2g/C NMR/pdata/1/email_MAM-1016-cz_20_1.pdf]

PCT de Universidad de Burgos
C13
PCT-D02-C13 CDCl3 {D:\PCT} PCT 11

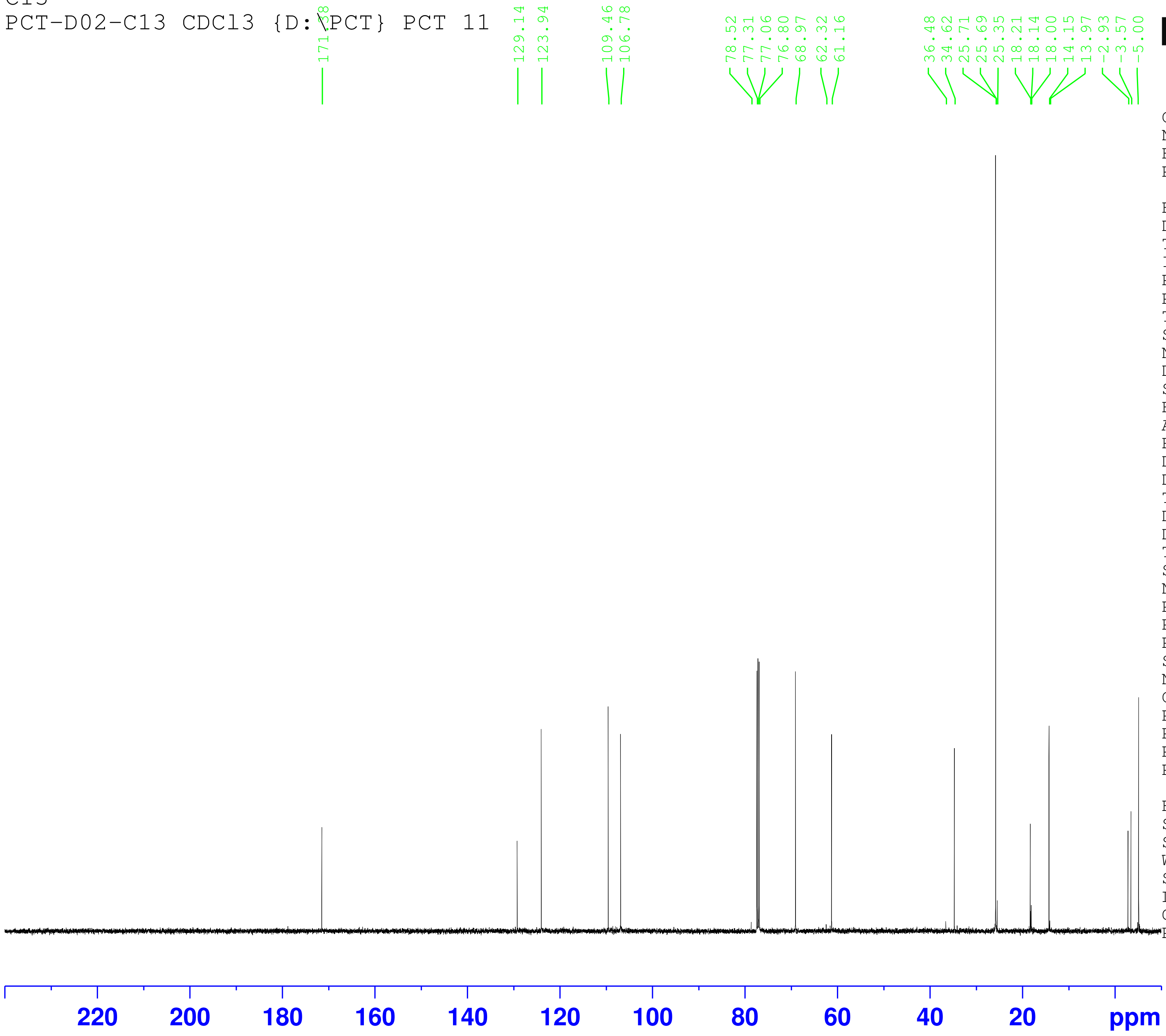

Current Data Parameters

NAME	MAM-1016-cz
EXPNO	20
PROCNO	1

F2 - Acquisition Parameters

Date_	20231106
Time	17.13 h
INSTRUM	AV NEO
PROBHD	Z151574_0146 (
PULPROG	zgpg30
TD	65536
SOLVENT	CDCl3
NS	100
DS	4
SWH	32679.739 Hz
FIDRES	0.997306 Hz
AQ	1.0027008 sec
RG	101
DW	15.300 usec
DE	6.50 usec
TE	298.0 K
D1	2.00000000 sec
D11	0.03000000 sec
TD0	1
SFO1	125.7791662 MHz
NUC1	13C
P0	3.00 usec
P1	9.00 usec
PLW1	99.70600128 W
SFO2	500.1620006 MHz
NUC2	1H
CPDPRG[2	waltz65
PCPD2	80.00 usec
PLW2	25.56200027 W
PLW12	0.25562000 W
PLW13	0.12858000 W

F2 - Processing parameters

SI	32768
SF	125.7653220 MHz
WDW	EM
SSB	0
LB	1.00 Hz
GB	0
PC	1.40
